# *Proteus mirabilis* inhibits cancer growth and pulmonary metastasis in a mouse breast cancer model

**DOI:** 10.1371/journal.pone.0188960

**Published:** 2017-12-05

**Authors:** Hong Zhang, Hongxiu Diao, Lixin Jia, Yujing Yuan, Douglas H. Thamm, Huanan Wang, Yipeng Jin, Shimin Pei, Bin Zhou, Fang Yu, Linna Zhao, Nan Cheng, Hongchao Du, Ying Huang, Di Zhang, Degui Lin

**Affiliations:** 1 Department of Veterinary Clinical Science, College of Veterinary Medicine, China Agricultural University, Beijing, China; 2 Department of Clinical Sciences, College of Veterinary Medicine and Biomedical Sciences, Colorado State University, Fort Collins, Colorado, United States of America; 3 Department of Veterinary, College of Animal Sciences, Zhejiang University, Hangzhou City, Zhejiang, China; 4 The Department of Veterinary Medicine, Hainan University, Haikou, Hainan, China; 5 The College of Animal Science and Technology, Zhejiang Agriculture and Forestry University, Hangzhou, Zhejiang, China; University of South Alabama Mitchell Cancer Institute, UNITED STATES

## Abstract

A variety of bacteria have been used as agents and vectors for antineoplastic therapy. A series of mechanisms, including native bacterial toxicity, sensitization of the immune system and competition for nutrients, may contribute to antitumor effects. However, the antitumor effects of *Proteus* species have been minimally studied, and it is not clear if bacteria can alter tumor hypoxia as a component of their antineoplastic effect. In the present study, *Proteus mirabilis* bacteria were evaluated for the ability to proliferate and accumulate in murine tumors after intravenous injection. To further investigate the efficacy and safety of bacterial injection, mice bearing 4T1 tumors were treated with an intravenous dose of 5×10^7^ CFU *Proteus mirabilis* bacteria via the tail vein weekly for three treatments. Histopathology, immunohistochemistry (IHC) and western analysis were then performed on excised tumors. The results suggested *Proteus mirabilis* localized preferentially to tumor tissues and remarkably suppressed the growth of primary breast cancer and pulmonary metastasis in murine 4T1 models. Results showed that the expression of NKp46 and CD11c was significantly increased after bacteria treatment. Furthermore, tumor expression of carbonic anhydrase IX (CA IX) and hypoxia inducible factor-1a (HIF-1a), surrogates for hypoxia, was significantly lower in the treated group than the control group mice as assessed by IHC and western analysis. These findings demonstrated that *Proteus mirabilis* may a promising bacterial strain for used against primary tumor growth and pulmonary metastasis, and the immune system and reduction of tumor hypoxia may contribute to the antineoplastic and antimetastatic effects observed.

## Introduction

Nearly 150 years ago, William B. Coley first found that *Streptococcus pyrogens* can be actively used in the treatment of cancer [1]. Since then, several bacteria, including *Salmonella typhimurium (S*. *typhimurium)* [2], *Clostridium* [3], and *Escherichia Coli (E*. *coli)* [4], have been found to specifically target tumors with limited toxicity, as well as being used as vectors for gene delivery [5]. There are also clinical trials in progress to confirm the efficacy of bacteria for the treatment of canine and human patients with cancer [6–8]. However, successful bacterial cancer treatment could cooperatively use a collection of bacterial strains designed for specialized purposes [9]. Therefore, it is essential to explore a variety of bacterial strains that may have activity against different tumor types or differing mechanisms of action.

Hypoxia is a prominent feature of solid tumors and contributes to several important processes: angiogenesis, epithelial-mesenchymal transition, migration/invasion, maintenance of cancer stem cells, metastasis, immune surveillance and resistance to chemotherapy and radiotherapy [10, 11]. It is known that some particular anaerobic and facultative anaerobic bacteria can selectively adapt to hypoxic tumor-specific microenvironments to replicate and/or preferentially accumulate in tumor tissues, leading to inhibition of tumor growth and metastasis. Cancer regression by bacteria depends on a complex set of mechanisms, including native bacterial toxicity, sensitization of the immune system and competition for nutrients [12]; however, it is still unknown if bacteria can not only colonize in but alter the hypoxic tumor microenvironment during colonization.

With the development of early detection and more effective therapeutic regimens, the mortality rate in breast cancer patients has been decreasing; however, breast cancer still ranks as the second leading cause of cancer-related deaths in women in the United States [13], primarily owing to the development of metastasis. So it is still urged to develop more effective therapies to treat breast cancer. Zhao and co-workers [14] demonstrate that a leucine-arginine auxotrophic strain of *S*. *typhimurium* can induce regression of breast tumors and metastasis in orthotopic nude mouse models. Intratumoral injection of *Clostridium* novyi-NT spores has been shown to inhibit cancer growth in both dogs and one human patient [15]. Therefore, bacteria therapy is possibly a novel strategy for breast cancer treatment.

*Proteus mirabilis* (*P*. *mirabilis*) is Gram-negative and a member of the family Enterobacteriaceae. It has been reported by researchers that *P*. *mirabilis* RMS-203 (Murata strain) possessed oncolytic effects in murine tumors [16]. In this study, we investigated the effects of a different *P*. *mirabilis* strain on breast cancer growth and pulmonary metastasis in murine models and evaluated treatment’s impact on immune system and tumor hypoxia.

## Materials and methods

### Animal and ethics statement

All animal studies were approved by China Agricultural University Laboratory Animal Welfare and Animal Experimental Ethical Committee (Approval ID: CAU 2015121701–1). 5-week-old female BALB/c mice used in this study were obtained from Beijing Vital River Laboratory Animal Technology Co., Ltd. Animal care and use were conducted with the rules of the laboratory animal welfare and animal experiment ethics issued by China Agricultural University Laboratory Animal Welfare and Animal Experimental Ethical Committee. All animals were housed under specific pathogen-free conditions in a temperature-controlled room, and fed with a regular diet. They were carefully observed on a daily basis and would be humanely sacrificed if they met the following endpoint criteria: prostration, significant bodyweight loss, difficulty breathing, and rotational motion. Mice were sacrificed by cervical dislocation.

### Bacteria and cell culture

*Proteus mirabilis* Hauser (ATCC^®^ 35659) was obtained from American Type Culture Collection (ATCC, Manassas, VA, USA) and grown in LB broth at 37°C. The murine 4T1 and the human MCF7, MDA-MB-231 breast tumor cell lines, and Chinese hamster ovary (CHO) cells were purchased from ATCC. The 4T1 cells were grown in RPMI-1640 (Gibco, USA) medium with 10% fetal bovine serum (FBS, Gibco, USA), and penicillin (100 units/mL) and streptomycin (100 units/mL). MDA-MB-231 cells, MCF7 cells, and CHO cells were grown in DMEM (Gibco, USA) medium with 10% FBS, plus penicillin and streptomycin. All cells were incubated at 37°C in a humidified 5% CO_2_ environment.

### Cell adherence and growth inhibition of bacteria *in vitro*

According to previous methods [14], tumor cells and CHO cells were grown in 24-well tissue plates at 10^4^ cells per well and allowed to adhere overnight. Grown to late-log phase in LB broth, *P*. *mirabilis* was added at a concentration of 5×10^7^ CFU/mL. Triplicate wells were used for each group. After 2 hours incubation, the cells were rinsed five times with 1 mL phosphate-buffered saline (PBS). To evaluate the bacterial adherence, cells were incubated with 0.2 mL 0.1% Triton X-100 for 10 minutes at 37°C in a constant temperature incubator. Then 0.8 mL LB broth was added to each sample and forcefully mixed. The mixture was grown on LB agar medium for 24 hours and then the number of bacterial colonies was counted. For growth inhibition assay, cell lines were plated in 96-well plates at a density of 1× 10^4^ cells per well, to which serial of dilutions of bacteria were added in quintuplicate. Cells were incubated for 0, 24, 48, 72, and 96 hours at 37°C. Relative viable cell number was determined using Typan Blue (Solarbio, Beijing, China) according to manufacturer directions. Relative viable cell number was then expressed as a percentage of live cells. Each experiment was repeated at least three times and mean [± error (SE)] calculated.

### Colony formation assay

Tumor cells and CHO cells were plated in 6-well plates at a density of 10^4^ cells per well. After 24 hours, bacteria were added to the tumor cells at a concentration of 5×10^7^ CFU/mL, and then cultured for 2 hours at 37°C in a humidified atmosphere of 5% CO_2_. Then the cells were rinsed five times with 1 mL PBS, and cultured for 7 days with fresh DMEM or RPMI-1640 medium containing 1 mg/mL ampicillin. The attached cells were stained with 0.1% (W/V) crystal violet (Solarbio, Beijing, China) and evaluated microscopically.

### Body distribution of *P*. *mirabilis* in tumor-bearing mice

Female 5-week-old BALB/c mice were used in this study. A suspension of 10^6^ 4T1 cells in 0.2 mL PBS was transplanted subcutaneously into the left mammary fat pad of mice. When the tumors were about 8–10 mm in diameter, bacteria were injected via the tail vein at a dose of 5×10^7^ CFU per mouse. To investigate the biodistribution of bacteria, mice (n = 5) were euthanized for collection of tumors and other organs, including the heart, liver, spleen, lung, and kidney 0, 6, 24, 48, 72 and 96 hours following treatment. All tissues were weighed and a fivefold volume of cold PBS added, and then minced and homogenized with a tissue homogenizer under sterile conditions. Tissue homogenates were diluted at different concentrations and 10 uL aliquots plated in LB agar in 6 cm Petri dishes at 37°C. Bacterial colonies were counted after 24 hours. Sections of fresh tumor tissues were fixed in 10% (v/v) neutral-buffered formalin and dehydrated in series of ethanol and xylene, and then embedded in paraffin wax. Sections (3 μm) were cut with a microtome (Leica, Germany) and stained with hematoxylin and eosin (H&E) according to standard protocols. The sections of tumor tissues were examined using a microscope.

### Therapeutic effect of *P*. *mirabilis* in tumor-bearing mice

Five-week-old female BALB/c mice were implanted with 4T1 tumor cells as described above. When the tumors became 8–10 mm in diameter, mice were injected with bacteria at a dose of 5×10^7^ CFU per mouse or PBS (control group) via the tail vein weekly for three injections. Mice were administered 100 mg/kg cyclophosphamide (CTX) by intraperitoneal injection once every other day over 6 days (i.e., three injections) as a drug control group [17]. There were 6 mice per group. Tumor volume and body weight were measured every three days, and tumor volume was estimated by measuring longitudinal cross-section (a) and transverse section (b) according to the formula V = (a×b^2^)/2 [18]. After 21 days of treatment, mice were euthanized to collect lungs, kidneys, and primary tumors, then lungs and tumors were weight. Fresh intact lung and kidney tissues were fixed in 10% (v/v) neutral-buffered formalin for 24 hours and then the number of metastases on the lung surface was counted. Lung and kidney tissues were dehydrated in series of ethanol and xylene and embedded in paraffin wax. Sections (3 μm) were cut and stained with H&E according to standard protocols. The sections were examined using a microscope. To investigate the liver function, the bloods were drawn from mice after 0, 72 hours and 21 days bacteria administration, then detected using Catalyst Dx Chemistry Analyzer (IDEXX, ME, USA).

### Cytokine assays

To evaluate the effect of bacterial therapy on serum cytokine levels, the RayBiotech Mouse Cytokine Antibody Array C1 was purchased from RayBiotech (Norcross, GA) and used according to the manufacturer’s instructions. The blood was collected from mice (n = 5) at 24 hours after bacterial or PBS injection. After blocking, membranes were incubated for overnight at 4°C with 10-fold diluted sera. The membranes were washed and then incubated with a biotinylated antibody cocktail for 2 hours at room temperature (RT). The membranes were washed again and incubated with HRP-streptavidin for 2 hours at RT, and finally exposed using a chemiluminescence imaging analysis system (Tanon 5200, China). The result was analyzed by Image-pro-plus software (Media Cybernetics, Washington, USA).

### Immunohistochemical analysis

Tumor and spleen tissues were fixed in 10% (v/v) neutral-buffered formalin and embedded in paraffin wax. For immunohistochemical studies, 3 μm sections were cut from each specimen and mounted on CITOGLAS^®^ adhesion microscope slides (CITOTEST, Jiangsu, China). The tumor sections were incubated overnight with primary antibodies specific for Ki-67 (ZM-0166, ZSGB-BIO, China, 1:100), CA IX (ab38898, Abcam, USA, 1:900), and HIF-1a (ab463, Abcam, USA, 1:500); the spleen tissues were stained with NKp46 (bs-2417R, Bioss, China, 1:400), CD11c (bs-2508R, Bioss, China, 1: 200), CD11b (bs-1014R, Bioss, China, 1:200), and Ly-6G (bs-2576R, Bioss, China, 1:400). The biotinylated secondary antibody was goat anti-mouse or anti-rabbit antibody IgG (ZSGB-BIO, China). Antibody binding was detected with 3, 3’ -diaminobenzidine tetrahydrochloride (DAB kit, ZSGB-BIO, China). After a final washing in distilled water, the sections were counterstained with haematoxylin, dehydrated, cleared and mounted. Images were captured with a digital microscope and analyzed by Image-pro-plus software.

### Western blotting

Tumor tissues were dissected and stored at -80°C. The frozen tumor tissues were lysed with ice-cold RIPA Lysis Buffer (P0013B, Beyotime, China) for 30 minutes on ice then homogenized with a tissue homogenizer. The lysates were centrifuged at 12,000 rpm for 20 minutes at 4°C, and the protein concentration was determined by BCA Protein Assay (Beyotime, China). Equivalent samples (20 μg protein per lane) were subjected to SDS-PAGE on 10% gel. The proteins were then transferred onto polyvinylidene fluoride (PVDF) membranes (IPVH000 10, MercKMillipore), and incubated with primary antibodies against CA IX (ab38898, Abcam, USA, 1:1500), HIF-1a (ab463, Abcam, USA, 1:500) and a-tubulin (as internal reference, sc-80350, Santa Cruz, USA, 1:500) overnight at 4°C. Then membranes were incubated with anti-mouse or anti-rabbit secondary antibody for 1 hour at RT. Primary antibody was visualized by binding horseradish peroxidase (HRP)-conjugated secondary antibody with an Electro-Chemi-Luminescence (ECL) plus kit (Thermo).

### Statistical analysis

Numerical results were expressed as mean ± SEM or mean ± SD and compared by analysis of variance or one-way ANOVA. All statistical analyses were performed using SPSS v20.0 (IBM., Chicago, IL, USA) or GraphPad Prism 5 (GraphPad Software, California, USA). In all statistical comparisons, p < 0.05 was accepted as significant differences.

## Results

### *P*. *mirabilis* has a non-specific growth inhibition effect *in vitro*

**Fig 1:**
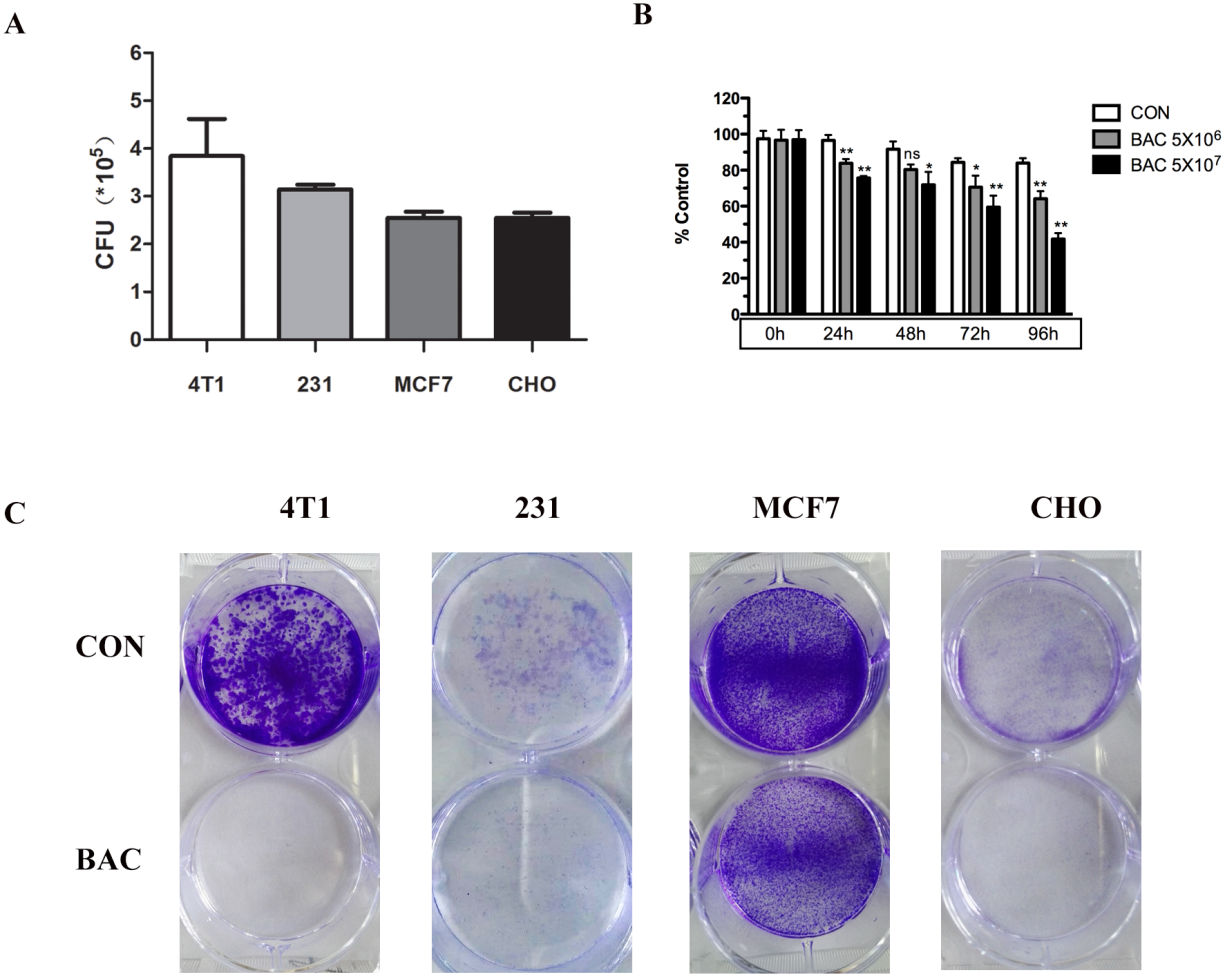
The adherence and growth inhibition by *P*. *mirabilis in vitro*. (A) *P*. *mirabilis* adhered to 4T1, MDA-MB-231, MCF7, and CHO cells *in vitro*. (B) The cells were counted by stained with Trypan Blue. Bacteria treatment showed dose- and time-dependent antiproliferative effects in 4T1 cell lines (C) In the colony formation assay, *P*. *mirabilis* inhibited colony formation of breast tumor cells and CHO cells by crystal violet staining. Data were expressed as the mean ± SD.

Human and murine breast cancer cells and CHO cells were incubated with *P*. *mirabilis* bacteria, and the number of adherent *P*. *mirabilis* was measured. The results showed *P*. *mirabilis* could attach to the tumor cells of 4T1 (3.84×10^5^ ± 1.34×10^5^ CFU), MDA-MB-231 (3.14×10^5^ ± 0.17×10^5^ CFU), MCF7 (2.55×10^5^ ± 0.22×10^5^ CFU) and CHO (2.54×10^5^ ± 0.20×10^5^ CFU) (Fig 1A). To assess the antiproliferative effect of bacteria *in vitro*, we performed growth inhibition assays by counting cells stained with Trypan Blue. Bacteria treatment demonstrated dose- and time-dependent antiproliferative effects in 4T1 cell lines (Fig 1B). Crystal violet staining suggested that bacteria inhibited colony formation of tumor cells and normal cells (Fig 1C). Taken together, those results demonstrated that the growth inhibition of bacteria was a non-specific effect *in vitro*.

### *P*. *mirabilis* distributes to tumor lesions after intravenous injection

**Fig 2:**
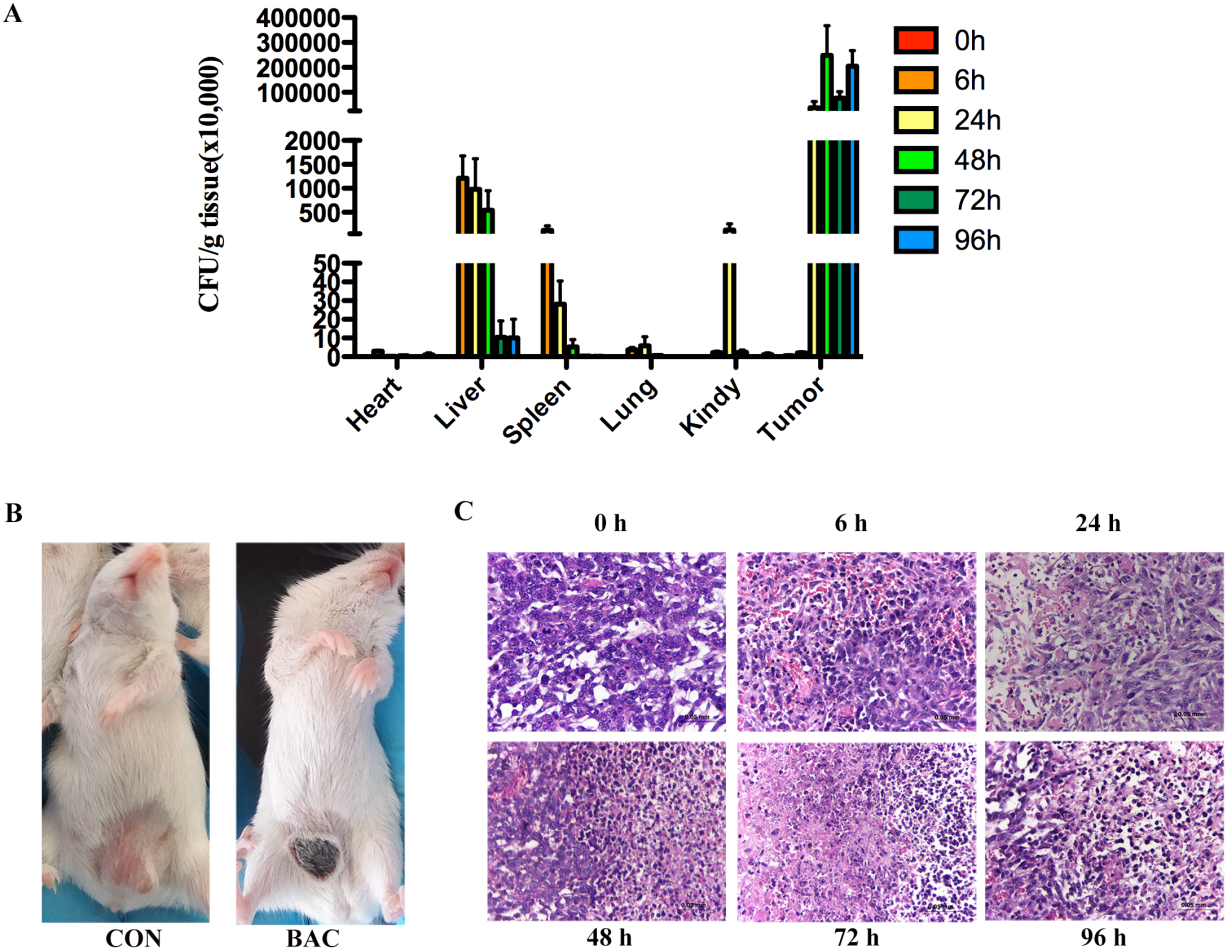
Body distribution of *P*. *mirabilis* in tumor-bearing mice. (A) The bacteria were detected in tumors and other organs, including heart, liver, spleen, lung and kidney at 0, 6, 24, 48, 72, and 96 hours after *P*. *mirabilis* injection. The number of bacteria in the liver, spleen and other organs gradually decreased with time; however, the number of bacteria in the tumor tissues increased. The means ± SEM of five mice per group were shown. (B) Surface ulceration of an allograft tumor was observed 24 hours after *P*. *mirabilis* treatment. (C) Morphological analysis of the tumor sections demonstrated tumor cell death and inflammatory cells infiltrated after bacteria intravenous injection.

To investigate the systemic distribution of *P*. *mirabilis* after intravenous injection in tumor-bearing mice, different tissue homogenates were cultured in LB agar for 24 hours at 37°C. The bacteria were mostly detected in liver and spleen at 6 hours after injection (Fig 2A). The CFU count of bacteria in the liver, spleen and other organs gradually decreased over time; however, the number of bacteria in the tumor tissues increased. At 24 hours after treatment, there were significantly more bacteria in tumor tissues than in the normal tissues. At 96 hours after injection, the number of living bacteria in the tumors was about 10^5^ and 10^6^ times those in livers and spleens (Fig 2A). These results suggested that bacteria specifically accumulated in tumor tissues with time. Grossly, the allograft tumor scabbed after 24 hours treatment (Fig 2B). The microscopic evaluation demonstrated the treatment could cause significant tumor cell death and infiltrated inflammatory cells after bacteria intravenous administration (Fig 2C).

### *P*. *mirabilis* inhibits 4T1 allograft growth *in vivo*

**Fig 3:**
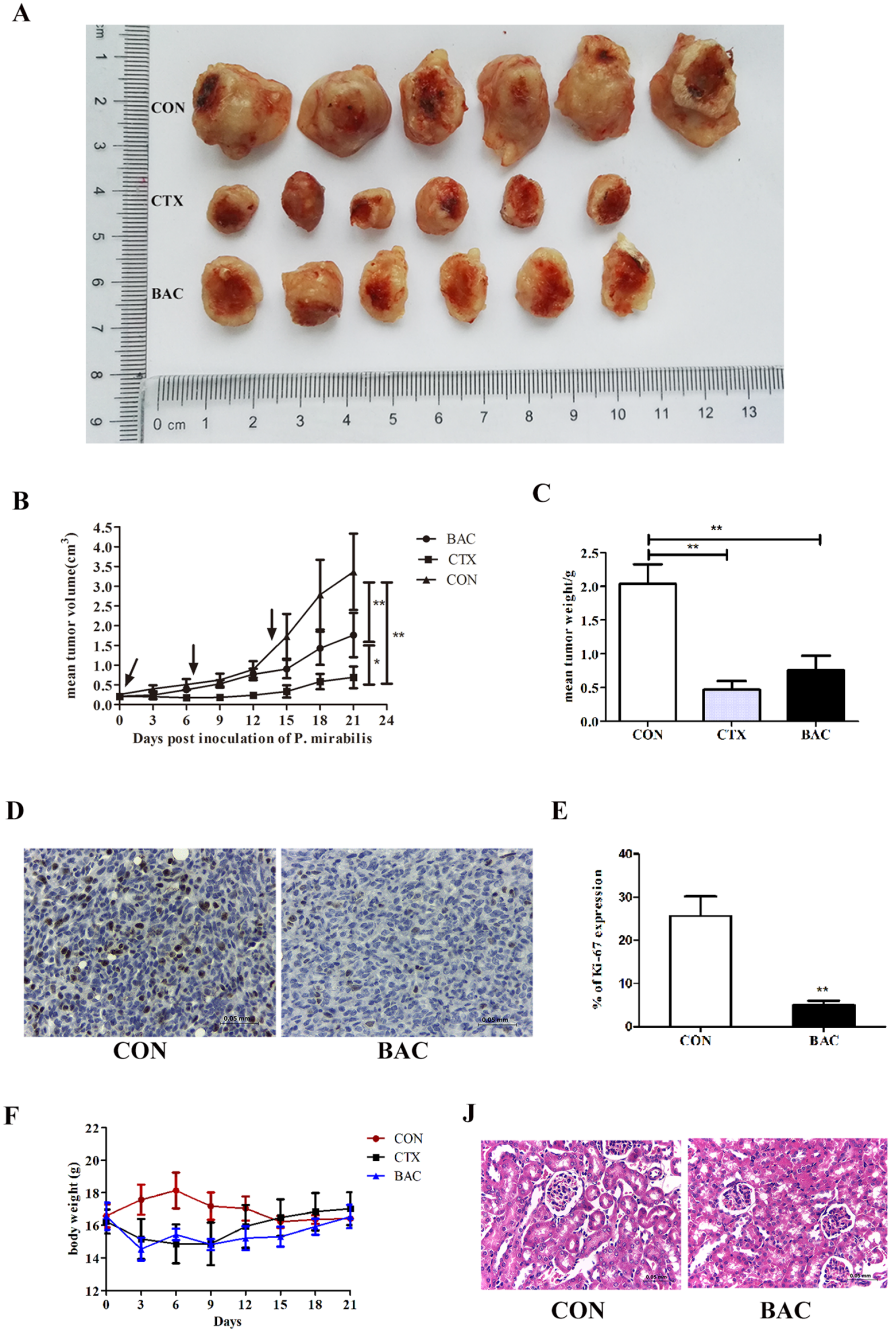
The therapeutic effect of *P*. *mirabilis* in 4T1 solid tumor model. (A) Bacterial treatment suppressed tumor growth at the end of the experiment. Quantitative analysis demonstrated that the volume (B) and weight (C) of tumors in the *P*. *mirabilis* and CTX treatment groups were significantly less than those in the control group (p < 0.05). Corresponding to the images in (D) and (E) quantitative analysis of Ki-67 staining further confirmed that tumor proliferation was significantly inhibited in the treatment group (p < 0.05). (F) The body weights of mice significantly decreased at the beginning of treatment, but at the end of the experiment, they were not significantly different among these groups. (J) Morphological analysis of mice kidney tissues: there were no obvious differences in morphology between the *P*. *mirabilis* treatment group and the PBS control group. Data were expressed as mean ± SD.

The therapeutic effect of *P*. *mirabilis* was investigated in orthotopic 4T1 tumors. As shown in Fig 3A, bacterial treatment significantly suppressed tumor growth. The volume and weight of the allograft tumors in treatment groups were significantly decreased compared with those in the PBS control group (p < 0.05), and the tumor volume in the CTX treatment group was smaller than those in the bacteria treatment group (p < 0.05); however, there were no significant differences of tumor weight between the *P*. *mirabilis* treatment group and the CTX control group (Fig 3B and 3C). Immunohistochemistry staining of Ki-67 further confirmed that tumor proliferation was significantly inhibited in the *P*. *mirabilis* treatment group compared with the PBS control group (Fig 3D and 3E) (p < 0.05). To figure out the effect of LPS on the tumor growth inhibition, the LPS was extracted from *P*. *mirabilis* and injected to mice bearing tumor. The result showed LPS had no impact on the tumor growth (S1 Fig). The safety of bacterial treatment is one of the most important factors when bacteria treatment will be applied in clinic. Therefore, body weights of mice, liver function test and histopathology of kidney were employed. Body weights of mice were significantly decreased at the beginning of treatment in both treatment groups, but recovered and continued to increase. By the end of the experiment, there was no apparent difference in the mice body weights among the groups (Fig 3F). Then we checked the liver function by doing the blood biochemical examination. Results showed aspartate transaminase (AST) and alanine transaminase (ALT) were elevated after 72 hours bacteria injection, but there were no differences between the control group and the group after 21 days bacteria treatment; other liver indicators were no differences among those groups (Table 1). To further observe the toxic of *P*. *mirabilis* treatment, kidney tissue sections were examined microscopically. All kidney cell nucleoli were clearly visible and no degeneration, bleeding or necrosis in the kidney cells was observed; there were no obvious differences in tubular or glomerular morphology between the *P*. *mirabilis* treatment group and the PBS control group (Fig 3J).

**Table 1:** Liver function detection of mice after *P*. *mirabilis* treatment.

	Control with PBS injection	72 hours after bacteria treatment	21 Days after bacteria treatment
TP (g/L)	53.93 ± 4.92	57.08 ± 5.26	60.93 ± 6.05
ALB (g/L)	30.05 ± 2.14	29.22 ± 3.42	30.486 ± 2.05
Glob (g/L)	23.87 ± 3.05	26.26 ± 6.58	30.45 ± 4.06
A/G	1.27 ± 0.12	1.17 ± 0.31	1.01 ± 0.07
ALT (U/L)	35.16 ± 5.85	139.76 ± 133.39	37.83 ± 10.12
AST (U/L)	309.8 ± 20.28	735.6 ± 395.74	289.8 ± 36.77

Note: TP: Total Protein; ALB: Albumn; Glob: Globulin; A/G: ALB/Glob; ALT: Alanine Transaminase; AST: Aspartate Transaminase.

### *P*. *mirabilis* suppresses spontaneous pulmonary metastases *in vivo*

**Fig 4:**
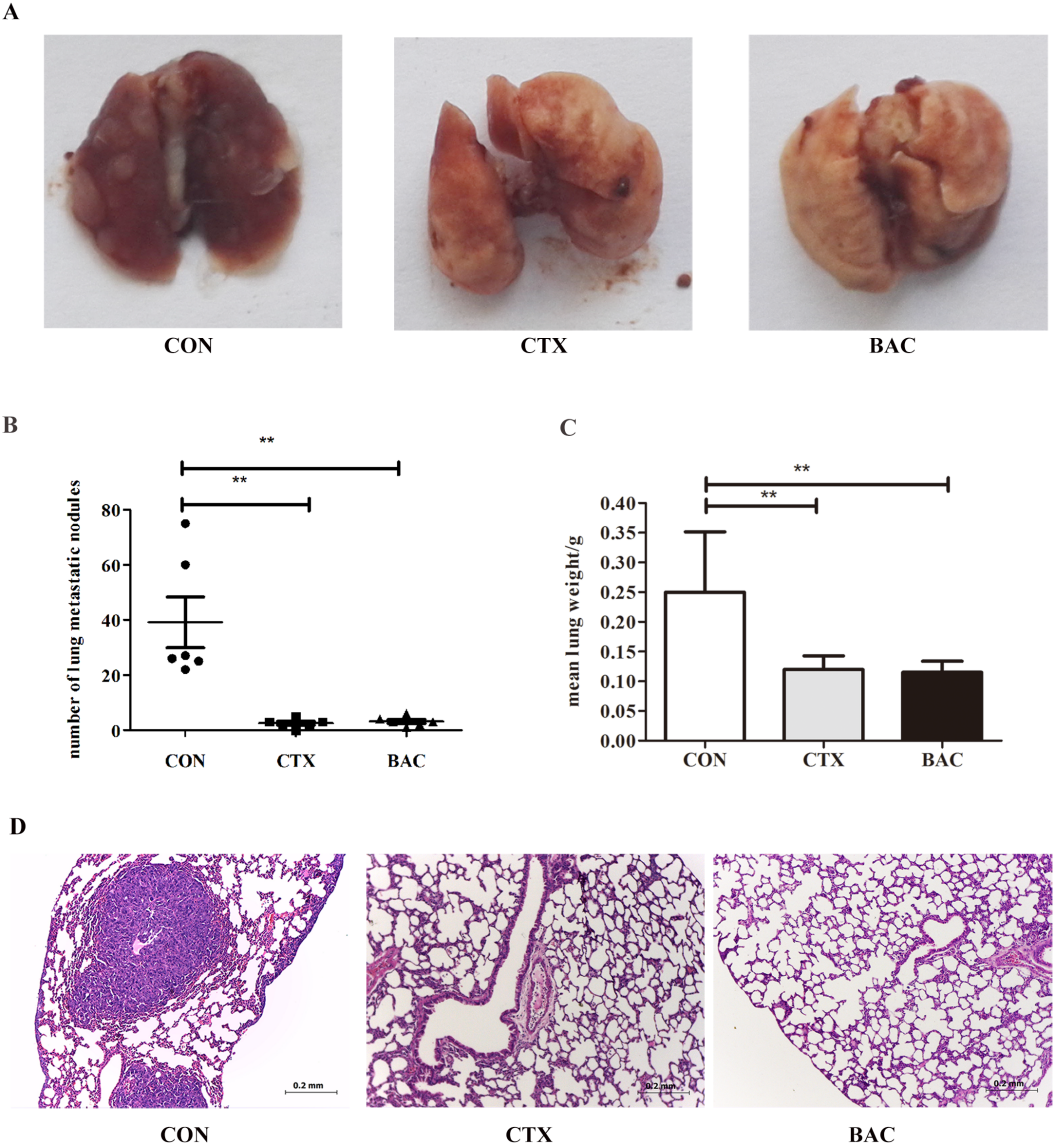
*P*. *mirabilis* suppressed spontaneous pulmonary metastases *in vivo*. Bacterial treatment inhibited tumor spontaneous pulmonary metastases by observing fresh lung tissues (A) at the end of the experiment. (B) Quantitative statistics of metastatic foci showed significant differences between *P*. *mirabilis* treatment group and PBS control group (n = 6) (p < 0.05). (C) Quantitative statistics showed lung weight of mice in the control group was significantly higher than those in the *P*. *mirabilis* treatment group (p < 0.05). (D) Those results were further confirmed by analyzing H&E stained sections. Data were expressed as the mean ± SD.

To further explore the effect of bacteria on suppressing spontaneous pulmonary metastases, two indicators, the number of surface metastatic foci and the weight of mice lung were compared among groups (Fig 4A). The number of surface metastatic foci in the *P*. *mirabilis* treatment group (3.2 ± 1.7) was significantly less than in the PBS control group (39.2 ± 22.5, p < 0.05) (Fig 4B). There were no statistically significant differences between the *P*. *mirabilis* treatment group and the CTX treatment group (2.5 ± 1.6) (Fig 4B). The lung weight was much lower in *P*. *mirabilis* treatment group (0.12 ± 0.02 g) than in the control group (0.25 ± 0.10 g, p < 0.05) (Fig 4C). Again, there was no statistically significant difference between the *P*. *mirabilis* treatment group and the CTX treatment group (0.12 ± 0.02 g) (Fig 4C). The results were further confirmed by examining sections under the microscope (Fig 4D).

### *P*. *mirabilis* treatment influences the immune system *in vivo*

**Fig 5:**
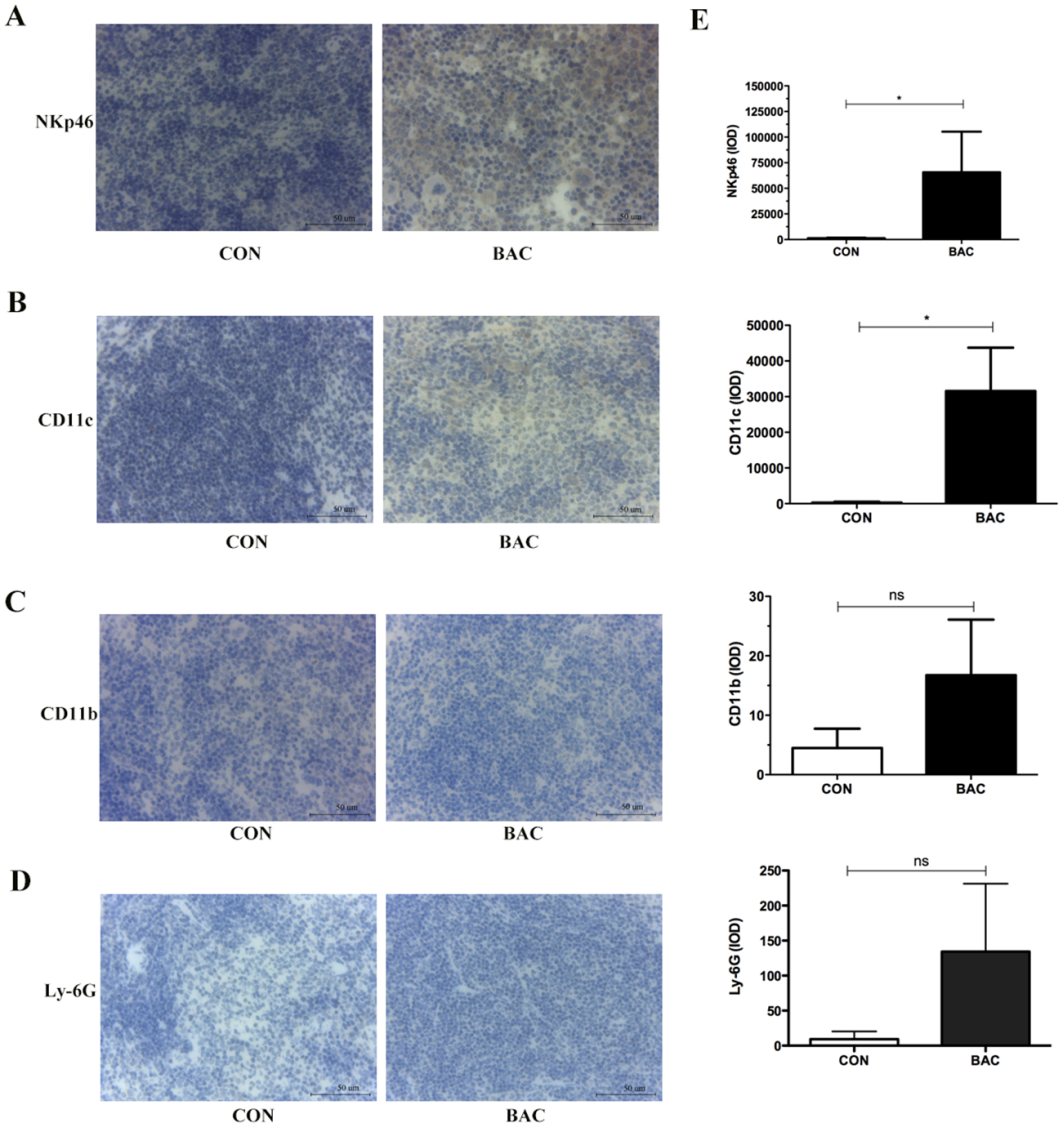
*P*. *mirabilis* treatment regulated the immune system *in vivo*. Results of IHC indicated that the expression of NKp46 (A) and CD11c (B) in spleen sections was significantly increased after 24 hours bacteria treatment (p < 0.05). There were no differences of the expression of CD11b (C) and Ly-6G (D) between the control groups and the groups after 24 hours bacteria administration. (E) Quantitative analysis of immunohistochemistry staining for the expression of NKp46, CD11c, CD11b, and Ly-6G in the mice spleen tissues, respectively.

To figure out the effect of the immune system on bacteria treatment, the spleen sections were stained with NKp46 (a marker for Natural killer cells, NK cells), CD11c (a marker for dendritic cells, DCs), CD11b (a maker for macrophage/monocyte), and Ly-6G (also known as Gr-1, a marker for granulocyte) antibodies. The results showed that the expression of NKp46 and CD11c was significantly different between the control group and the group of 24 hours after bacteria treatment (Fig 5). However, measurements of serum cytokines showed that 22 cytokines (S1 Table) did not change significantly, only the serum concentration of granulocyte colony-stimulating factor (G-CSF) was a light higher 24 hours following treatment in the bacterial therapy group compared with those in the PBS control group.

### *P*. *mirabilis* treatment reduces indicators of hypoxia in the tumor microenvironment

**Fig 6:**
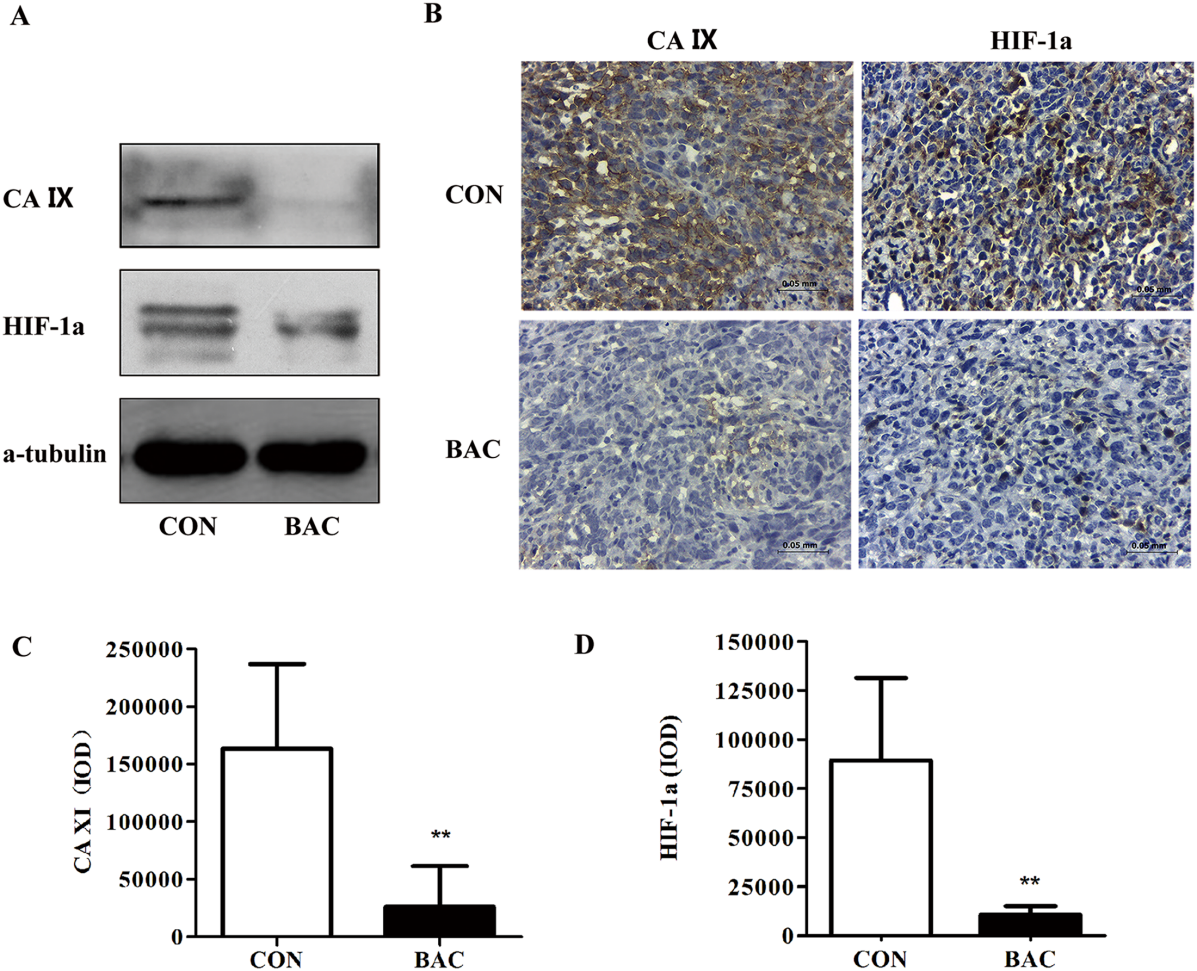
*P*. *mirabilis* treatment alters markers of hypoxia in the tumor microenvironment. (A) Western blotting demonstrated reduced expressions of CA IX and HIF-1a in the bacteria treatment group after 21 days of treatment; (B) The result was confirmed by immunohistochemistry for CA IX and HIF-1a in tumor sections of treated and control mice. CA IX positivity was localized in the cytoplasm and HIF-1a in the nucleus. (C and D) Quantitative analysis of immunohistochemistry staining indicated that CA IX and HIF-1a expression were lower in the bacteria treatment group than those in the control group (p < 0.05).

To further study the mechanism by which *P*. *mirabilis* suppressed cancer growth and lung metastasis, western blotting and IHC analysis were conducted. The result of western blotting demonstrated that the expressions of CA IX and HIF-1a were lower in mice in the *P*. *mirabilis* treatment group than those in the PBS control group (Fig 6A); this was confirmed quantitatively by IHC (Fig 6B–6D).

## Discussion

In the present study, we evaluated the suppressive effect of *P*. *mirabilis* on breast cancer growth and metastasis *in vitro* and *in vivo*. Results suggested *P*. *mirabilis* Hauser could adhere cells and inhibit cell growth in a non-specific way *in vitro*, preferentially accumulate in tumor tissues (Fig 1), and could markedly suppress both tumor growth and pulmonary metastasis in a mouse breast cancer model.

*P*. *mirabilis* is a facultative anaerobic bacteria which is not limited by the availiablity of O_2_ and have a great number of alternative pathways and enzymes which are regulated and expressed under the appropriate conditions [19, 20]. Therefore, *P*. *mirabilis* can grow both in the anaerobic and aerobic conditions. High densities of bacteria were observed in tumor tissues 24 hours after injection treatment and persisted for several days (Fig 2A). This specific accumulation of *P*. *mirabilis* in a tumor can be utilized to deliver genes, prodrugs, cytotoxic proteins and immunomodulatory proteins to tumors, as has been demonstrated with *S*. *typhimurium* [21], *E*. *coli* [22], and *Clostridium* [23]. Jeong et al [24] have developed an *S*. *typhimurium* strain, which specificity delivers the mitochondrial targeting domain of Noxa (MTD), as a potential therapeutic cargo protein, and the antitumor effect is considerably greater than that without delivering MTD. *P*. *mirabilis* may have the potential to become a similarly effective vector to specifically deliver therapeutic transgenes to tumors.

*P*. *mirabilis* mainly causes urinary tract infections (UTI) in patients, which triggers between 1%-10% of all UTI [25], however, only a few percent of cases with uncomplicated cystitis or acute pyelonephritis are infected by this bacteria [26]. In our study, the kidneys of mice treated with *P*. *mirabilis* had no apparent histological difference versus those in the PBS control group, and there were very few bacteria in mice kidney tissues at 72 hours after injection (Fig 2A). Moreover, the *P*. *mirabilis* strain utilized here is sensitive to many antibiotics, including fluoroquinolones, nitrofurantoin, fosfomycin, imipenem or amikacin, making treatment of any potential adverse effects straightforward [27]. During the 21-day treatment, the body weight of treated mice initially decreased; however, it recovered at the end of 21 days treatment (Fig 3). By doing the blood biochemical examination, results showed AST and ALT were elevated at 72 hours after bacteria injection, but there were no differences between the control group and the group after 21 days treatment; other liver indicators were no change among all groups (Table 1). Those indicate bacteria can cause liver transient damage during bacteria therapy, however, this damage will be recovered later. Since injection of 4T1 cells into mouse fat pad in spontaneous metastasis model has long been well known to cause leukemoid reaction with profound granulocytosis resulting in increasingly apparent liver injury [28], it would be very difficult to evaluate whether liver is further damaged upon bacteria injection, arguing that the 4T1 spontaneous cancer metastasis assay is an appropriate model. However, one study indicates that there is significantly increased AST serum concentrations and liver necrosis/damage in the 4T1-bearing mice after postsublethal endotoxin challenge compared with nontumor-bearing animals [29], which means 4T1-bearing mice may be a better model for assessing the liver function during the bacteria treatment. Moreover, there are also a lot of literatures that utilize this model to assess the liver injury during the treatment, including the bacteria therapy [29–33]. Therefore, 4T1 spontaneous cancer metastasis assay can be an appropriate model to evaluate the liver injury during the bacteria injection. One of the reasons that we did not observe how normal mice reacted after intravenously received *P*. *mirabilis* is to reduce the number of mice used as much as possible because of animal welfare. Another reason is the *Proteus* strains show little toxic to mice according to the previous studies [16, 34]. The above results suggest that *P*. *mirabilis* may be less virulent than other bacteria such as *S*. *typhimurium*, which can only be safely administered to animals when it is attenuated [35]. However, attenuated *P*. *mirabilis* strains may be more preferable for clinical use, therefore additional studies should evaluate attenuated *P*. *mirabilis* strains which retain tumor targeting capacity but may be associated with even further reduced toxicity.

After *P*. *mirabilis* administration, large areas of tumor cell death and infiltrated inflammatory cells were observed compared with the PBS control group (Fig 2); thus, *P*. *mirabilis* triggers cell death and anticancer effect presumably by recruiting inflammatory cells. The expressions of Ly-6G (Gr-1) and CD11b are significantly increased in the tumor, blood, lung, and liver of 4T1-bearing mice [36–41]. However, those results are analyzed at different days after 4T1 cells transplantation. Our IHC results showed Ly-6G and CD11b were expressed in the lung at 24 hours after bacteria injection (i.e. 5 days after the 4T1 cells transplantation), which was similar with the previous results [37], but there were no different between the control and bacteria treatment groups (the data didn’t show). The increasing expressions of those markers in the tumor are usually detected at 10 days or more than 10 days after tumor transplantation by using IHC or flow cytometry [37, 40]. In our study, to observe the immune reaction to the bacteria treatment in the mouse model, the experiment was carried on at 5 days after the 4T1 cells transplantation. For the spleen, the results show the expressions of Ly-6G and CD11b are at lower levels in the 4T1-bearing mice by flow cytometry [36]. Therefore, those may be the reasons why those proteins were at a really low level expression on the mice spleen and tumor by using IHC in our study. Our results further demonstrated the expression of NKp46 and CD11c was significantly increased after 24 hours bacteria treatment in the mice spleen sections by IHC (Fig 5). Natural killer (NK) cells are a cytotoxic lymphocyte of the innate immune system. NKp46 is an Ig-like superfamily cell surface receptor participated in mouse NK cell activation [42]. Dendritic cells (DCs) are classically defined as the sentinel of the immune system. DCs may possess cytotoxic abilities [43, 44]. The NK-cell-DCs cross-talk was necessary to induce NK cells cytotoxic activity and result in innate anti-tumor immune responses in *vivo* [45–49].

Cancer metastasis is life-threatening and is always resistant to chemotherapy and radiation. So far, it is reported that two strains bacteria, Escherichia coli K-12 [50] and a Modified Salmonella typhimurium [51], can target metastasis of large primary tumors, however, it is difficult for most bacteria to target small non-necrotic and non-hypoxia metastases [52]. In our study, we found that *P*. *mirabilis* exerted a suppressive effect on tumor metastasis (Fig 4), which responsible for the majority of breast cancer-related deaths. In previous studies, the express of certain cytokines increases following bacteria treatment, which contributes to the prevention of tumor growth and metastasis, such as TGF-a, IL-6, and IL-12 [53, 54]. However, in our study measurements of serum cytokines showed 22 cytokines did not significantly change. It has been reported that DCs can directly initiate NK cells activation without involving specific cytokine, resulting in innate anti-tumor immune response in the mouse model [45], which may be one of the reasons why there was no significant change in detecting 22 cytokines following bacteria treatment in our results.

One of the major causes that may play a critical role in the metastasis of cancer is hypoxia, which occurs in most solid human cancers [55, 56]. The intriguing evidence for hypoxia in tumor development and its therapeutic importance make hypoxia a high priority target for tumor therapy [57]. Previous studies have shown that over-expression of CA IX and HIF-1a, surrogates for hypoxia, correlates with a poor prognosis in invasive breast carcinomas [58], and may enhance metastatic properties of cancer cells by modulating tumor-associated cell migration and invasion [59–61]. Many studies also indicate that the expressions of HIF-1a and CA IX are independent of tumor size in the breast cancer [62–70]. Our results demonstrated that expression of CA IX and HIF-1a in tumor tissues of mice was reduced by bacteria treatment via IHC and western blotting analysis (Fig 5). Therefore, these results suggest *P*. *mirabilis* may alter the hypoxic tumor microenvironment and then inhibit the development of lung metastasis in mouse cancer models. These new insights will helpful for the understanding of the relationship between bacteria and tumor, and for improving the antitumor effect of bacteria treatment.

## Conclusions

These results suggest that *Proteus mirabilis* can suppress the growth of primary tumors and pulmonary metastasis in mouse breast cancer models, and alteration of the immune system and the hypoxic tumor microenvironment could play a role in these observations.

## Supporting information

S1 Text Adherence assay. (XLSX)

S2 Text Body distribution of bacteria in tumor bearing mice. (XLSX)

S3 Text Cytokine array analyzed by Image-pro-plus software. (XLSX)

S4 Text The weight and volume of mice tumor. (XLSX)

S5 Text The metastatic foci of mice lung. (XLSX)

S6 Text The weight of mice lung. (XLSX)

S7 Text The body weight of mice. (XLSX)

S8 Text The data of IHC images of the mice tumor sections analyzed by Image-pro-plus software. (XLSX)

S9 Text Western image. (DOCX)

S10 Text Growth inhibition of bacteria treatment. (XLSX)

S11 Text The data of IHC images of the mice spleen sections analyzed by Image-pro-plus software. (XLSX)

S12 Text Tumor volume and lung metastasis in mice with LPS treatment. (XLSX)

S1 Table 22 mouse cytokines detected by Mouse Cytokine Antibody Array C1. (DOCX)

S1 Fig The effect of LPS on tumor growth and spontaneous pulmonary metastases in mouse breast tumor model. (A) The LPS was extracted from *P*. *mirabilis* by using hot aqueous-phenol extraction methods. Line a was the ladder, and line b was LPS of *P*. *mirabilis*. (B) There was no surface ulceration observed on mice tumor on gross at 24 and 96 hours after LPS treatment. There were no significantly differences in tumor volume (C) and the formation of spontaneous lung metastatic foci (D) between the LPS treatment and the PBS control treatment following 21 days treatment.
(TIF)

S2 Fig IHC images for Ki-67 in mice tumor sections of the control group. (ZIP)

S3 Fig IHC images for Ki-67 in mice tumor sections of the bacteria group. (ZIP)

S4 Fig IHC images for CA IX in mice tumor sections of the control group. (ZIP)

S5 Fig IHC images for CA IX in mice tumor sections of the bacteria group. (ZIP)

S6 Fig IHC images for HIF-1a in mice tumor sections of the control group. (ZIP)

S7 Fig IHC images for HIF-1a in mice tumor sections of the bacteria group. (ZIP)

S8 Fig IHC images for CD11b in mice spleen sections 24 hours after bacteria treatment. (ZIP)

S9 Fig IHC images for CD11b in mice spleen sections of the control group. (ZIP)

S10 Fig IHC images for CD11c in mice spleen sections 24 hours after bacteria treatment. (ZIP)

S11 Fig IHC images for CD11c in mice spleen sections of the control group. (ZIP)

S12 Fig IHC images for Ly-6G in mice spleen sections 24 hours after bacteria treatment. (ZIP)

S13 Fig IHC images for Ly-6G in mice spleen sections of the control group. (ZIP)

S14 Fig IHC images for NKp46 in mice spleen sections 24 hours after bacteria treatment. (ZIP)

S15 Fig IHC images for NKp46 in mice spleen sections of the control group. (ZIP)
