# Supplementary figures and images for: Proteus mirabilis inhibits cancer growth and pulmonary metastasis in a mouse breast cancer model (part 1 of 5)
Source: PLoS One. 2017 Dec 5;12(12):e0188960. doi: 10.1371/journal.pone.0188960 (PMC5716547; doi:10.1371/journal.pone.0188960)

HIF-1a

C 1 B 1 C 2 B 2 C 3 B 3


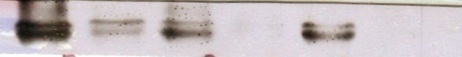


C 4 B 4 C 5 B 5 C 6 B 6


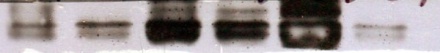


Ca IX

C 1 B 1 C 2 B 2 C 3 B 3


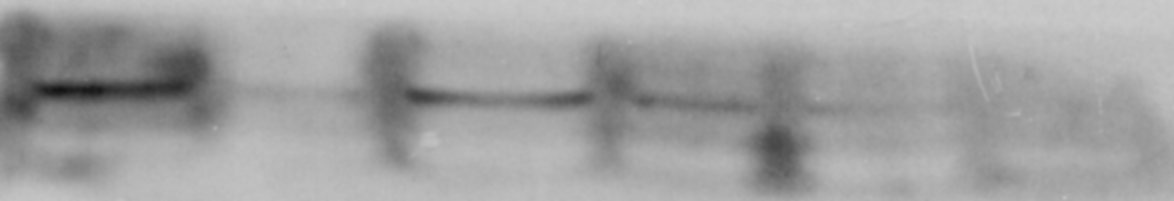


C 4 B 4 C 5 B 5 C 6 B 6


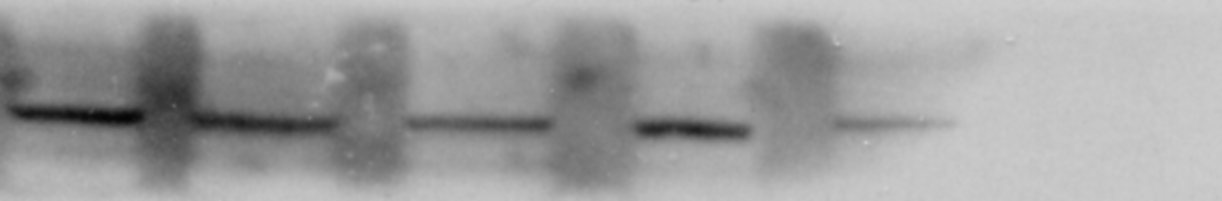

Supplement: S9 Text — (DOCX) [file pone.0188960.s009.docx]

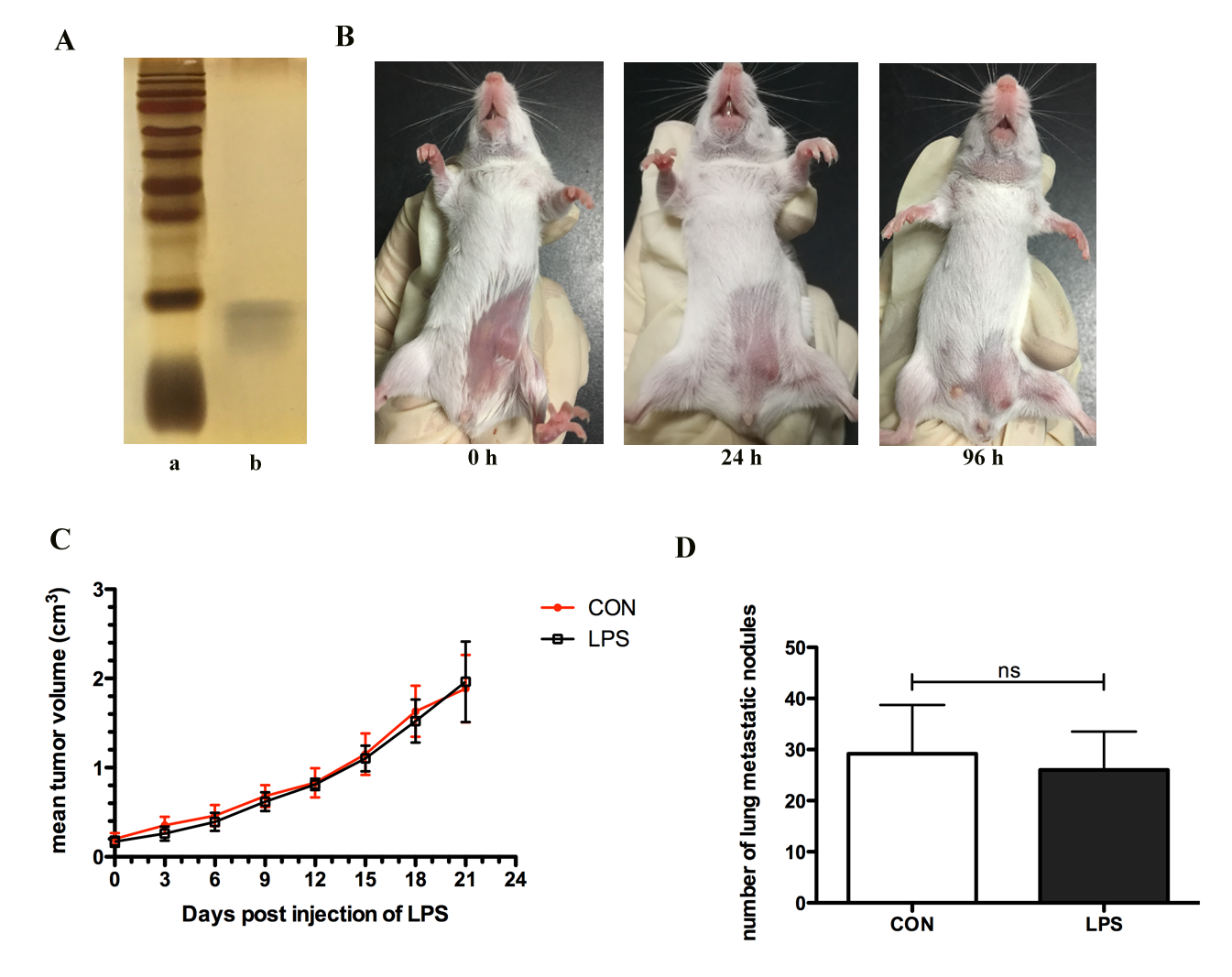

Supplement: S1 Fig — (A) The LPS was extracted from P. mirabilis by using hot aqueous-phenol extraction methods. Line a was the ladder, and line b was LPS of P. mirabilis. (B) There was no surface ulceration observed on mice tumor on gross at 24 and 96 hours after LPS treatment. There were no significantly differences in tumor volume (C) and the formation of spontaneous lung metastatic foci (D) between the LPS treatment and the PBS control treatment following 21 days treatment. (TIF) [file pone.0188960.s014.tif]

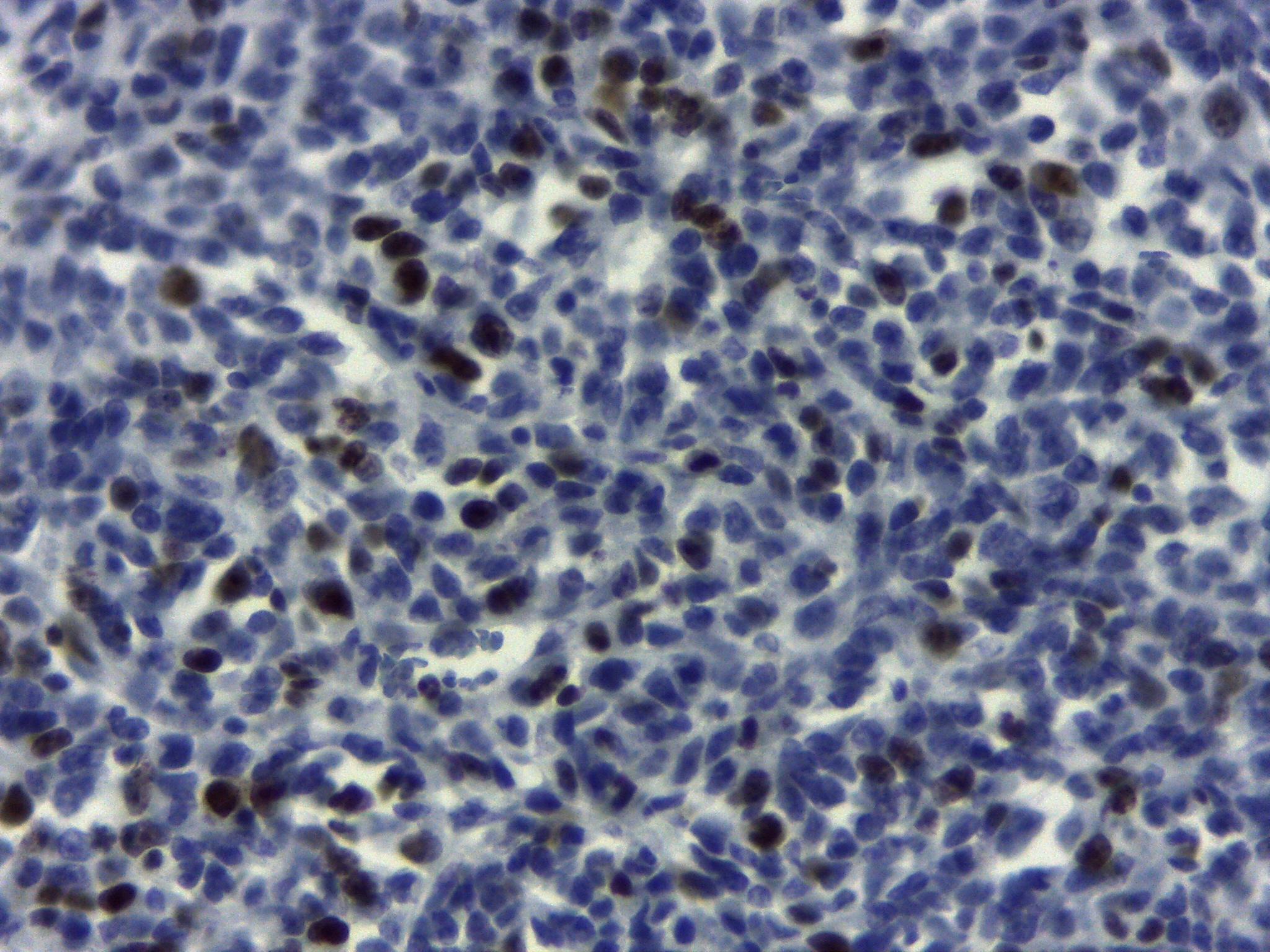

Supplement: S2 Fig — (ZIP) [file pone.0188960.s015.zip › Ki-67 IHC image con/Ki-67 con1-1.jpg]

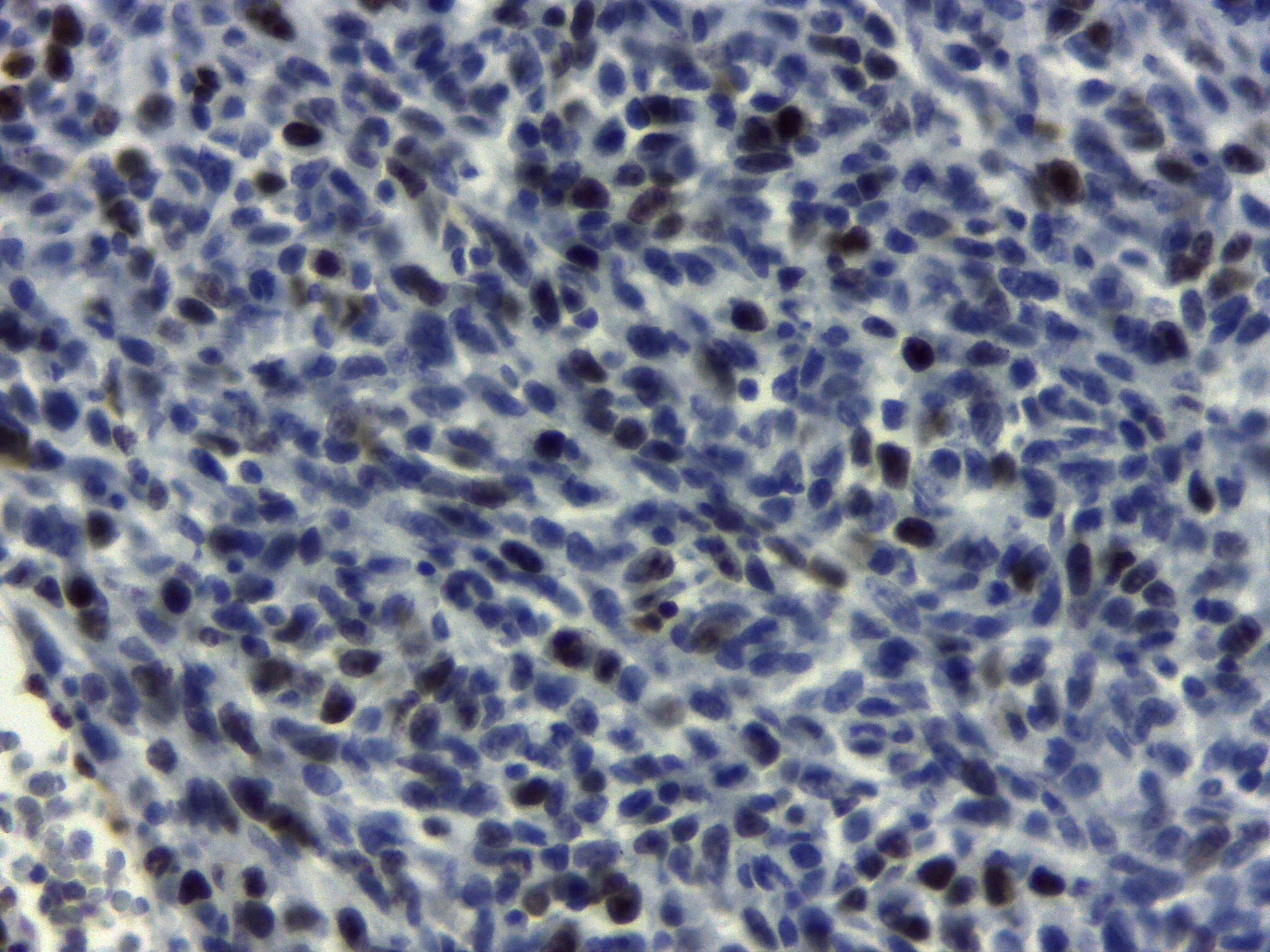

Supplement: S2 Fig — (ZIP) [file pone.0188960.s015.zip › Ki-67 IHC image con/Ki-67 con1-2.jpg]

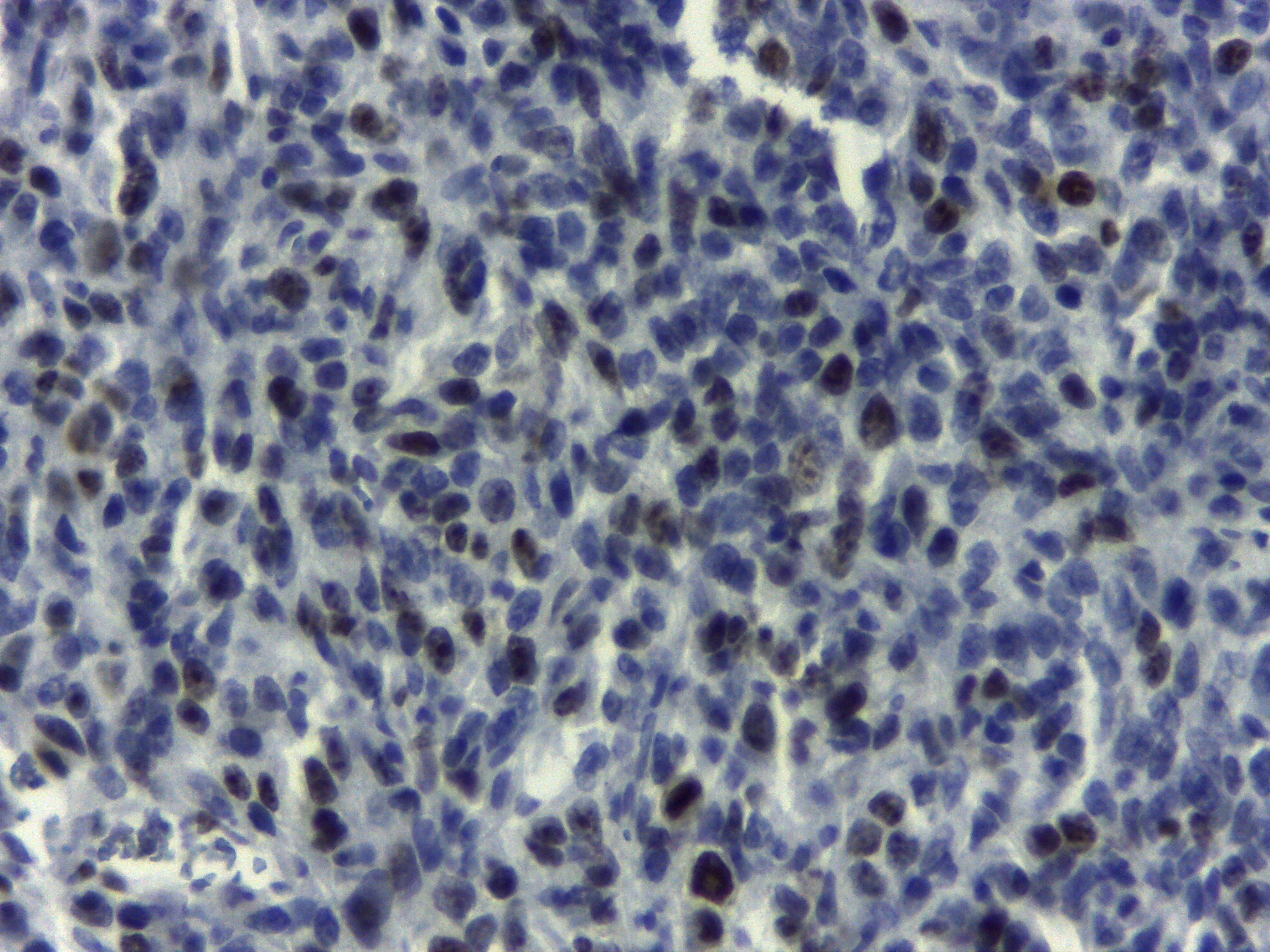

Supplement: S2 Fig — (ZIP) [file pone.0188960.s015.zip › Ki-67 IHC image con/Ki-67 con1-3.jpg]

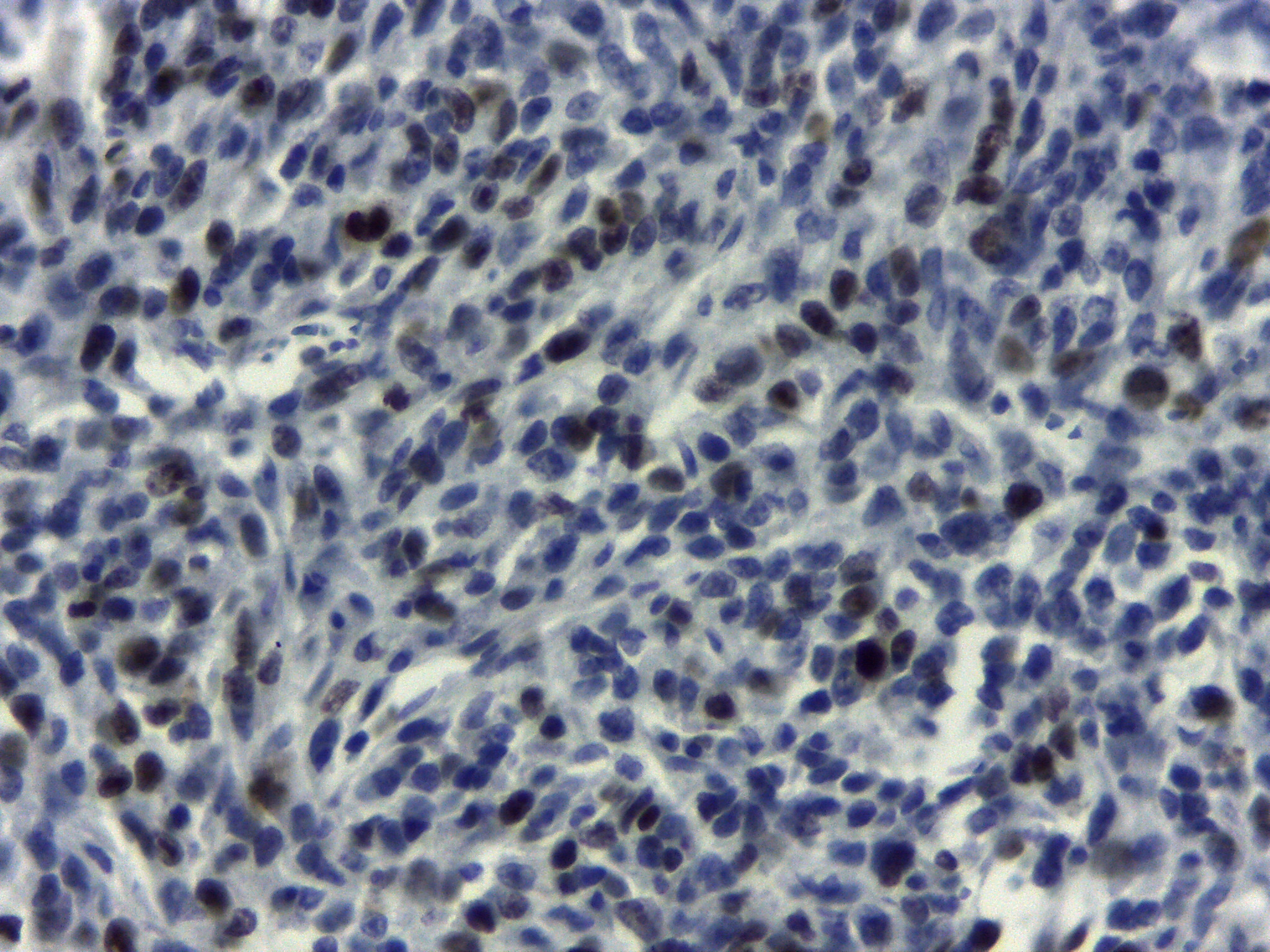

Supplement: S2 Fig — (ZIP) [file pone.0188960.s015.zip › Ki-67 IHC image con/Ki-67 con1-4.jpg]

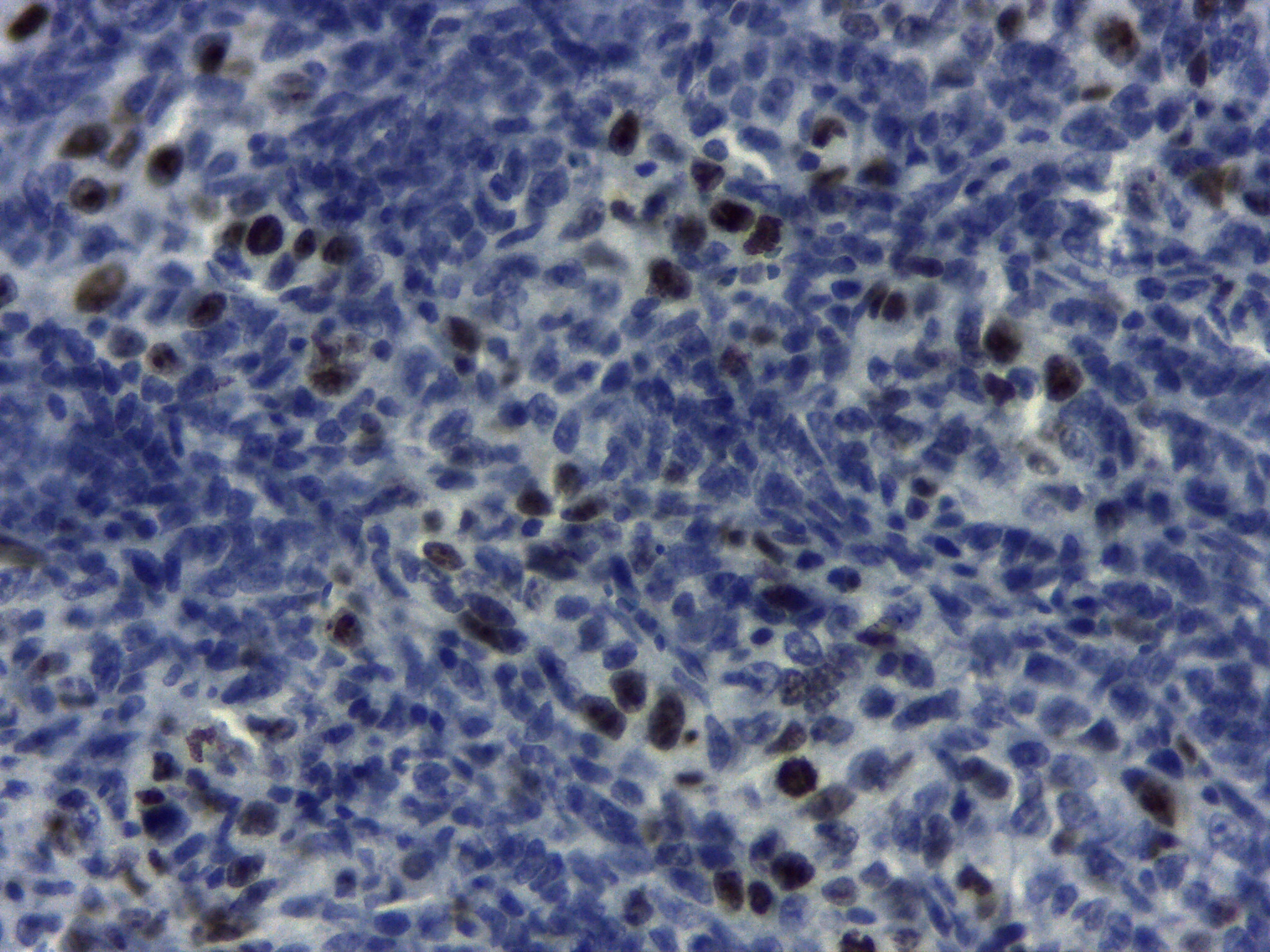

Supplement: S2 Fig — (ZIP) [file pone.0188960.s015.zip › Ki-67 IHC image con/Ki-67 con1-5.jpg]

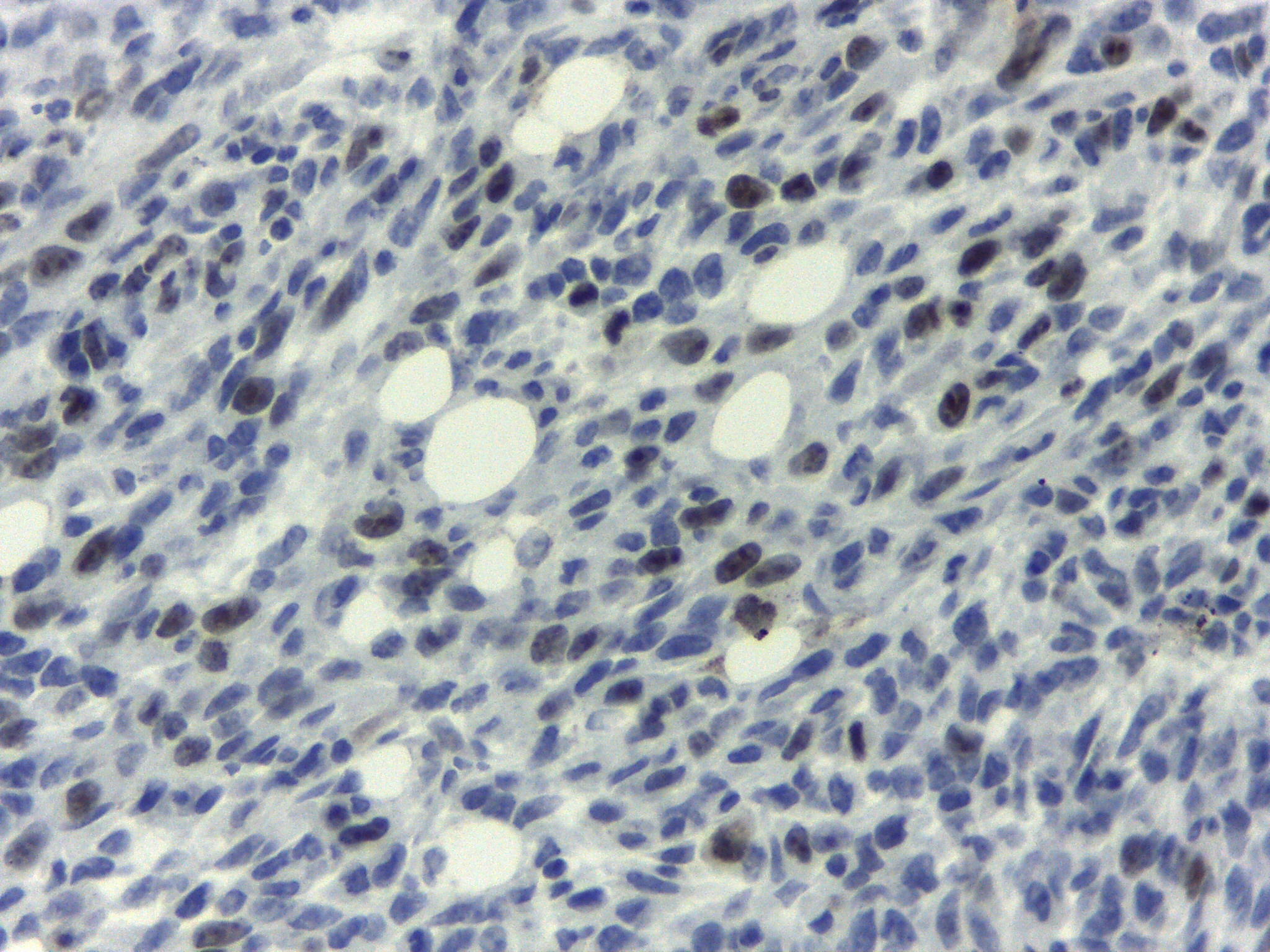

Supplement: S2 Fig — (ZIP) [file pone.0188960.s015.zip › Ki-67 IHC image con/Ki-67 con2-1.jpg]

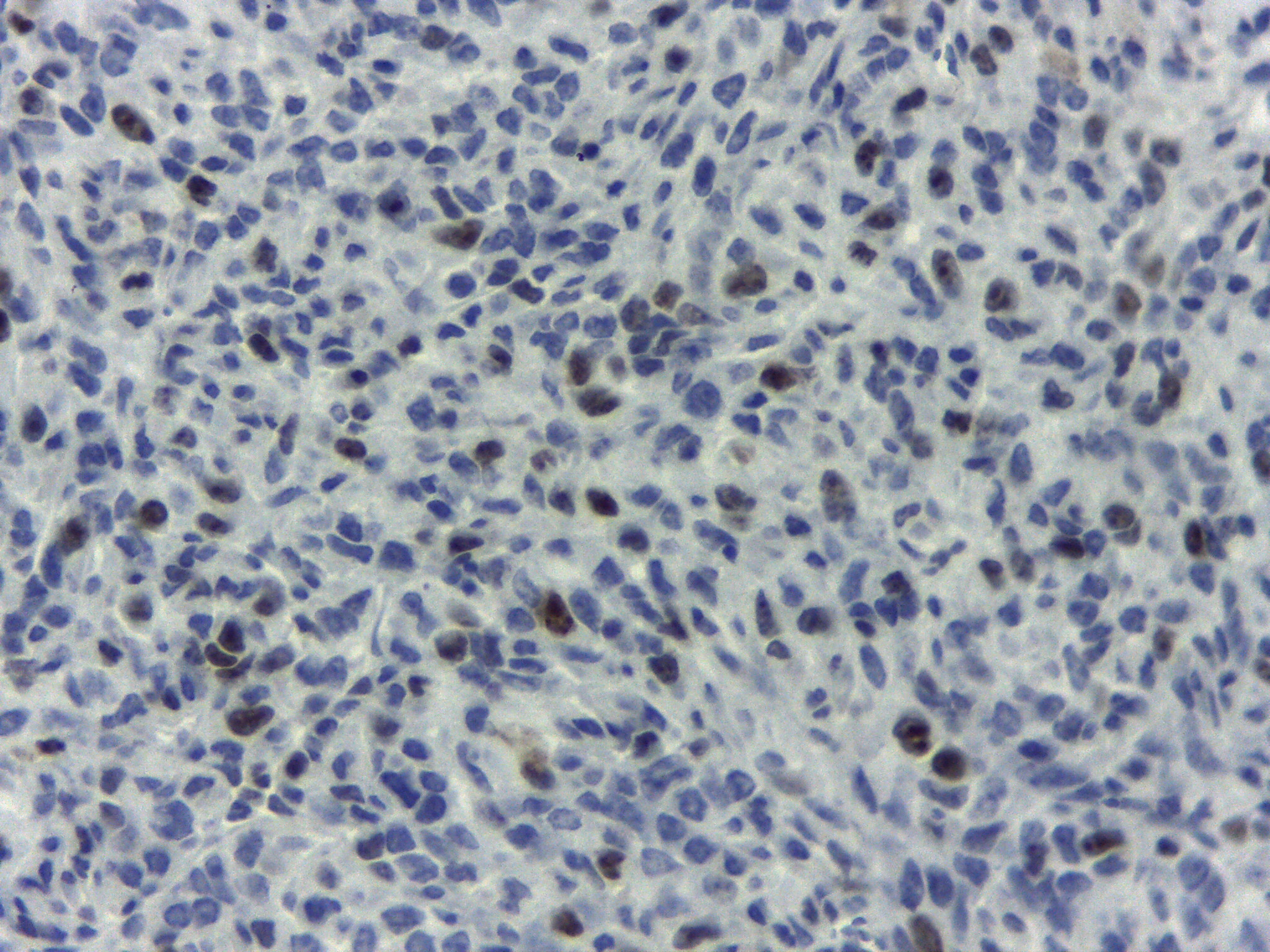

Supplement: S2 Fig — (ZIP) [file pone.0188960.s015.zip › Ki-67 IHC image con/Ki-67 con2-2.jpg]

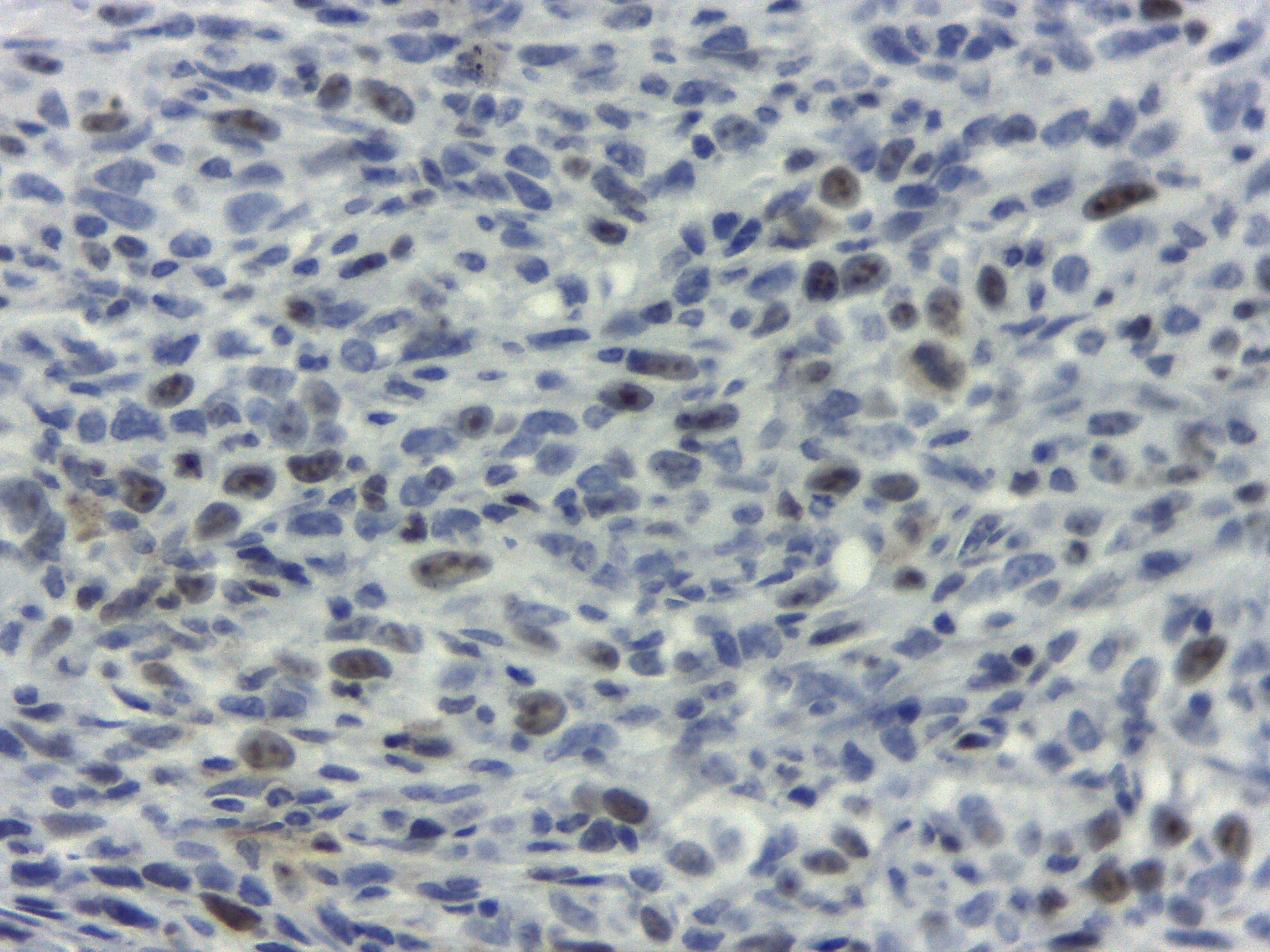

Supplement: S2 Fig — (ZIP) [file pone.0188960.s015.zip › Ki-67 IHC image con/Ki-67 con2-3.jpg]

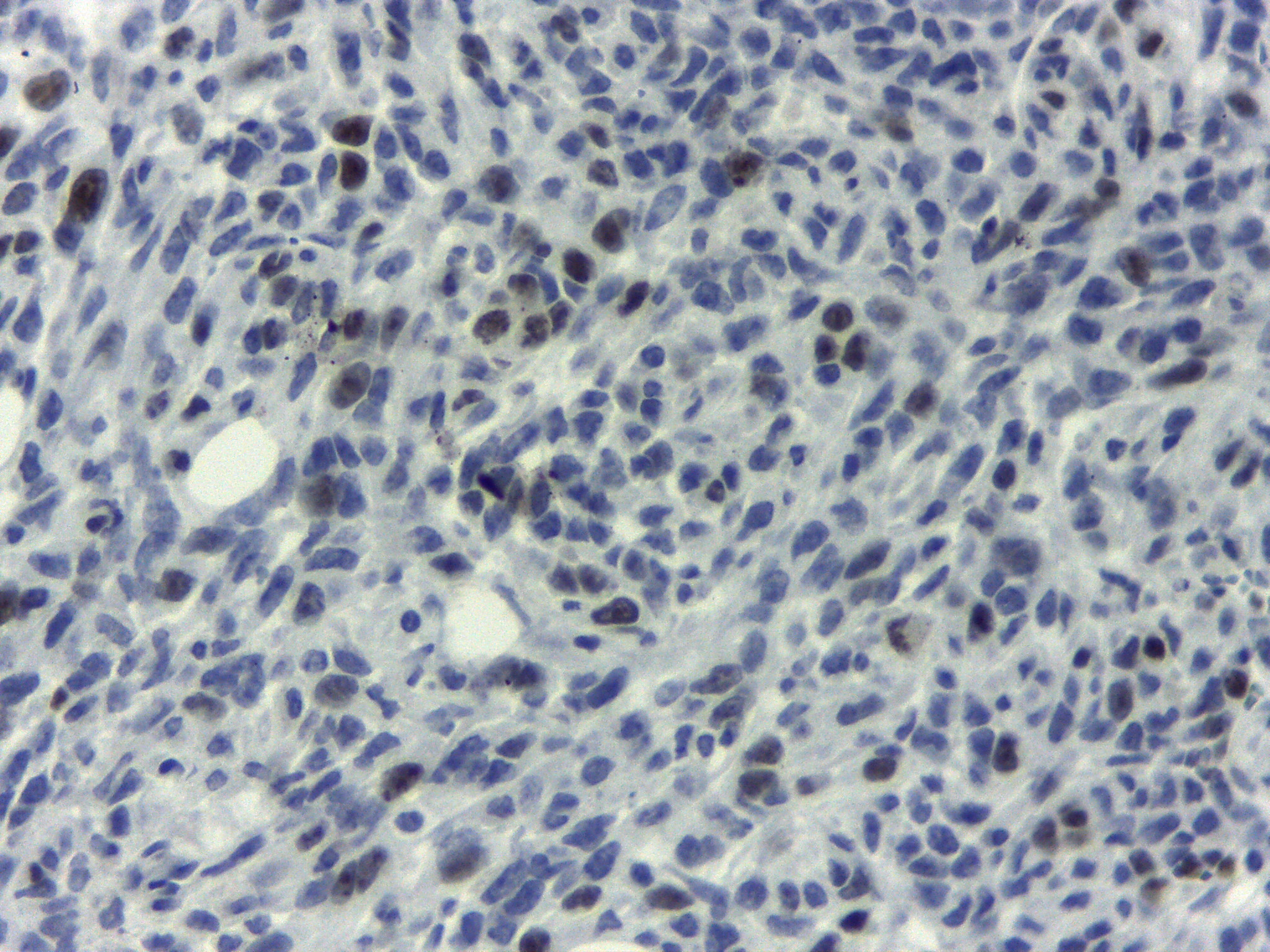

Supplement: S2 Fig — (ZIP) [file pone.0188960.s015.zip › Ki-67 IHC image con/Ki-67 con2-4.jpg]

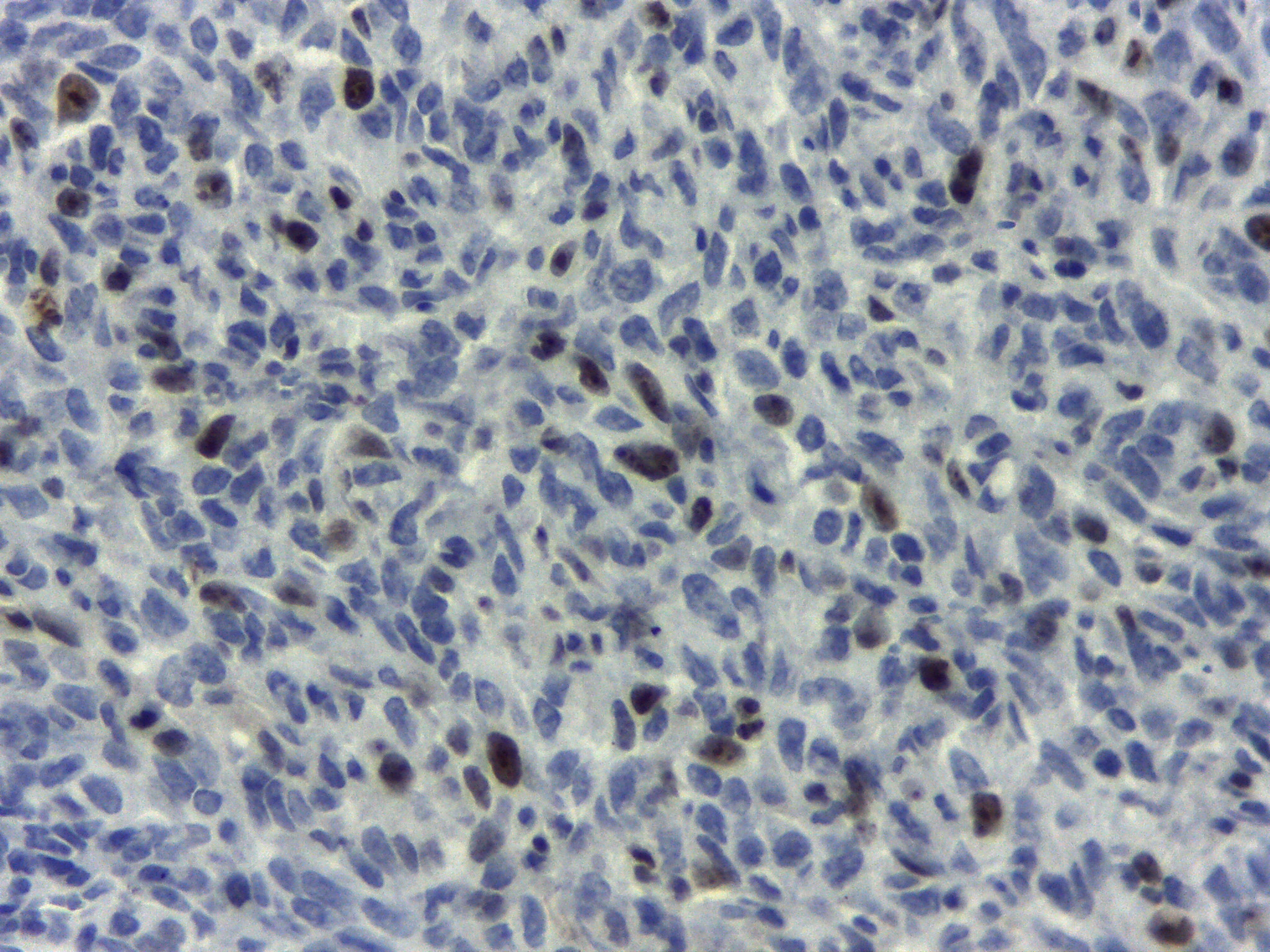

Supplement: S2 Fig — (ZIP) [file pone.0188960.s015.zip › Ki-67 IHC image con/Ki-67 con2-5.jpg]

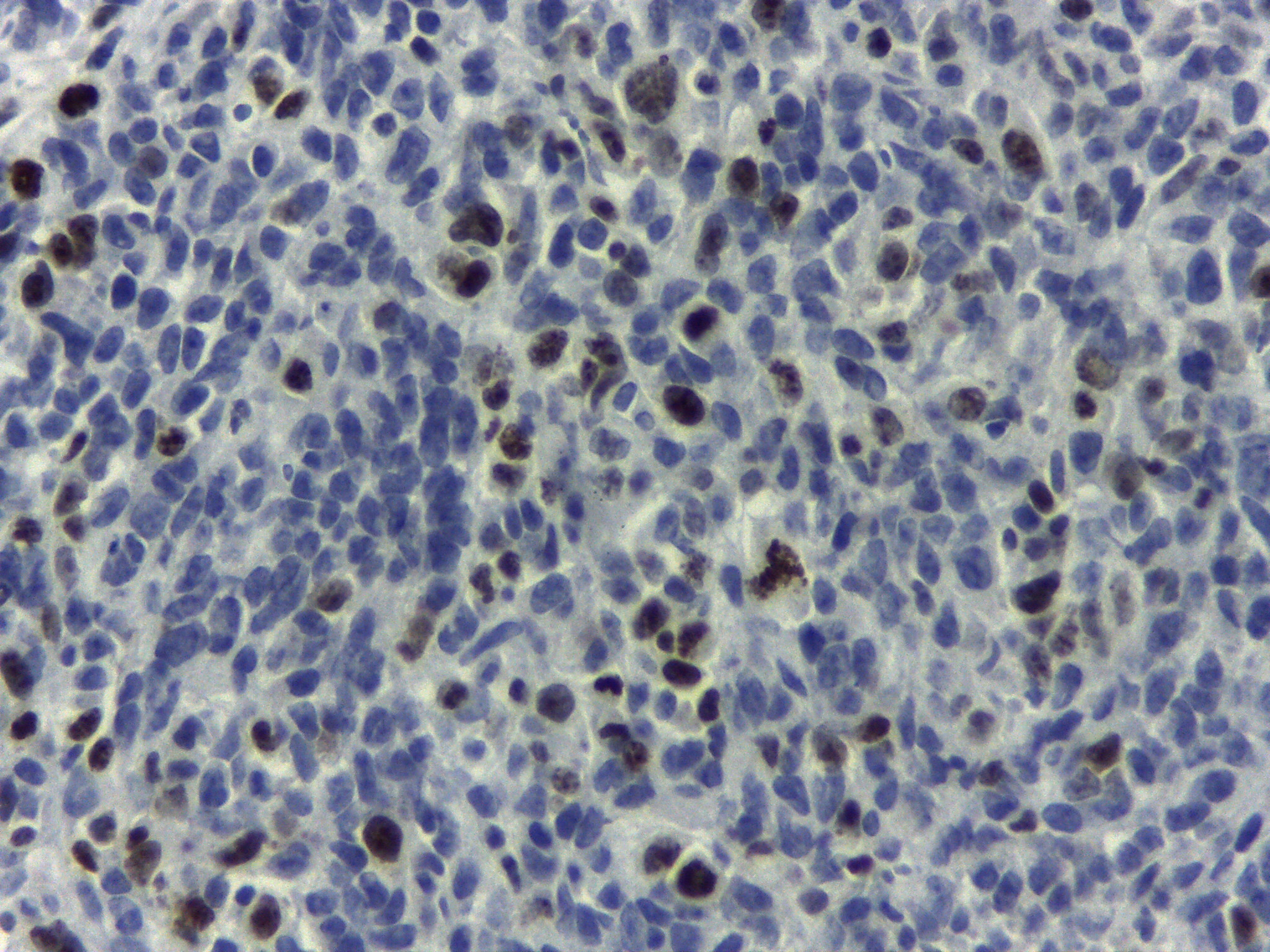

Supplement: S2 Fig — (ZIP) [file pone.0188960.s015.zip › Ki-67 IHC image con/Ki-67 con3-1.jpg]

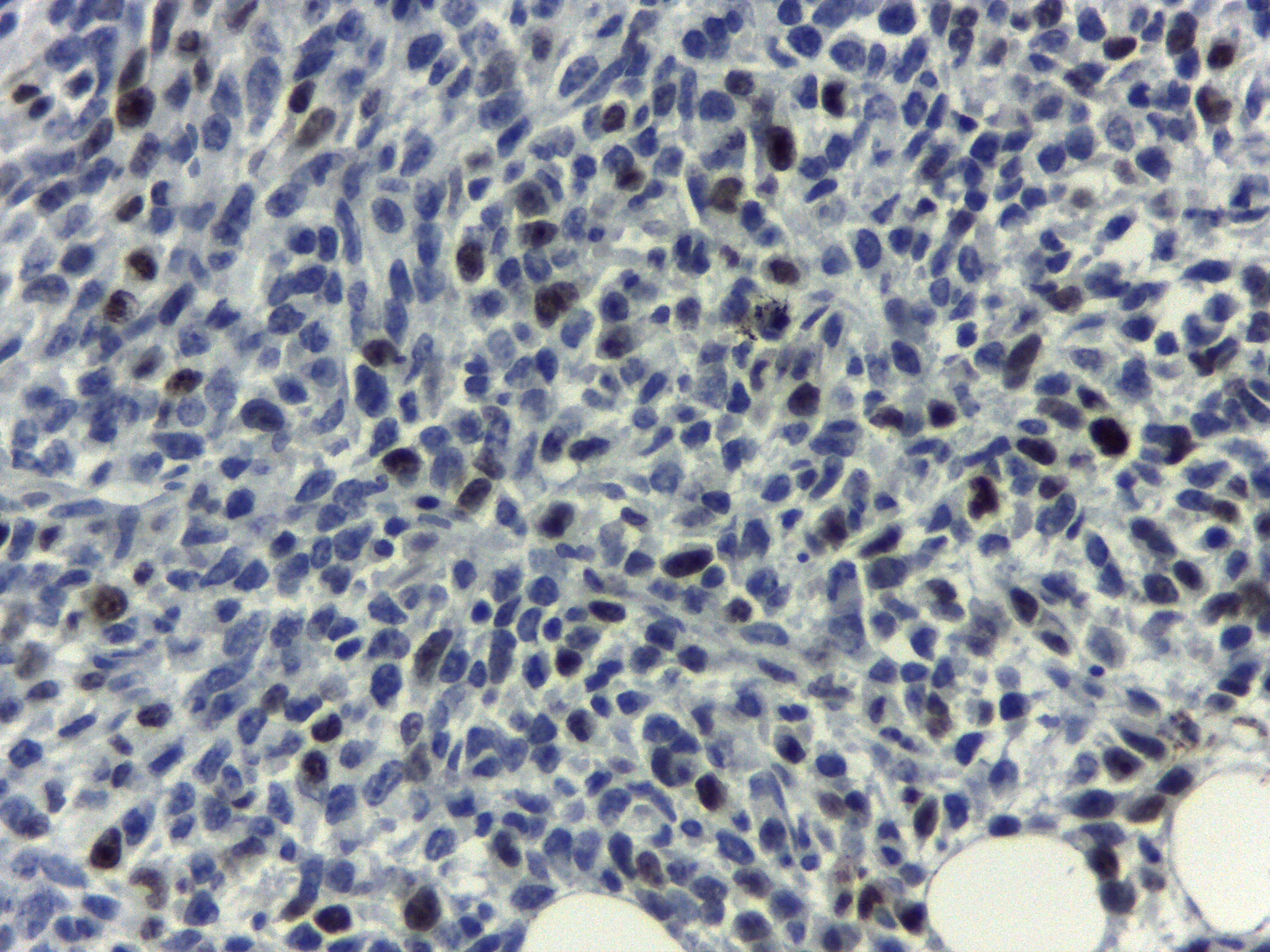

Supplement: S2 Fig — (ZIP) [file pone.0188960.s015.zip › Ki-67 IHC image con/Ki-67 con3-2.jpg]

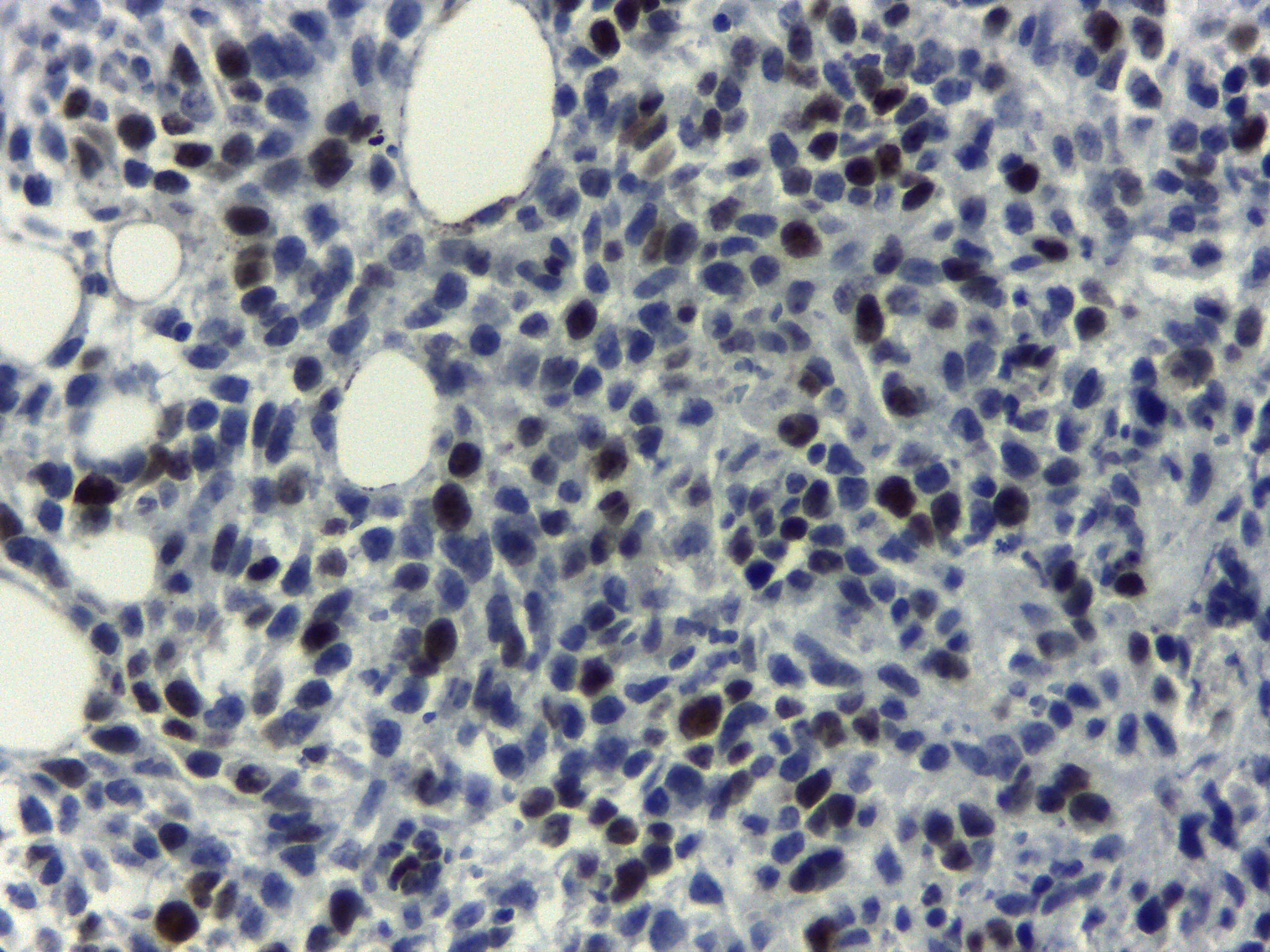

Supplement: S2 Fig — (ZIP) [file pone.0188960.s015.zip › Ki-67 IHC image con/Ki-67 con3-3.jpg]

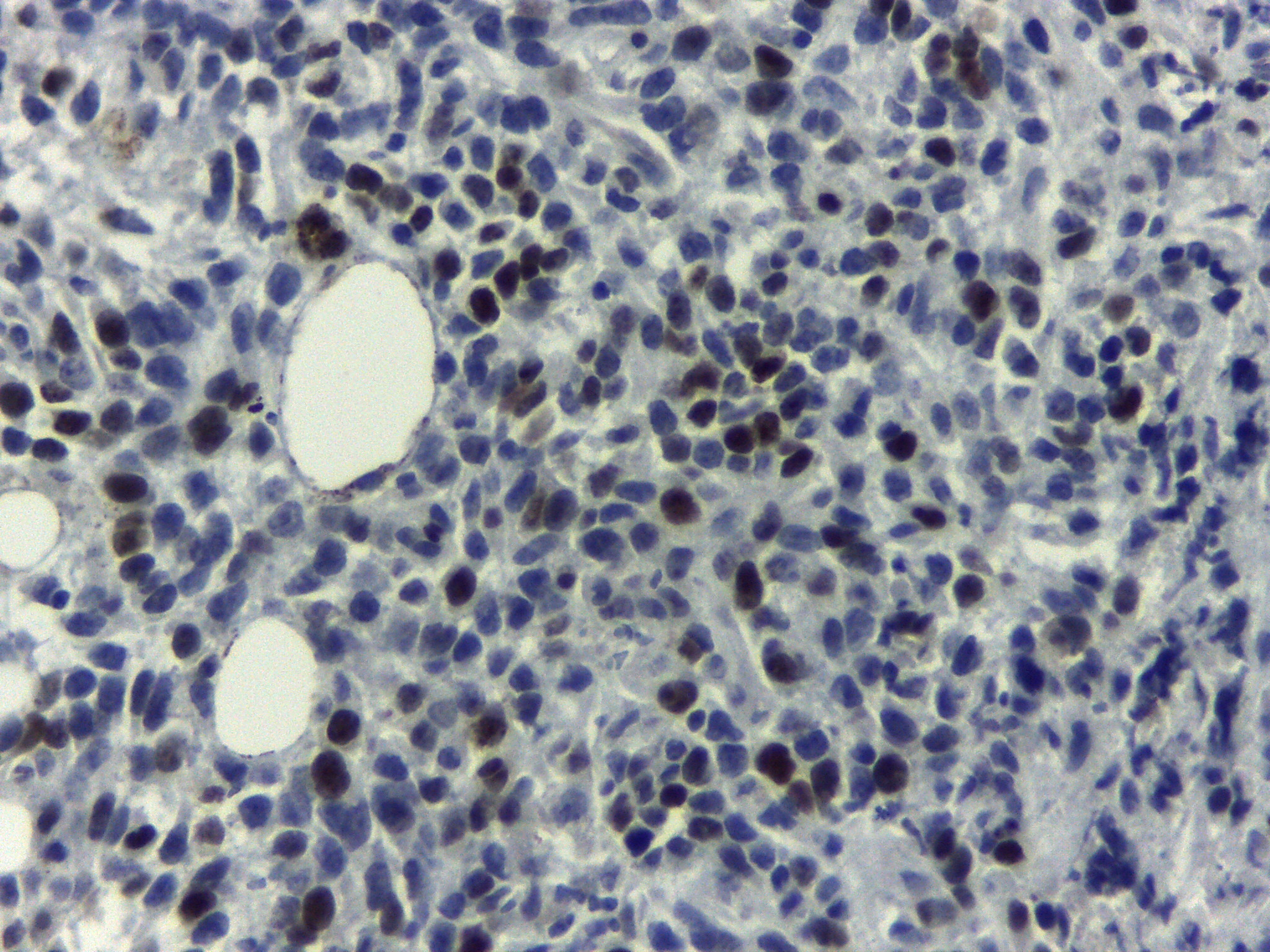

Supplement: S2 Fig — (ZIP) [file pone.0188960.s015.zip › Ki-67 IHC image con/Ki-67 con3-4.jpg]

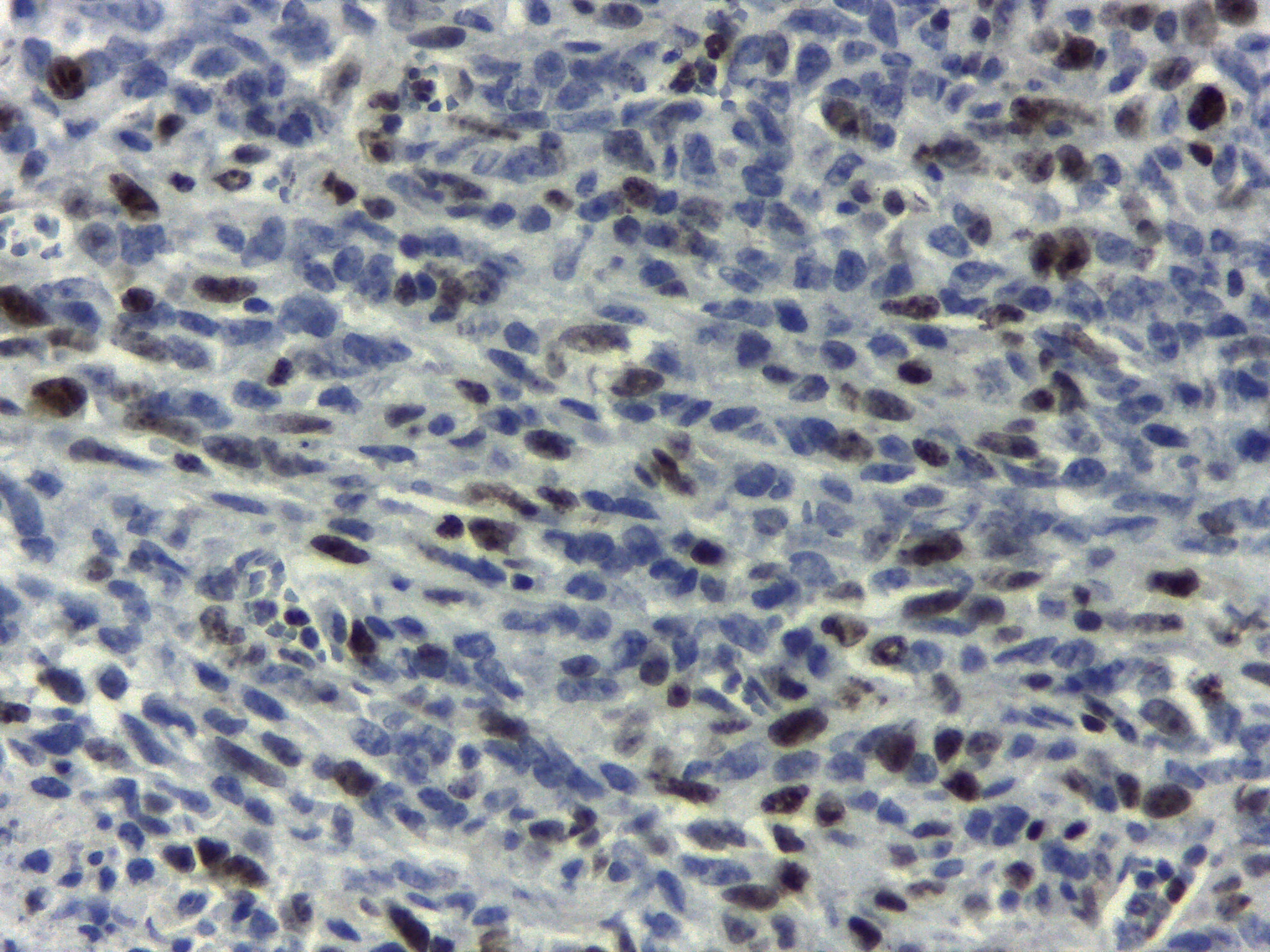

Supplement: S2 Fig — (ZIP) [file pone.0188960.s015.zip › Ki-67 IHC image con/Ki-67 con3-5.jpg]

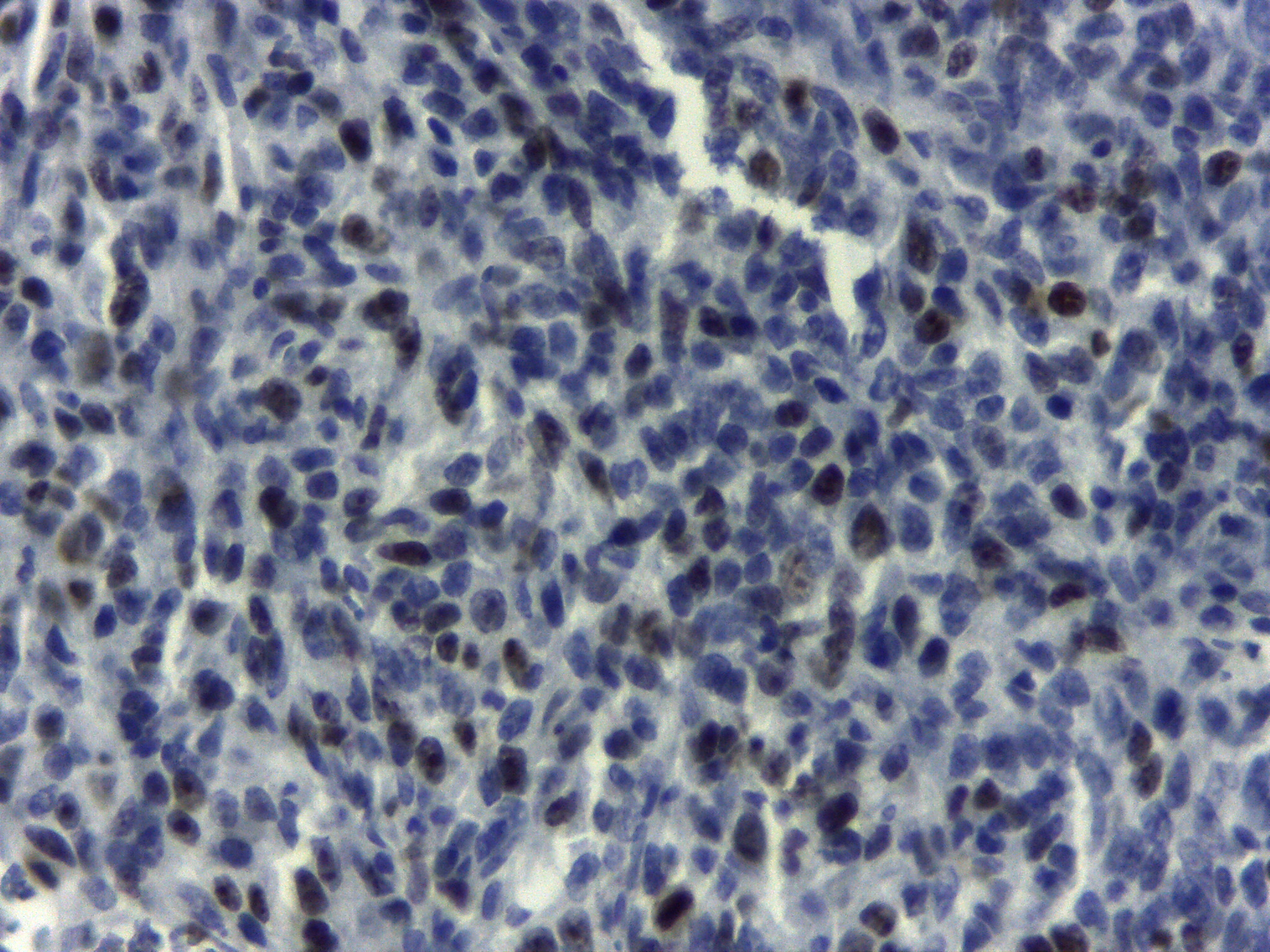

Supplement: S2 Fig — (ZIP) [file pone.0188960.s015.zip › Ki-67 IHC image con/Ki-67 con4-1.jpg]

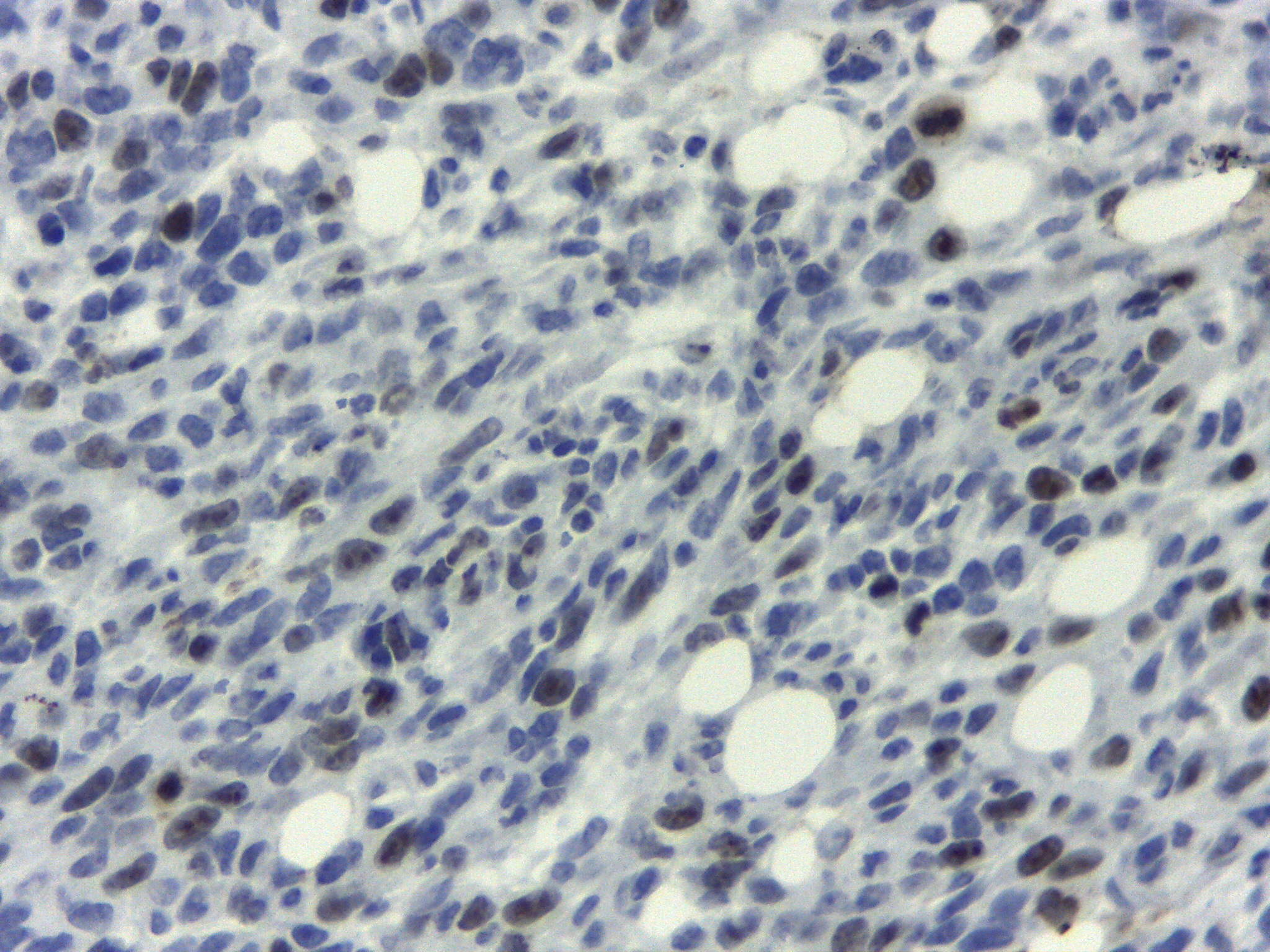

Supplement: S2 Fig — (ZIP) [file pone.0188960.s015.zip › Ki-67 IHC image con/Ki-67 con4-2.jpg]

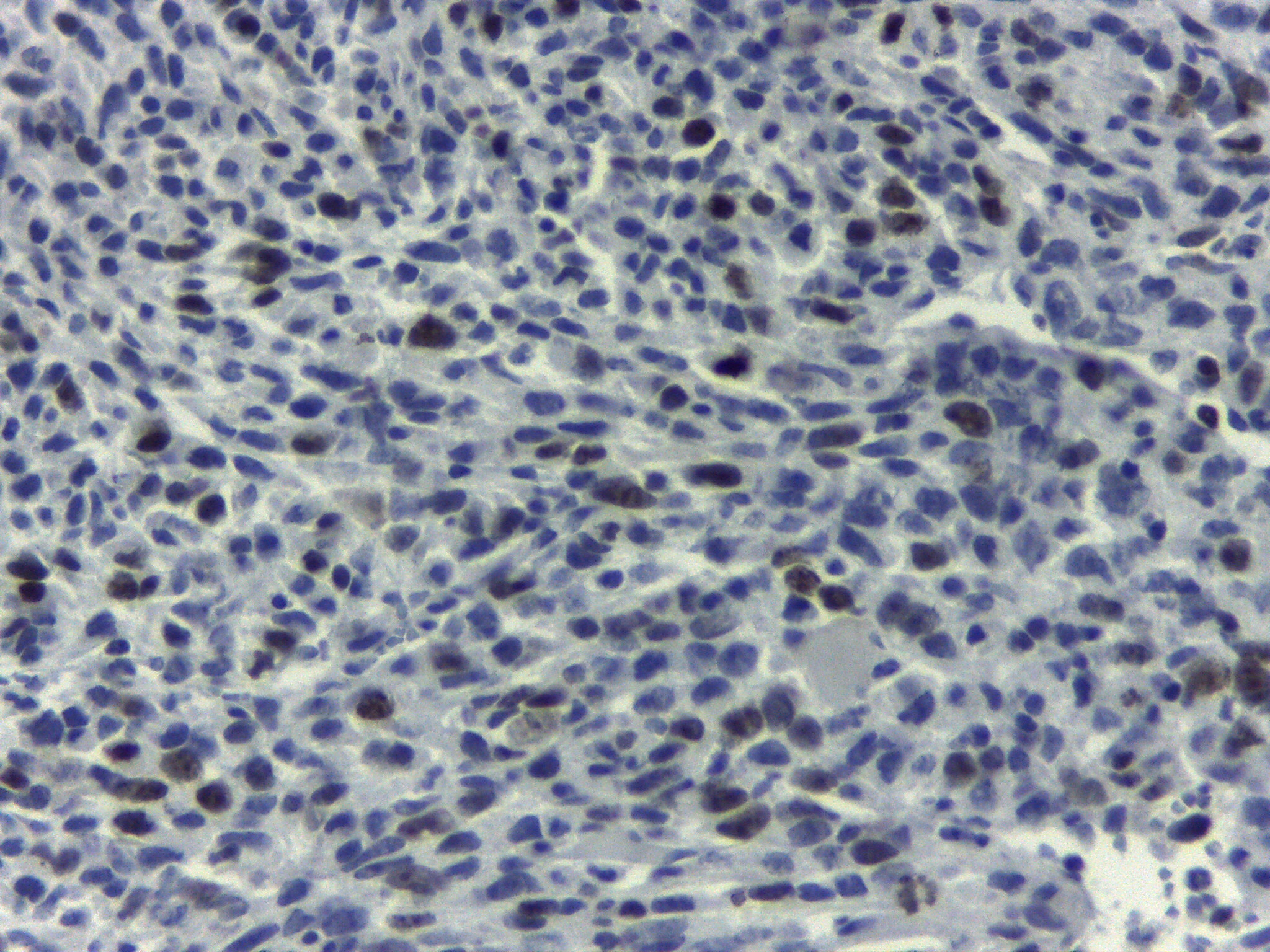

Supplement: S2 Fig — (ZIP) [file pone.0188960.s015.zip › Ki-67 IHC image con/Ki-67 con4-3.jpg]

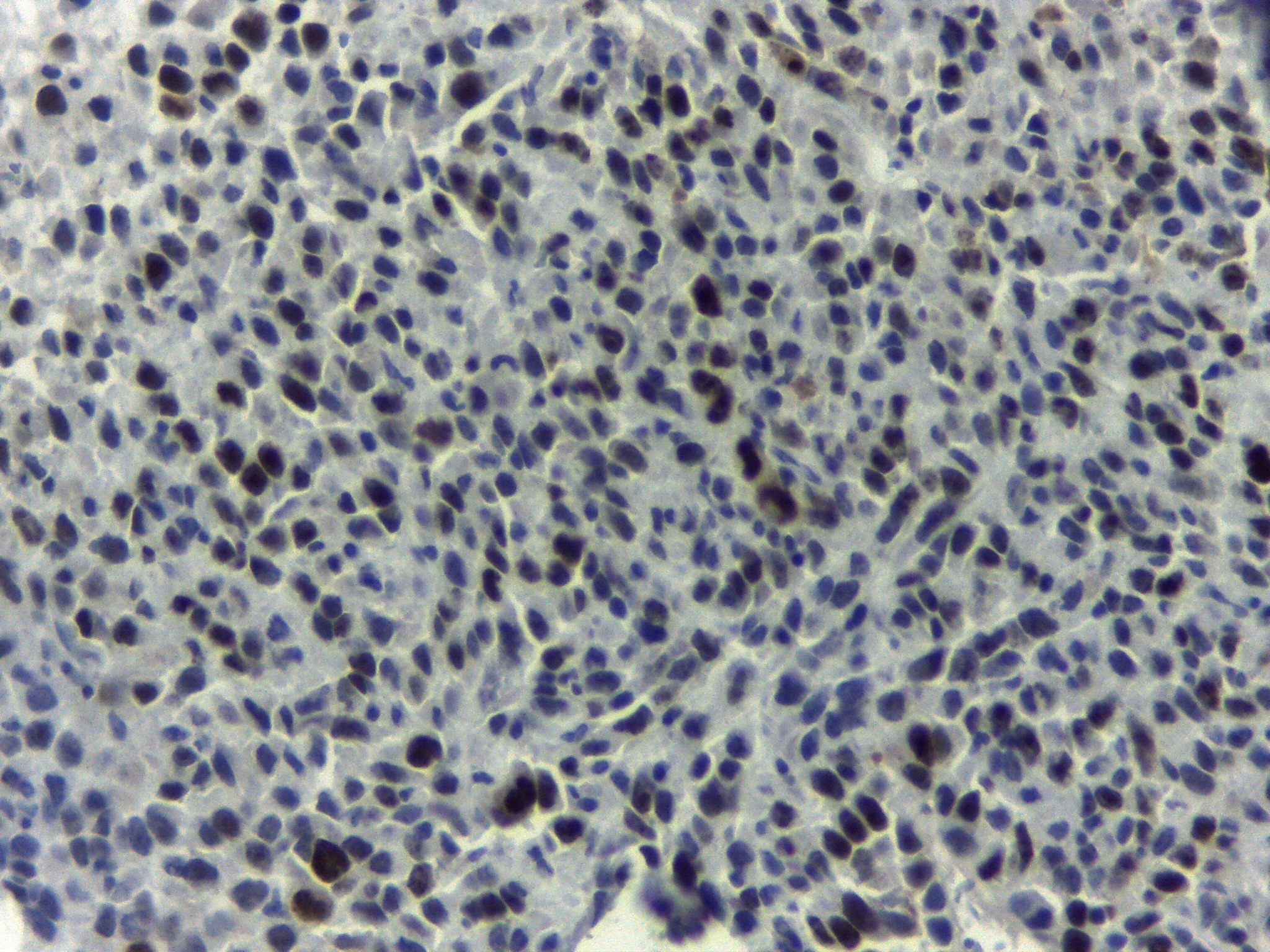

Supplement: S2 Fig — (ZIP) [file pone.0188960.s015.zip › Ki-67 IHC image con/Ki-67 con4-4.jpg]

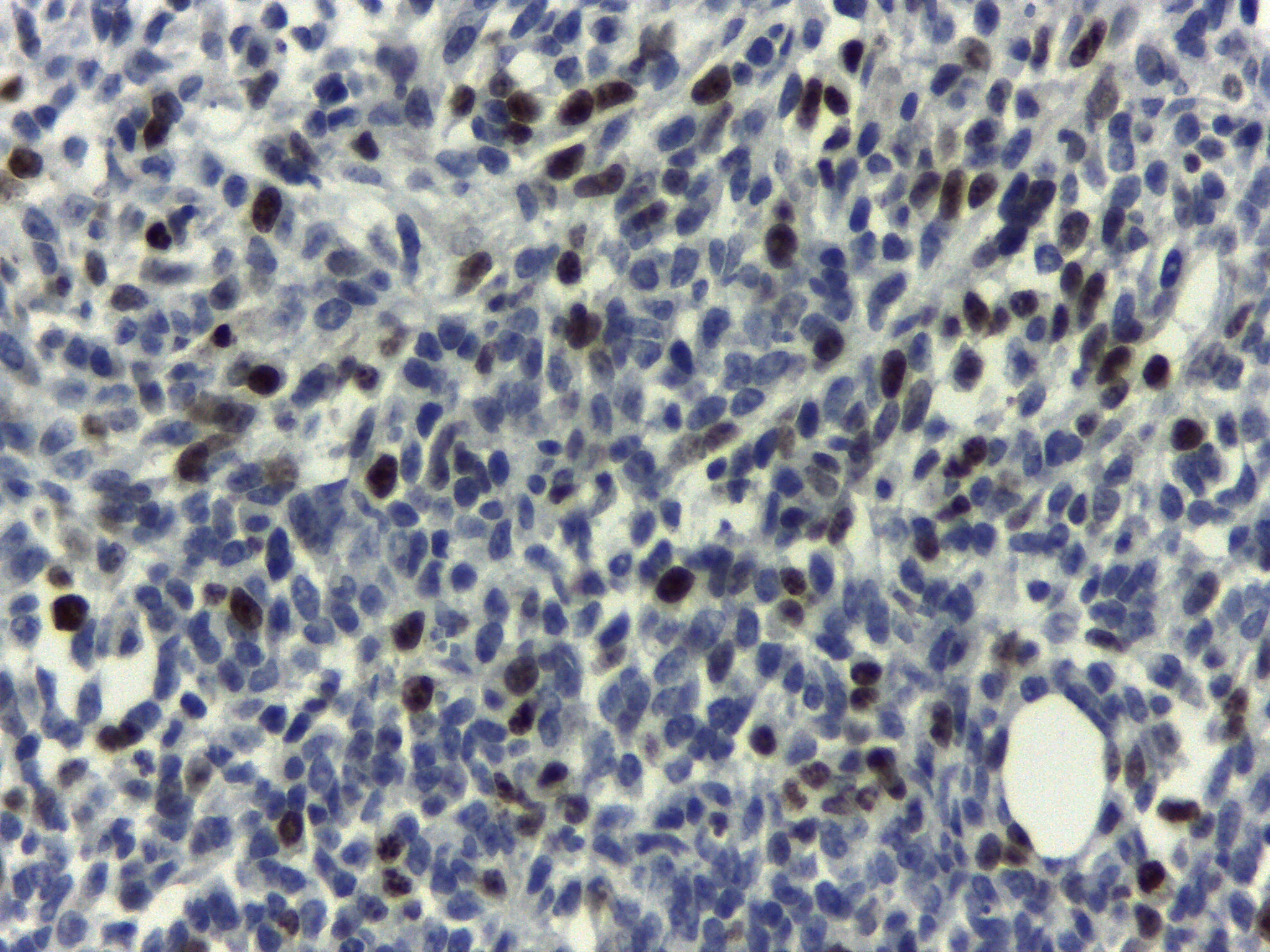

Supplement: S2 Fig — (ZIP) [file pone.0188960.s015.zip › Ki-67 IHC image con/Ki-67 con4-5.jpg]

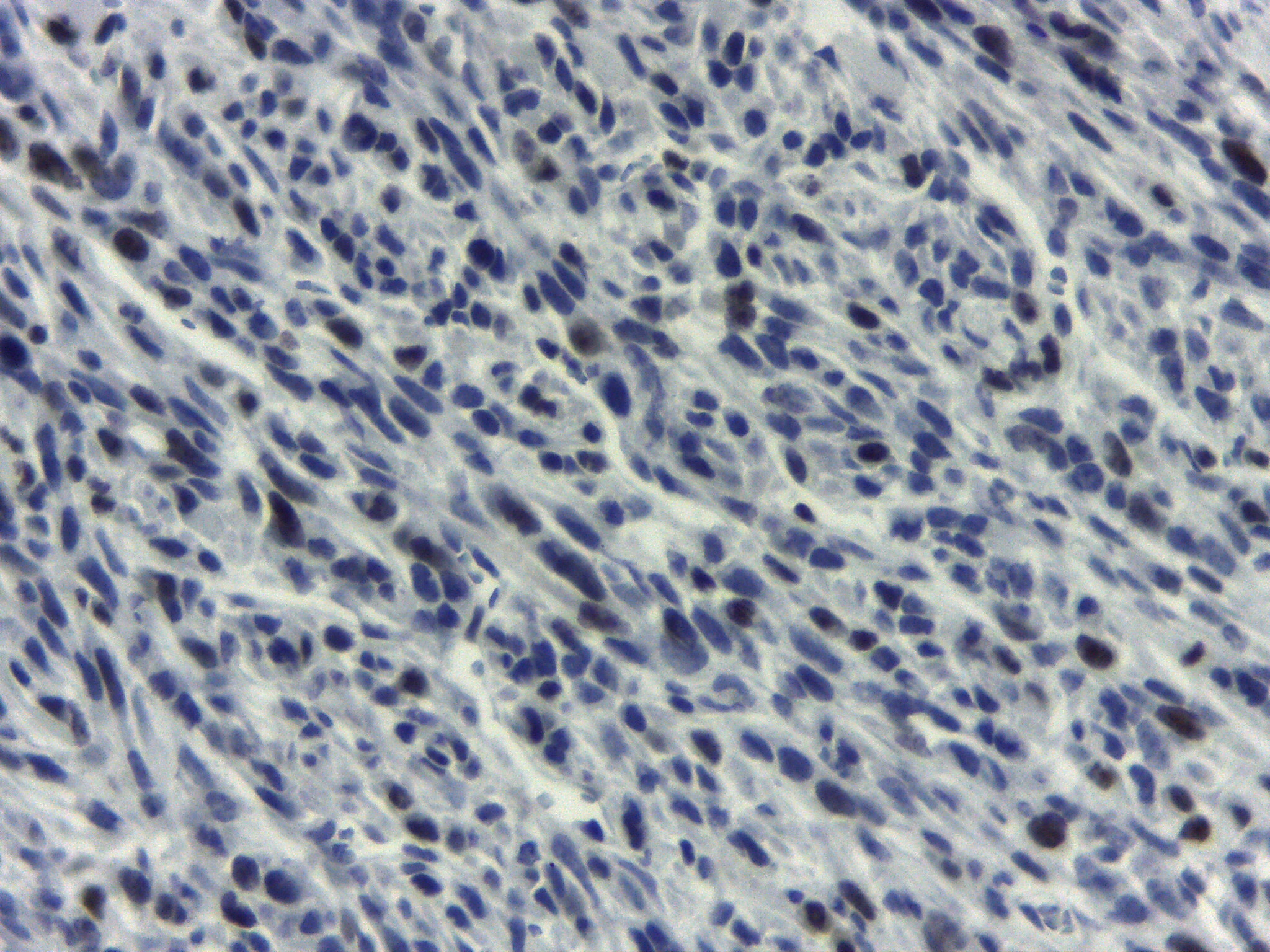

Supplement: S2 Fig — (ZIP) [file pone.0188960.s015.zip › Ki-67 IHC image con/Ki-67 con5-1.jpg]

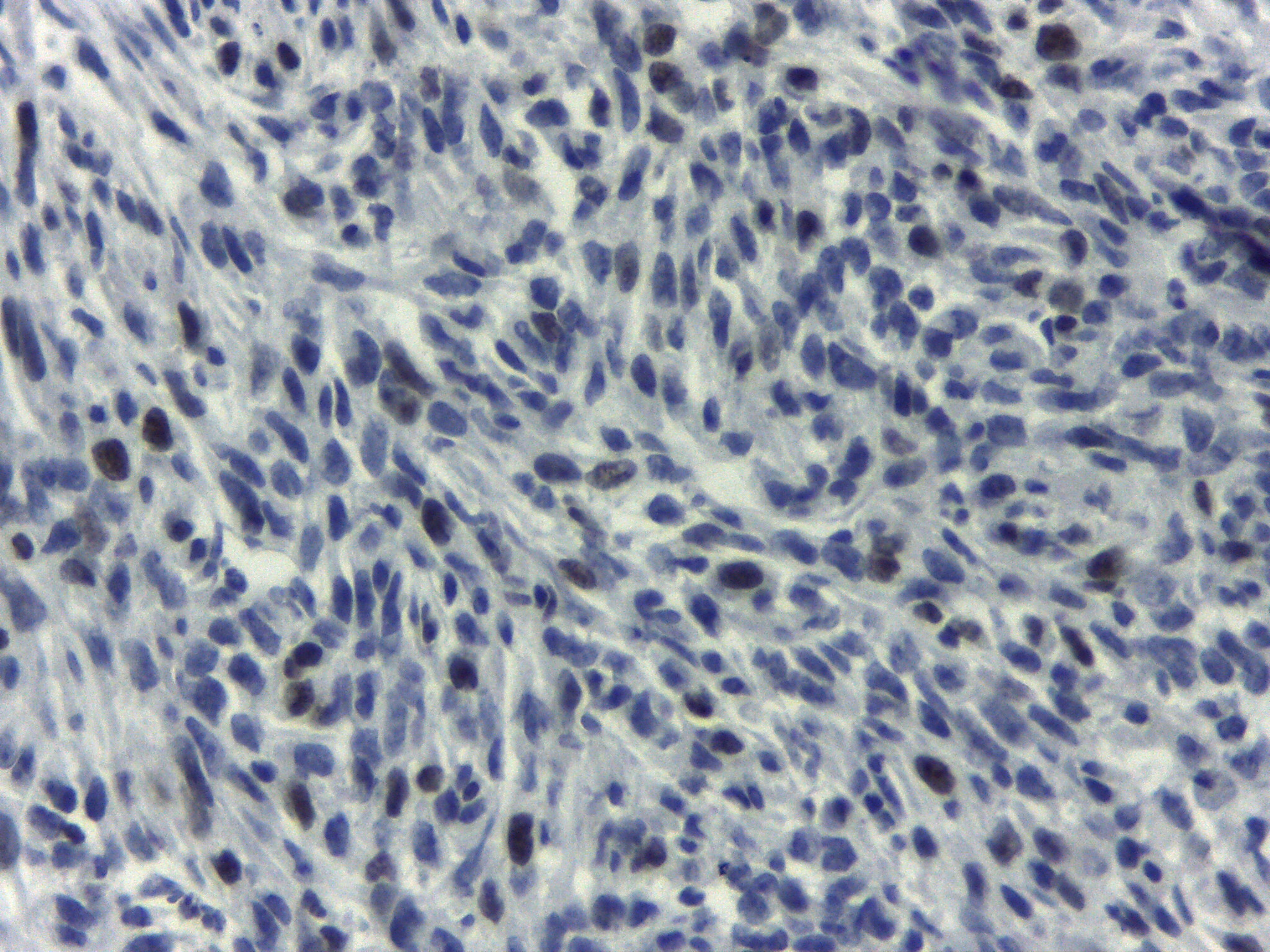

Supplement: S2 Fig — (ZIP) [file pone.0188960.s015.zip › Ki-67 IHC image con/Ki-67 con5-2.jpg]

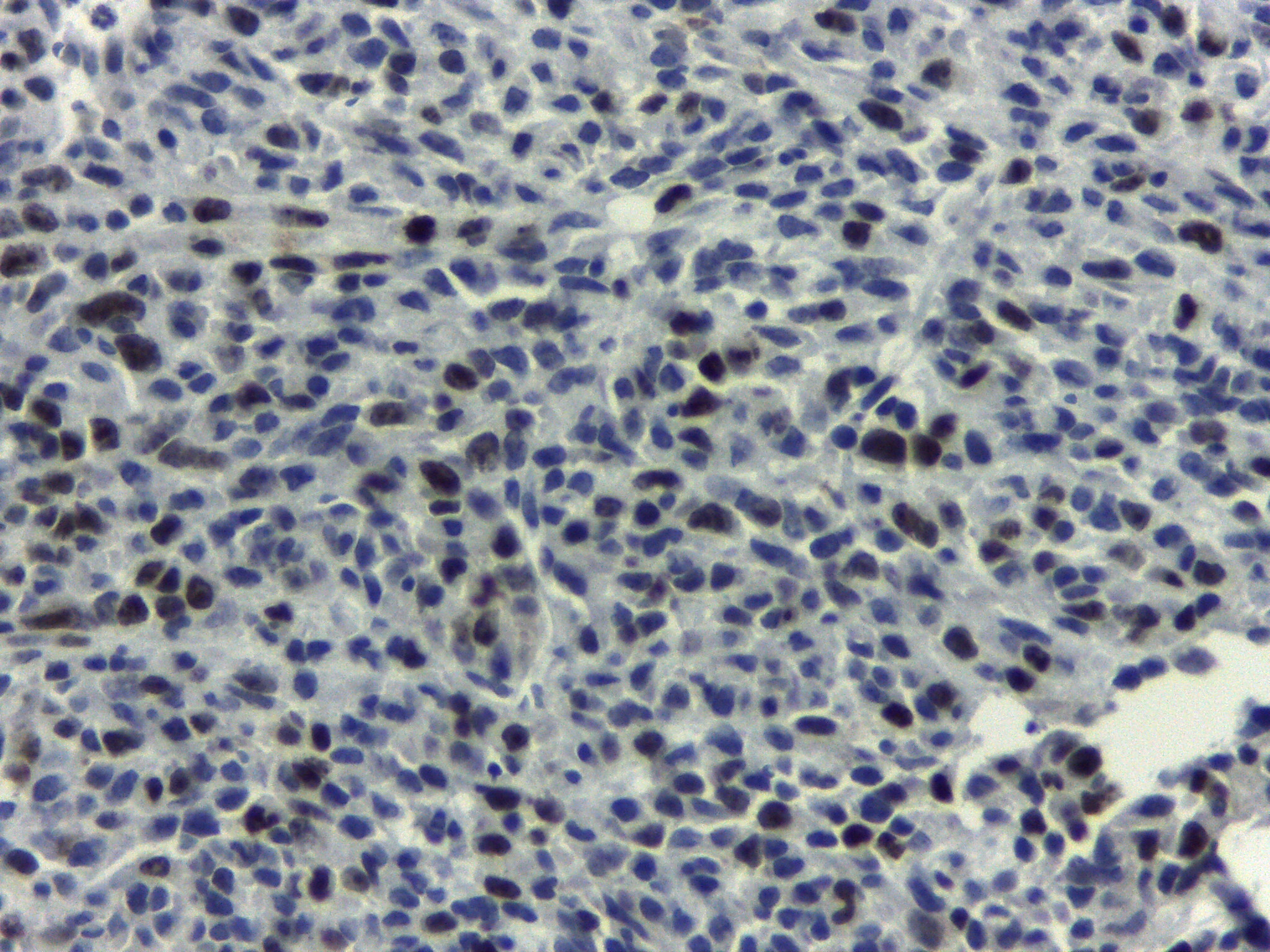

Supplement: S2 Fig — (ZIP) [file pone.0188960.s015.zip › Ki-67 IHC image con/Ki-67 con5-3.jpg]

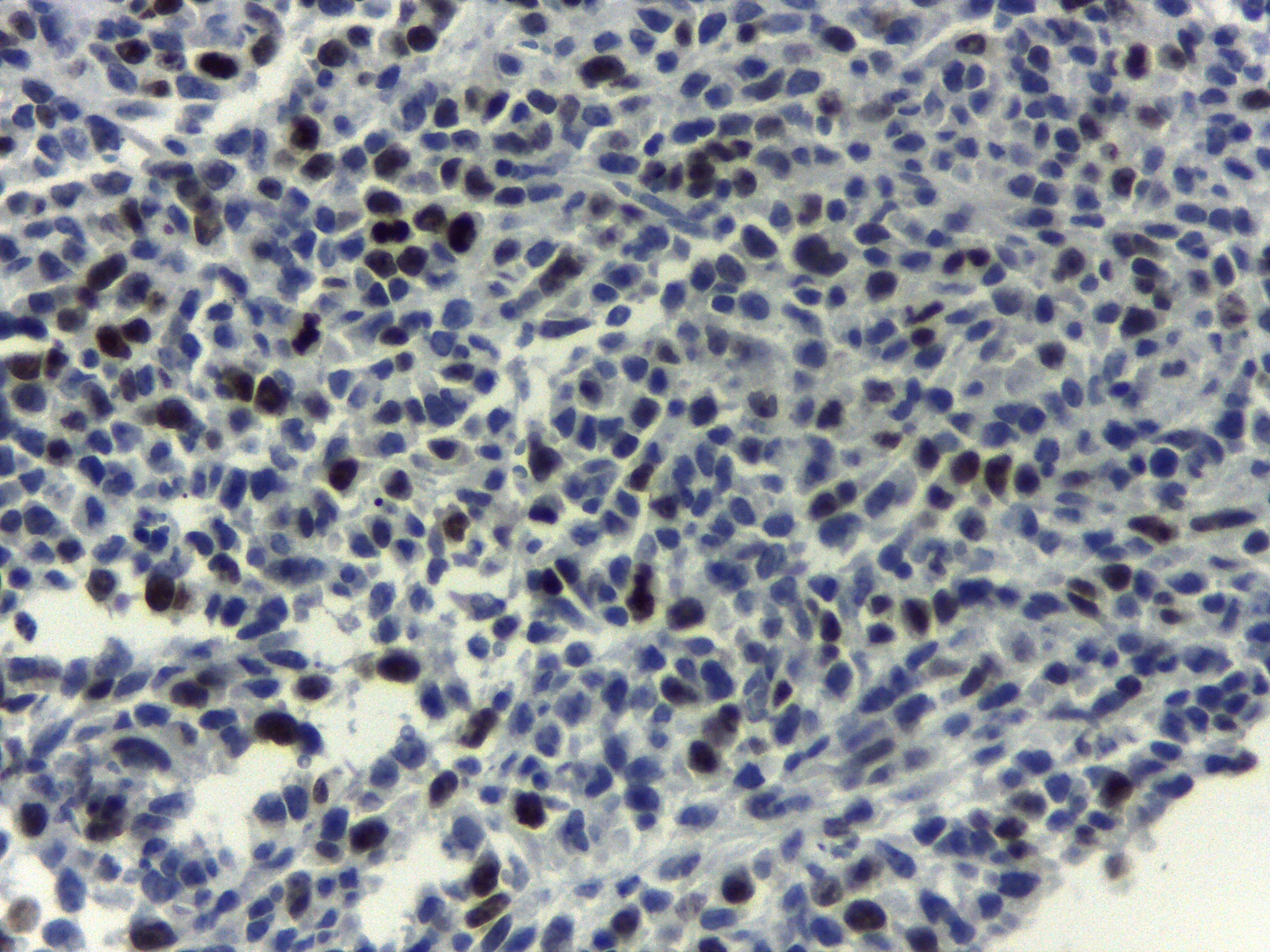

Supplement: S2 Fig — (ZIP) [file pone.0188960.s015.zip › Ki-67 IHC image con/Ki-67 con5-4.jpg]

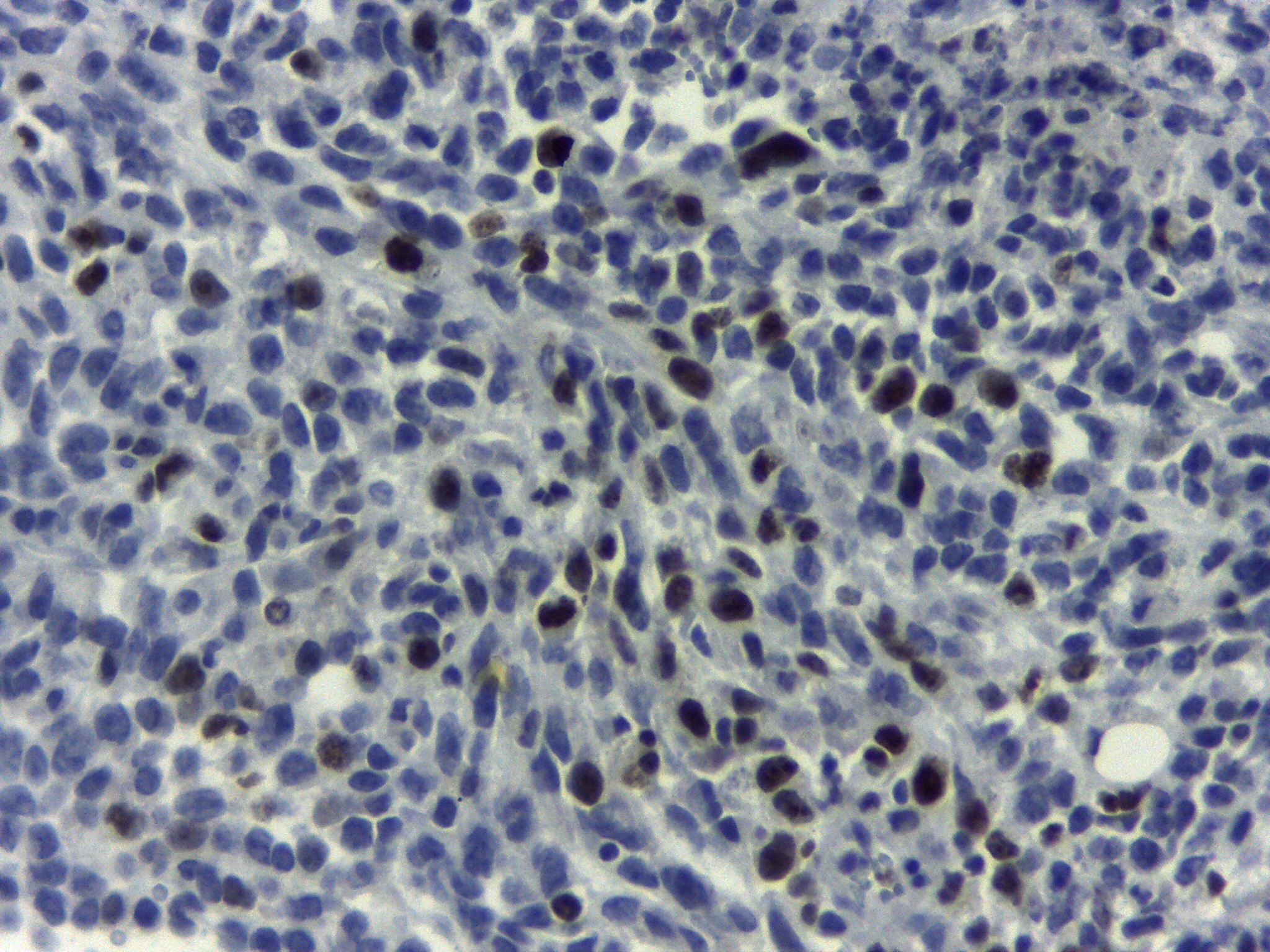

Supplement: S2 Fig — (ZIP) [file pone.0188960.s015.zip › Ki-67 IHC image con/Ki-67 con5-5.jpg]

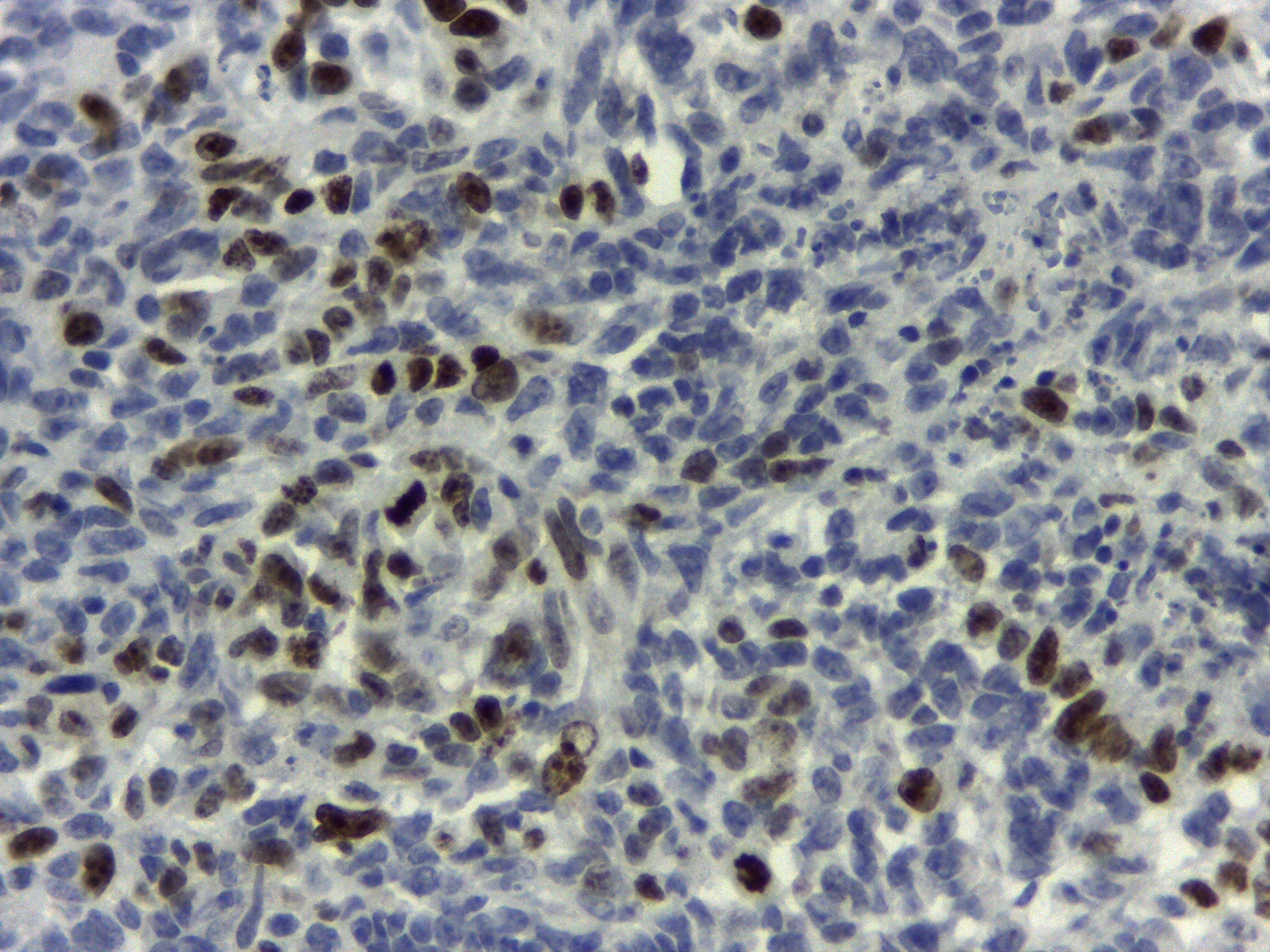

Supplement: S2 Fig — (ZIP) [file pone.0188960.s015.zip › Ki-67 IHC image con/Ki-67 con6-1.jpg]

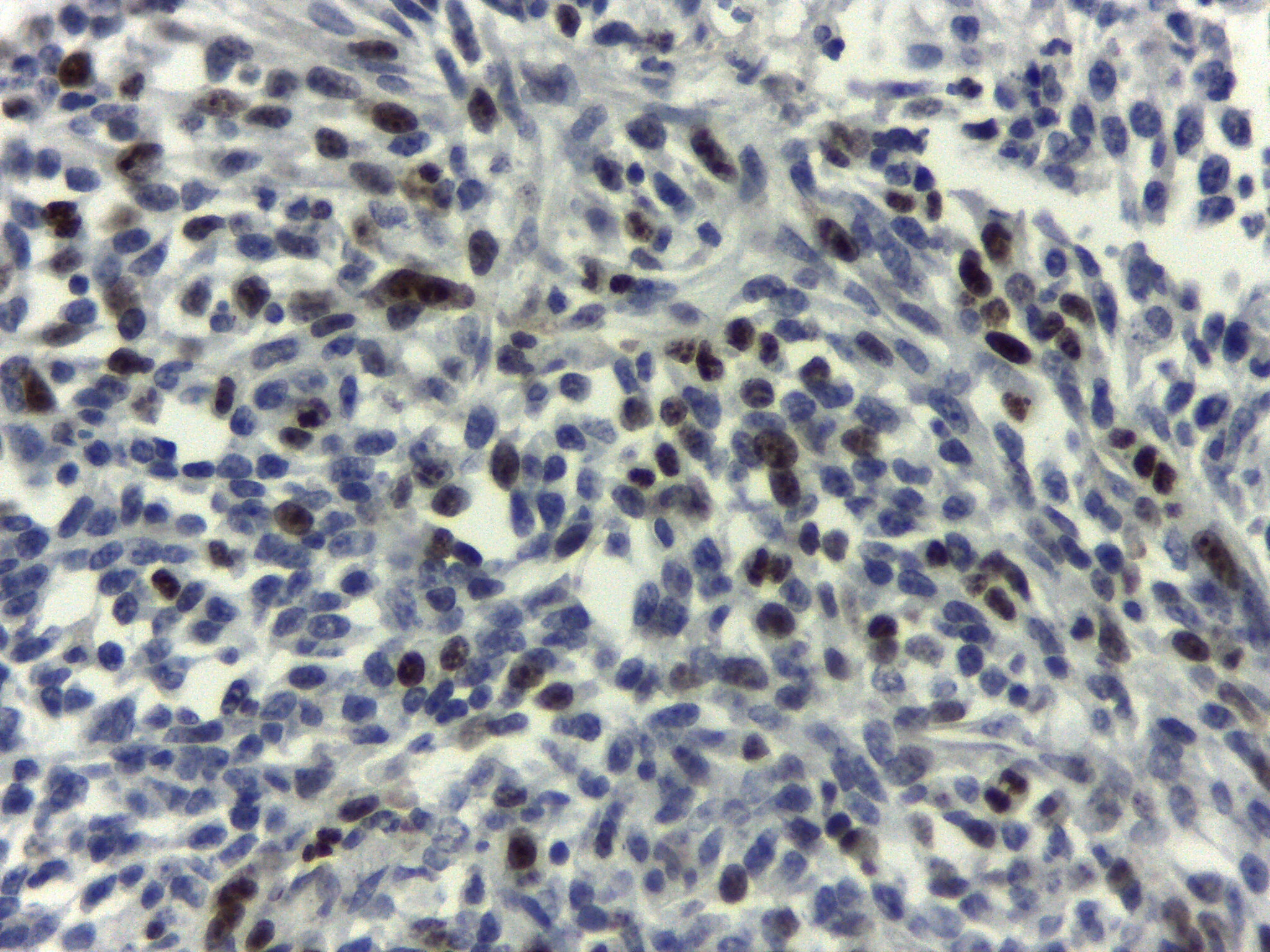

Supplement: S2 Fig — (ZIP) [file pone.0188960.s015.zip › Ki-67 IHC image con/Ki-67 con6-2.jpg]

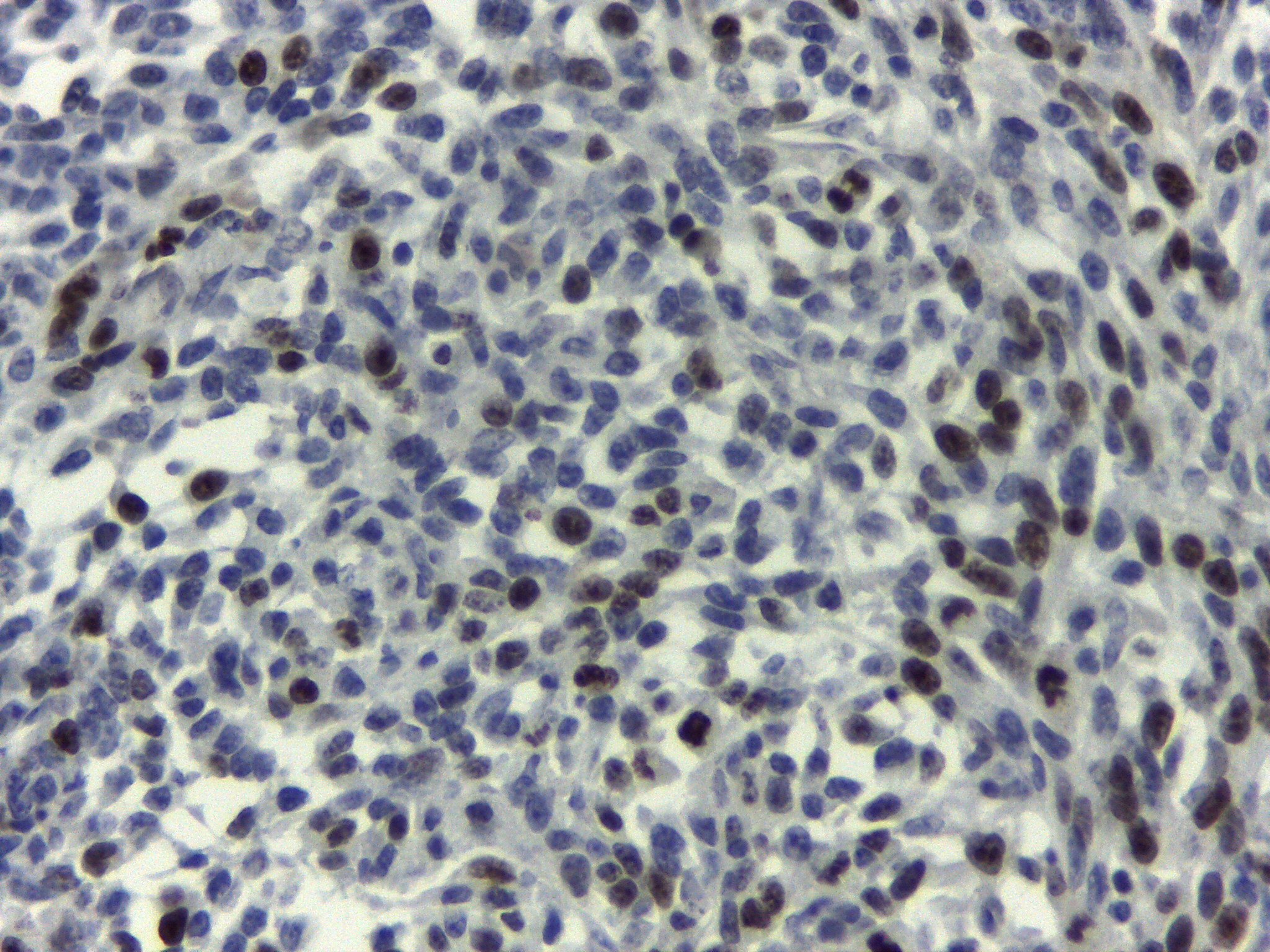

Supplement: S2 Fig — (ZIP) [file pone.0188960.s015.zip › Ki-67 IHC image con/Ki-67 con6-3.jpg]

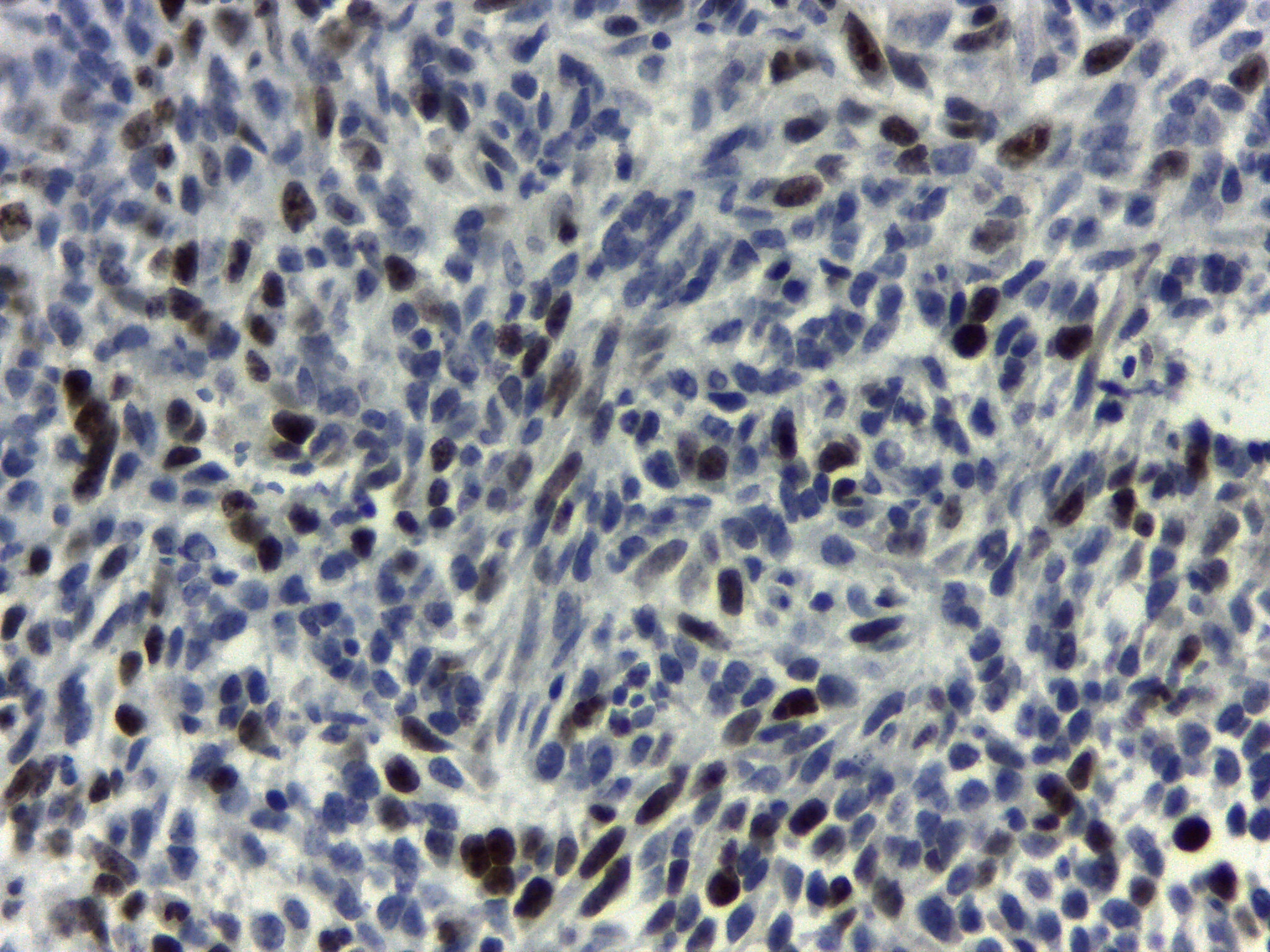

Supplement: S2 Fig — (ZIP) [file pone.0188960.s015.zip › Ki-67 IHC image con/Ki-67 con6-4.jpg]

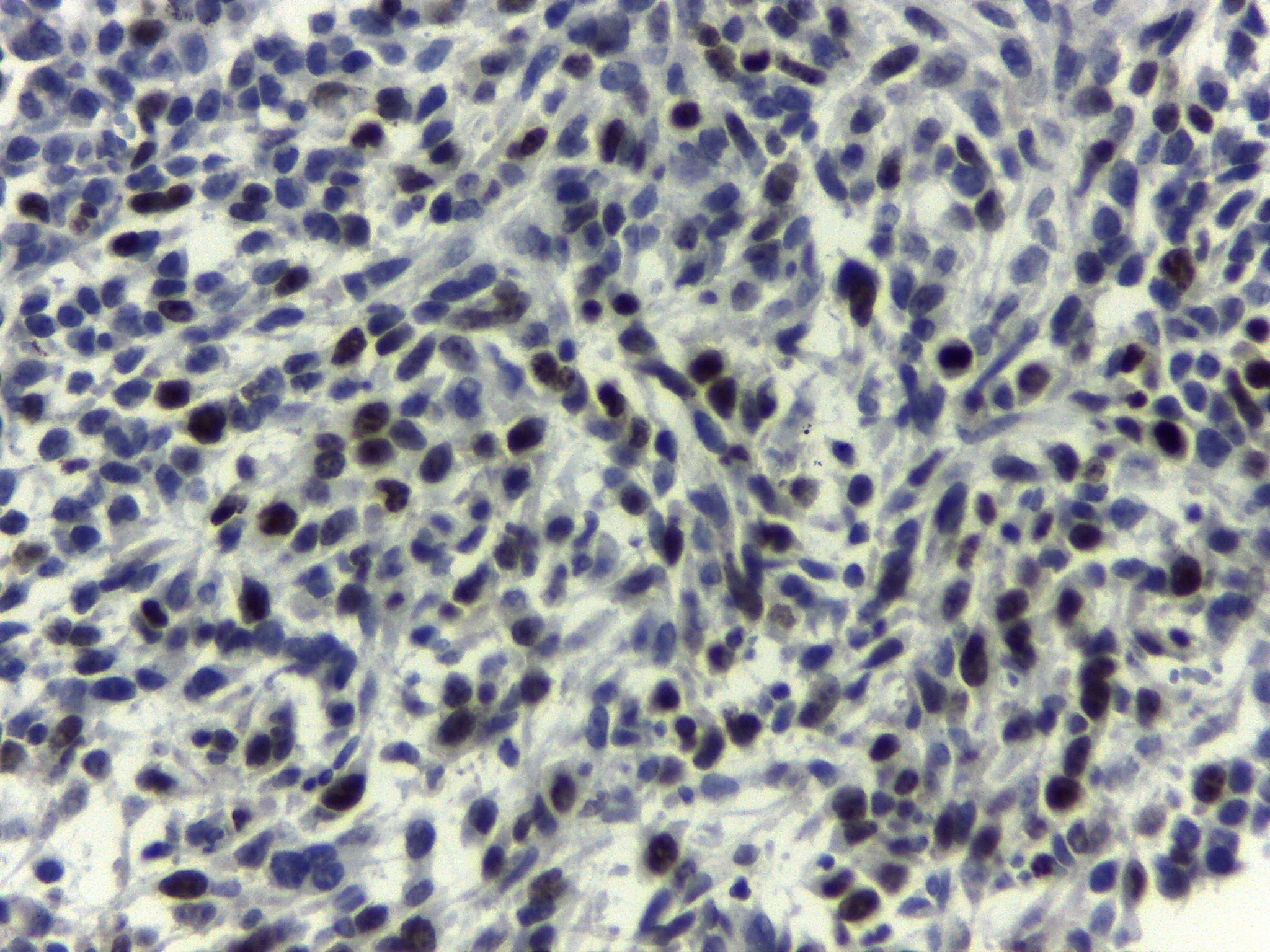

Supplement: S2 Fig — (ZIP) [file pone.0188960.s015.zip › Ki-67 IHC image con/Ki-67 con6-5.jpg]

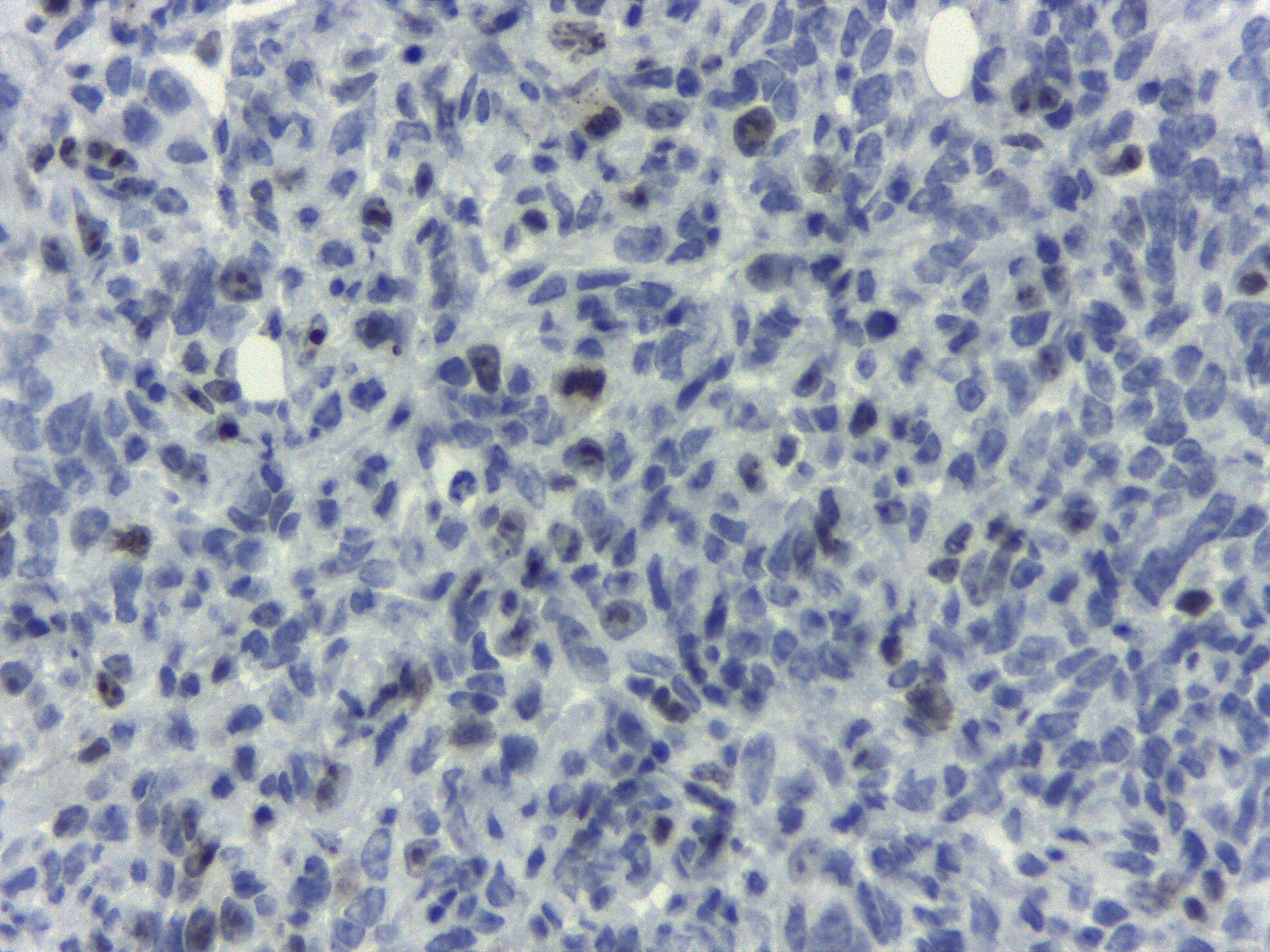

Supplement: S3 Fig — (ZIP) [file pone.0188960.s016.zip › Ki-67 IHC image bac/Ki-67 bac1-1.jpg]

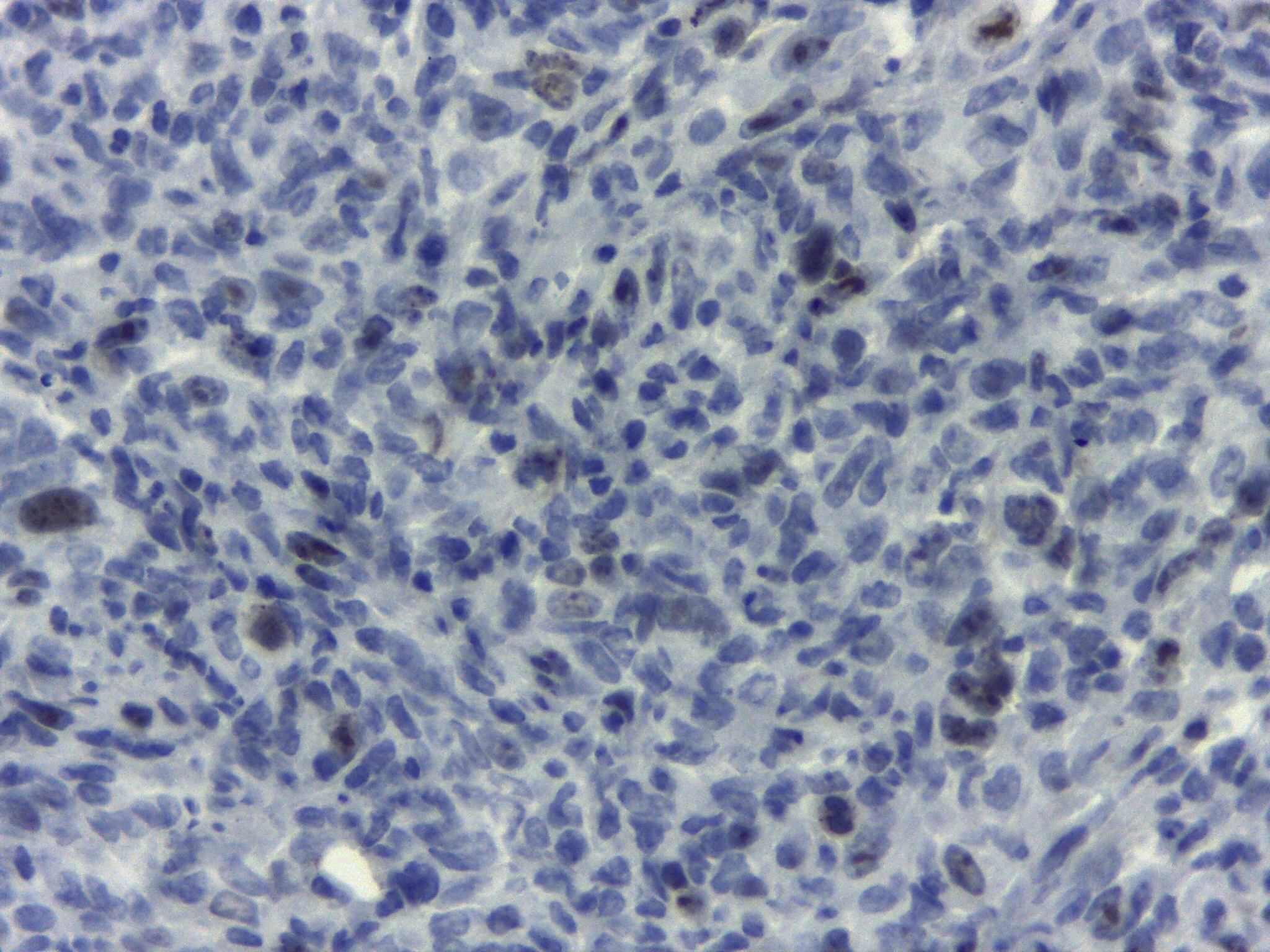

Supplement: S3 Fig — (ZIP) [file pone.0188960.s016.zip › Ki-67 IHC image bac/Ki-67 bac1-2.jpg]

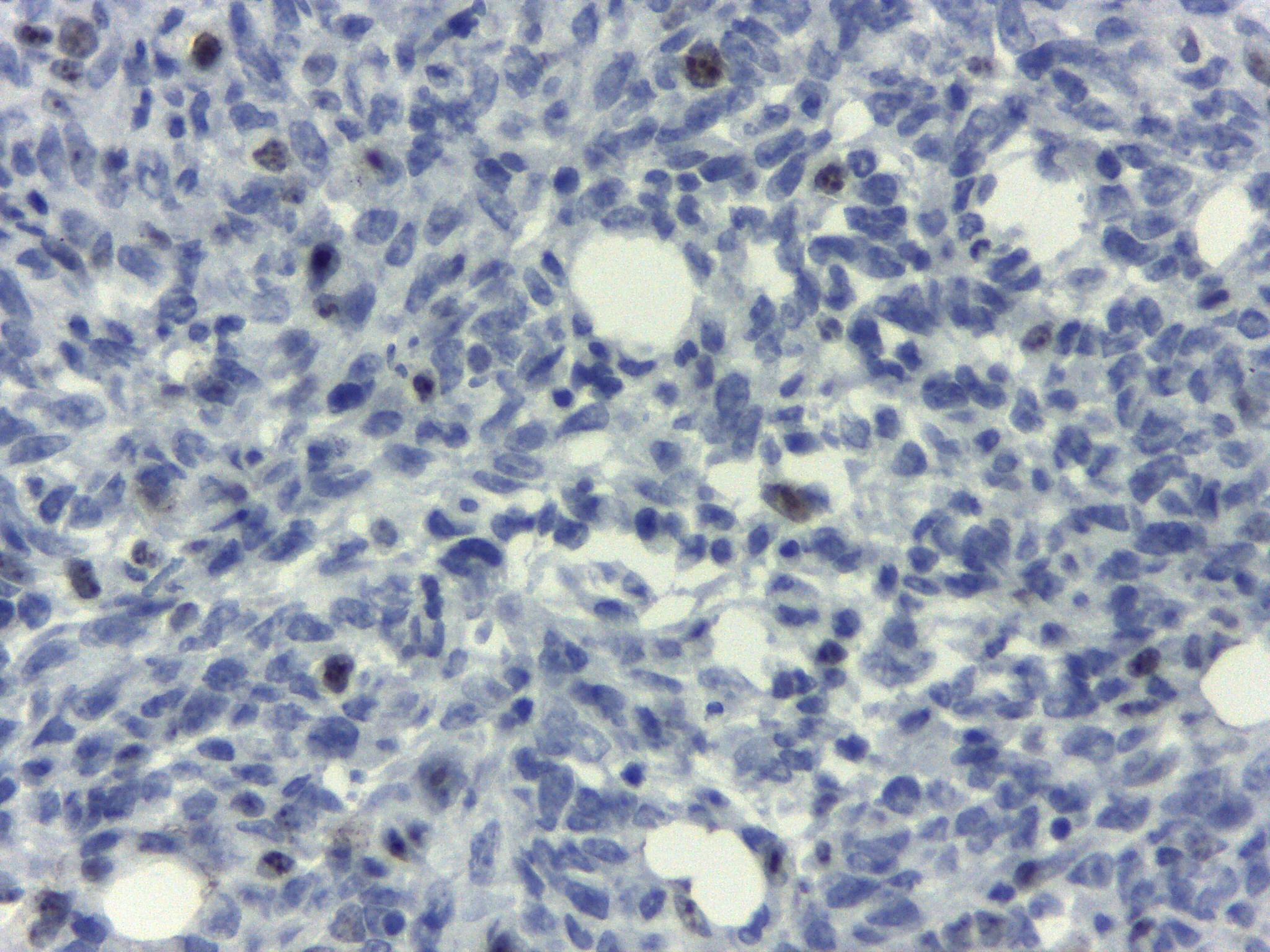

Supplement: S3 Fig — (ZIP) [file pone.0188960.s016.zip › Ki-67 IHC image bac/Ki-67 bac1-3.jpg]

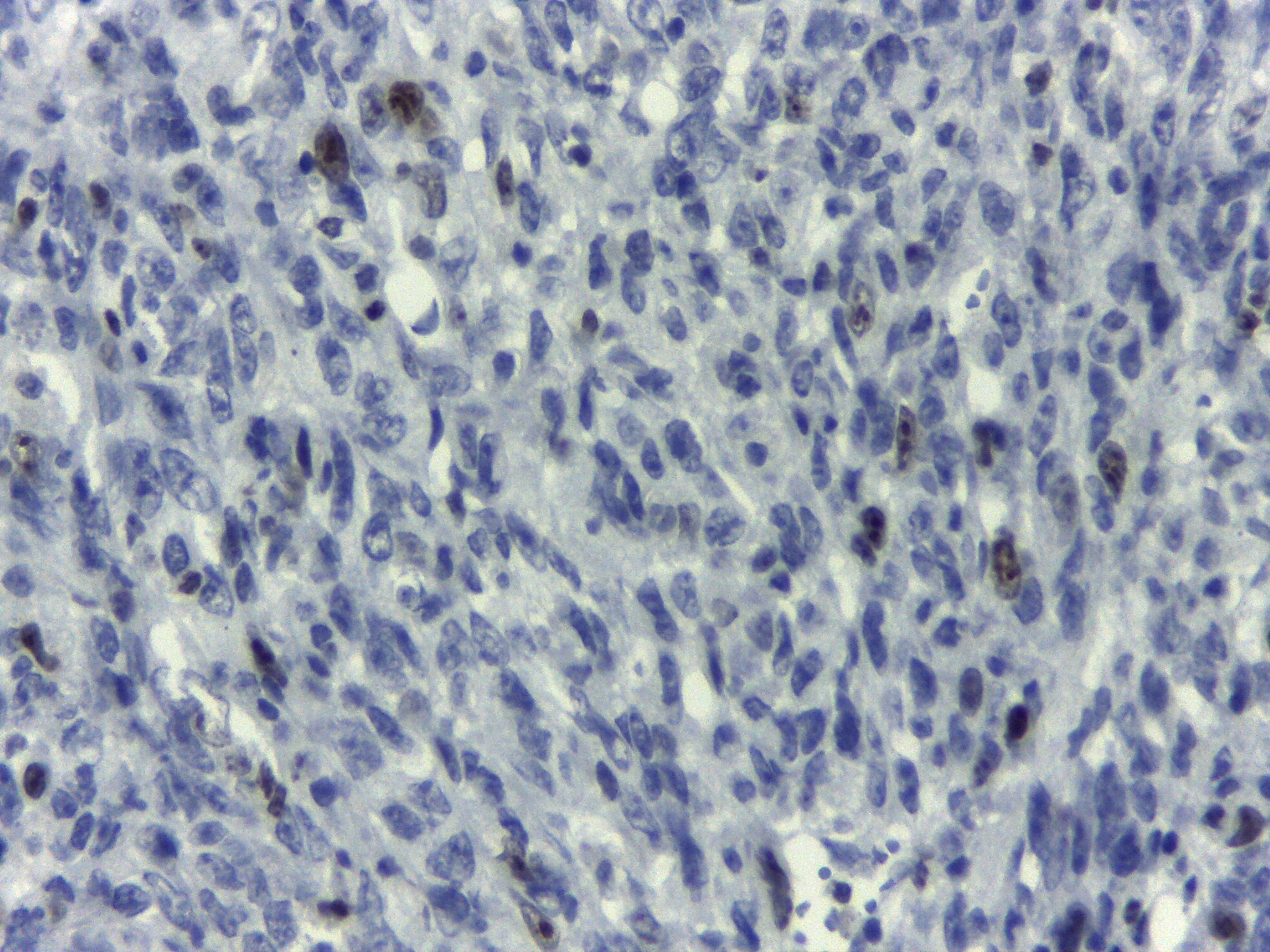

Supplement: S3 Fig — (ZIP) [file pone.0188960.s016.zip › Ki-67 IHC image bac/Ki-67 bac1-4.jpg]

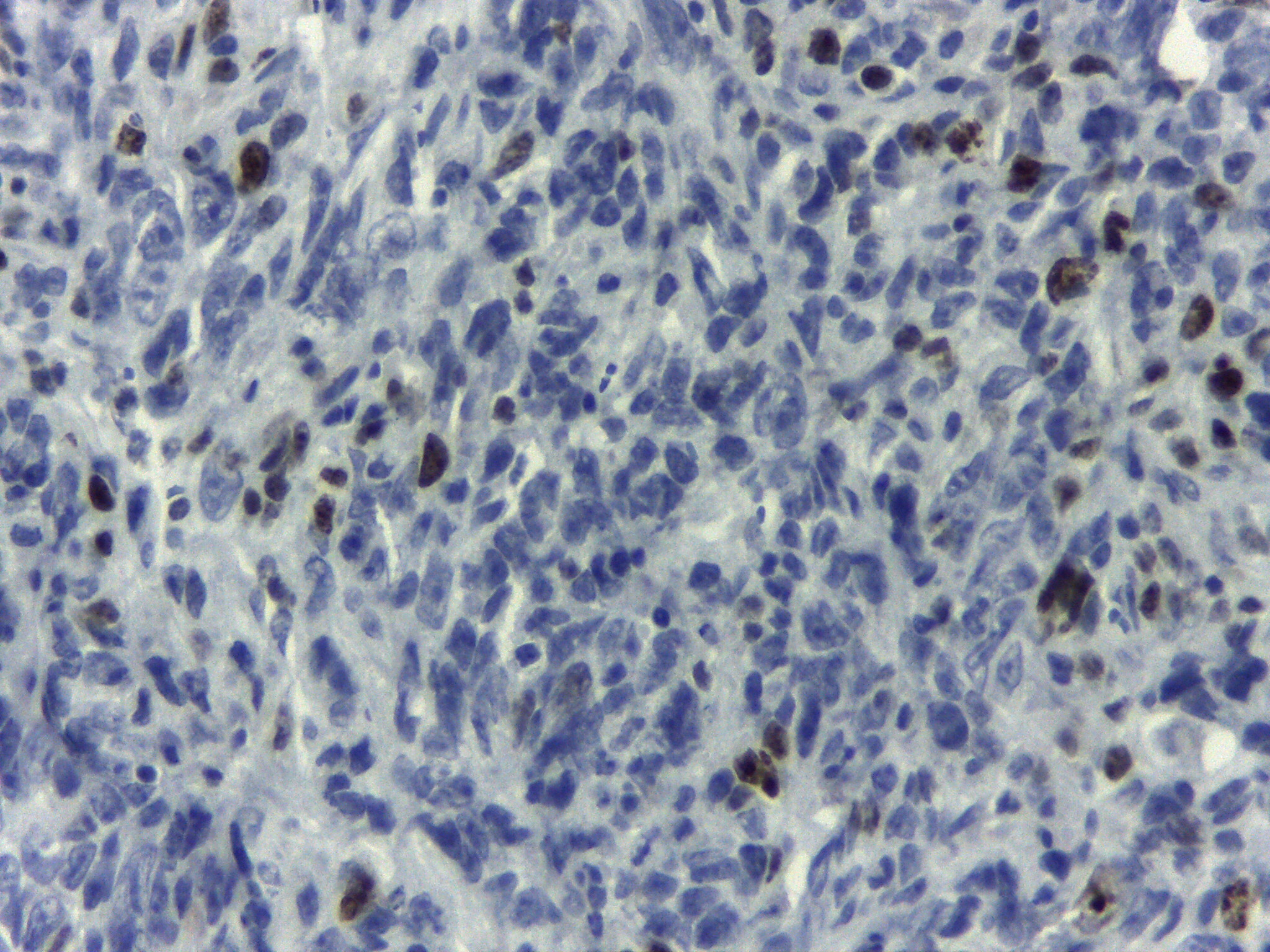

Supplement: S3 Fig — (ZIP) [file pone.0188960.s016.zip › Ki-67 IHC image bac/Ki-67 bac1-5.jpg]

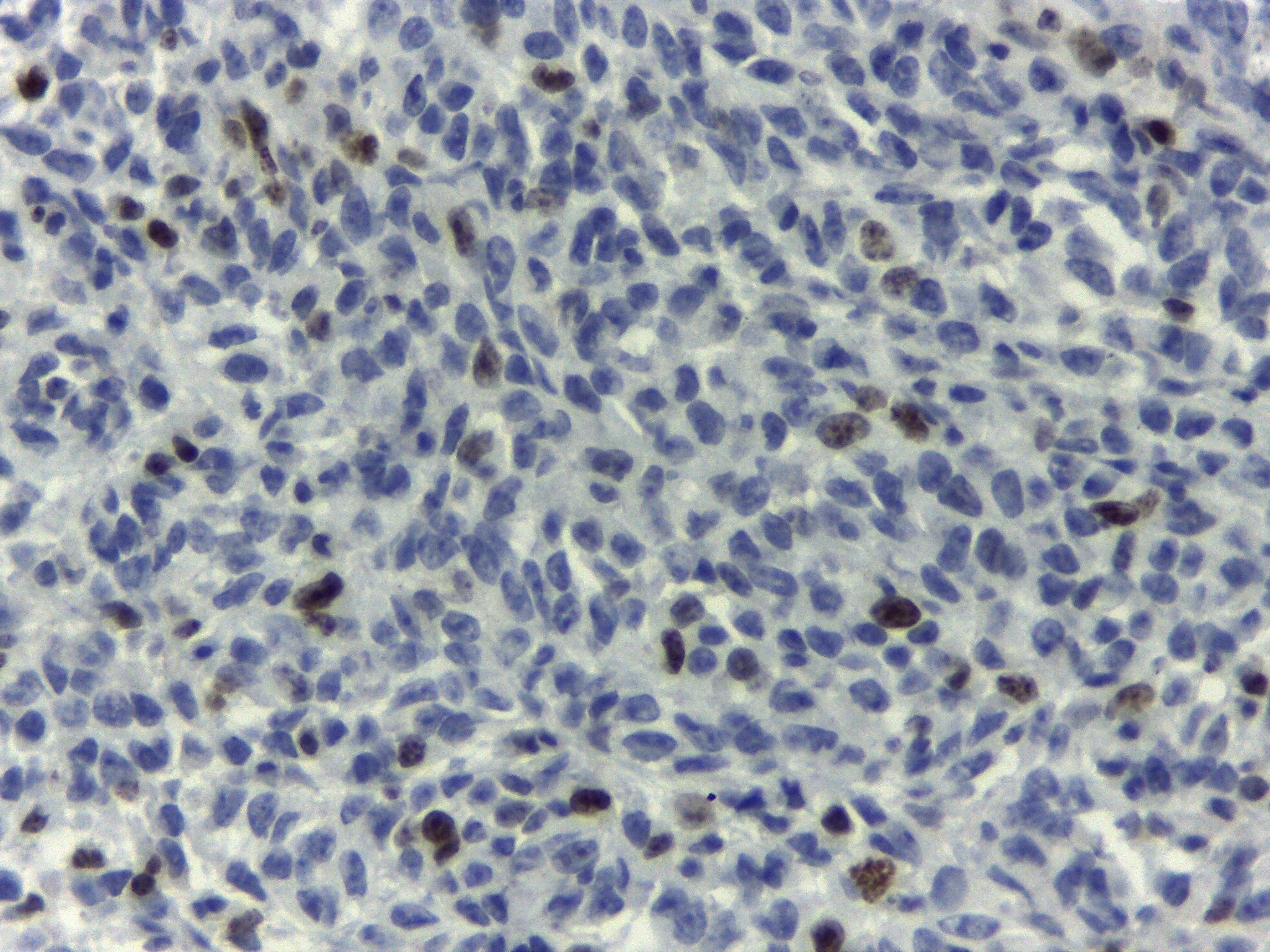

Supplement: S3 Fig — (ZIP) [file pone.0188960.s016.zip › Ki-67 IHC image bac/Ki-67 bac2-1.jpg]

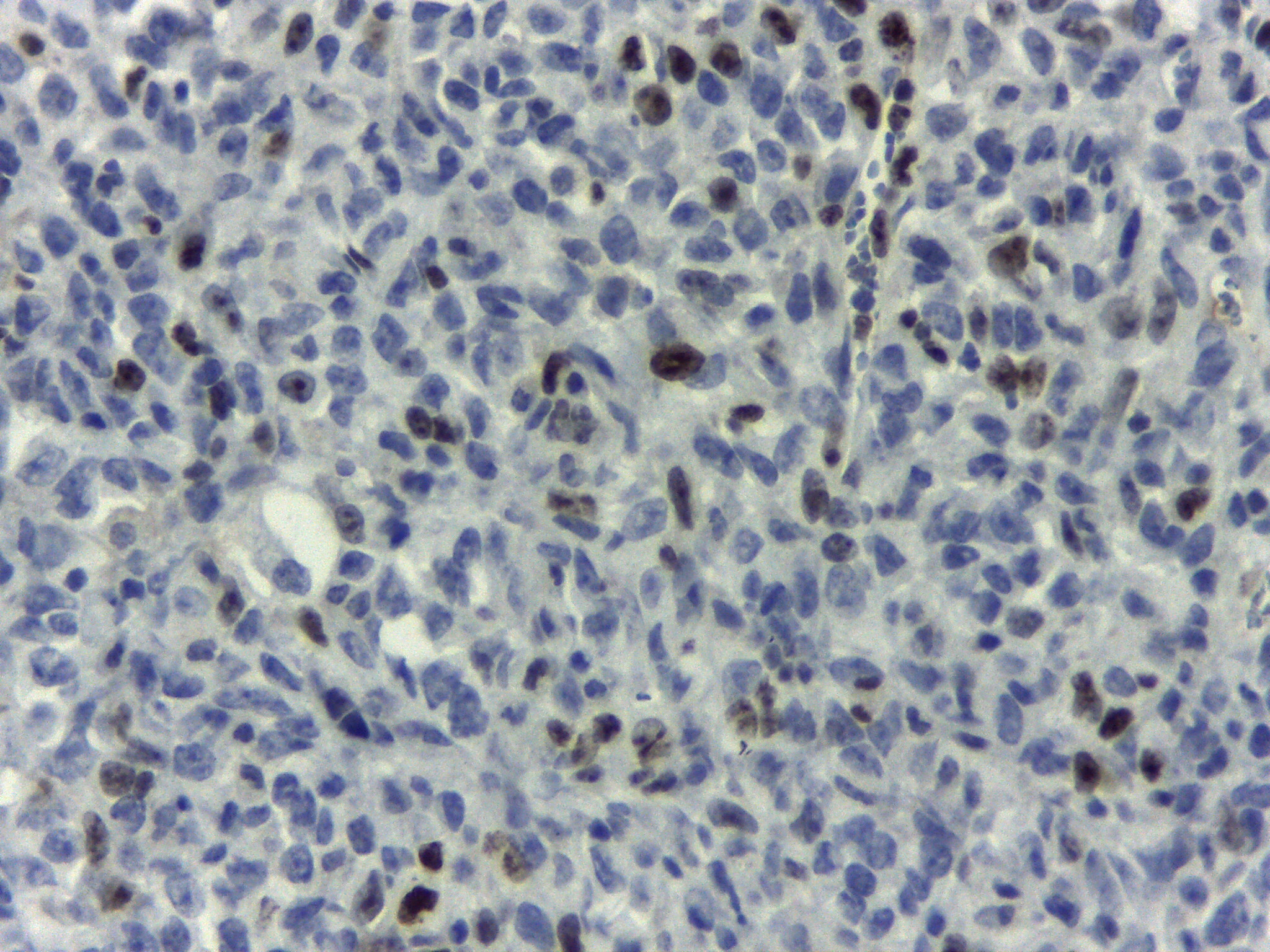

Supplement: S3 Fig — (ZIP) [file pone.0188960.s016.zip › Ki-67 IHC image bac/Ki-67 bac2-2.jpg]

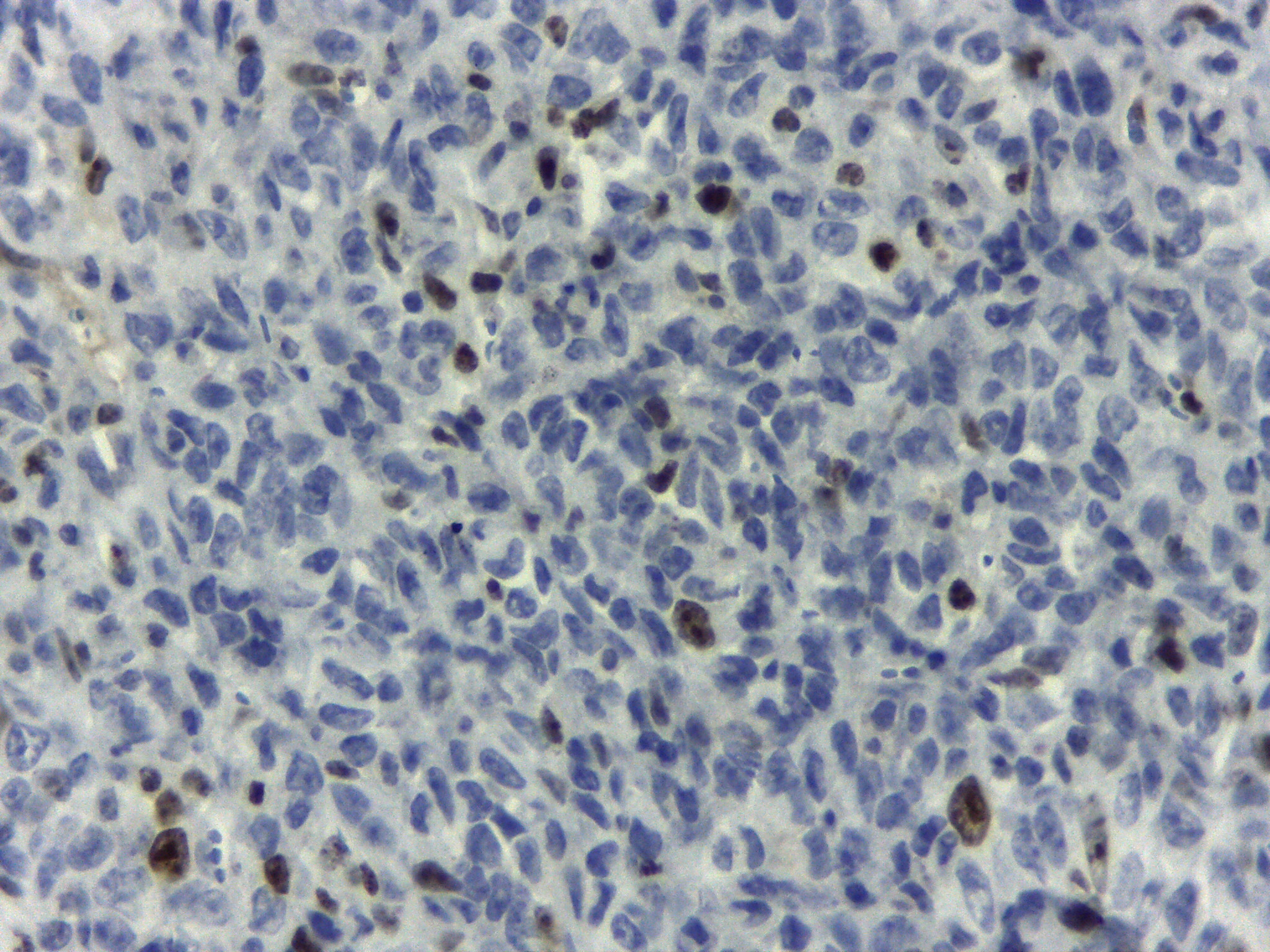

Supplement: S3 Fig — (ZIP) [file pone.0188960.s016.zip › Ki-67 IHC image bac/Ki-67 bac2-3.jpg]

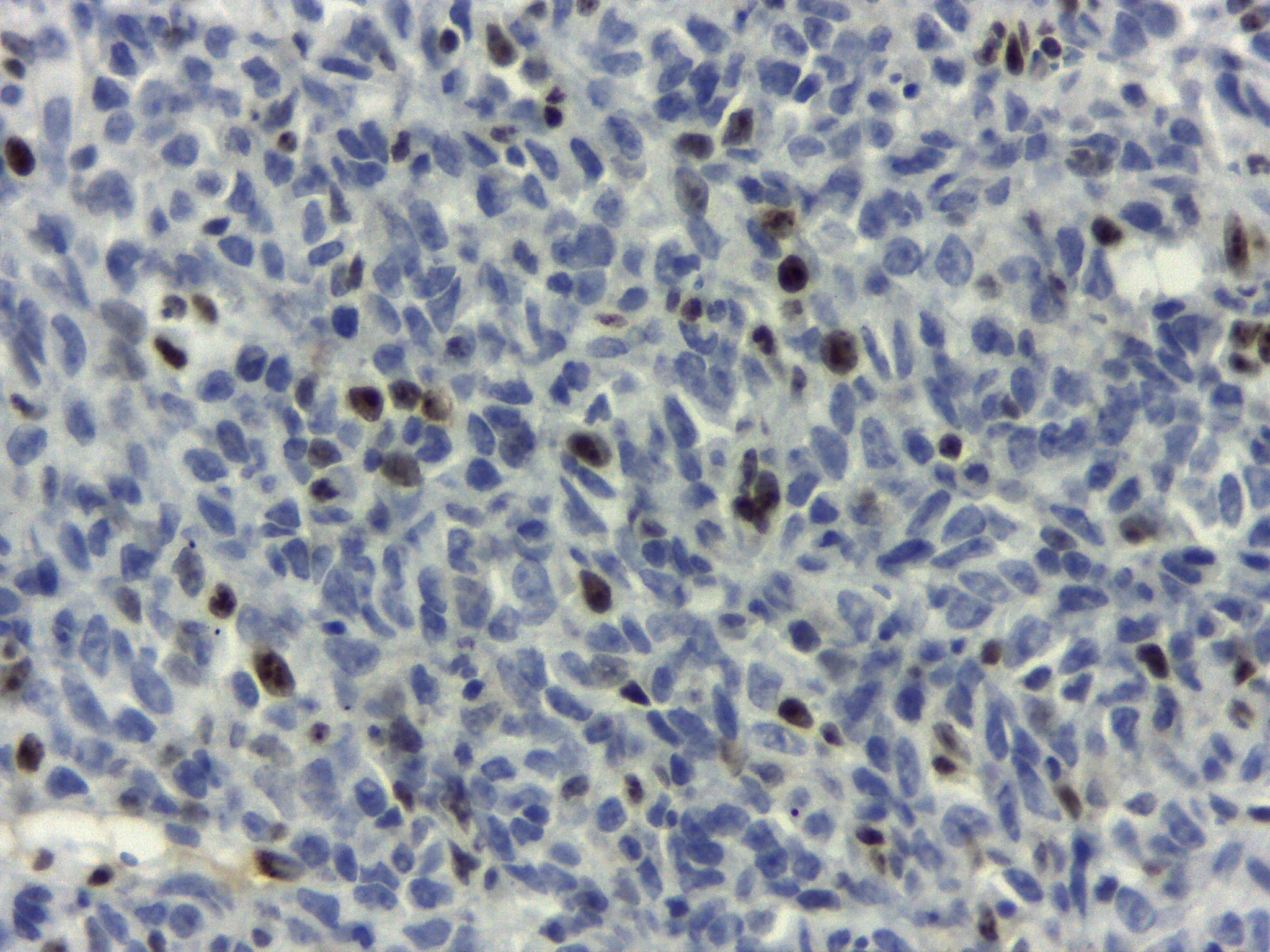

Supplement: S3 Fig — (ZIP) [file pone.0188960.s016.zip › Ki-67 IHC image bac/Ki-67 bac2-4.jpg]

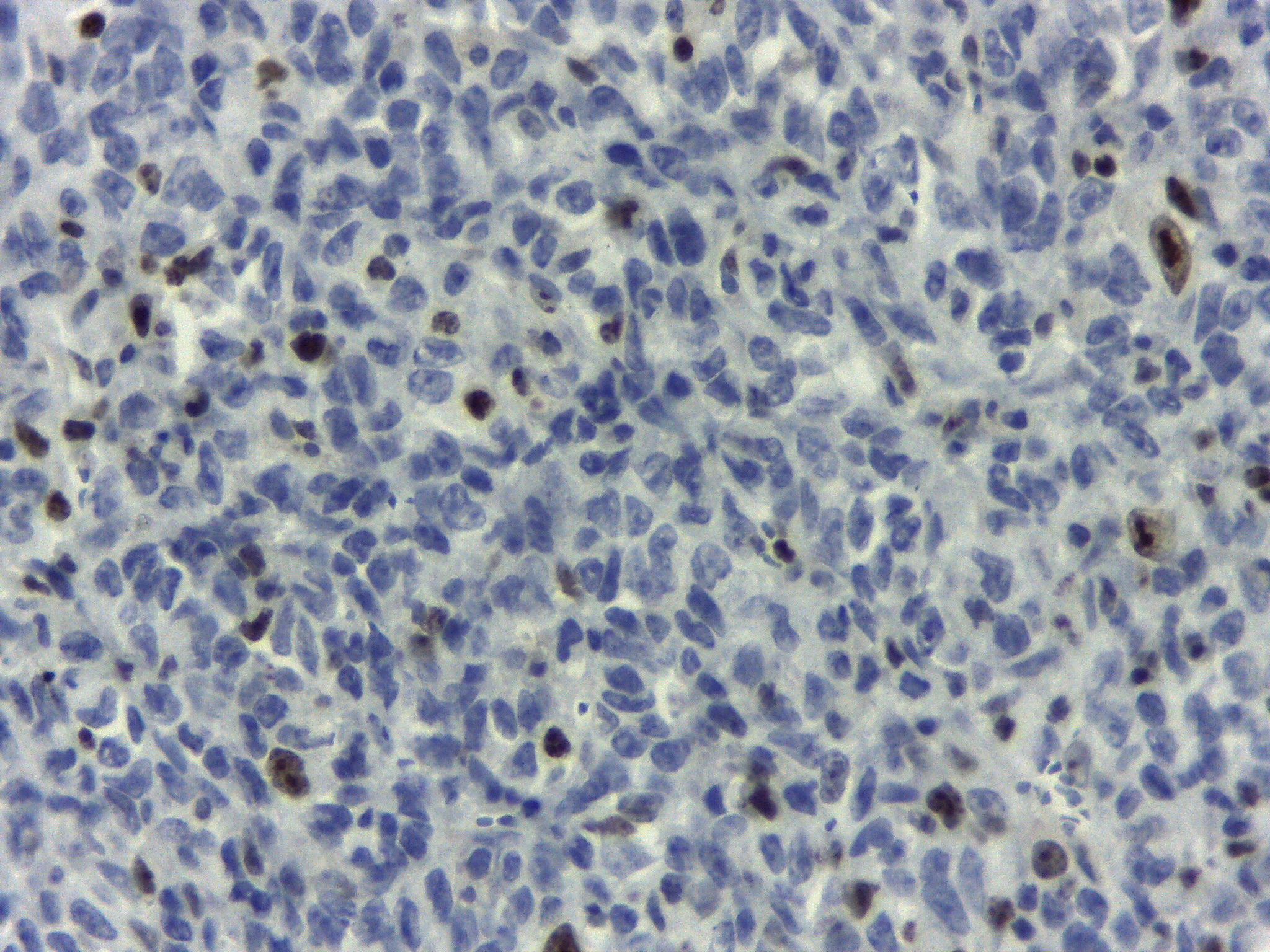

Supplement: S3 Fig — (ZIP) [file pone.0188960.s016.zip › Ki-67 IHC image bac/Ki-67 bac2-5.jpg]

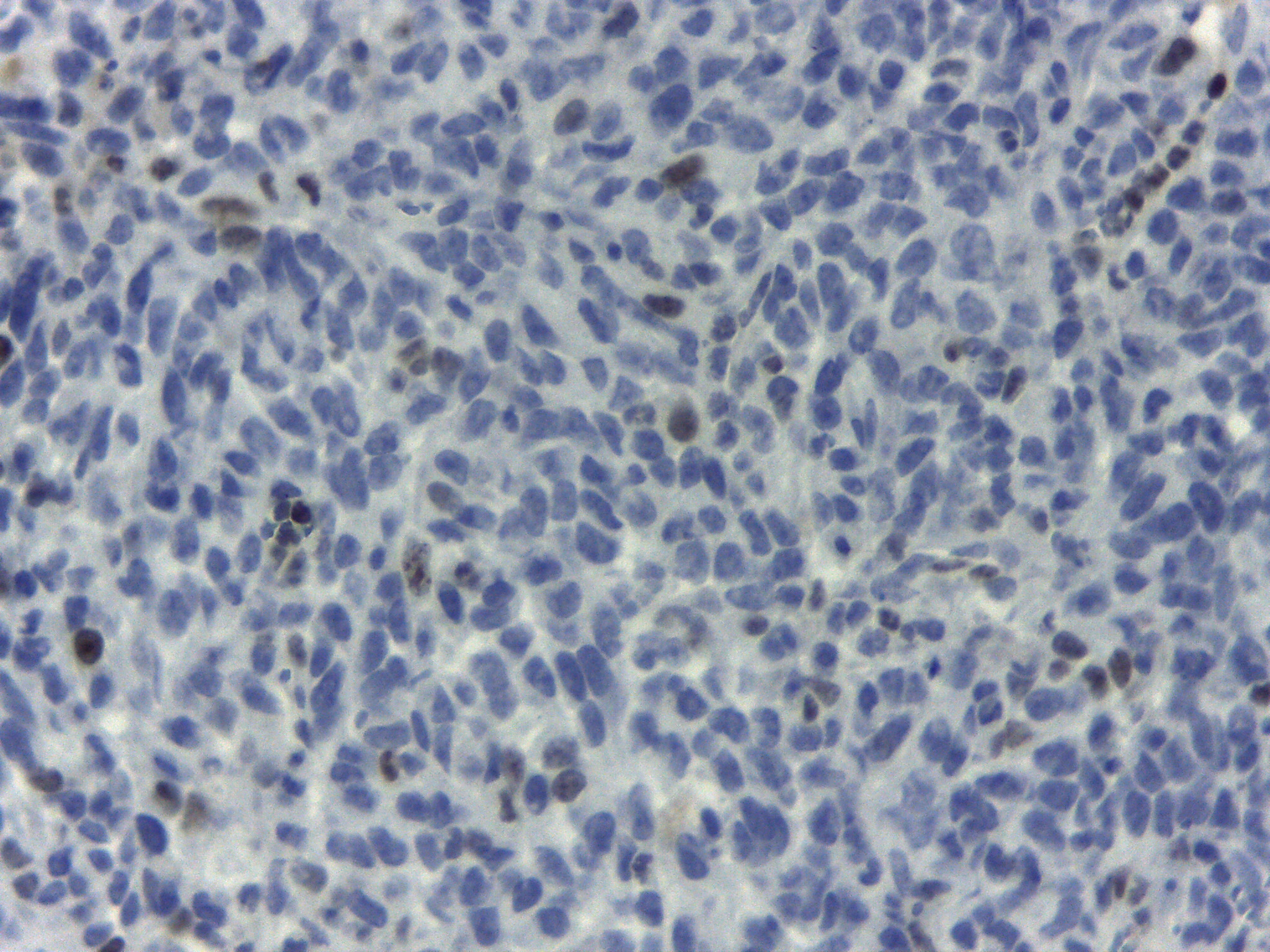

Supplement: S3 Fig — (ZIP) [file pone.0188960.s016.zip › Ki-67 IHC image bac/Ki-67 bac3-1.jpg]

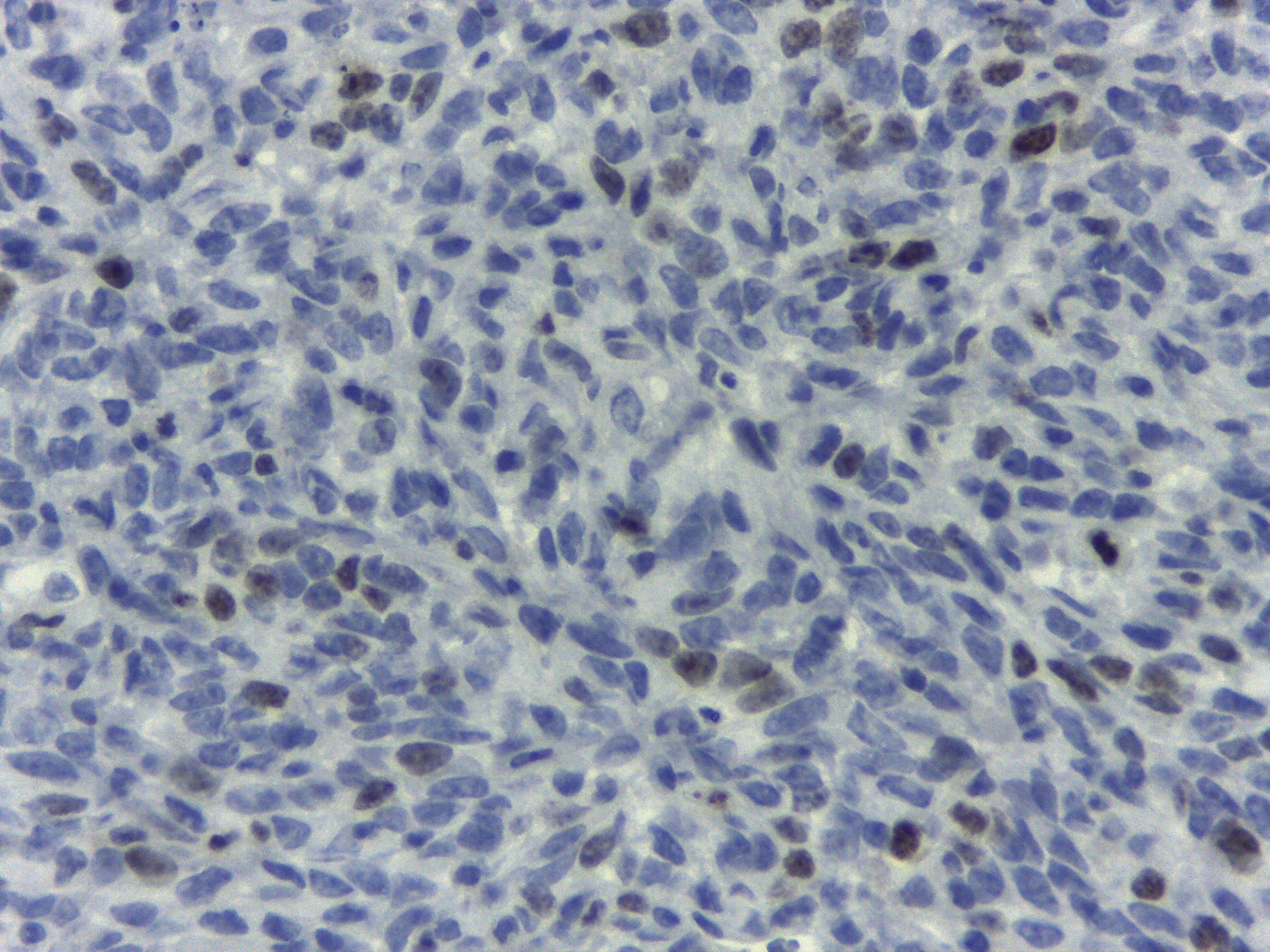

Supplement: S3 Fig — (ZIP) [file pone.0188960.s016.zip › Ki-67 IHC image bac/Ki-67 bac3-2.jpg]

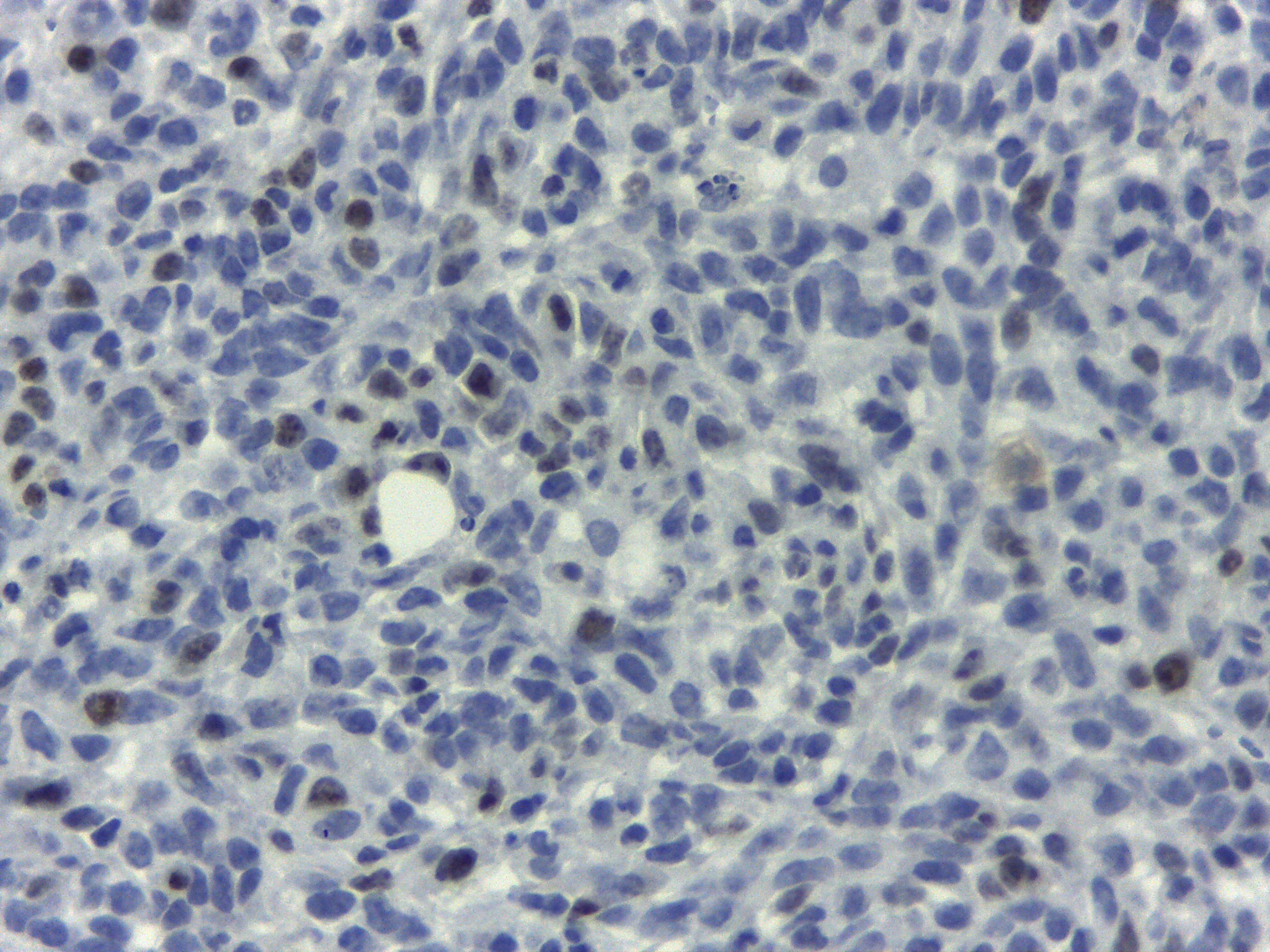

Supplement: S3 Fig — (ZIP) [file pone.0188960.s016.zip › Ki-67 IHC image bac/Ki-67 bac3-3.jpg]

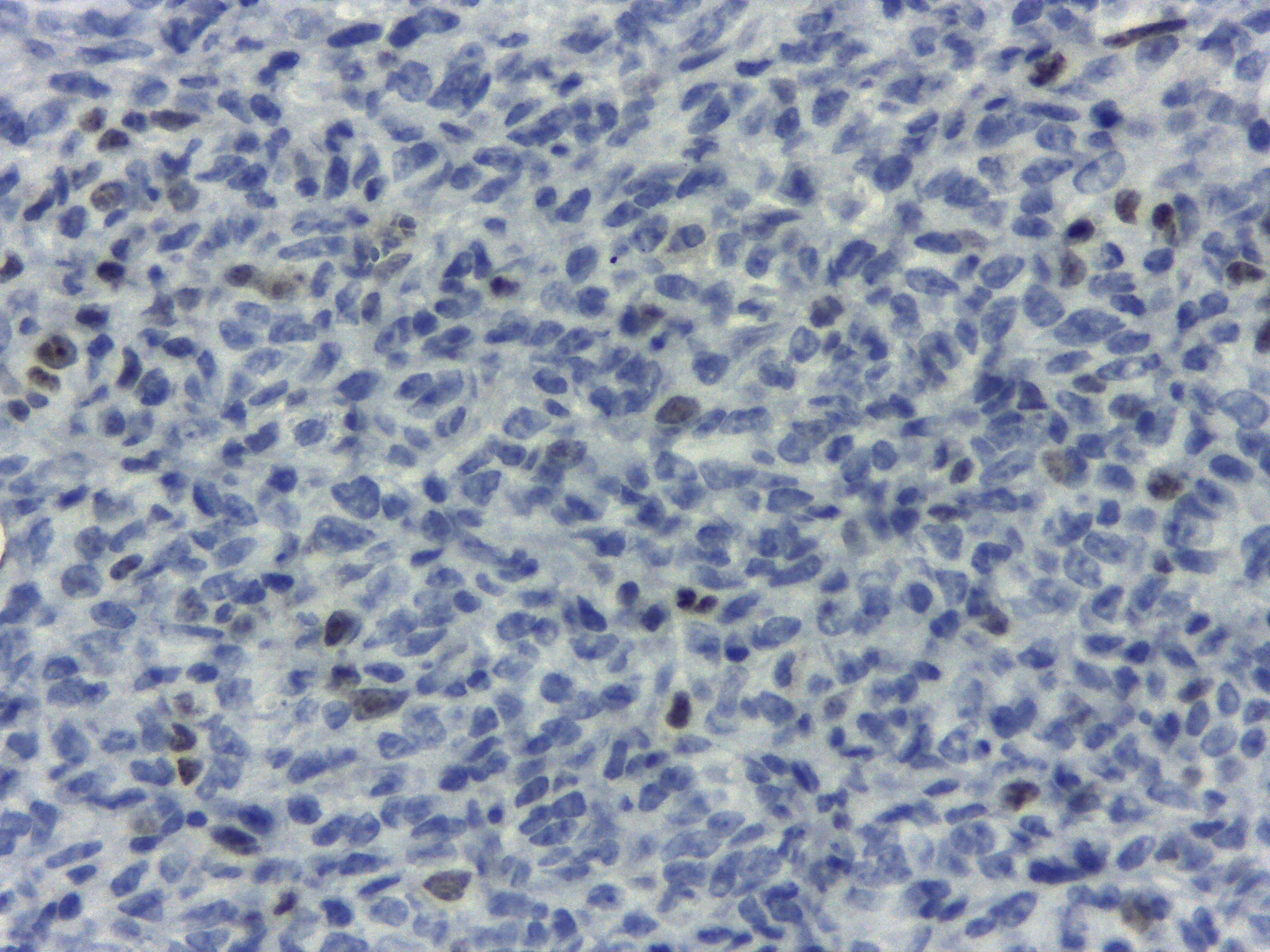

Supplement: S3 Fig — (ZIP) [file pone.0188960.s016.zip › Ki-67 IHC image bac/Ki-67 bac3-4.jpg]

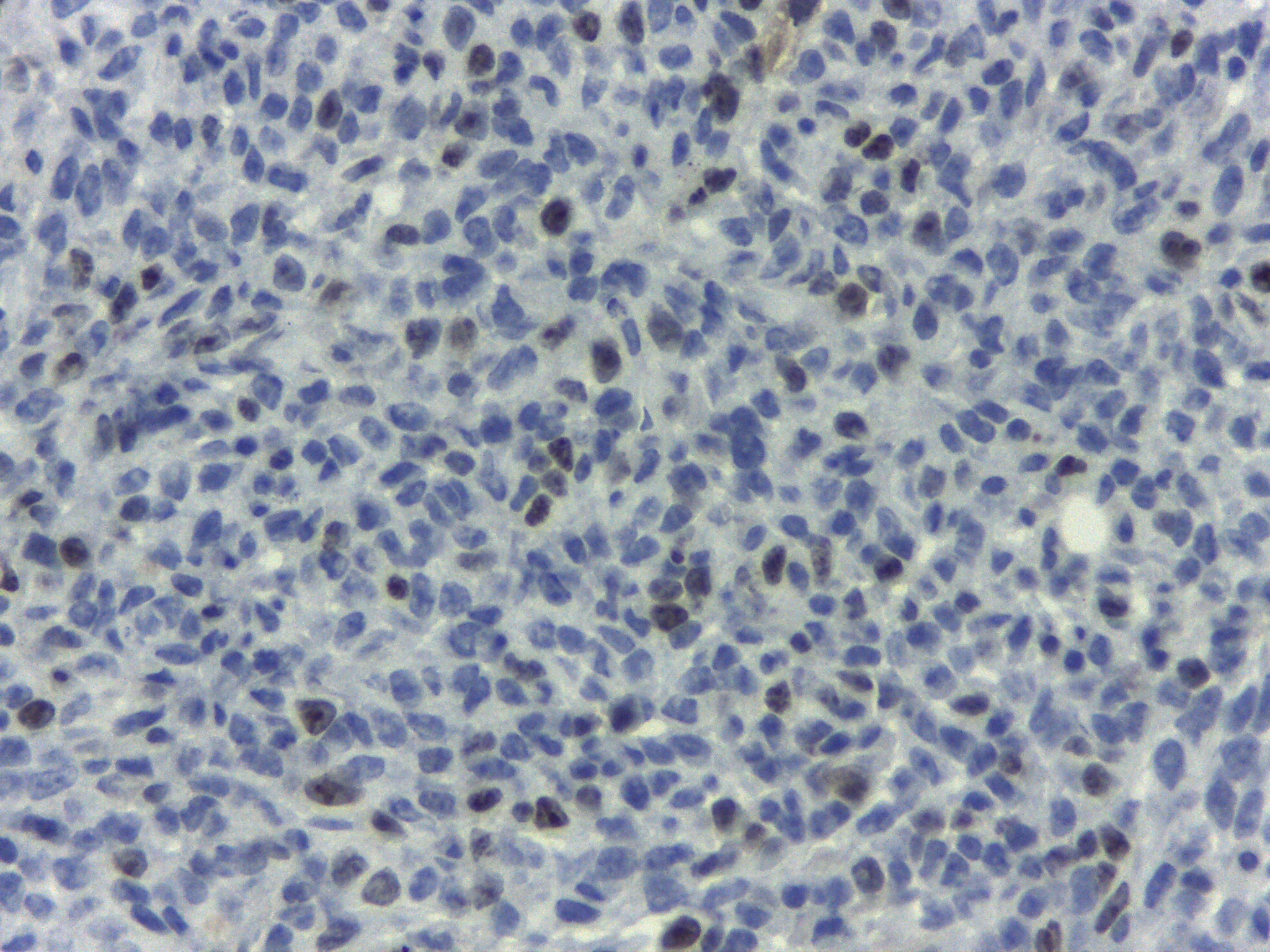

Supplement: S3 Fig — (ZIP) [file pone.0188960.s016.zip › Ki-67 IHC image bac/Ki-67 bac3-5.jpg]

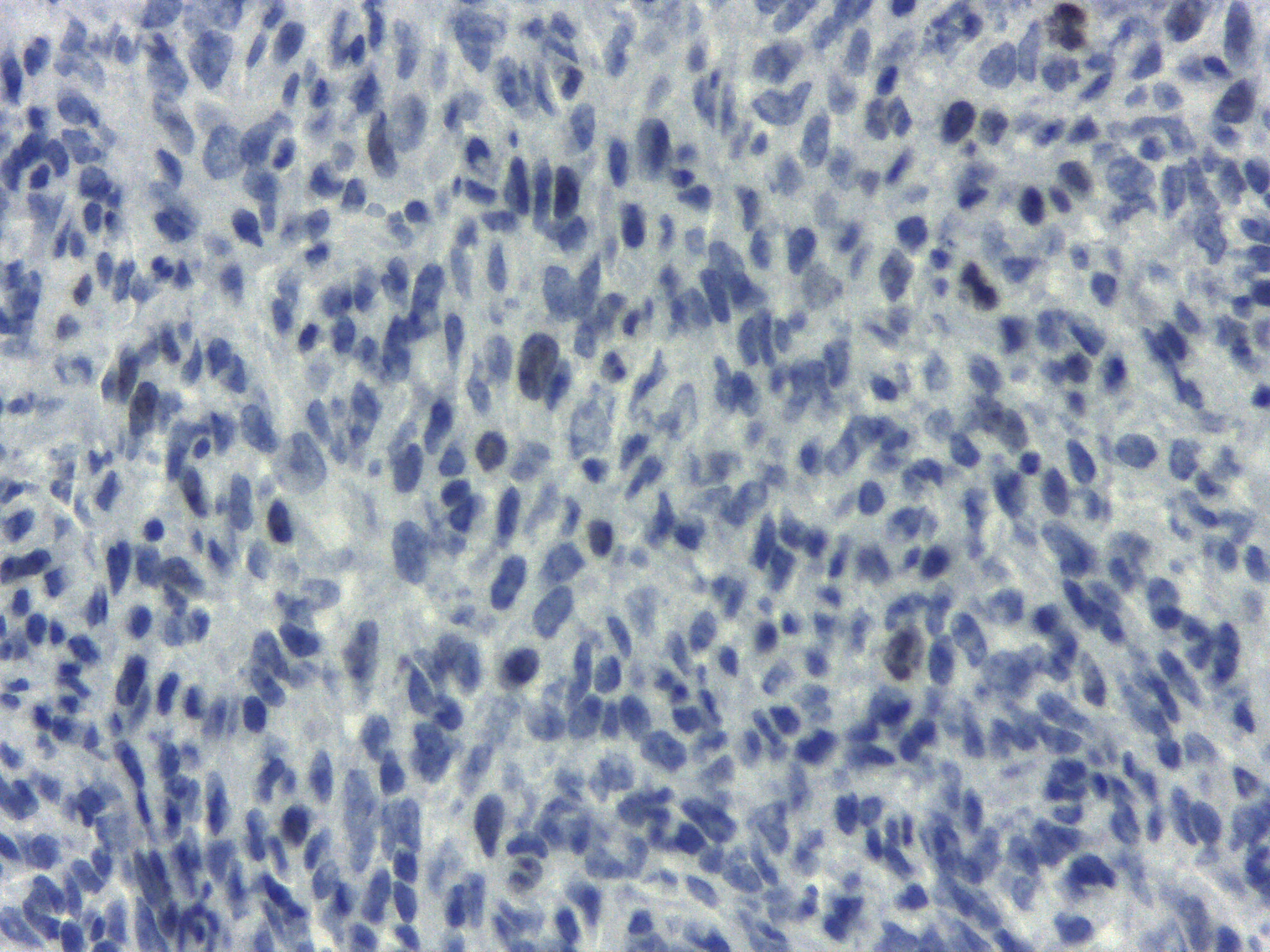

Supplement: S3 Fig — (ZIP) [file pone.0188960.s016.zip › Ki-67 IHC image bac/Ki-67 bac4-1.jpg]

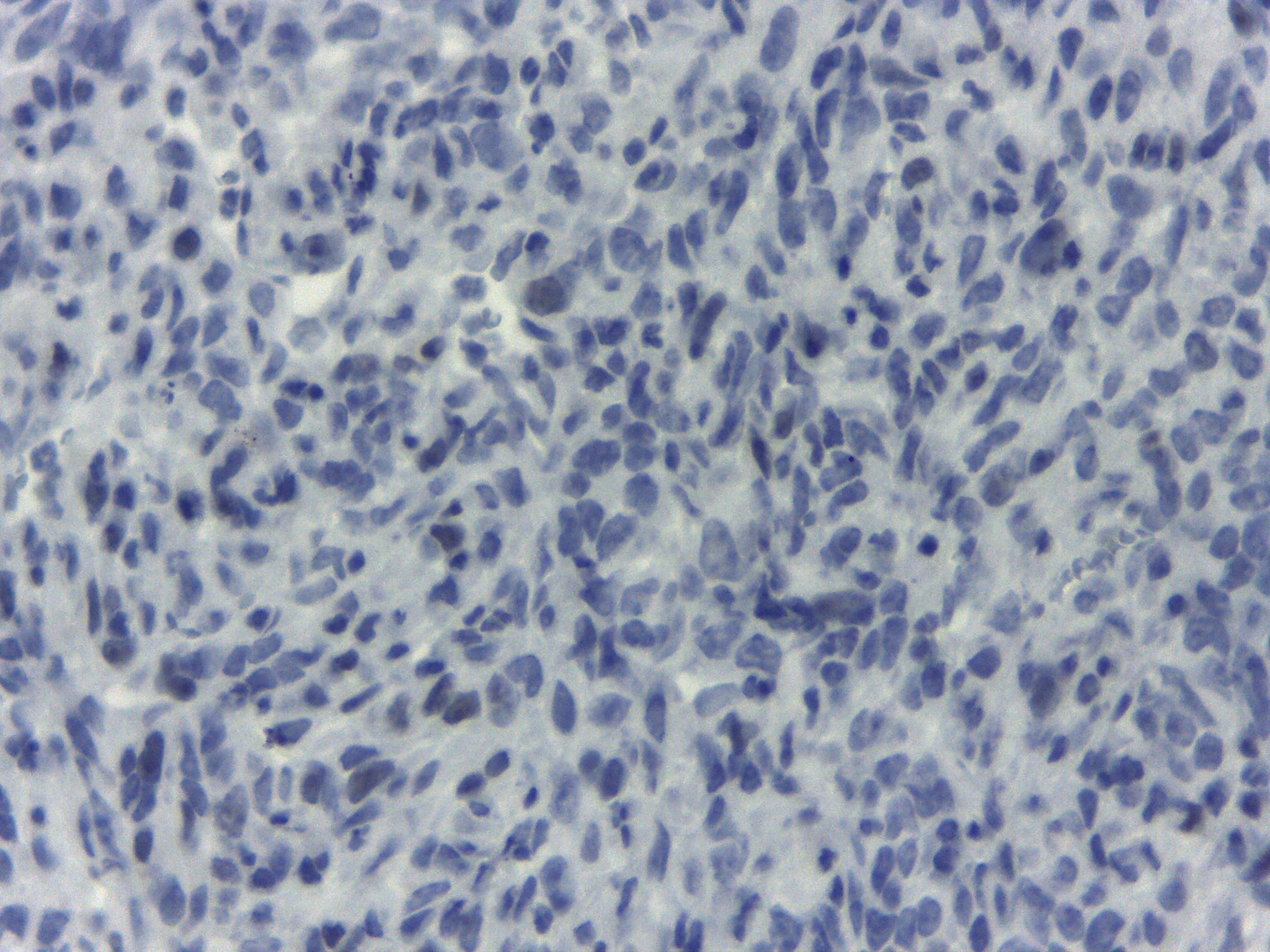

Supplement: S3 Fig — (ZIP) [file pone.0188960.s016.zip › Ki-67 IHC image bac/Ki-67 bac4-2.jpg]

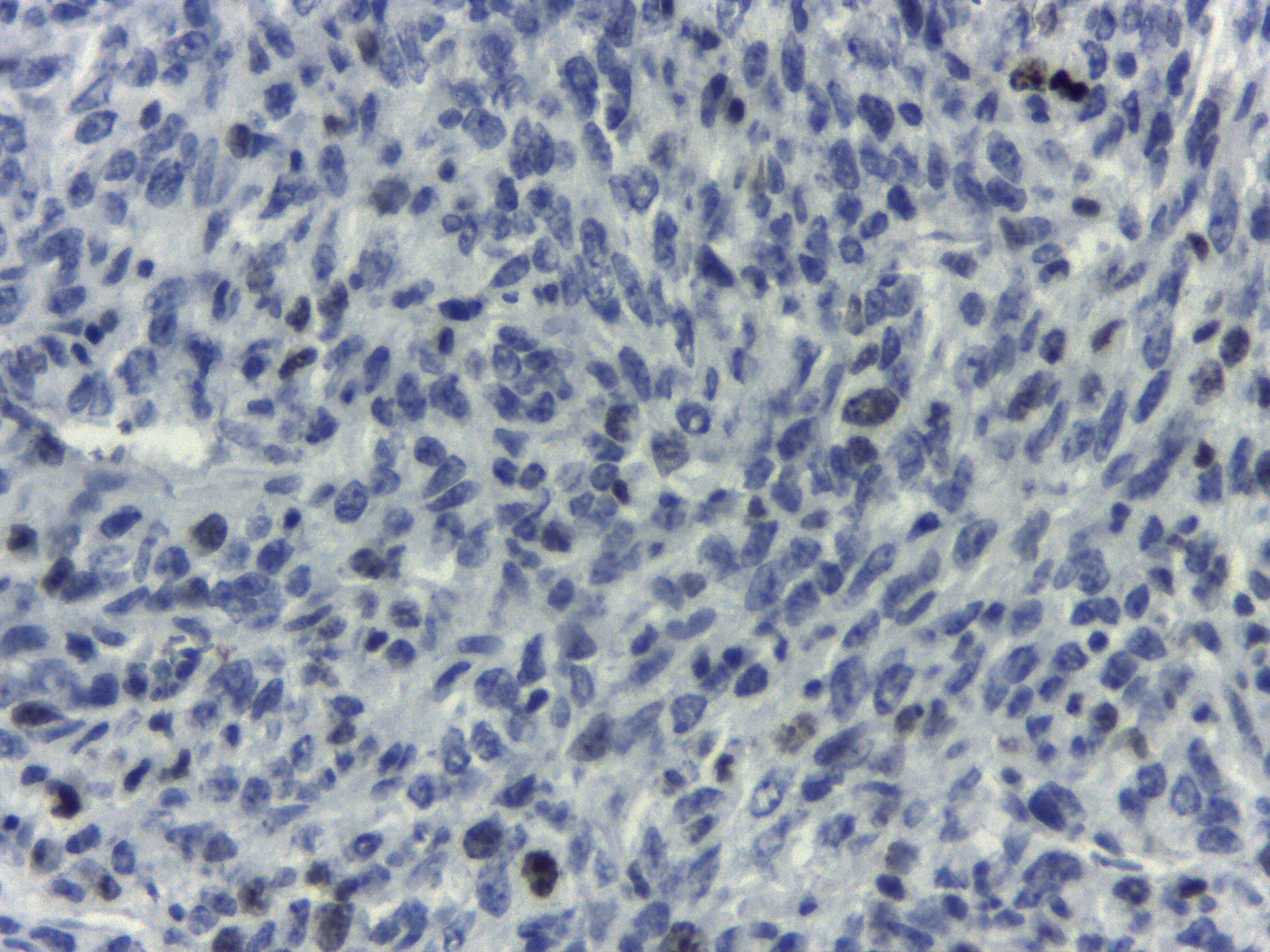

Supplement: S3 Fig — (ZIP) [file pone.0188960.s016.zip › Ki-67 IHC image bac/Ki-67 bac4-3.jpg]

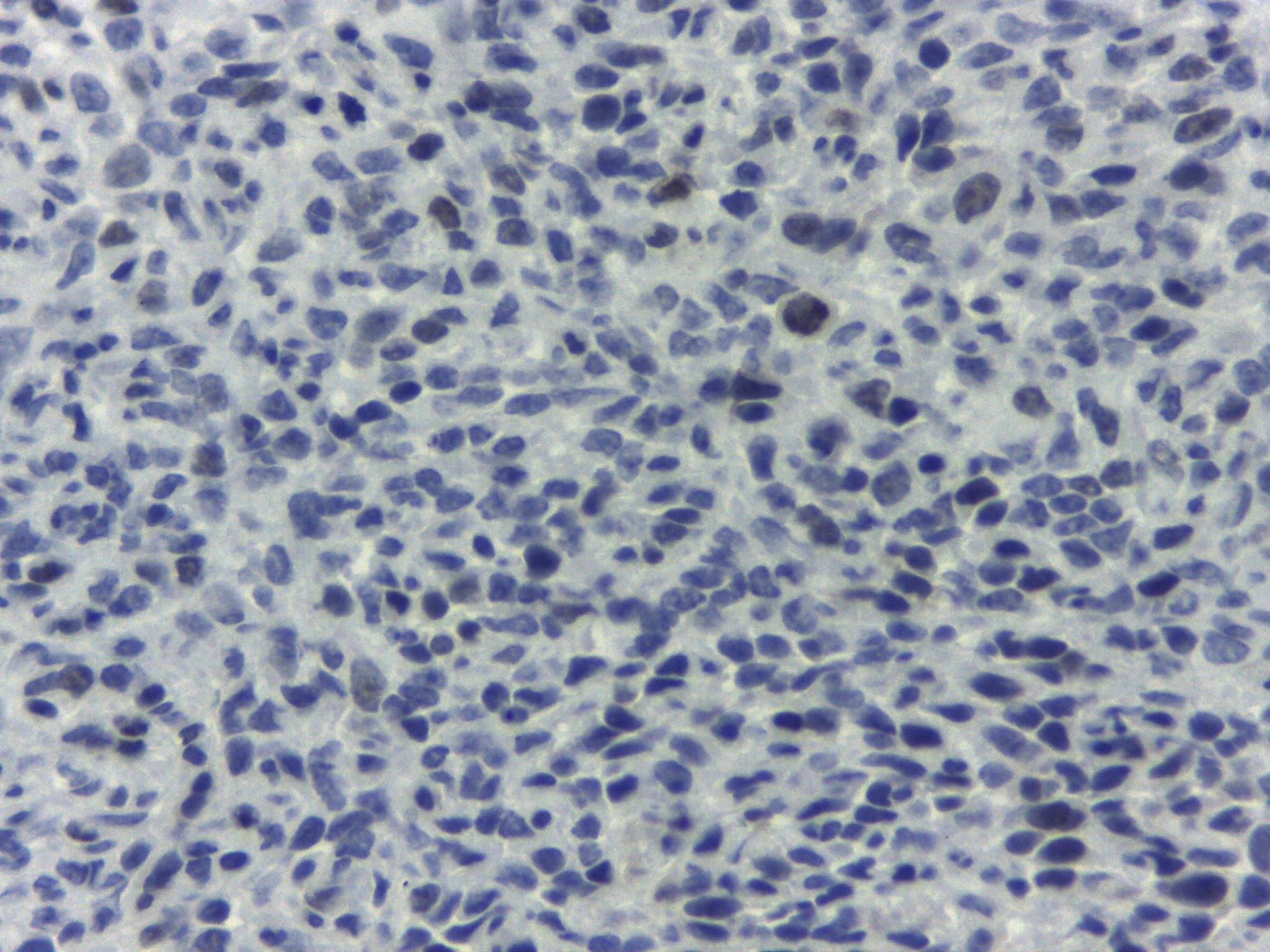

Supplement: S3 Fig — (ZIP) [file pone.0188960.s016.zip › Ki-67 IHC image bac/Ki-67 bac4-4.jpg]

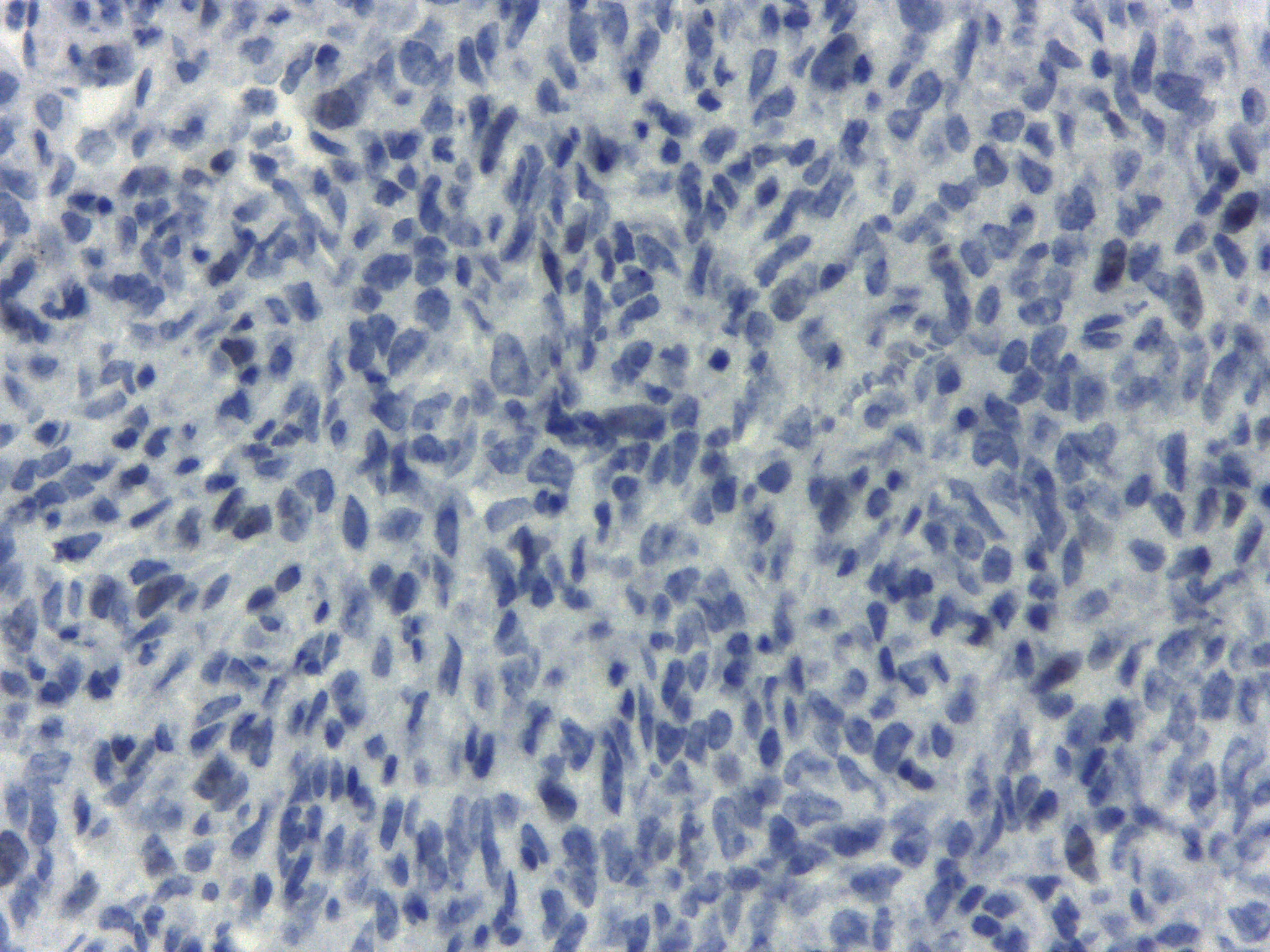

Supplement: S3 Fig — (ZIP) [file pone.0188960.s016.zip › Ki-67 IHC image bac/Ki-67 bac4-5.jpg]

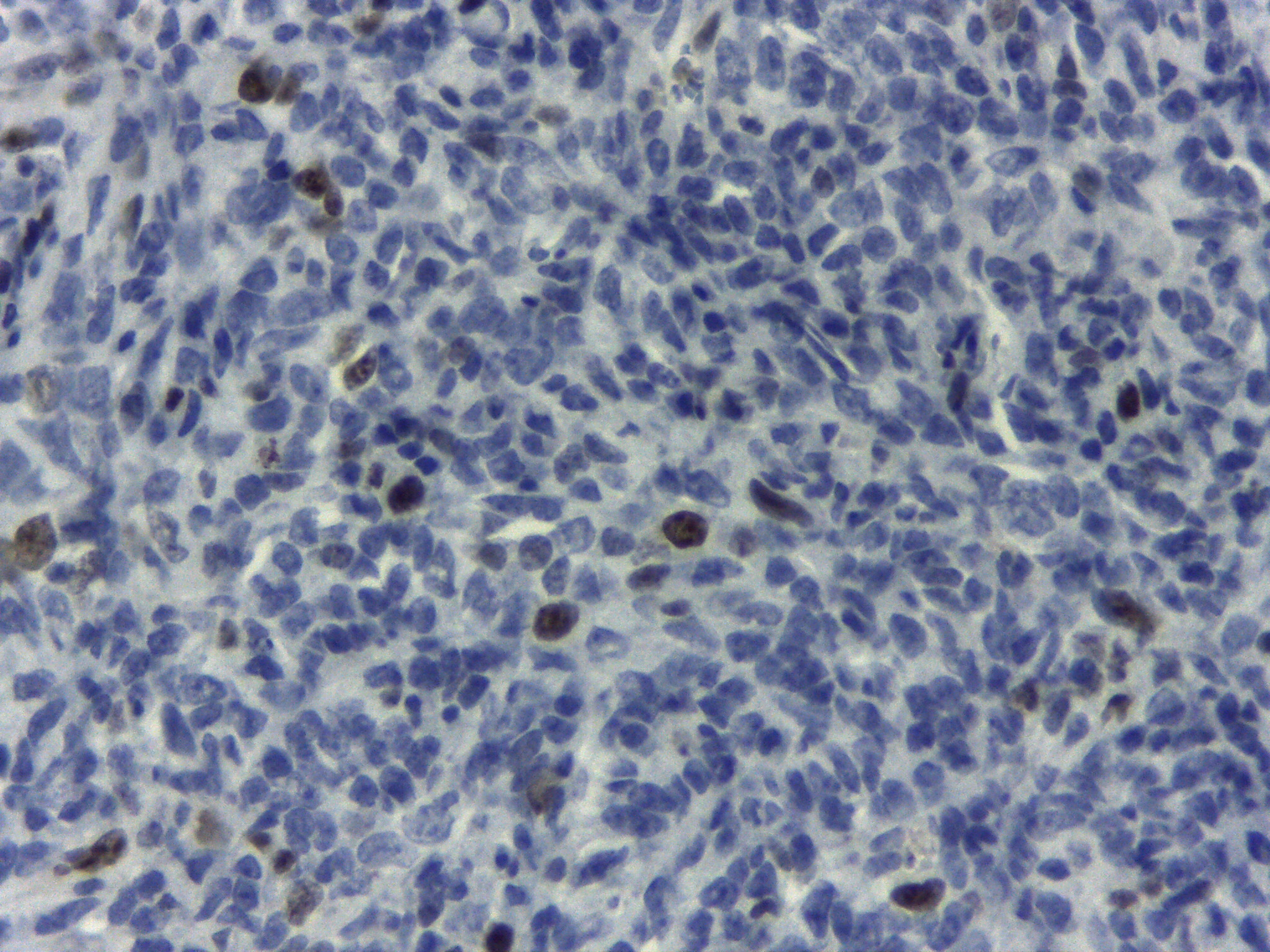

Supplement: S3 Fig — (ZIP) [file pone.0188960.s016.zip › Ki-67 IHC image bac/Ki-67 bac5-1.jpg]

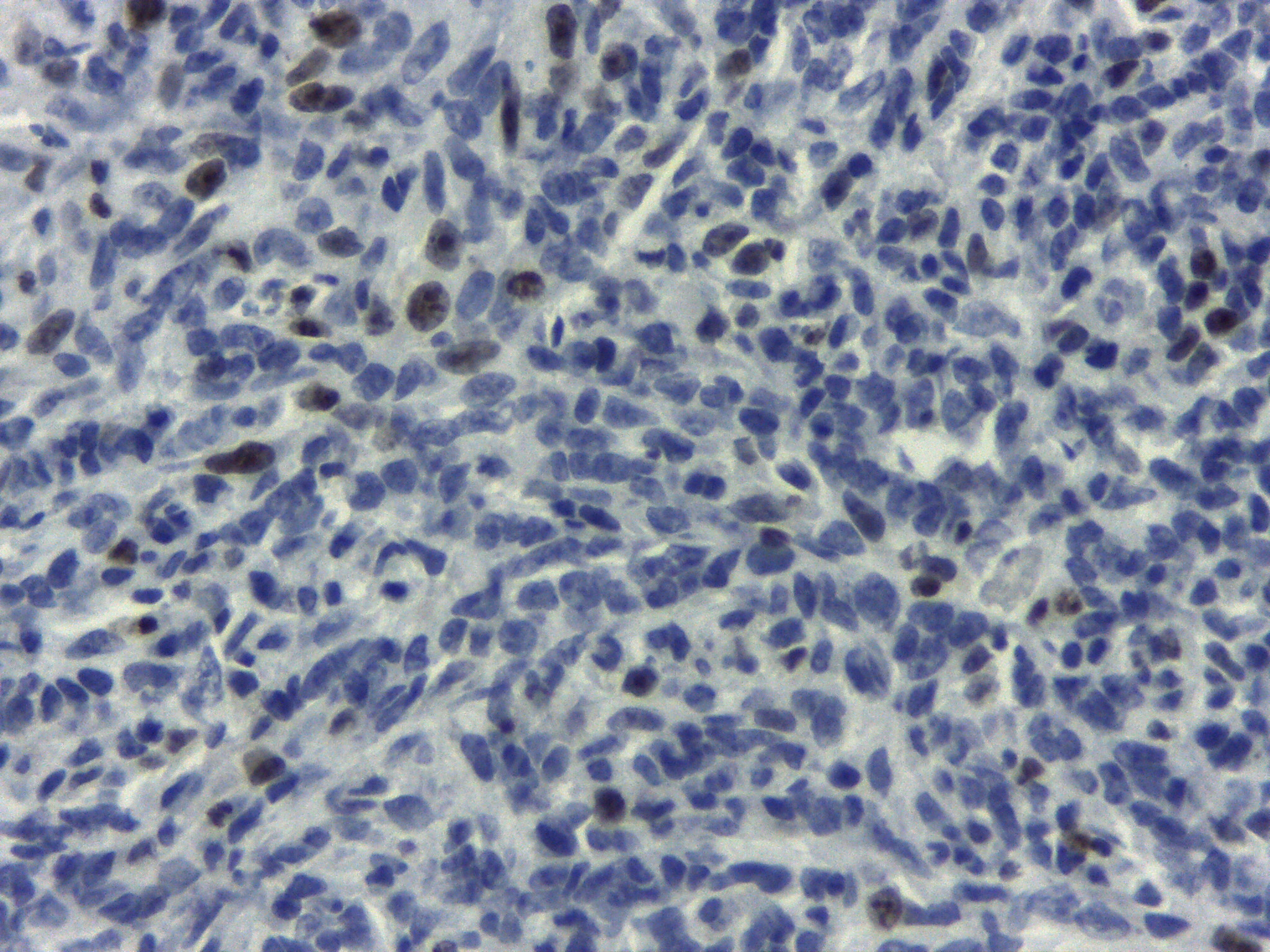

Supplement: S3 Fig — (ZIP) [file pone.0188960.s016.zip › Ki-67 IHC image bac/Ki-67 bac5-2.jpg]

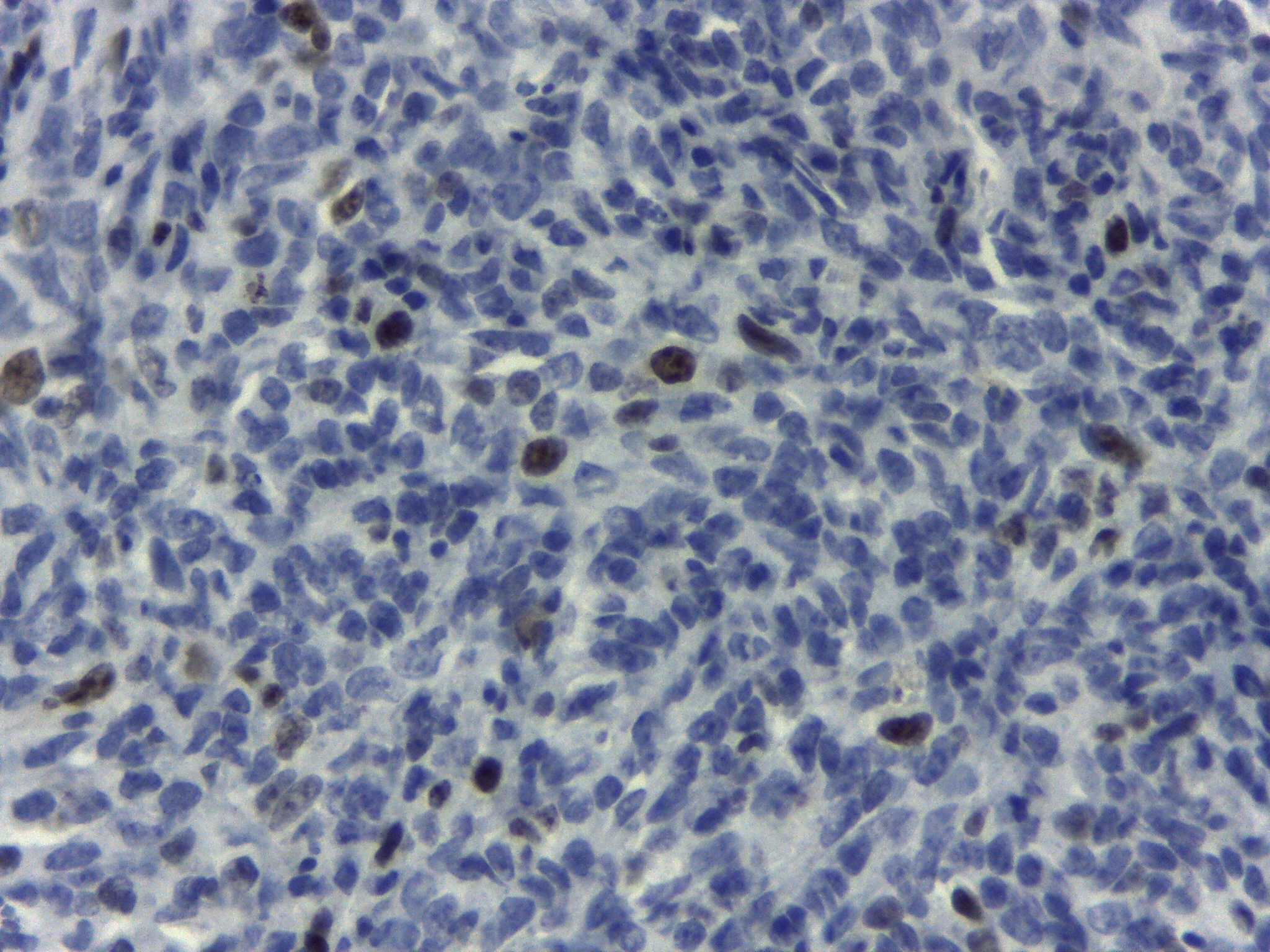

Supplement: S3 Fig — (ZIP) [file pone.0188960.s016.zip › Ki-67 IHC image bac/Ki-67 bac5-3.jpg]

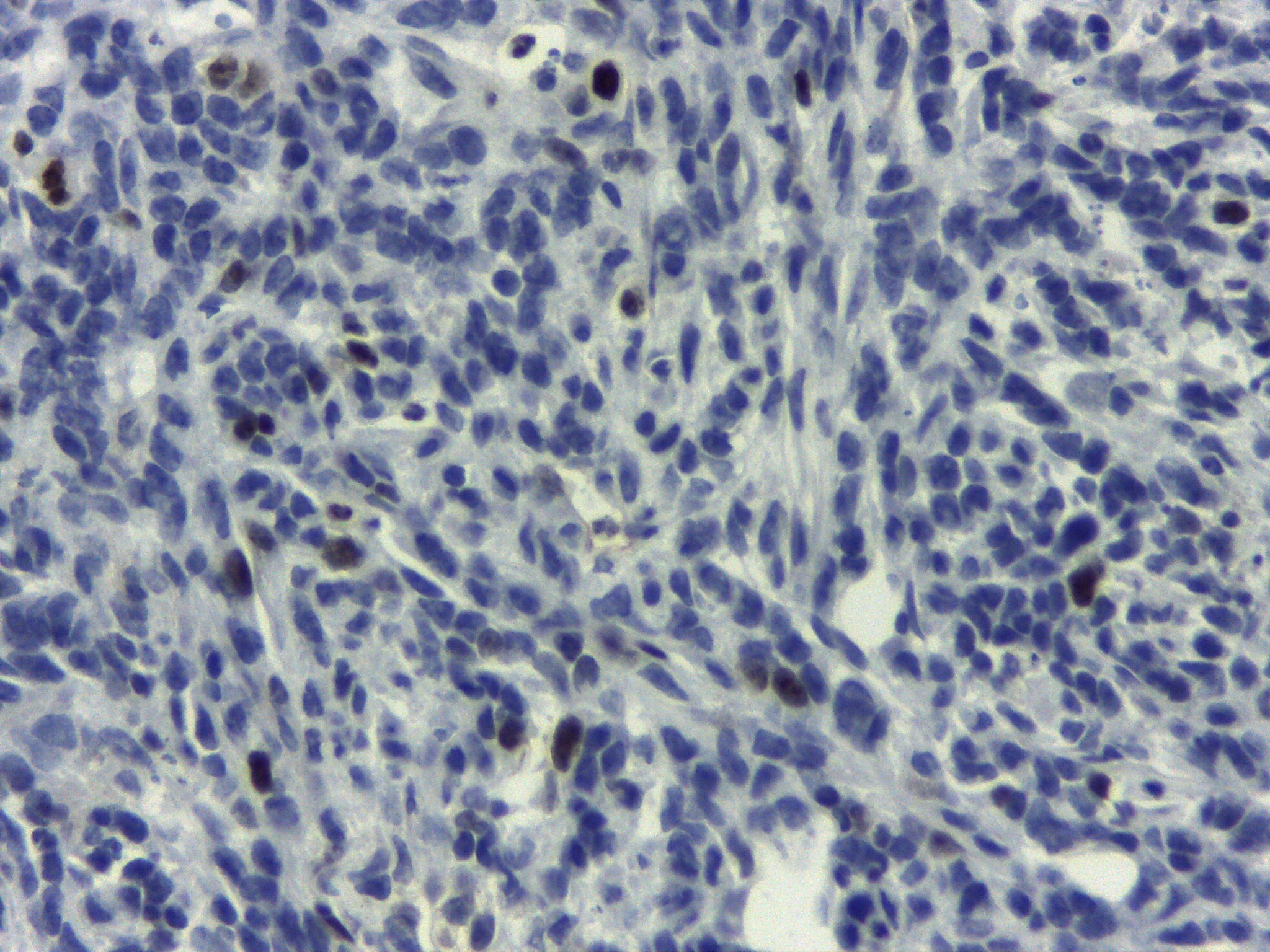

Supplement: S3 Fig — (ZIP) [file pone.0188960.s016.zip › Ki-67 IHC image bac/Ki-67 bac5-4.jpg]

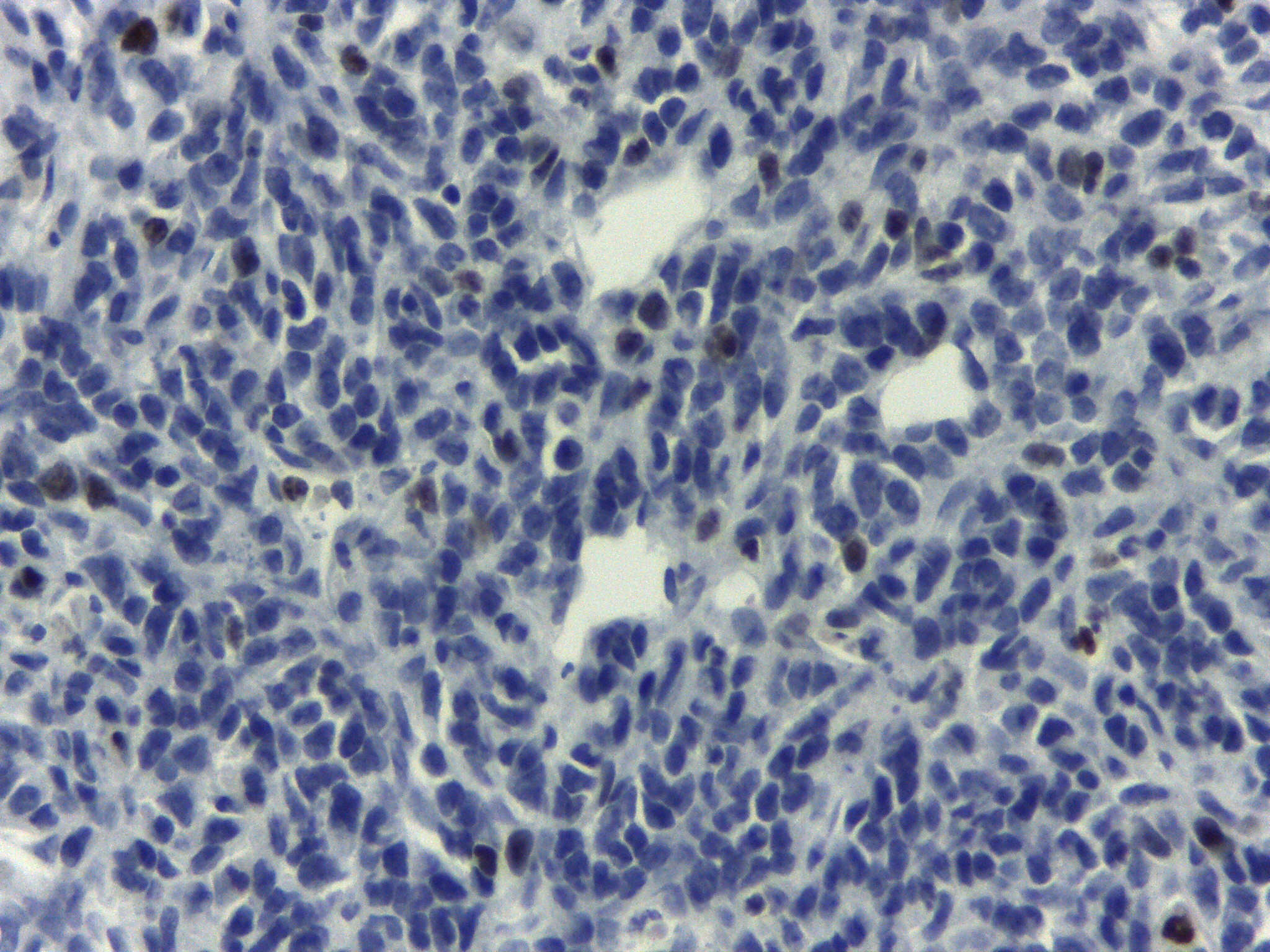

Supplement: S3 Fig — (ZIP) [file pone.0188960.s016.zip › Ki-67 IHC image bac/Ki-67 bac5-5.jpg]

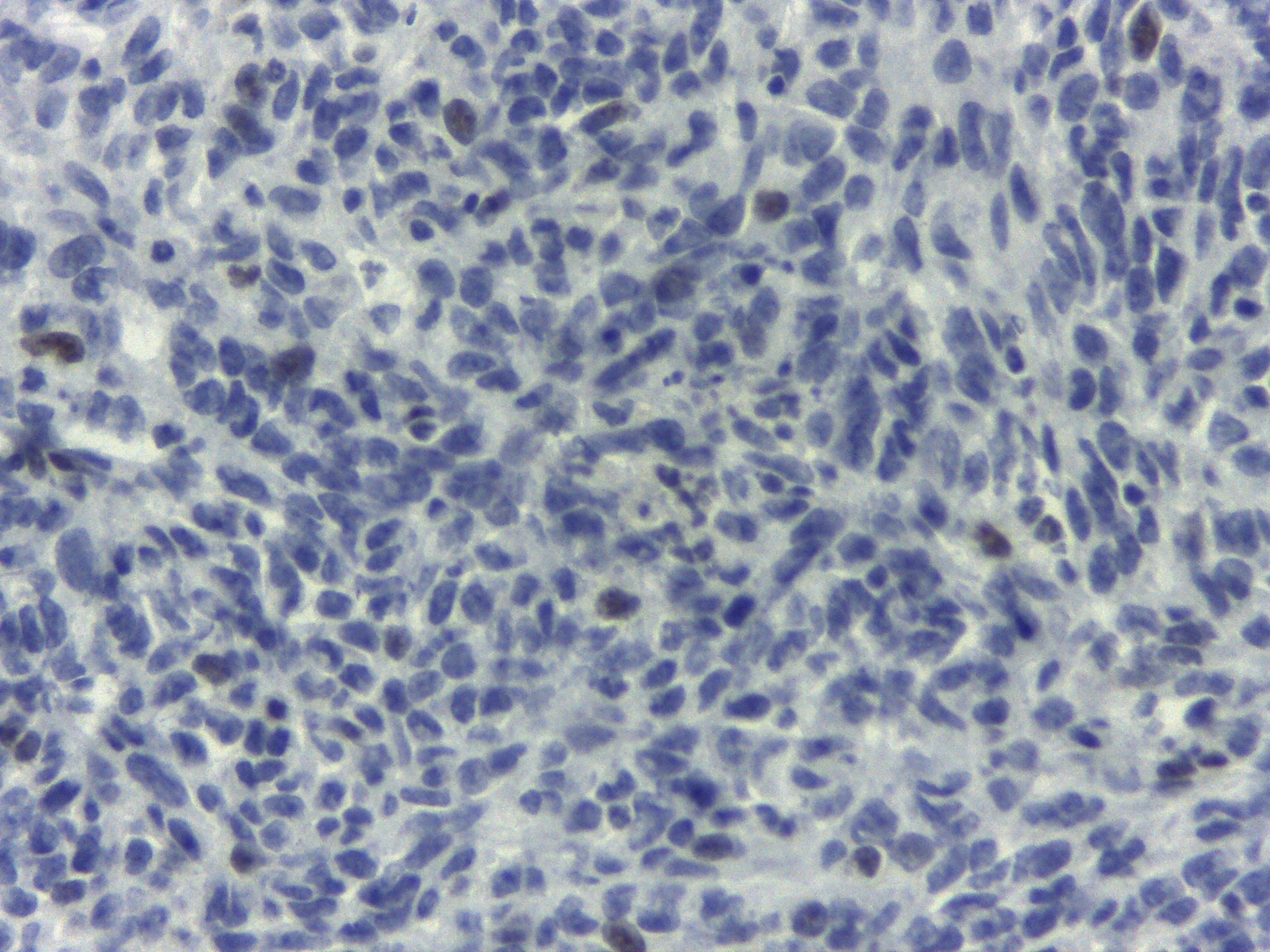

Supplement: S3 Fig — (ZIP) [file pone.0188960.s016.zip › Ki-67 IHC image bac/Ki-67 bac6-1.jpg]

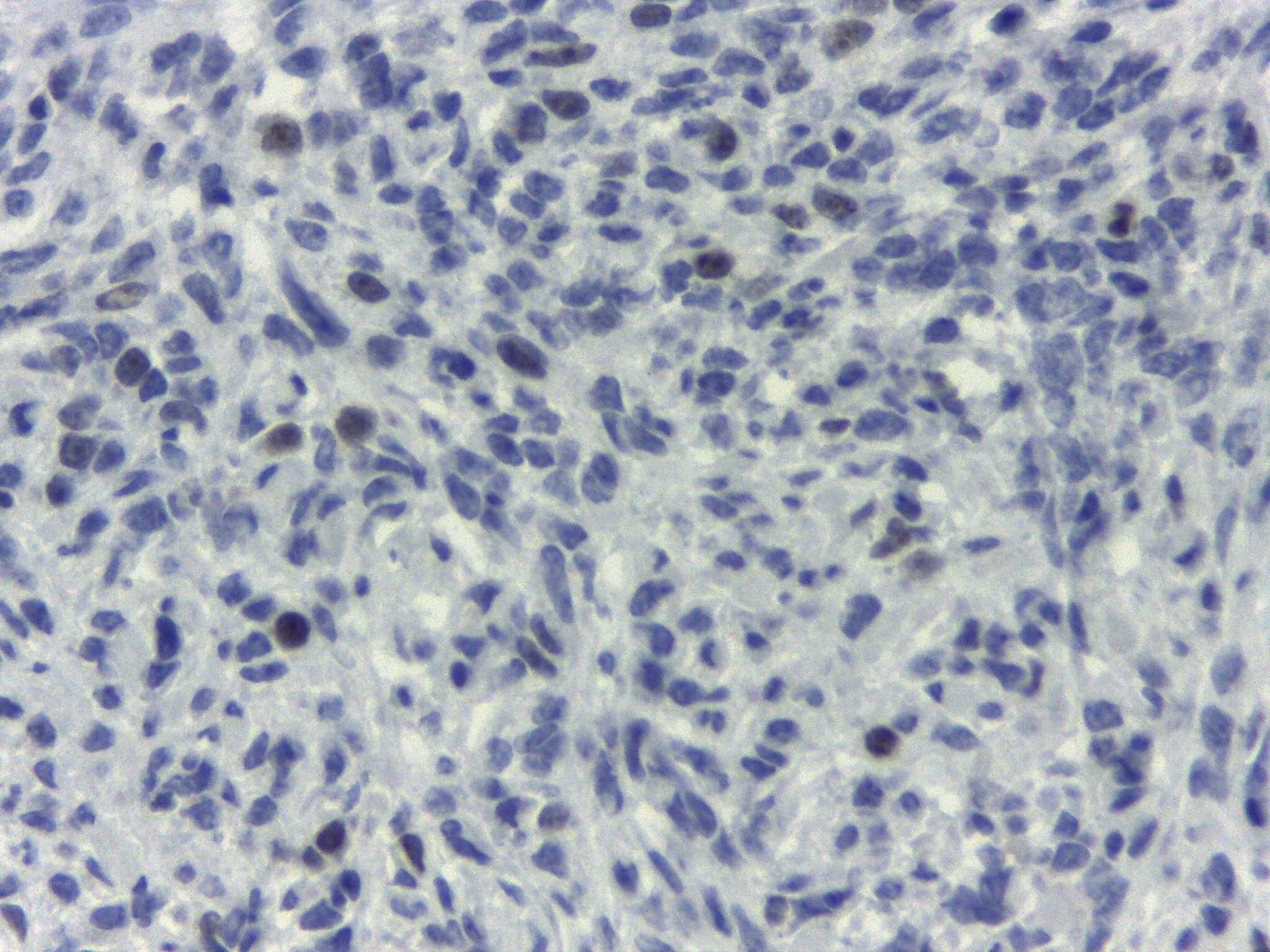

Supplement: S3 Fig — (ZIP) [file pone.0188960.s016.zip › Ki-67 IHC image bac/Ki-67 bac6-2.jpg]

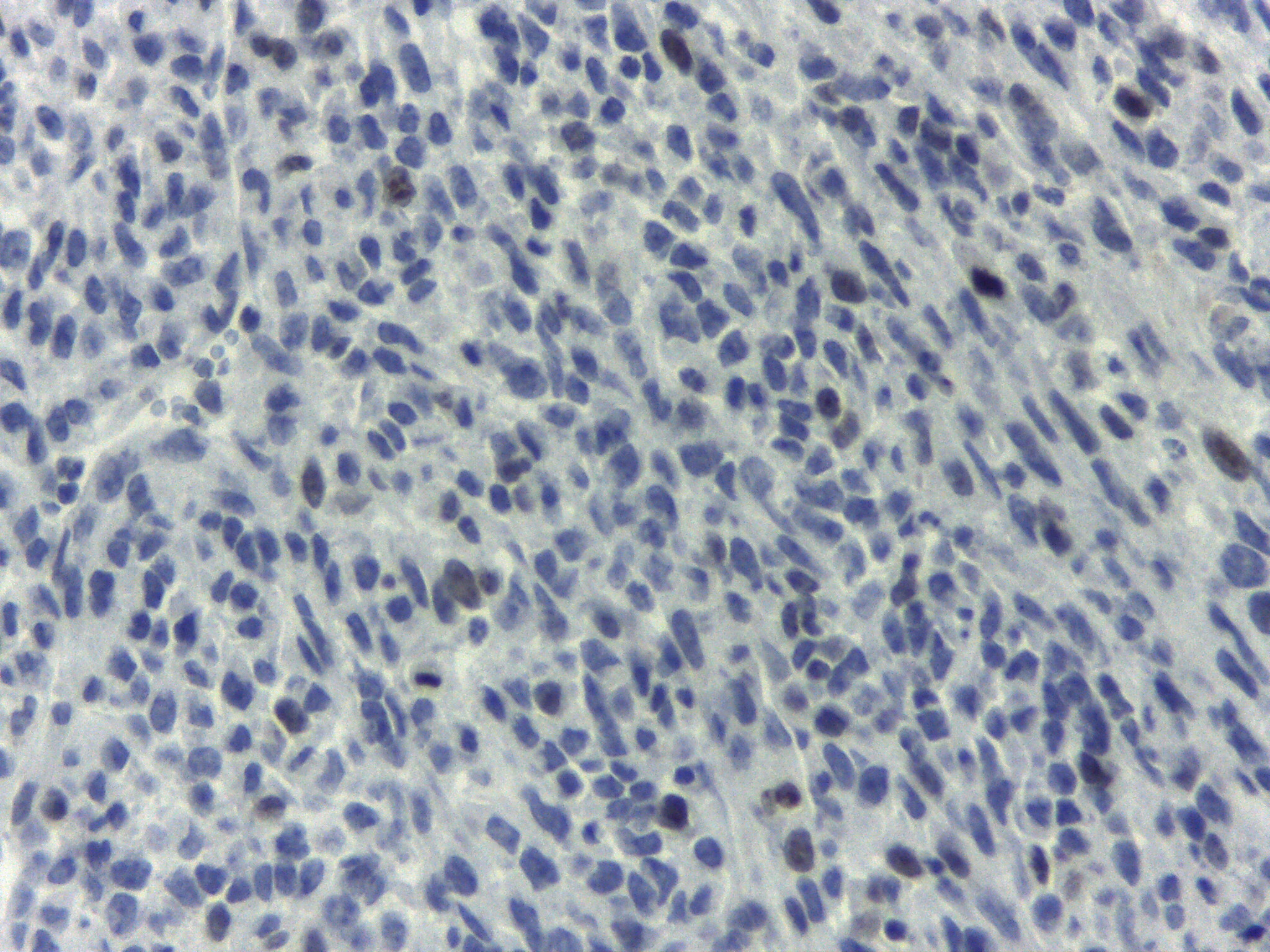

Supplement: S3 Fig — (ZIP) [file pone.0188960.s016.zip › Ki-67 IHC image bac/Ki-67 bac6-3.jpg]

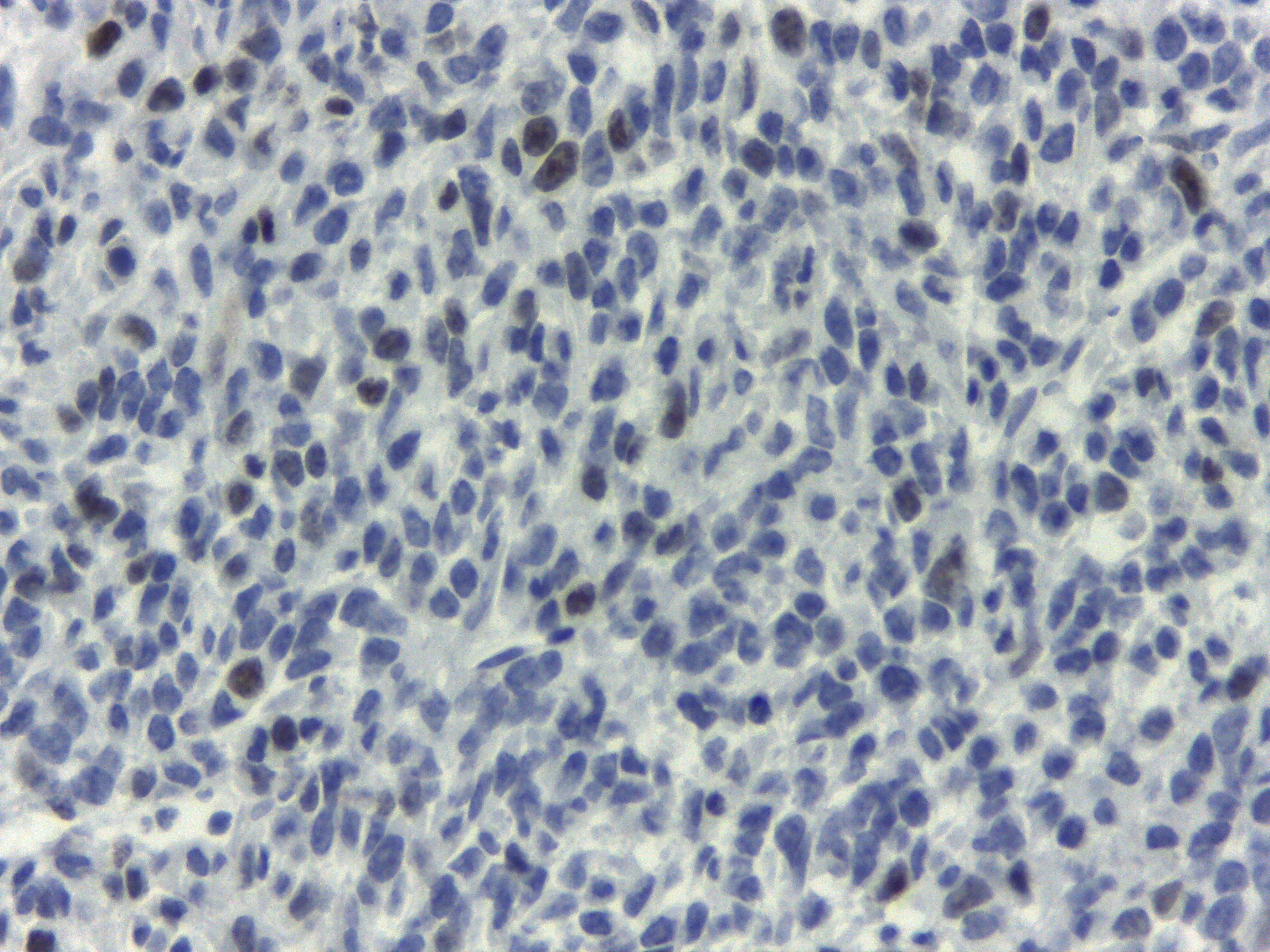

Supplement: S3 Fig — (ZIP) [file pone.0188960.s016.zip › Ki-67 IHC image bac/Ki-67 bac6-4.jpg]

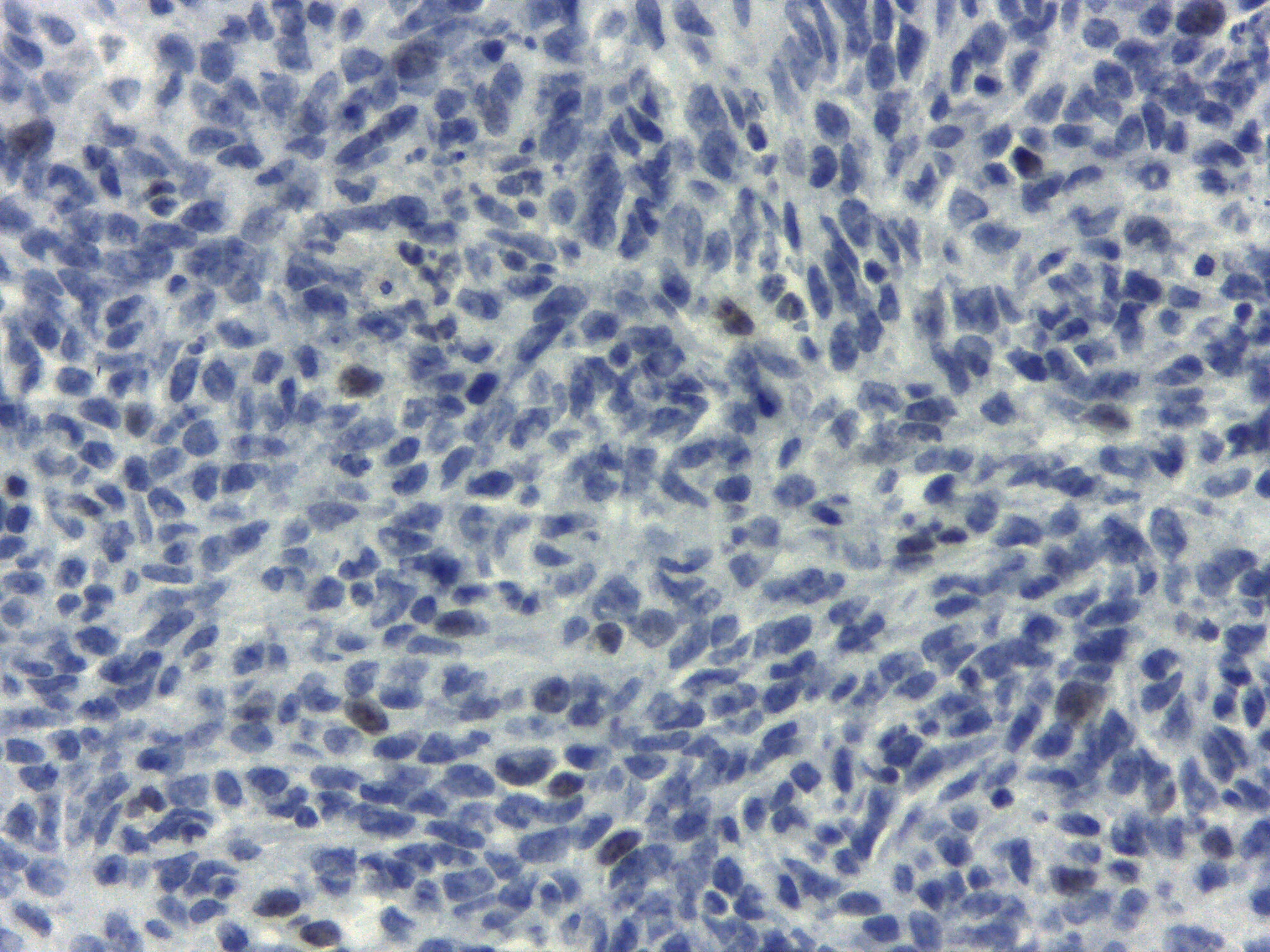

Supplement: S3 Fig — (ZIP) [file pone.0188960.s016.zip › Ki-67 IHC image bac/Ki-67 bac6-5.jpg]

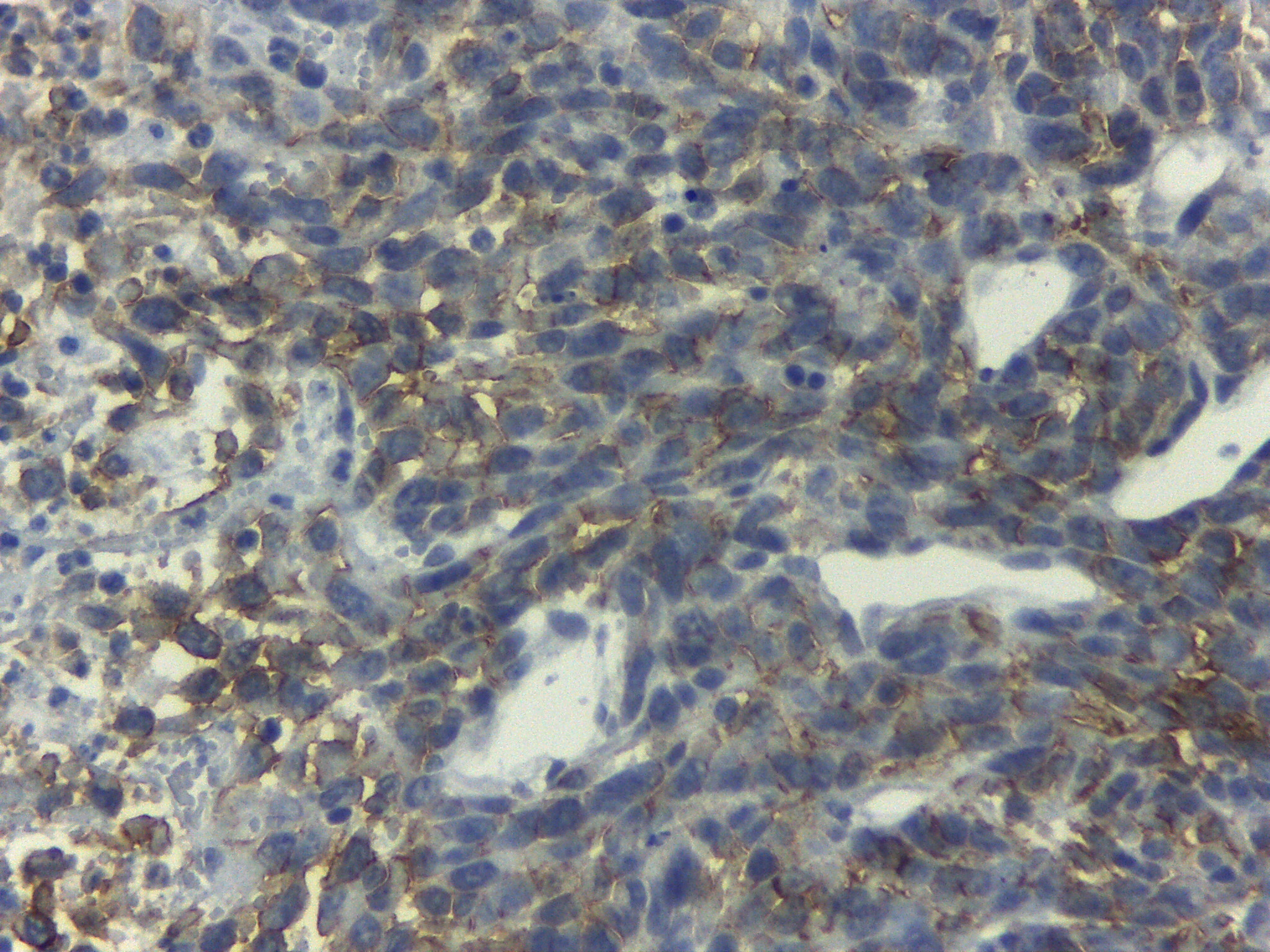

Supplement: S4 Fig — (ZIP) [file pone.0188960.s017.zip › Ca IX IHC image CON/Ca IX con1-1.jpg]

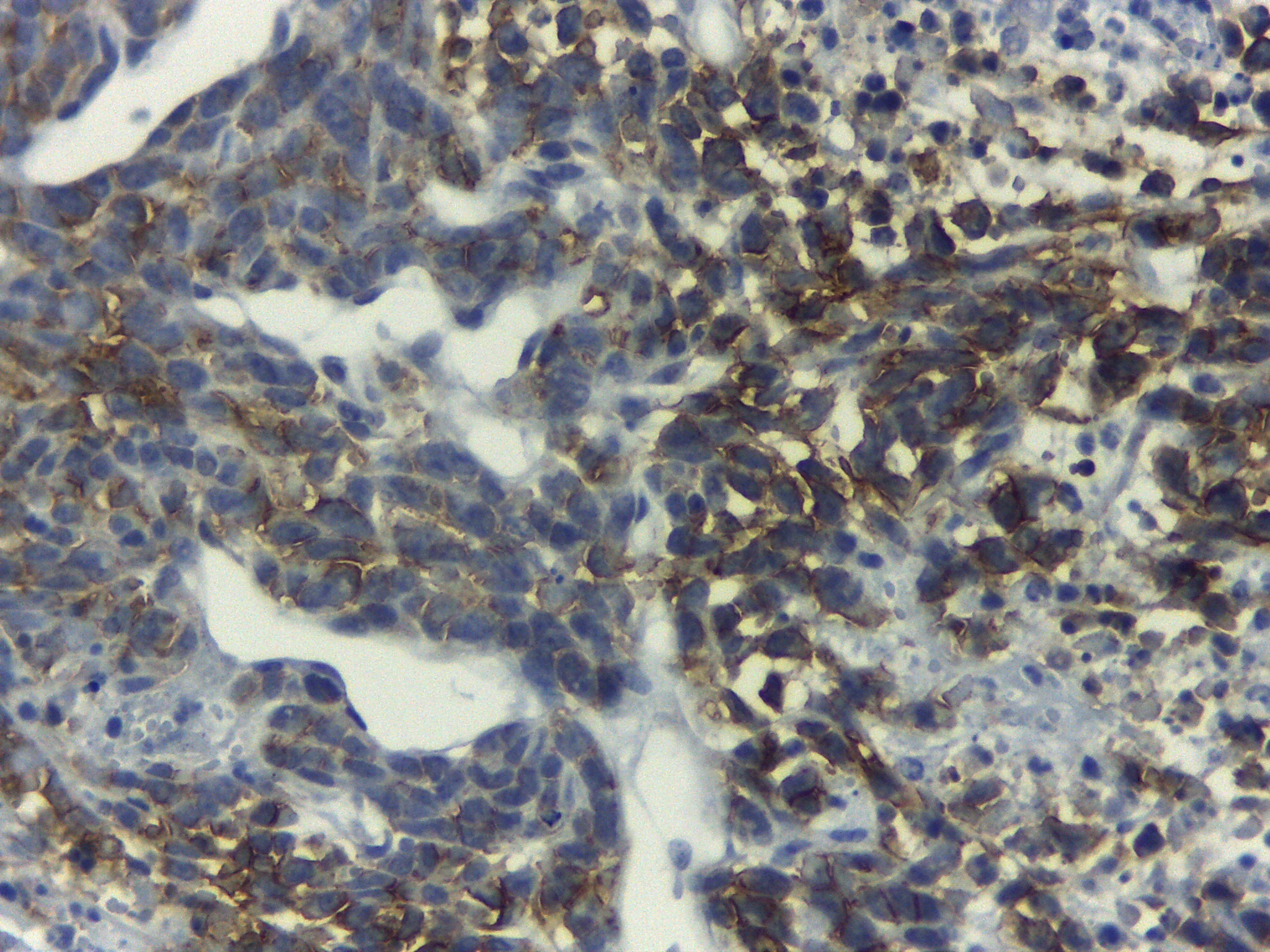

Supplement: S4 Fig — (ZIP) [file pone.0188960.s017.zip › Ca IX IHC image CON/Ca IX con1-2.jpg]

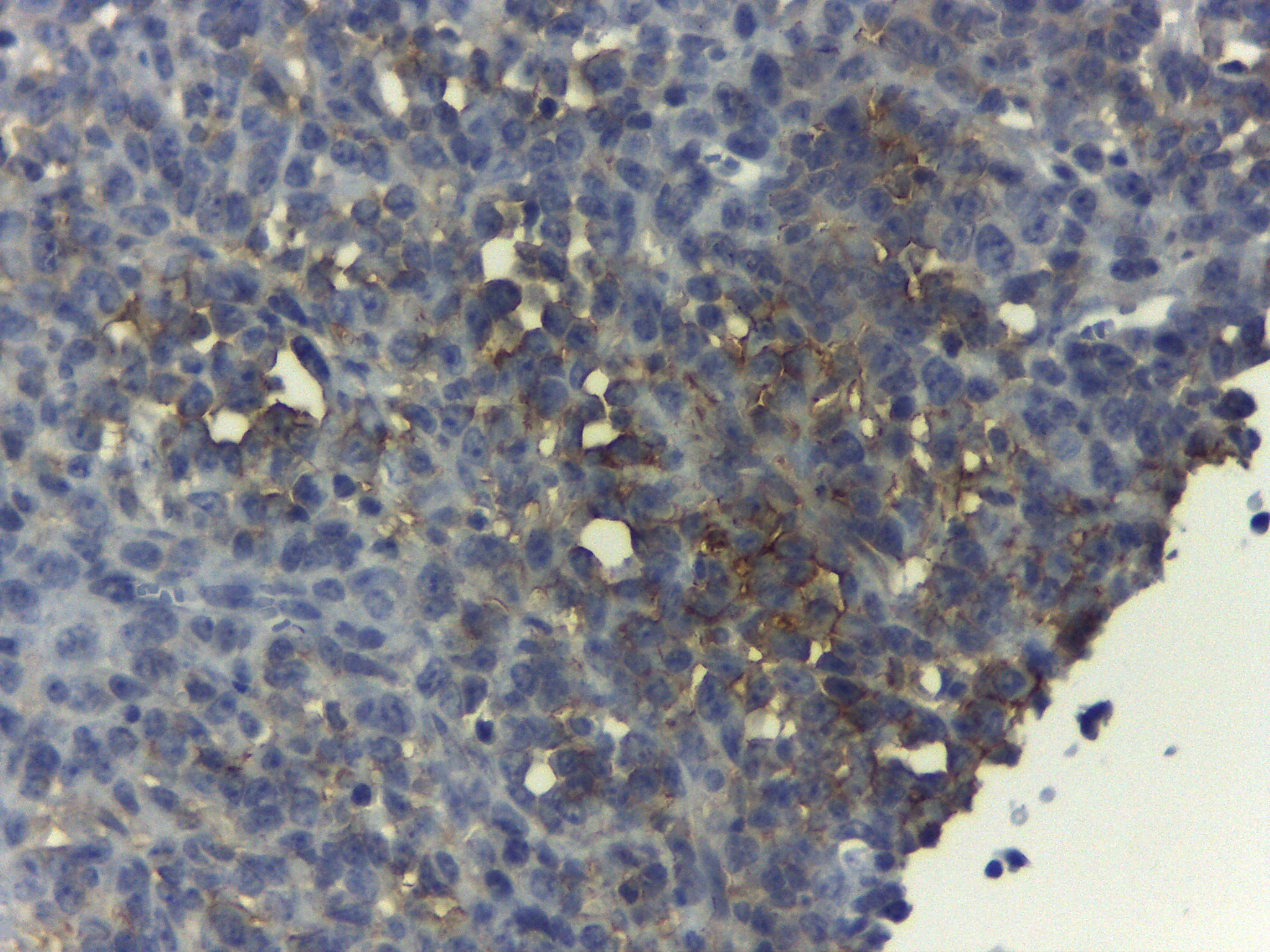

Supplement: S4 Fig — (ZIP) [file pone.0188960.s017.zip › Ca IX IHC image CON/Ca IX con1-3.jpg]

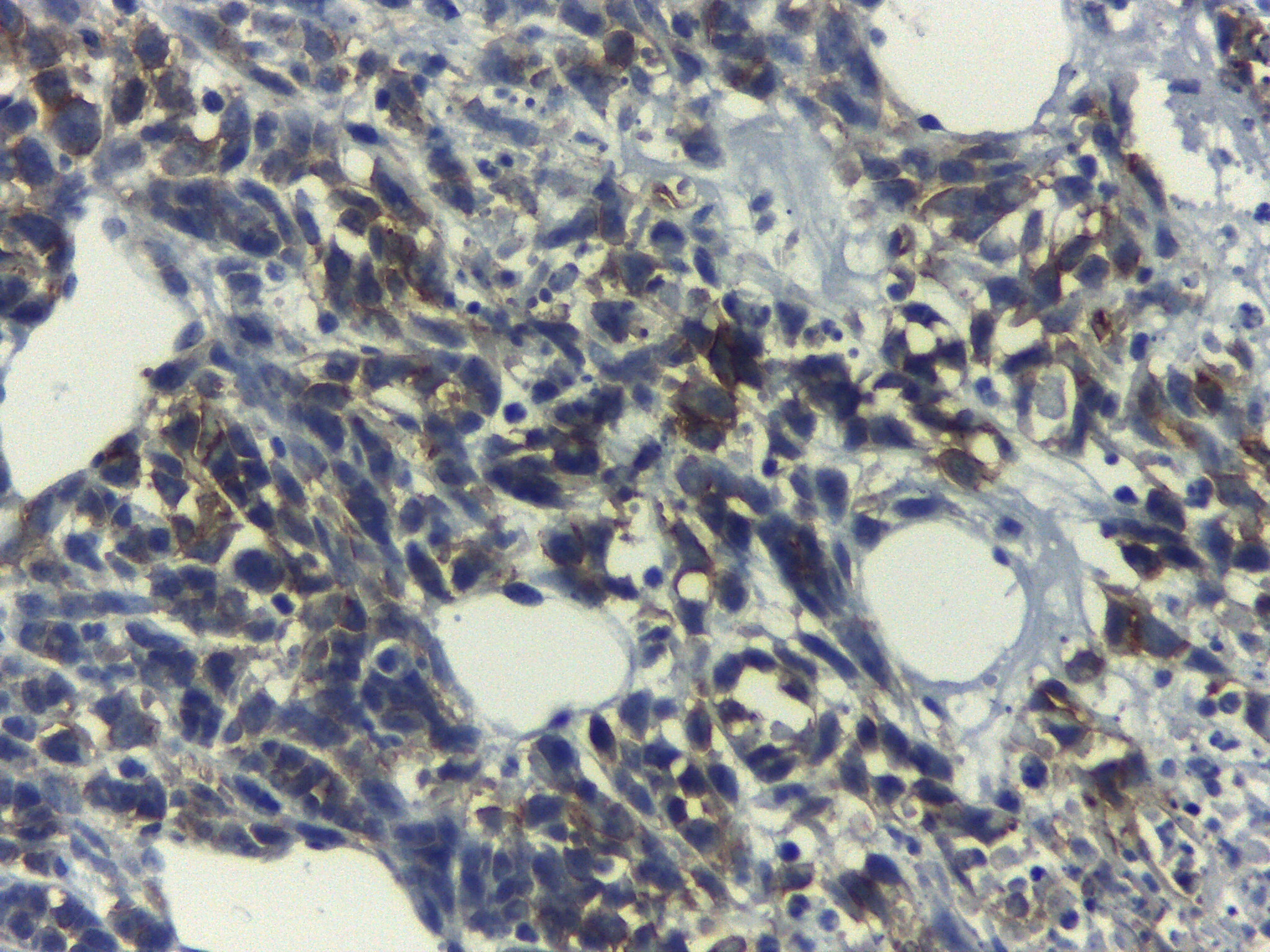

Supplement: S4 Fig — (ZIP) [file pone.0188960.s017.zip › Ca IX IHC image CON/Ca IX con1-4.jpg]

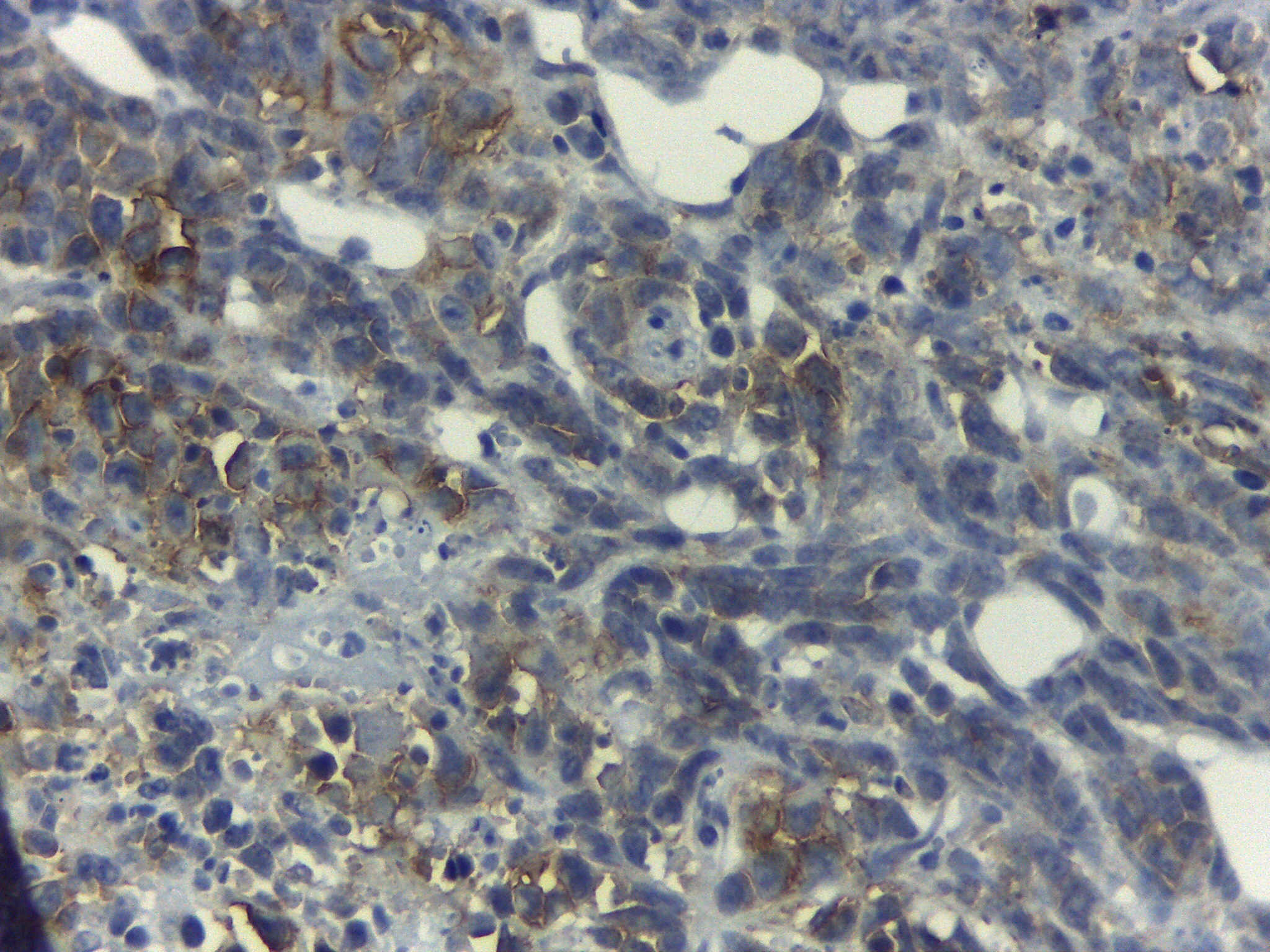

Supplement: S4 Fig — (ZIP) [file pone.0188960.s017.zip › Ca IX IHC image CON/Ca IX con1-5.jpg]

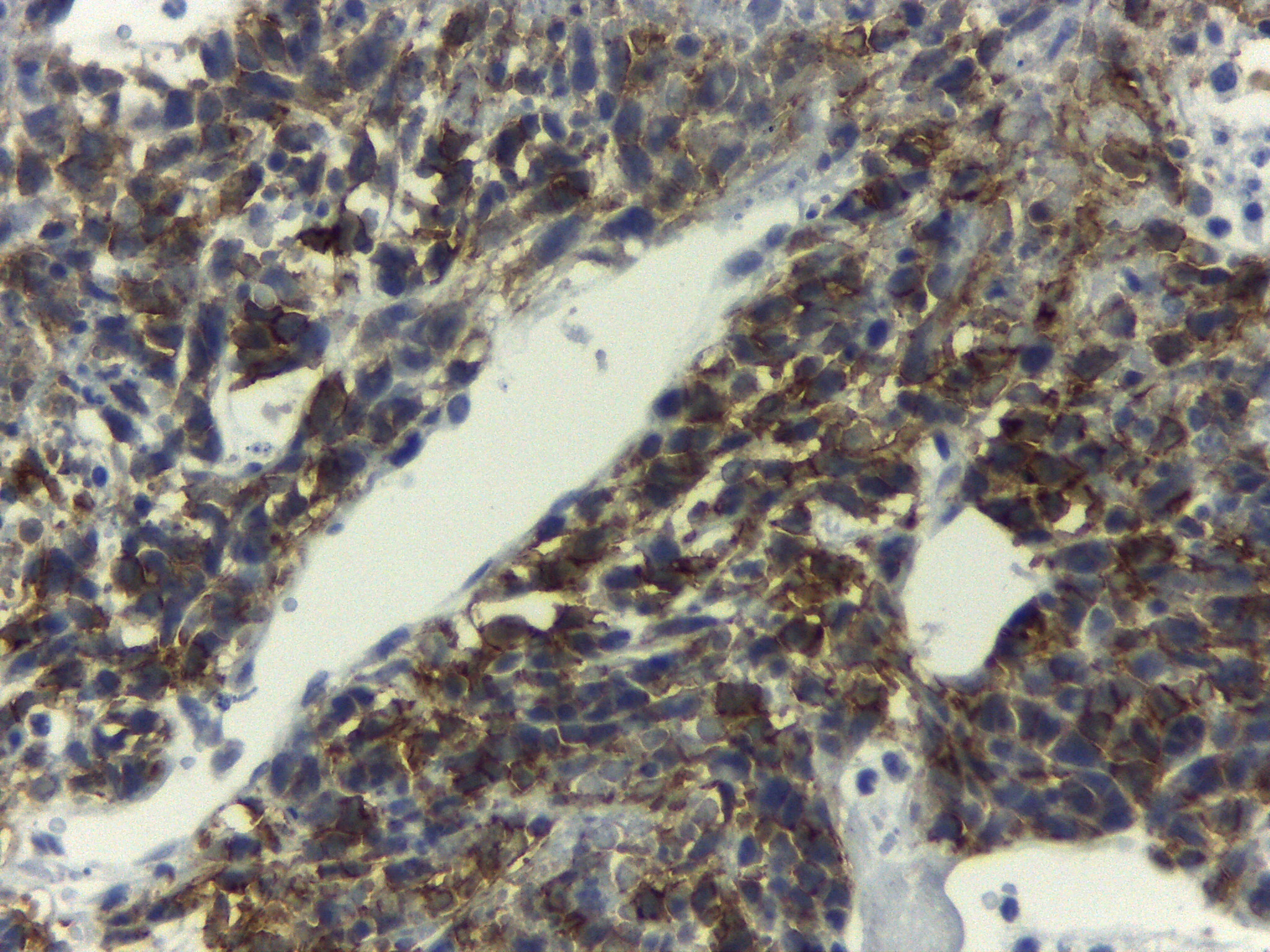

Supplement: S4 Fig — (ZIP) [file pone.0188960.s017.zip › Ca IX IHC image CON/Ca IX con2-1.jpg]

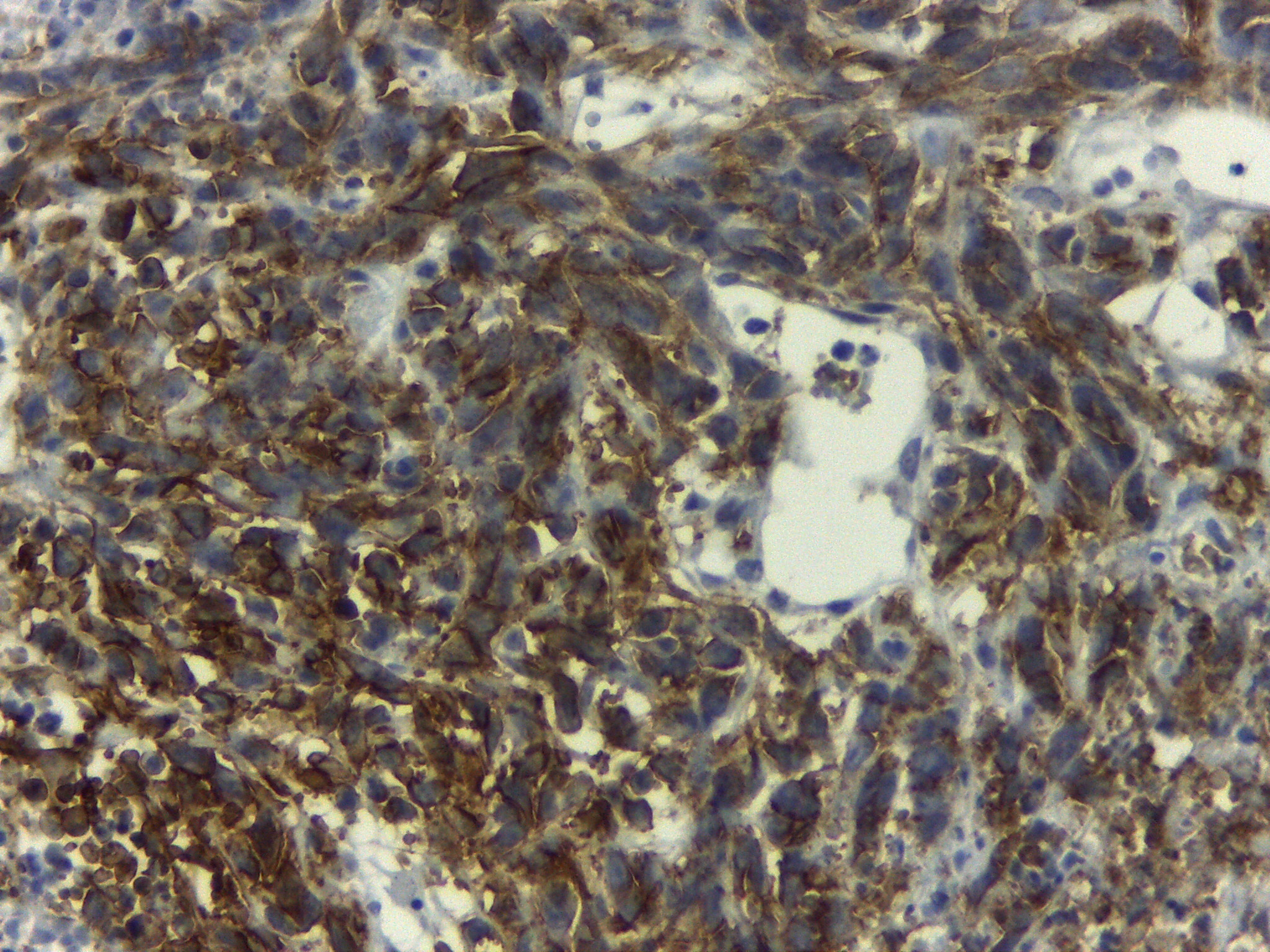

Supplement: S4 Fig — (ZIP) [file pone.0188960.s017.zip › Ca IX IHC image CON/Ca IX con2-2.jpg]

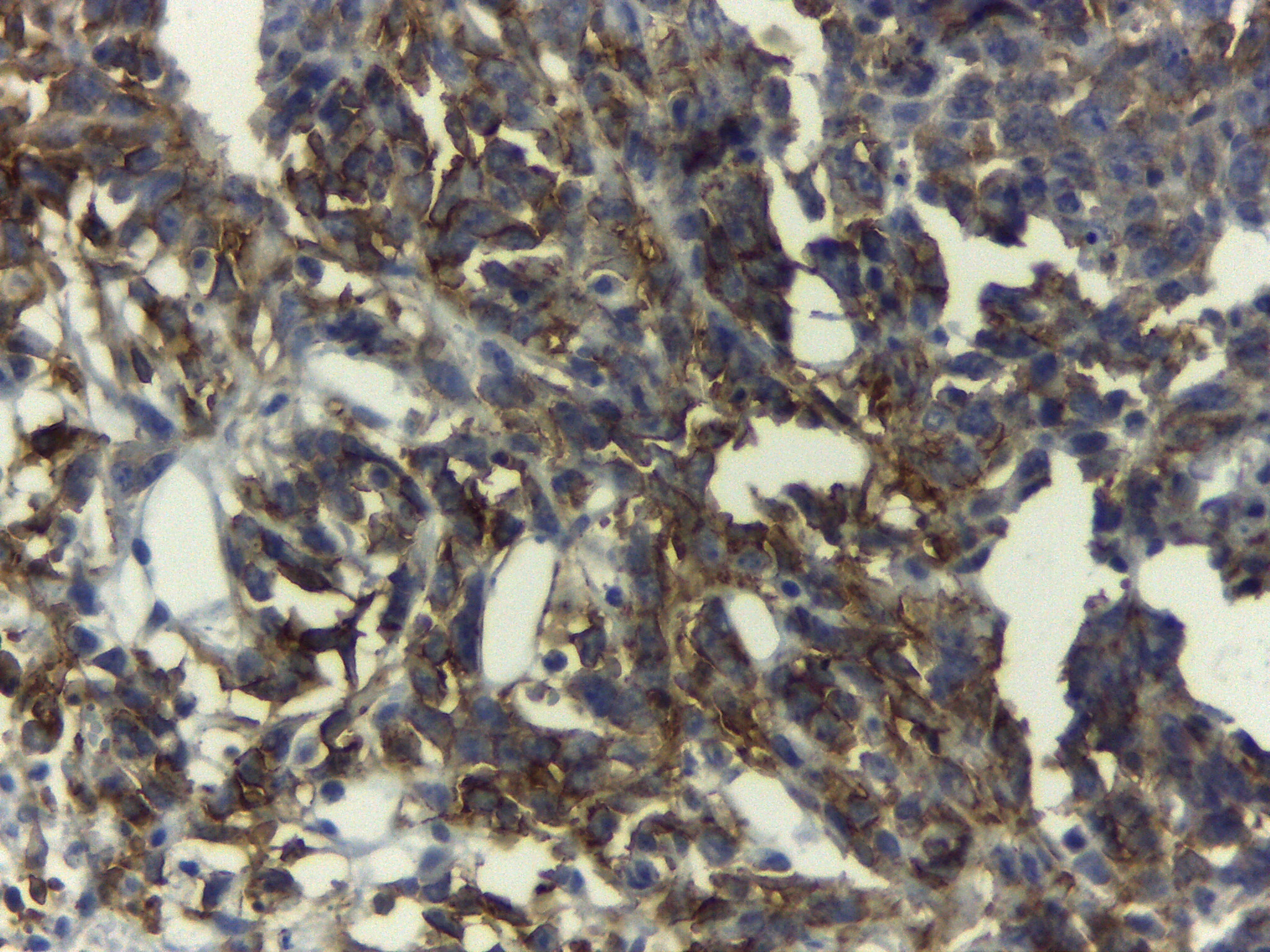

Supplement: S4 Fig — (ZIP) [file pone.0188960.s017.zip › Ca IX IHC image CON/Ca IX con2-3.jpg]

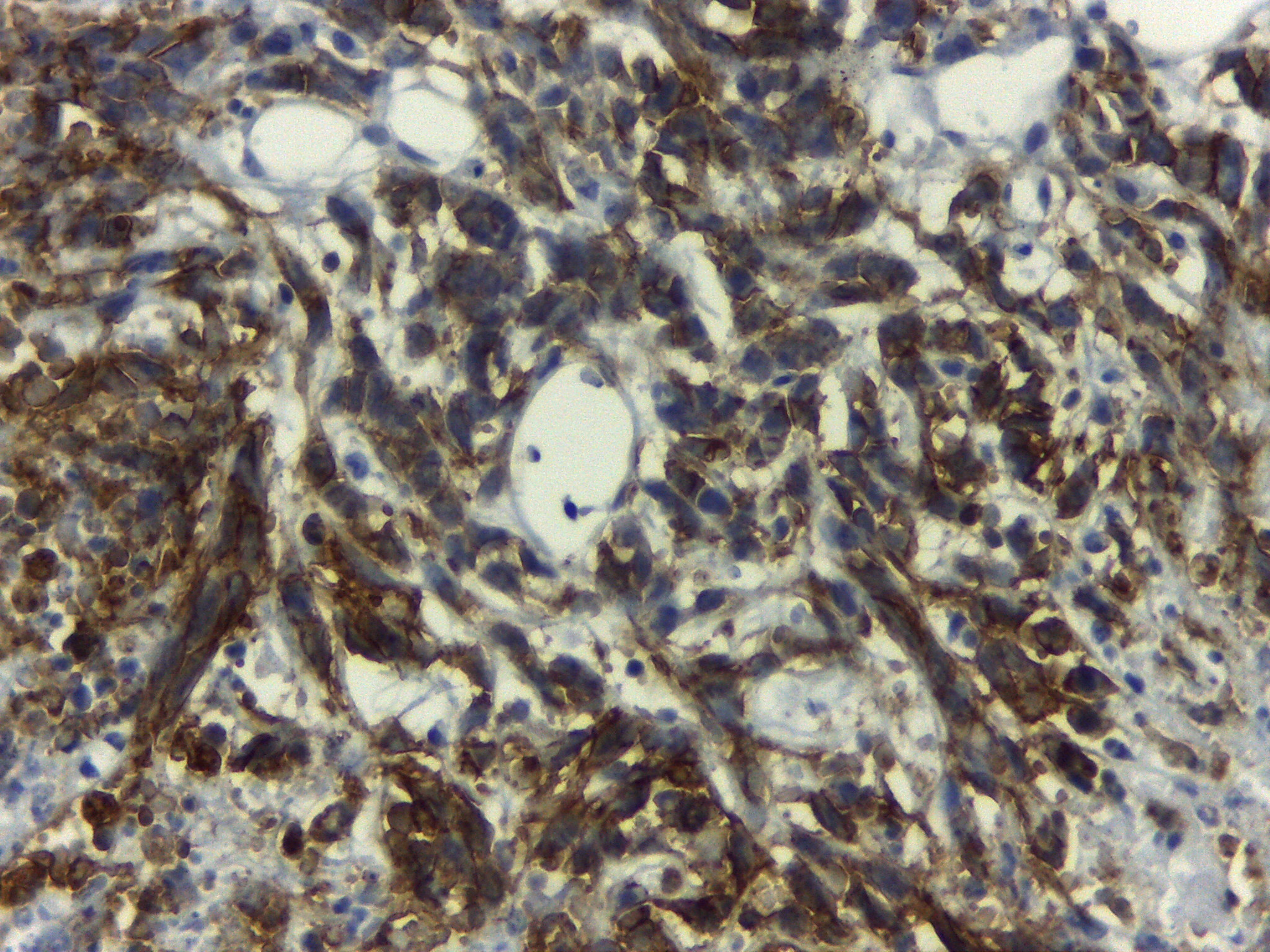

Supplement: S4 Fig — (ZIP) [file pone.0188960.s017.zip › Ca IX IHC image CON/Ca IX con2-4.jpg]

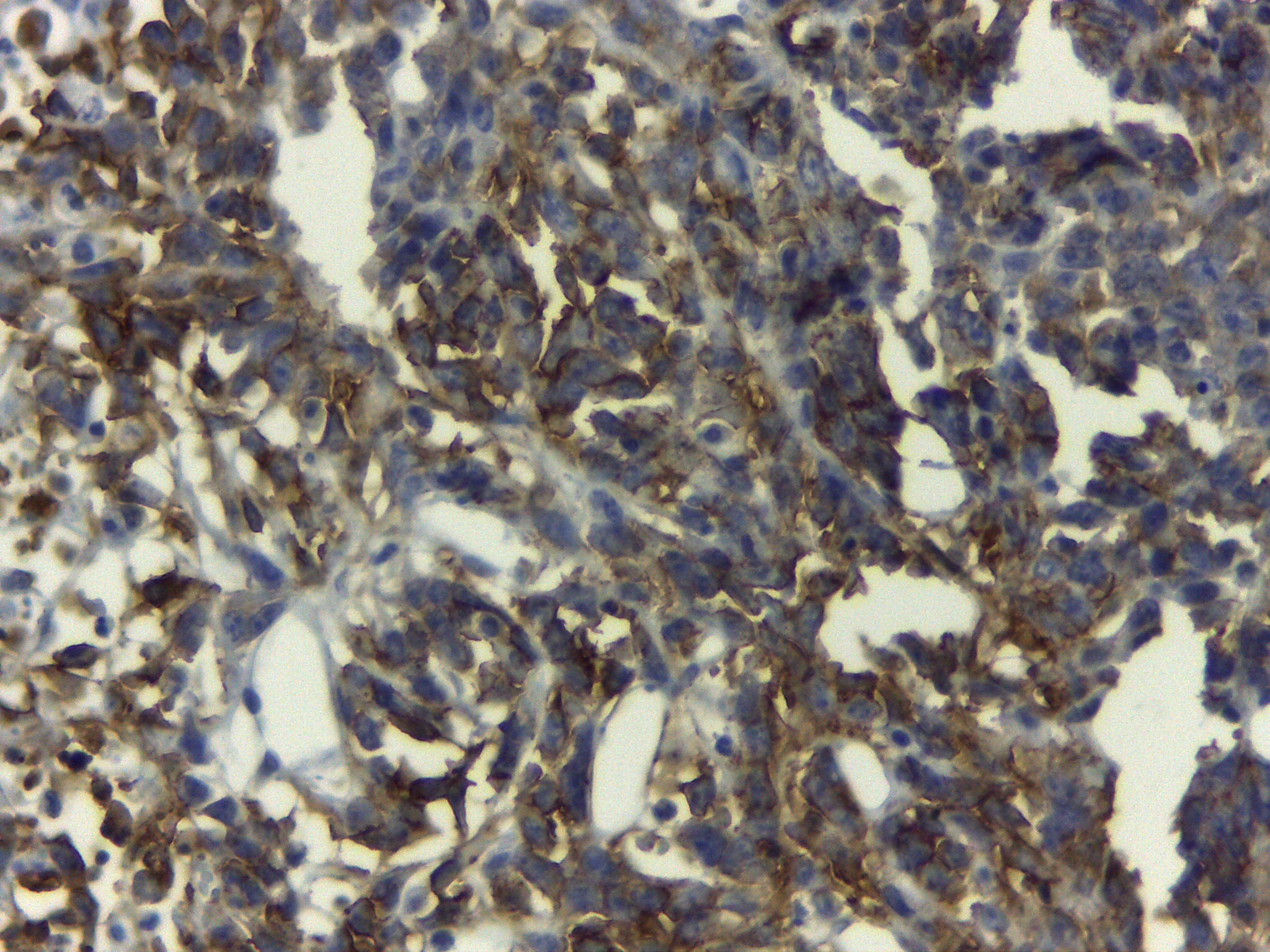

Supplement: S4 Fig — (ZIP) [file pone.0188960.s017.zip › Ca IX IHC image CON/Ca IX con2-5.jpg]

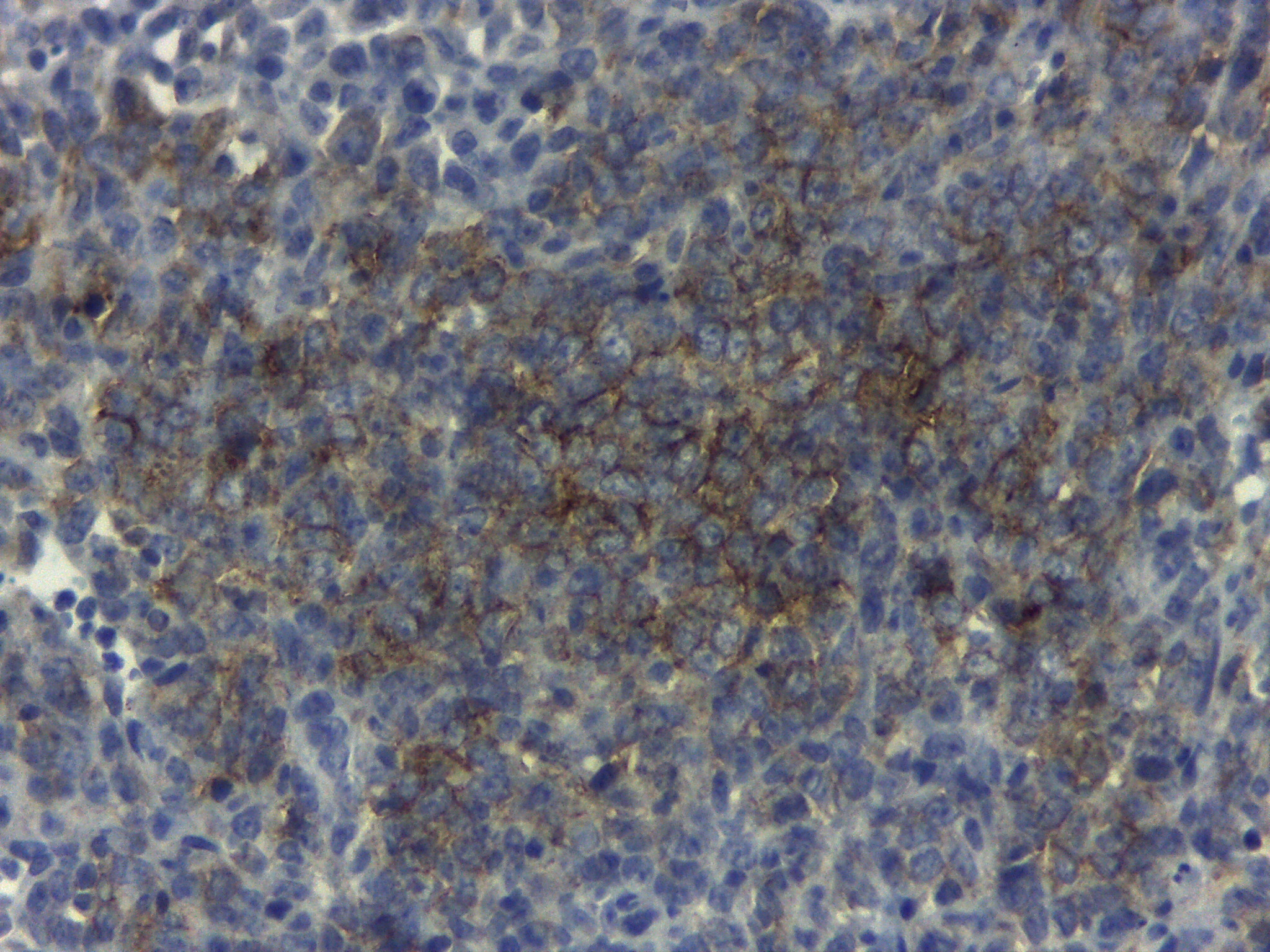

Supplement: S4 Fig — (ZIP) [file pone.0188960.s017.zip › Ca IX IHC image CON/Ca IX con3-1.jpg]

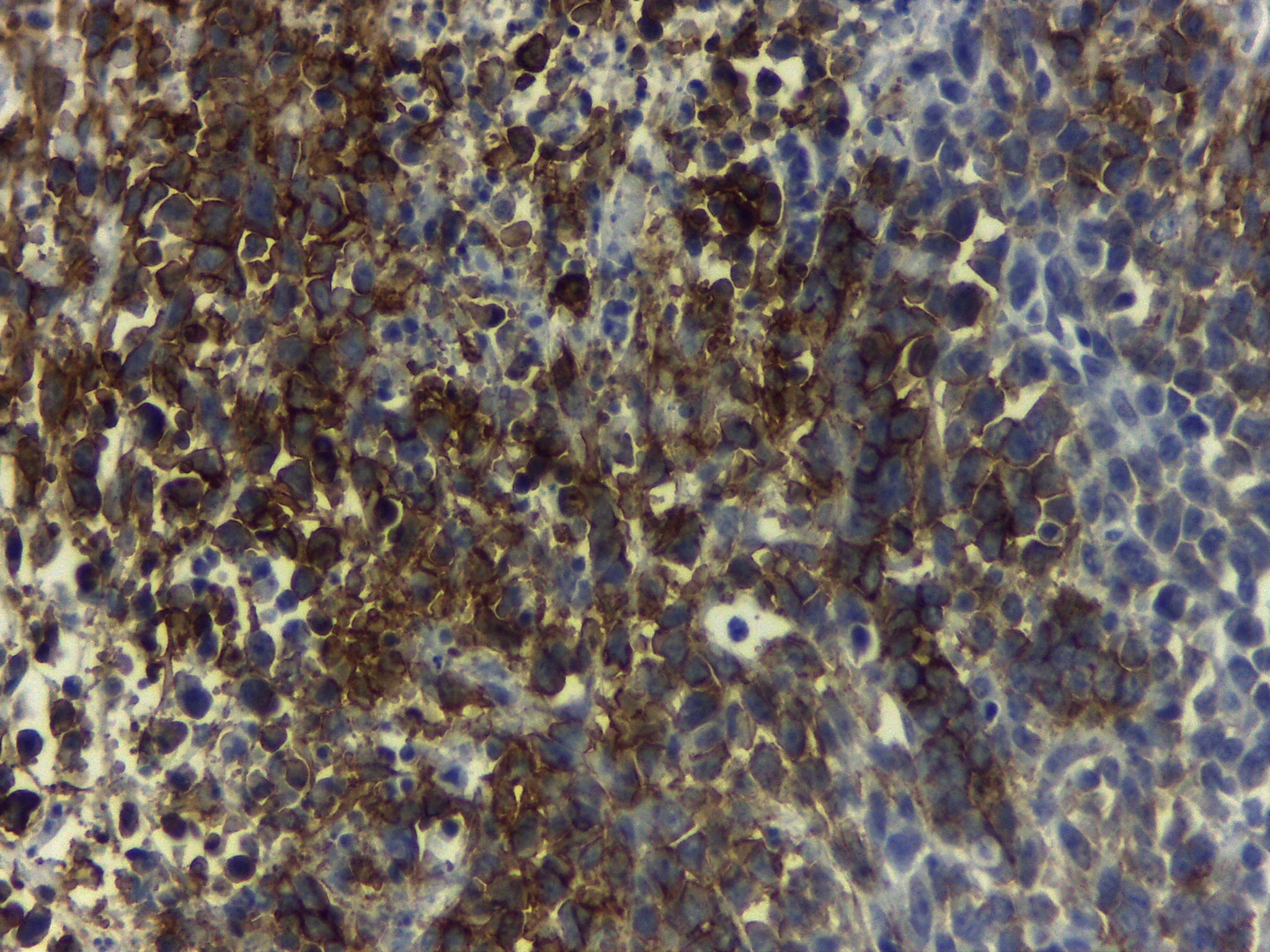

Supplement: S4 Fig — (ZIP) [file pone.0188960.s017.zip › Ca IX IHC image CON/Ca IX con3-2.jpg]

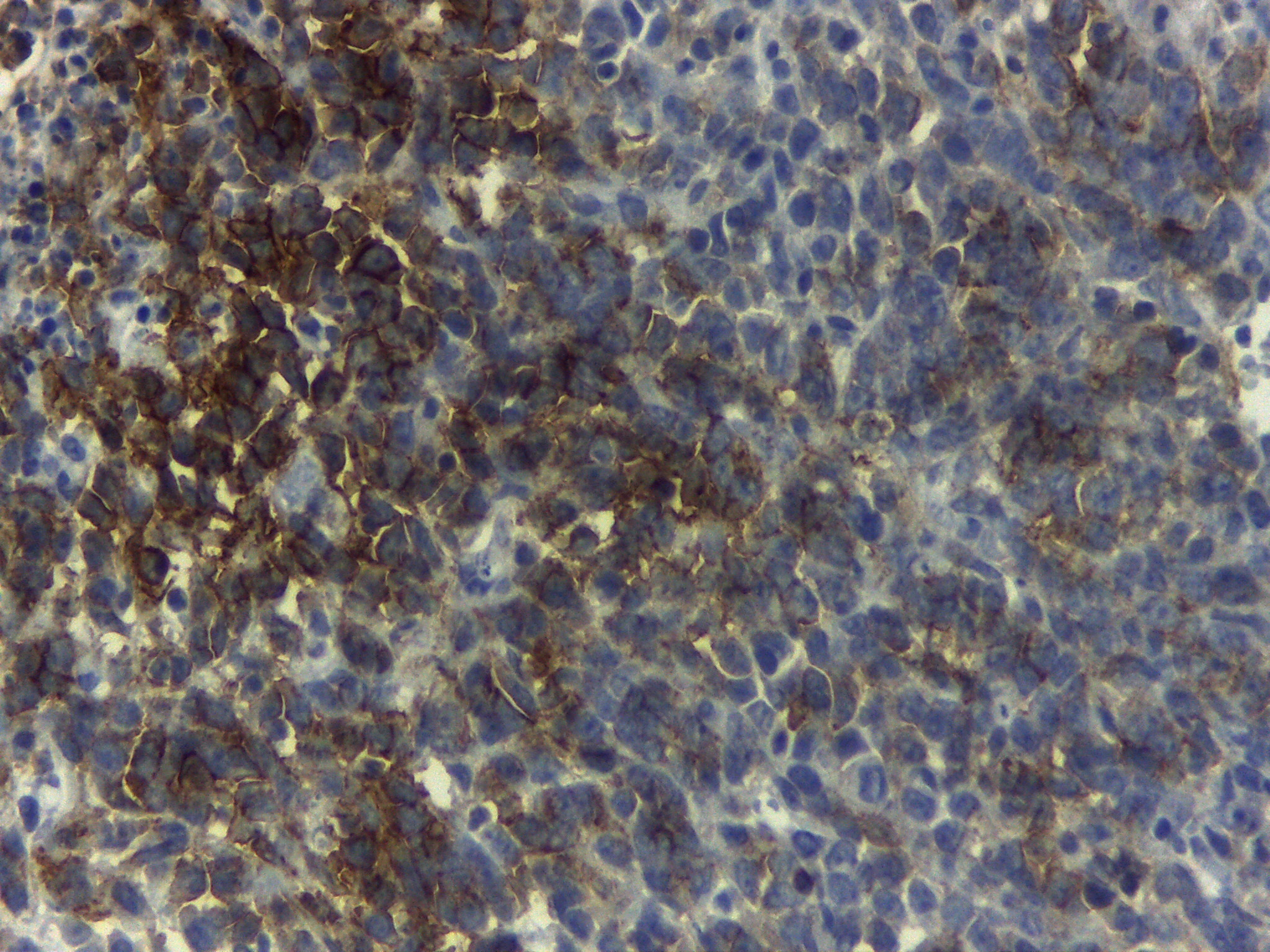

Supplement: S4 Fig — (ZIP) [file pone.0188960.s017.zip › Ca IX IHC image CON/Ca IX con3-3.jpg]

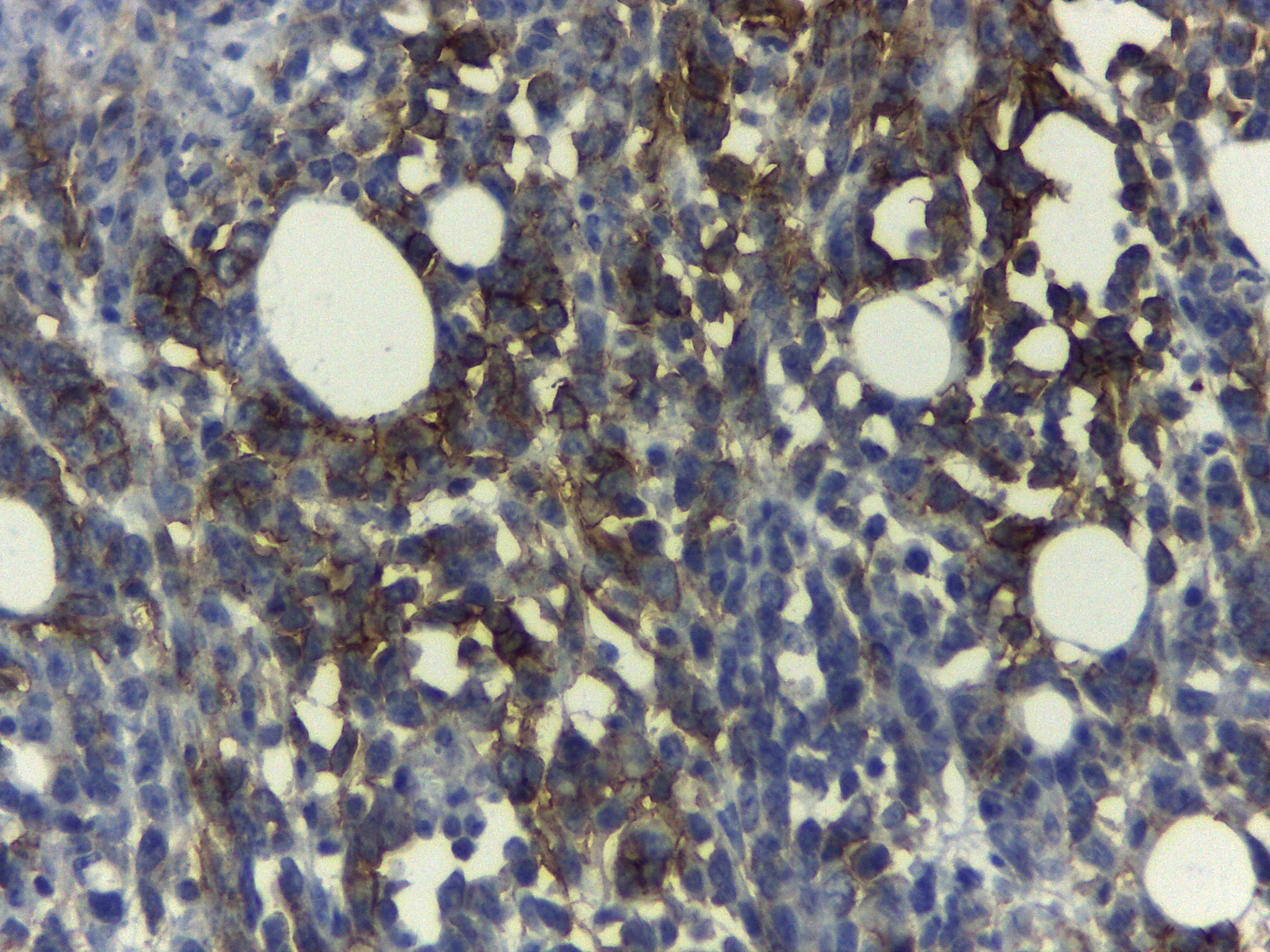

Supplement: S4 Fig — (ZIP) [file pone.0188960.s017.zip › Ca IX IHC image CON/Ca IX con3-4.jpg]

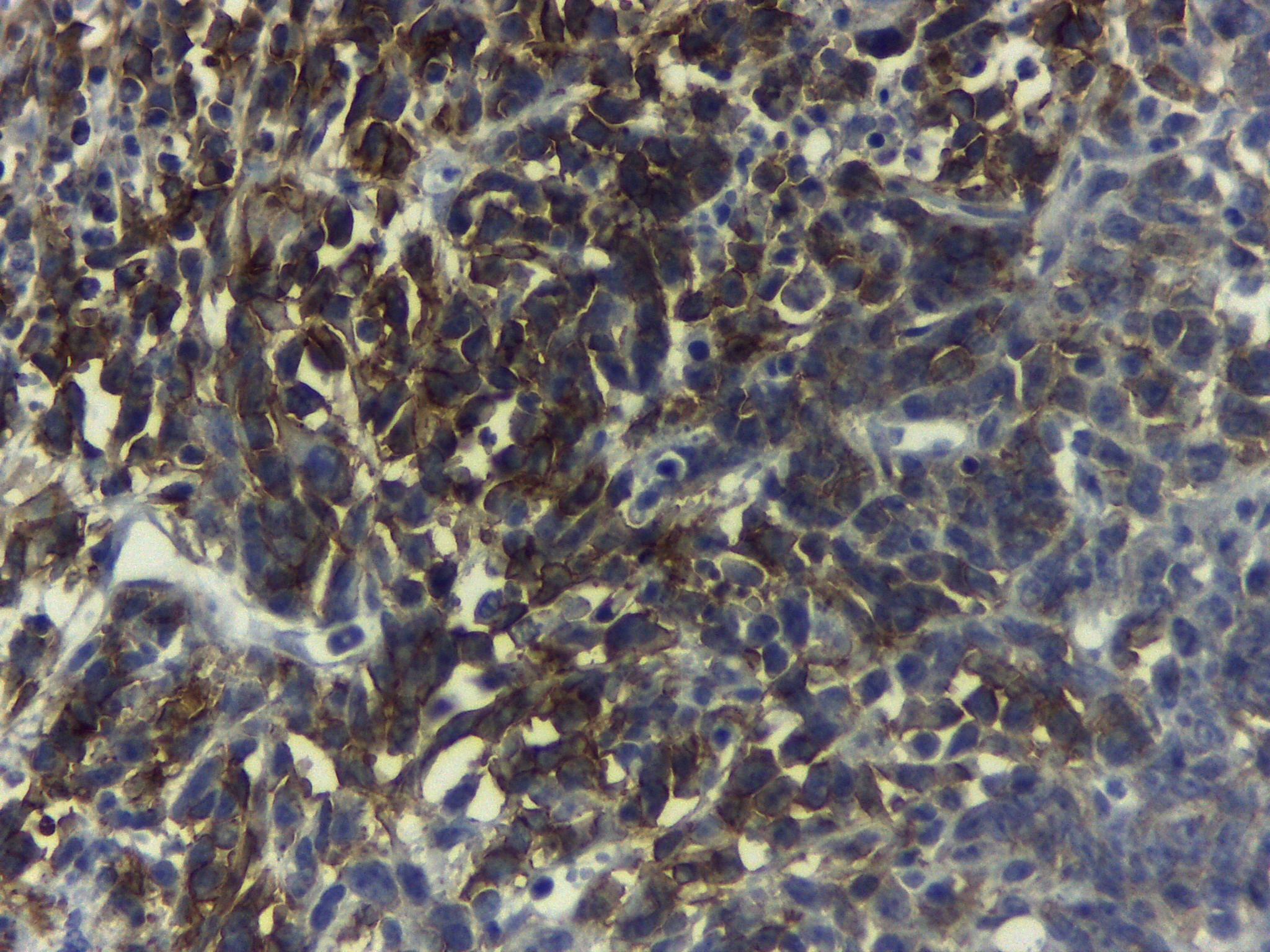

Supplement: S4 Fig — (ZIP) [file pone.0188960.s017.zip › Ca IX IHC image CON/Ca IX con3-5.jpg]

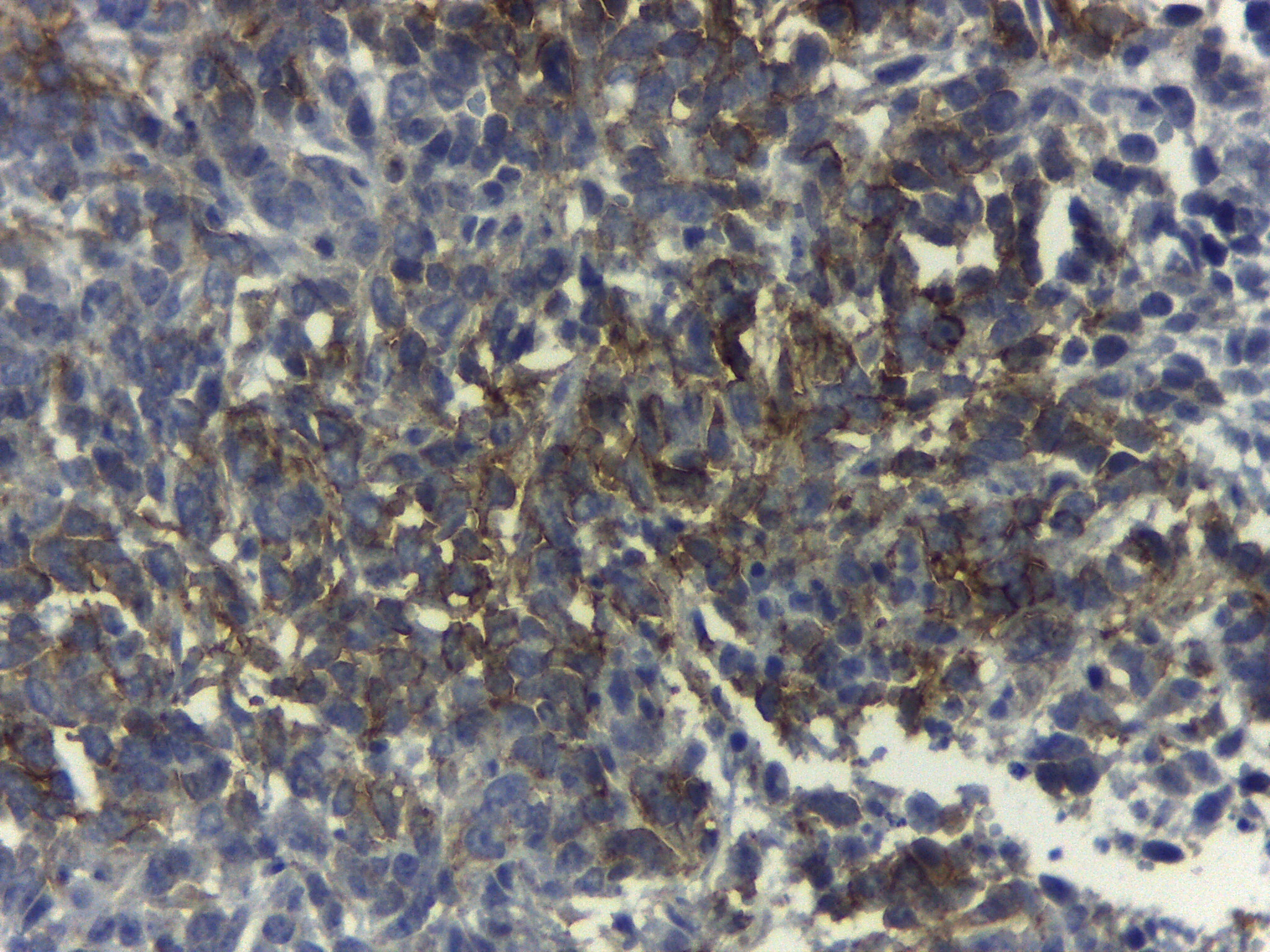

Supplement: S4 Fig — (ZIP) [file pone.0188960.s017.zip › Ca IX IHC image CON/Ca IX con4-1.jpg]

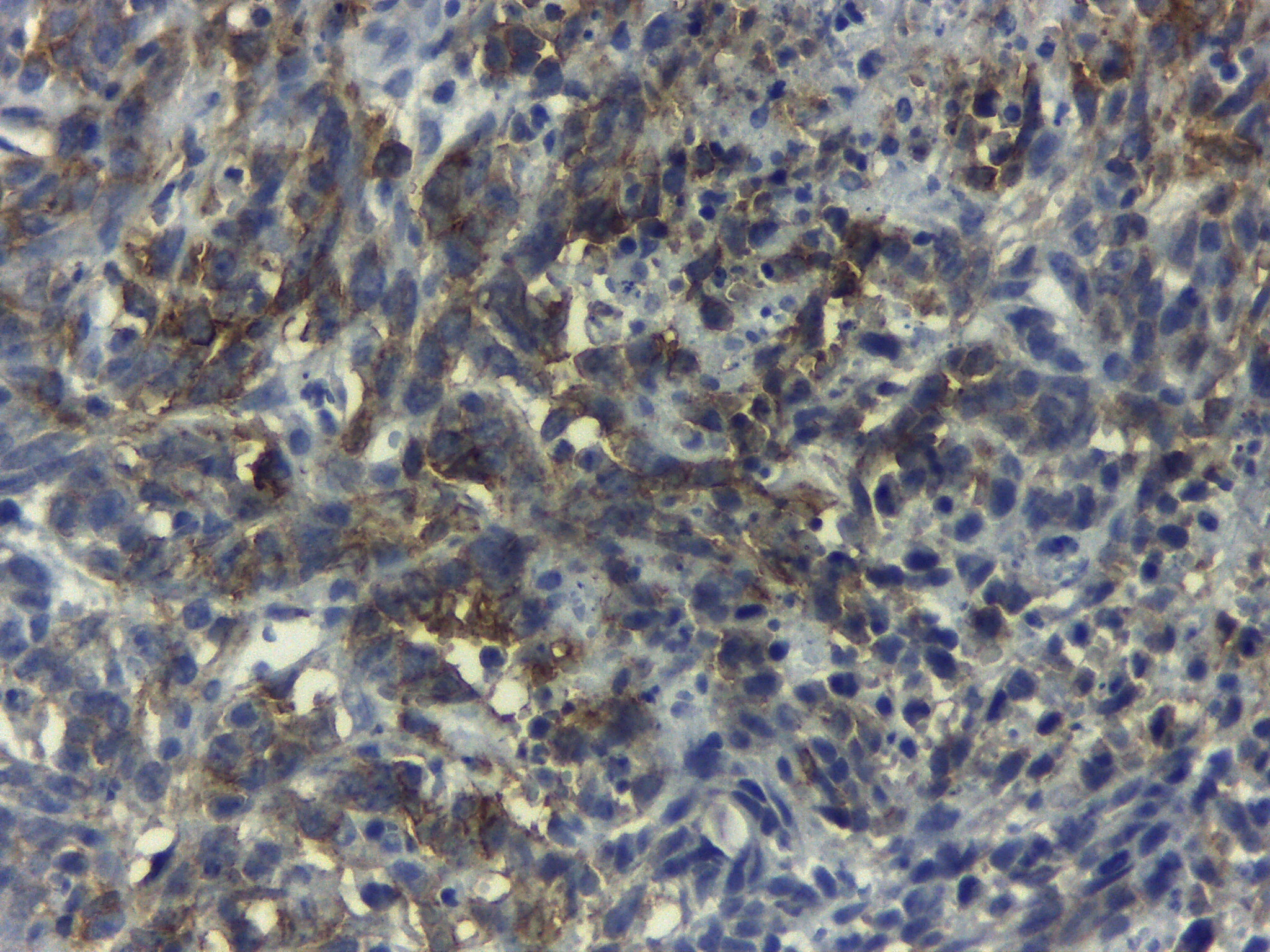

Supplement: S4 Fig — (ZIP) [file pone.0188960.s017.zip › Ca IX IHC image CON/Ca IX con4-2.jpg]

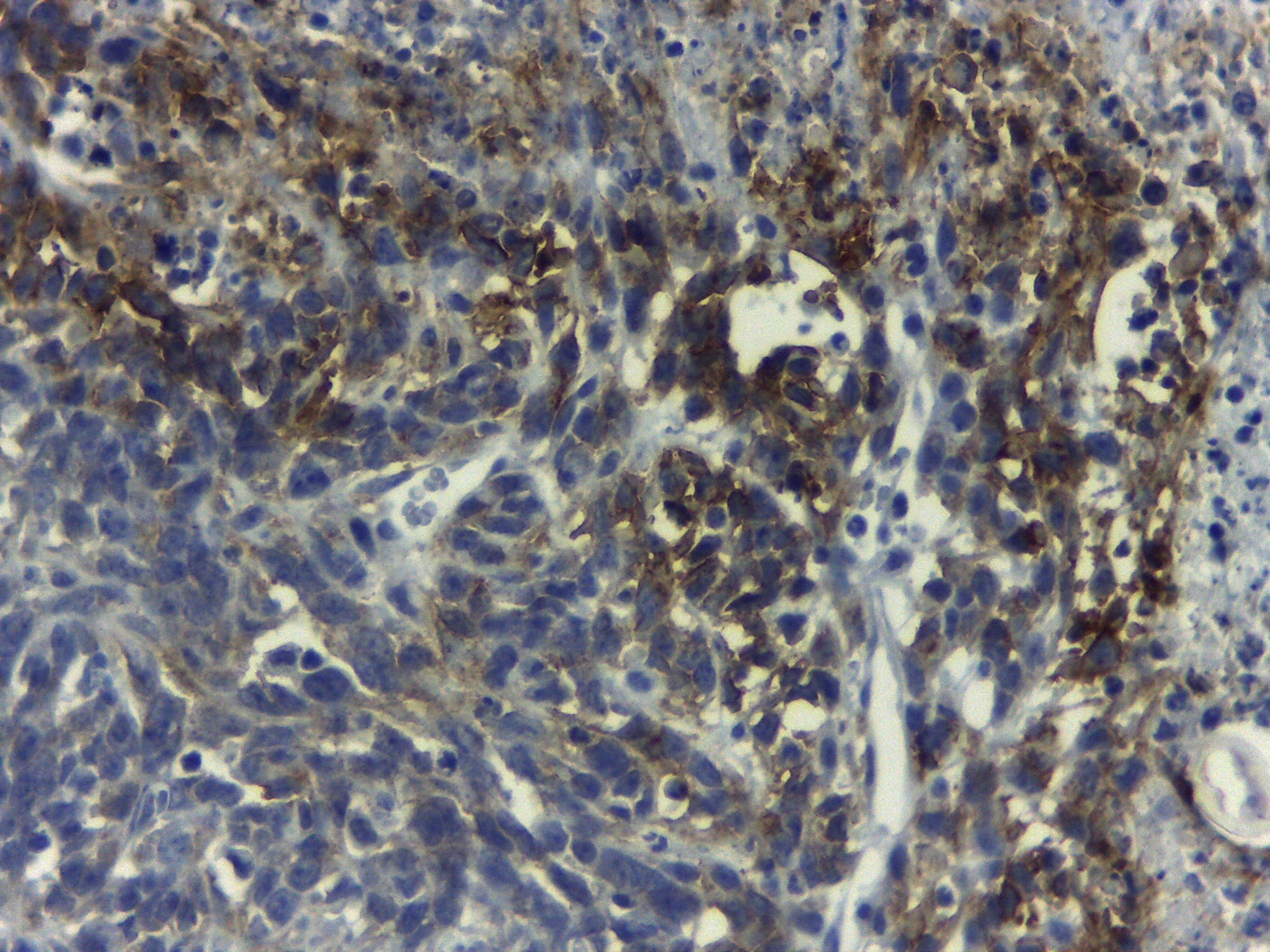

Supplement: S4 Fig — (ZIP) [file pone.0188960.s017.zip › Ca IX IHC image CON/Ca IX con4-3.jpg]

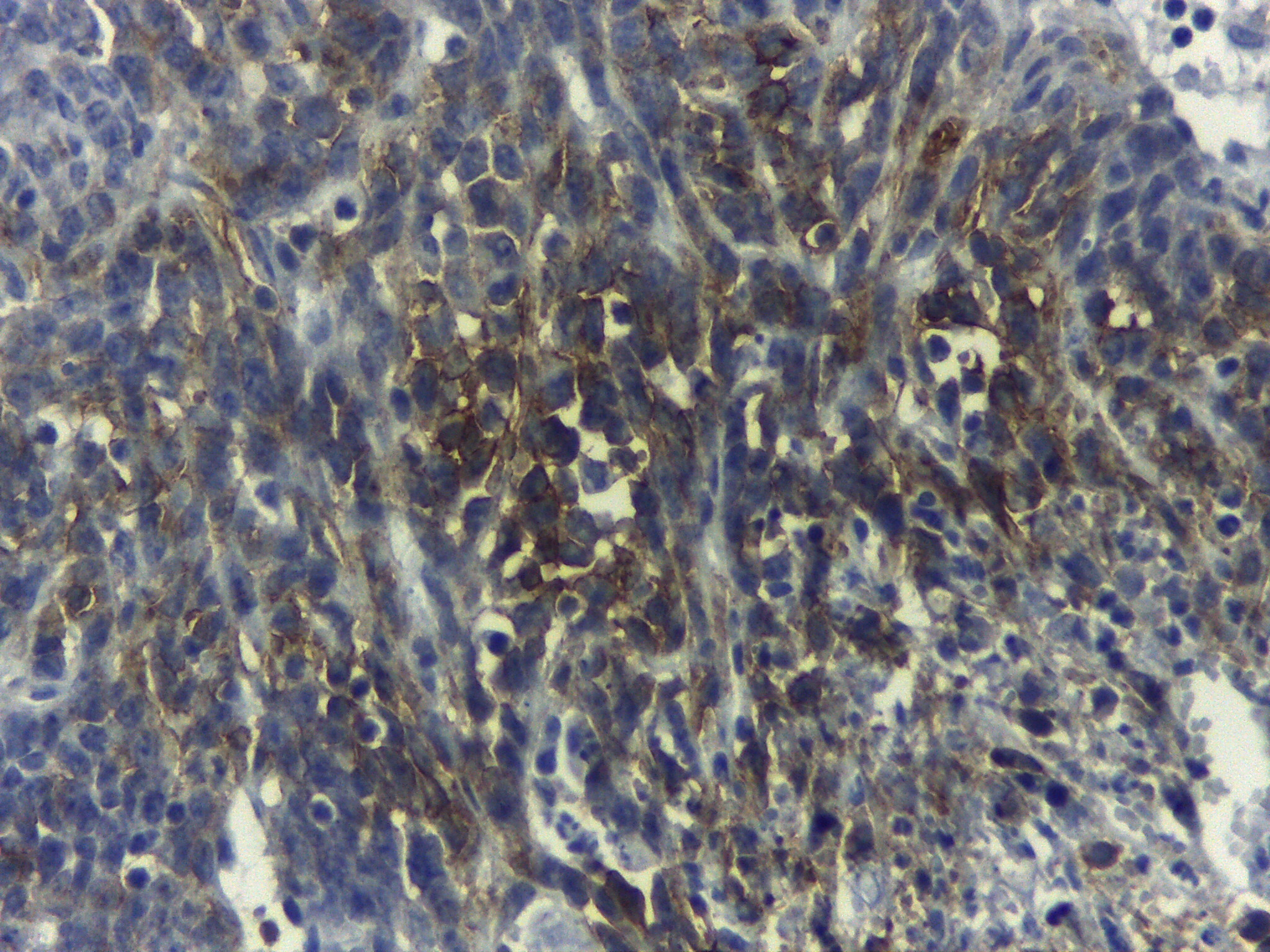

Supplement: S4 Fig — (ZIP) [file pone.0188960.s017.zip › Ca IX IHC image CON/Ca IX con4-4.jpg]

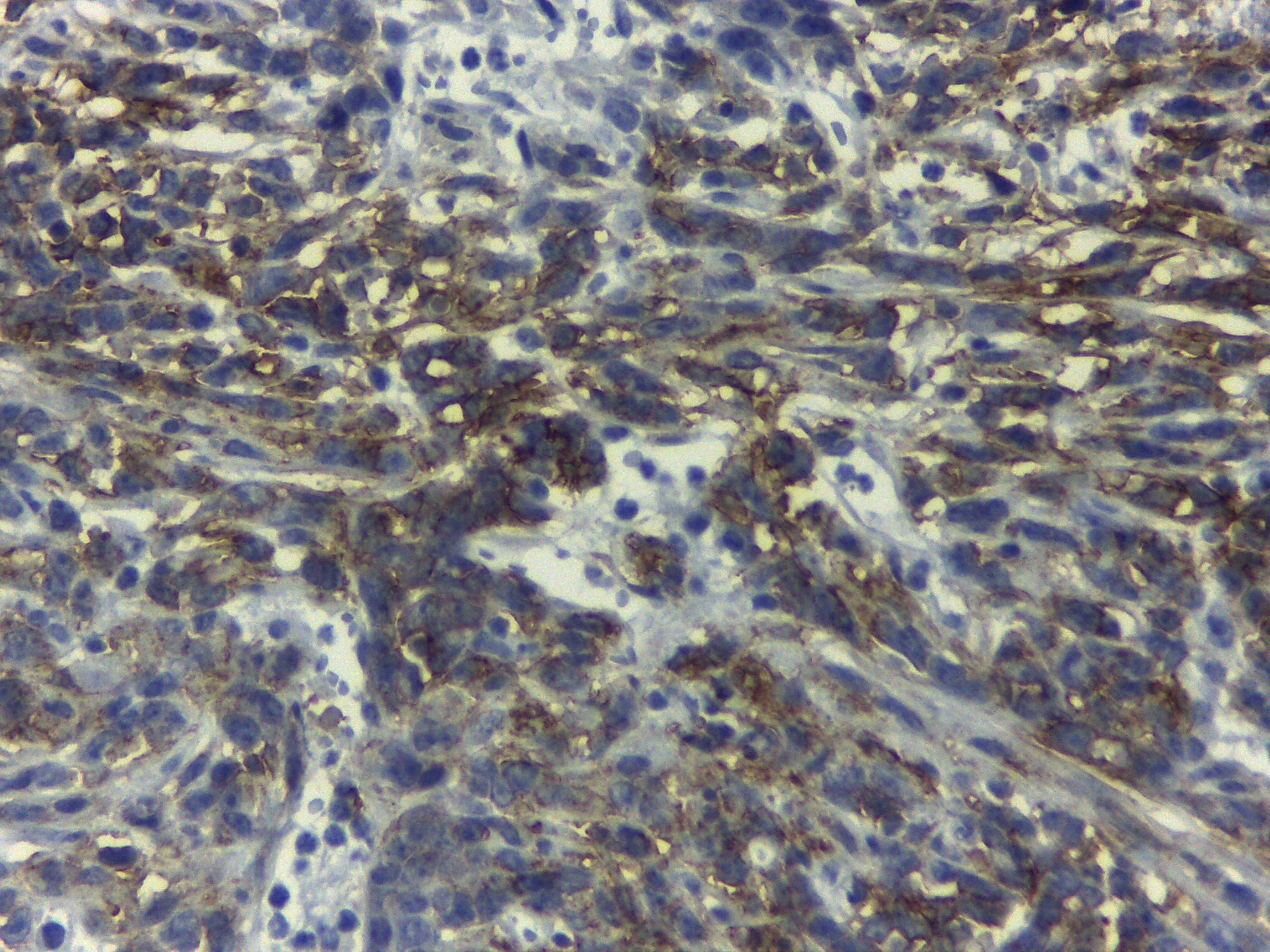

Supplement: S4 Fig — (ZIP) [file pone.0188960.s017.zip › Ca IX IHC image CON/Ca IX con4-5.jpg]

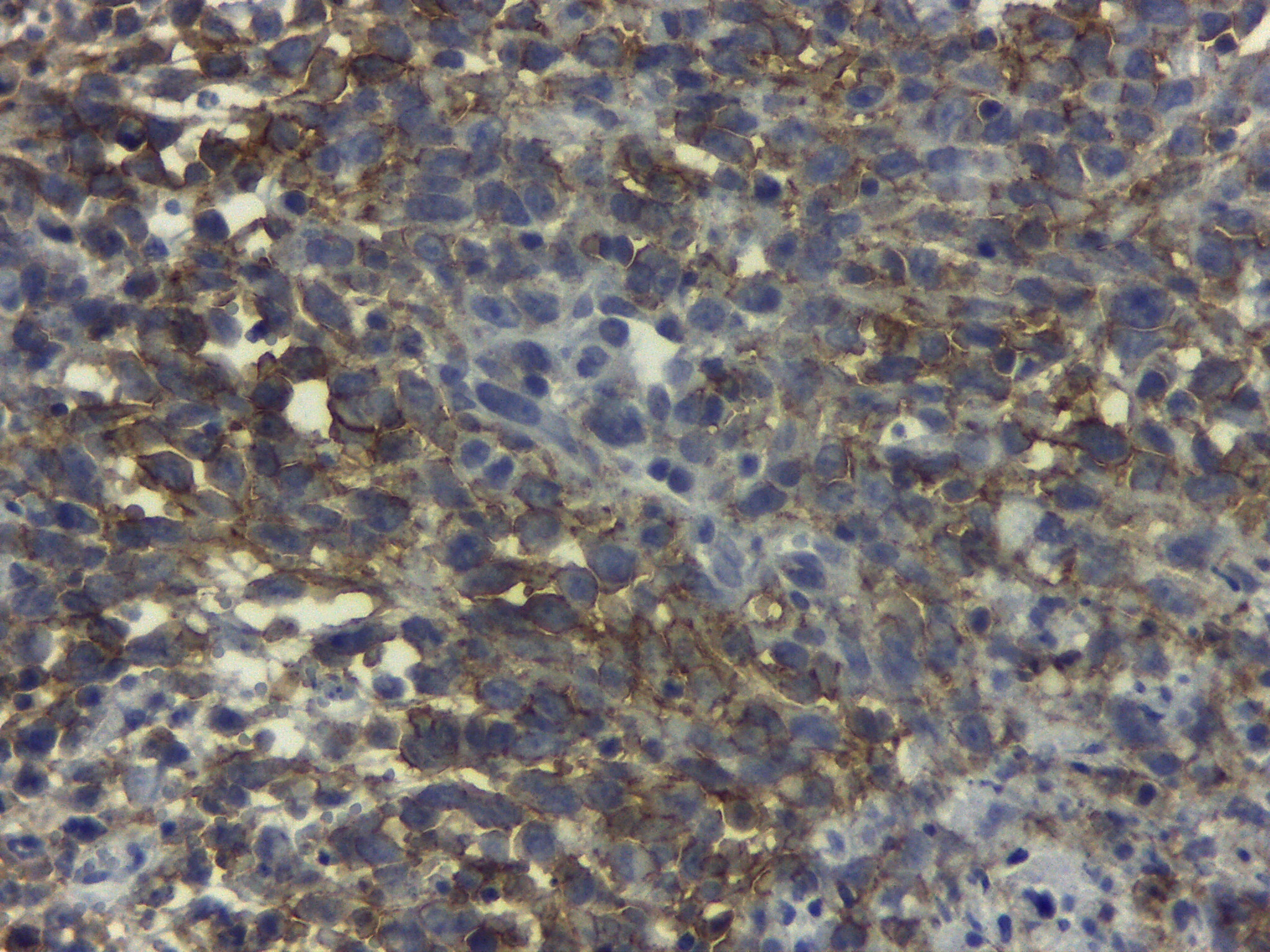

Supplement: S4 Fig — (ZIP) [file pone.0188960.s017.zip › Ca IX IHC image CON/Ca IX con5-1.jpg]

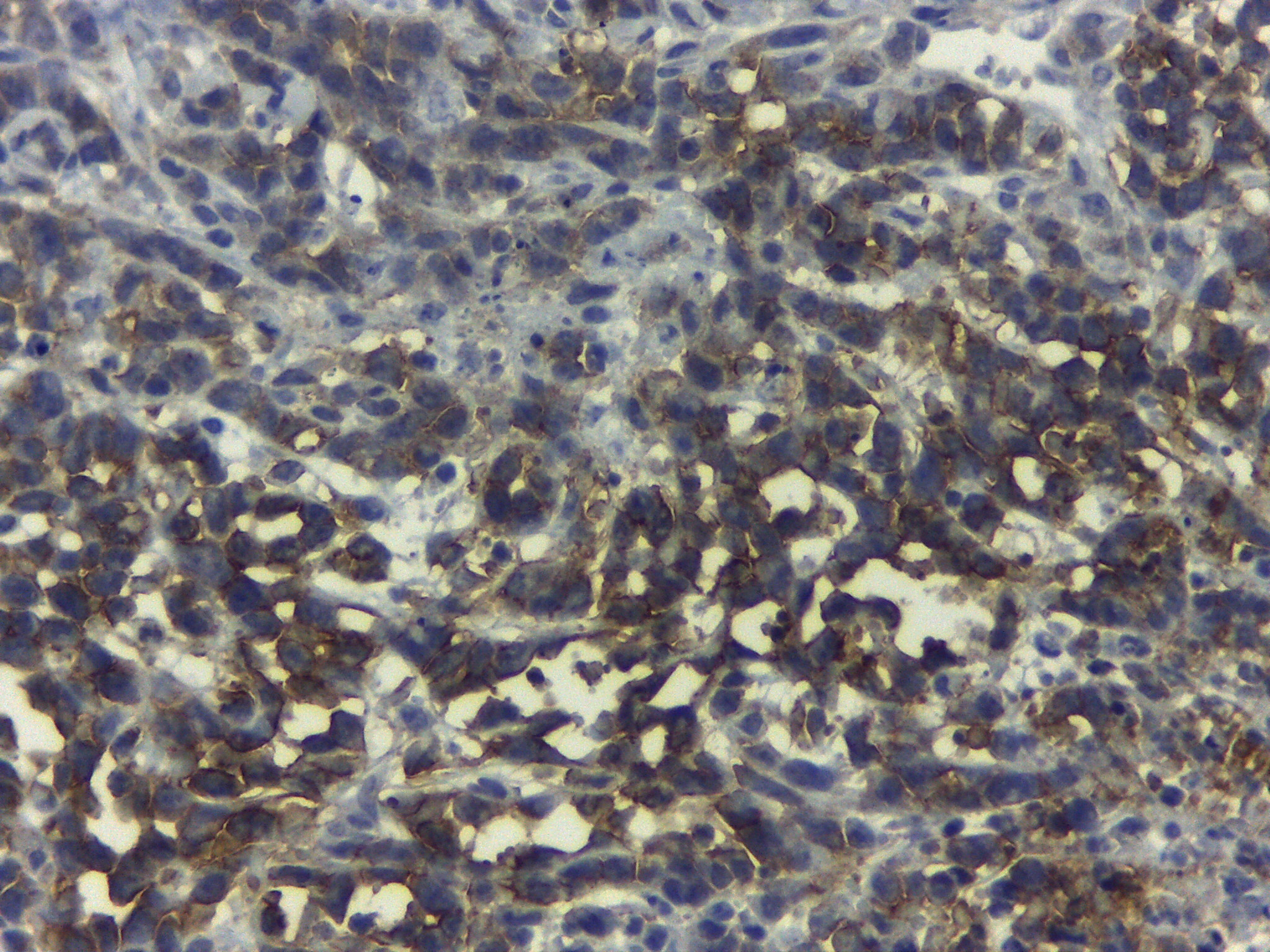

Supplement: S4 Fig — (ZIP) [file pone.0188960.s017.zip › Ca IX IHC image CON/Ca IX con5-2.jpg]

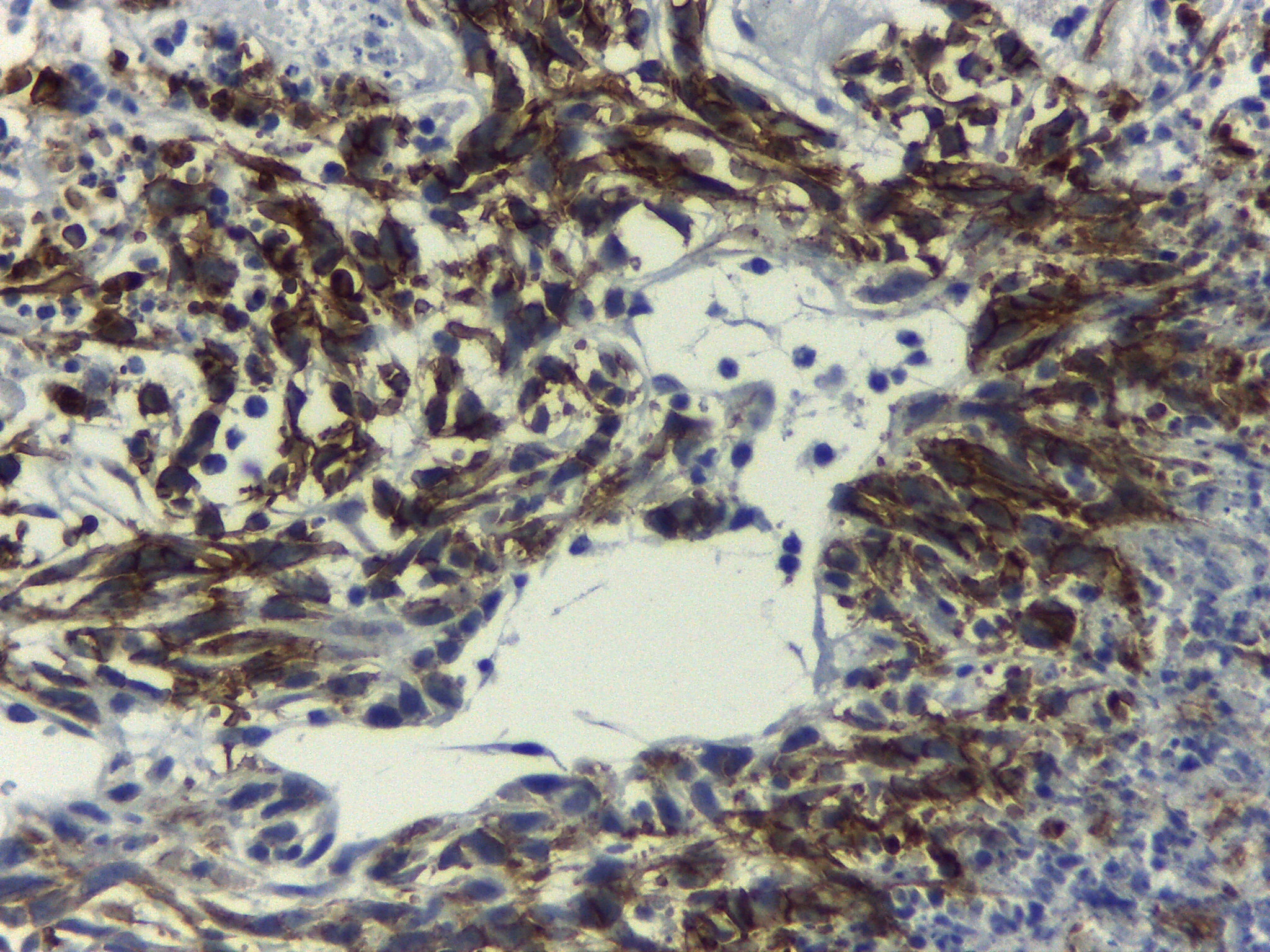

Supplement: S4 Fig — (ZIP) [file pone.0188960.s017.zip › Ca IX IHC image CON/Ca IX con5-3.jpg]

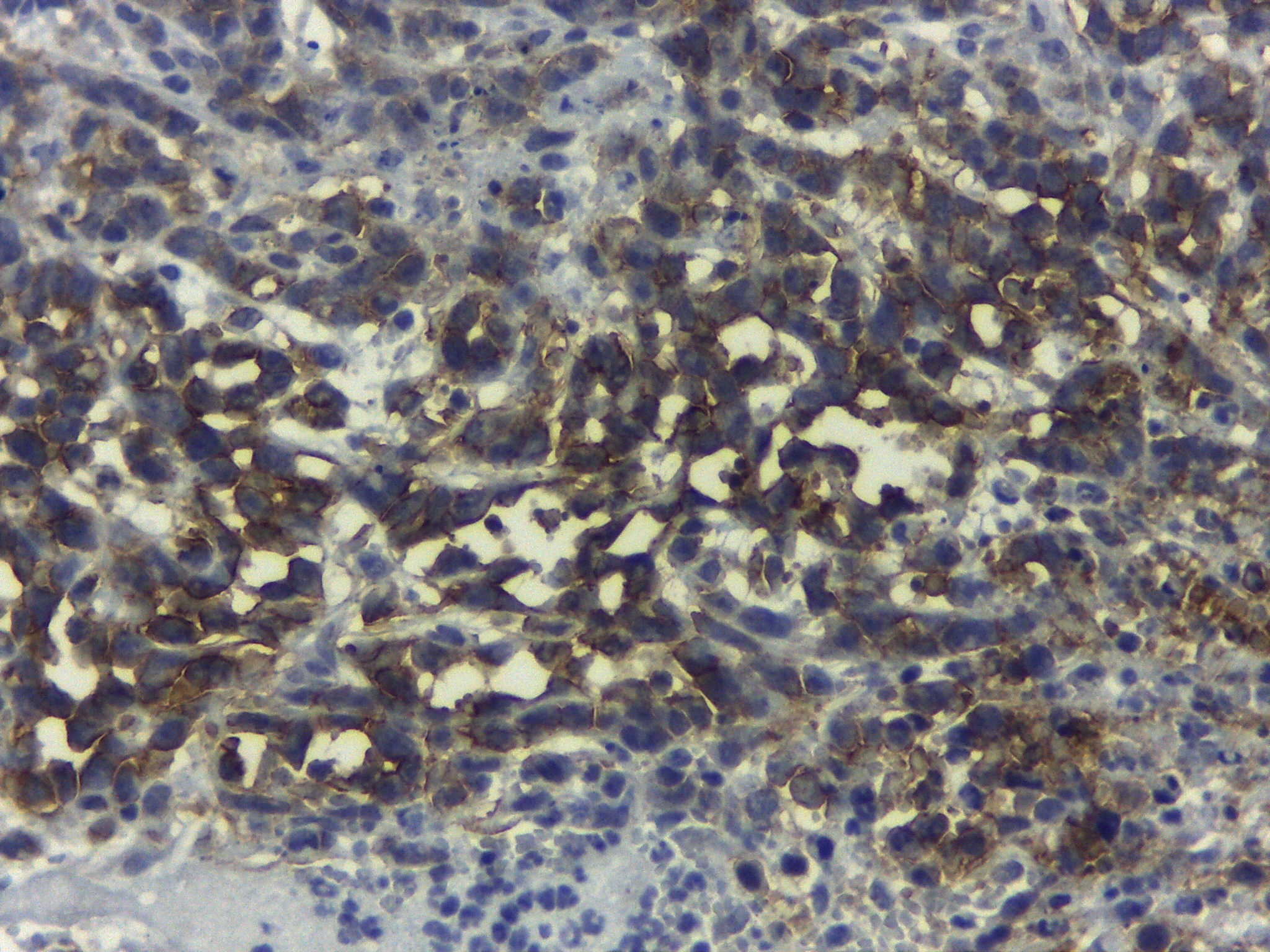

Supplement: S4 Fig — (ZIP) [file pone.0188960.s017.zip › Ca IX IHC image CON/Ca IX con5-4.jpg]

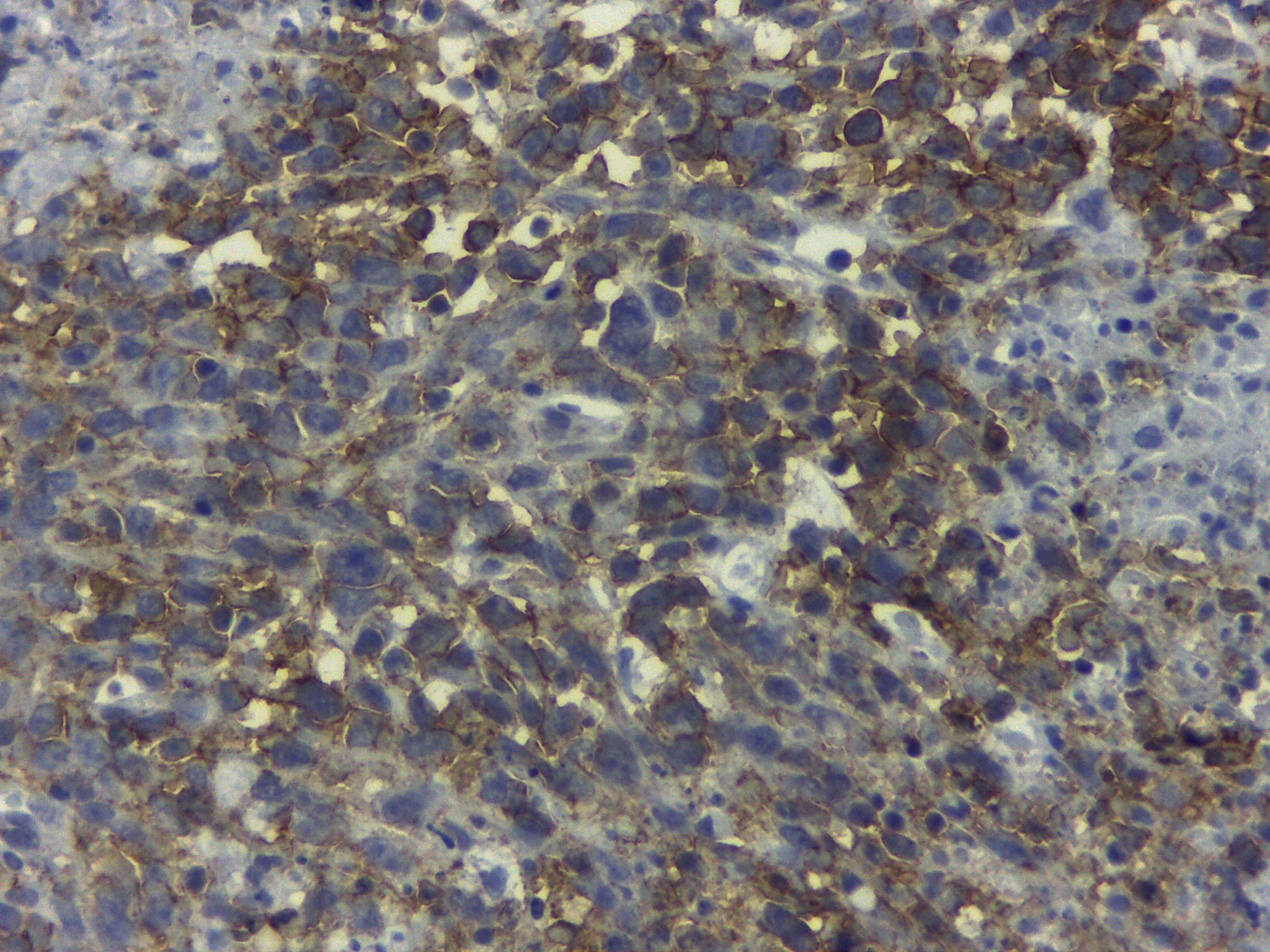

Supplement: S4 Fig — (ZIP) [file pone.0188960.s017.zip › Ca IX IHC image CON/Ca IX con5-5.jpg]

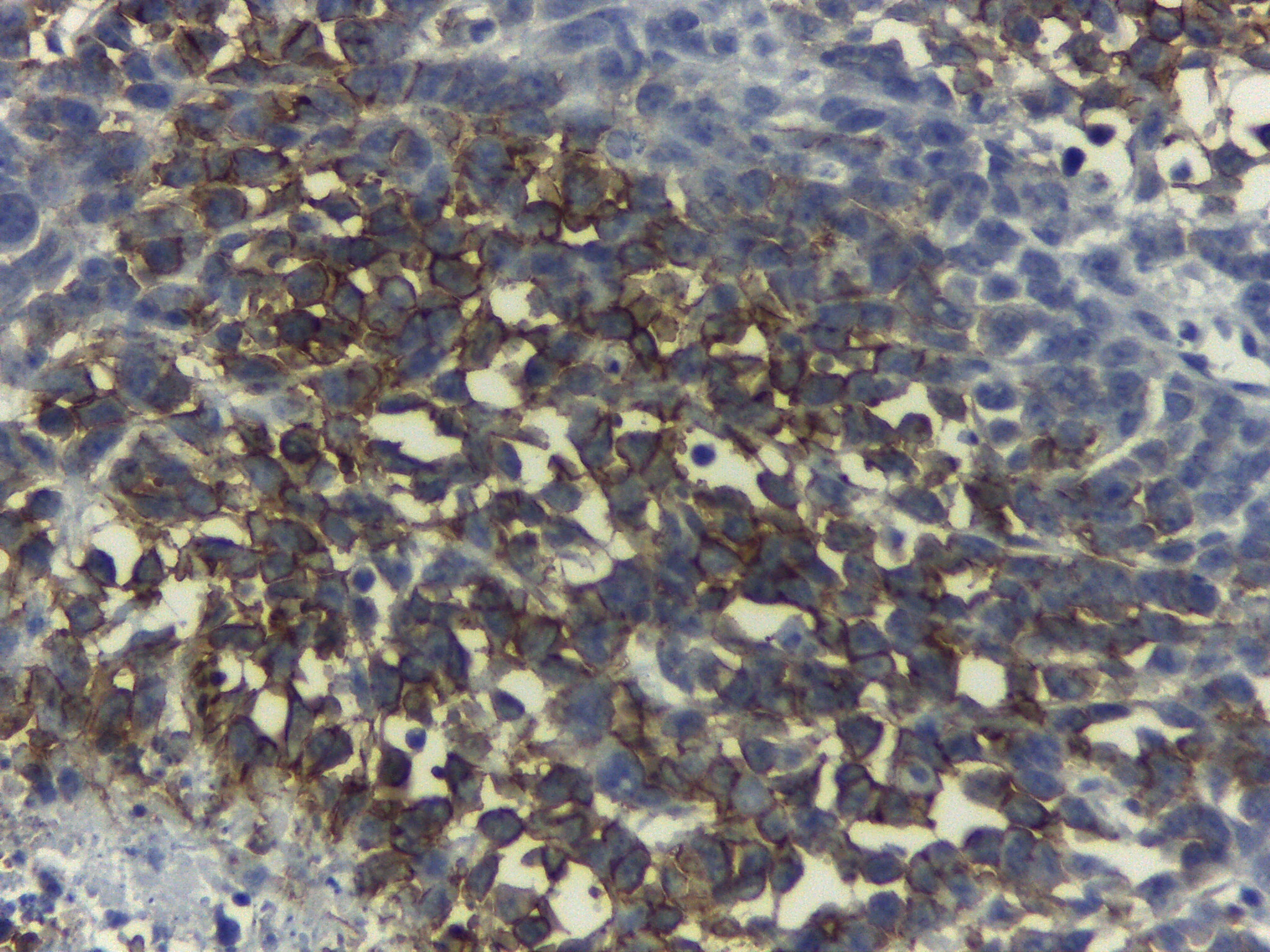

Supplement: S4 Fig — (ZIP) [file pone.0188960.s017.zip › Ca IX IHC image CON/Ca IX con6-1.jpg]

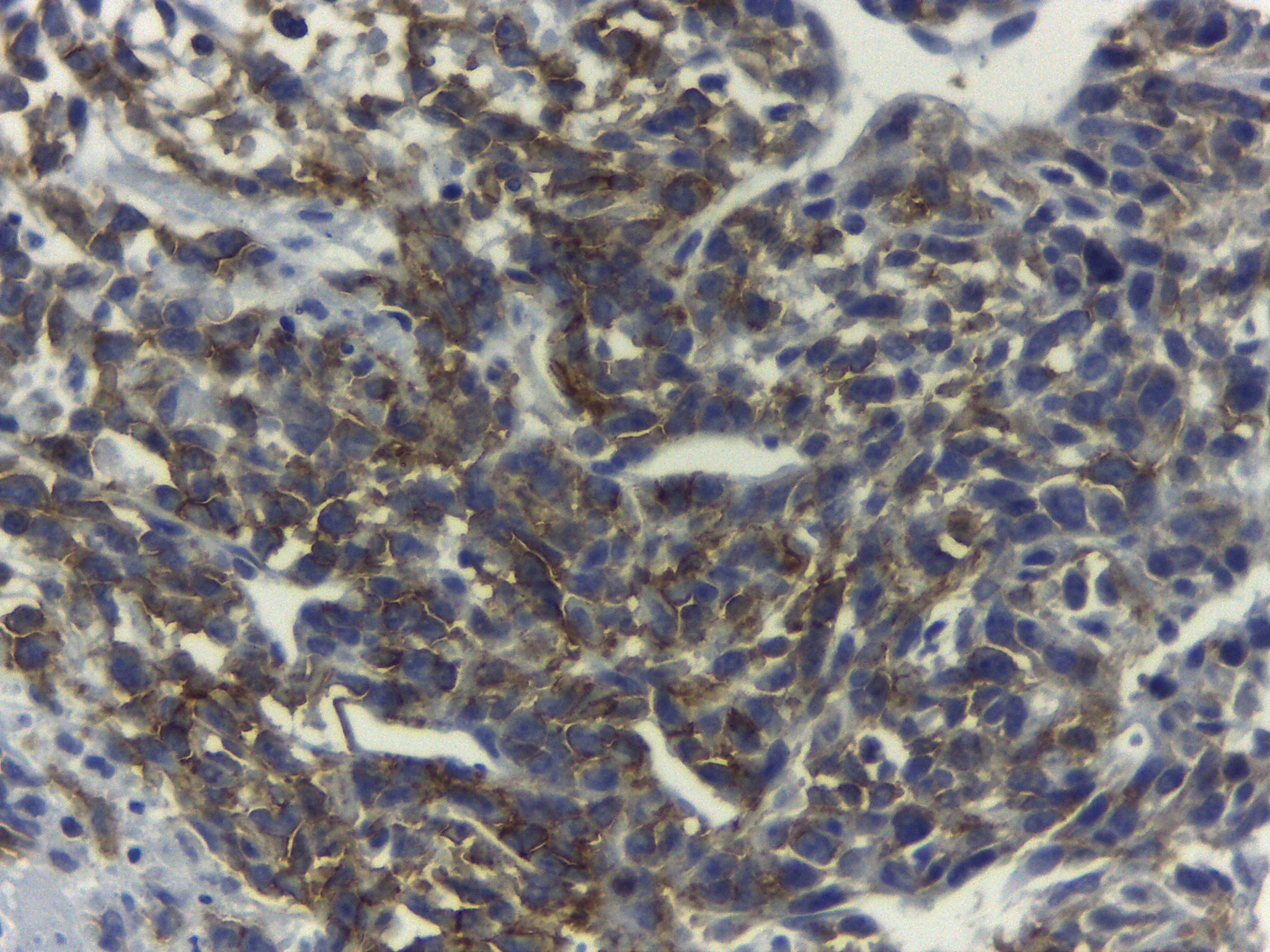

Supplement: S4 Fig — (ZIP) [file pone.0188960.s017.zip › Ca IX IHC image CON/Ca IX con6-2.jpg]

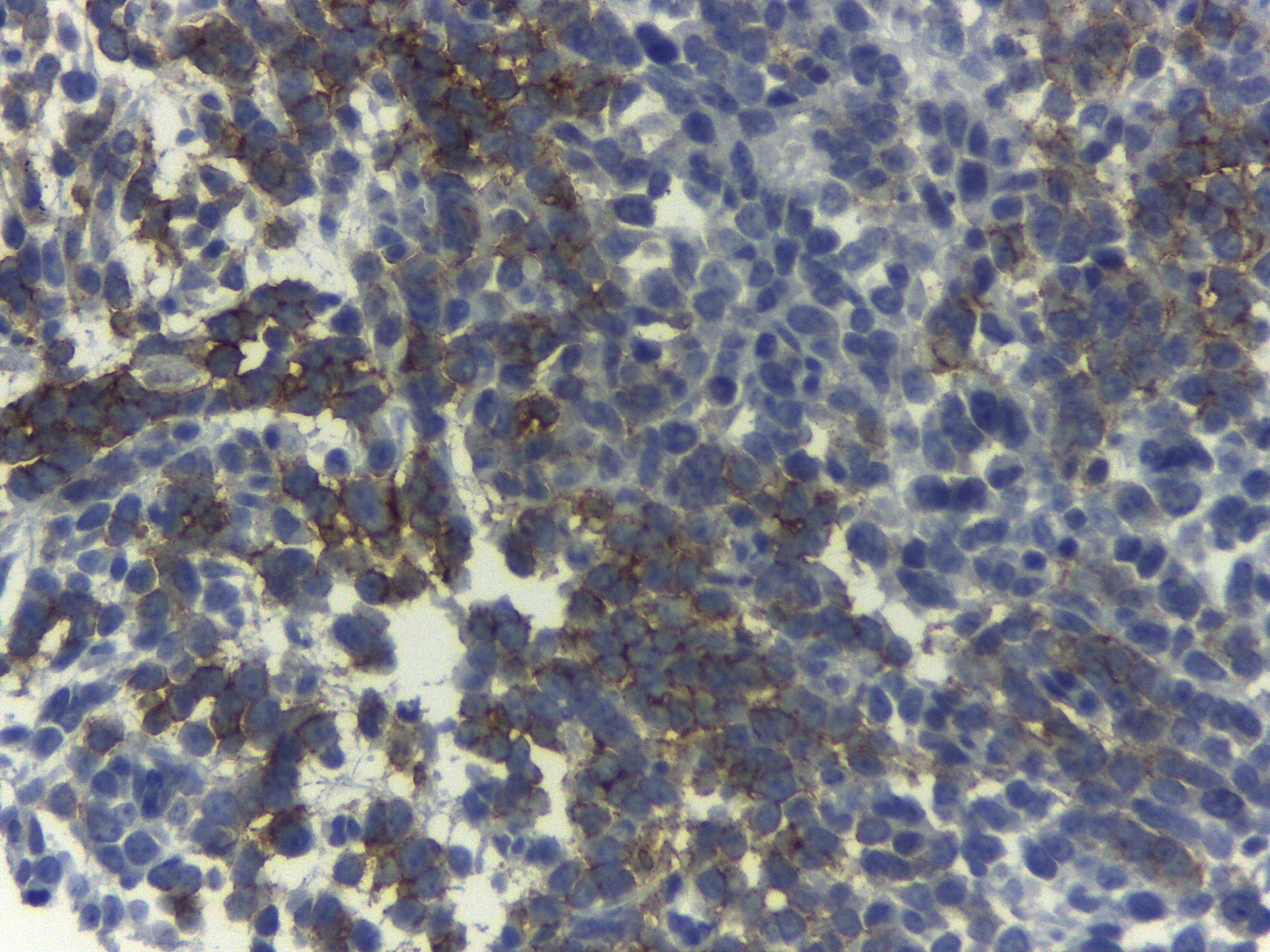

Supplement: S4 Fig — (ZIP) [file pone.0188960.s017.zip › Ca IX IHC image CON/Ca IX con6-3.jpg]

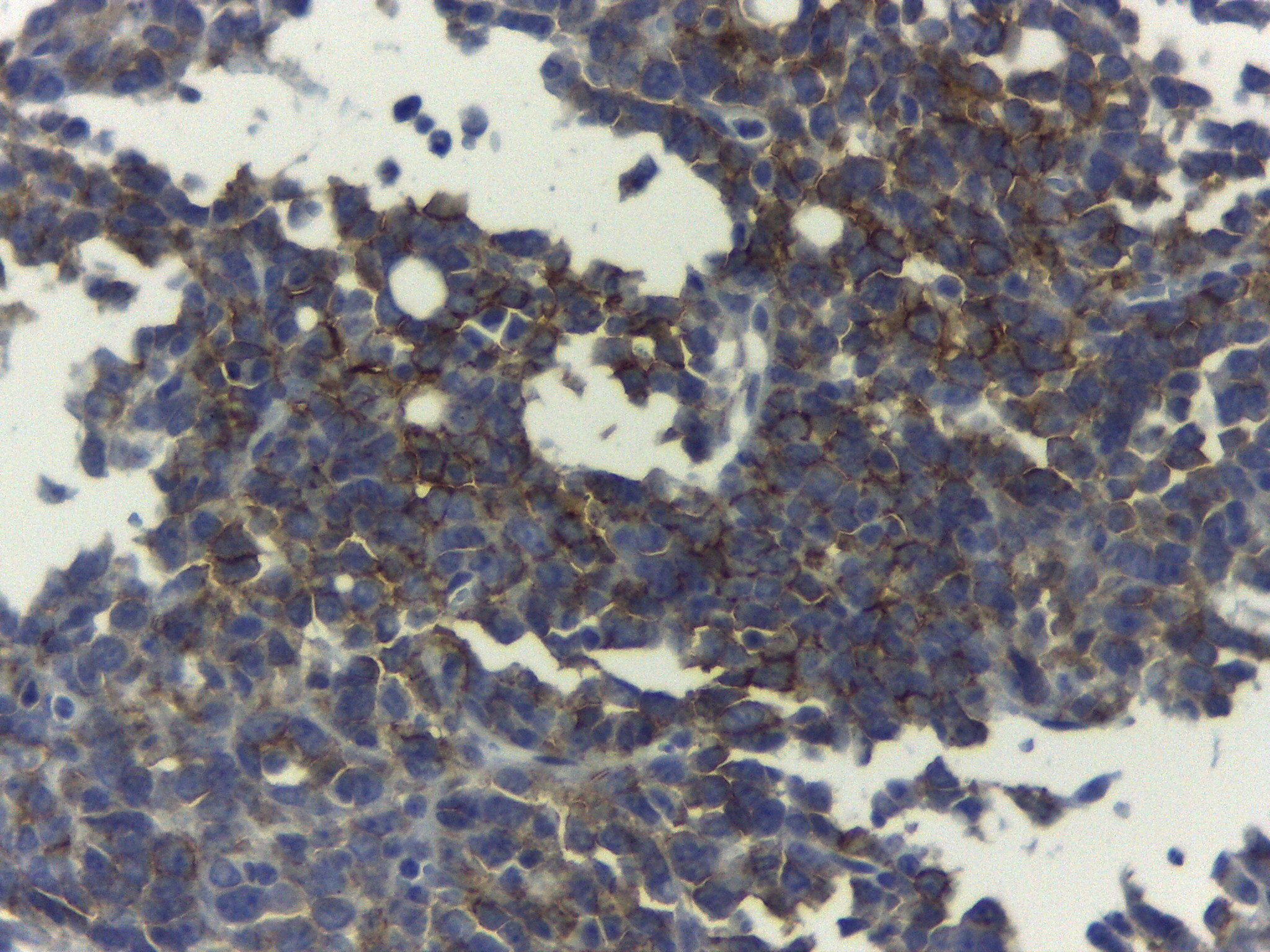

Supplement: S4 Fig — (ZIP) [file pone.0188960.s017.zip › Ca IX IHC image CON/Ca IX con6-4.jpg]

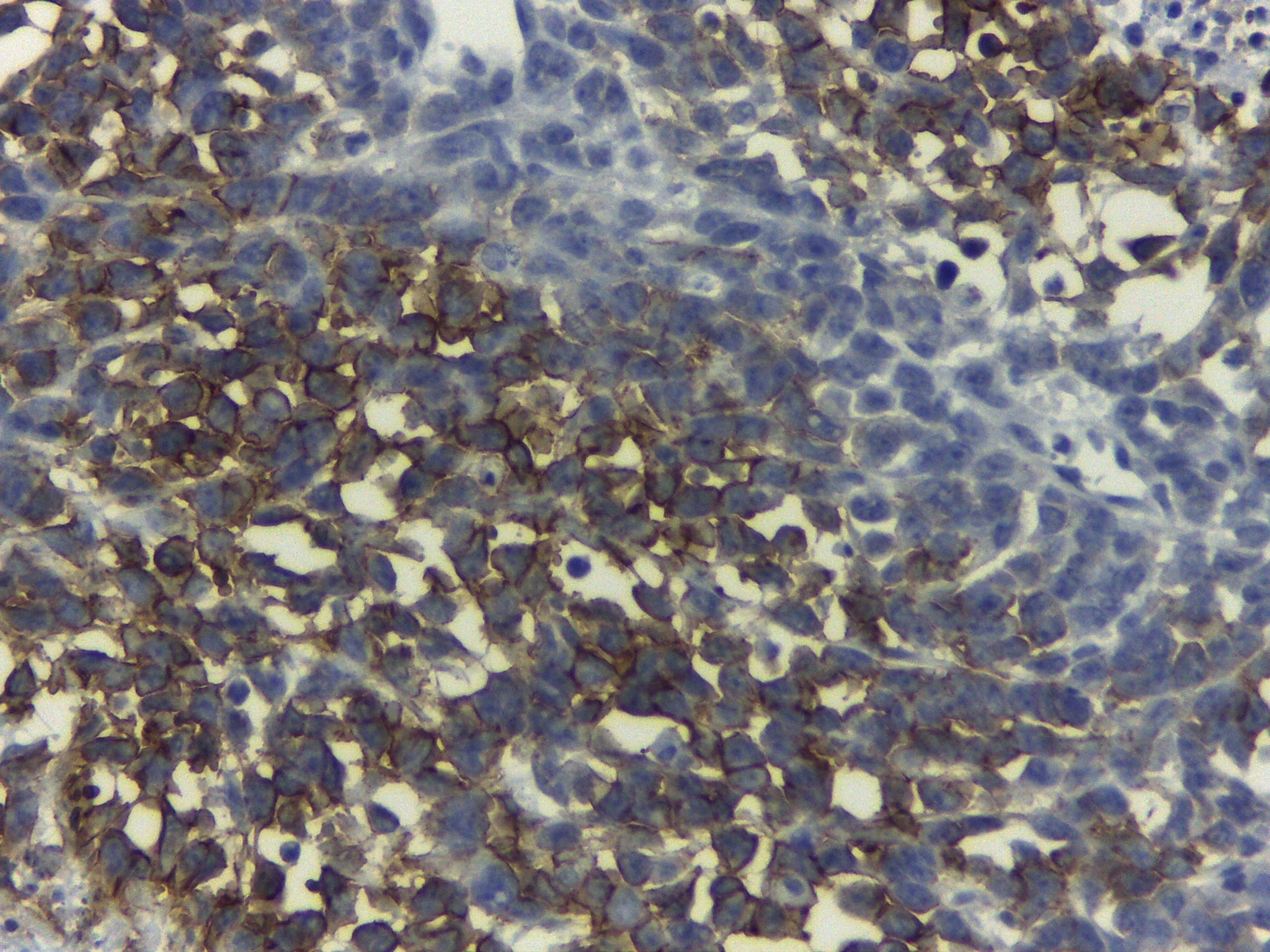

Supplement: S4 Fig — (ZIP) [file pone.0188960.s017.zip › Ca IX IHC image CON/Ca IX con6-5.jpg]

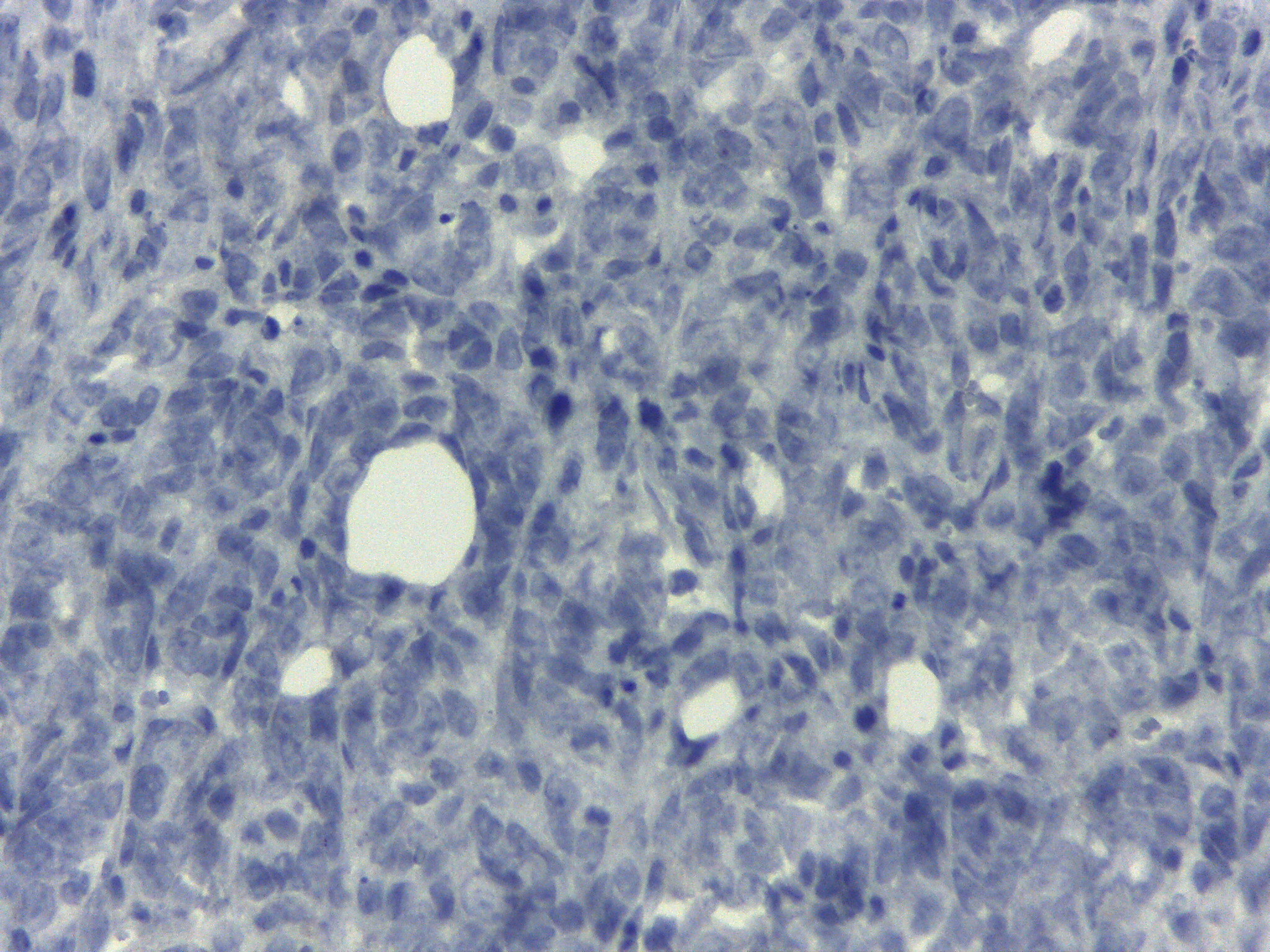

Supplement: S5 Fig — (ZIP) [file pone.0188960.s018.zip › Ca IX IHC image BAC/Ca IX bac1-1.jpg]

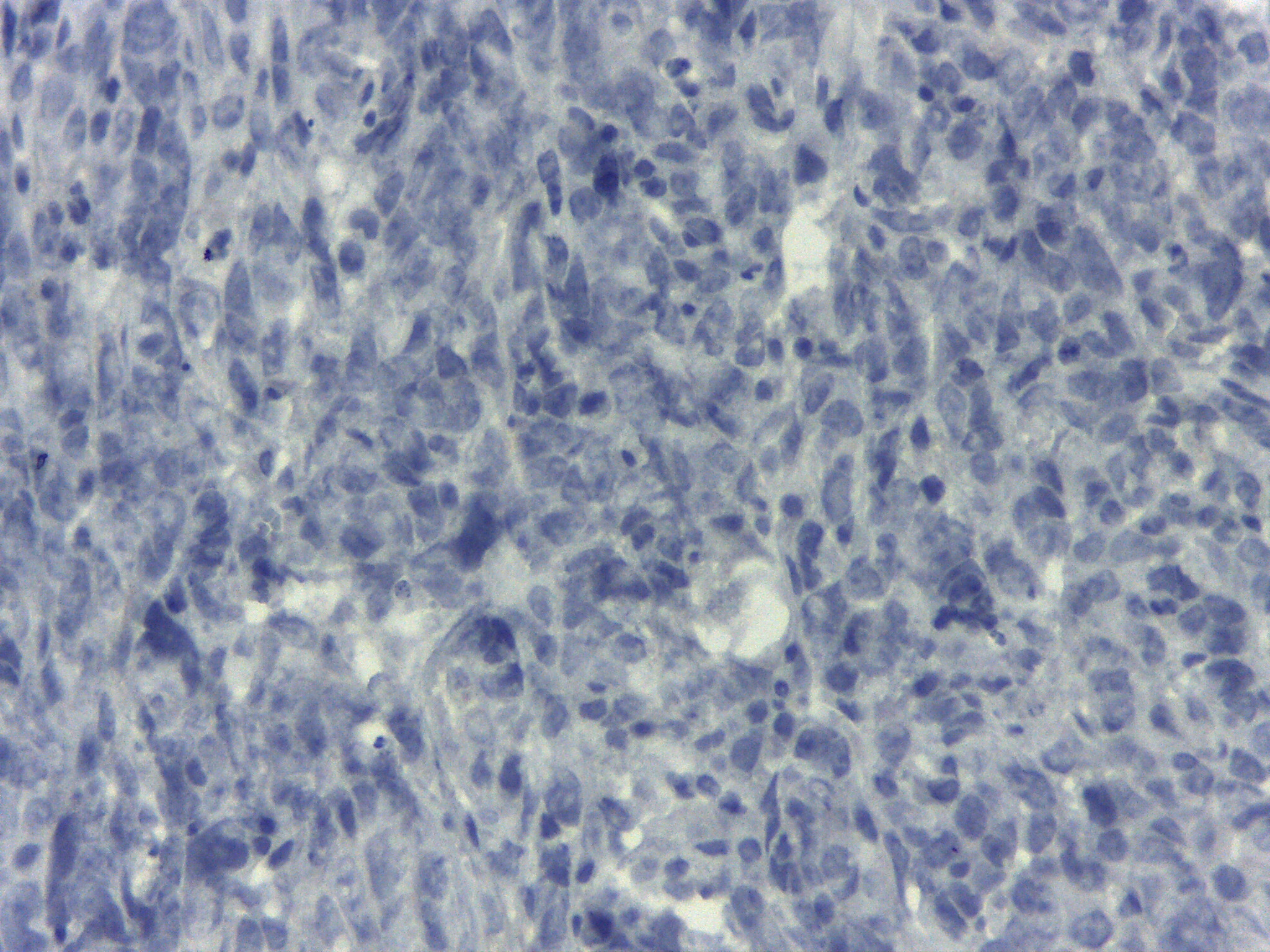

Supplement: S5 Fig — (ZIP) [file pone.0188960.s018.zip › Ca IX IHC image BAC/Ca IX bac1-2.jpg]

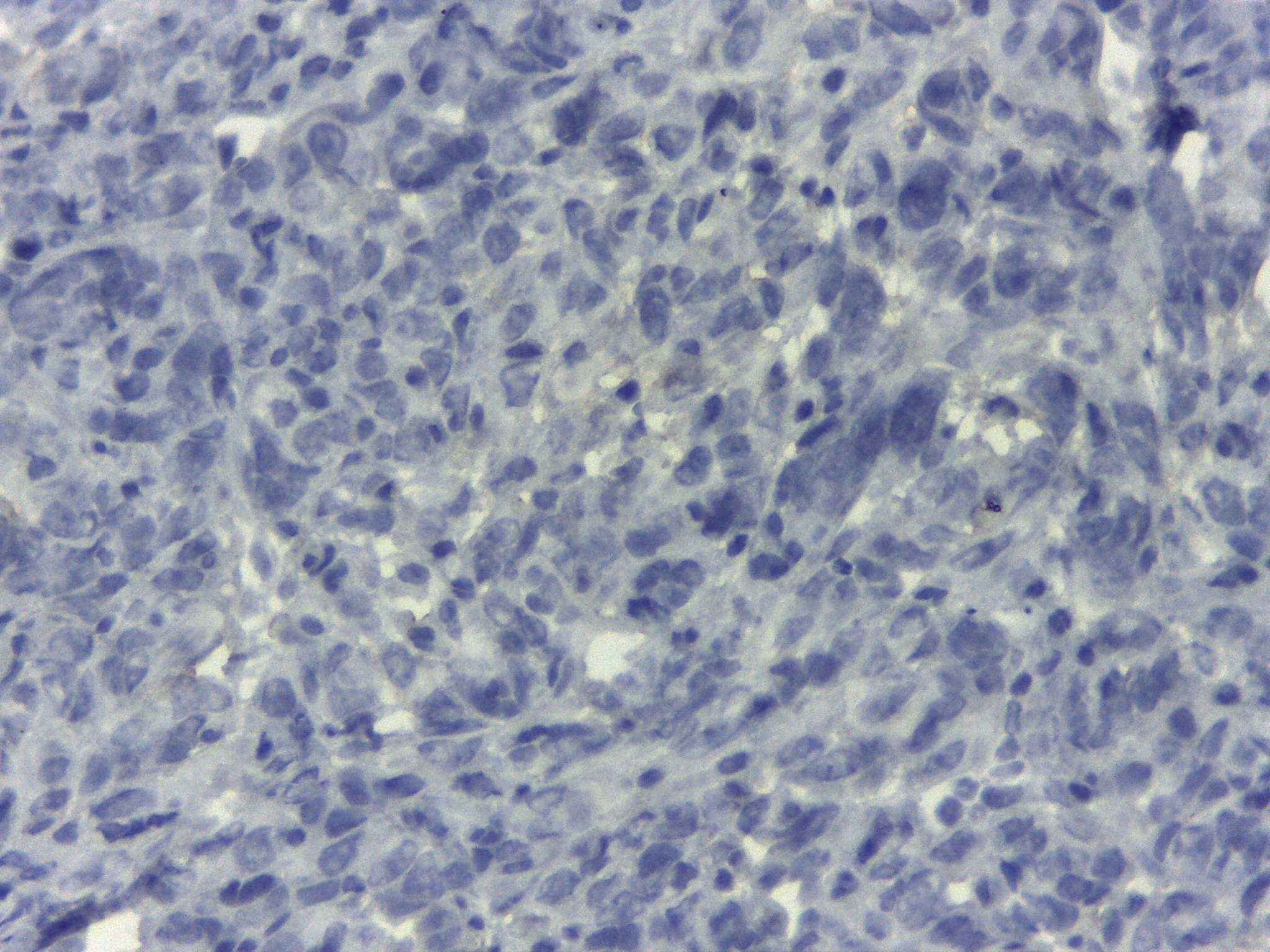

Supplement: S5 Fig — (ZIP) [file pone.0188960.s018.zip › Ca IX IHC image BAC/Ca IX bac1-3.jpg]

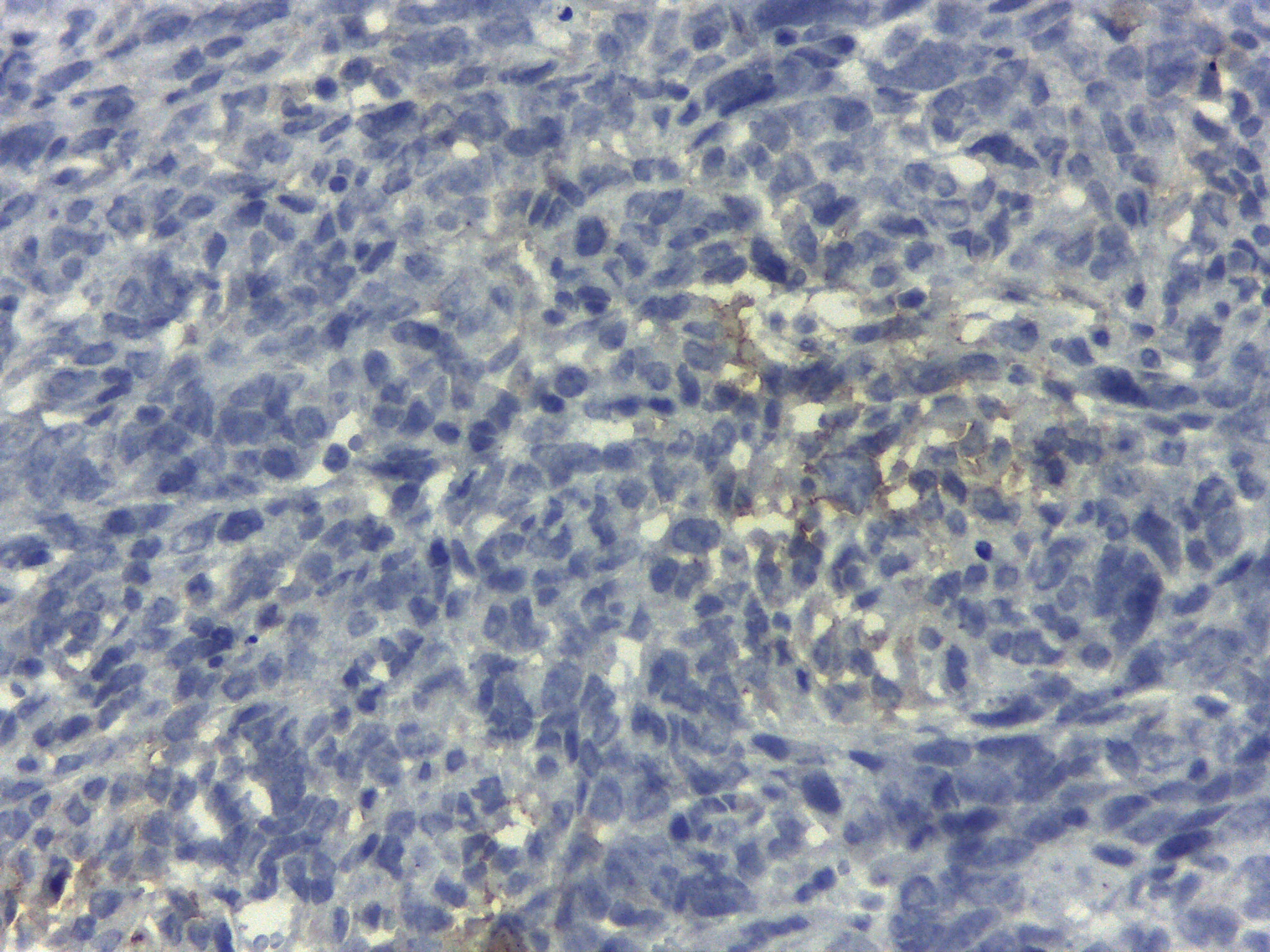

Supplement: S5 Fig — (ZIP) [file pone.0188960.s018.zip › Ca IX IHC image BAC/Ca IX bac1-4.jpg]

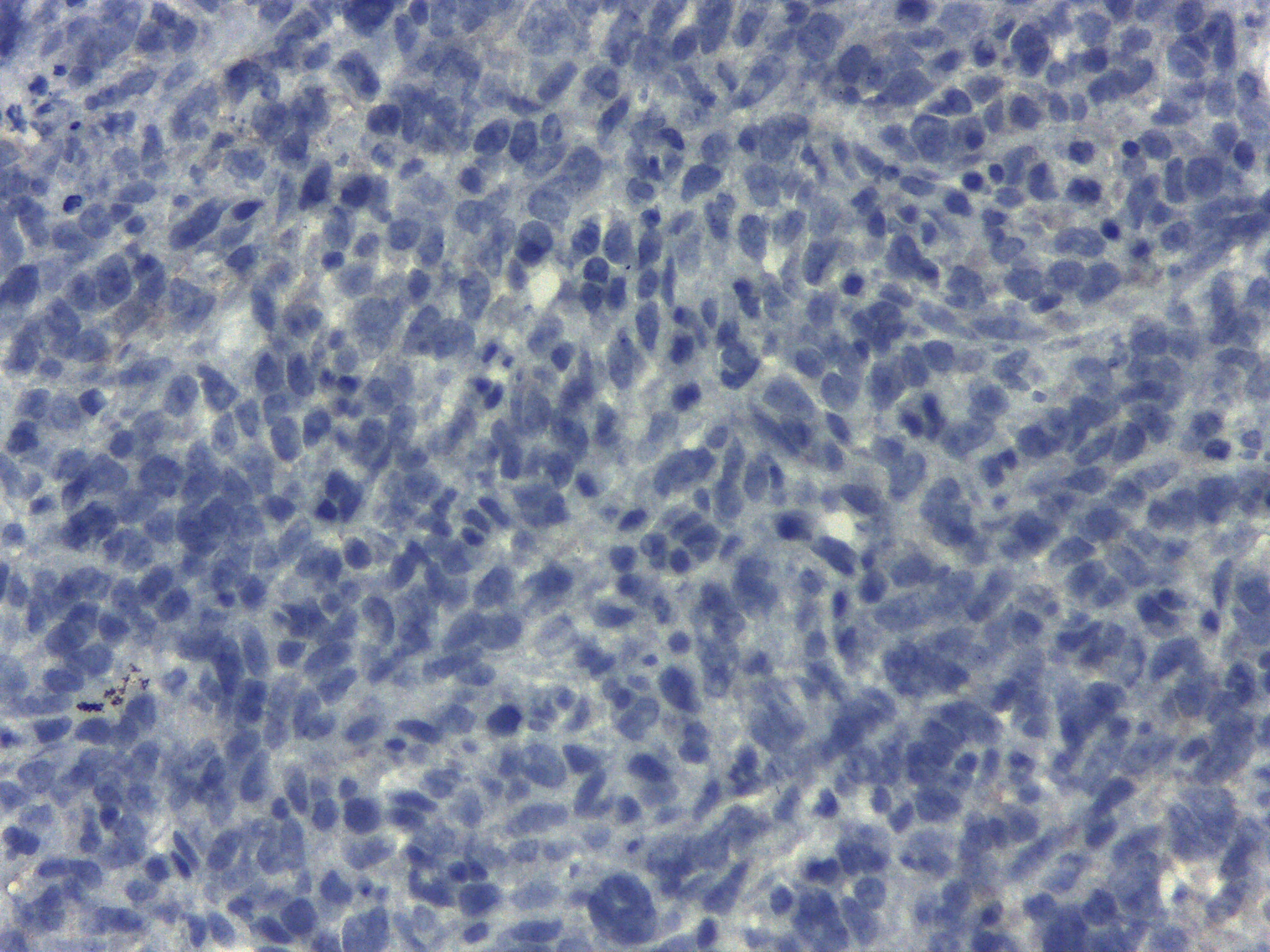

Supplement: S5 Fig — (ZIP) [file pone.0188960.s018.zip › Ca IX IHC image BAC/Ca IX bac1-5.jpg]

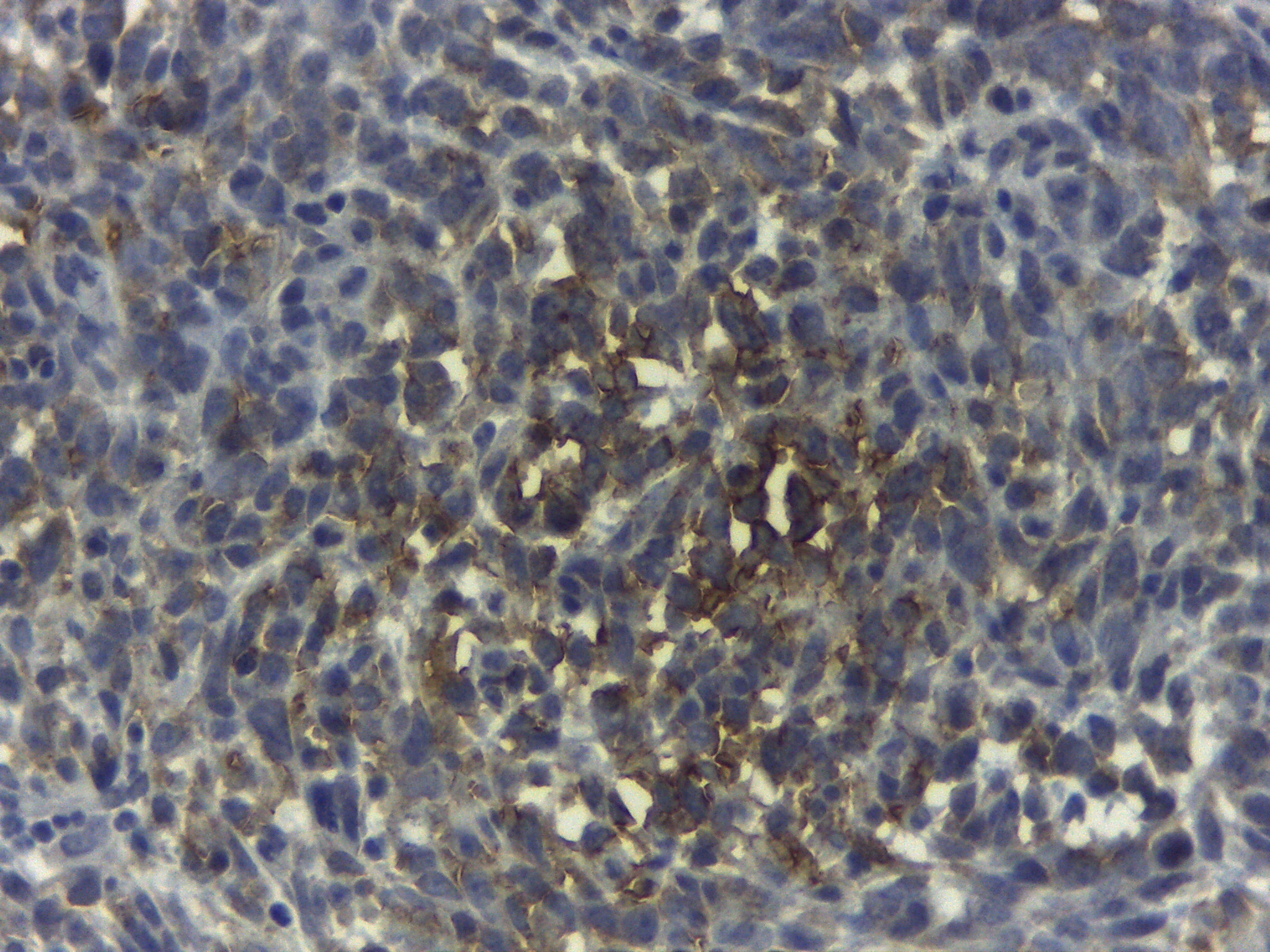

Supplement: S5 Fig — (ZIP) [file pone.0188960.s018.zip › Ca IX IHC image BAC/Ca IX bac2-1.jpg]

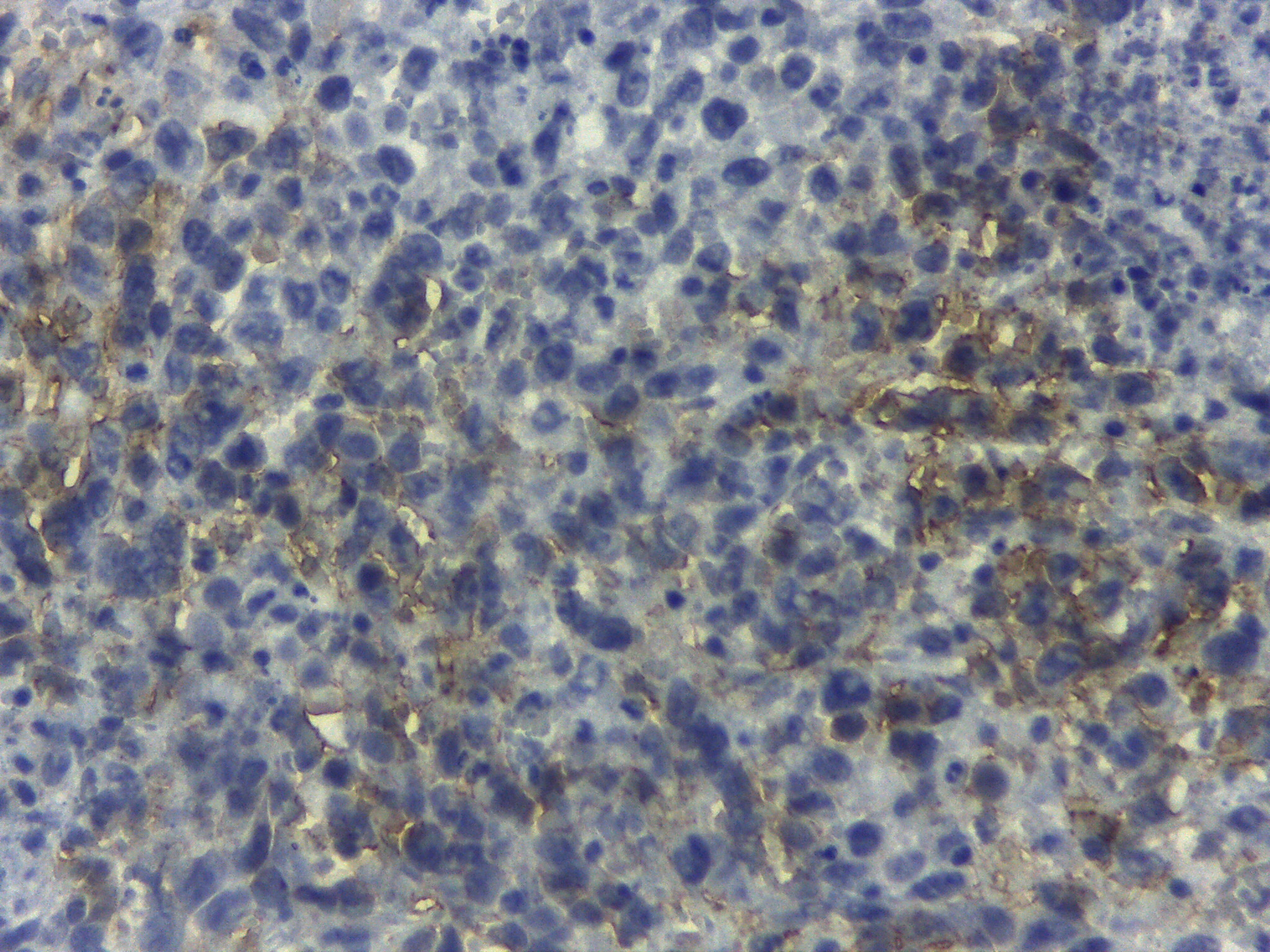

Supplement: S5 Fig — (ZIP) [file pone.0188960.s018.zip › Ca IX IHC image BAC/Ca IX bac2-2.jpg]

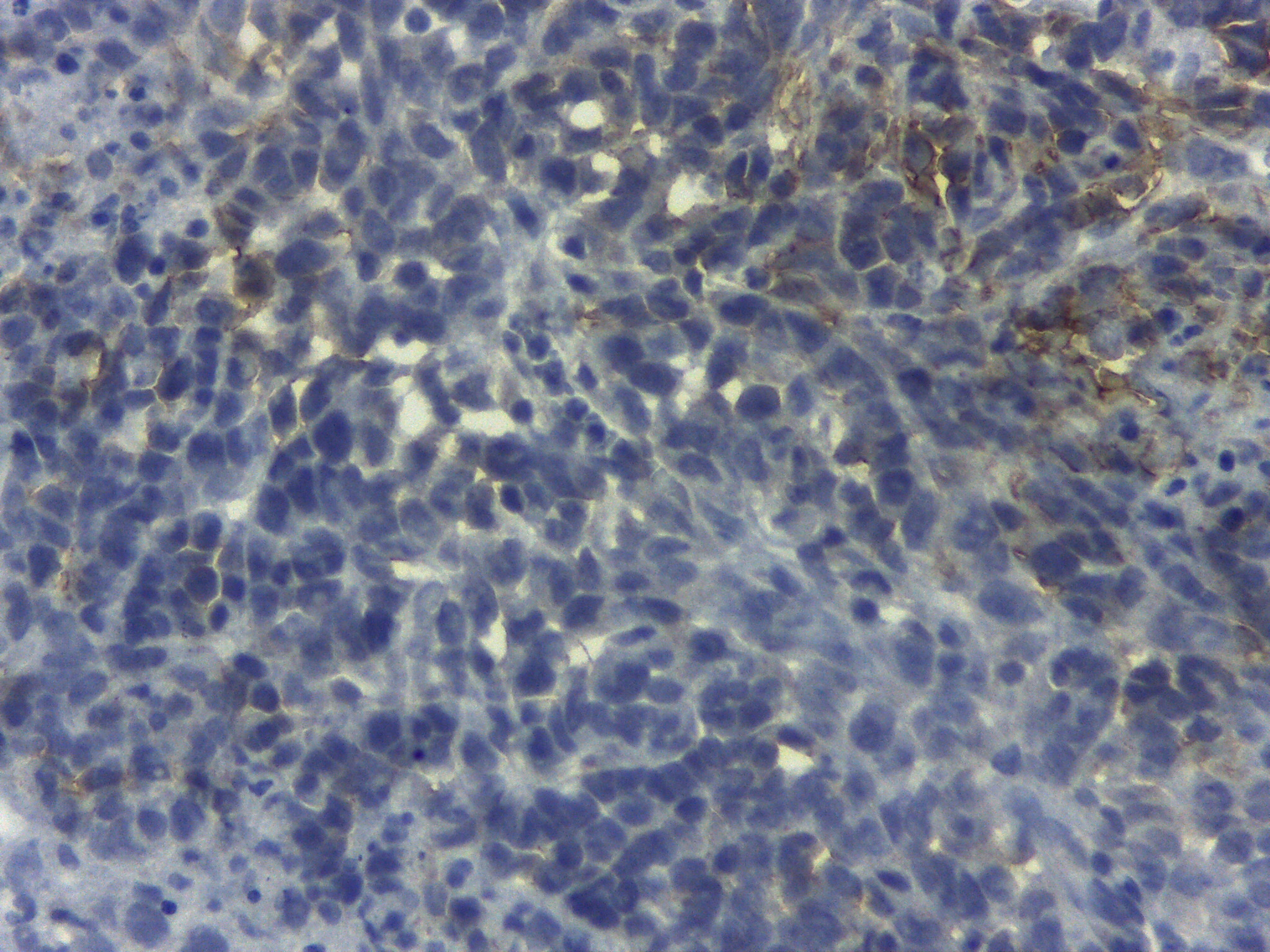

Supplement: S5 Fig — (ZIP) [file pone.0188960.s018.zip › Ca IX IHC image BAC/Ca IX bac2-3.jpg]
