# Supplementary material for: Proteus mirabilis inhibits cancer growth and pulmonary metastasis in a mouse breast cancer model
Source: PLoS One. 2017 Dec 5;12(12):e0188960. doi: 10.1371/journal.pone.0188960 (PMC5716547; doi:10.1371/journal.pone.0188960)
Supplement: S1 Table — (DOCX) [file pone.0188960.s013.docx]

Table S1: 22 mouse cytokines detected by Mouse Cytokine Antibody Array C1

| **1** | **2** | **3** | **4** | **5** | **6** | **7** | **8** |
| --- | --- | --- | --- | --- | --- | --- | --- |
| GCSF | GM-CSF | IL-2 | IL-3 | IL-4 | IL-5 | IL-6 | IL-9 |
| **9** | **10** | **11** | **12** | **13** | **14** | **15** | **16** |
| IL-10 | IL-12  P40/p70 | IL-12 p70 | IL-13 | IL-17A | IFN-gamma | MCP-1  (CCL2) | MCP-5 |
| **17** | **18** | **19** | **20** | **21** | **22** |  |  |
| RANTES  (CCL5) | SCF | TNF RI  (TNFRSF1A) | TNF alpha | TPO | VEGF-A |  |  |
