# Supplementary figures and images for: Proteus mirabilis inhibits cancer growth and pulmonary metastasis in a mouse breast cancer model (part 2 of 5)
Source: PLoS One. 2017 Dec 5;12(12):e0188960. doi: 10.1371/journal.pone.0188960 (PMC5716547; doi:10.1371/journal.pone.0188960)

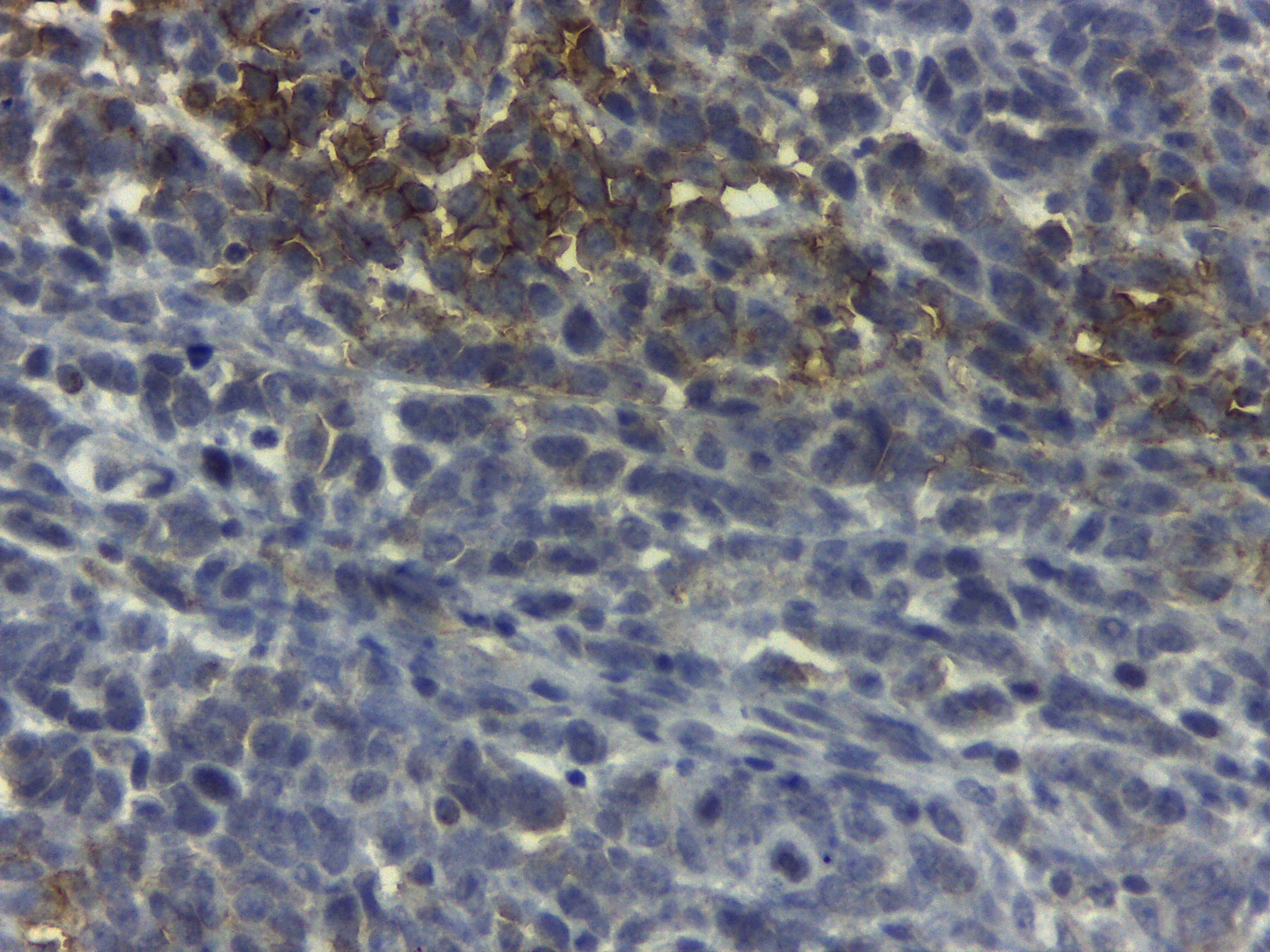

Supplement: S5 Fig — (ZIP) [file pone.0188960.s018.zip › Ca IX IHC image BAC/Ca IX bac2-4.jpg]

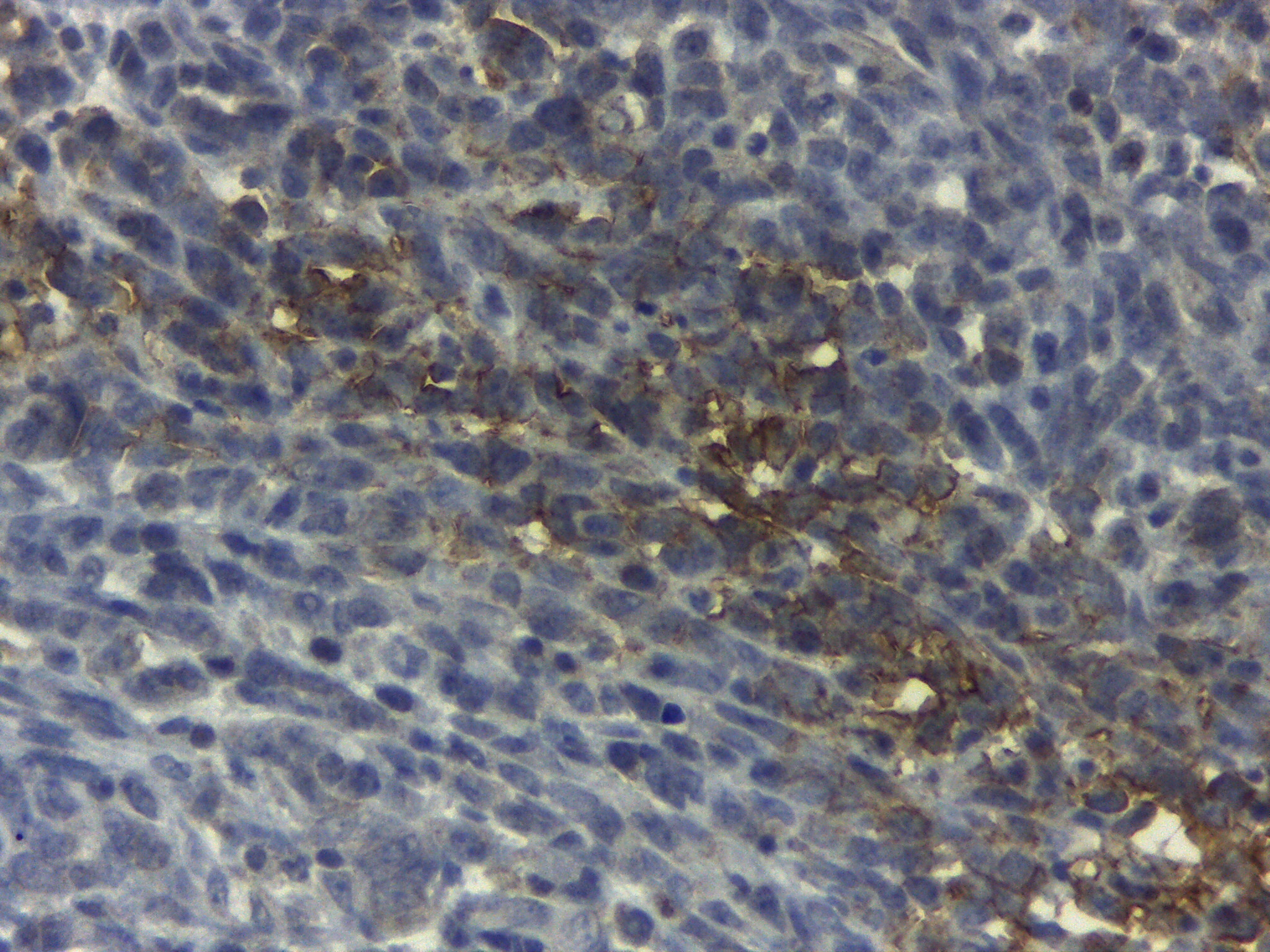

Supplement: S5 Fig — (ZIP) [file pone.0188960.s018.zip › Ca IX IHC image BAC/Ca IX bac2-5.jpg]

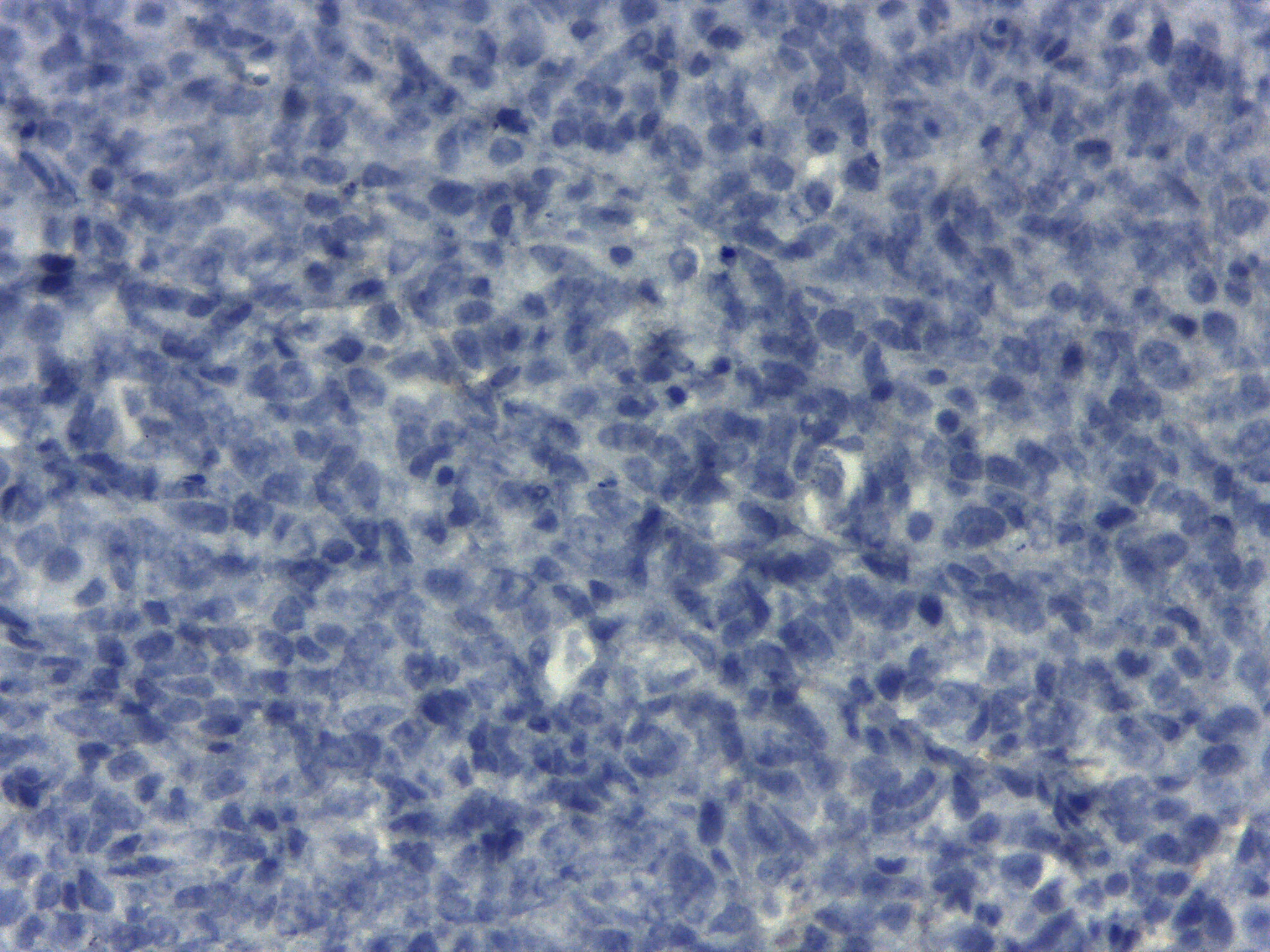

Supplement: S5 Fig — (ZIP) [file pone.0188960.s018.zip › Ca IX IHC image BAC/Ca IX bac3-1.jpg]

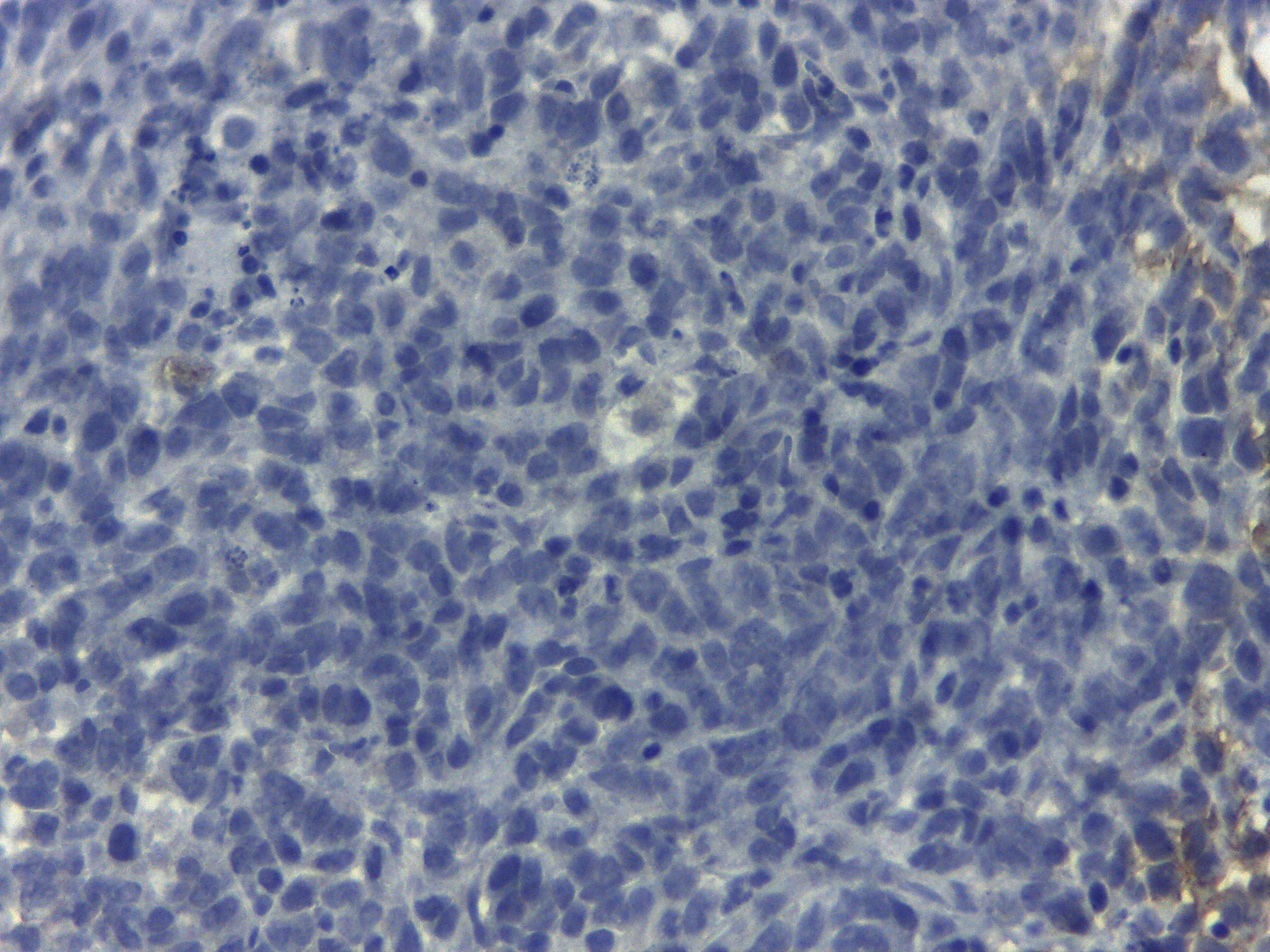

Supplement: S5 Fig — (ZIP) [file pone.0188960.s018.zip › Ca IX IHC image BAC/Ca IX bac3-2.jpg]

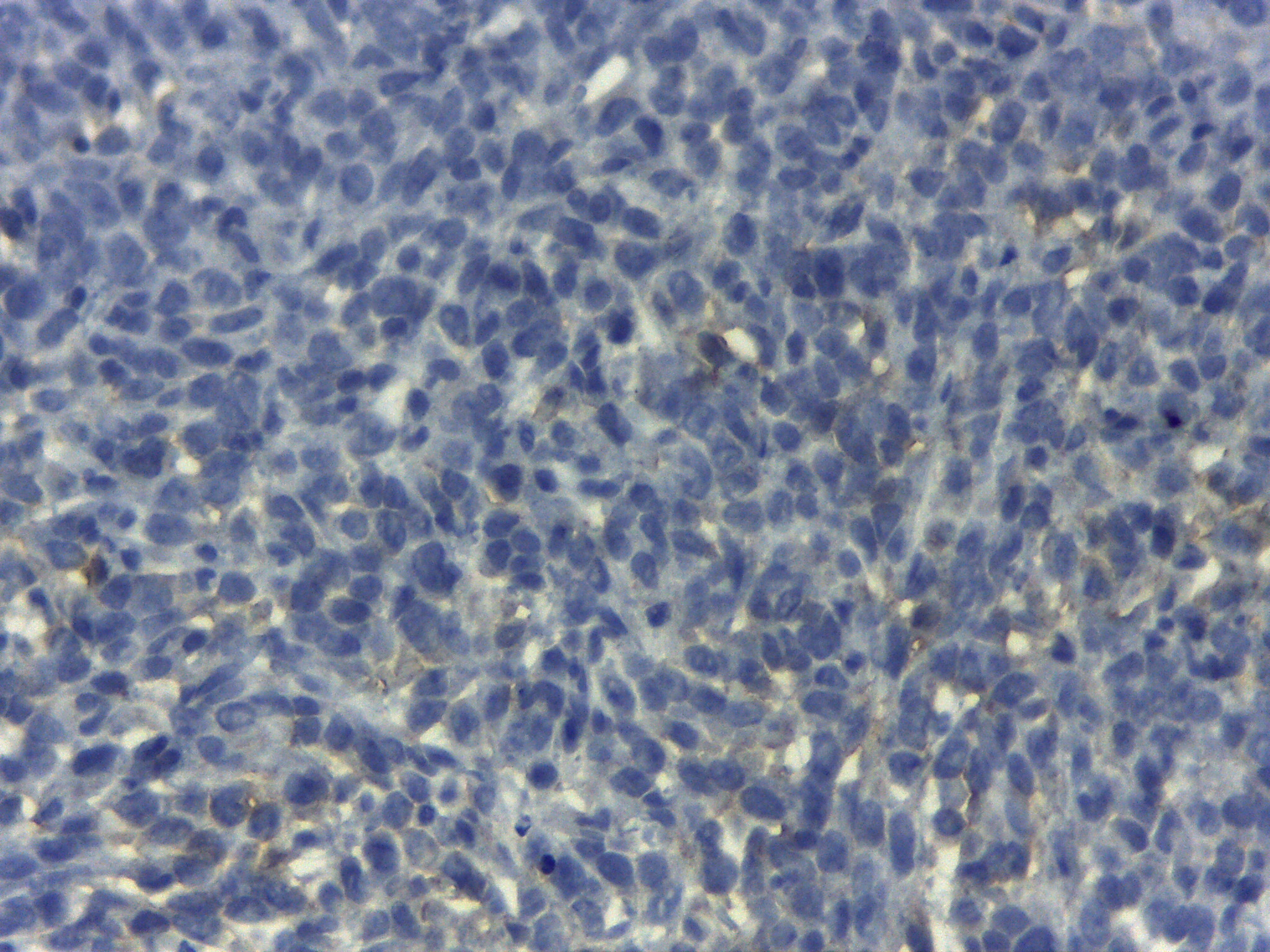

Supplement: S5 Fig — (ZIP) [file pone.0188960.s018.zip › Ca IX IHC image BAC/Ca IX bac3-3.jpg]

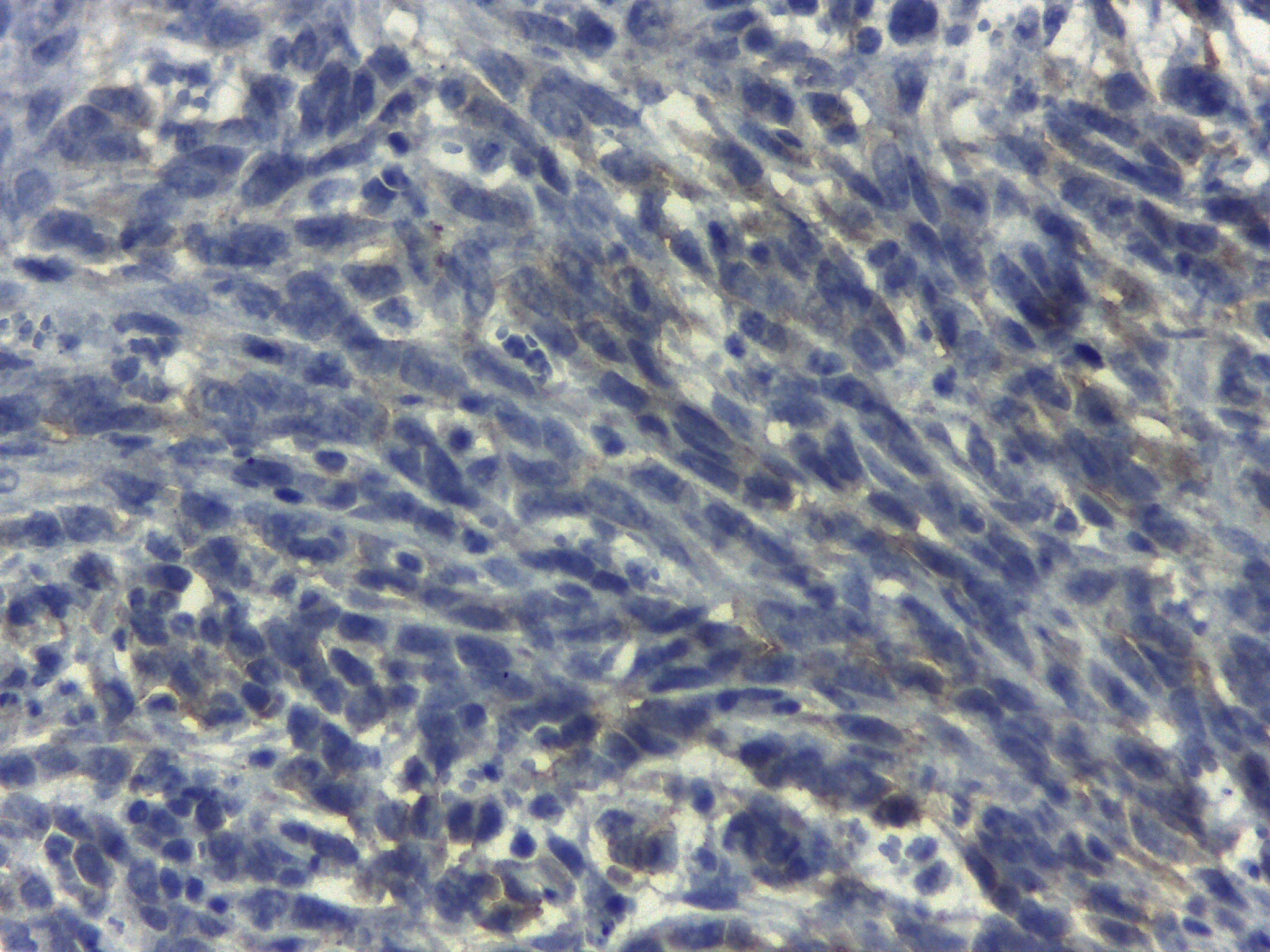

Supplement: S5 Fig — (ZIP) [file pone.0188960.s018.zip › Ca IX IHC image BAC/Ca IX bac3-4.jpg]

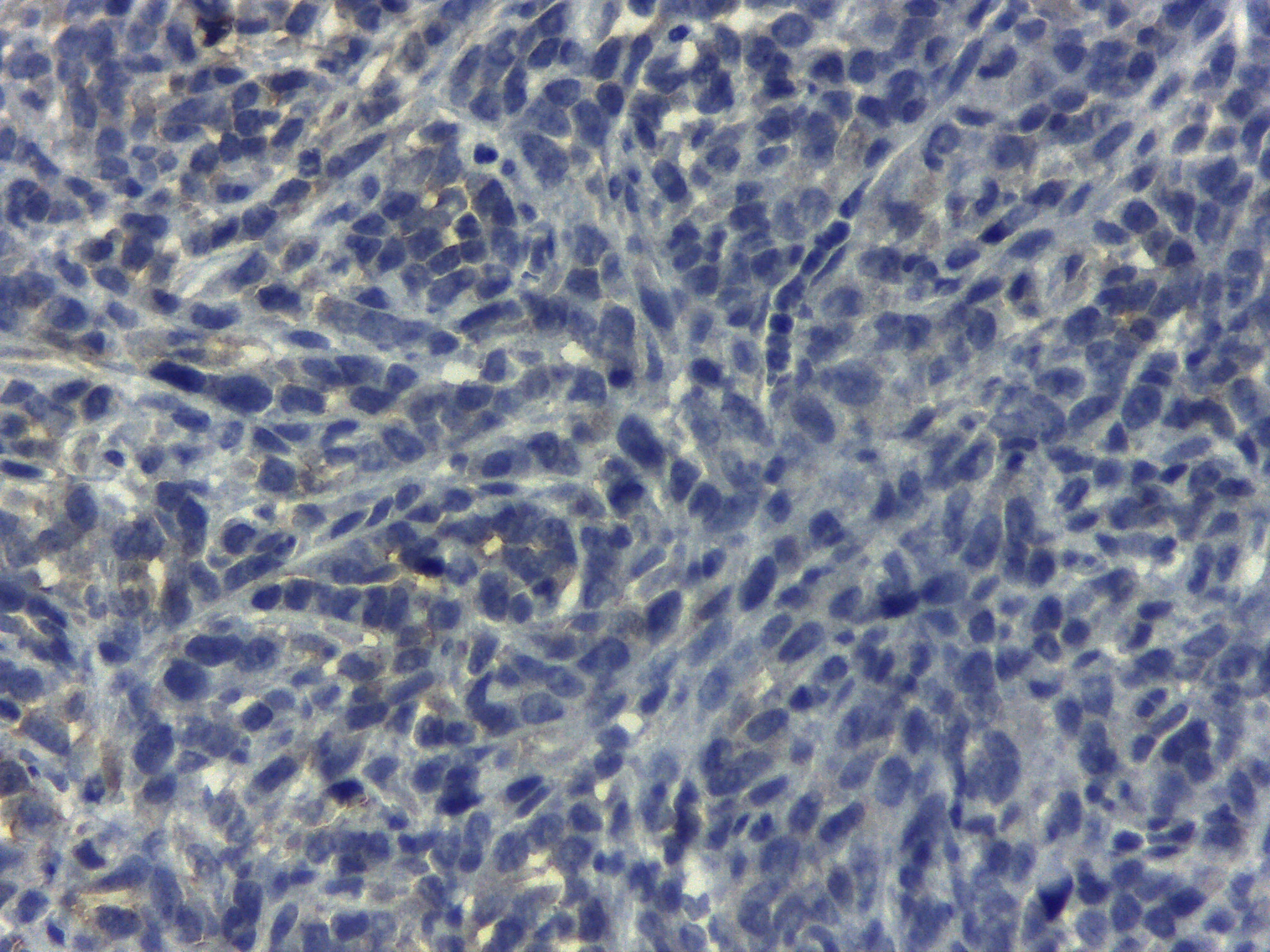

Supplement: S5 Fig — (ZIP) [file pone.0188960.s018.zip › Ca IX IHC image BAC/Ca IX bac3-5.jpg]

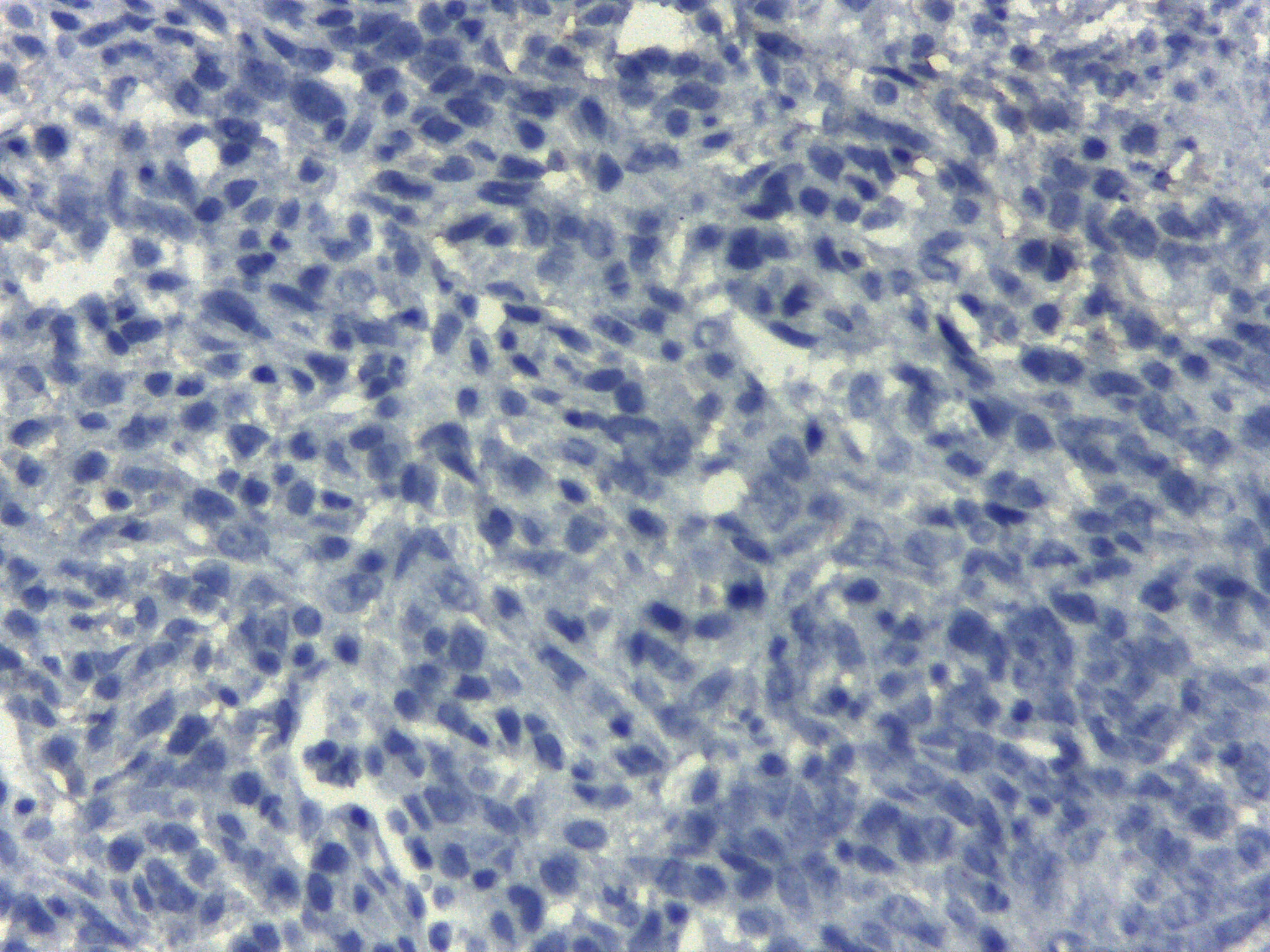

Supplement: S5 Fig — (ZIP) [file pone.0188960.s018.zip › Ca IX IHC image BAC/Ca IX bac4-1.jpg]

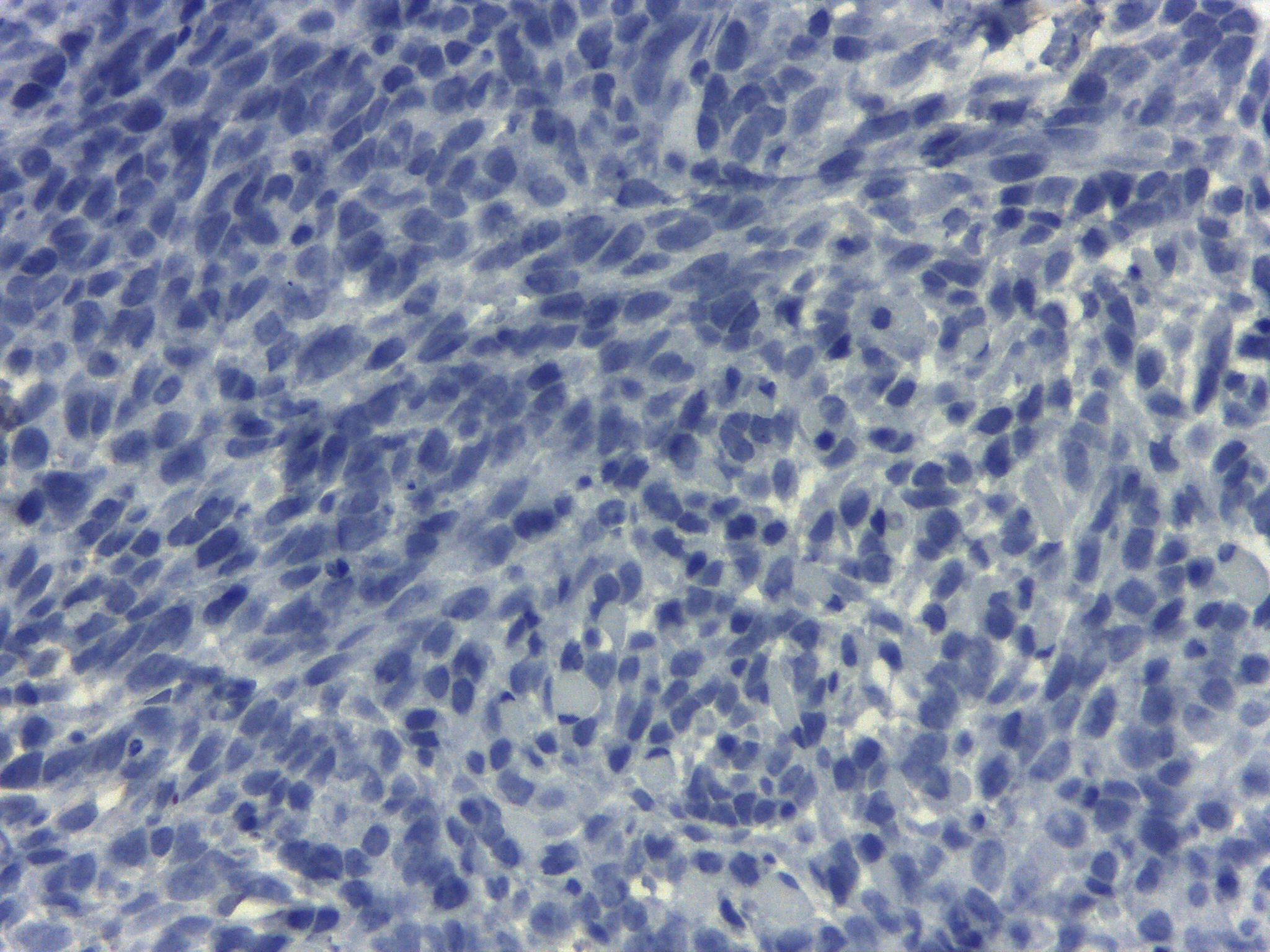

Supplement: S5 Fig — (ZIP) [file pone.0188960.s018.zip › Ca IX IHC image BAC/Ca IX bac4-2.jpg]

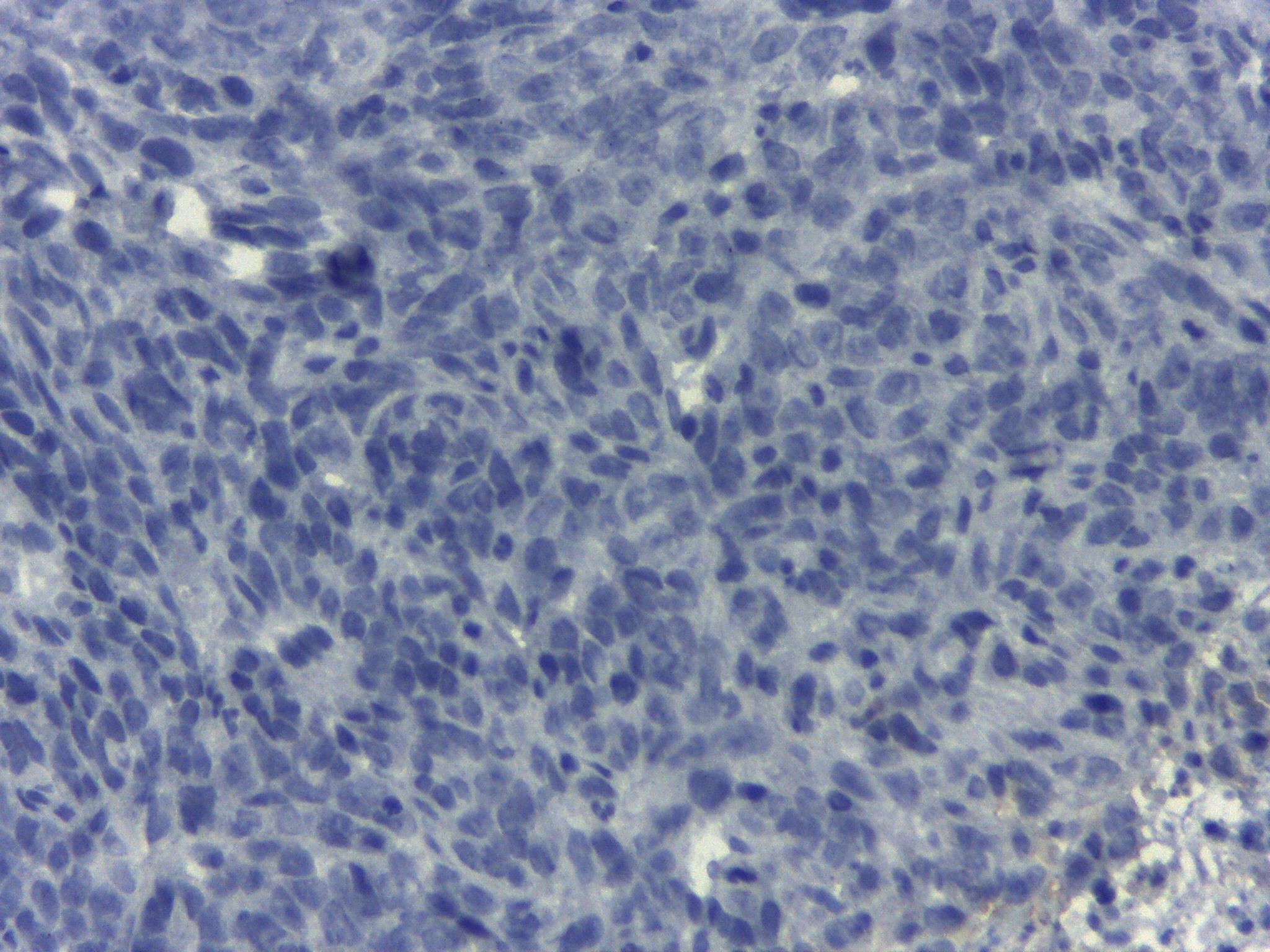

Supplement: S5 Fig — (ZIP) [file pone.0188960.s018.zip › Ca IX IHC image BAC/Ca IX bac4-3.jpg]

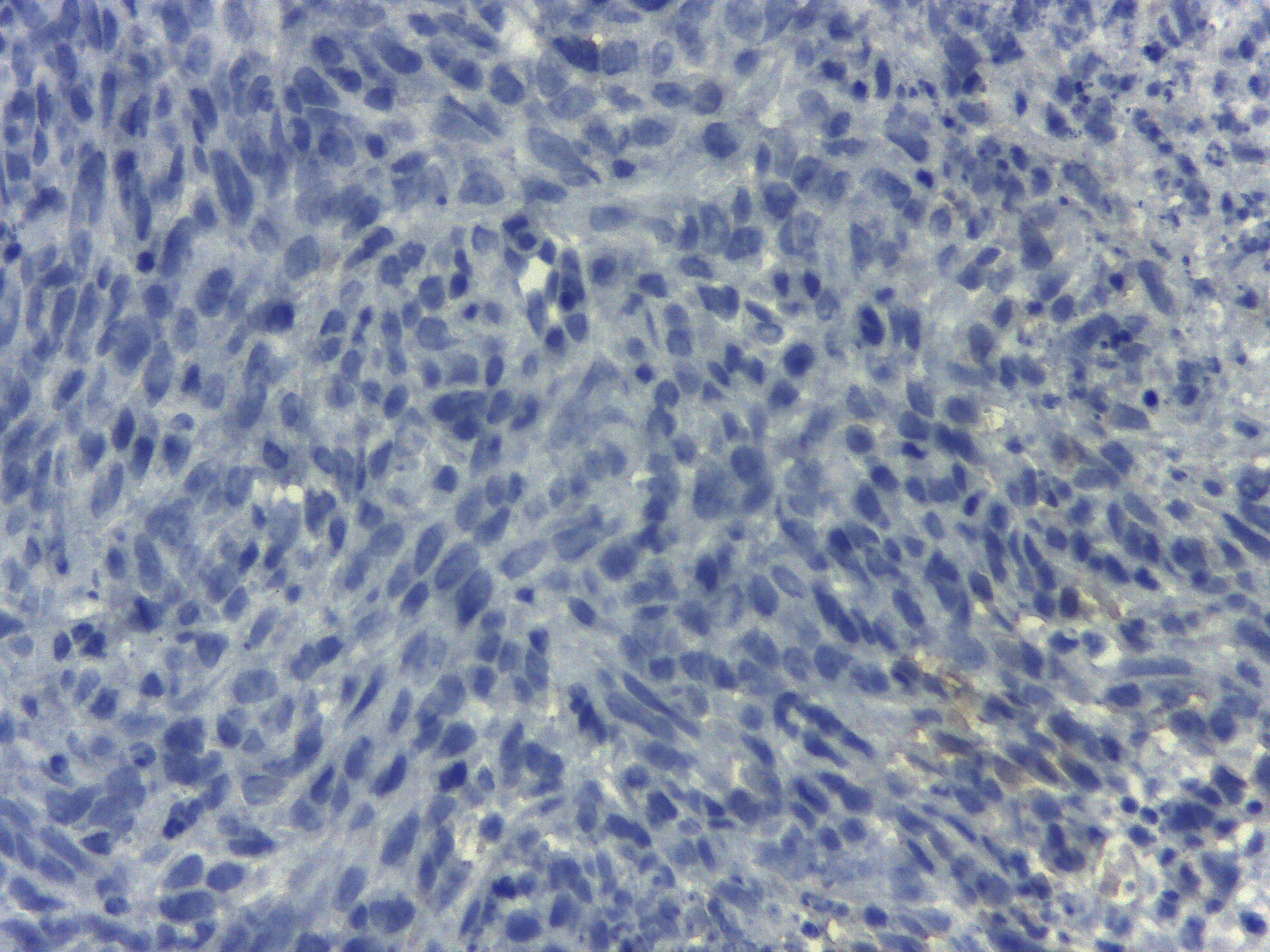

Supplement: S5 Fig — (ZIP) [file pone.0188960.s018.zip › Ca IX IHC image BAC/Ca IX bac4-4.jpg]

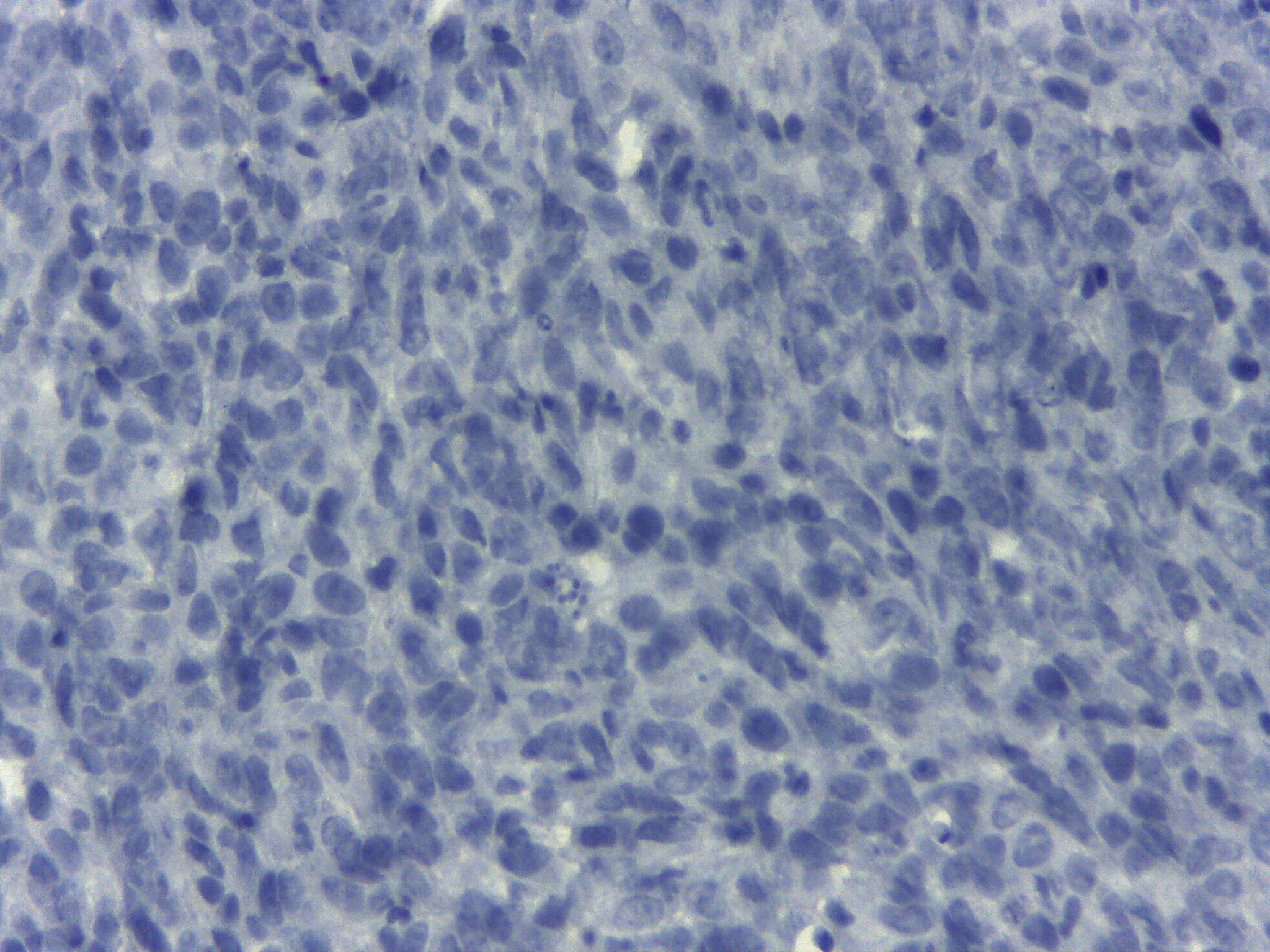

Supplement: S5 Fig — (ZIP) [file pone.0188960.s018.zip › Ca IX IHC image BAC/Ca IX bac4-5.jpg]

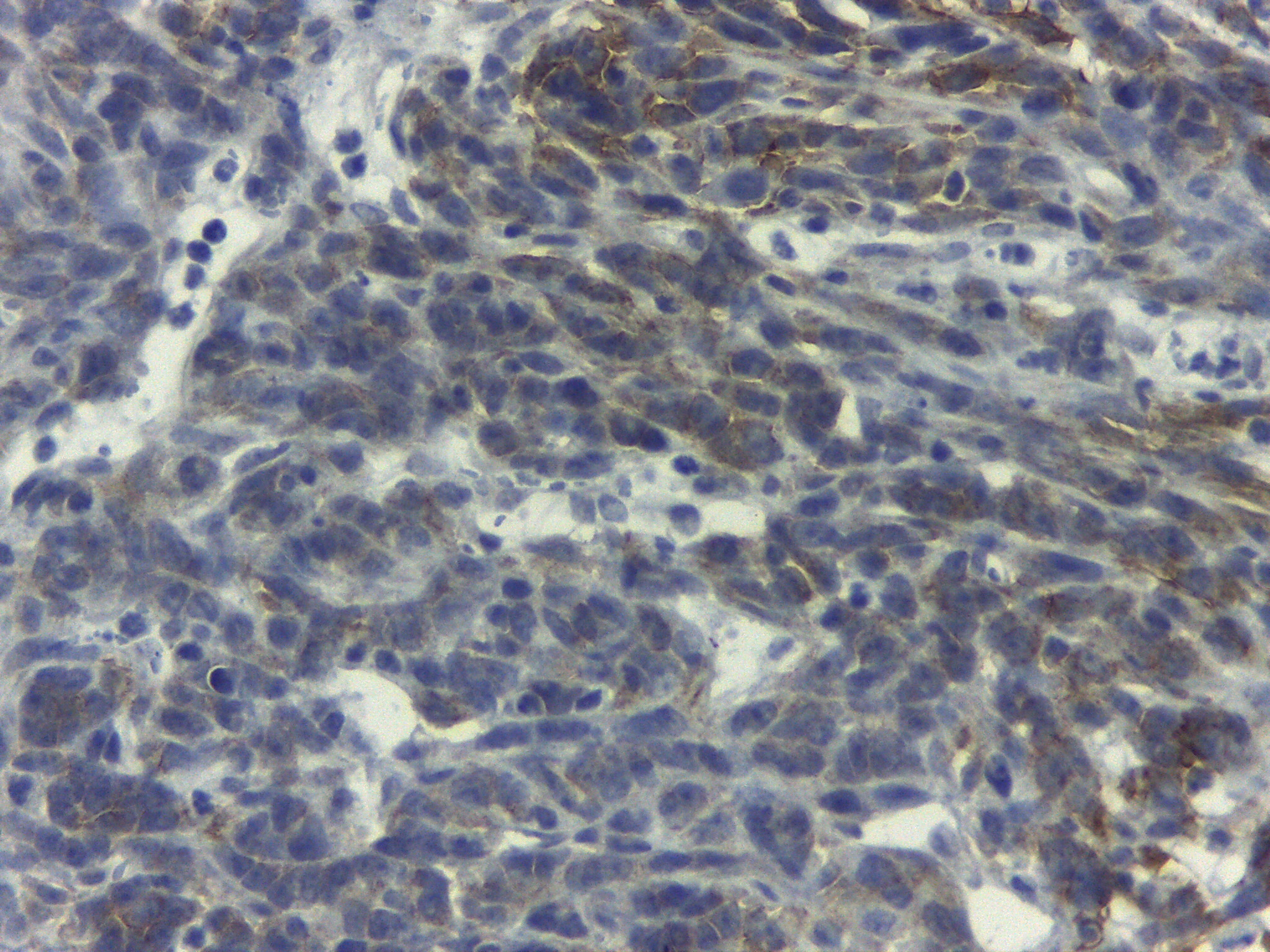

Supplement: S5 Fig — (ZIP) [file pone.0188960.s018.zip › Ca IX IHC image BAC/Ca IX bac5-1.jpg]

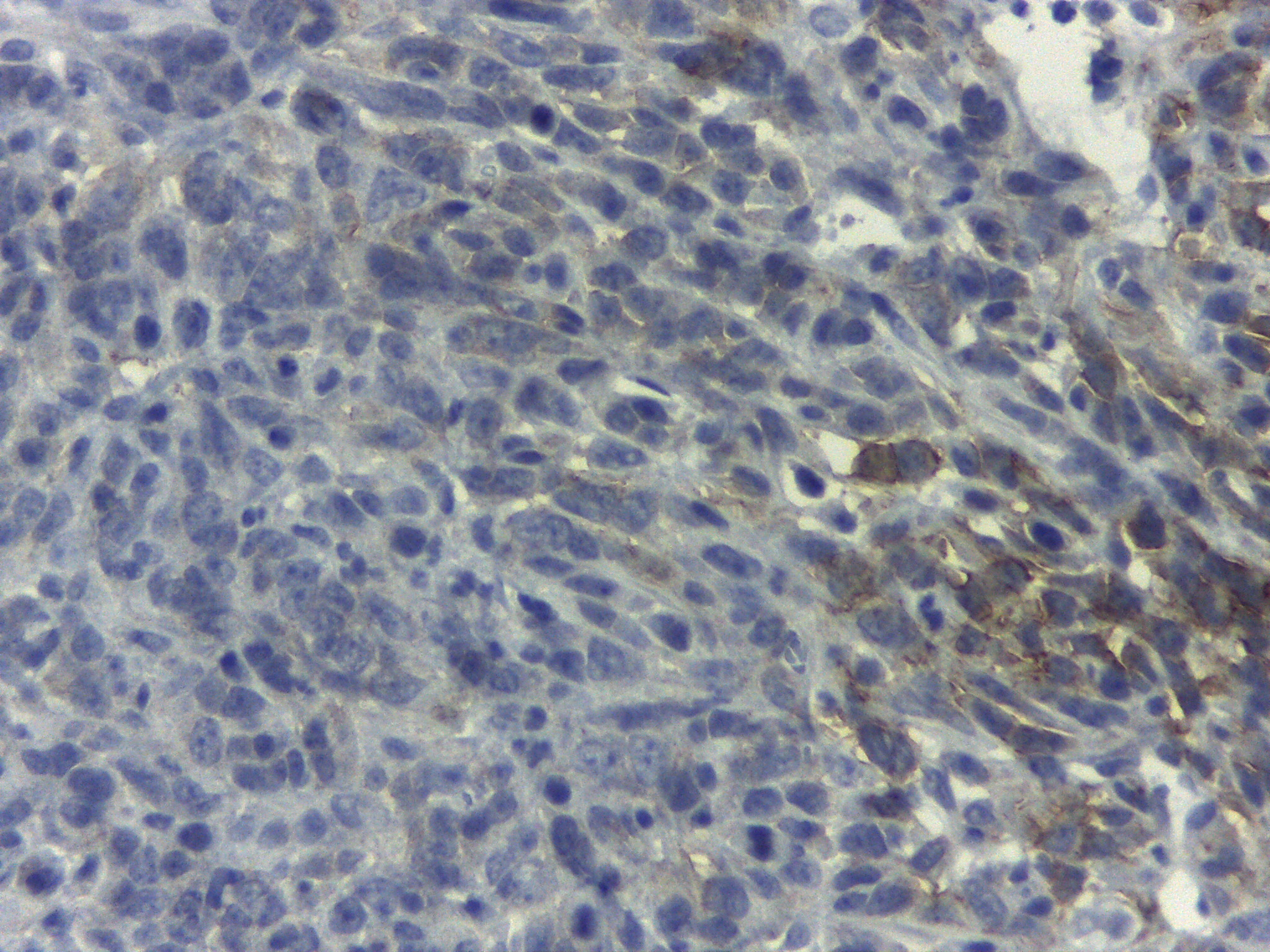

Supplement: S5 Fig — (ZIP) [file pone.0188960.s018.zip › Ca IX IHC image BAC/Ca IX bac5-2.jpg]

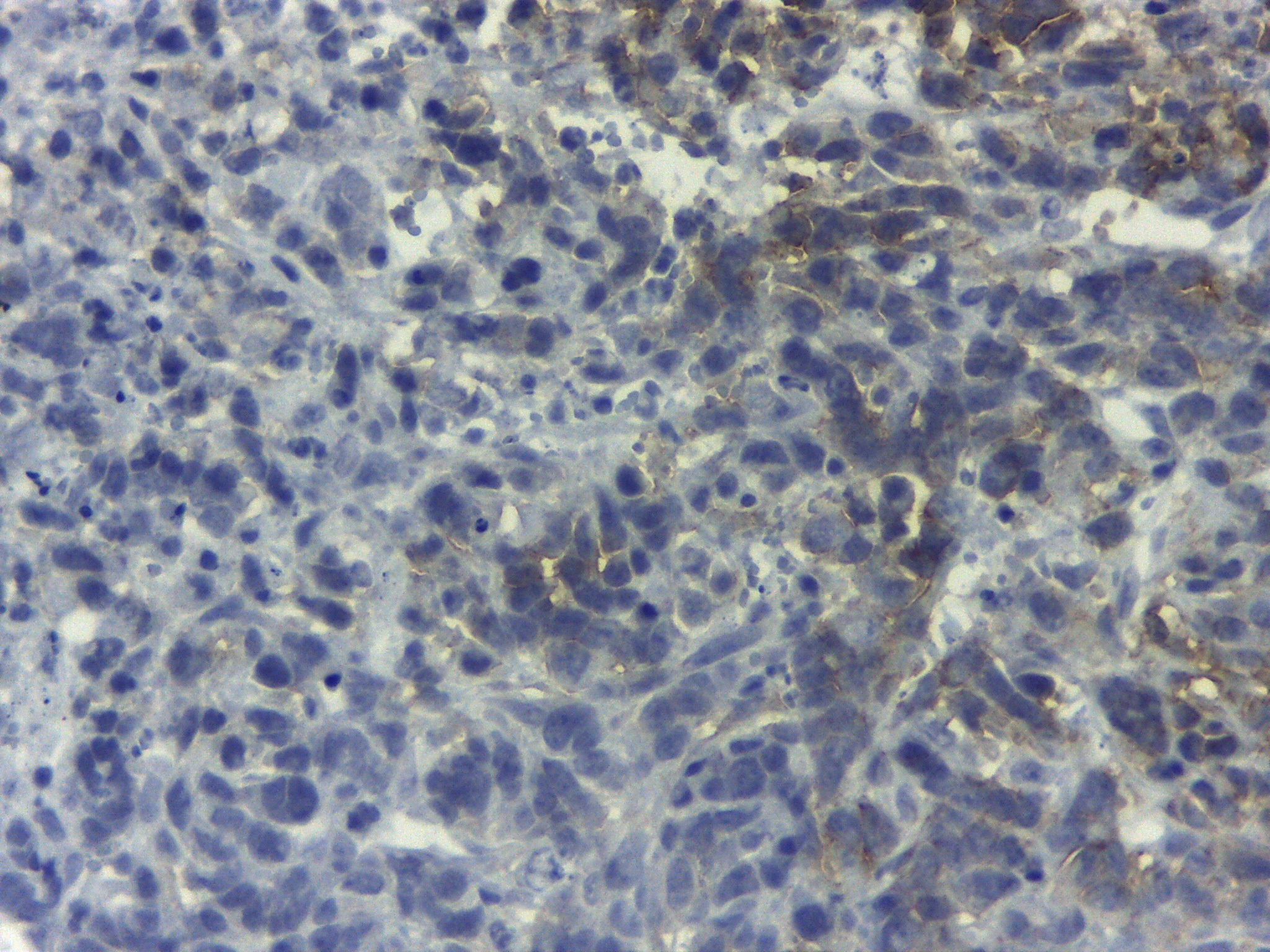

Supplement: S5 Fig — (ZIP) [file pone.0188960.s018.zip › Ca IX IHC image BAC/Ca IX bac5-3.jpg]

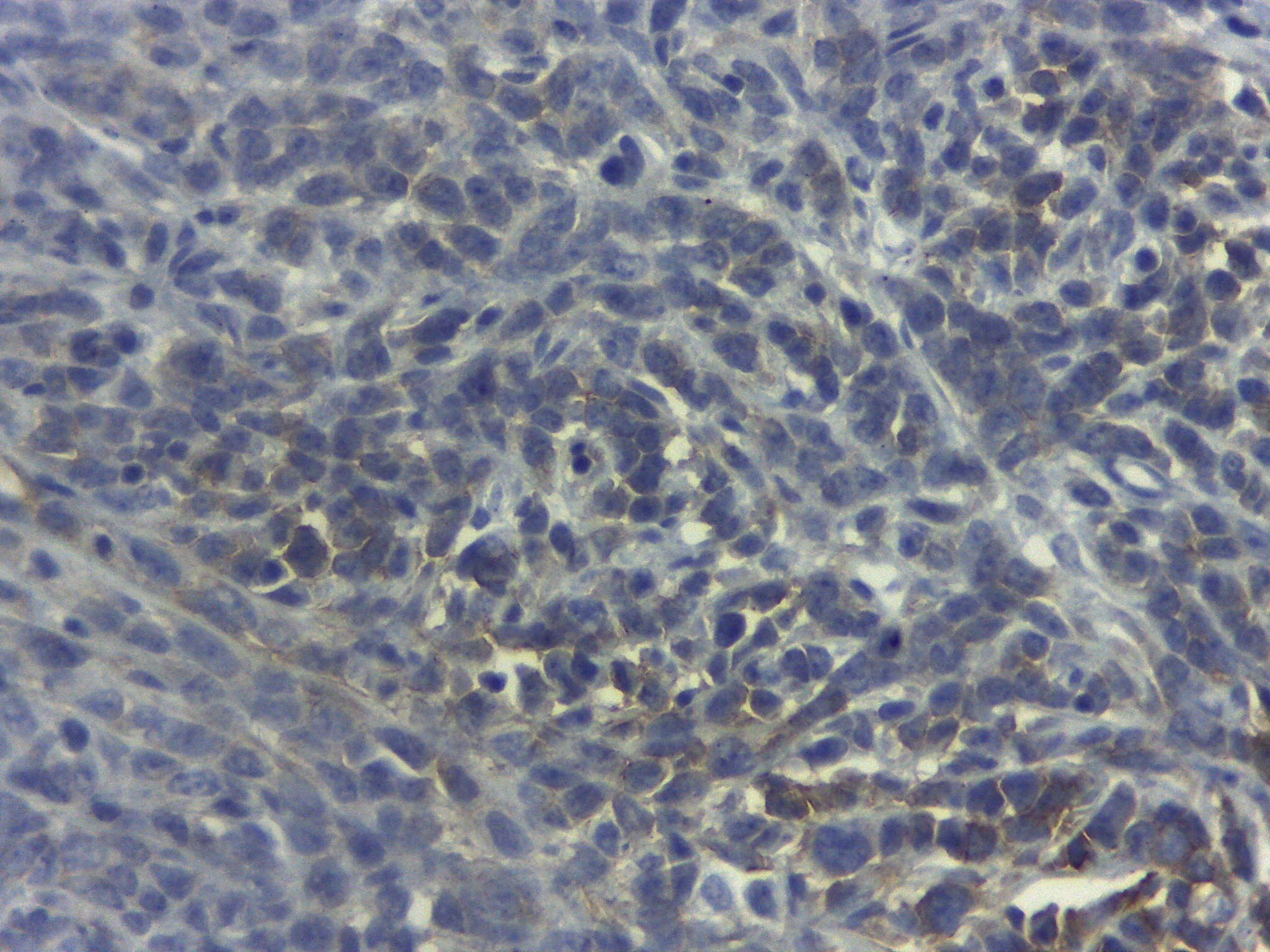

Supplement: S5 Fig — (ZIP) [file pone.0188960.s018.zip › Ca IX IHC image BAC/Ca IX bac5-4.jpg]

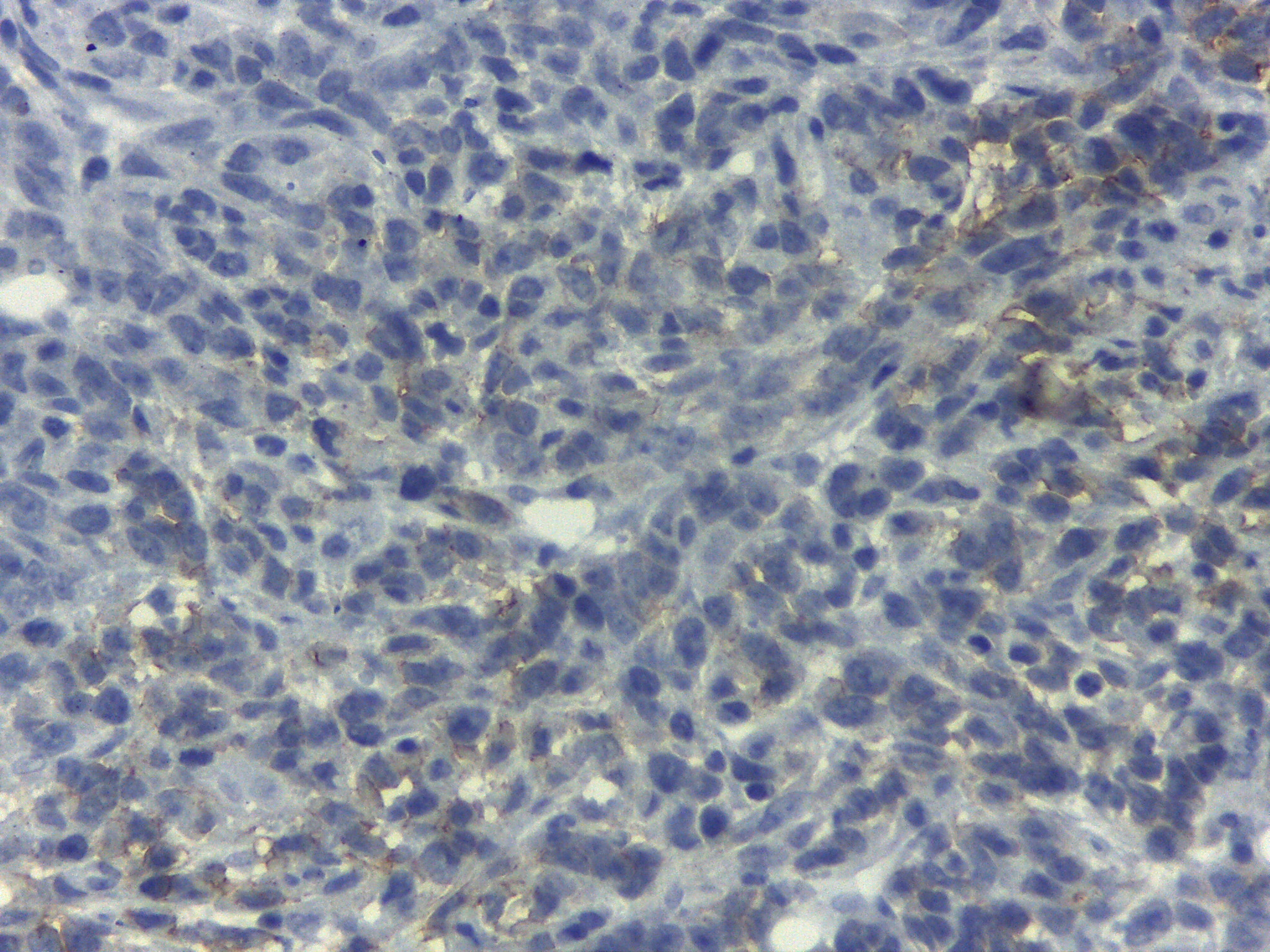

Supplement: S5 Fig — (ZIP) [file pone.0188960.s018.zip › Ca IX IHC image BAC/Ca IX bac5-5.jpg]

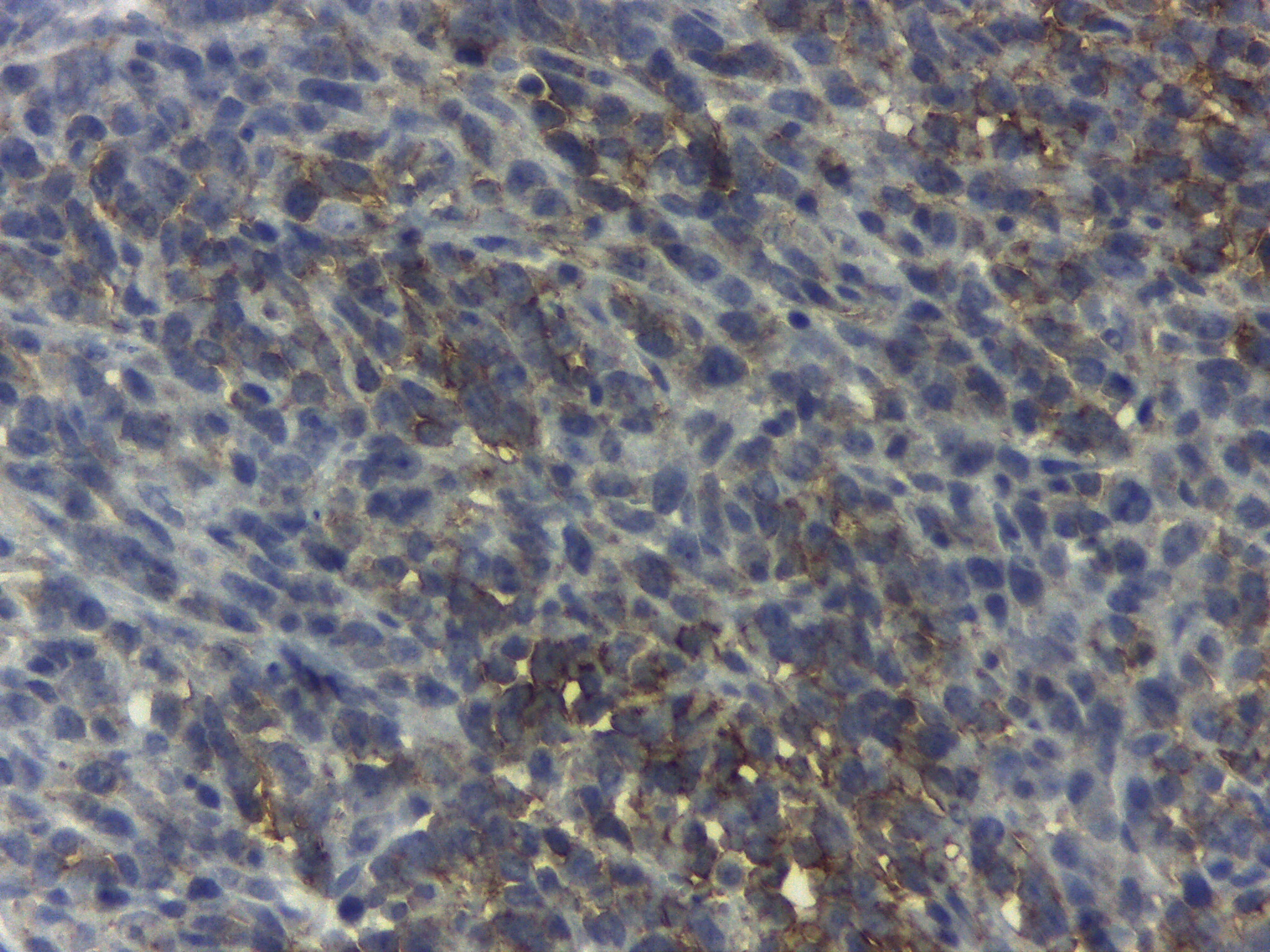

Supplement: S5 Fig — (ZIP) [file pone.0188960.s018.zip › Ca IX IHC image BAC/Ca IX bac6-1.jpg]

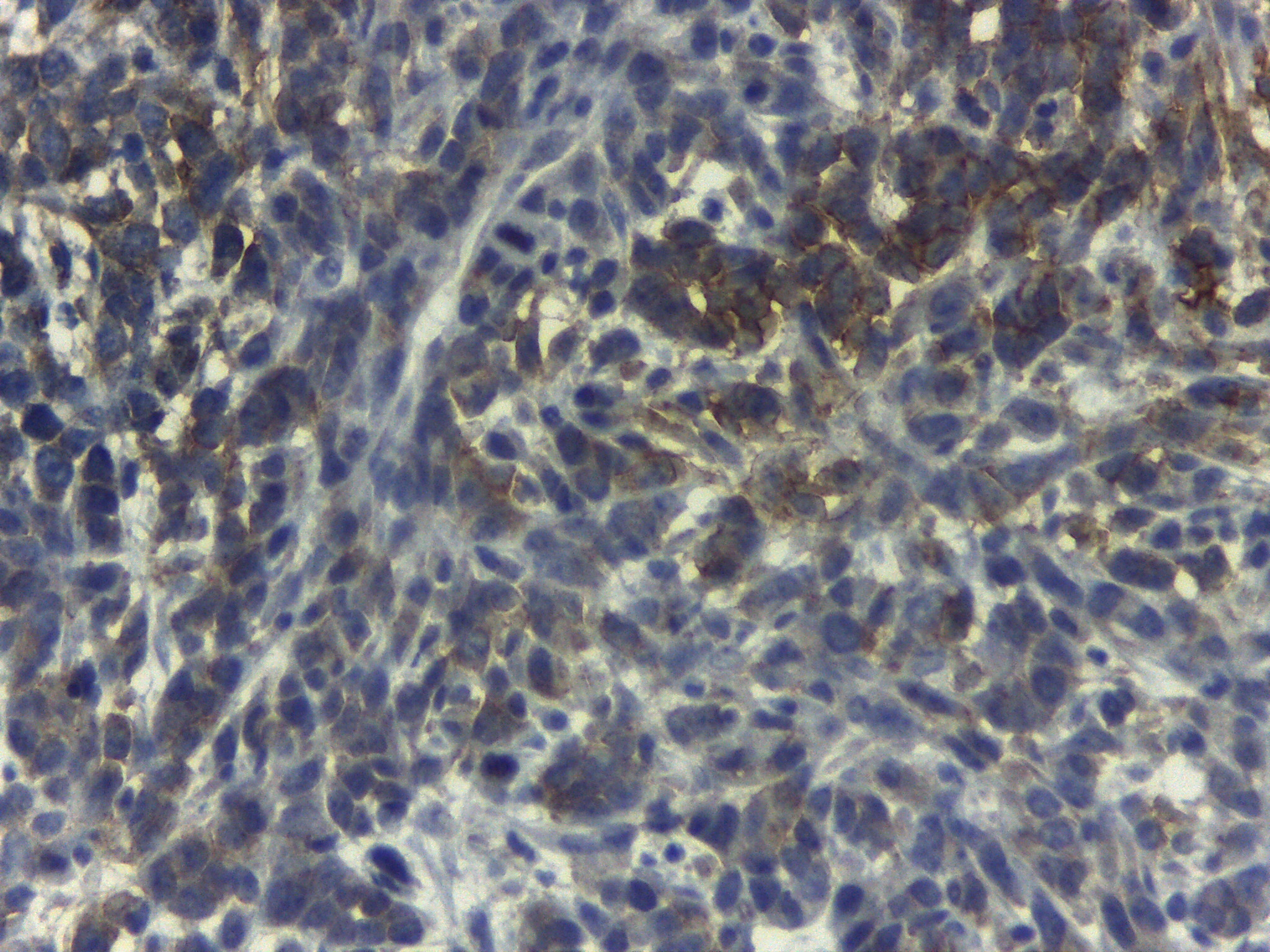

Supplement: S5 Fig — (ZIP) [file pone.0188960.s018.zip › Ca IX IHC image BAC/Ca IX bac6-2.jpg]

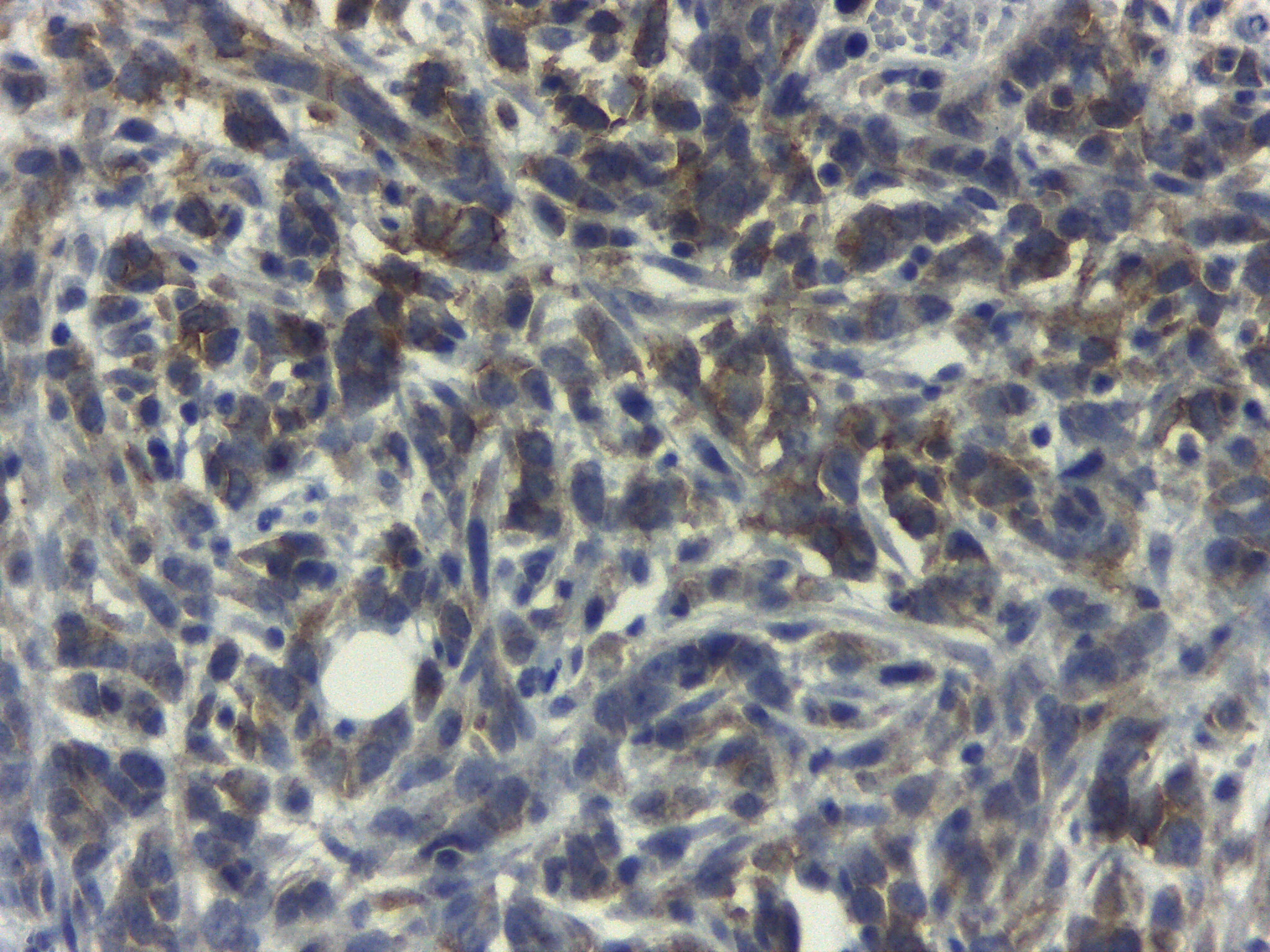

Supplement: S5 Fig — (ZIP) [file pone.0188960.s018.zip › Ca IX IHC image BAC/Ca IX bac6-3.jpg]

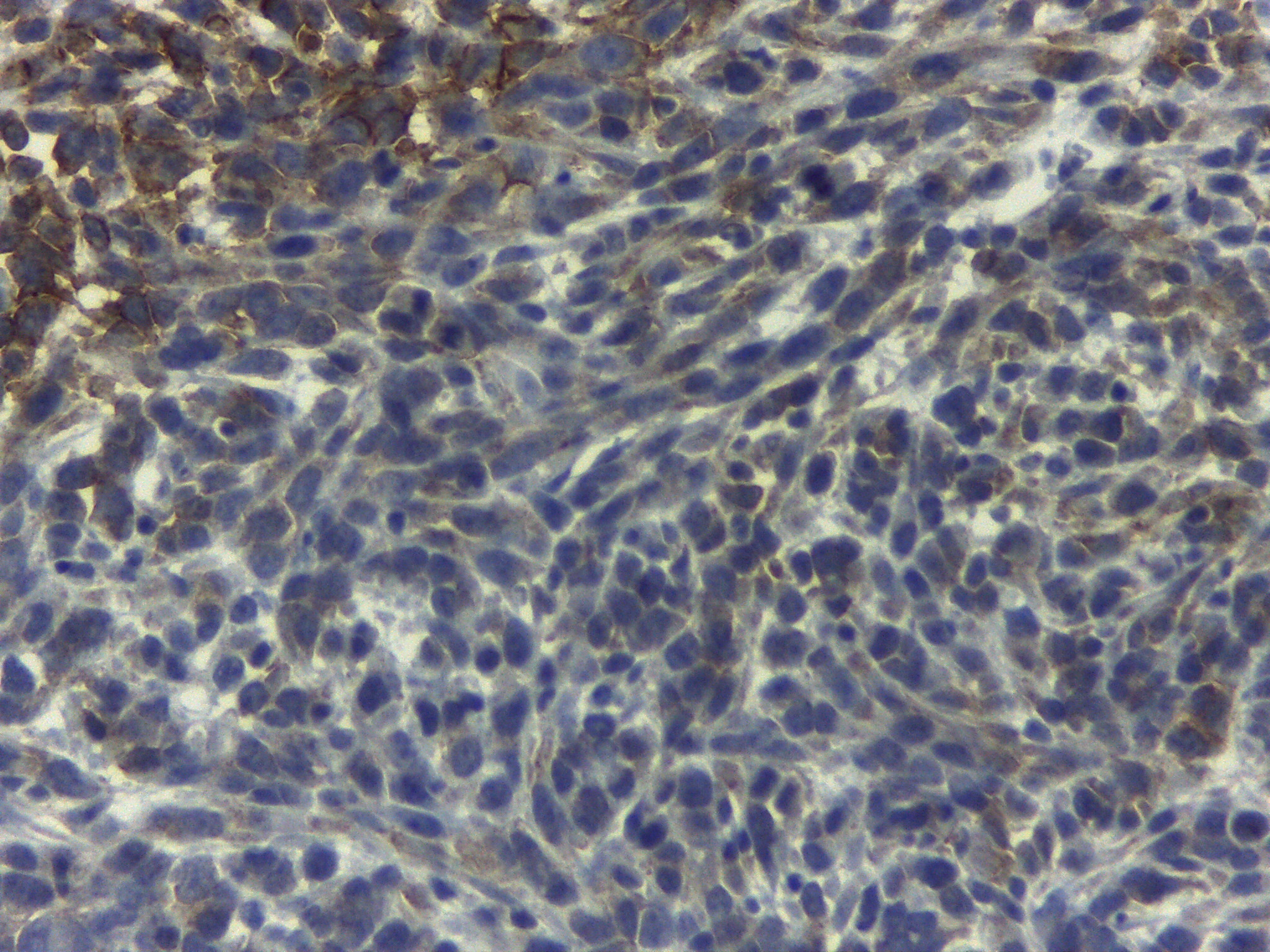

Supplement: S5 Fig — (ZIP) [file pone.0188960.s018.zip › Ca IX IHC image BAC/Ca IX bac6-4.jpg]

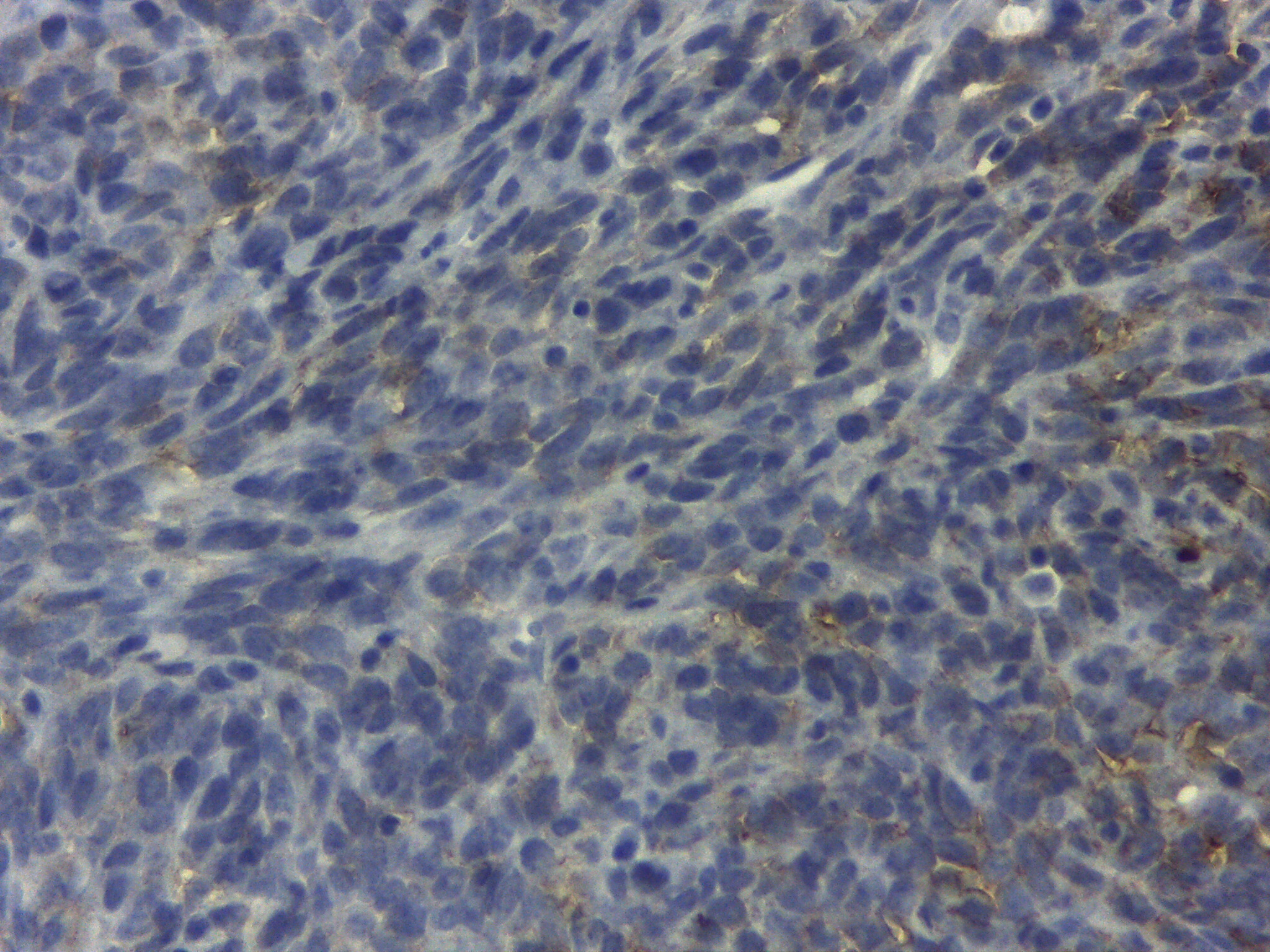

Supplement: S5 Fig — (ZIP) [file pone.0188960.s018.zip › Ca IX IHC image BAC/Ca IX bac6-5.jpg]

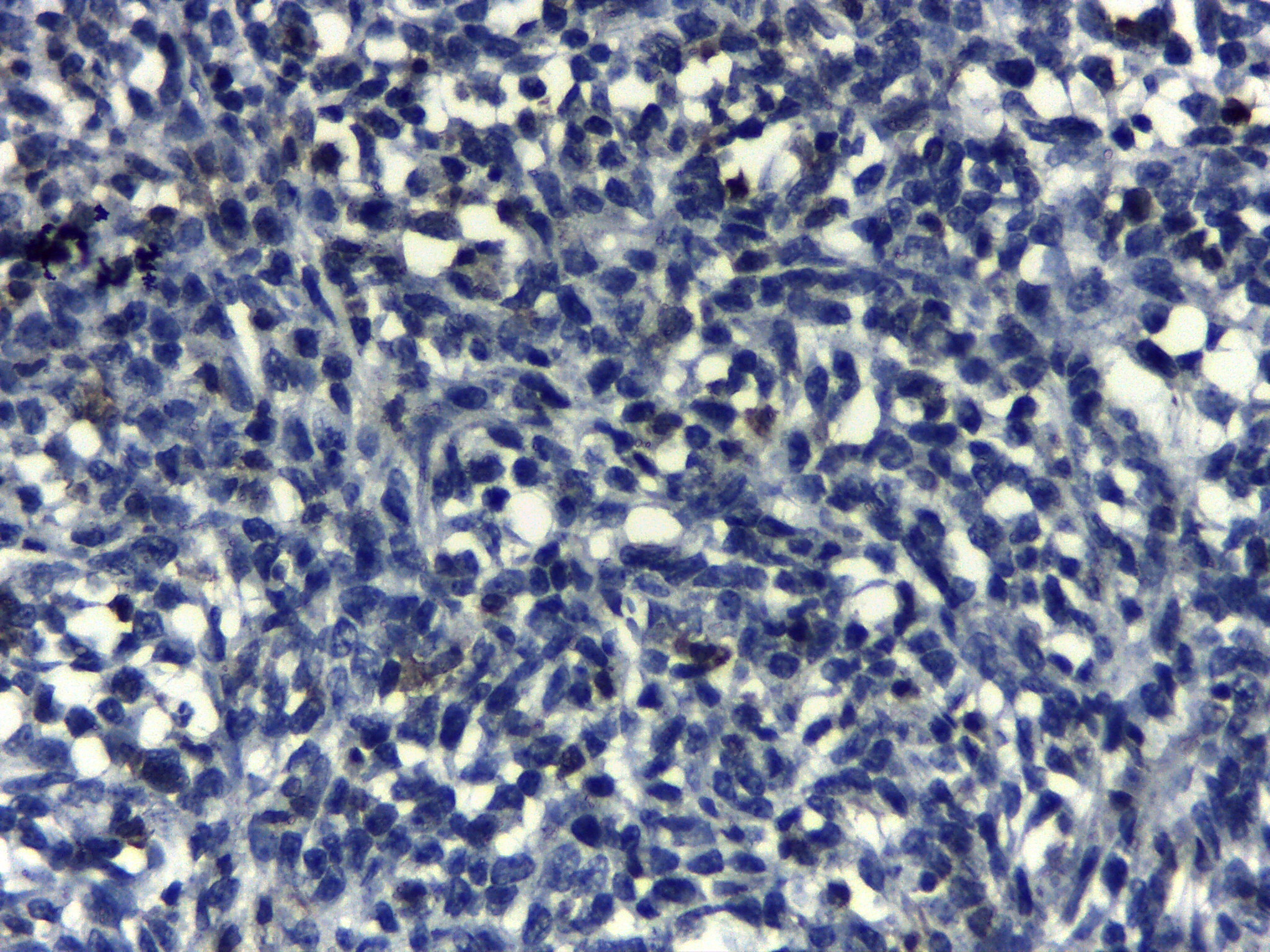

Supplement: S6 Fig — (ZIP) [file pone.0188960.s019.zip › HIF-1a IHC image CON/HIF-1a con1-1.jpg]

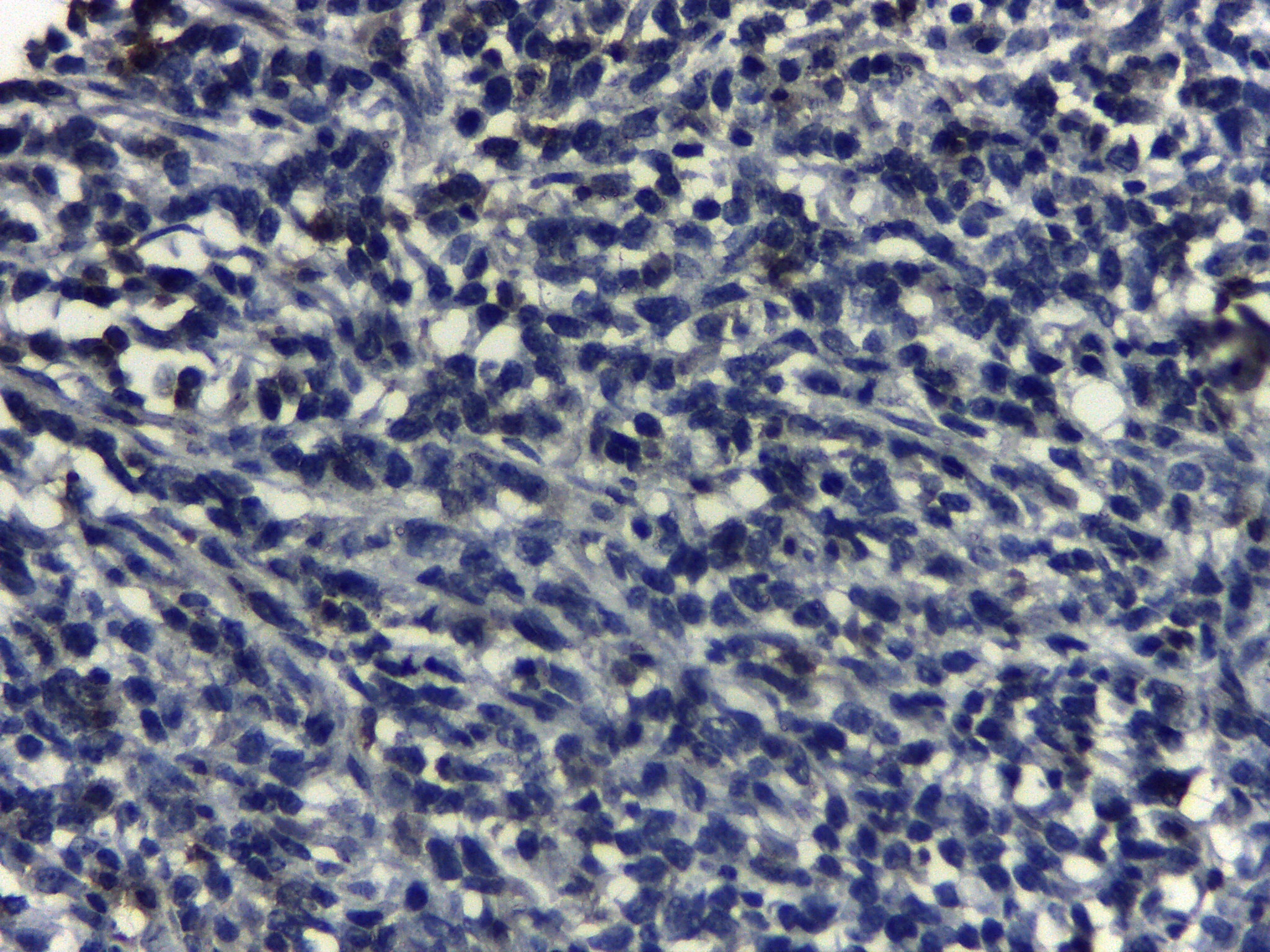

Supplement: S6 Fig — (ZIP) [file pone.0188960.s019.zip › HIF-1a IHC image CON/HIF-1a con1-2.jpg]

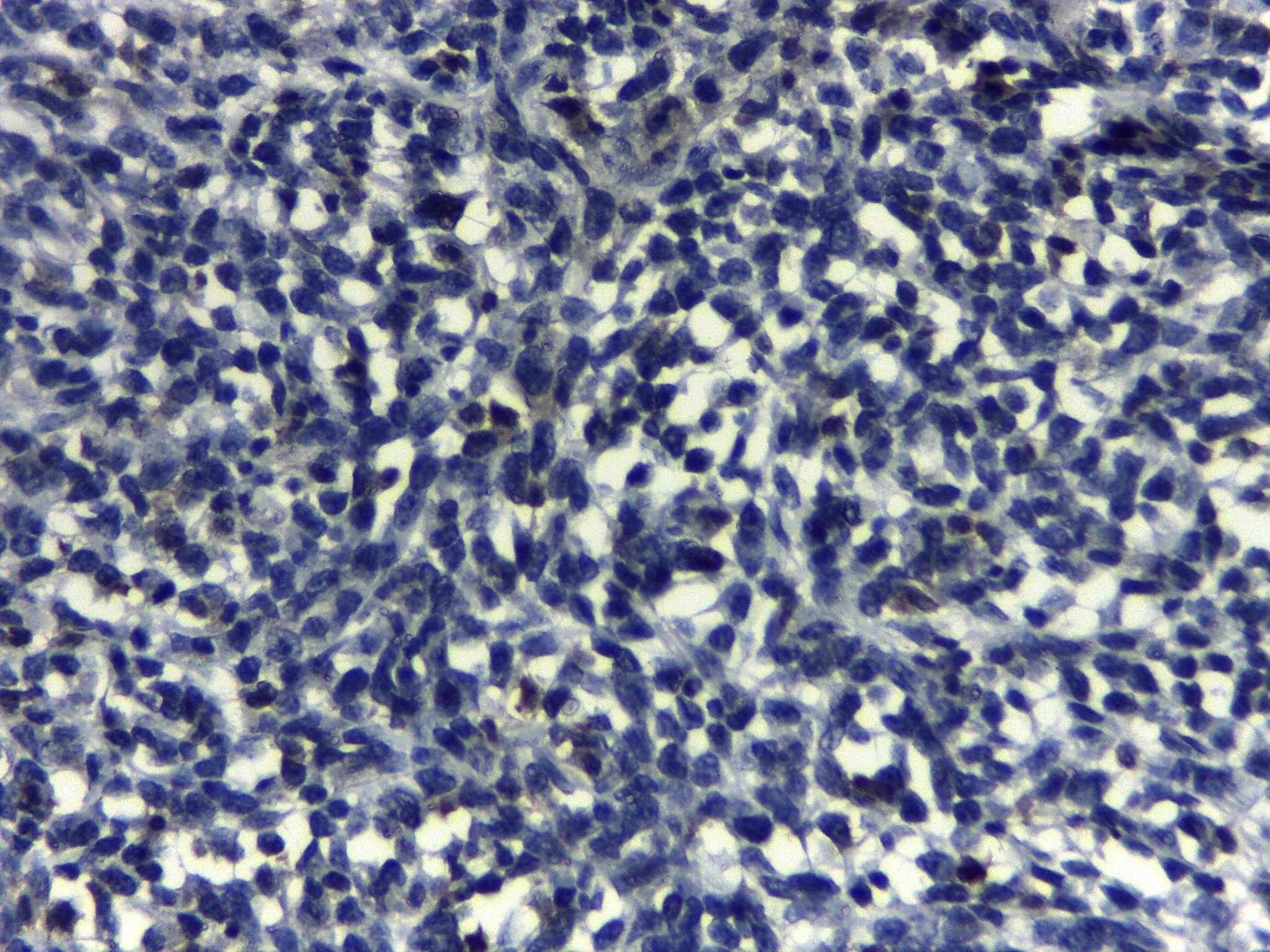

Supplement: S6 Fig — (ZIP) [file pone.0188960.s019.zip › HIF-1a IHC image CON/HIF-1a con1-3.jpg]

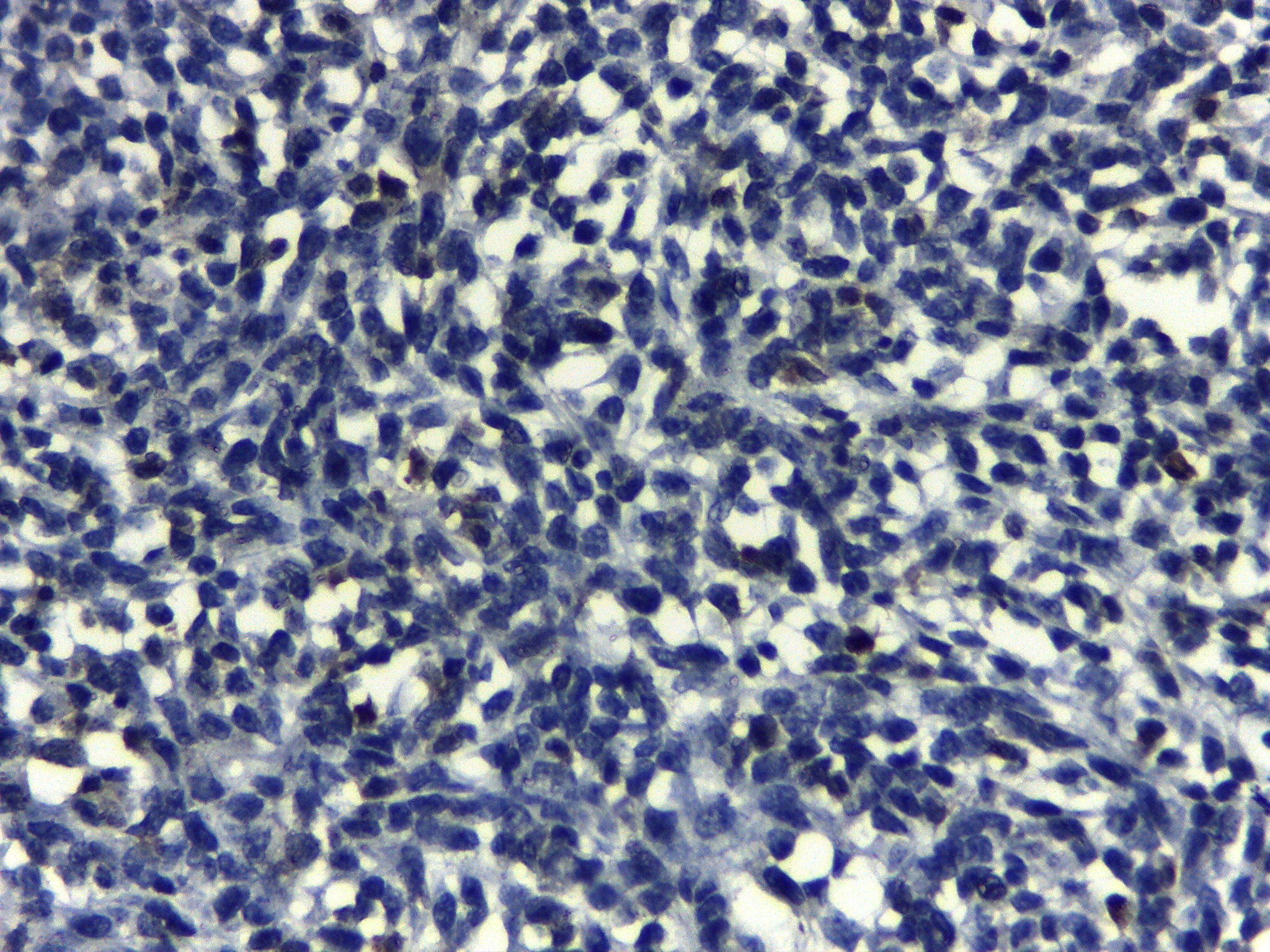

Supplement: S6 Fig — (ZIP) [file pone.0188960.s019.zip › HIF-1a IHC image CON/HIF-1a con1-4.jpg]

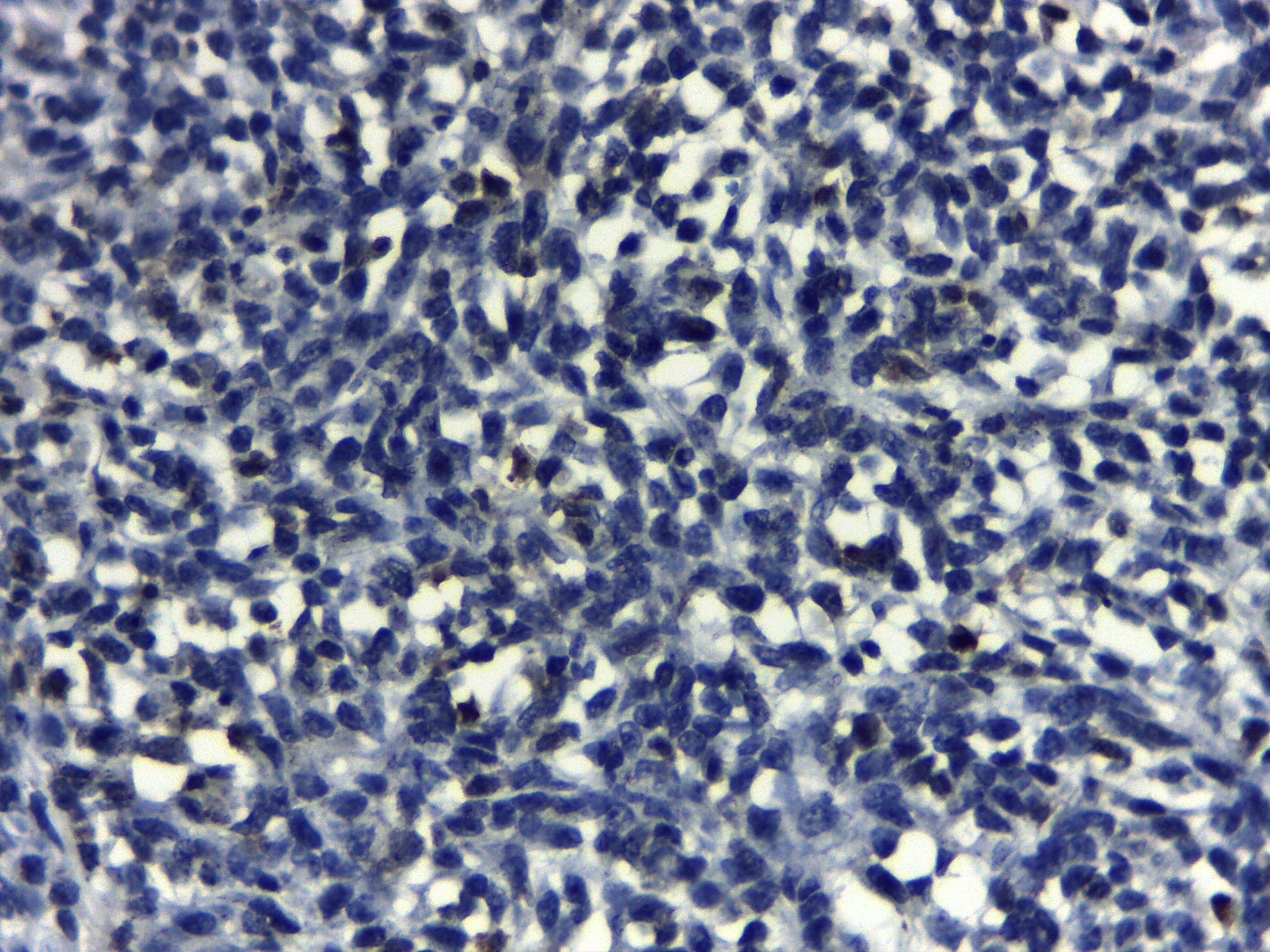

Supplement: S6 Fig — (ZIP) [file pone.0188960.s019.zip › HIF-1a IHC image CON/HIF-1a con1-5.jpg]

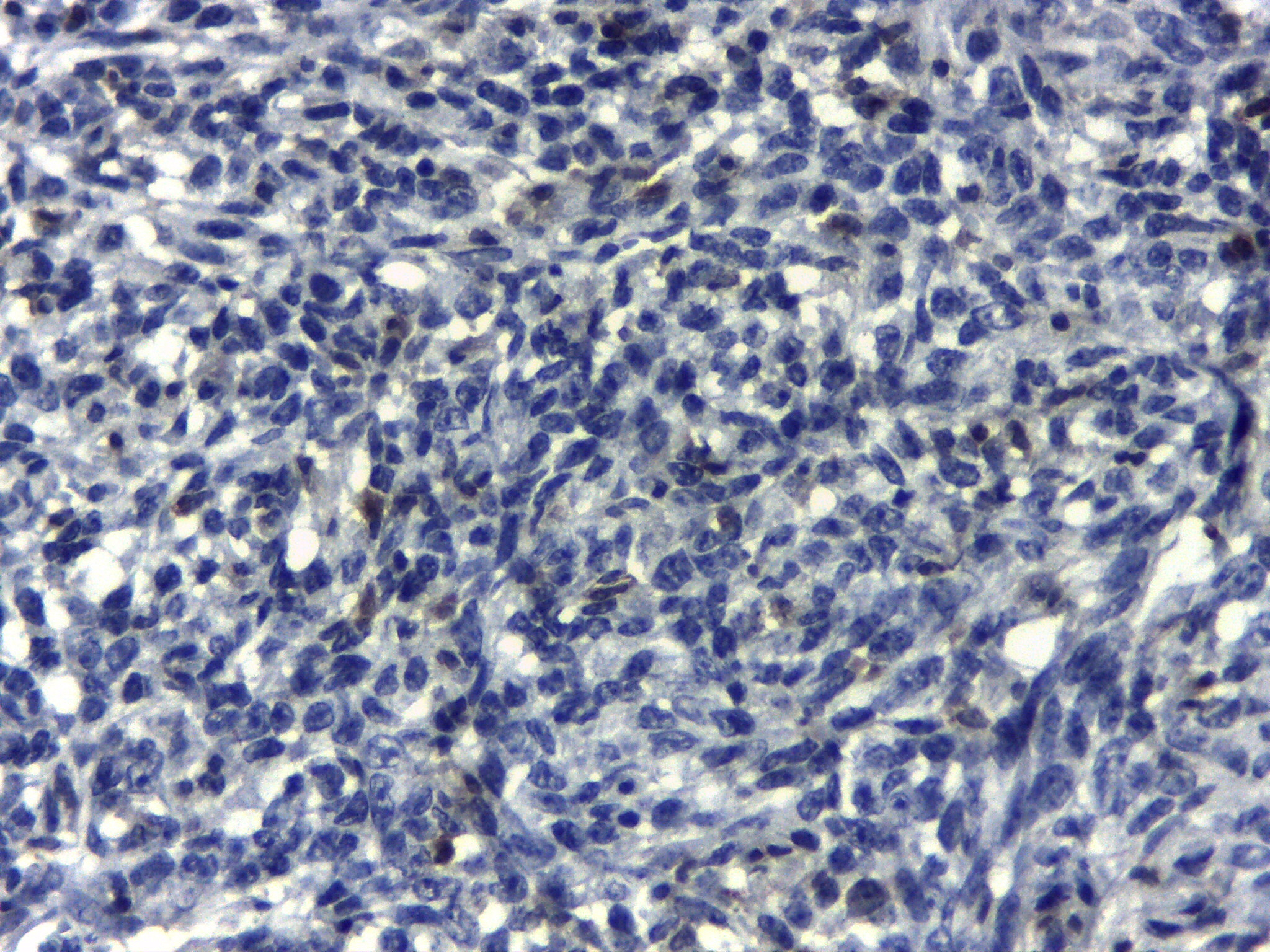

Supplement: S6 Fig — (ZIP) [file pone.0188960.s019.zip › HIF-1a IHC image CON/HIF-1a con2-1.jpg]

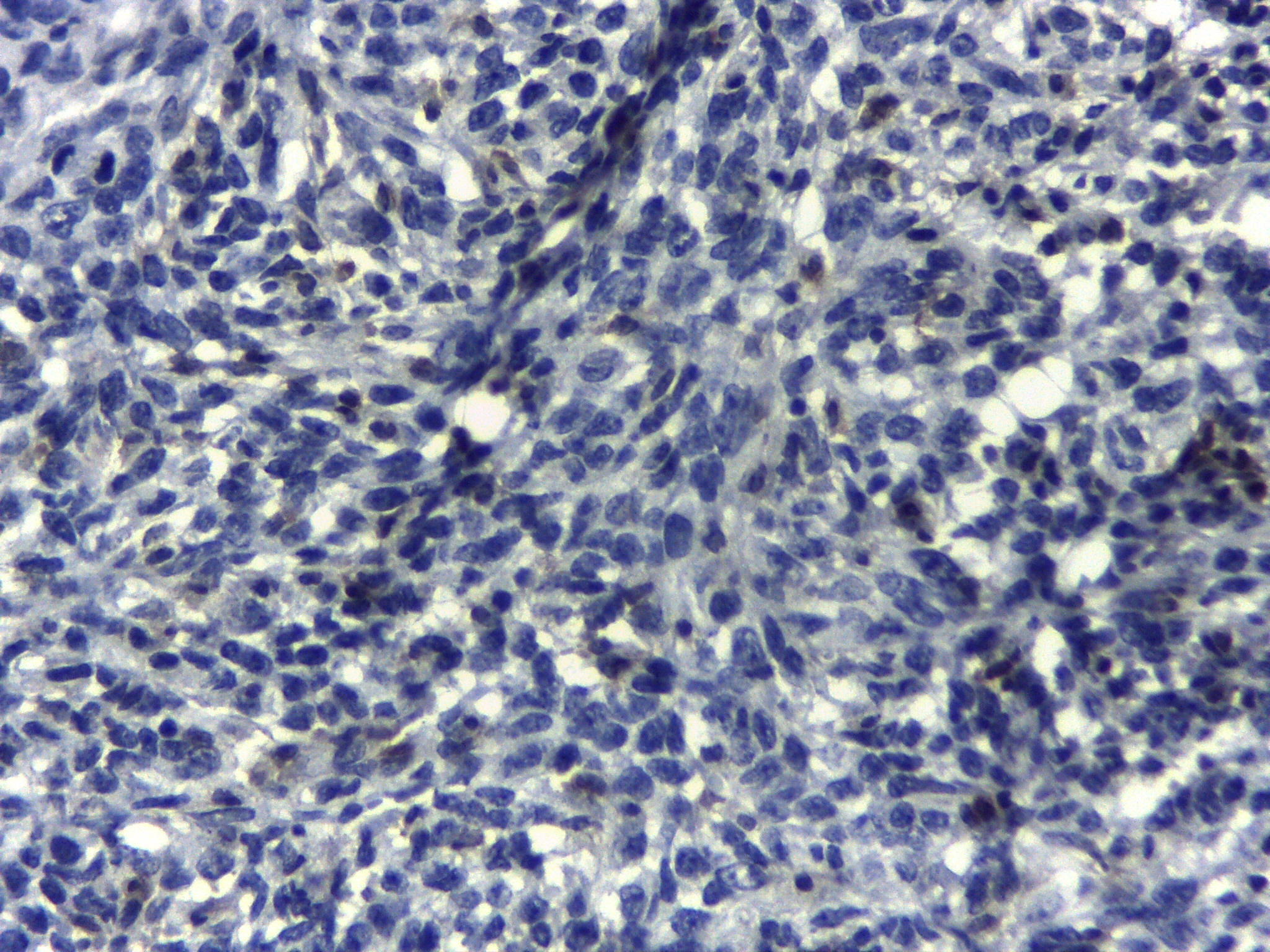

Supplement: S6 Fig — (ZIP) [file pone.0188960.s019.zip › HIF-1a IHC image CON/HIF-1a con2-2.jpg]

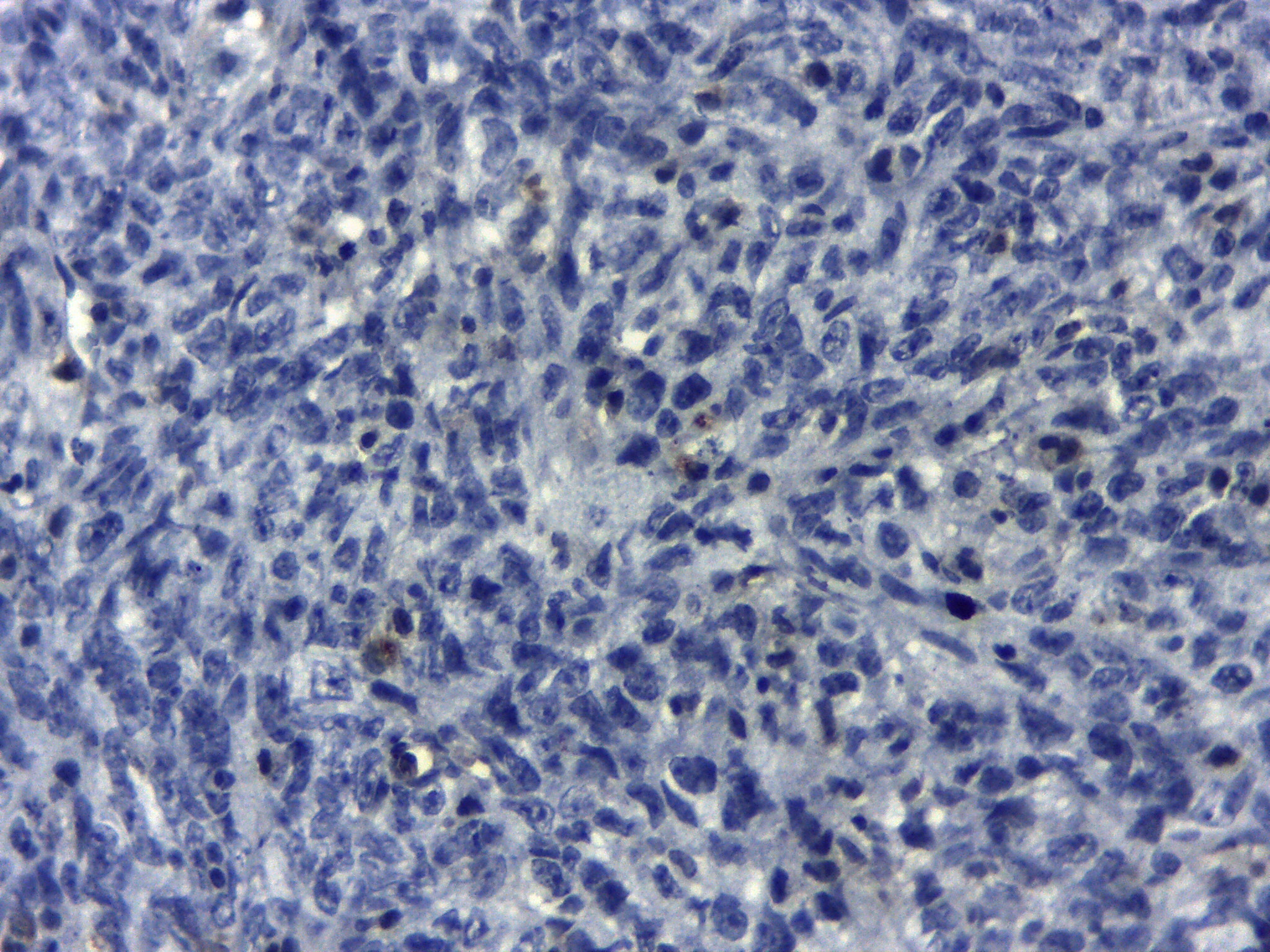

Supplement: S6 Fig — (ZIP) [file pone.0188960.s019.zip › HIF-1a IHC image CON/HIF-1a con2-3.jpg]

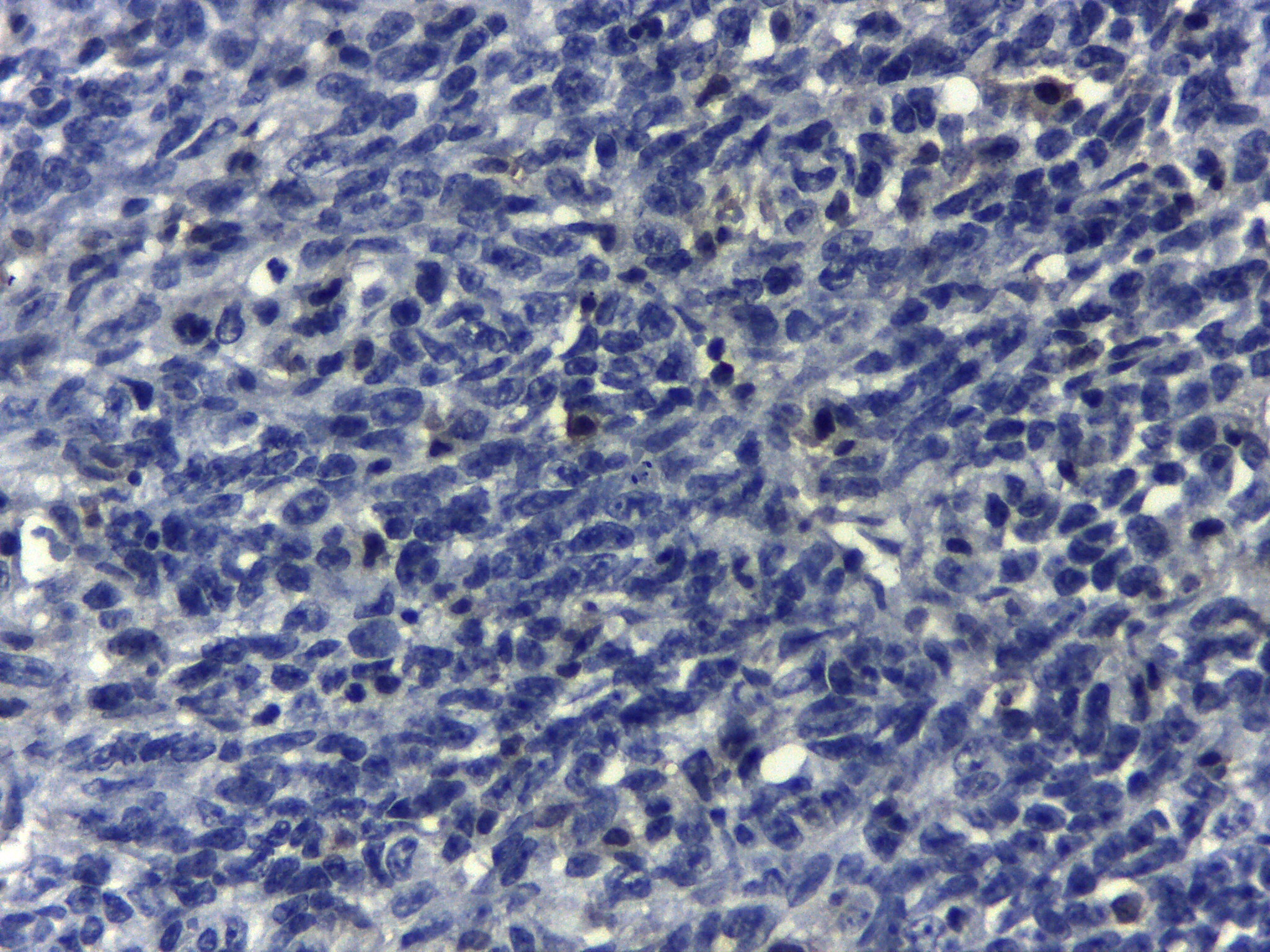

Supplement: S6 Fig — (ZIP) [file pone.0188960.s019.zip › HIF-1a IHC image CON/HIF-1a con2-4.jpg]

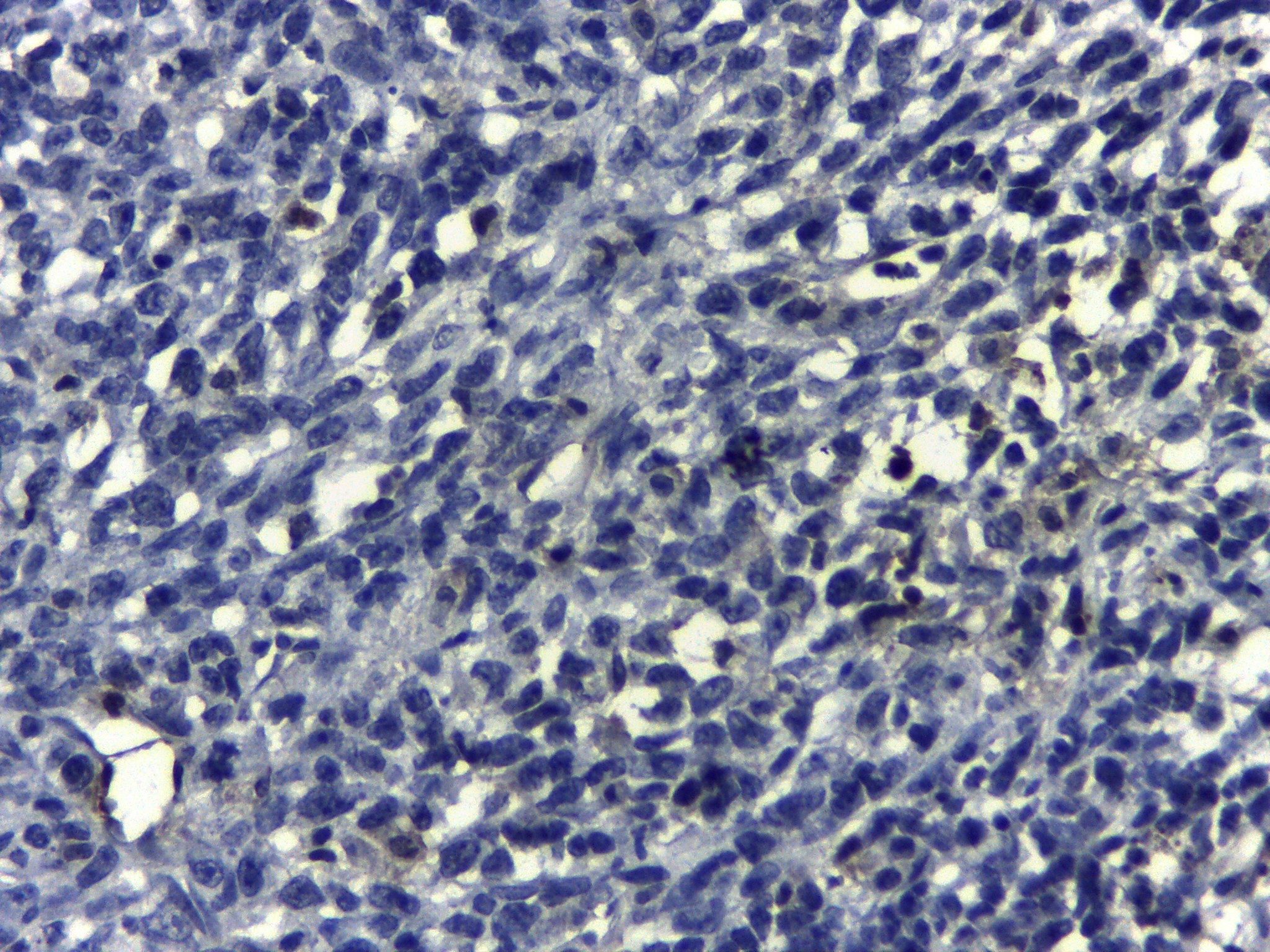

Supplement: S6 Fig — (ZIP) [file pone.0188960.s019.zip › HIF-1a IHC image CON/HIF-1a con2-5.jpg]

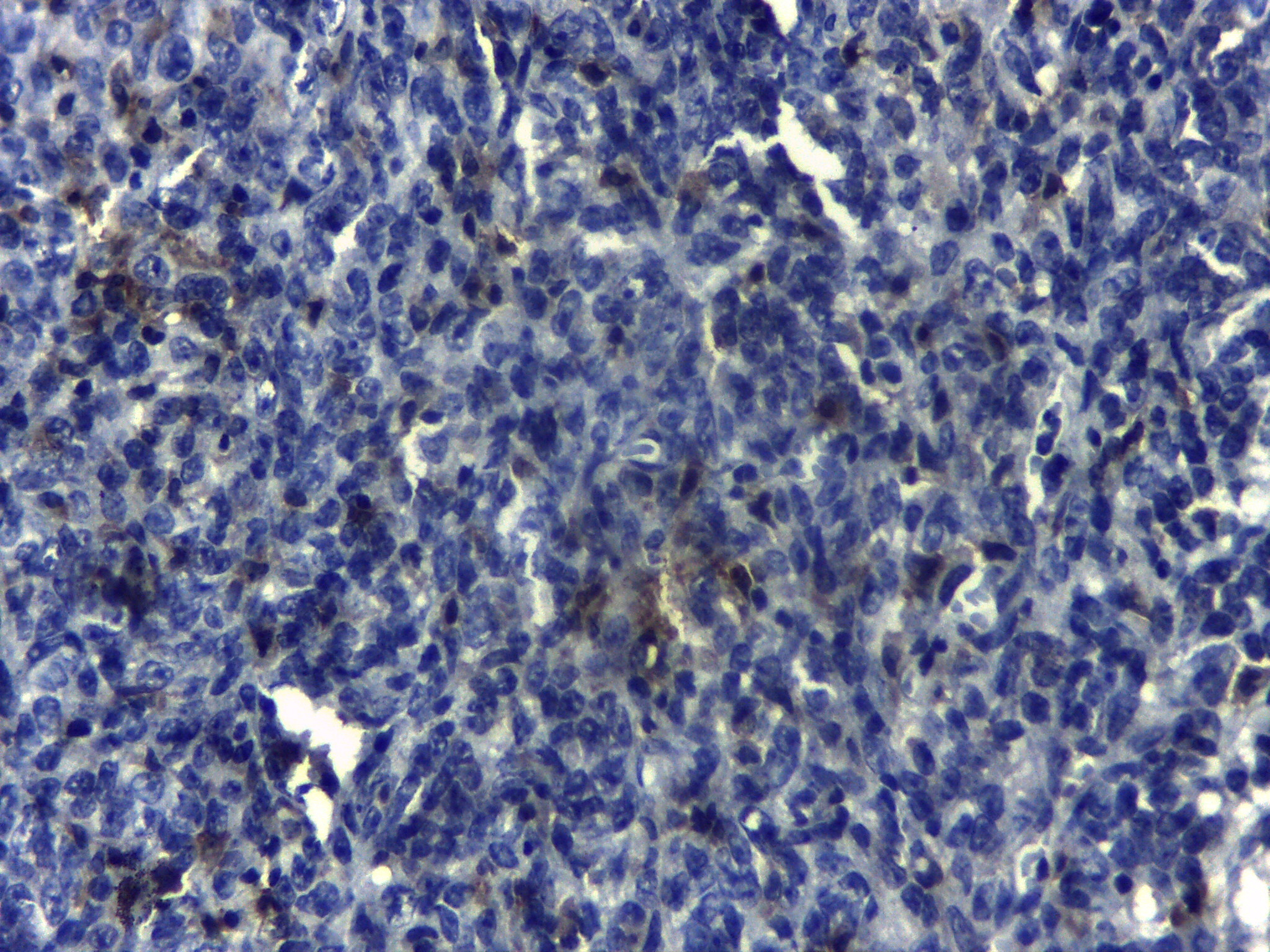

Supplement: S6 Fig — (ZIP) [file pone.0188960.s019.zip › HIF-1a IHC image CON/HIF-1a con3-1.jpg]

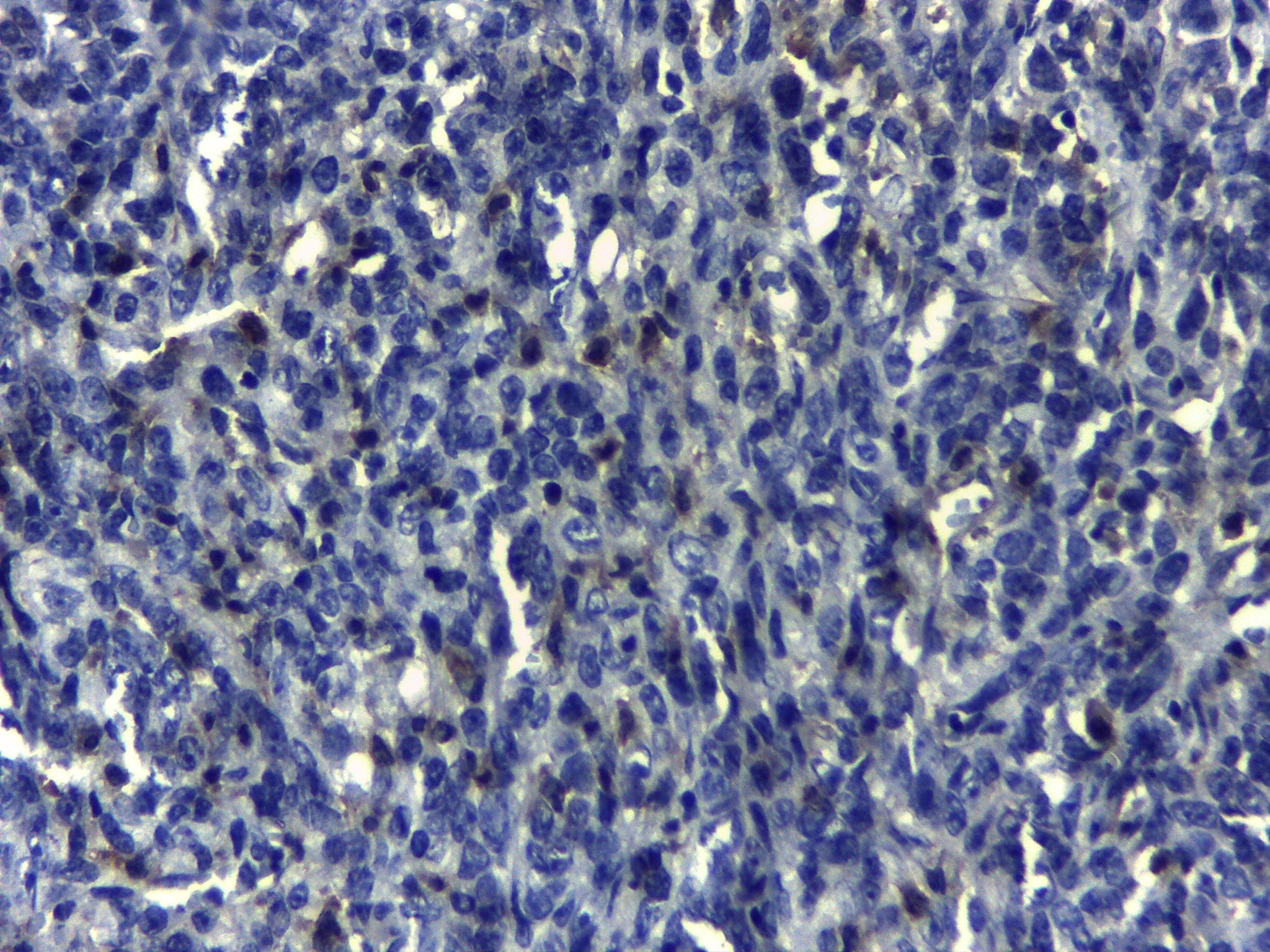

Supplement: S6 Fig — (ZIP) [file pone.0188960.s019.zip › HIF-1a IHC image CON/HIF-1a con3-2.jpg]

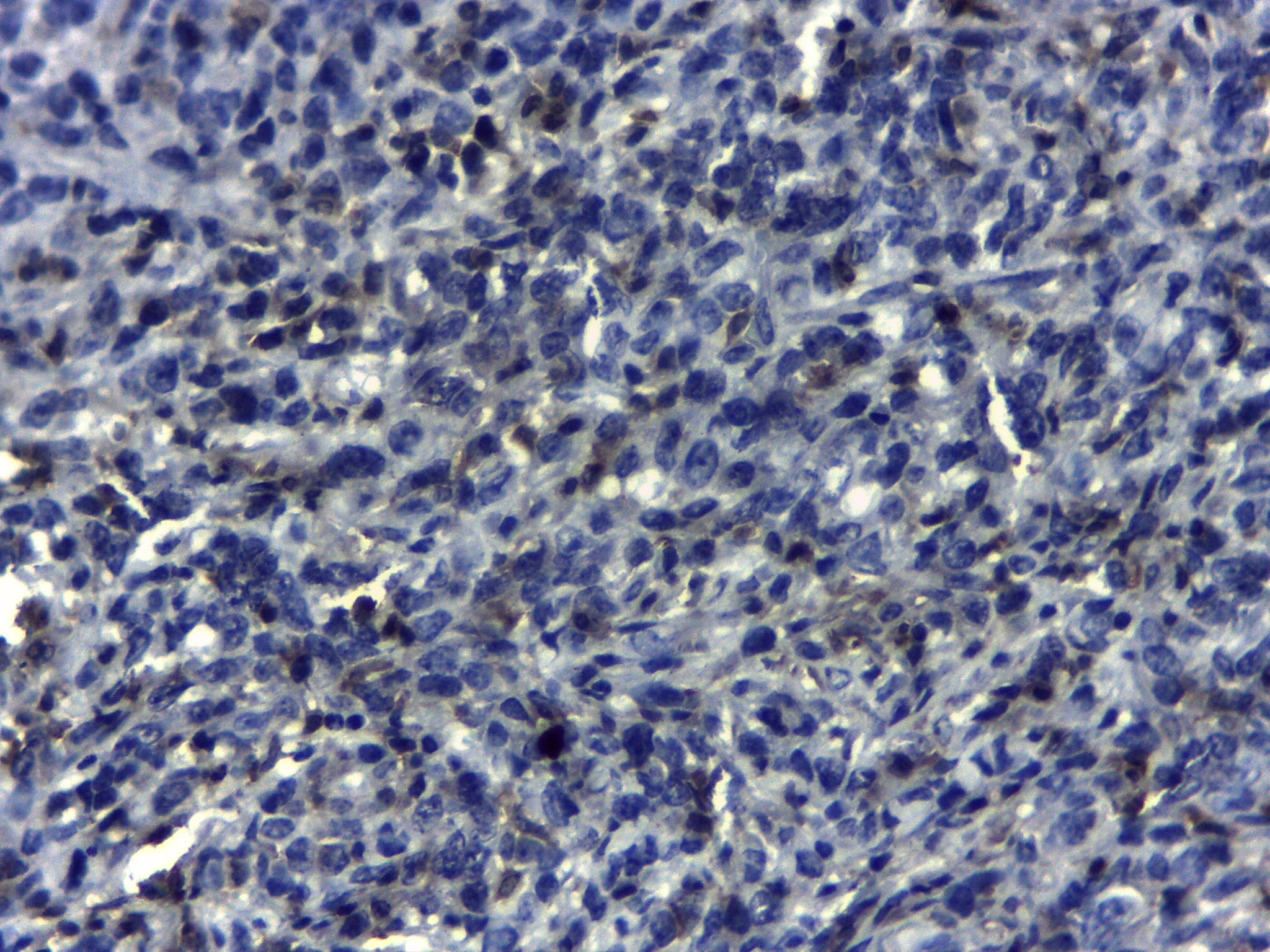

Supplement: S6 Fig — (ZIP) [file pone.0188960.s019.zip › HIF-1a IHC image CON/HIF-1a con3-3.jpg]

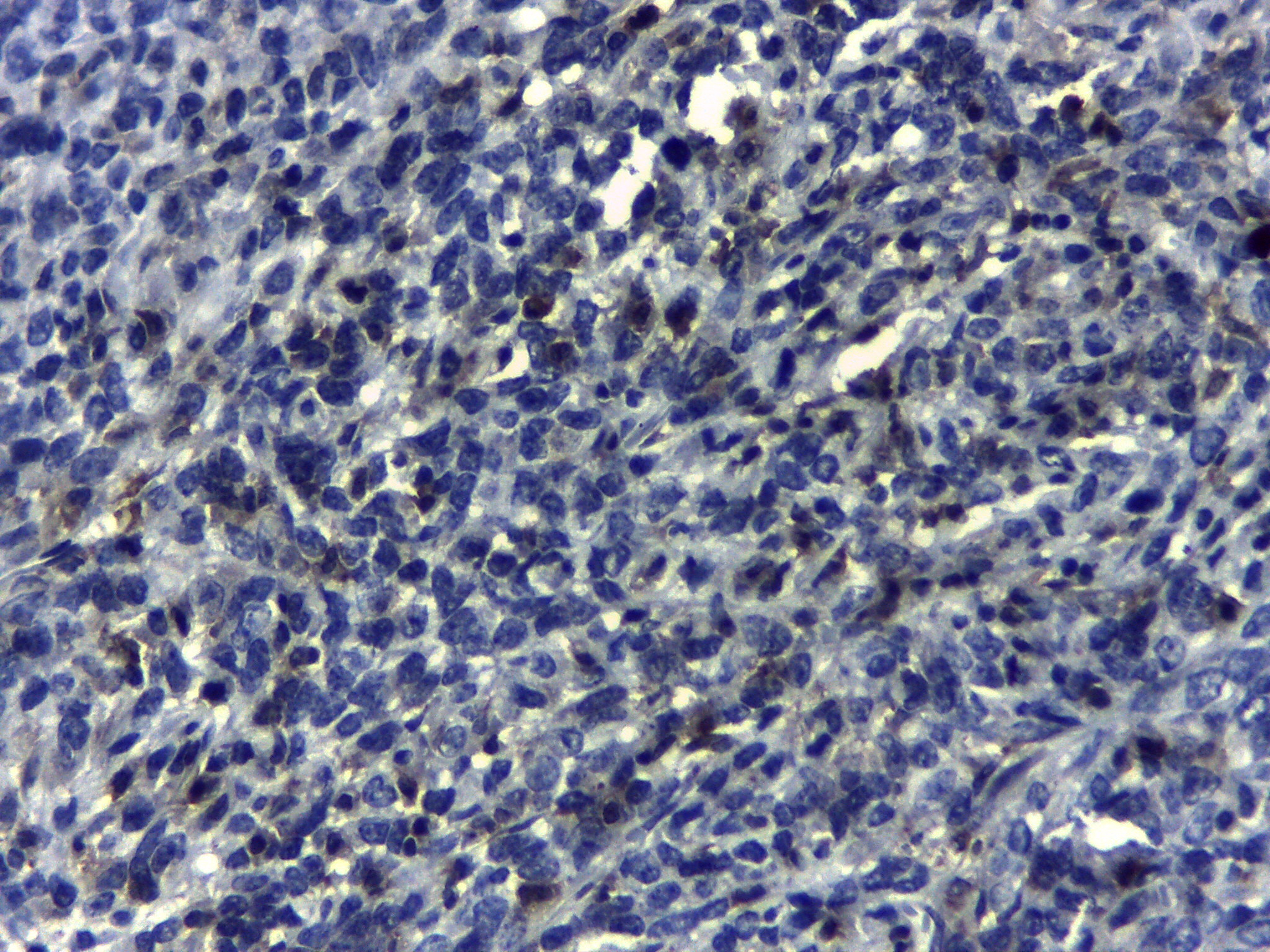

Supplement: S6 Fig — (ZIP) [file pone.0188960.s019.zip › HIF-1a IHC image CON/HIF-1a con3-4.jpg]

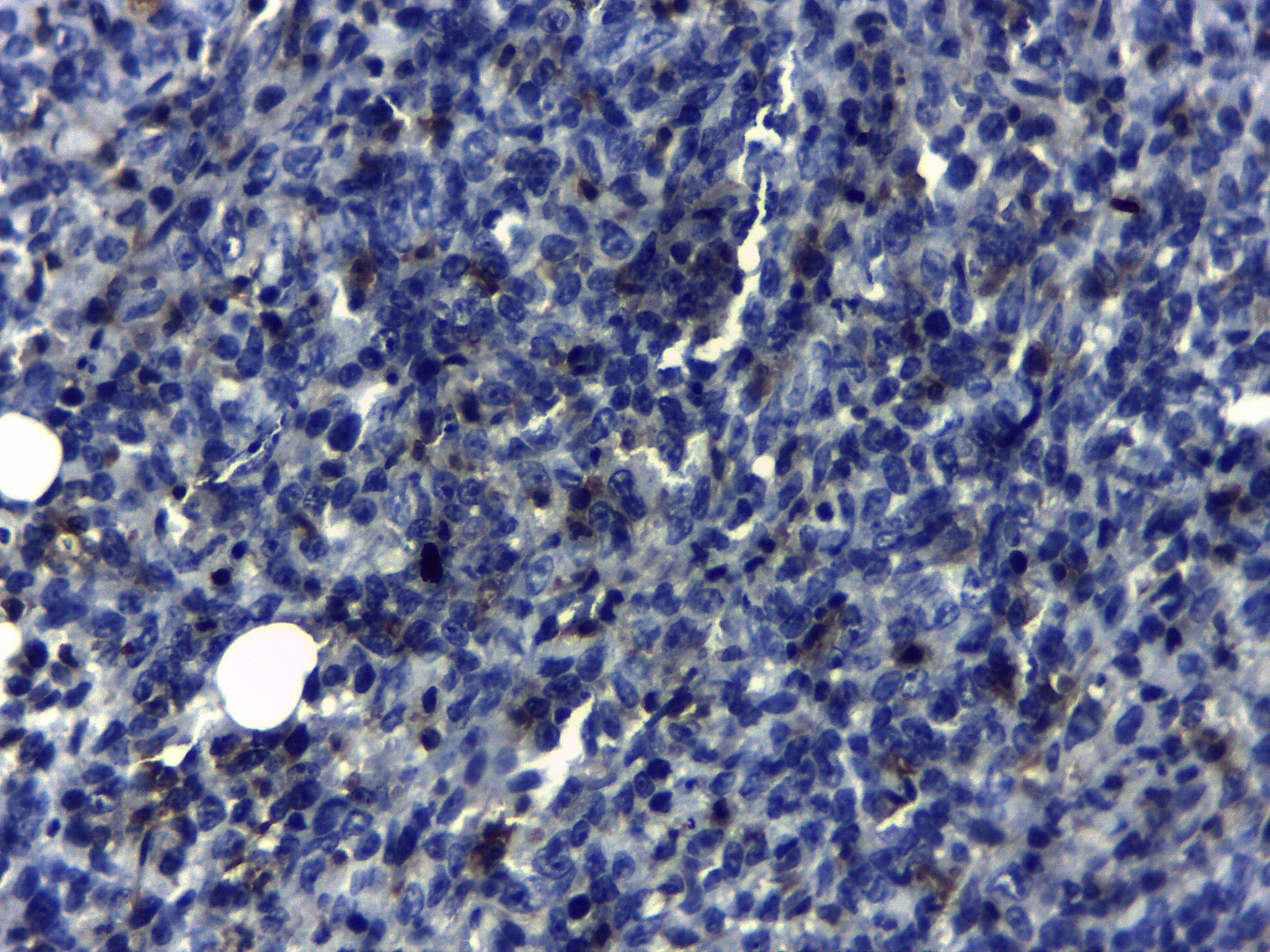

Supplement: S6 Fig — (ZIP) [file pone.0188960.s019.zip › HIF-1a IHC image CON/HIF-1a con3-5.jpg]

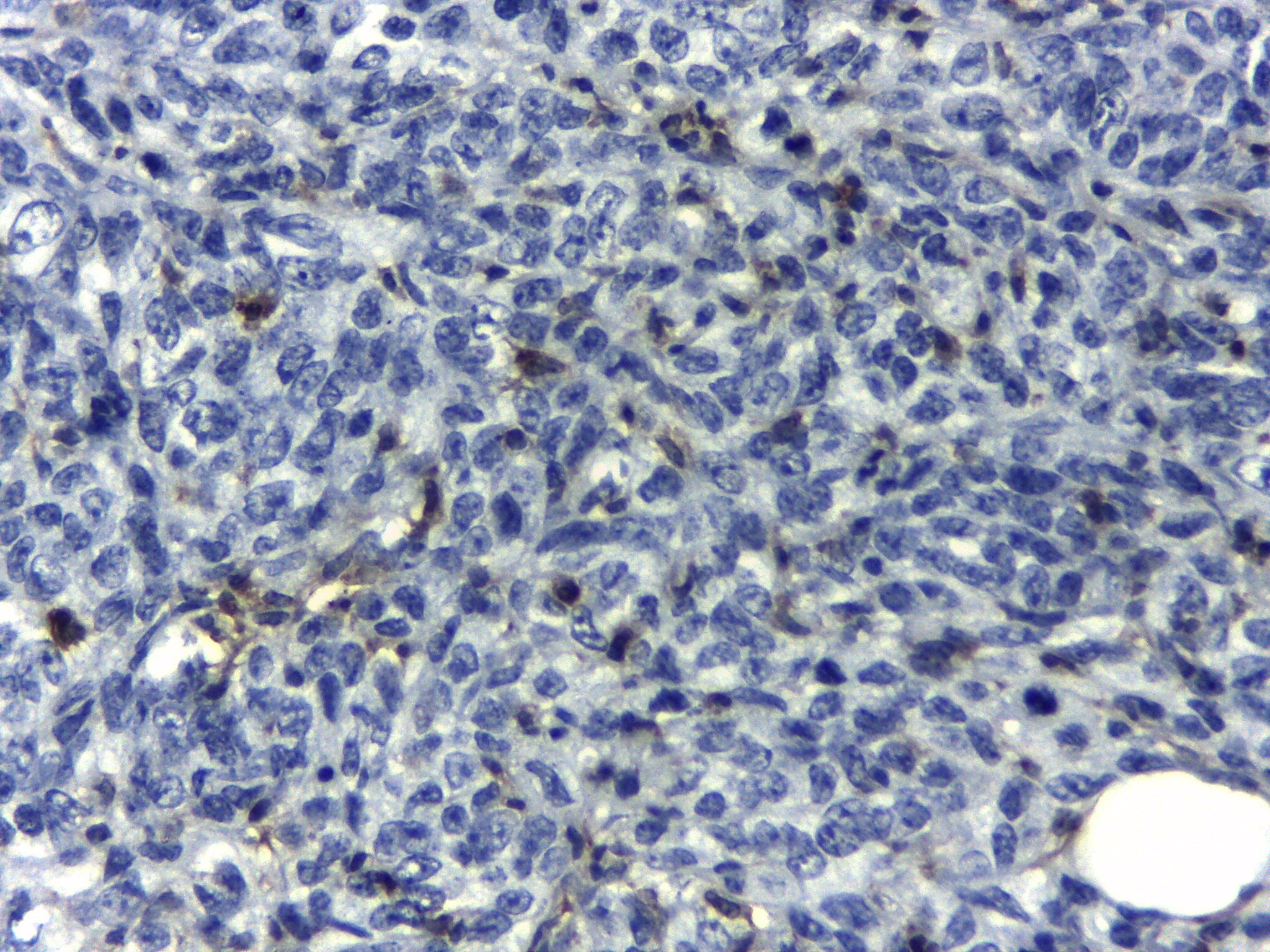

Supplement: S6 Fig — (ZIP) [file pone.0188960.s019.zip › HIF-1a IHC image CON/HIF-1a con4-1.jpg]

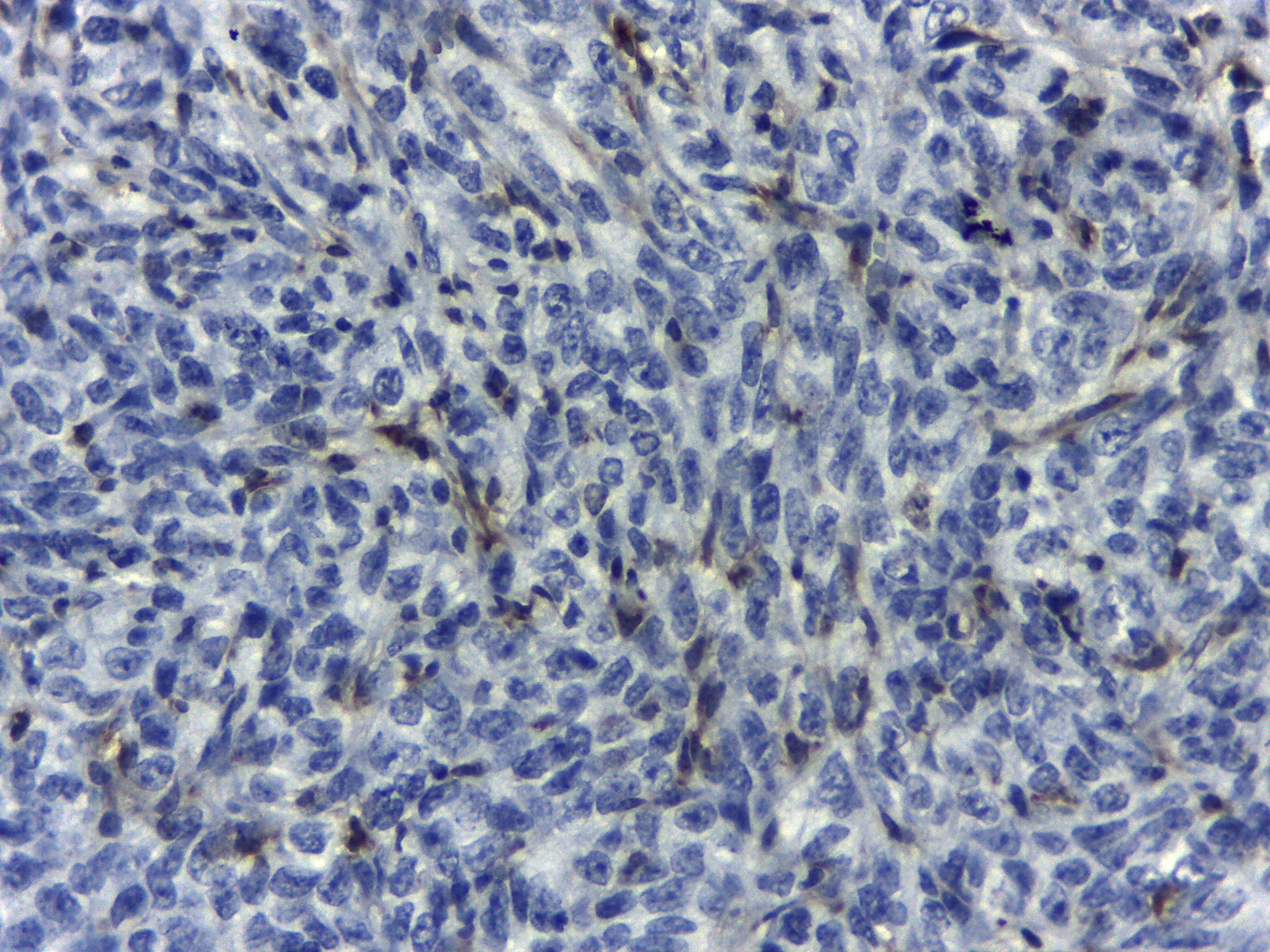

Supplement: S6 Fig — (ZIP) [file pone.0188960.s019.zip › HIF-1a IHC image CON/HIF-1a con4-2.jpg]

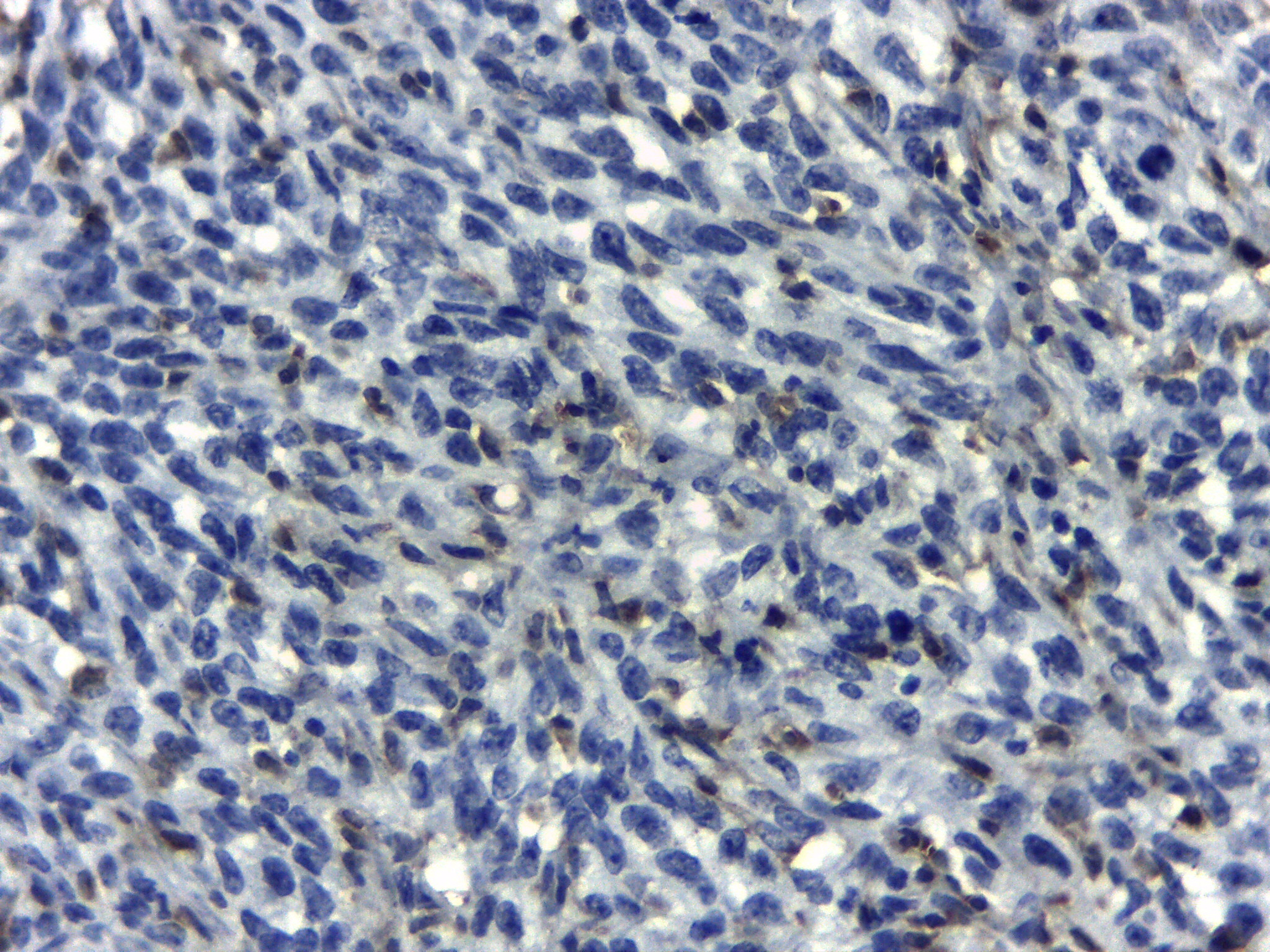

Supplement: S6 Fig — (ZIP) [file pone.0188960.s019.zip › HIF-1a IHC image CON/HIF-1a con4-3.jpg]

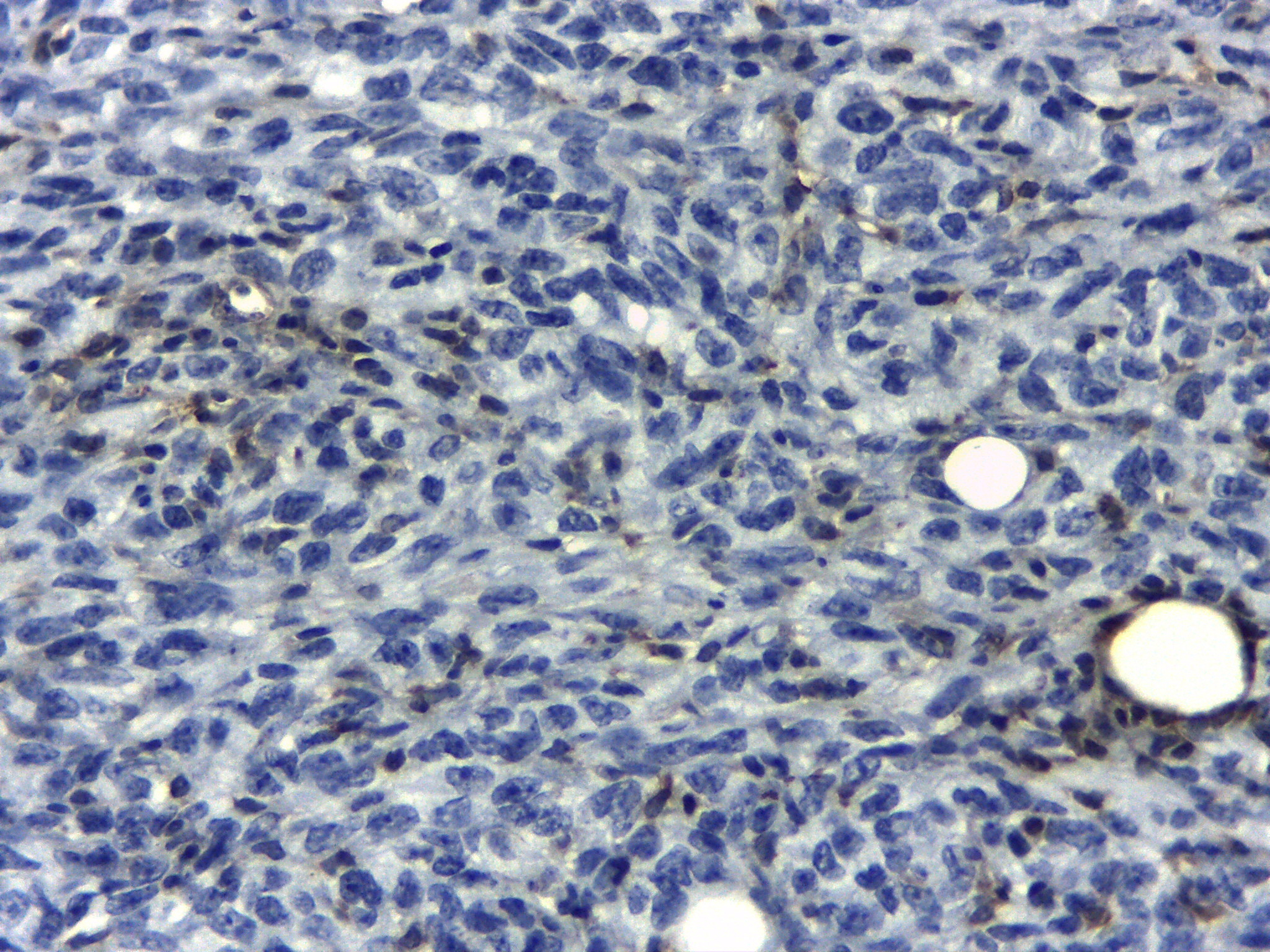

Supplement: S6 Fig — (ZIP) [file pone.0188960.s019.zip › HIF-1a IHC image CON/HIF-1a con4-4.jpg]

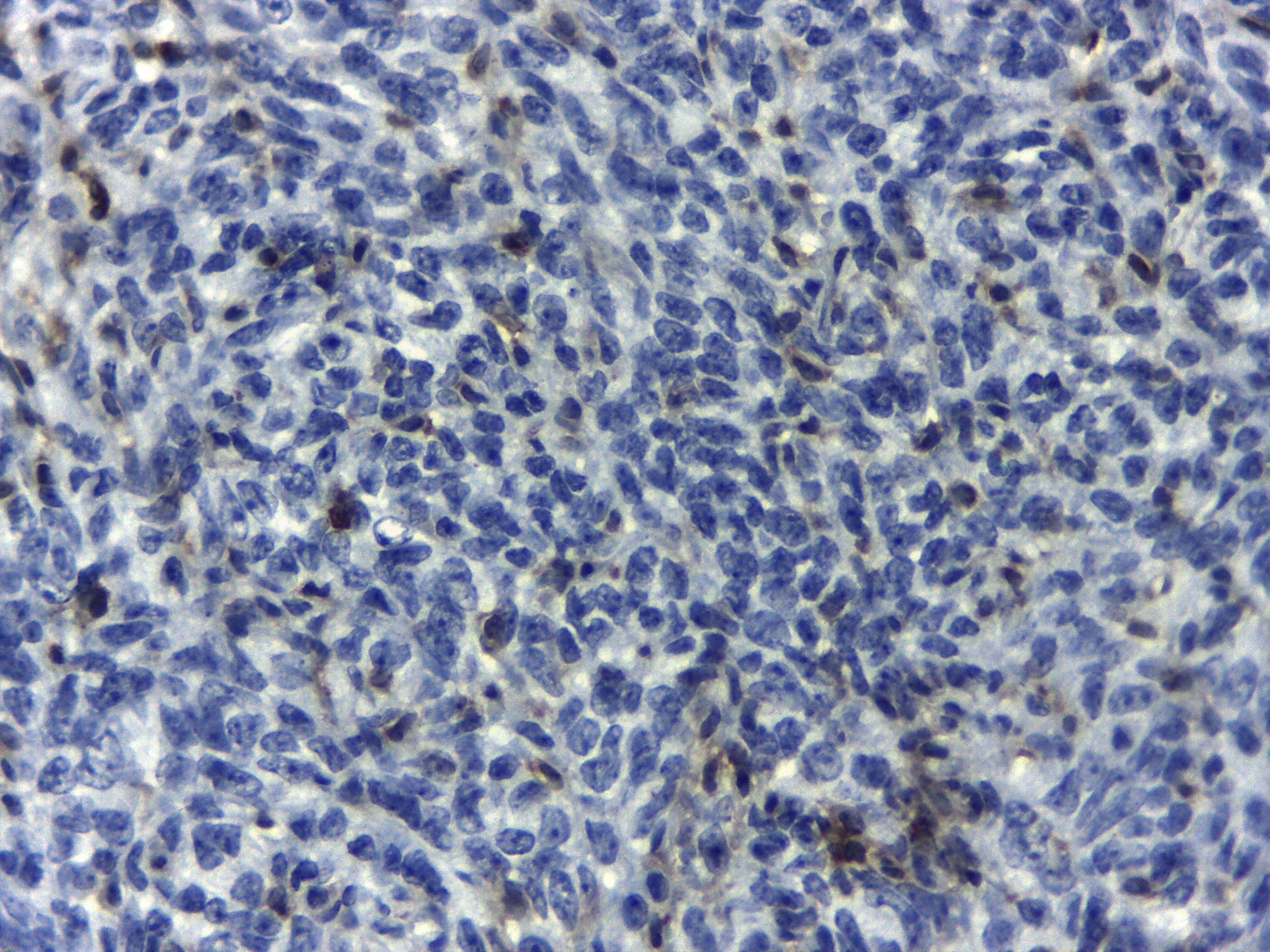

Supplement: S6 Fig — (ZIP) [file pone.0188960.s019.zip › HIF-1a IHC image CON/HIF-1a con4-5.jpg]

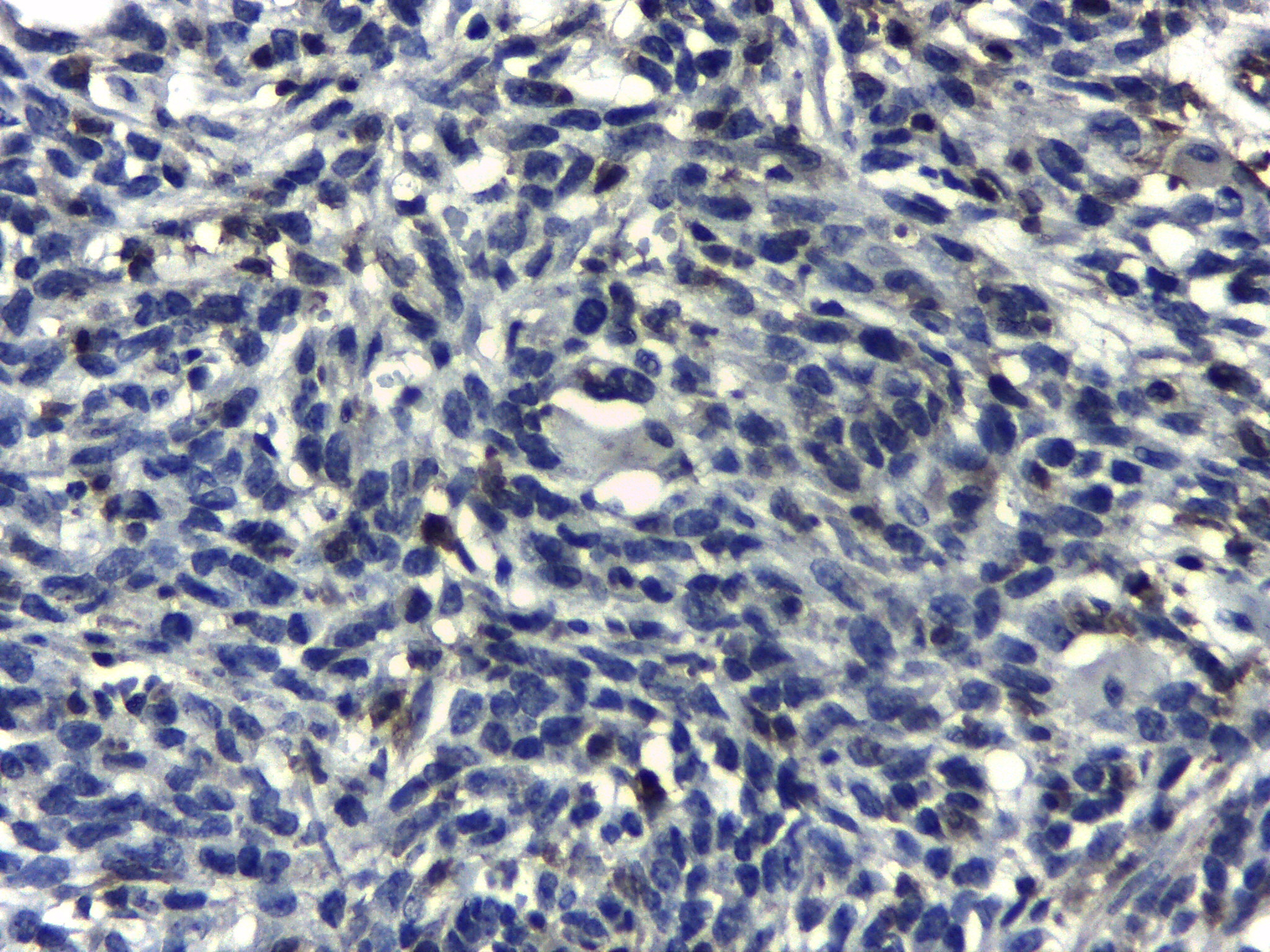

Supplement: S6 Fig — (ZIP) [file pone.0188960.s019.zip › HIF-1a IHC image CON/HIF-1a con5-1.jpg]

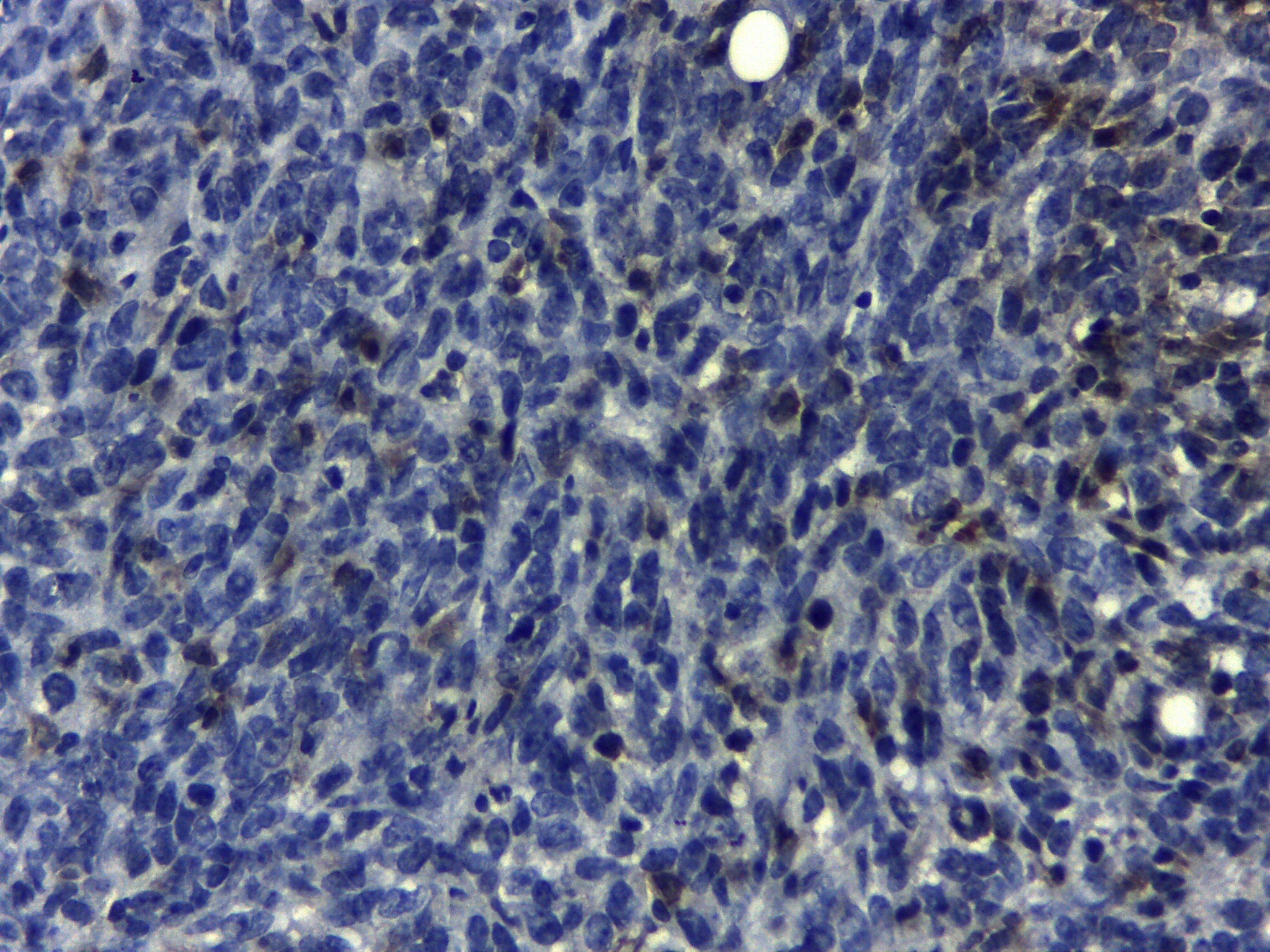

Supplement: S6 Fig — (ZIP) [file pone.0188960.s019.zip › HIF-1a IHC image CON/HIF-1a con5-2.jpg]

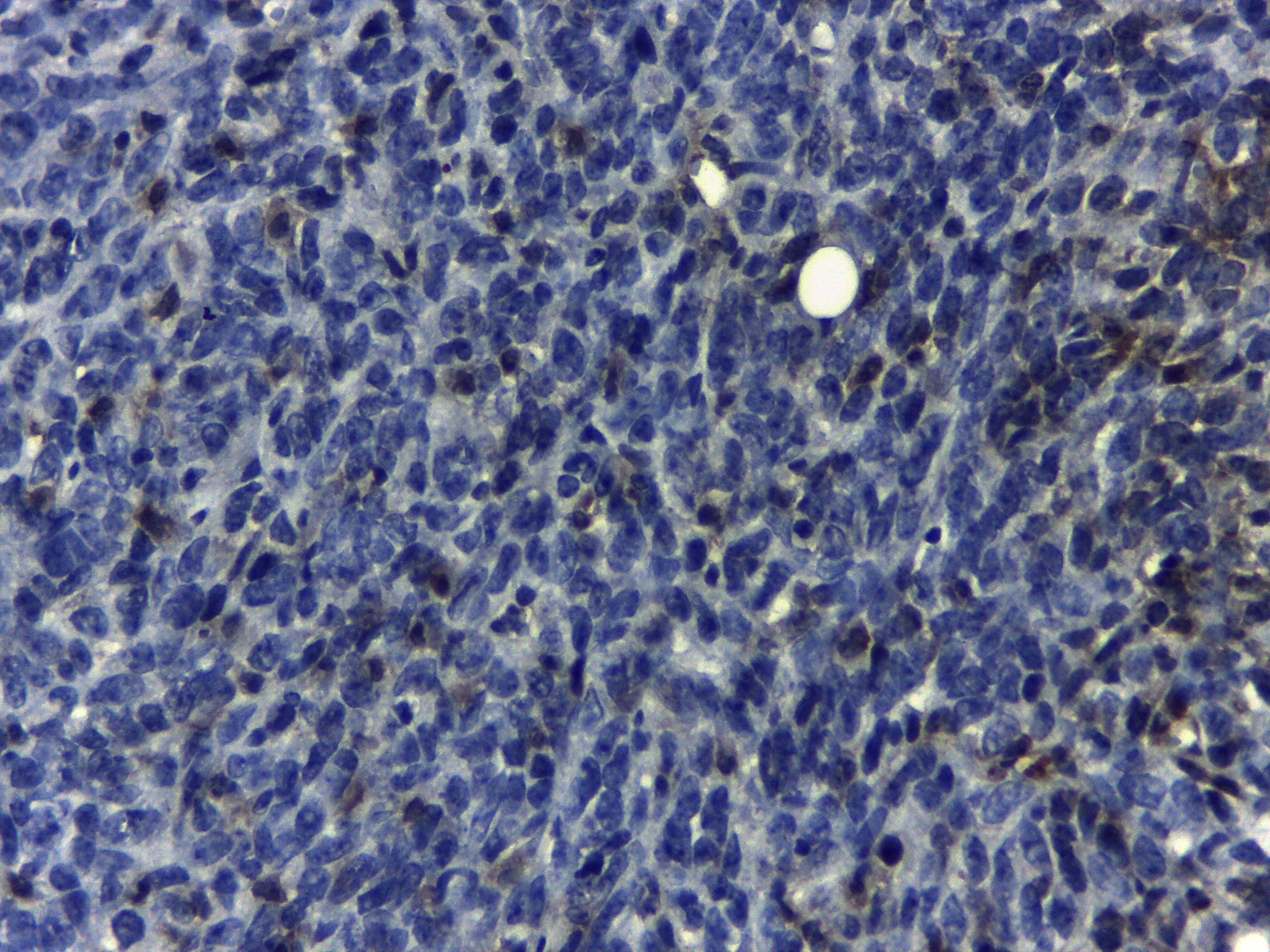

Supplement: S6 Fig — (ZIP) [file pone.0188960.s019.zip › HIF-1a IHC image CON/HIF-1a con5-3.jpg]

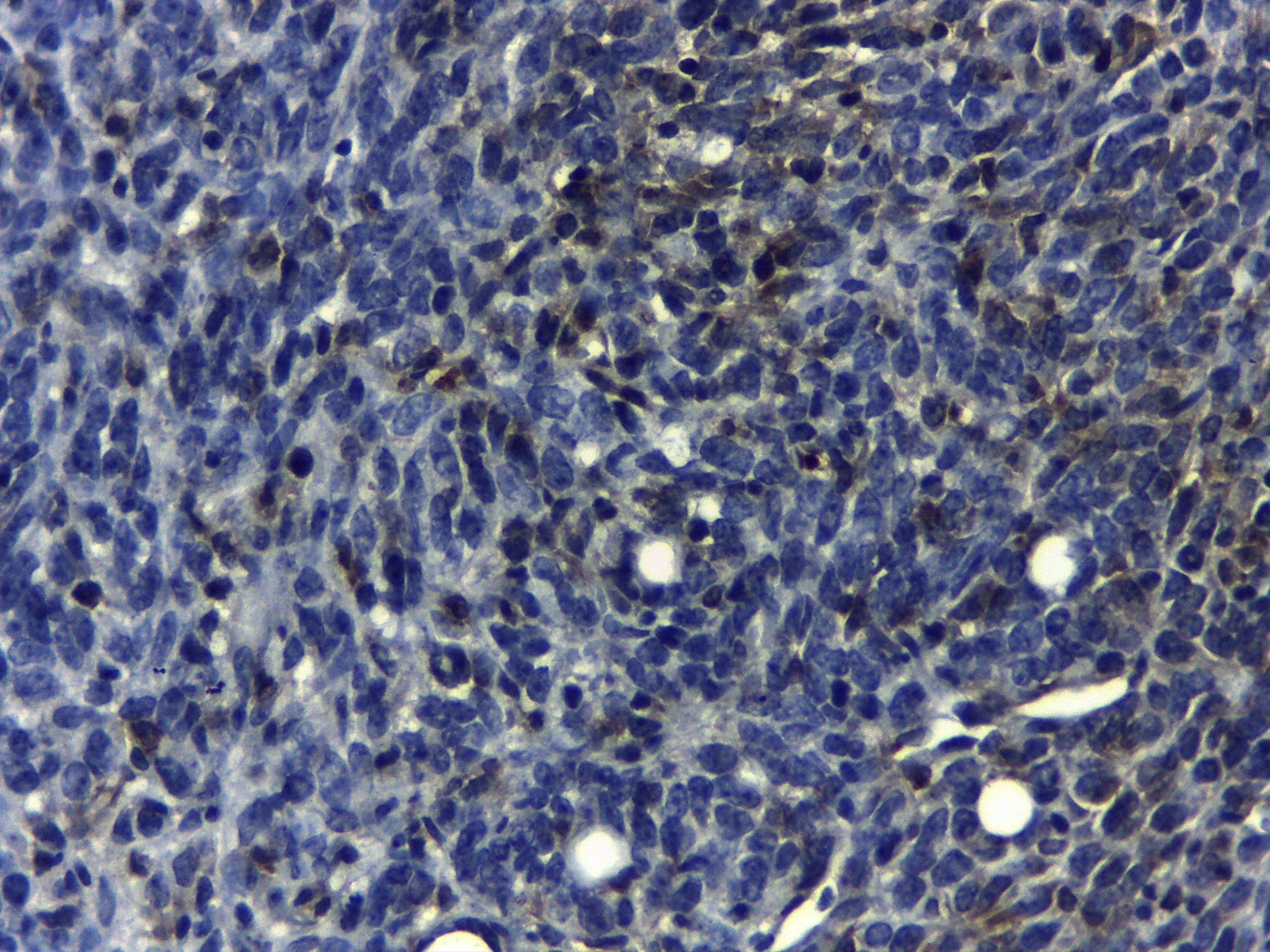

Supplement: S6 Fig — (ZIP) [file pone.0188960.s019.zip › HIF-1a IHC image CON/HIF-1a con5-4.jpg]

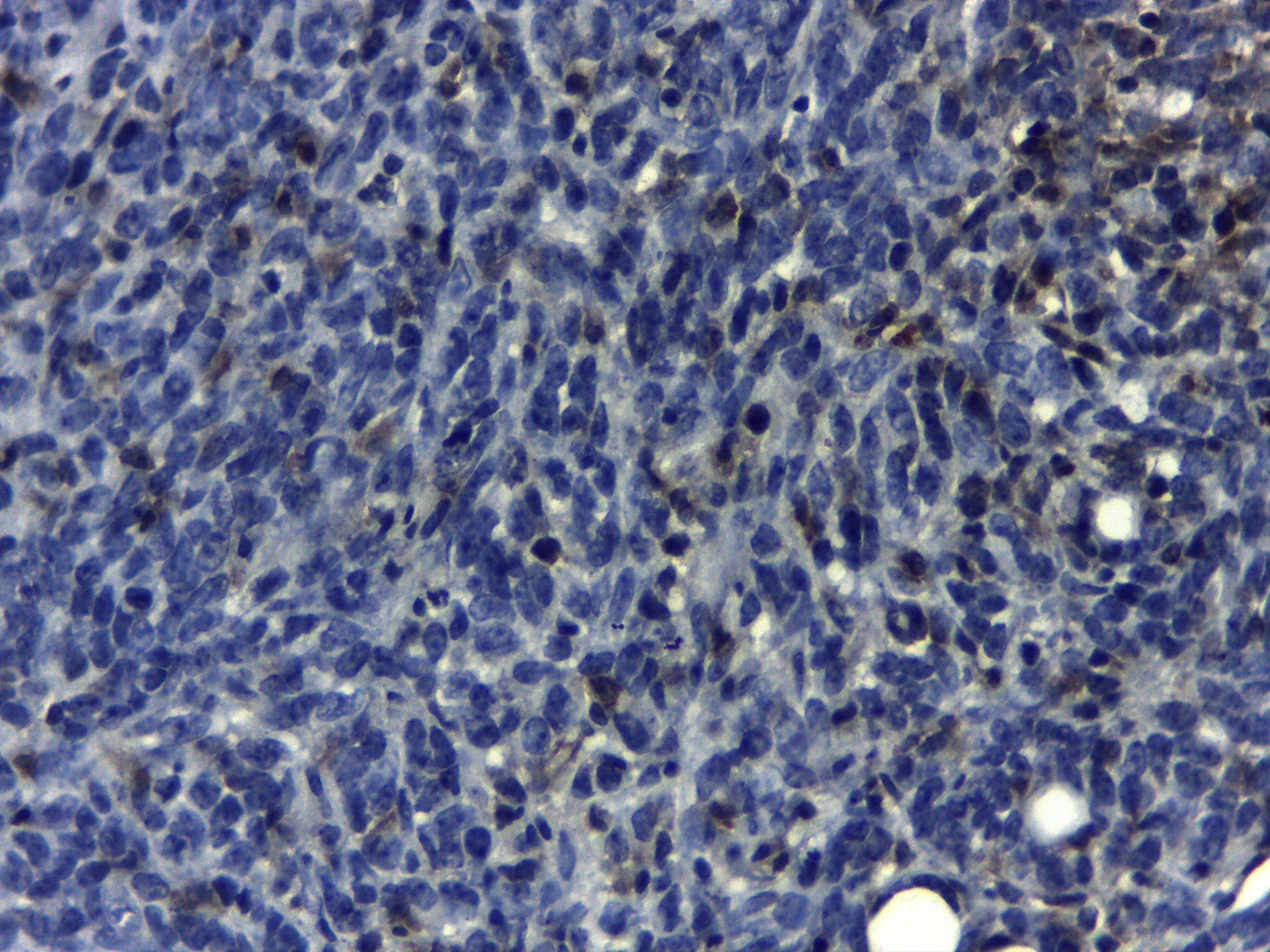

Supplement: S6 Fig — (ZIP) [file pone.0188960.s019.zip › HIF-1a IHC image CON/HIF-1a con5-5.jpg]

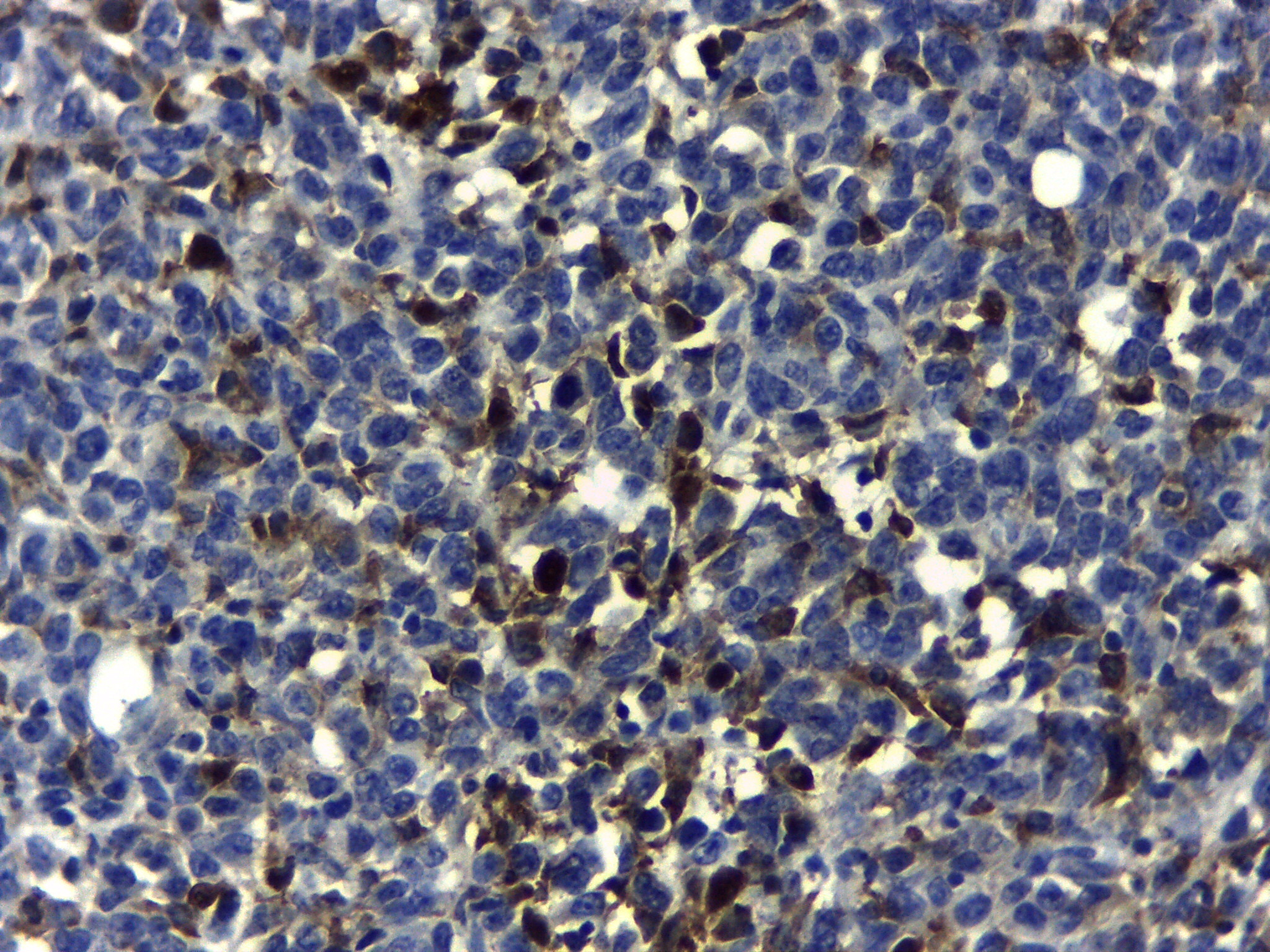

Supplement: S6 Fig — (ZIP) [file pone.0188960.s019.zip › HIF-1a IHC image CON/HIF-1a con6-1.jpg]

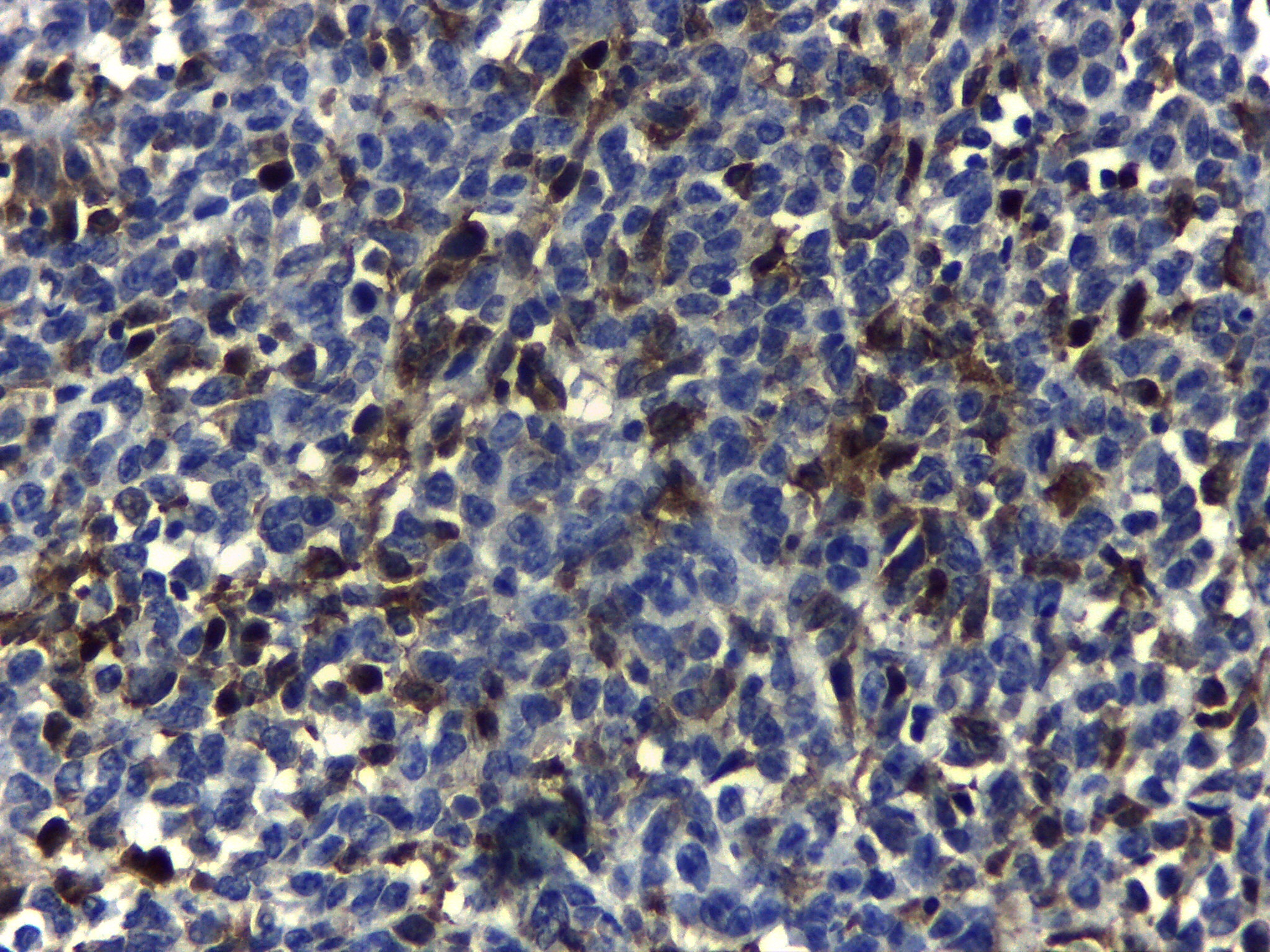

Supplement: S6 Fig — (ZIP) [file pone.0188960.s019.zip › HIF-1a IHC image CON/HIF-1a con6-2.jpg]

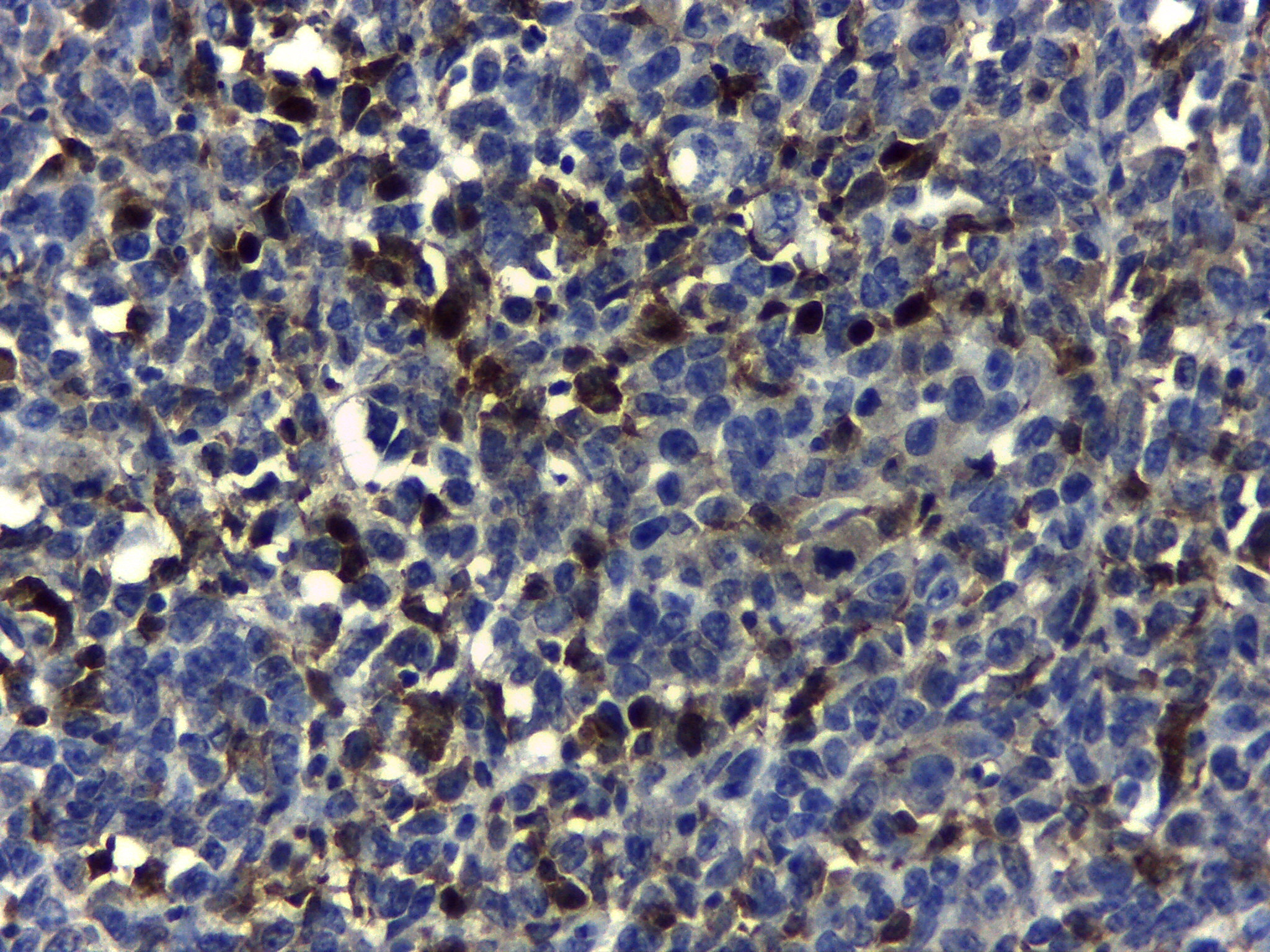

Supplement: S6 Fig — (ZIP) [file pone.0188960.s019.zip › HIF-1a IHC image CON/HIF-1a con6-3.jpg]

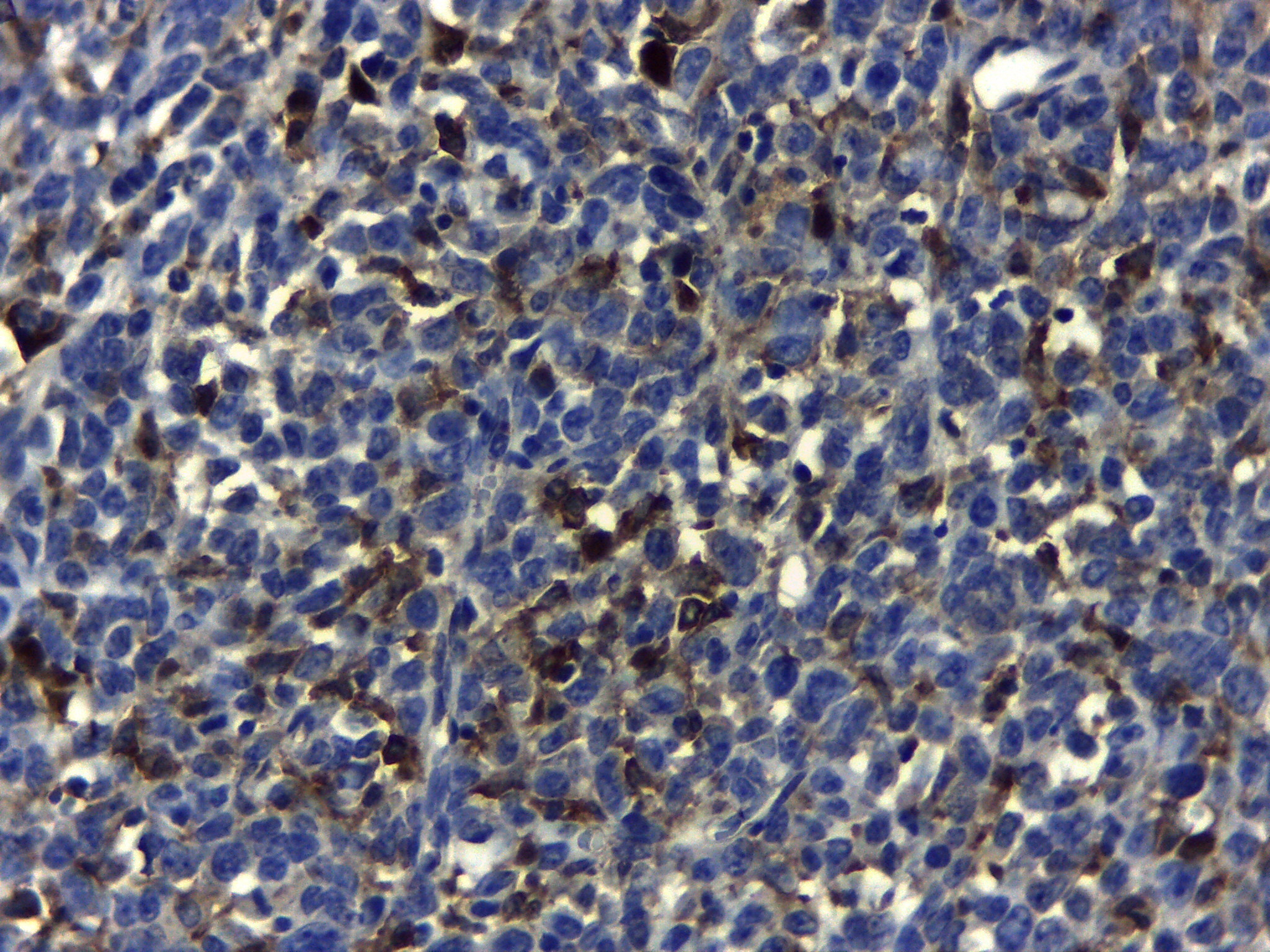

Supplement: S6 Fig — (ZIP) [file pone.0188960.s019.zip › HIF-1a IHC image CON/HIF-1a con6-4.jpg]

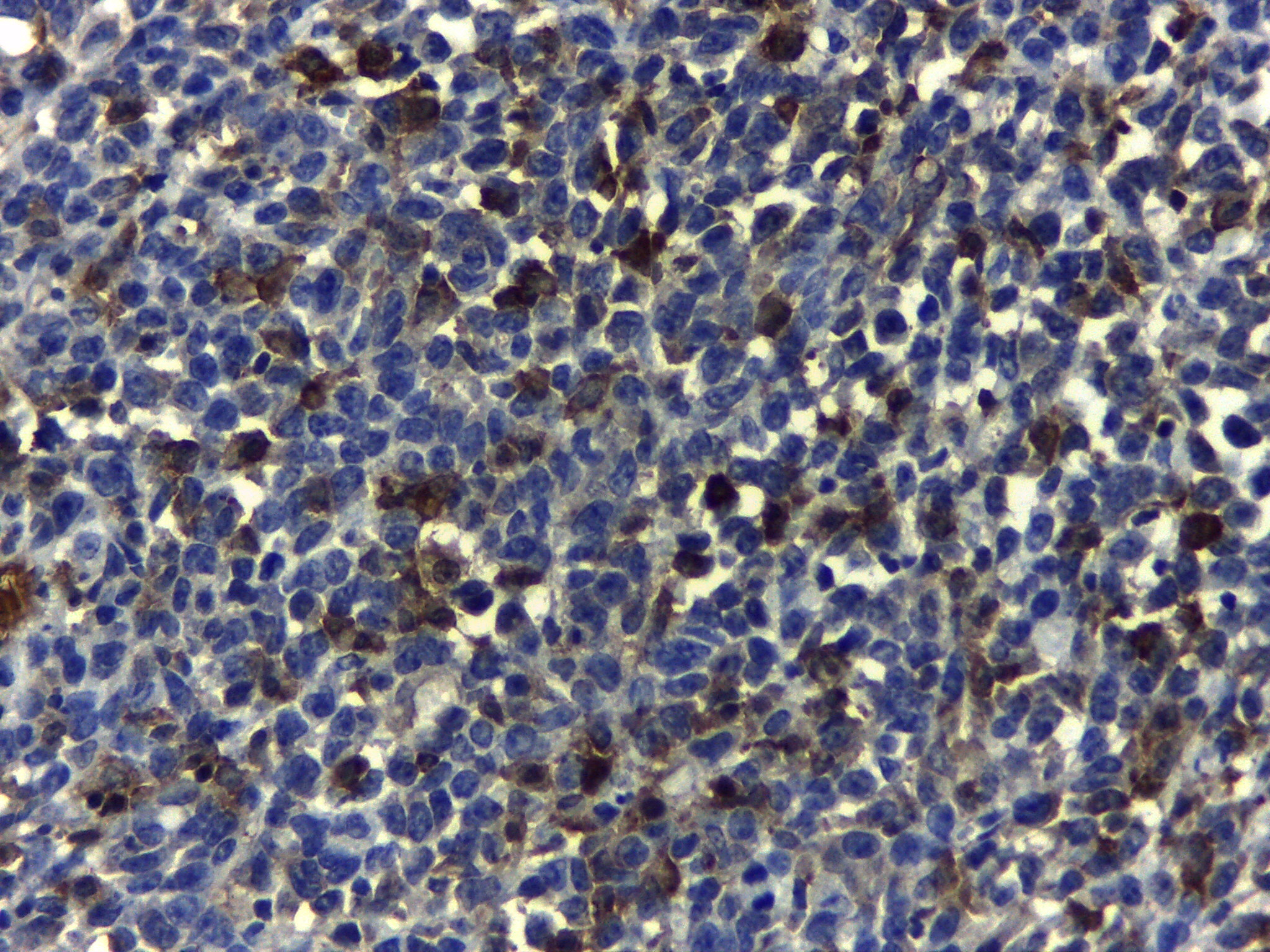

Supplement: S6 Fig — (ZIP) [file pone.0188960.s019.zip › HIF-1a IHC image CON/HIF-1a con6-5.jpg]

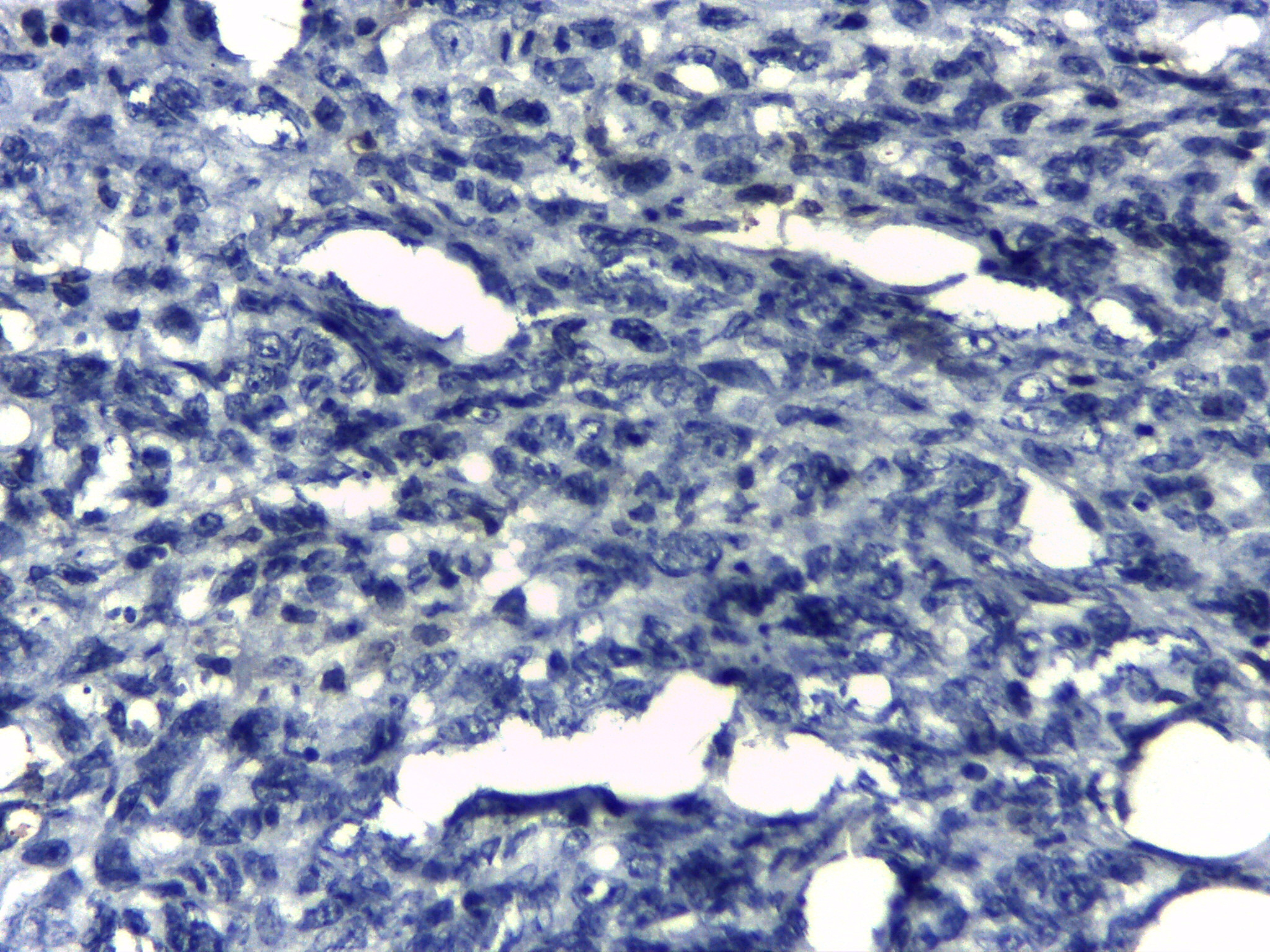

Supplement: S7 Fig — (ZIP) [file pone.0188960.s020.zip › HIF-1a IHC image BAC/HIF-1a bac1-1.jpg]

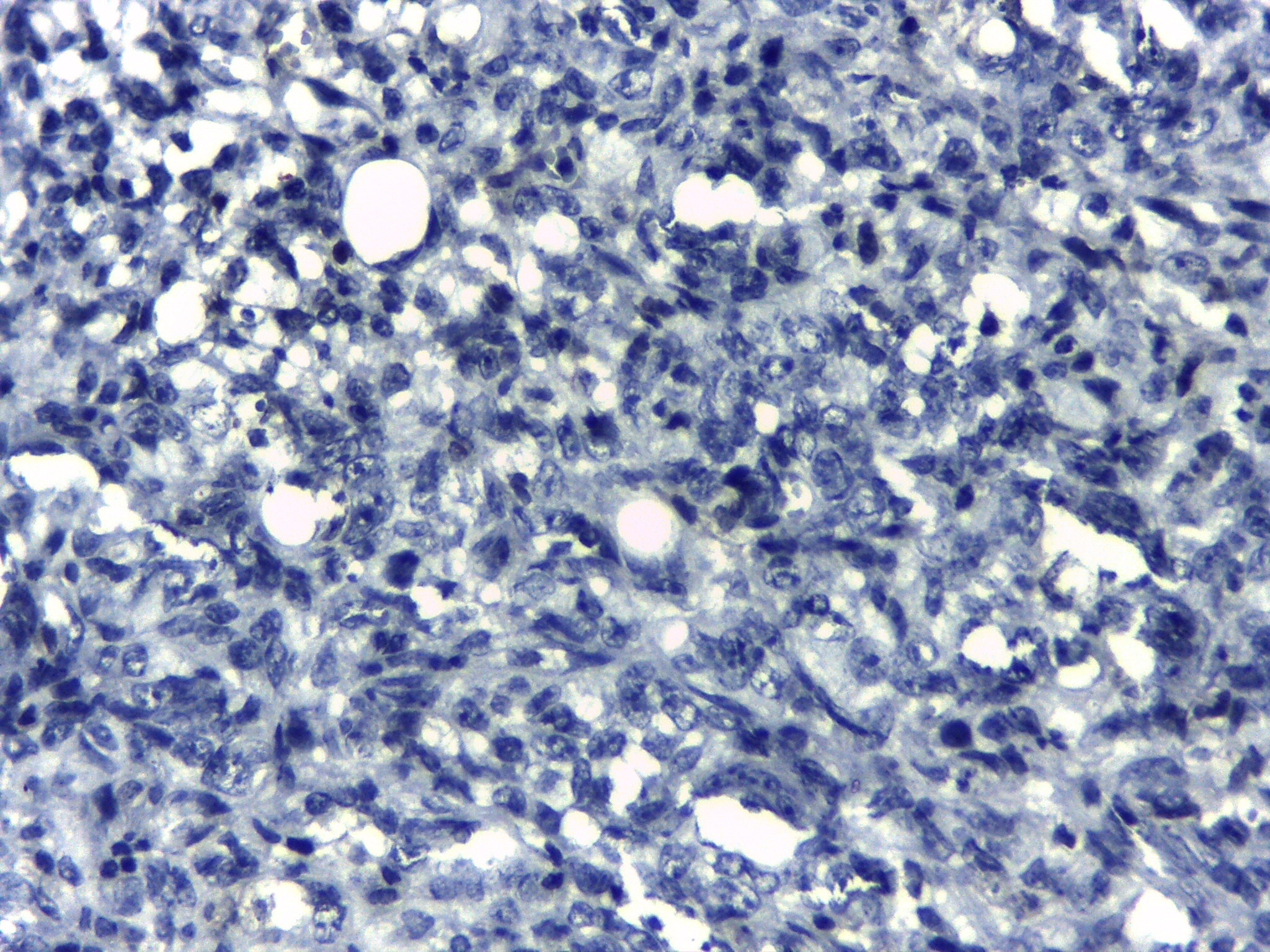

Supplement: S7 Fig — (ZIP) [file pone.0188960.s020.zip › HIF-1a IHC image BAC/HIF-1a bac1-2.jpg]

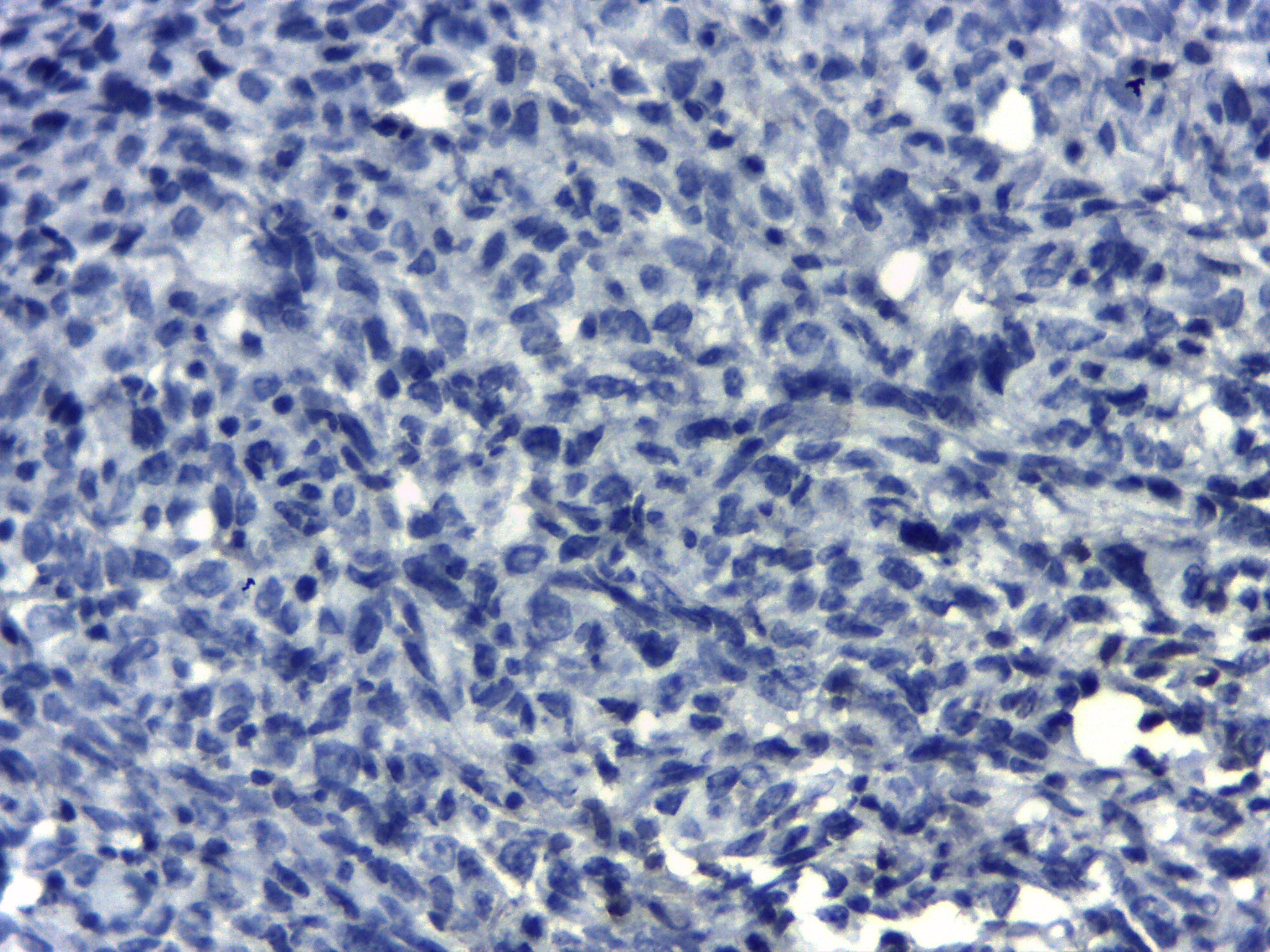

Supplement: S7 Fig — (ZIP) [file pone.0188960.s020.zip › HIF-1a IHC image BAC/HIF-1a bac1-3.jpg]

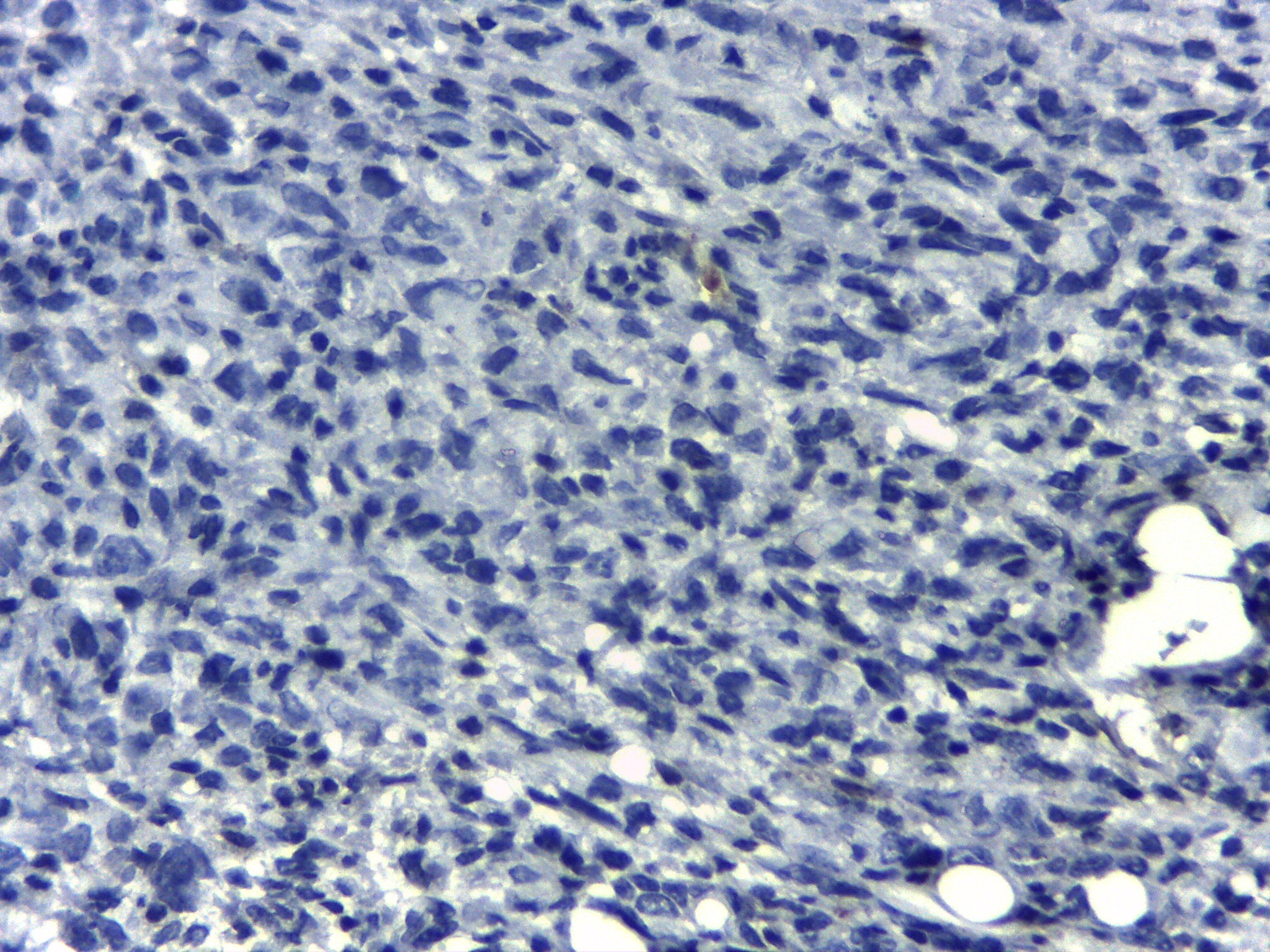

Supplement: S7 Fig — (ZIP) [file pone.0188960.s020.zip › HIF-1a IHC image BAC/HIF-1a bac1-4.jpg]

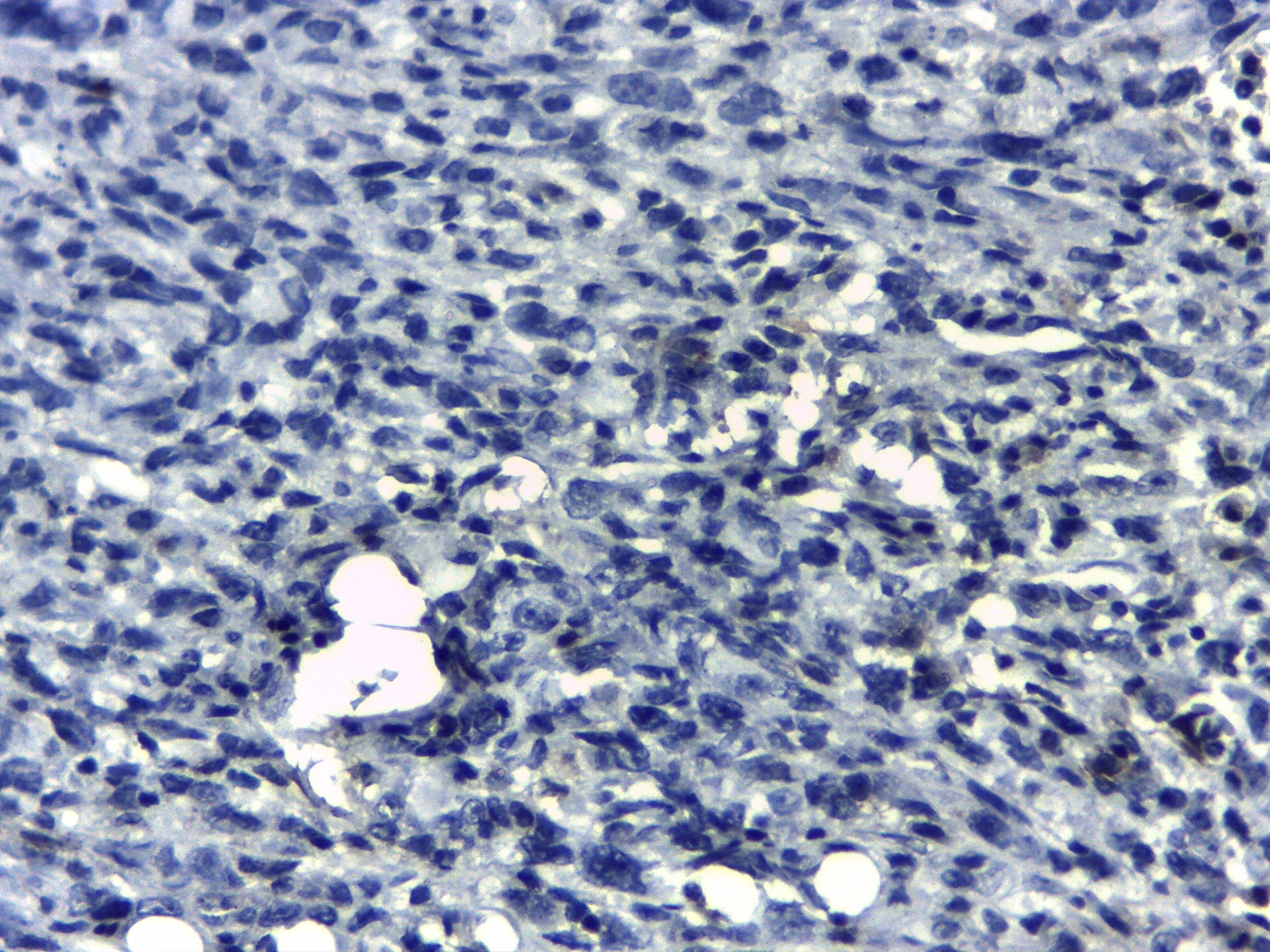

Supplement: S7 Fig — (ZIP) [file pone.0188960.s020.zip › HIF-1a IHC image BAC/HIF-1a bac1-5.jpg]

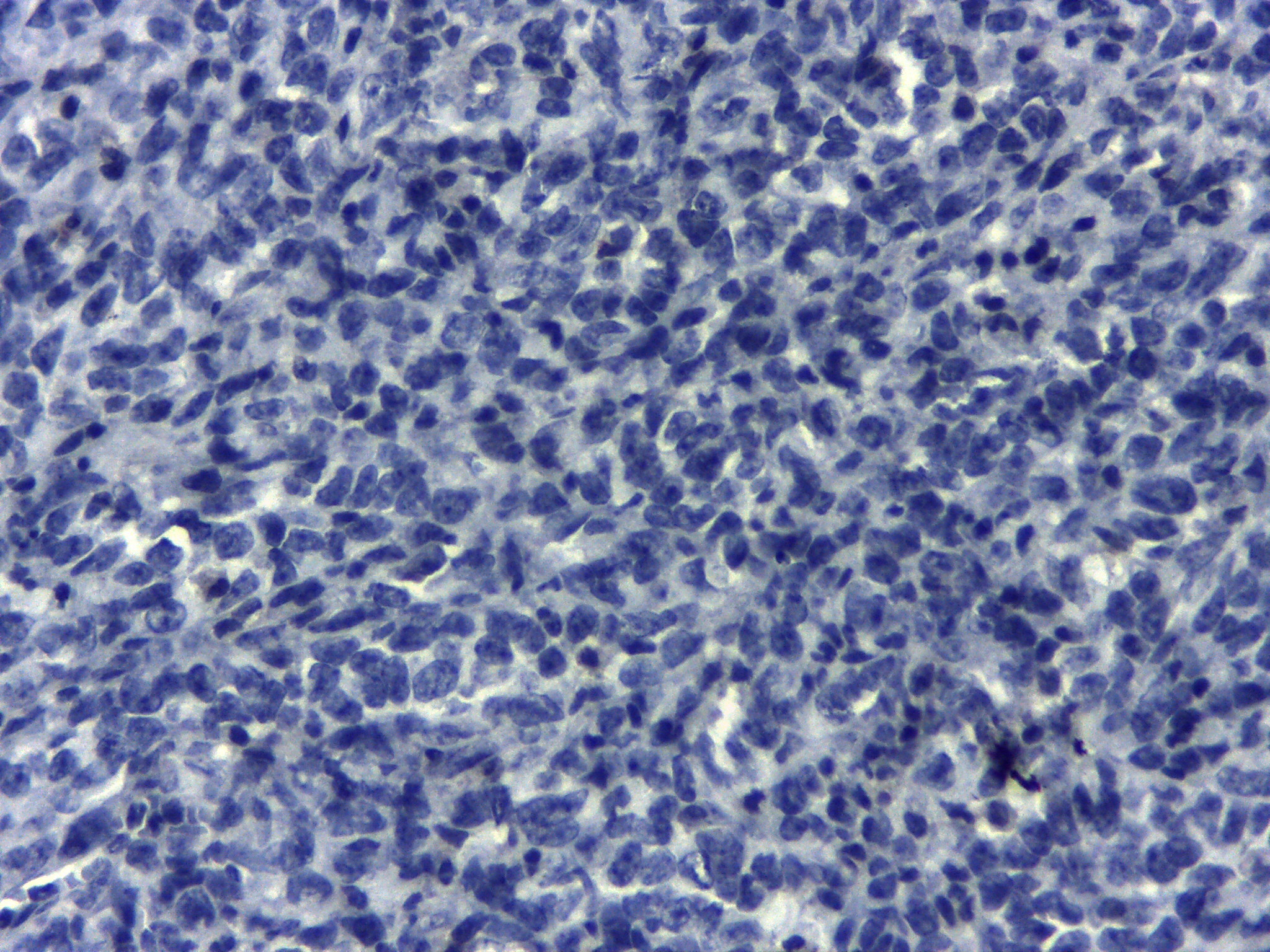

Supplement: S7 Fig — (ZIP) [file pone.0188960.s020.zip › HIF-1a IHC image BAC/HIF-1a bac2-1.jpg]

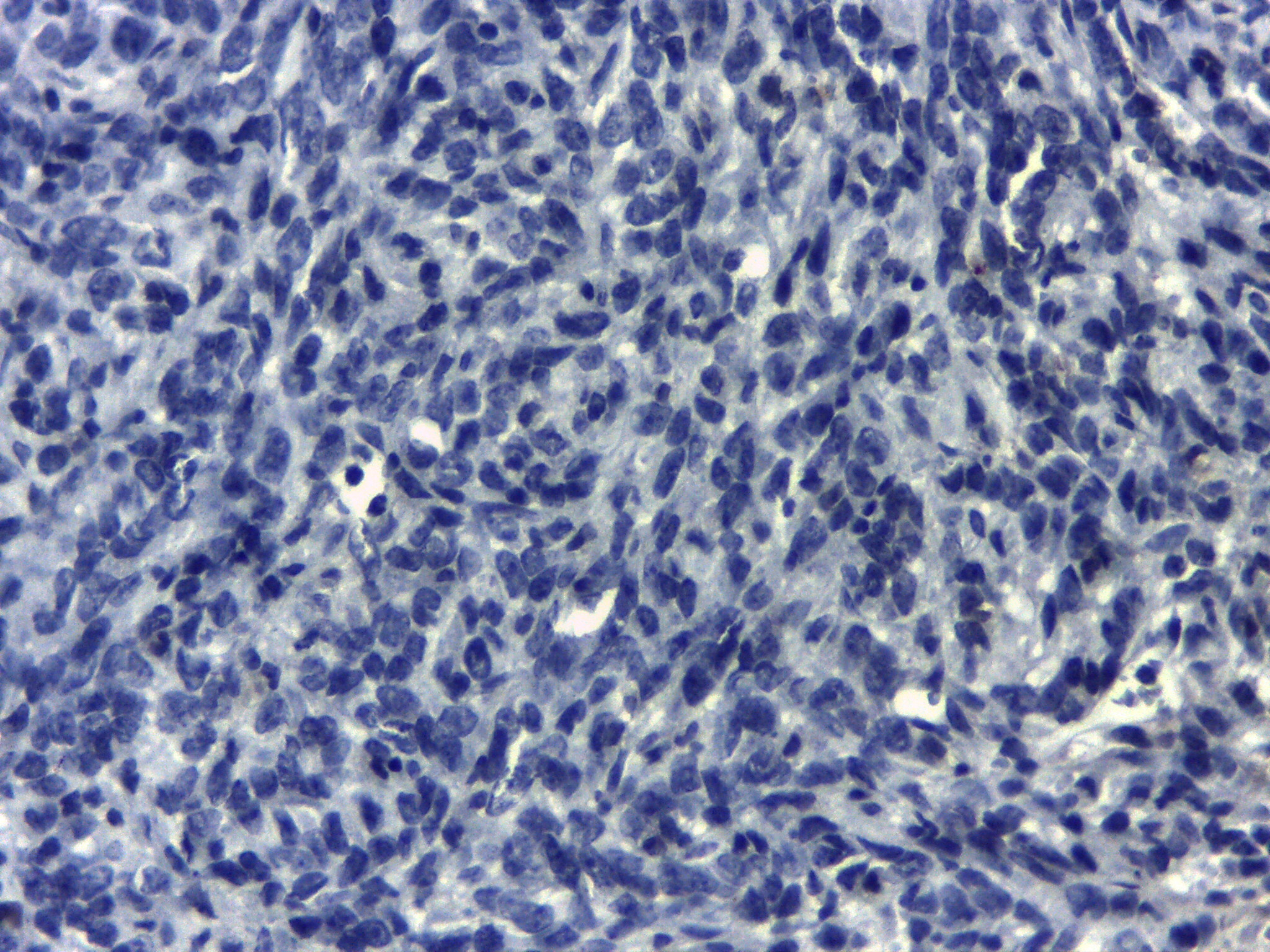

Supplement: S7 Fig — (ZIP) [file pone.0188960.s020.zip › HIF-1a IHC image BAC/HIF-1a bac2-2.jpg]

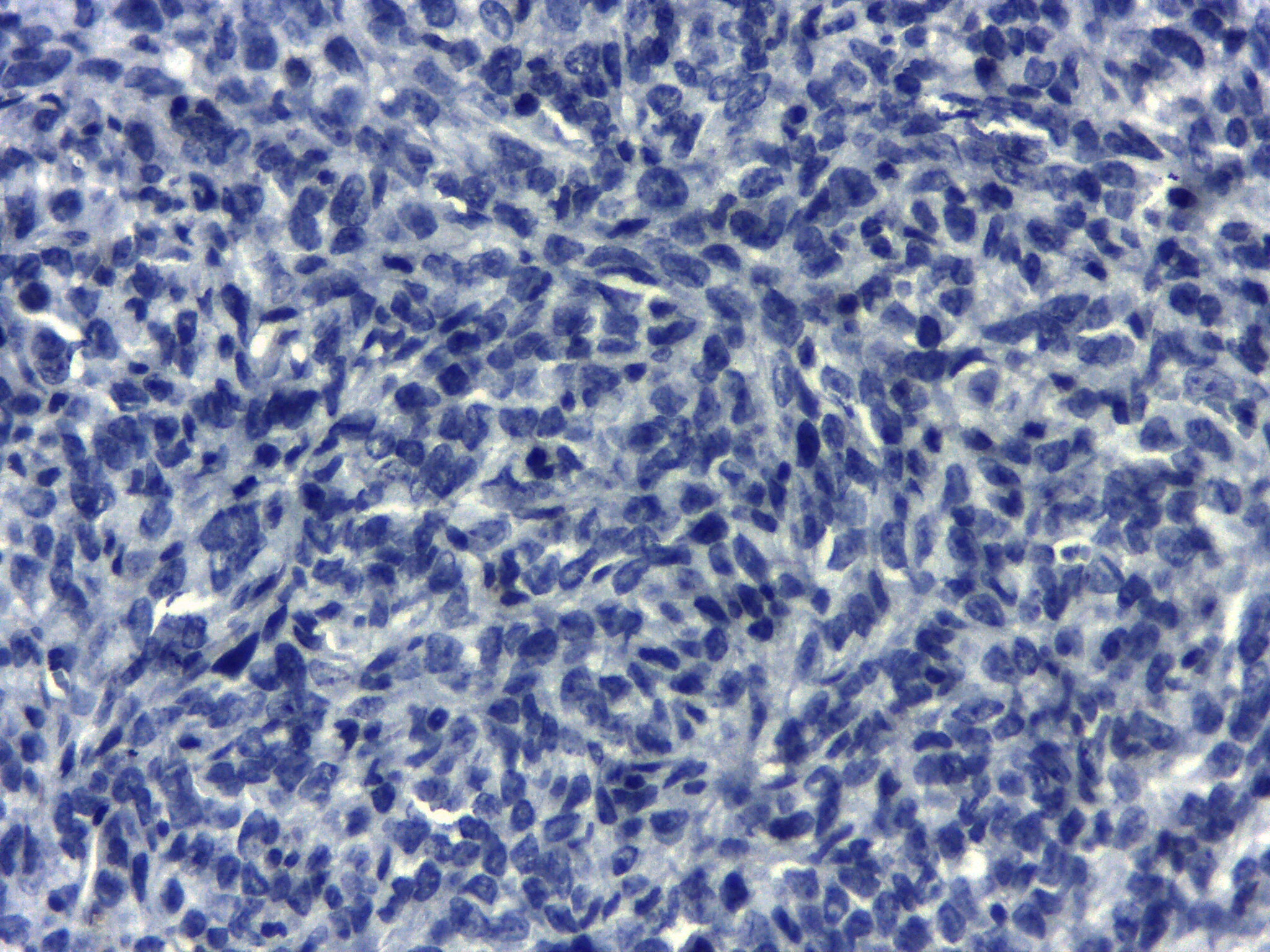

Supplement: S7 Fig — (ZIP) [file pone.0188960.s020.zip › HIF-1a IHC image BAC/HIF-1a bac2-3.jpg]

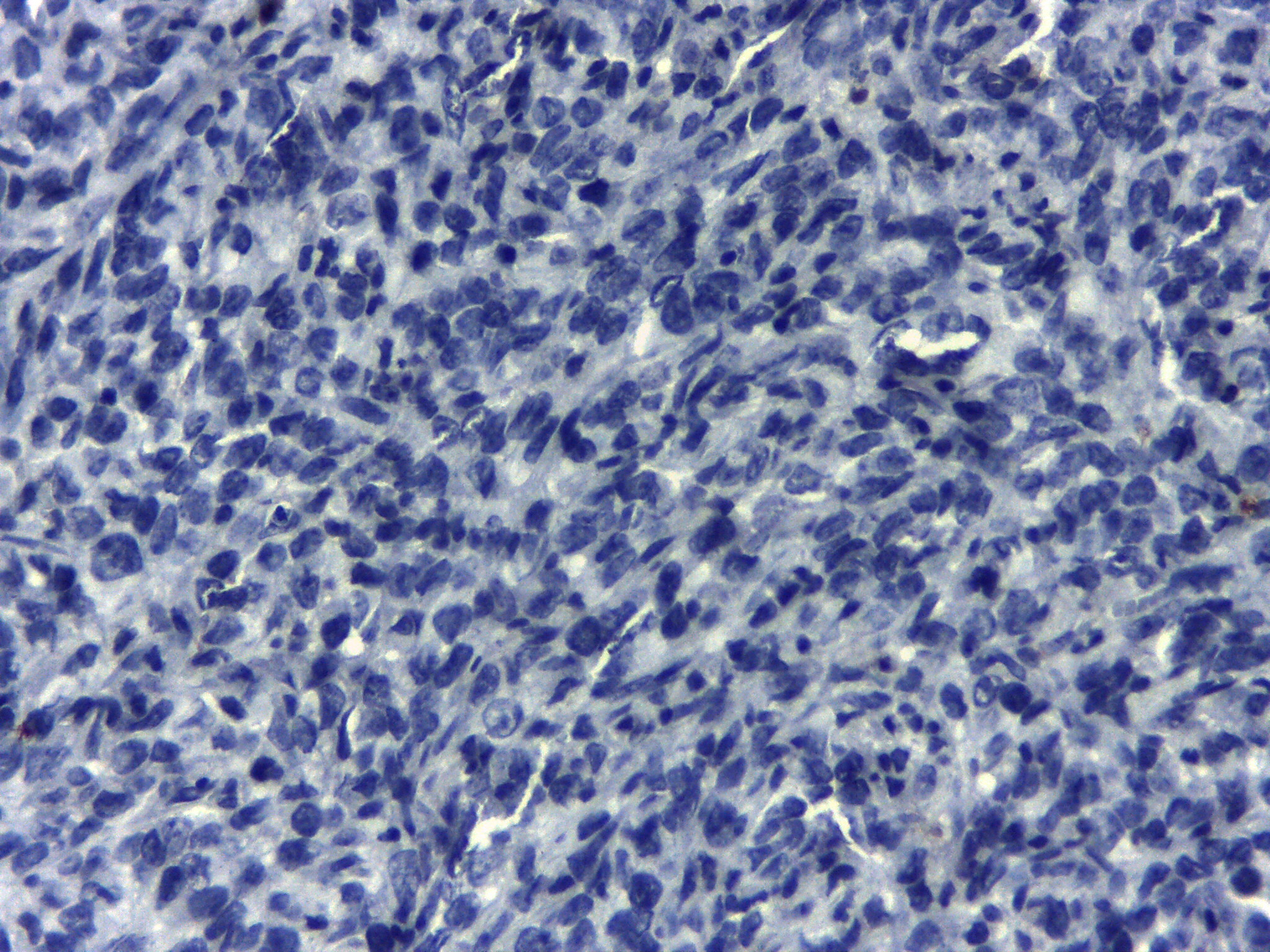

Supplement: S7 Fig — (ZIP) [file pone.0188960.s020.zip › HIF-1a IHC image BAC/HIF-1a bac2-4.jpg]

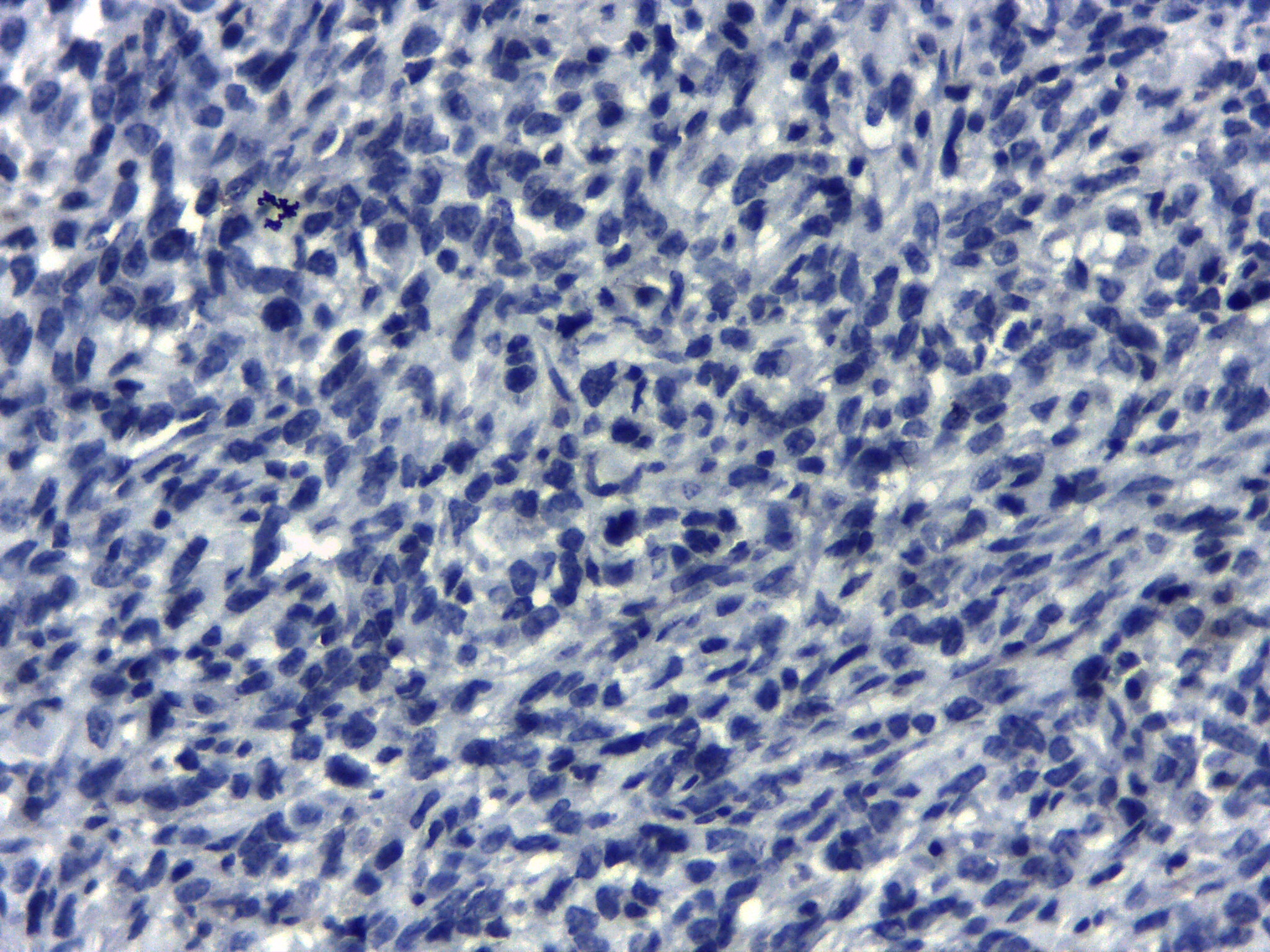

Supplement: S7 Fig — (ZIP) [file pone.0188960.s020.zip › HIF-1a IHC image BAC/HIF-1a bac2-5.jpg]

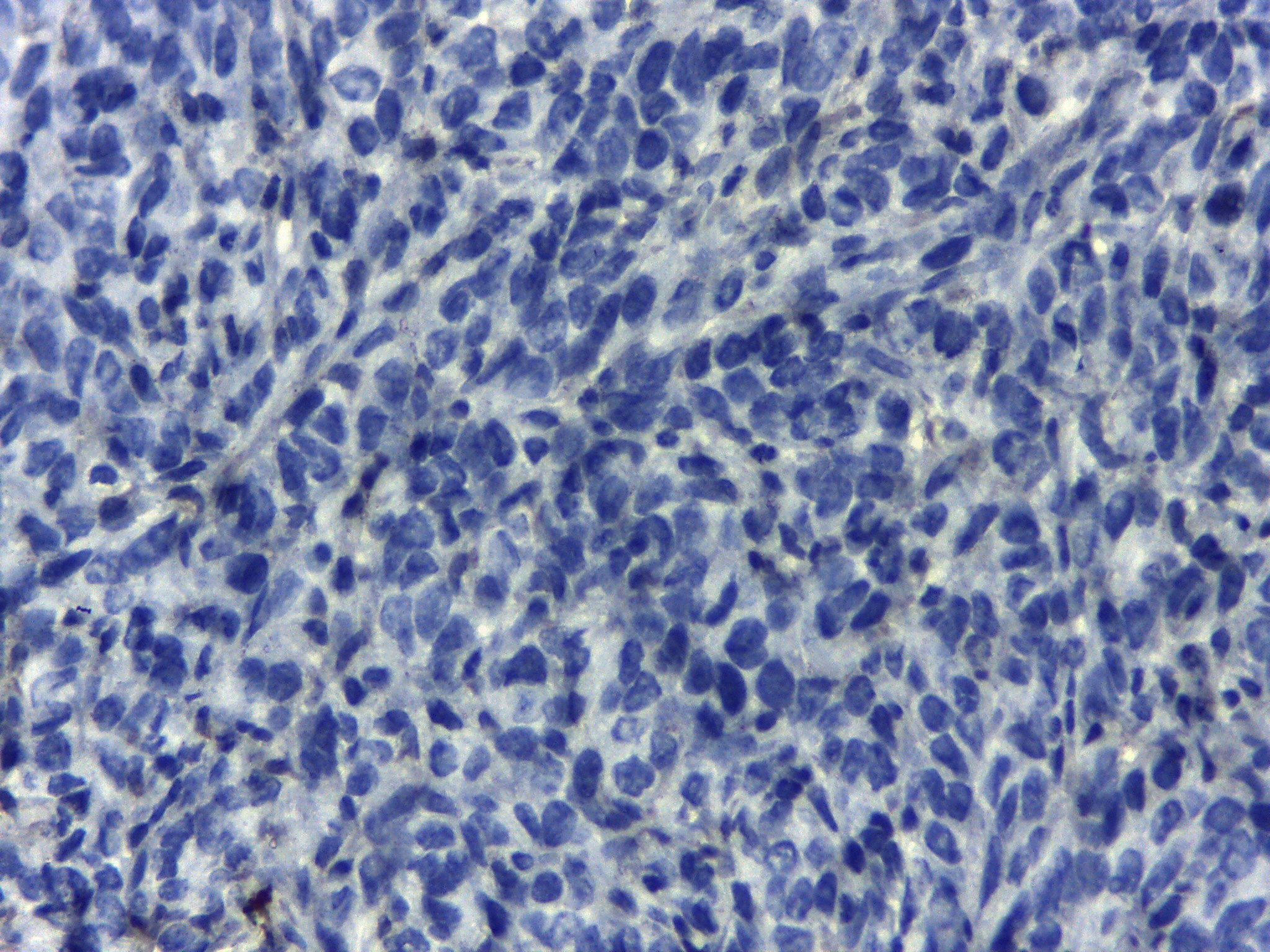

Supplement: S7 Fig — (ZIP) [file pone.0188960.s020.zip › HIF-1a IHC image BAC/HIF-1a bac3-1.jpg]

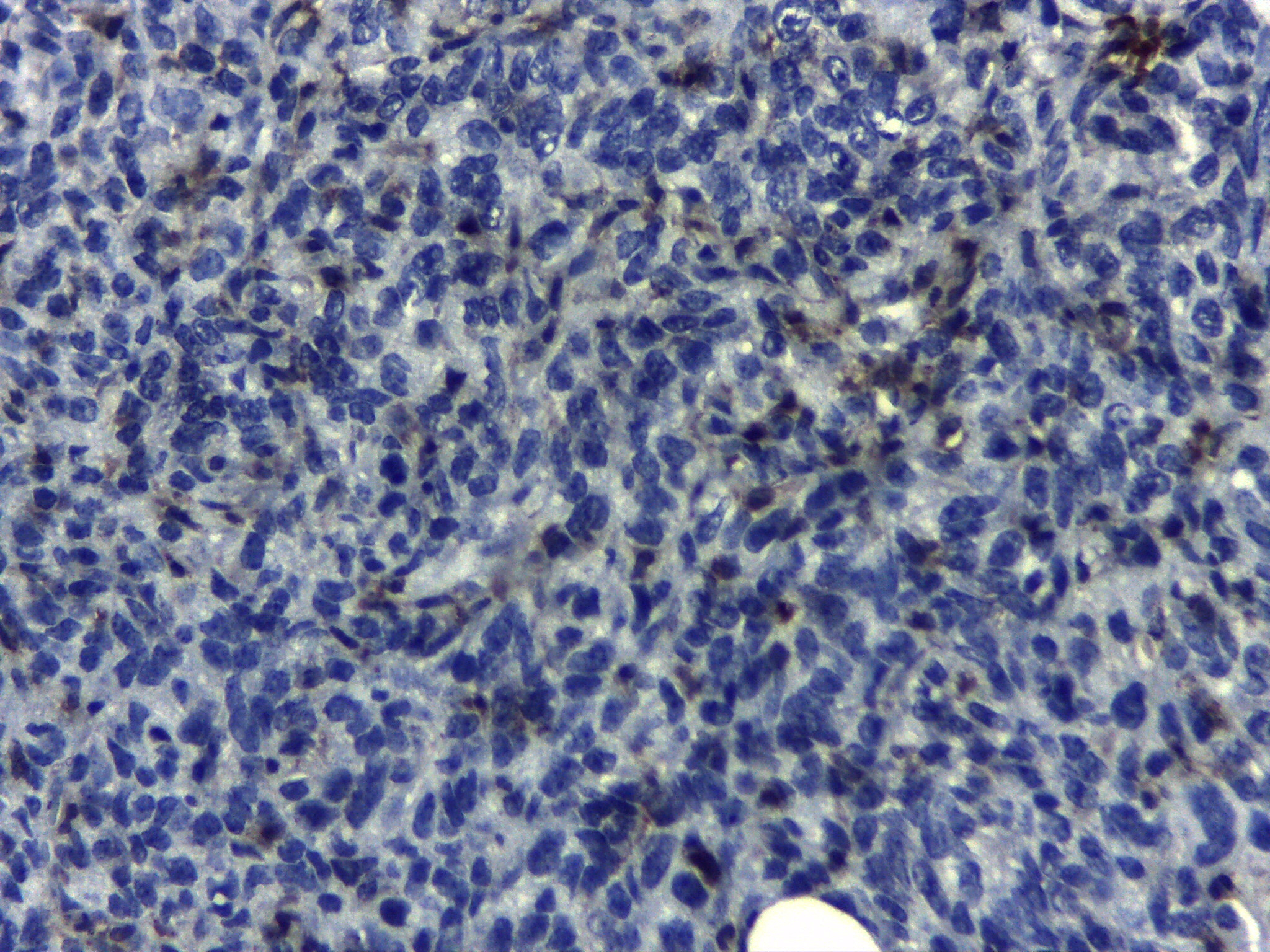

Supplement: S7 Fig — (ZIP) [file pone.0188960.s020.zip › HIF-1a IHC image BAC/HIF-1a bac3-2.jpg]

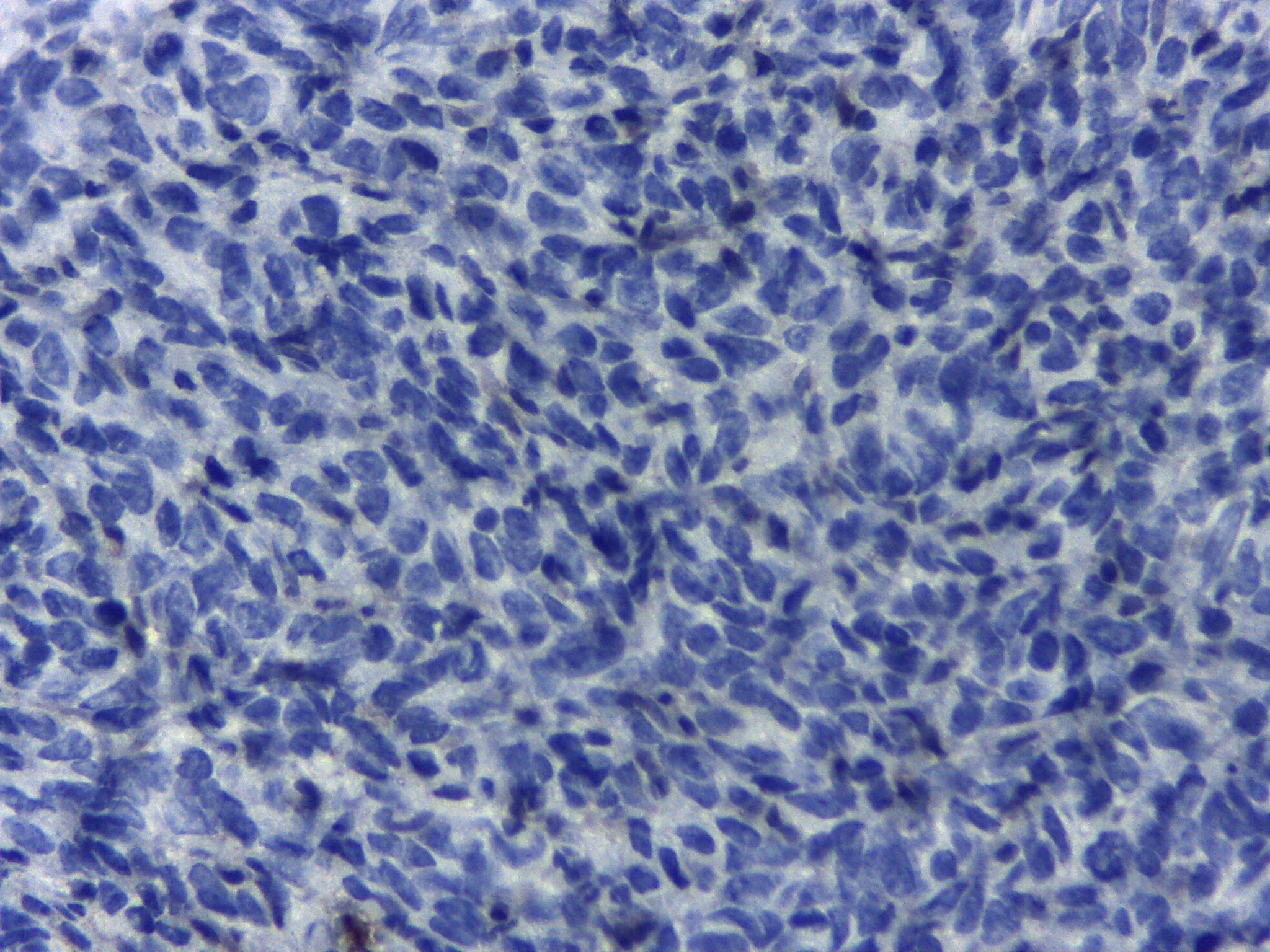

Supplement: S7 Fig — (ZIP) [file pone.0188960.s020.zip › HIF-1a IHC image BAC/HIF-1a bac3-3.jpg]

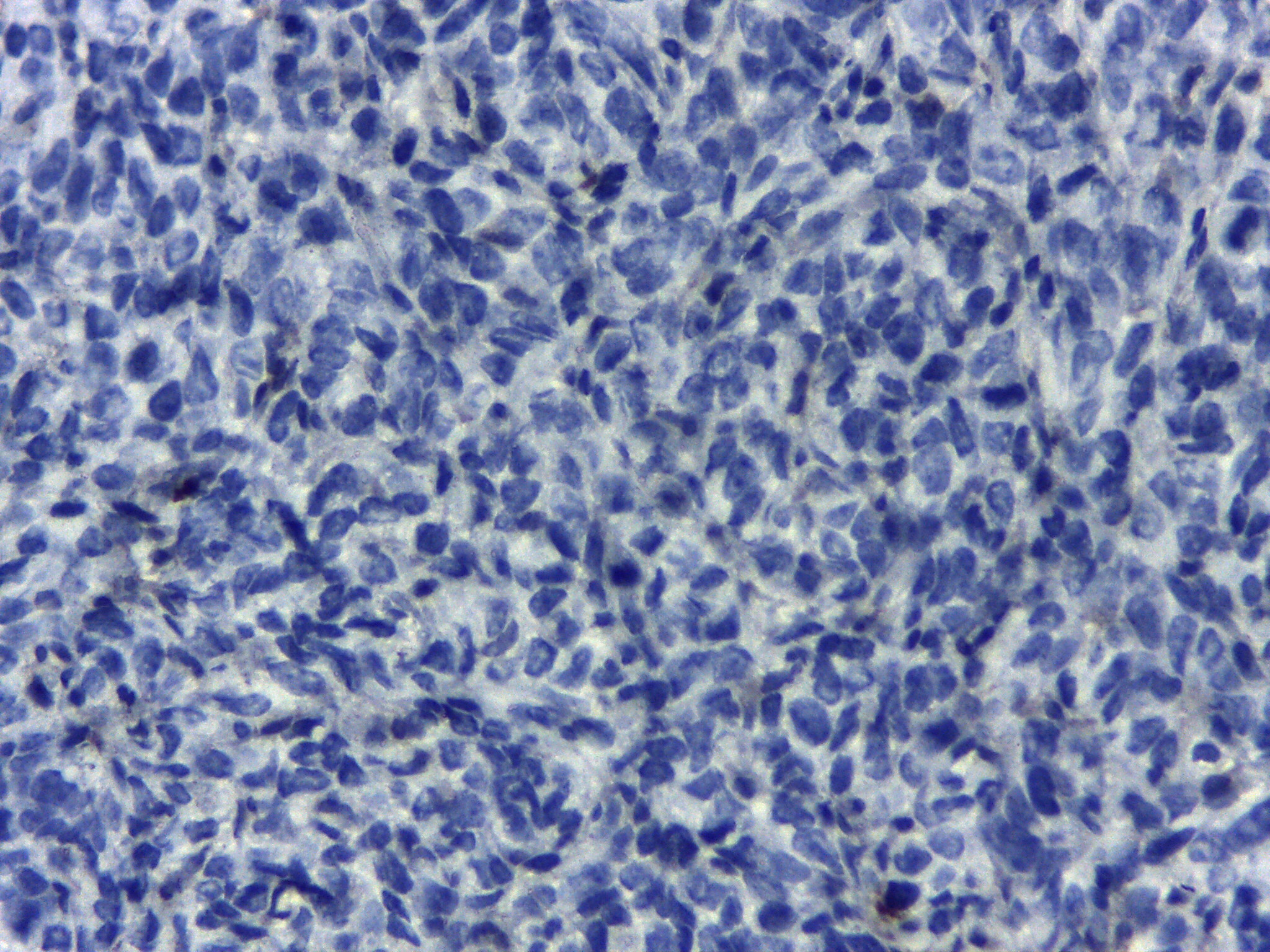

Supplement: S7 Fig — (ZIP) [file pone.0188960.s020.zip › HIF-1a IHC image BAC/HIF-1a bac3-4.jpg]

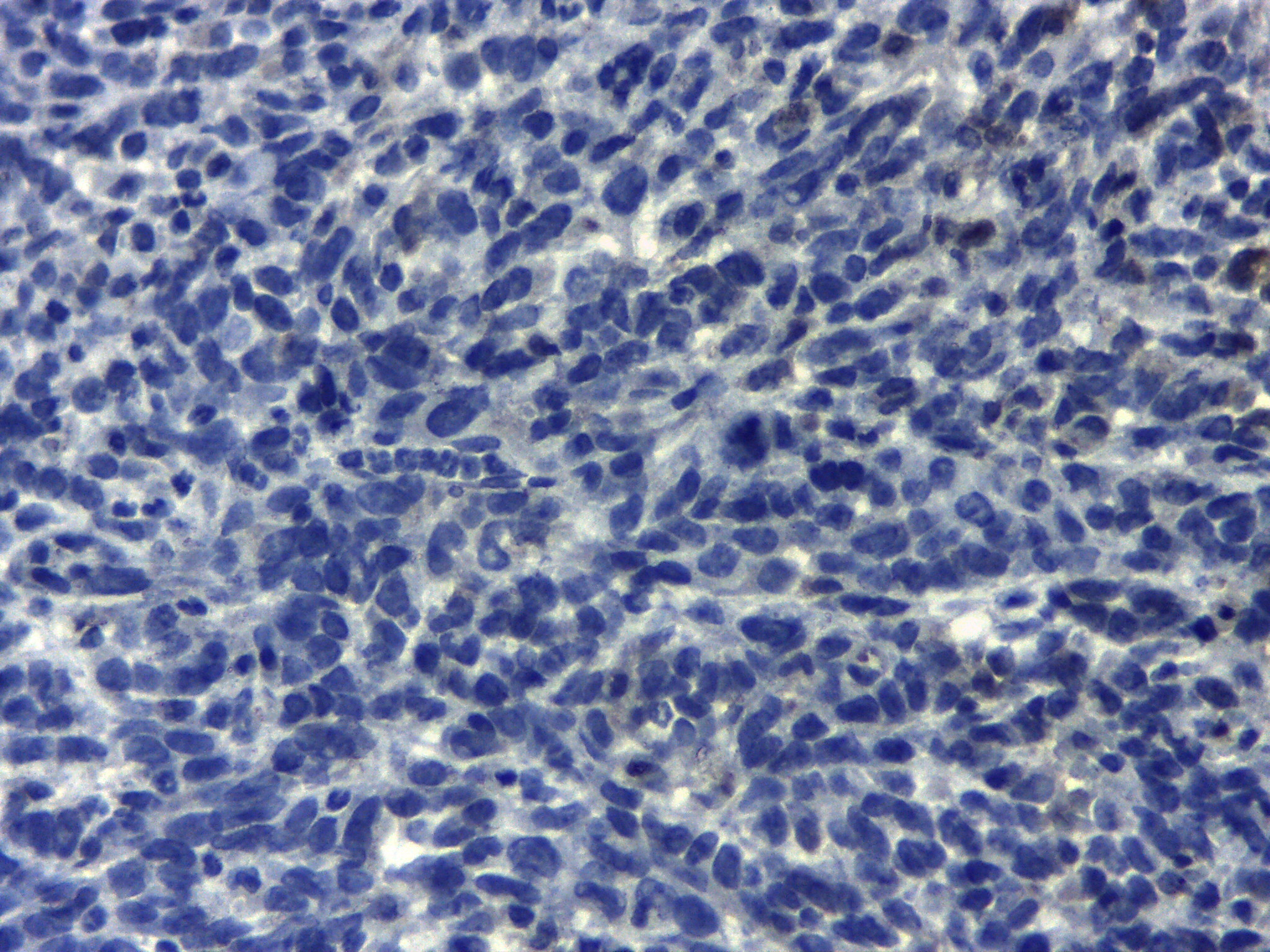

Supplement: S7 Fig — (ZIP) [file pone.0188960.s020.zip › HIF-1a IHC image BAC/HIF-1a bac3-5.jpg]

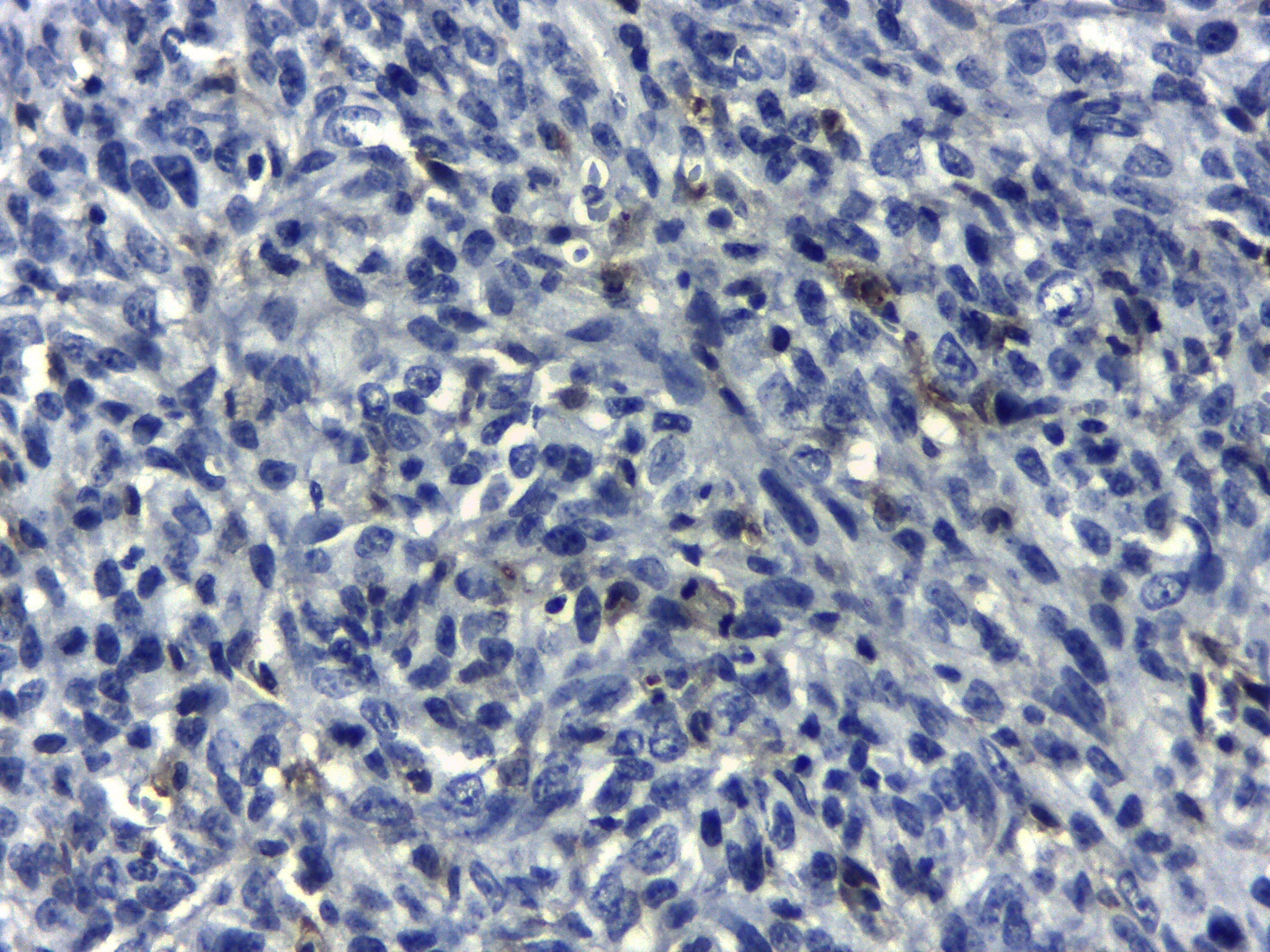

Supplement: S7 Fig — (ZIP) [file pone.0188960.s020.zip › HIF-1a IHC image BAC/HIF-1a bac4-1.jpg]

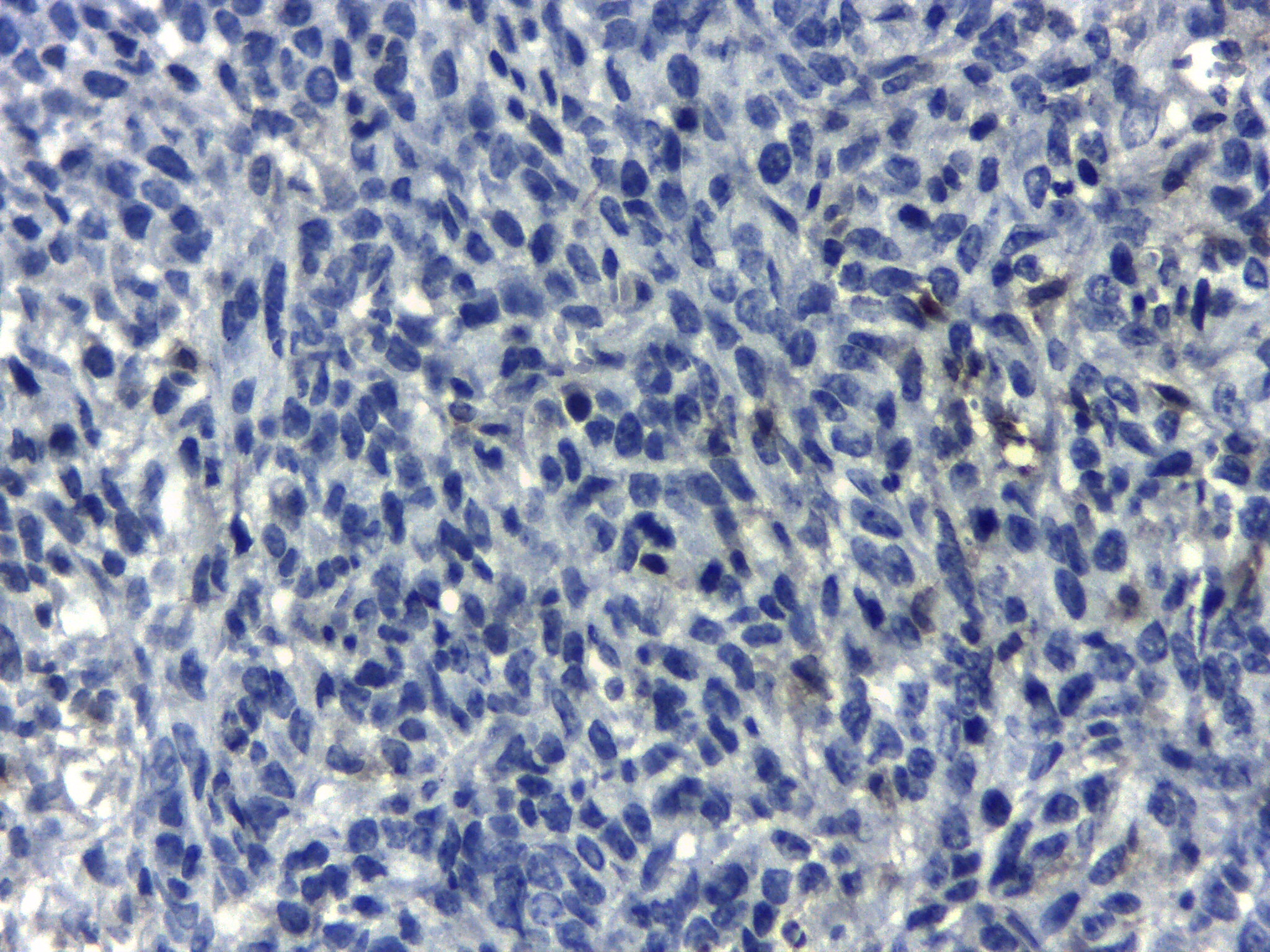

Supplement: S7 Fig — (ZIP) [file pone.0188960.s020.zip › HIF-1a IHC image BAC/HIF-1a bac4-2.jpg]

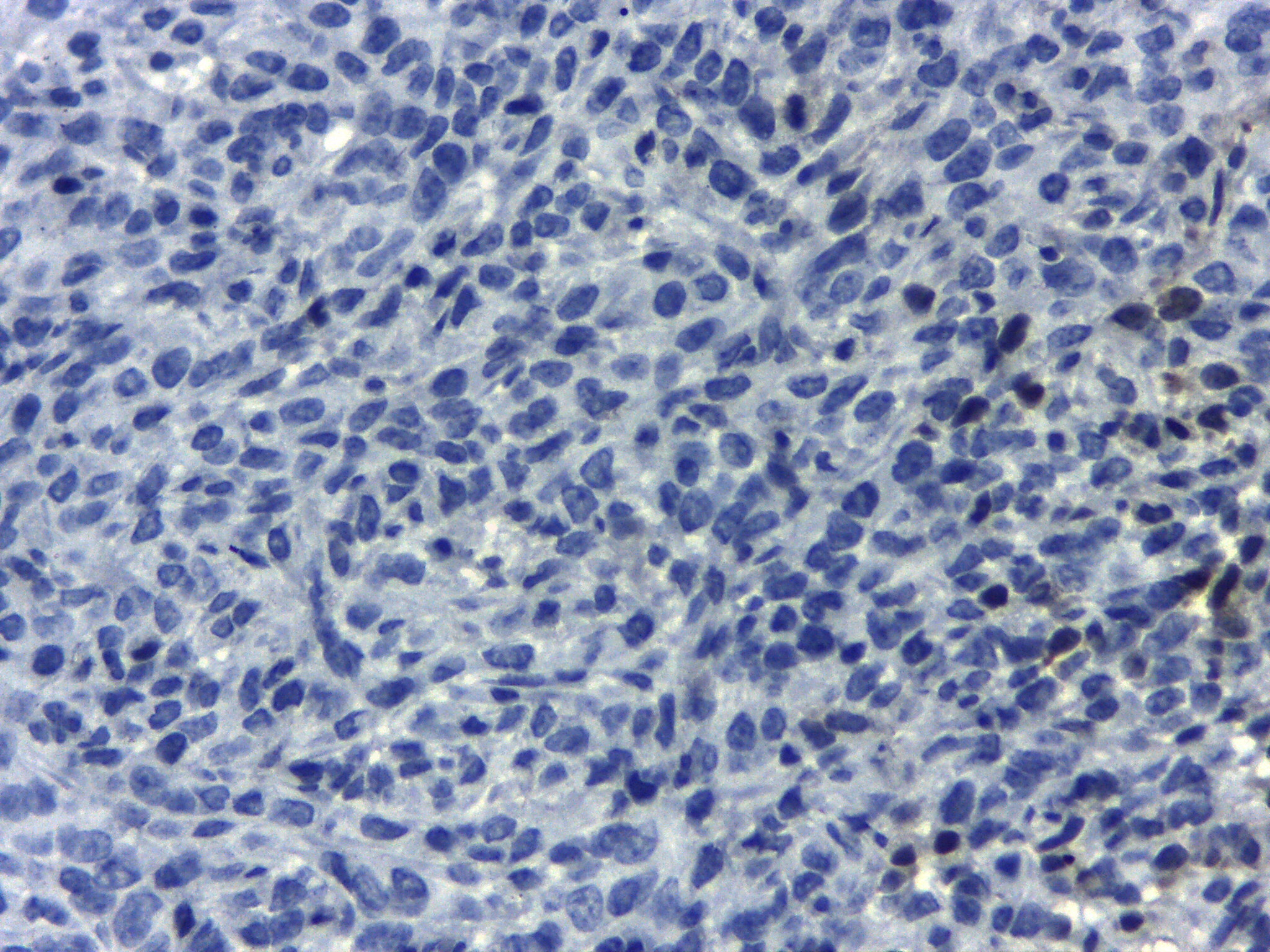

Supplement: S7 Fig — (ZIP) [file pone.0188960.s020.zip › HIF-1a IHC image BAC/HIF-1a bac4-3.jpg]

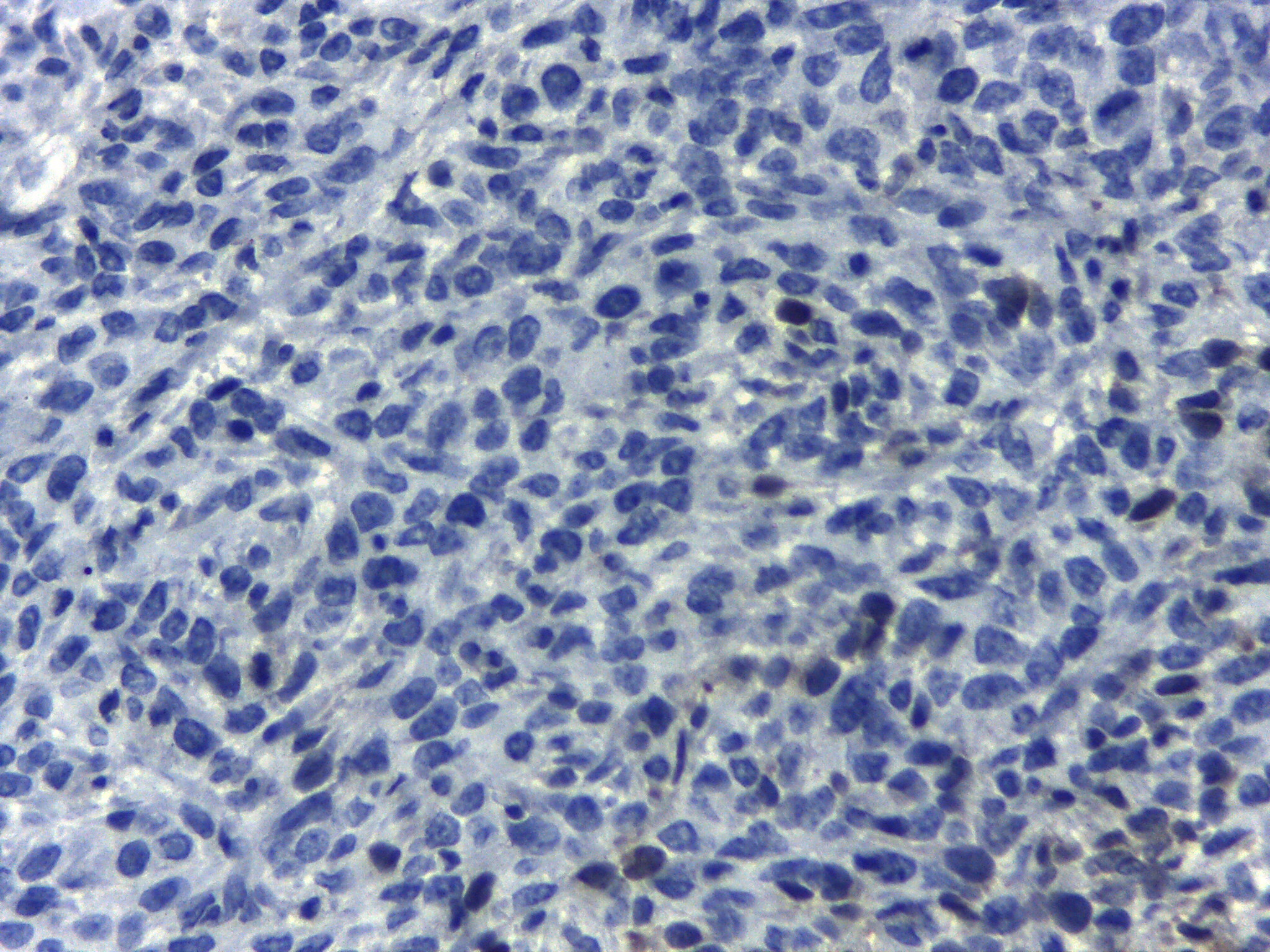

Supplement: S7 Fig — (ZIP) [file pone.0188960.s020.zip › HIF-1a IHC image BAC/HIF-1a bac4-4.jpg]

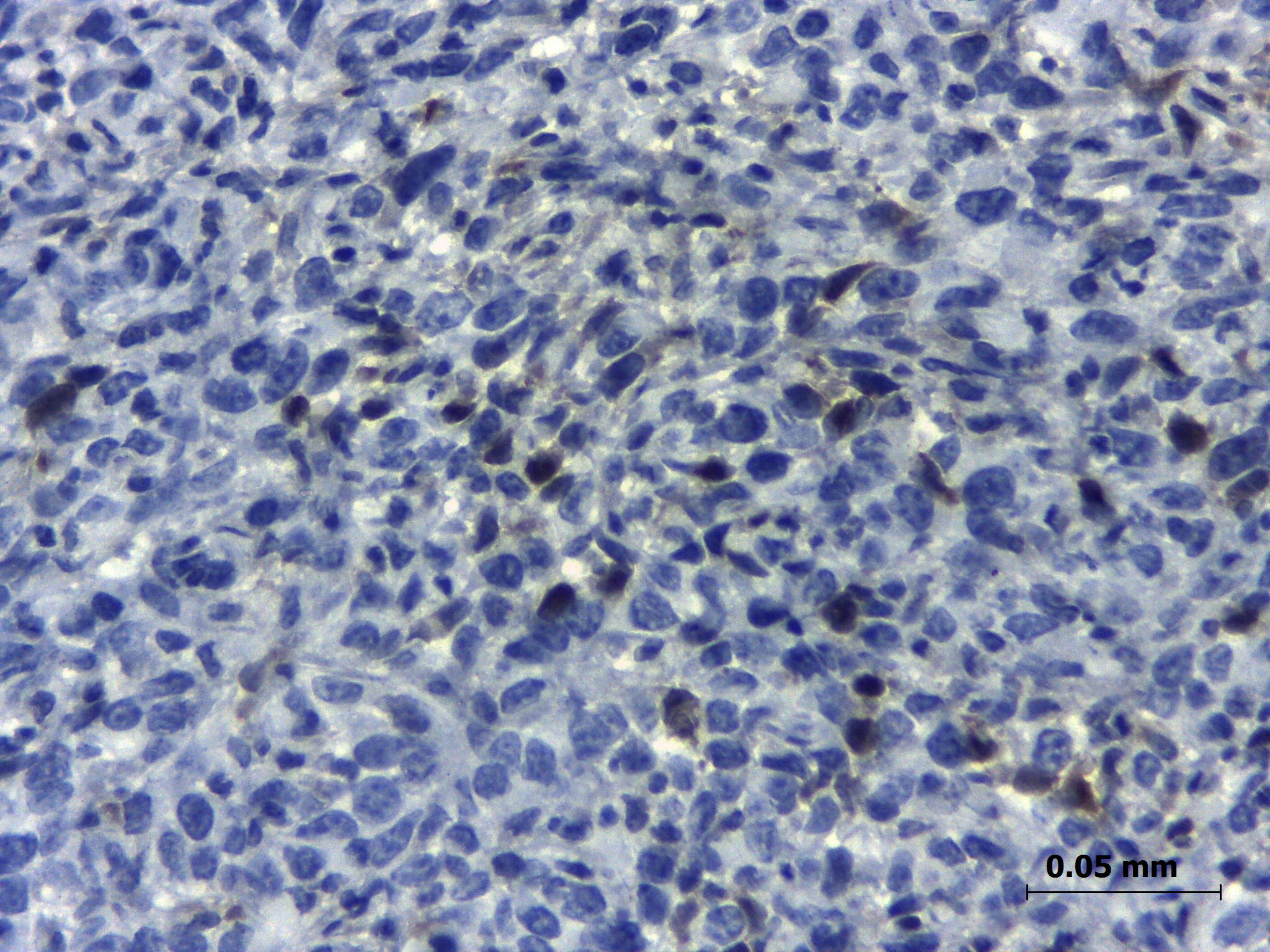

Supplement: S7 Fig — (ZIP) [file pone.0188960.s020.zip › HIF-1a IHC image BAC/HIF-1a bac4-5.jpg]

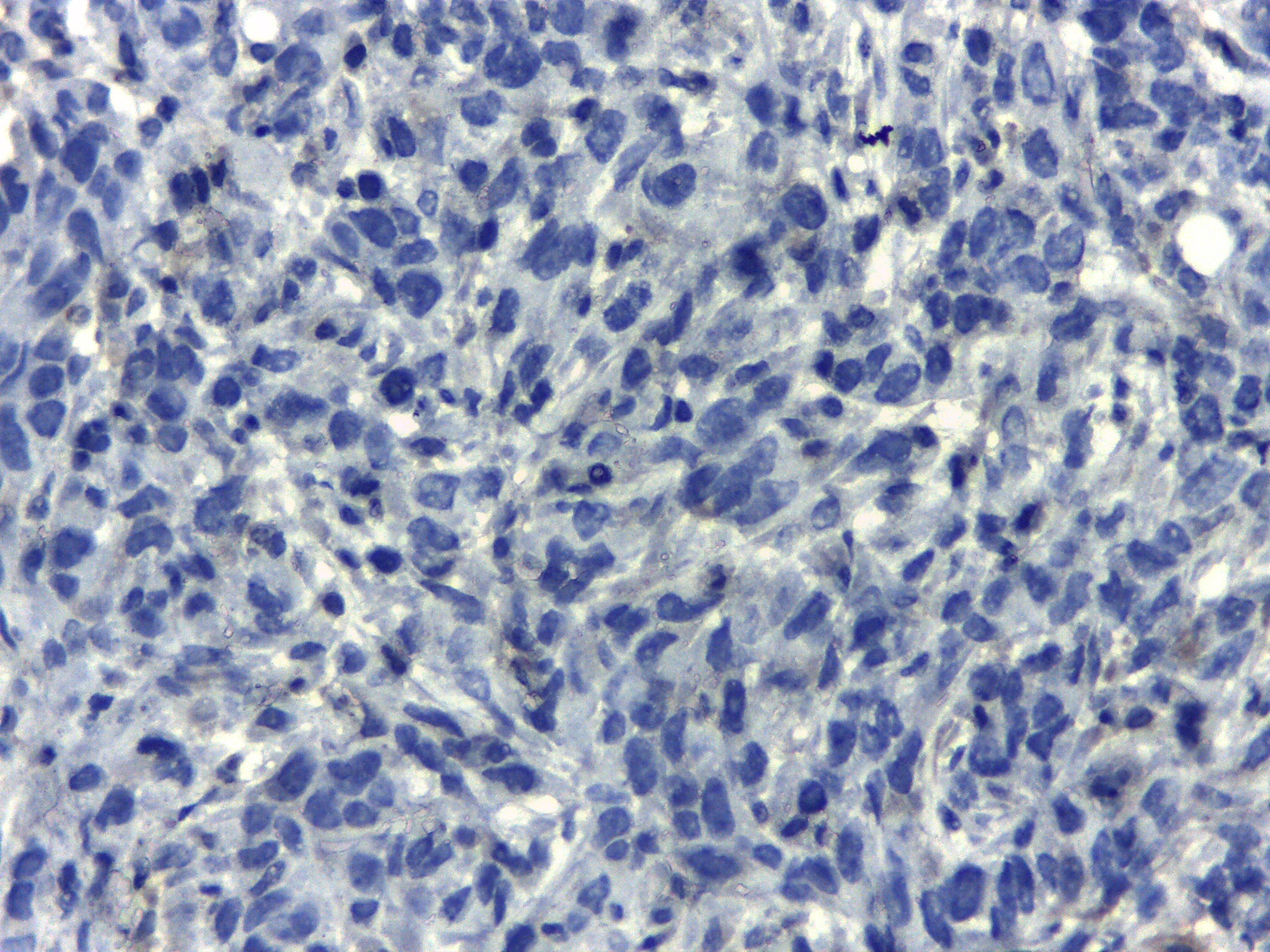

Supplement: S7 Fig — (ZIP) [file pone.0188960.s020.zip › HIF-1a IHC image BAC/HIF-1a bac5-1.jpg]

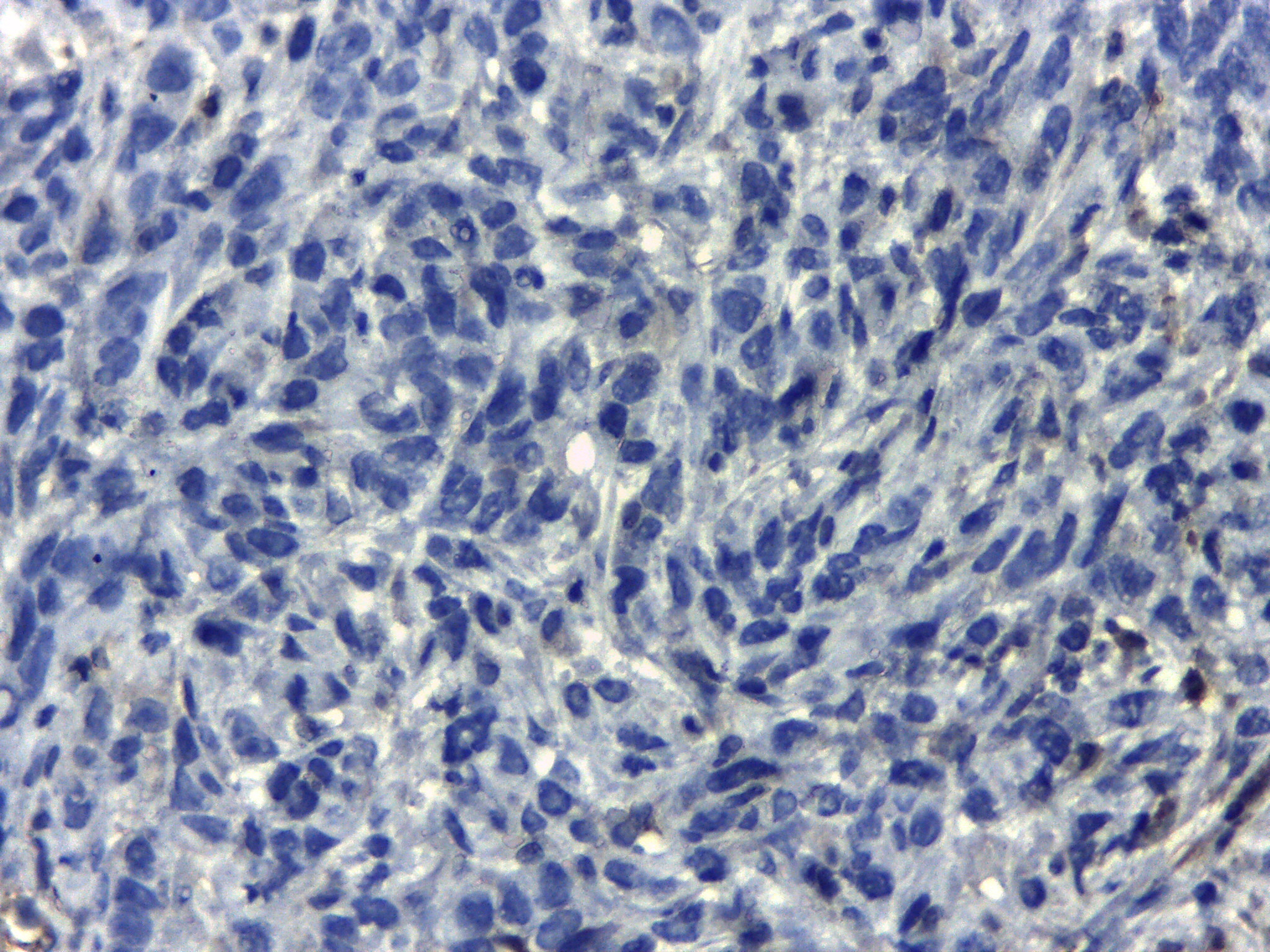

Supplement: S7 Fig — (ZIP) [file pone.0188960.s020.zip › HIF-1a IHC image BAC/HIF-1a bac5-2.jpg]

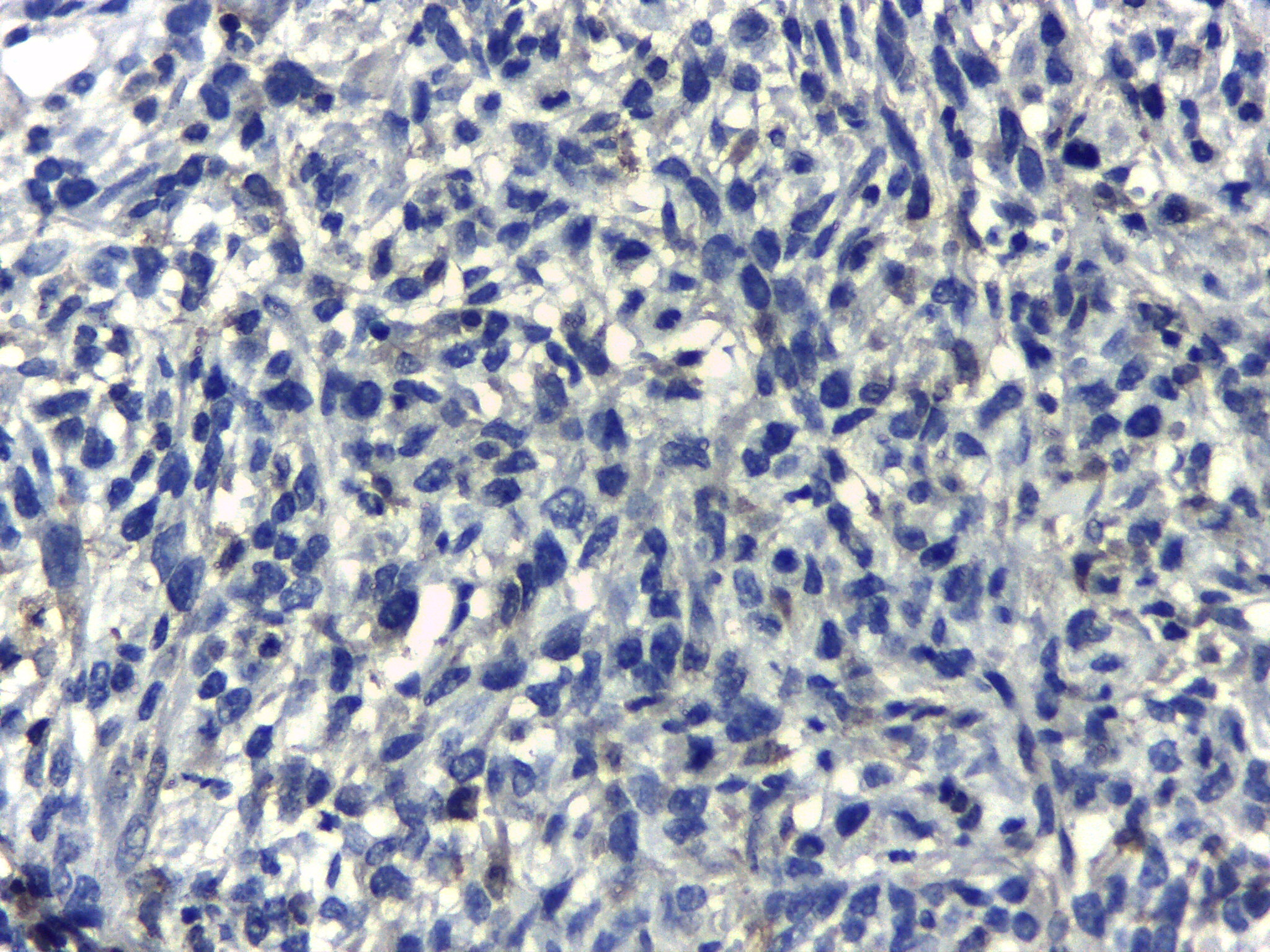

Supplement: S7 Fig — (ZIP) [file pone.0188960.s020.zip › HIF-1a IHC image BAC/HIF-1a bac5-3.jpg]

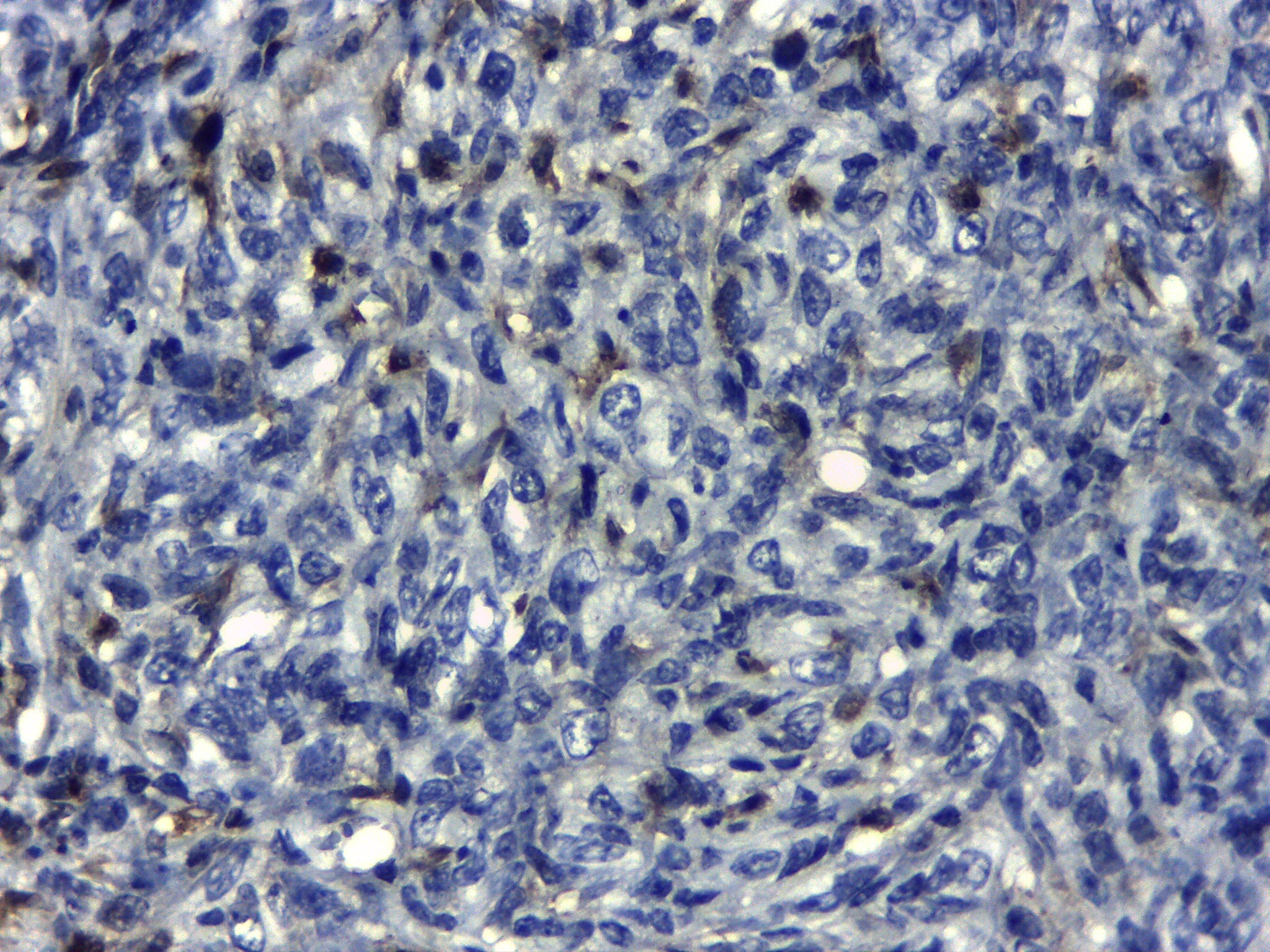

Supplement: S7 Fig — (ZIP) [file pone.0188960.s020.zip › HIF-1a IHC image BAC/HIF-1a bac5-4.jpg]

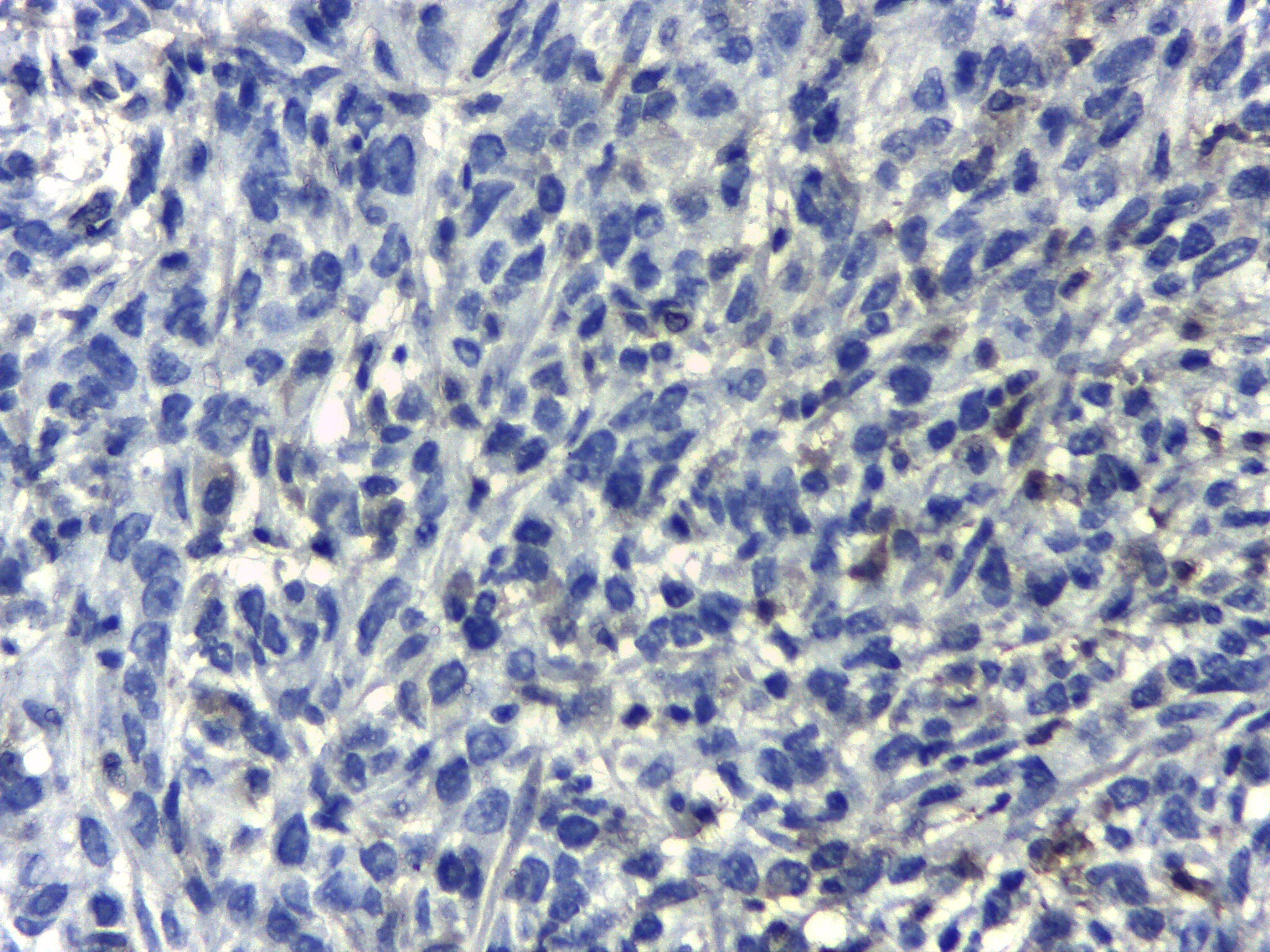

Supplement: S7 Fig — (ZIP) [file pone.0188960.s020.zip › HIF-1a IHC image BAC/HIF-1a bac5-5.jpg]

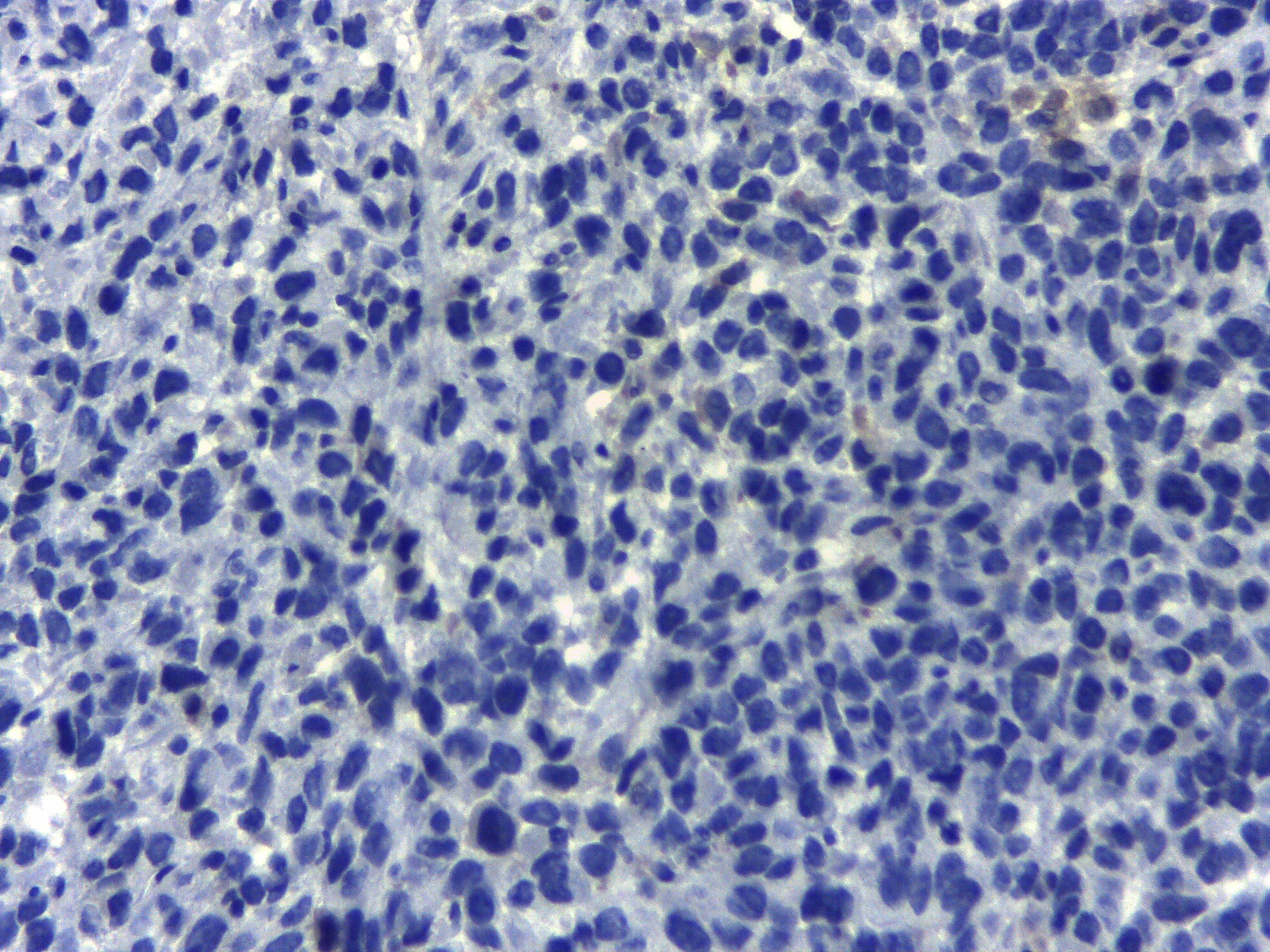

Supplement: S7 Fig — (ZIP) [file pone.0188960.s020.zip › HIF-1a IHC image BAC/HIF-1a bac6-1.jpg]

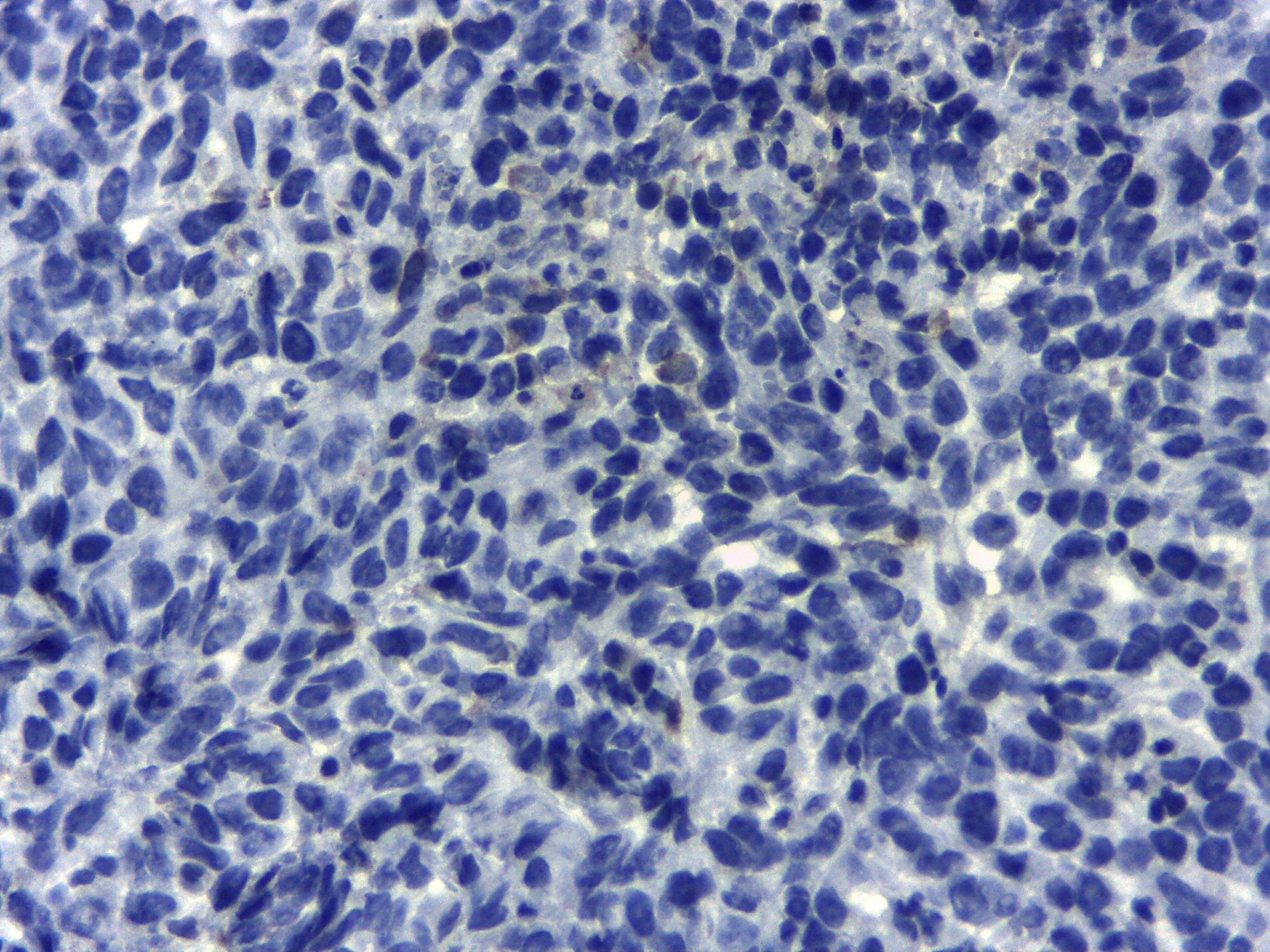

Supplement: S7 Fig — (ZIP) [file pone.0188960.s020.zip › HIF-1a IHC image BAC/HIF-1a bac6-2.jpg]

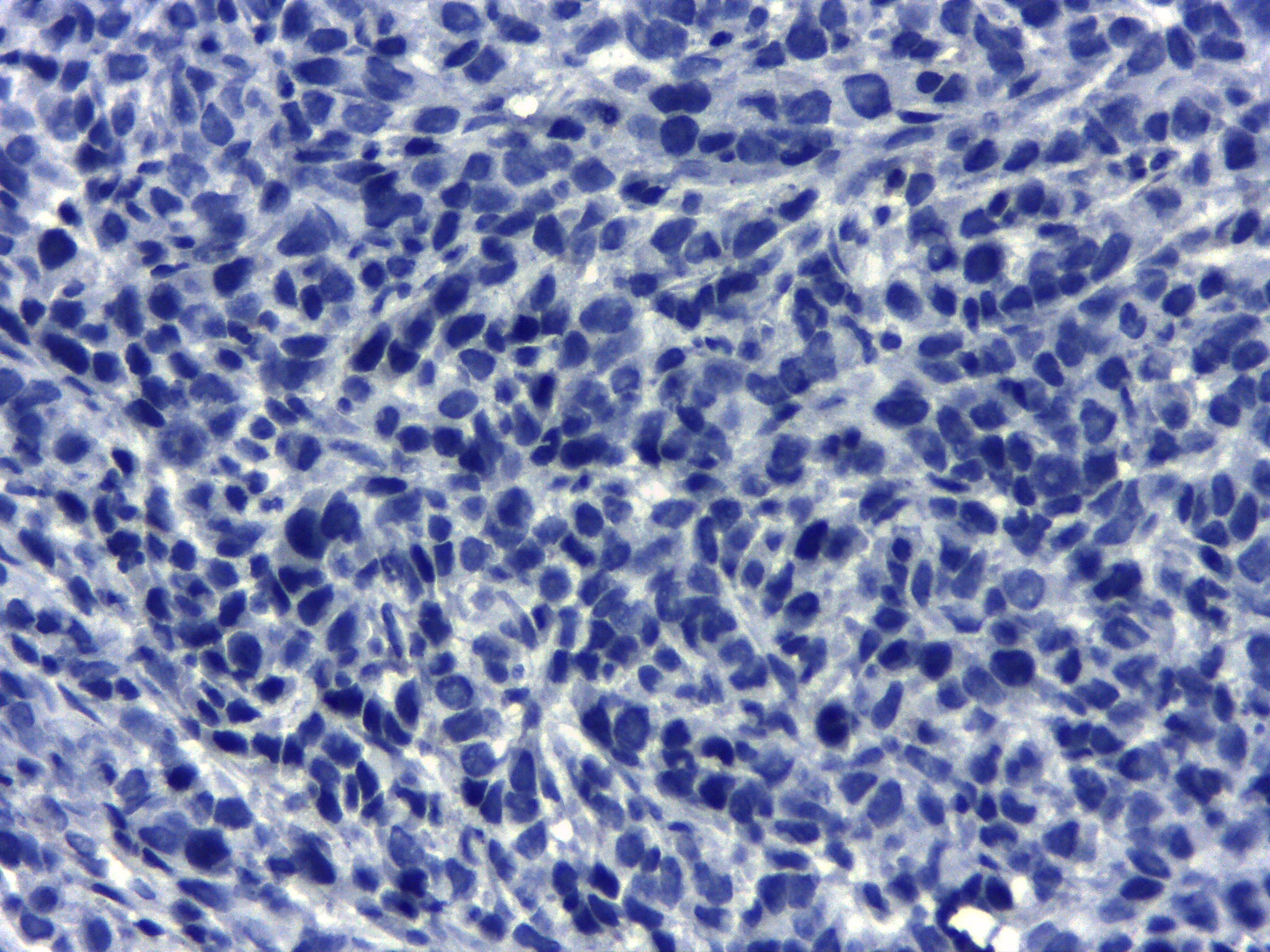

Supplement: S7 Fig — (ZIP) [file pone.0188960.s020.zip › HIF-1a IHC image BAC/HIF-1a bac6-3.jpg]

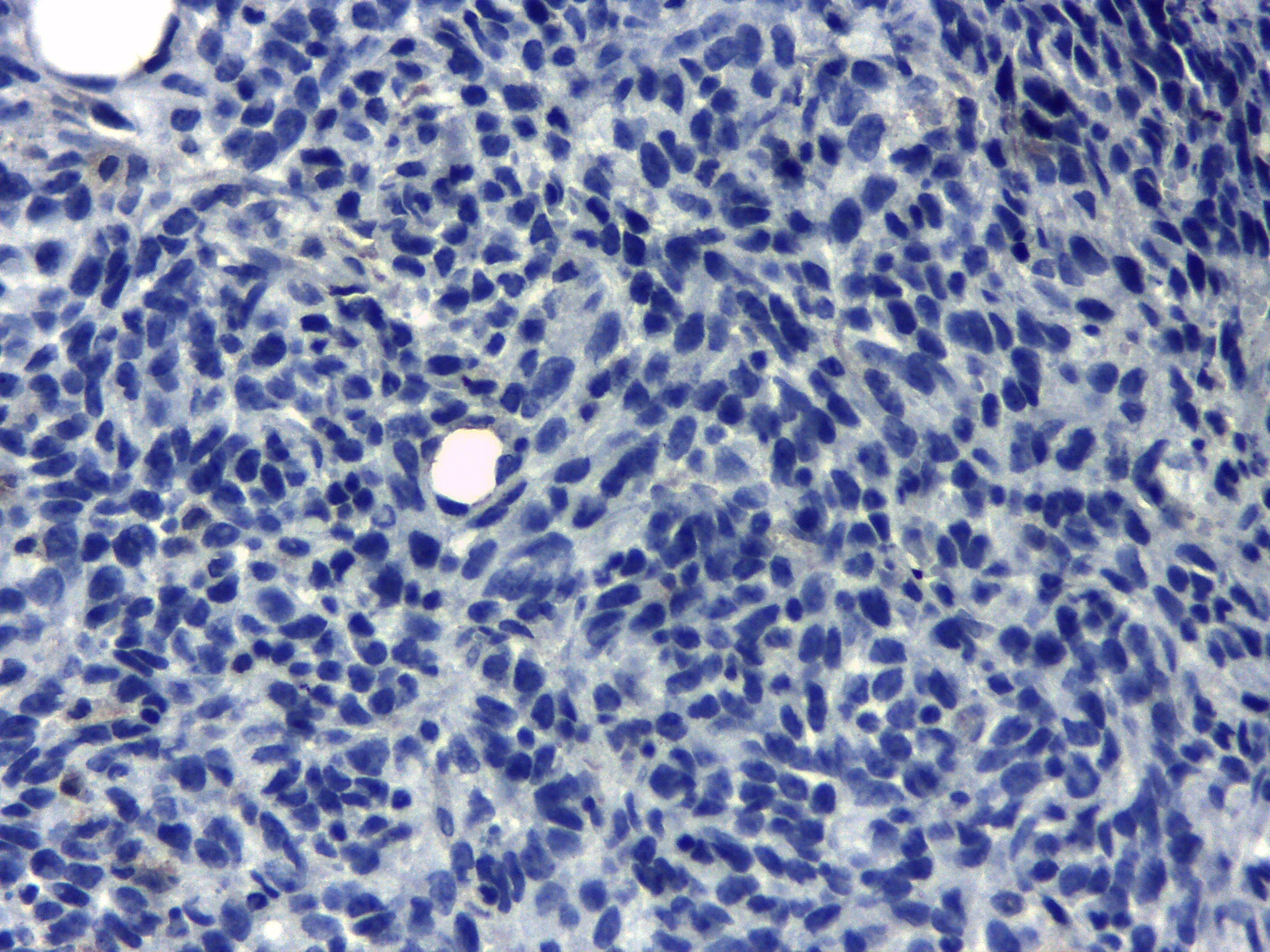

Supplement: S7 Fig — (ZIP) [file pone.0188960.s020.zip › HIF-1a IHC image BAC/HIF-1a bac6-4.jpg]

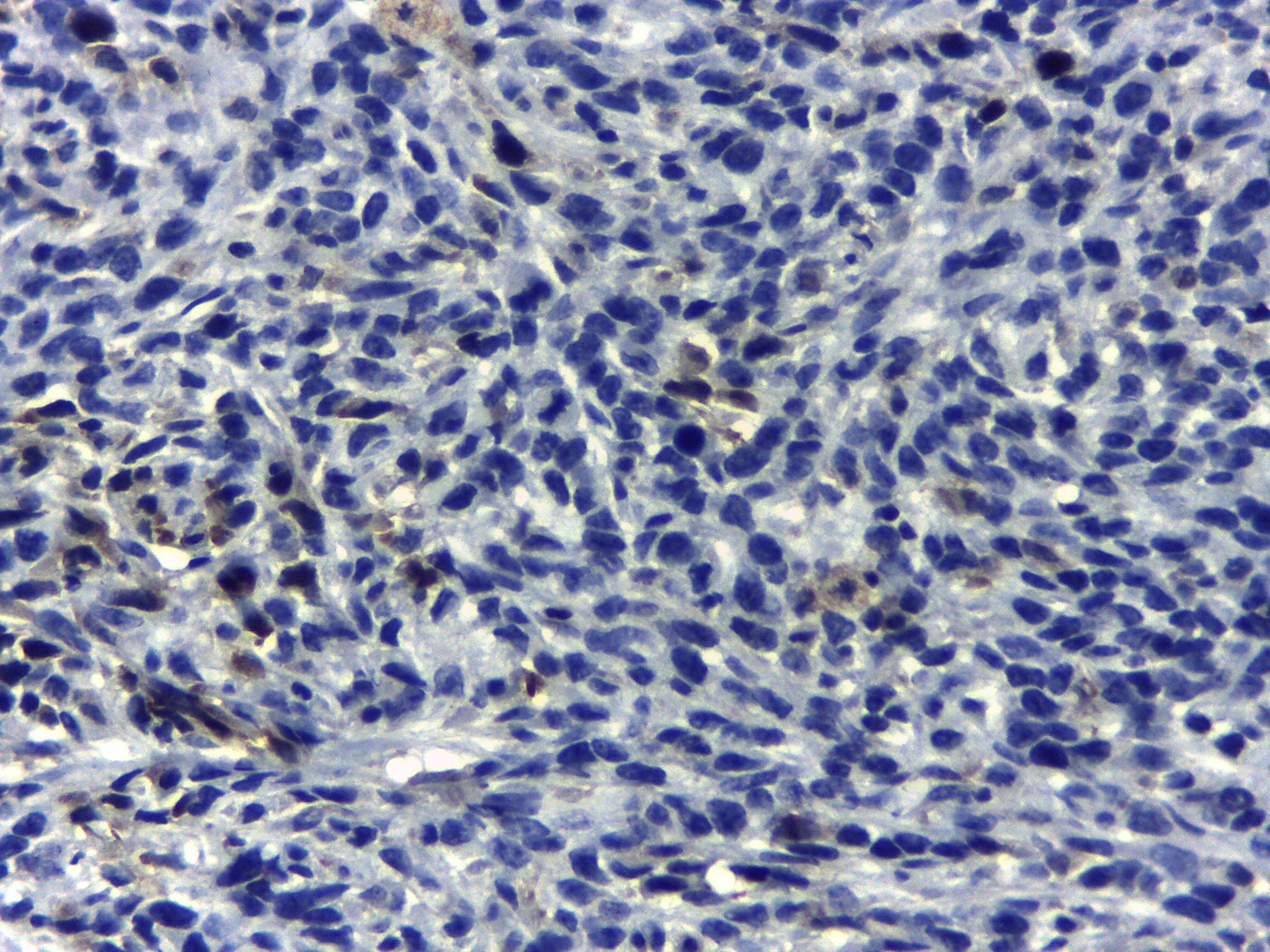

Supplement: S7 Fig — (ZIP) [file pone.0188960.s020.zip › HIF-1a IHC image BAC/HIF-1a bac6-5.jpg]

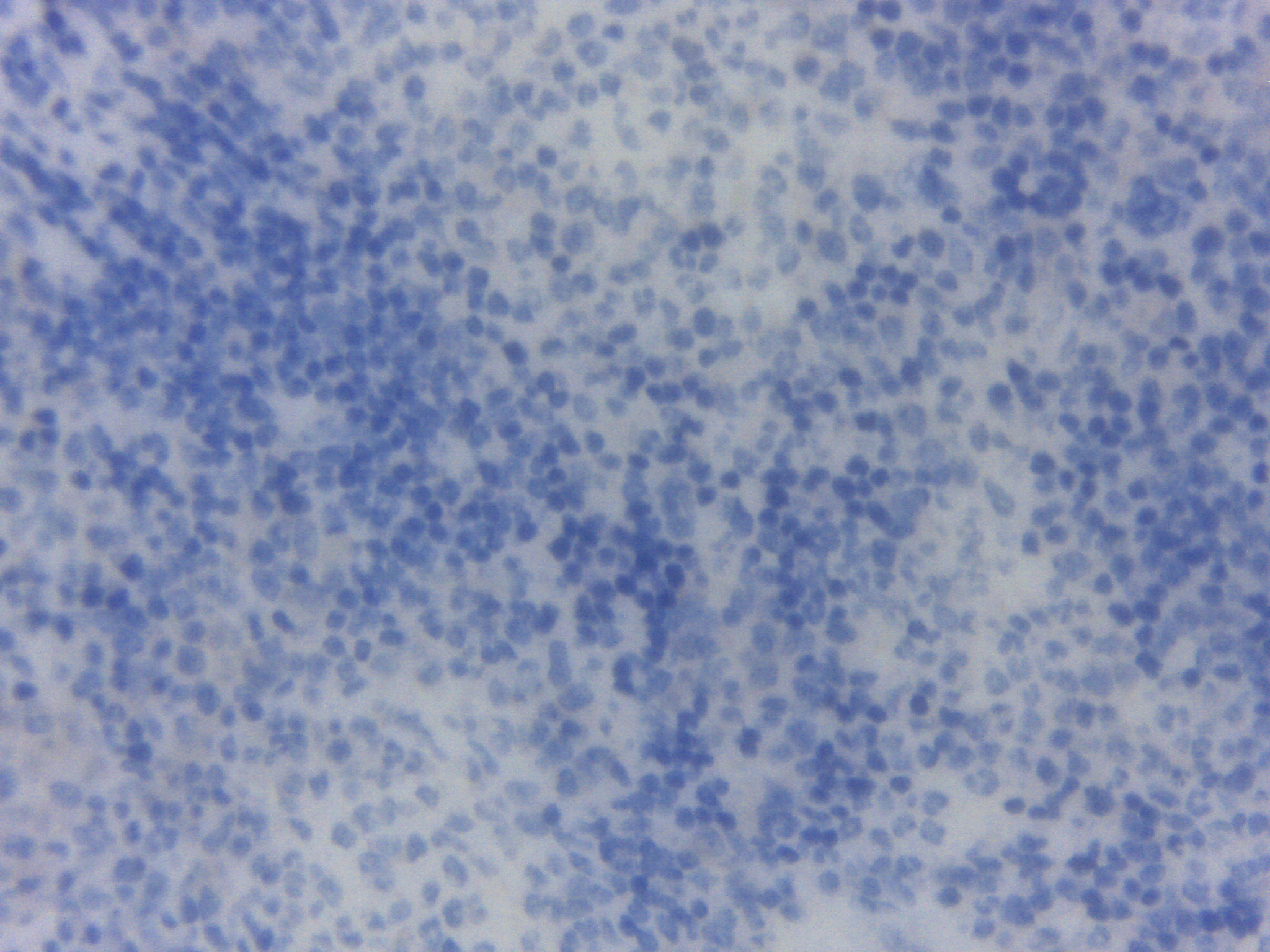

Supplement: S8 Fig — (ZIP) [file pone.0188960.s021.zip › CD11b IHC image 24 hours/96h-1-1.jpg]

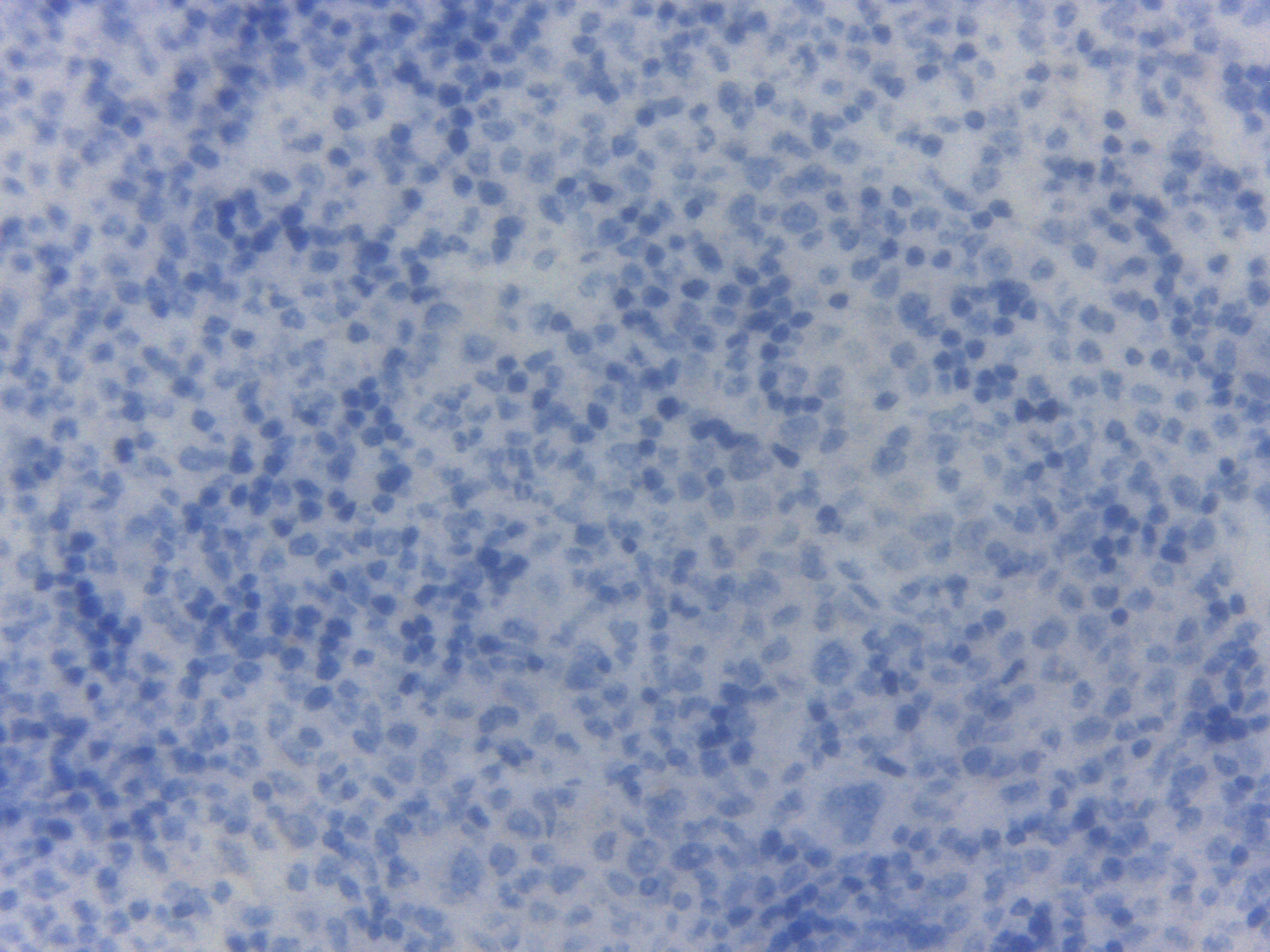

Supplement: S8 Fig — (ZIP) [file pone.0188960.s021.zip › CD11b IHC image 24 hours/96h-1-2.jpg]

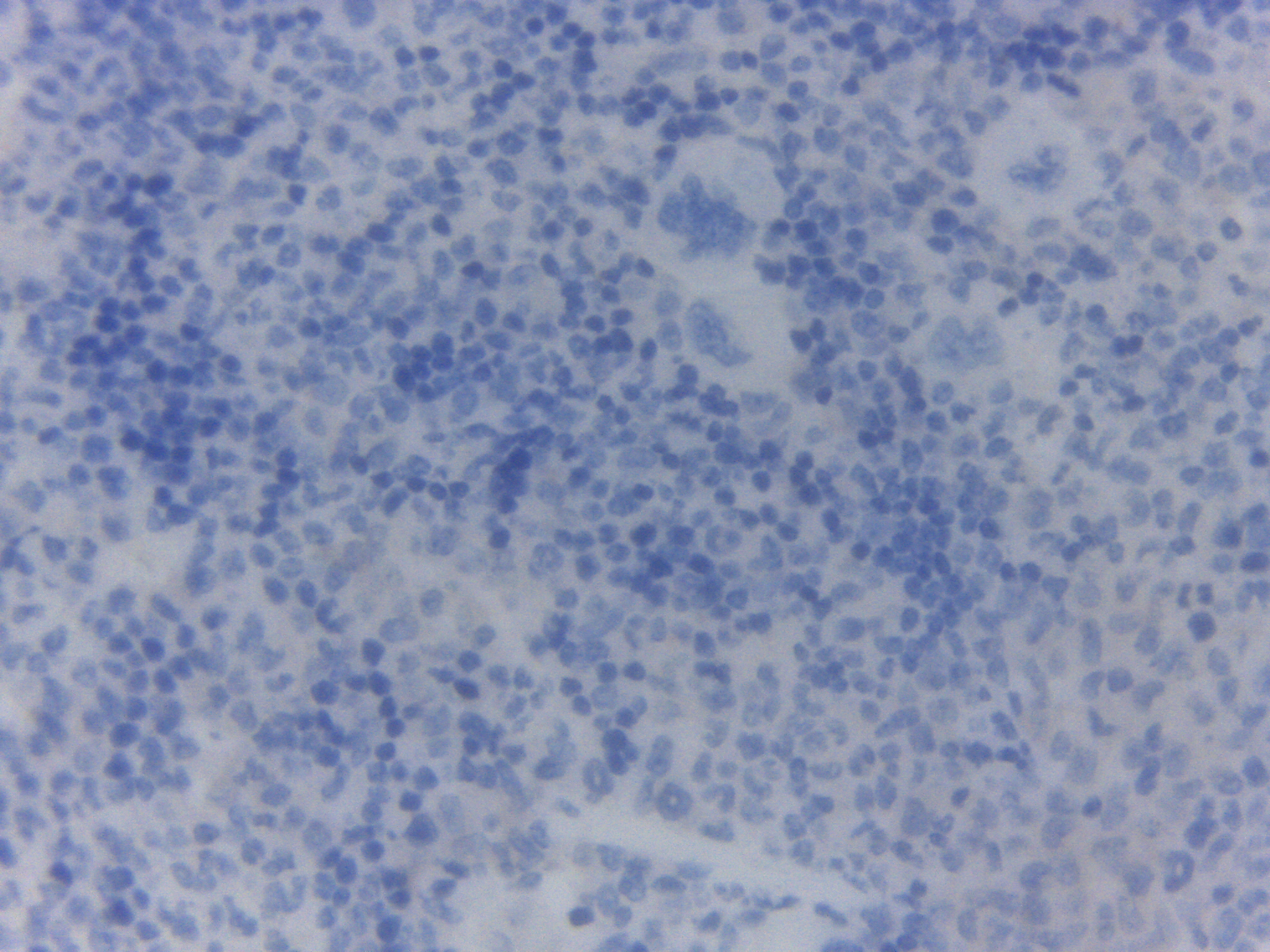

Supplement: S8 Fig — (ZIP) [file pone.0188960.s021.zip › CD11b IHC image 24 hours/96h-1-3.jpg]

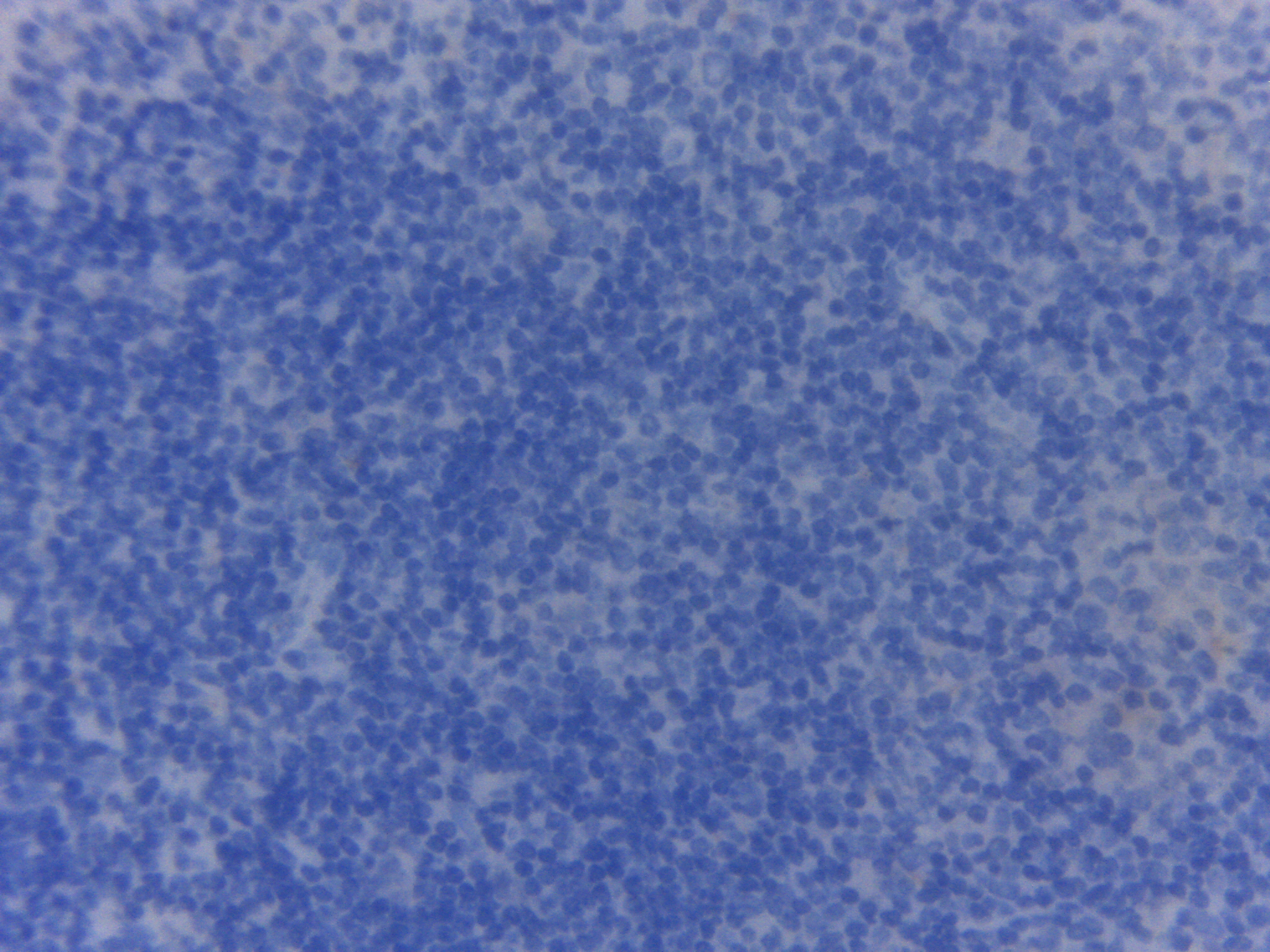

Supplement: S8 Fig — (ZIP) [file pone.0188960.s021.zip › CD11b IHC image 24 hours/96h-1-4.jpg]

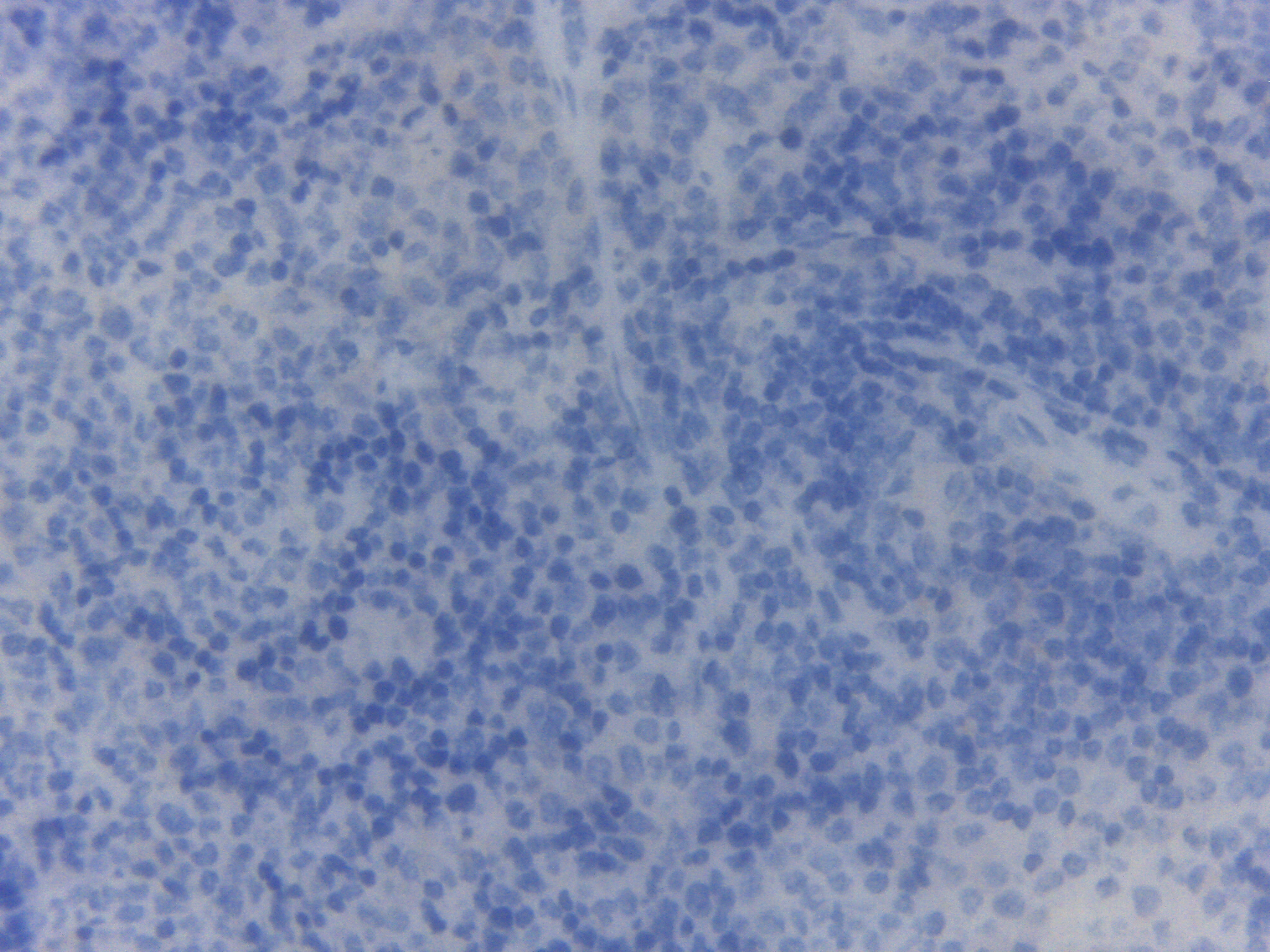

Supplement: S8 Fig — (ZIP) [file pone.0188960.s021.zip › CD11b IHC image 24 hours/96h-1-5.jpg]

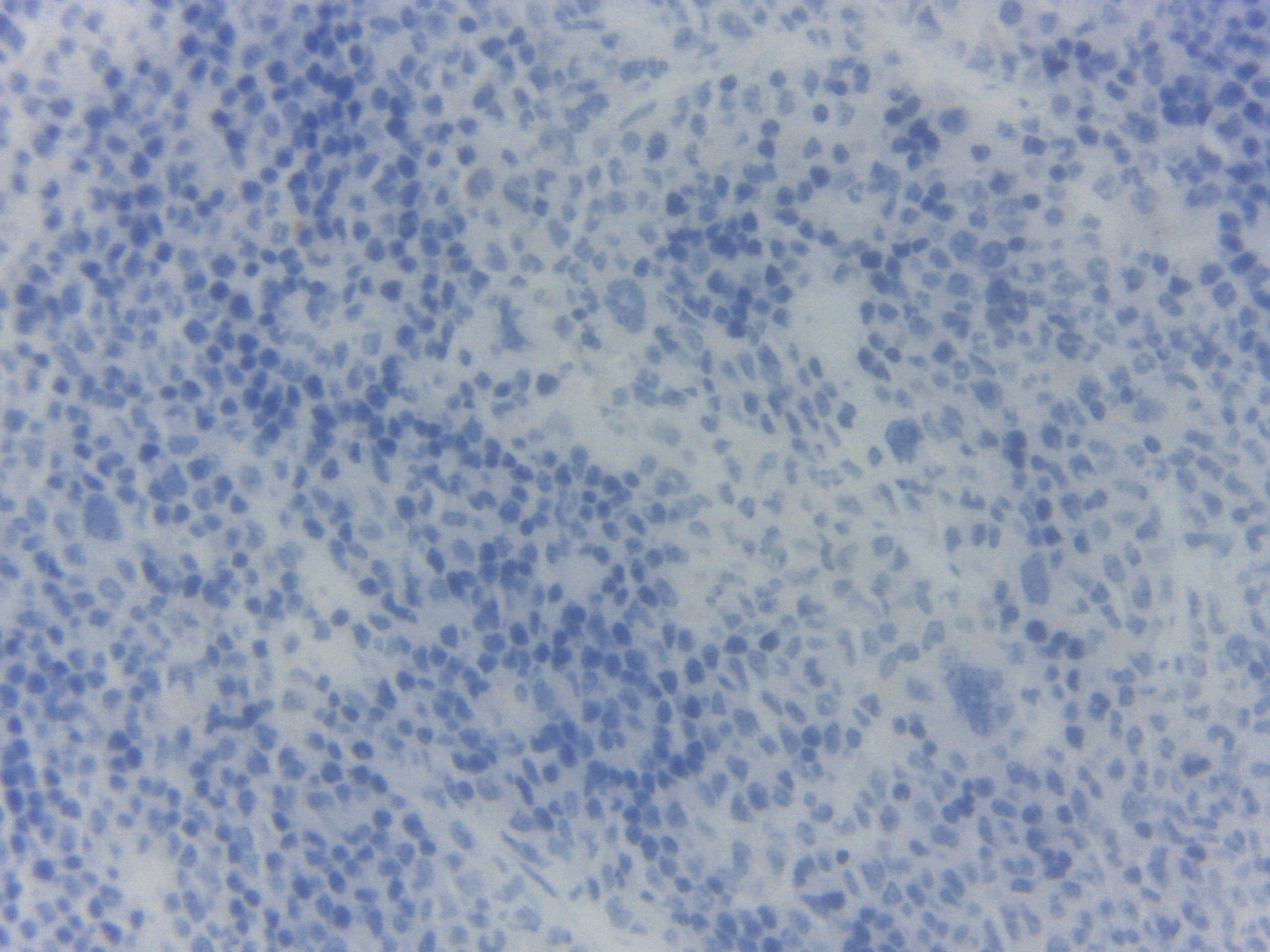

Supplement: S8 Fig — (ZIP) [file pone.0188960.s021.zip › CD11b IHC image 24 hours/96h-2-1.jpg]

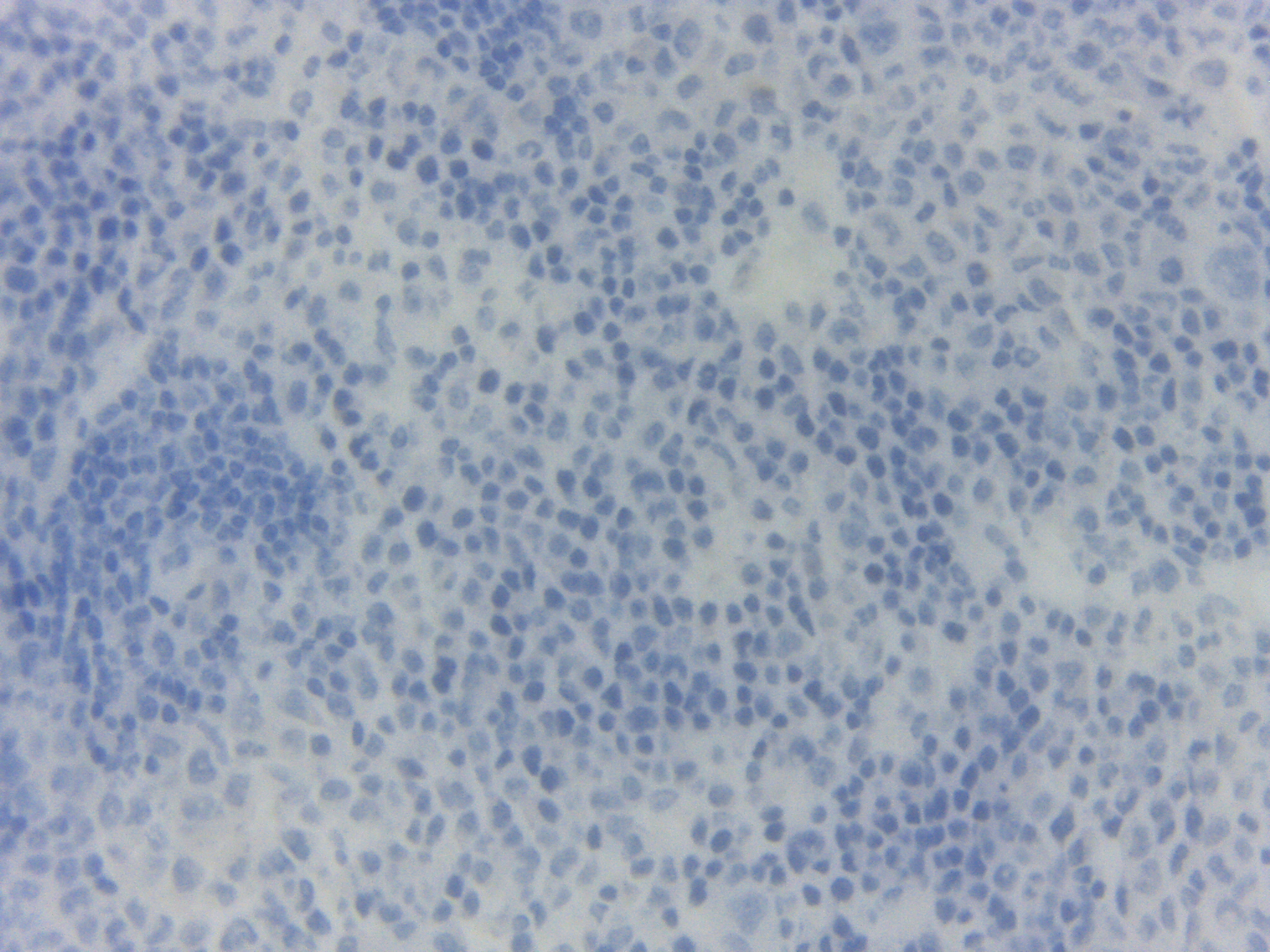

Supplement: S8 Fig — (ZIP) [file pone.0188960.s021.zip › CD11b IHC image 24 hours/96h-2-2.jpg]

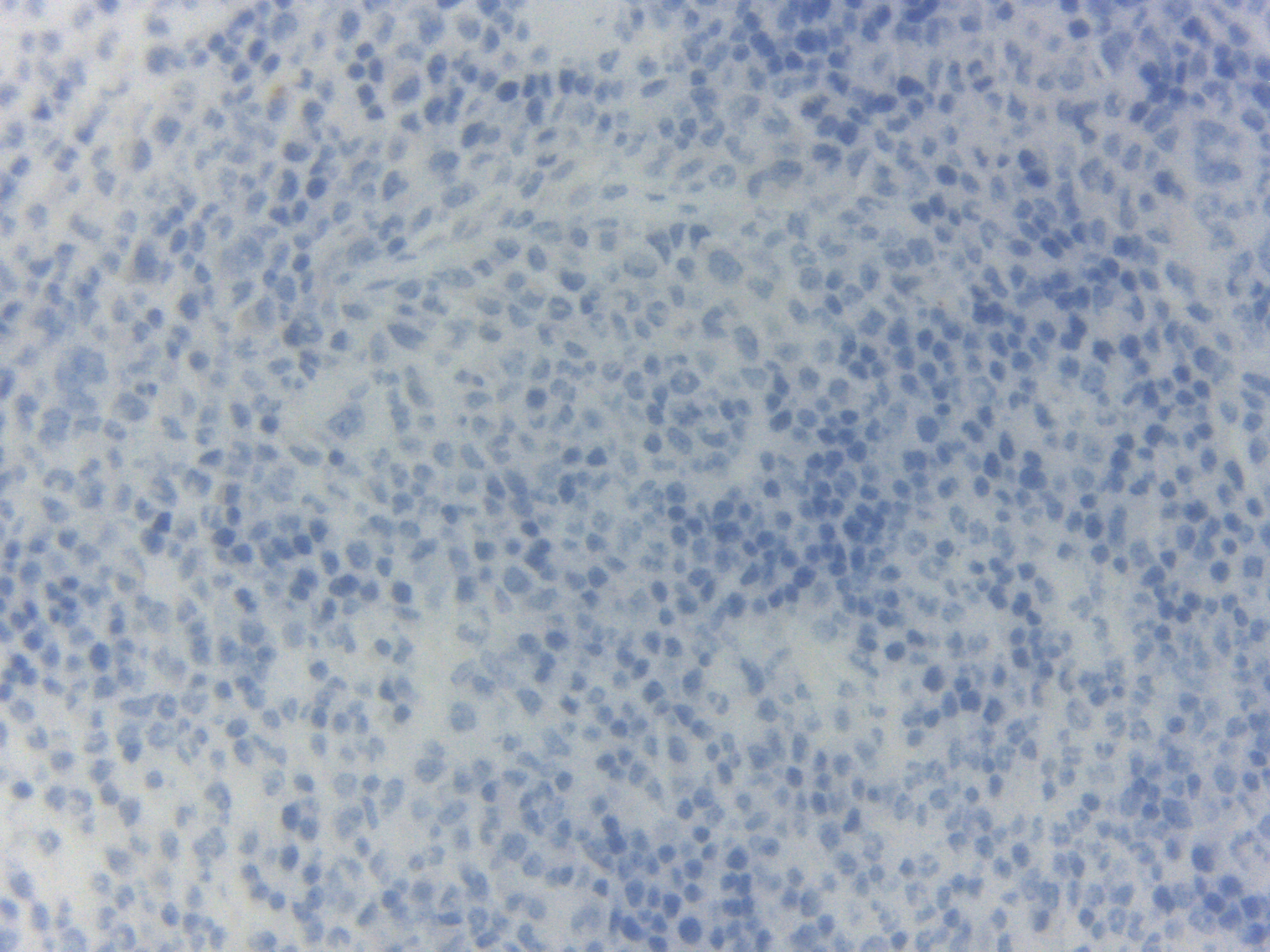

Supplement: S8 Fig — (ZIP) [file pone.0188960.s021.zip › CD11b IHC image 24 hours/96h-2-3.jpg]

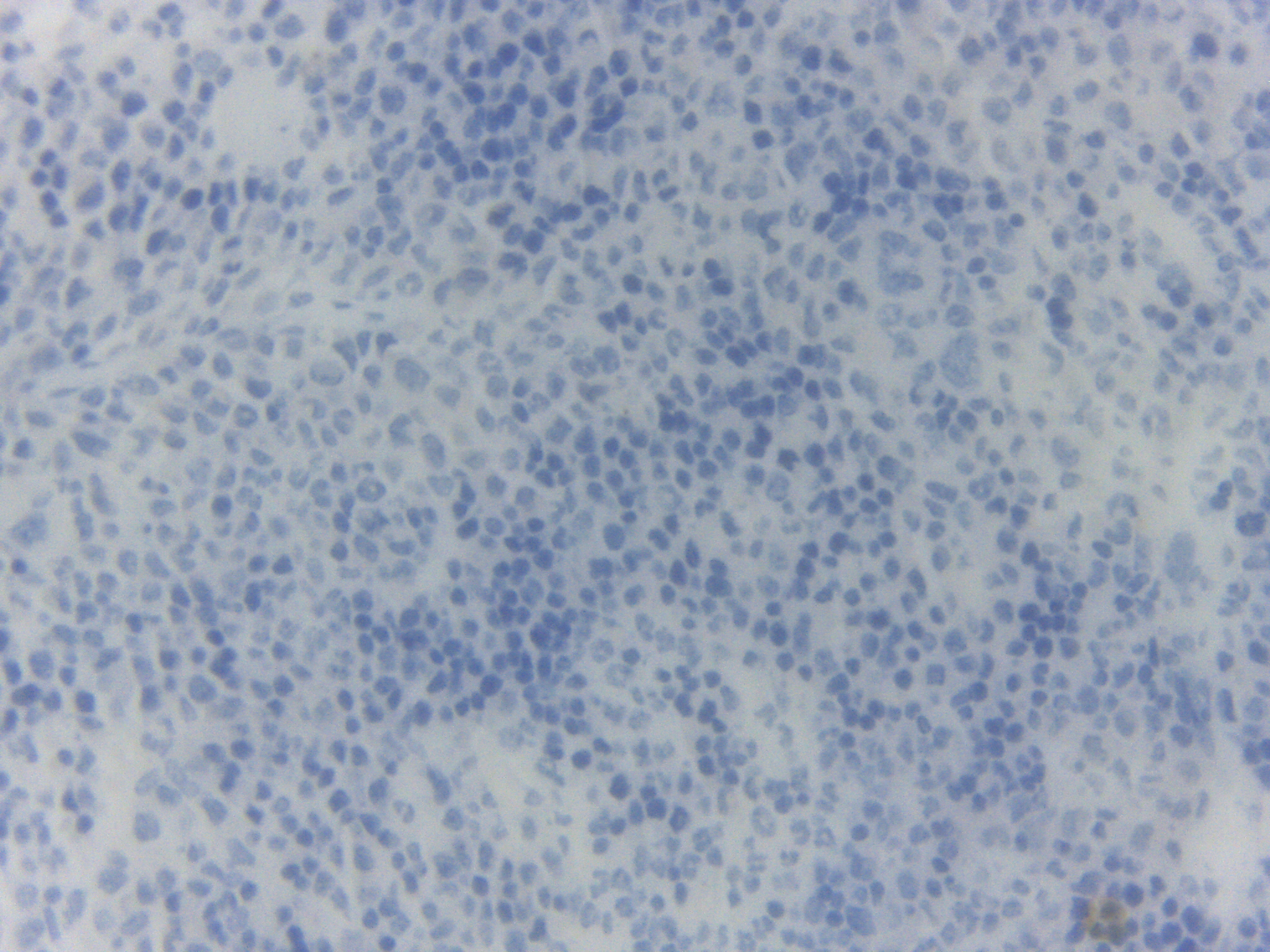

Supplement: S8 Fig — (ZIP) [file pone.0188960.s021.zip › CD11b IHC image 24 hours/96h-2-4.jpg]

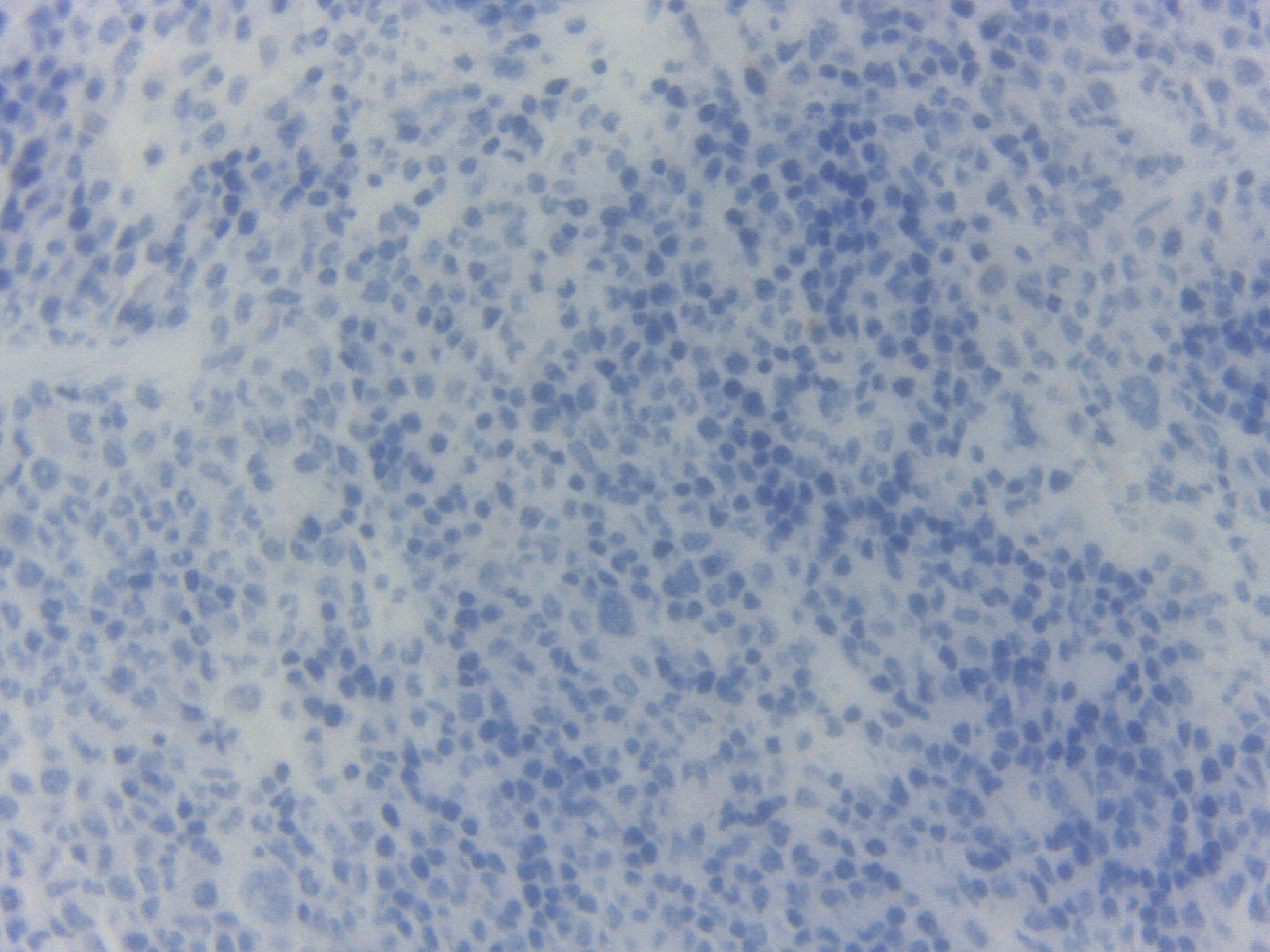

Supplement: S8 Fig — (ZIP) [file pone.0188960.s021.zip › CD11b IHC image 24 hours/96h-2-5.jpg]

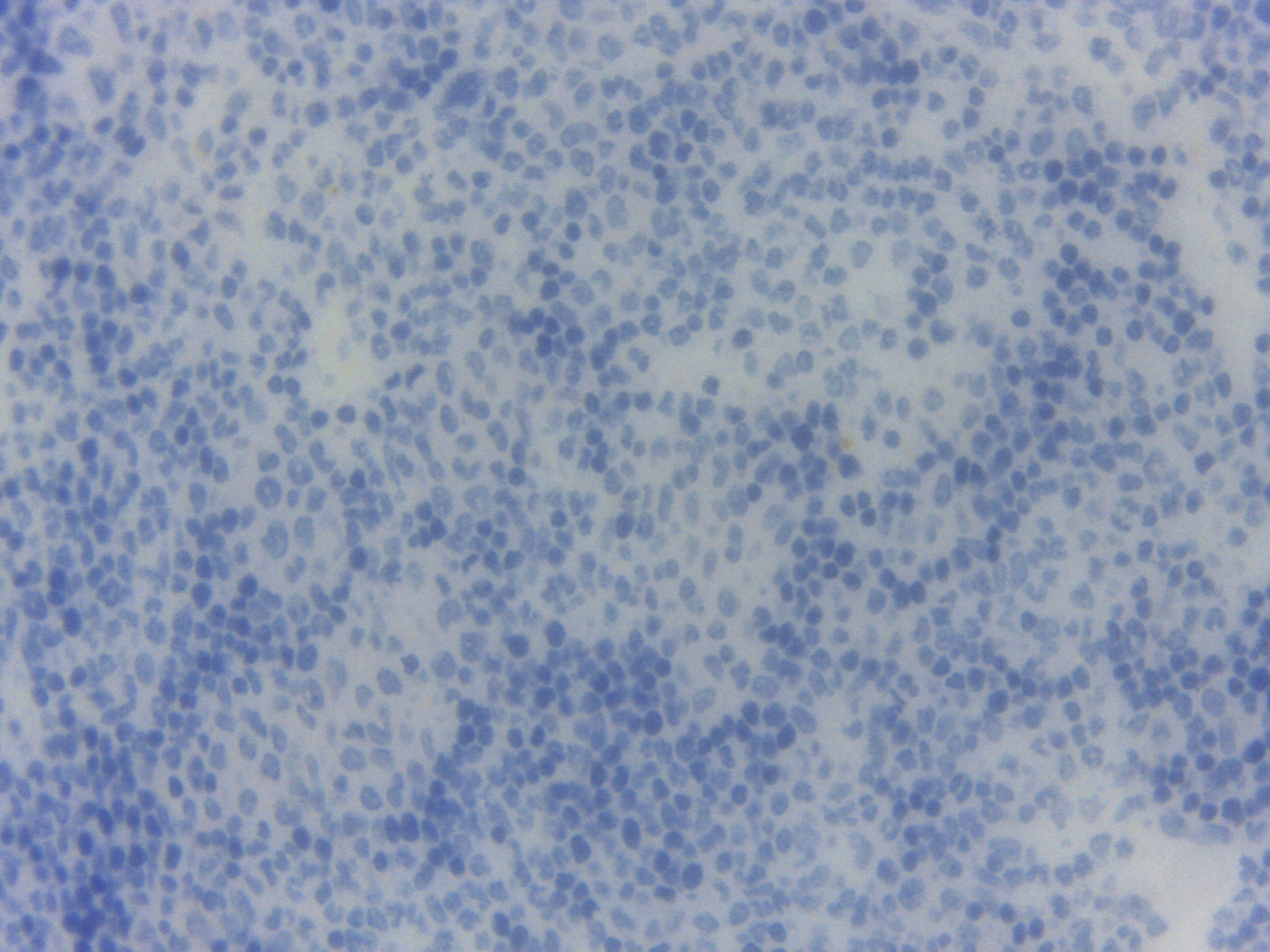

Supplement: S8 Fig — (ZIP) [file pone.0188960.s021.zip › CD11b IHC image 24 hours/96h-3-1.jpg]

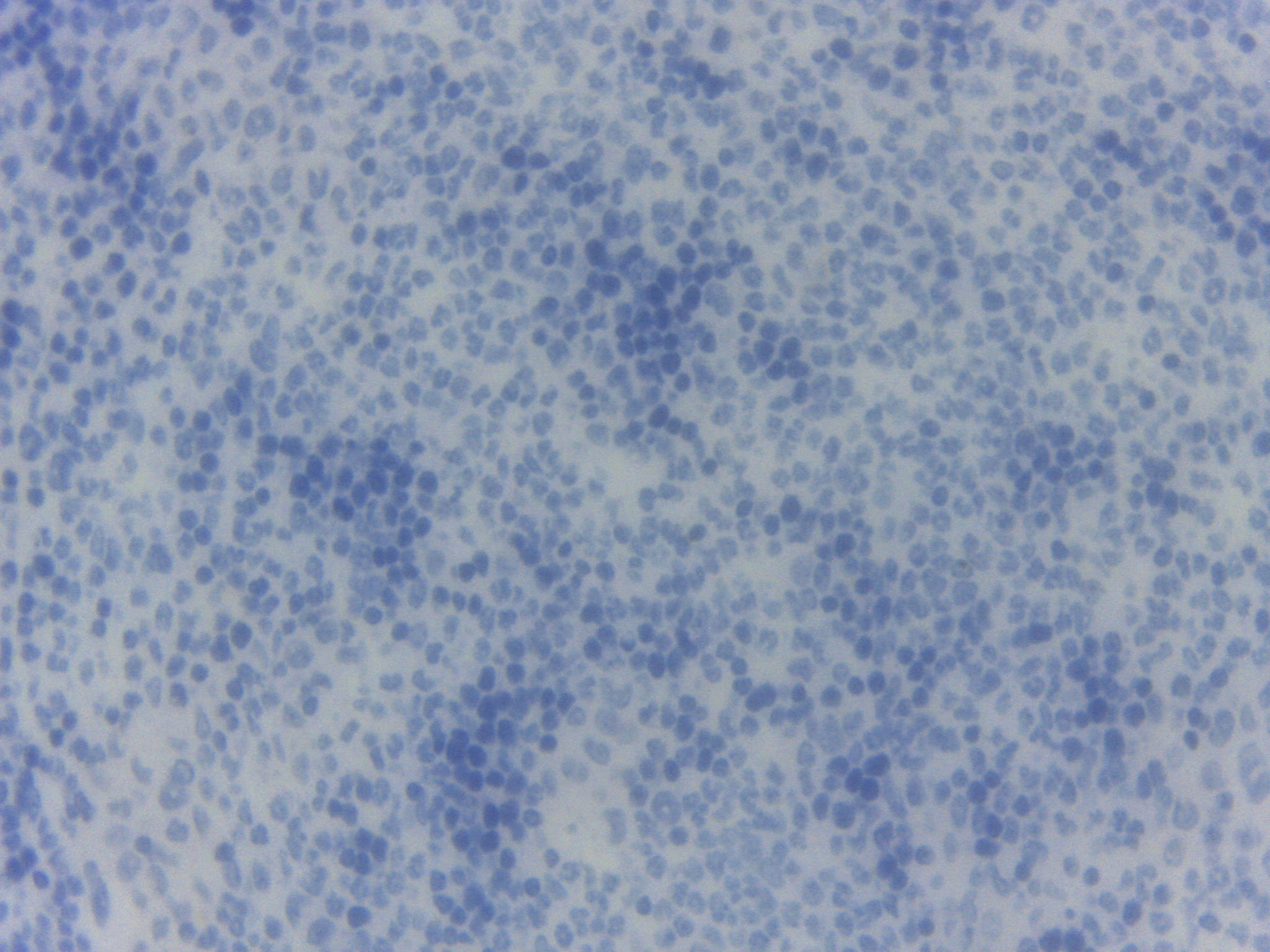

Supplement: S8 Fig — (ZIP) [file pone.0188960.s021.zip › CD11b IHC image 24 hours/96h-3-2.jpg]

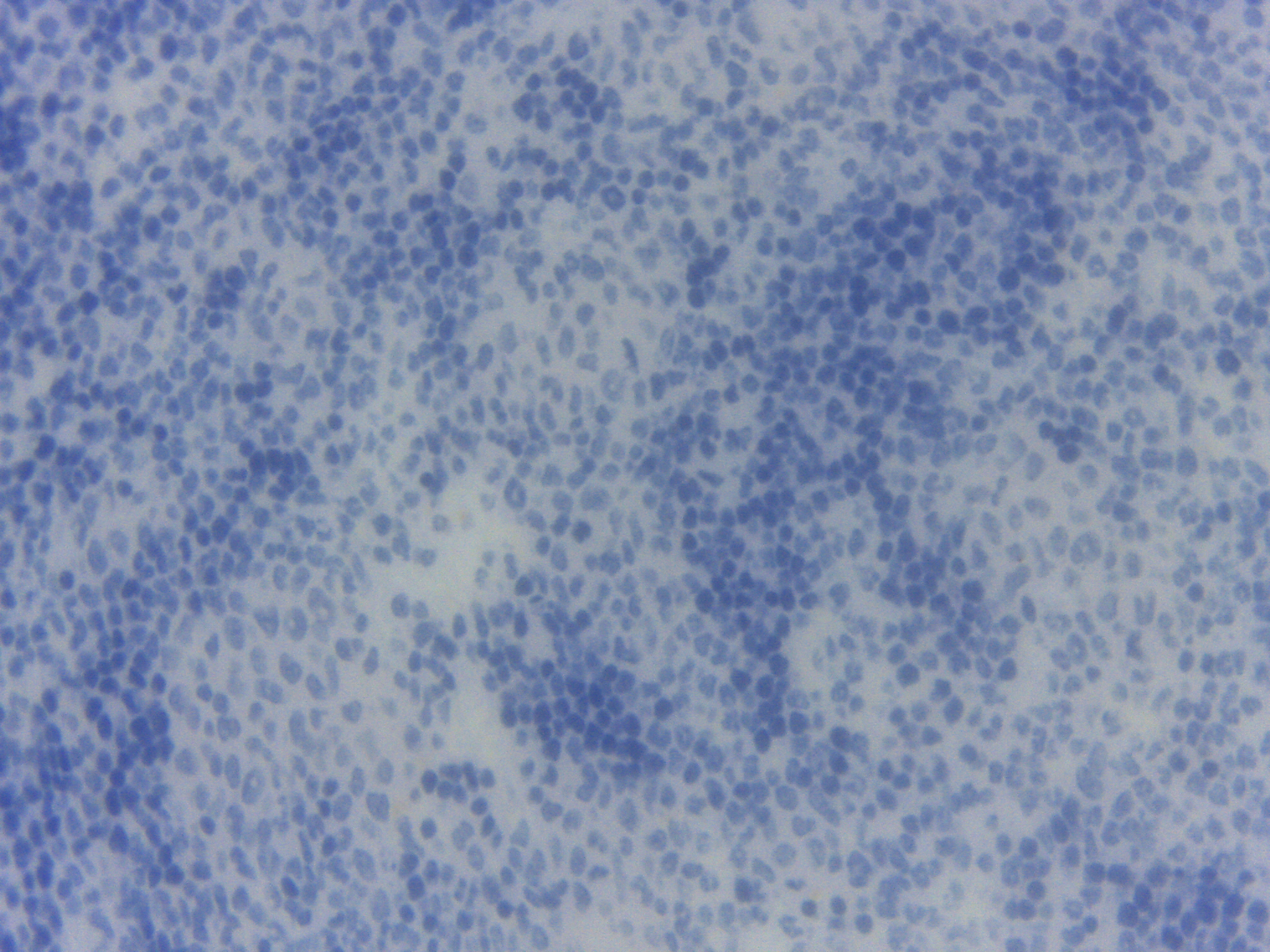

Supplement: S8 Fig — (ZIP) [file pone.0188960.s021.zip › CD11b IHC image 24 hours/96h-3-3.jpg]

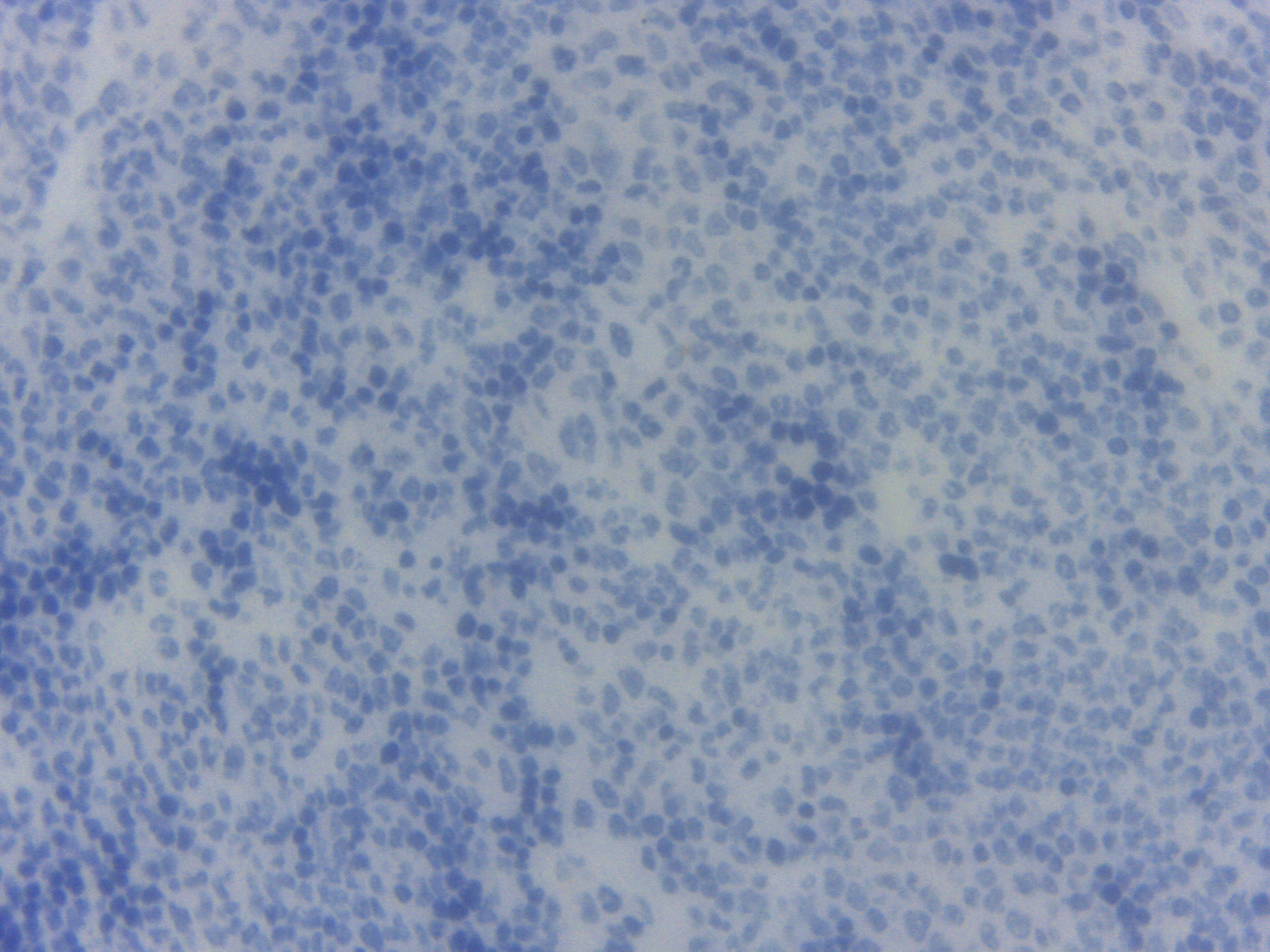

Supplement: S8 Fig — (ZIP) [file pone.0188960.s021.zip › CD11b IHC image 24 hours/96h-3-4.jpg]

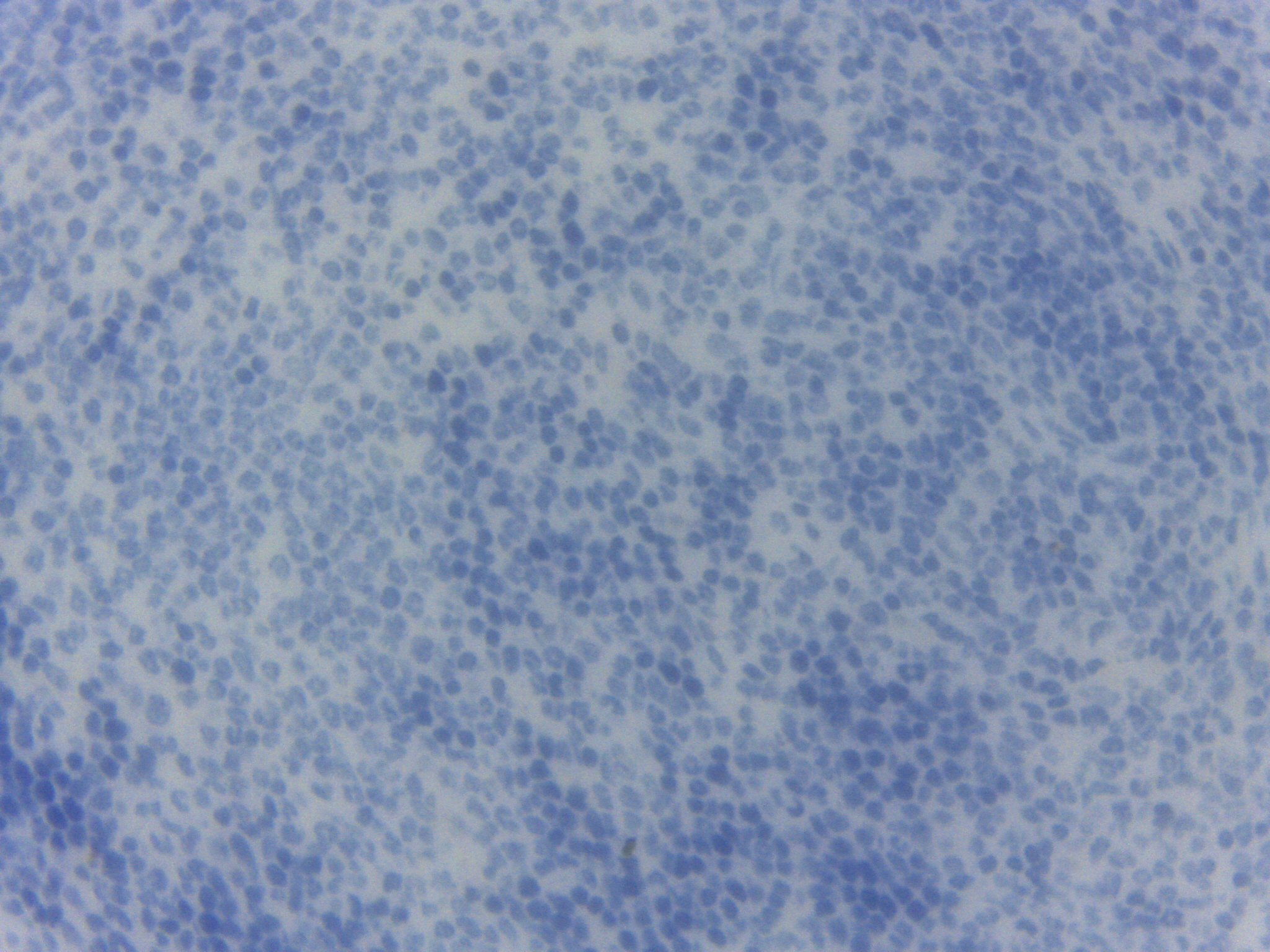

Supplement: S8 Fig — (ZIP) [file pone.0188960.s021.zip › CD11b IHC image 24 hours/96h-3-5.jpg]

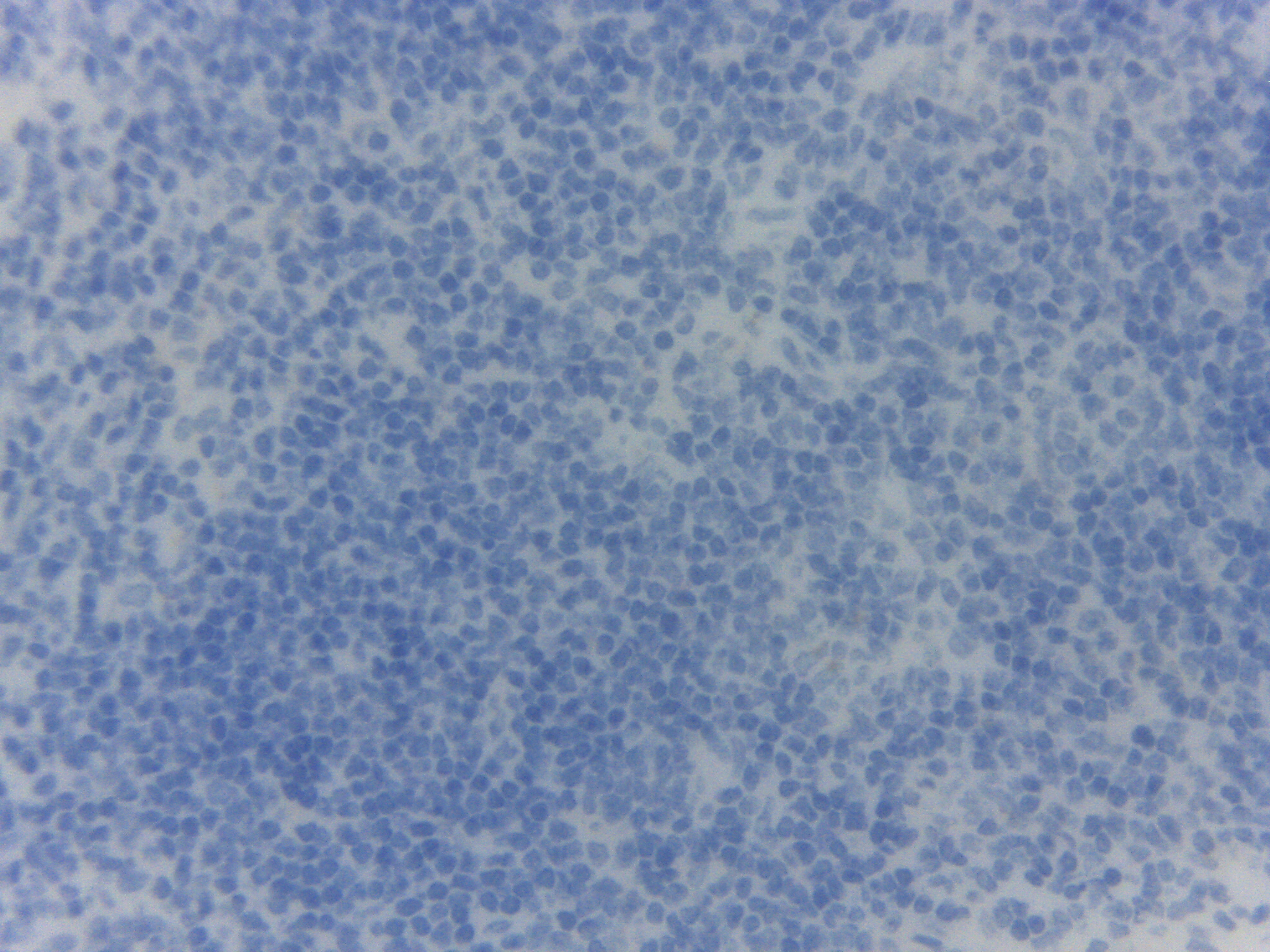

Supplement: S8 Fig — (ZIP) [file pone.0188960.s021.zip › CD11b IHC image 24 hours/96h-4-1.jpg]

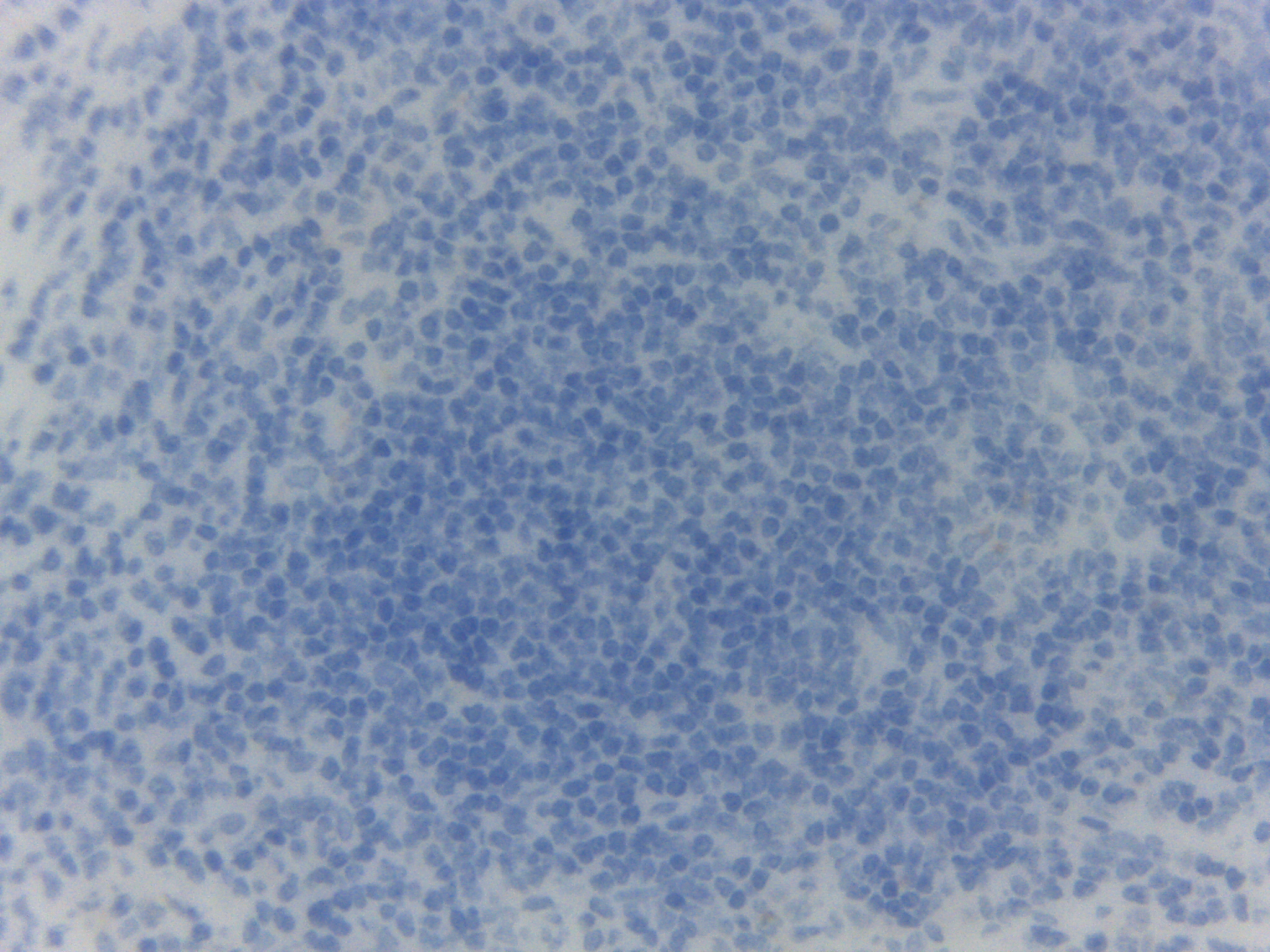

Supplement: S8 Fig — (ZIP) [file pone.0188960.s021.zip › CD11b IHC image 24 hours/96h-4-2.jpg]

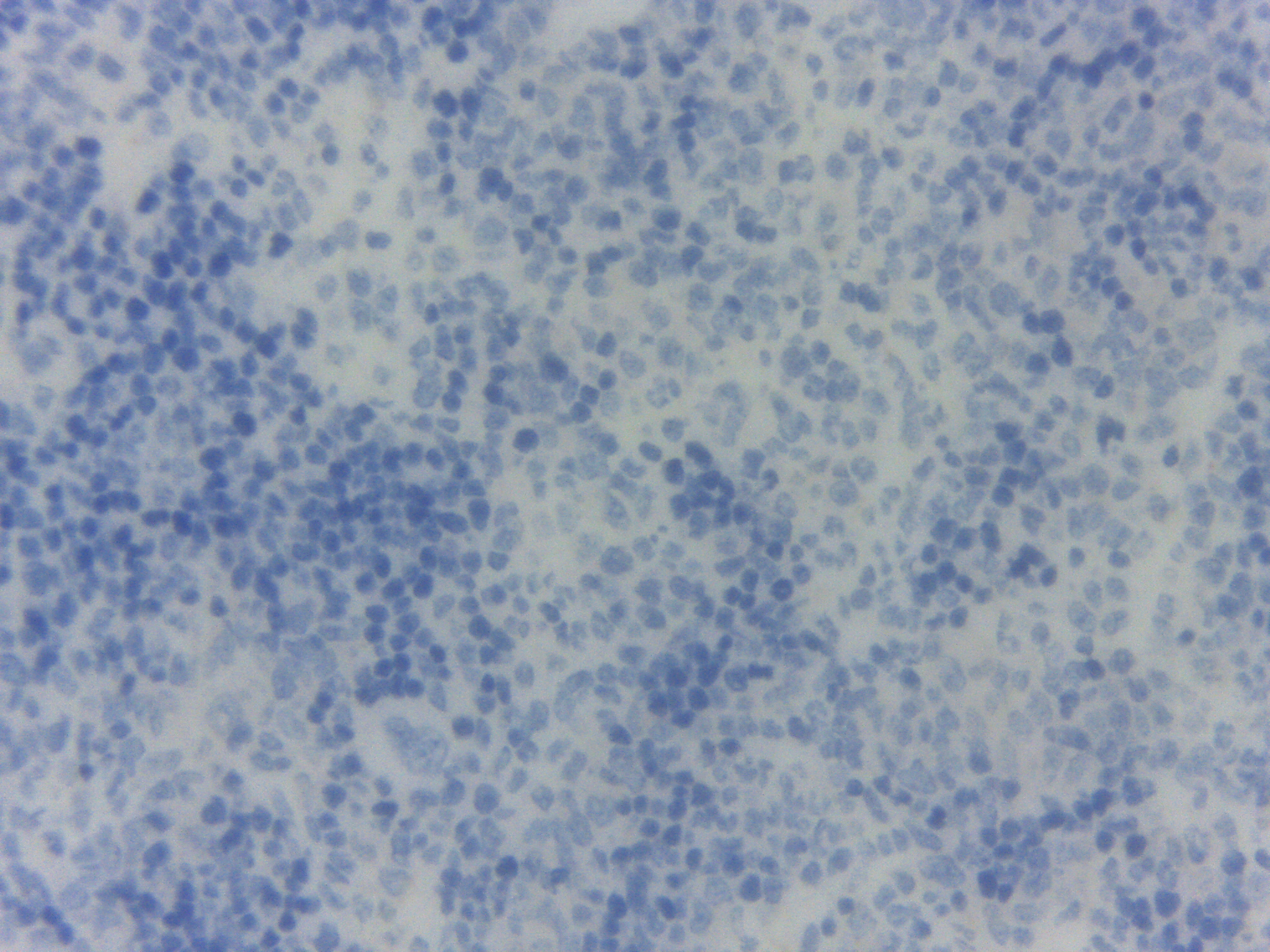

Supplement: S8 Fig — (ZIP) [file pone.0188960.s021.zip › CD11b IHC image 24 hours/96h-4-3.jpg]
