# Supplementary figures and images for: Proteus mirabilis inhibits cancer growth and pulmonary metastasis in a mouse breast cancer model (part 4 of 5)
Source: PLoS One. 2017 Dec 5;12(12):e0188960. doi: 10.1371/journal.pone.0188960 (PMC5716547; doi:10.1371/journal.pone.0188960)

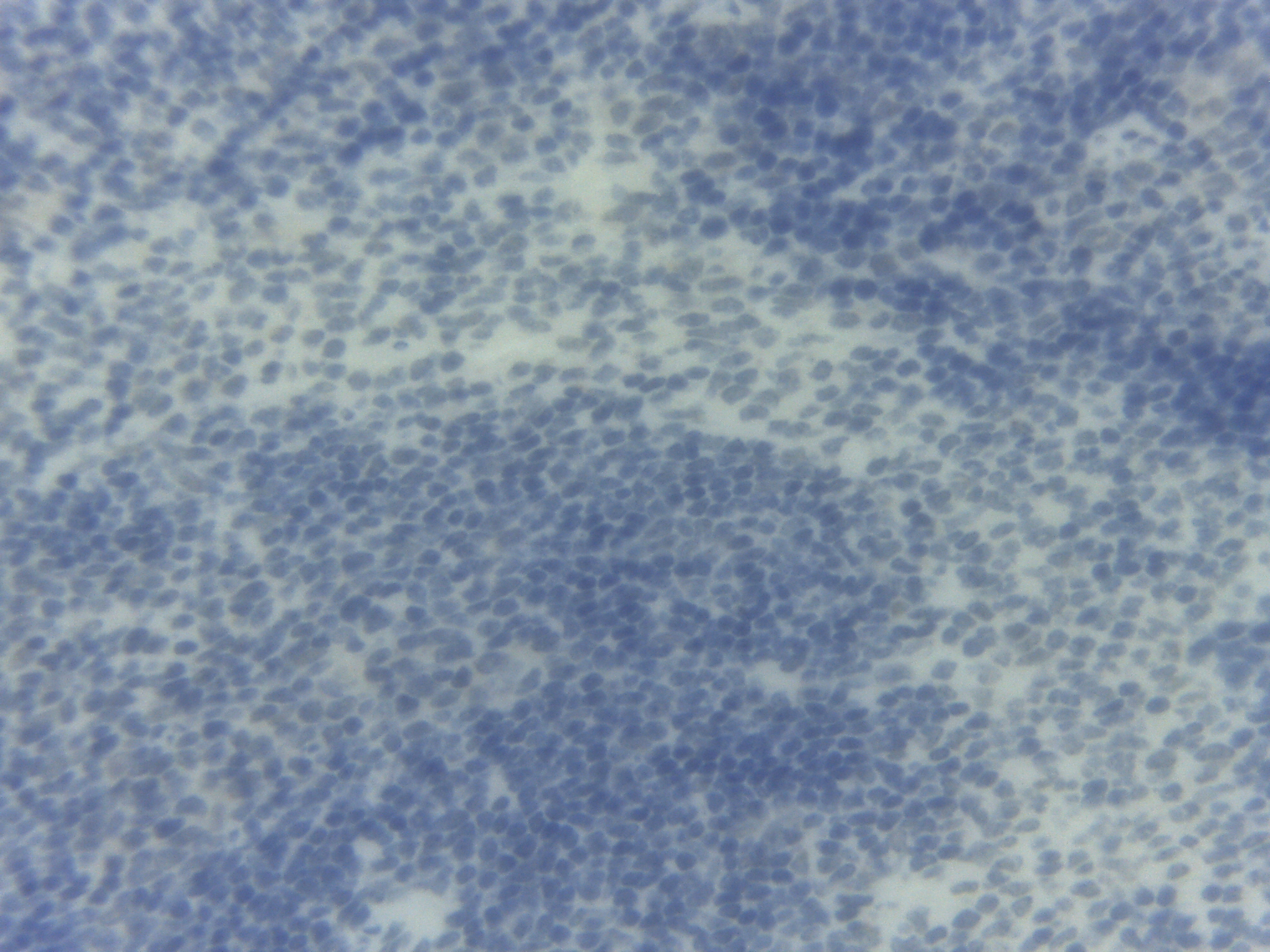

Supplement: S11 Fig — (ZIP) [file pone.0188960.s024.zip › CD11c IHC image CON/con-6-4.jpg]

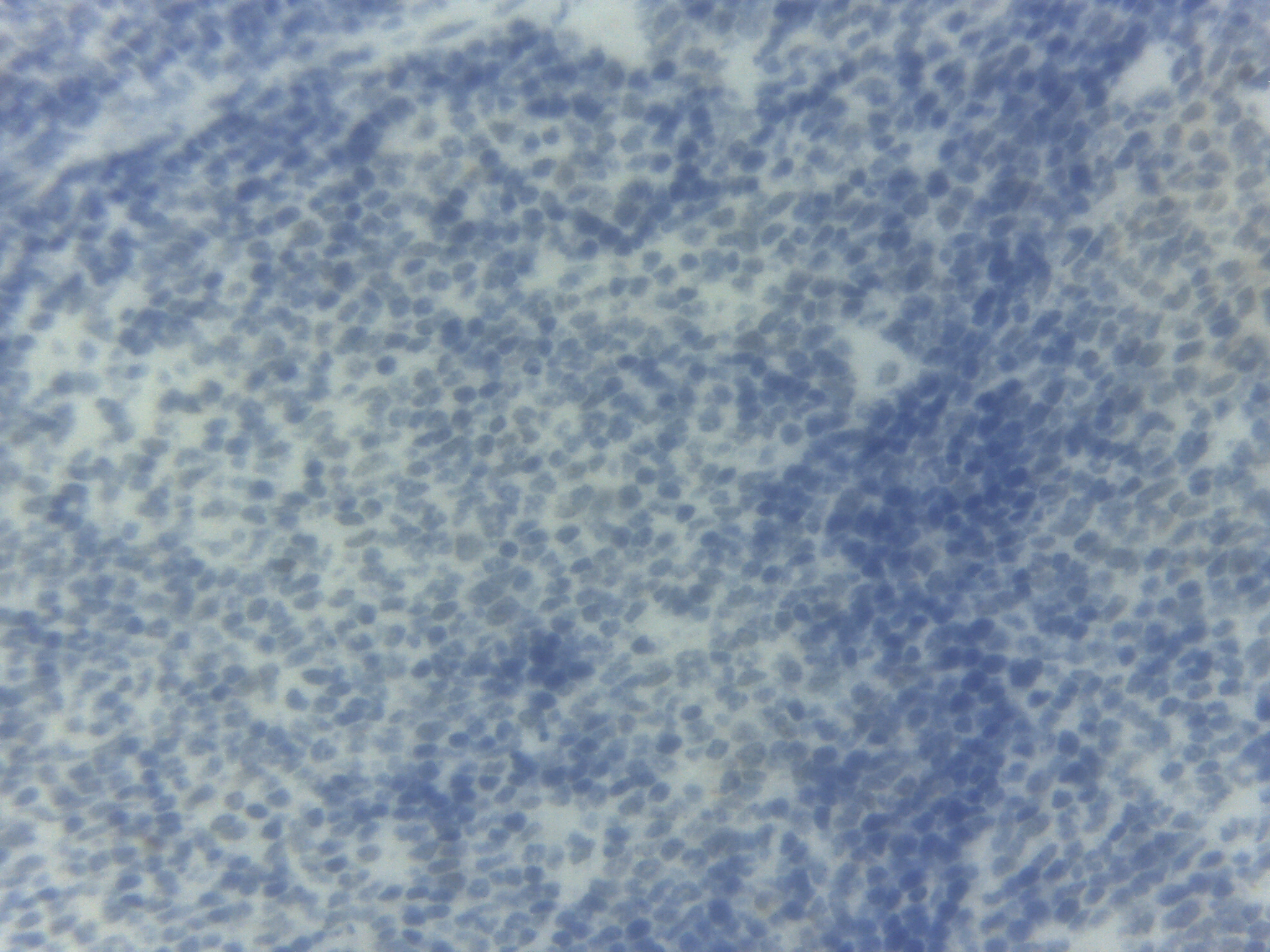

Supplement: S11 Fig — (ZIP) [file pone.0188960.s024.zip › CD11c IHC image CON/con-6-5.jpg]

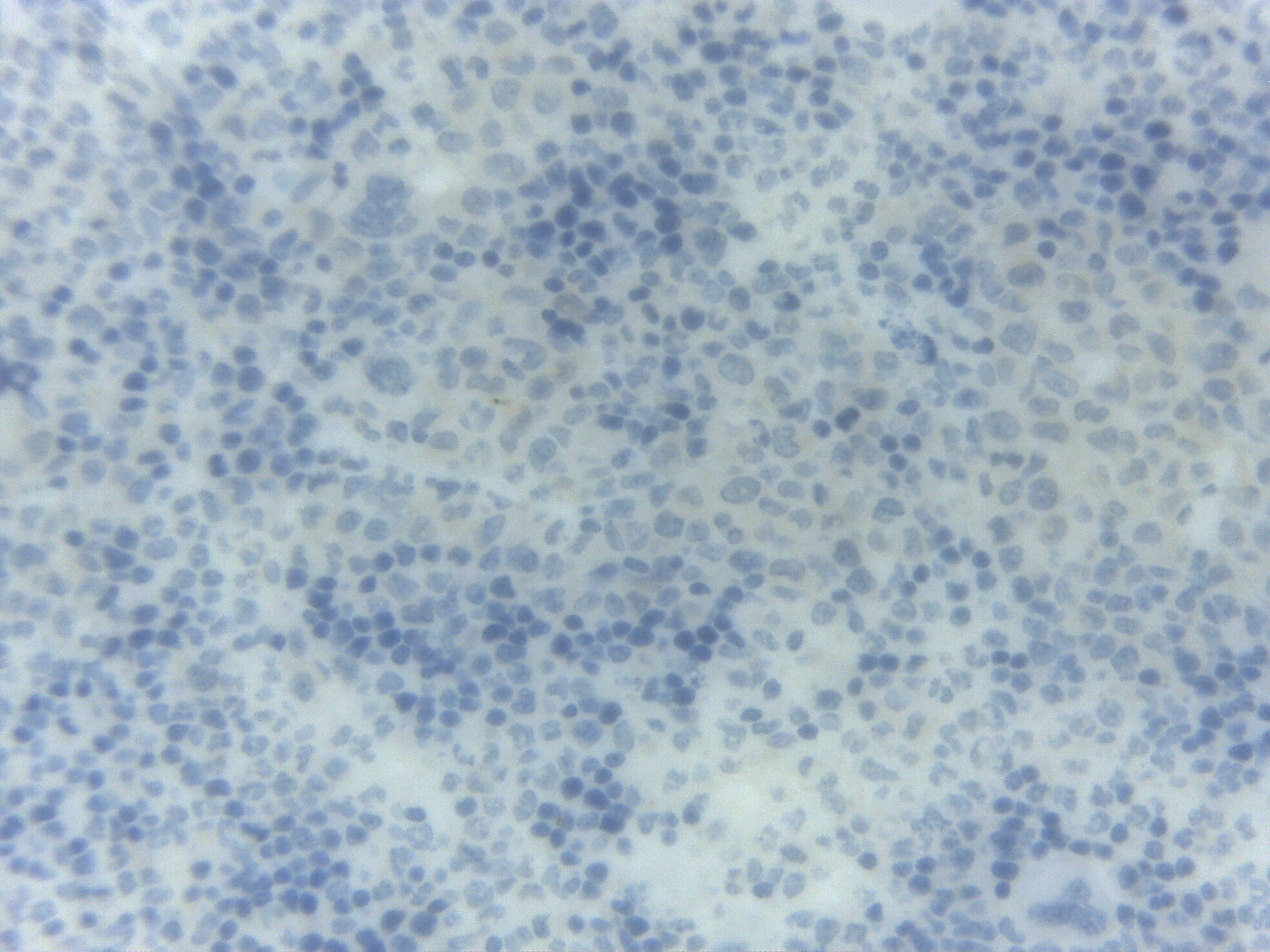

Supplement: S12 Fig — (ZIP) [file pone.0188960.s025.zip › Ly-6G IHC image24 hours/24h-1-1.jpg]

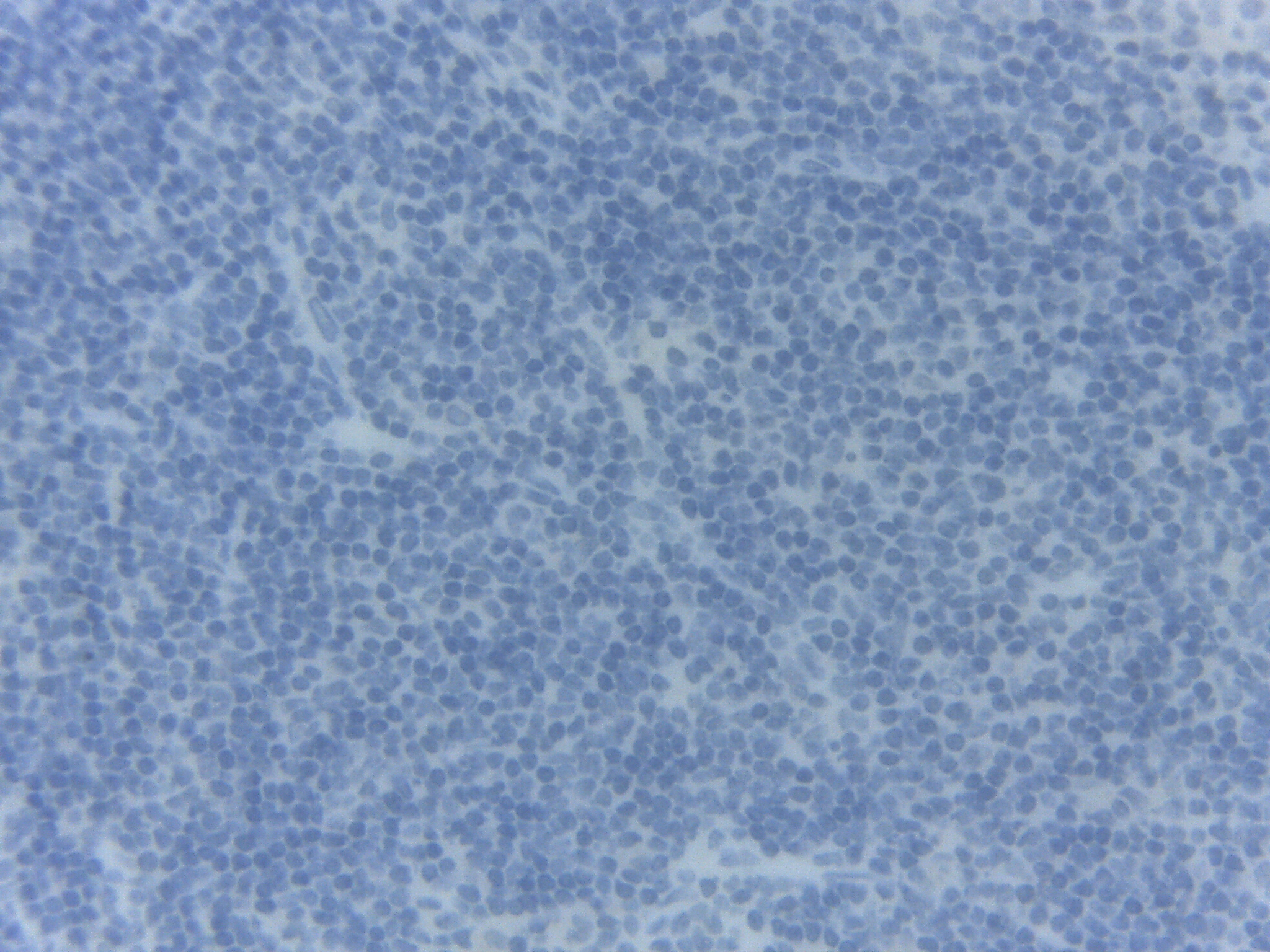

Supplement: S12 Fig — (ZIP) [file pone.0188960.s025.zip › Ly-6G IHC image24 hours/24h-1-2.jpg]

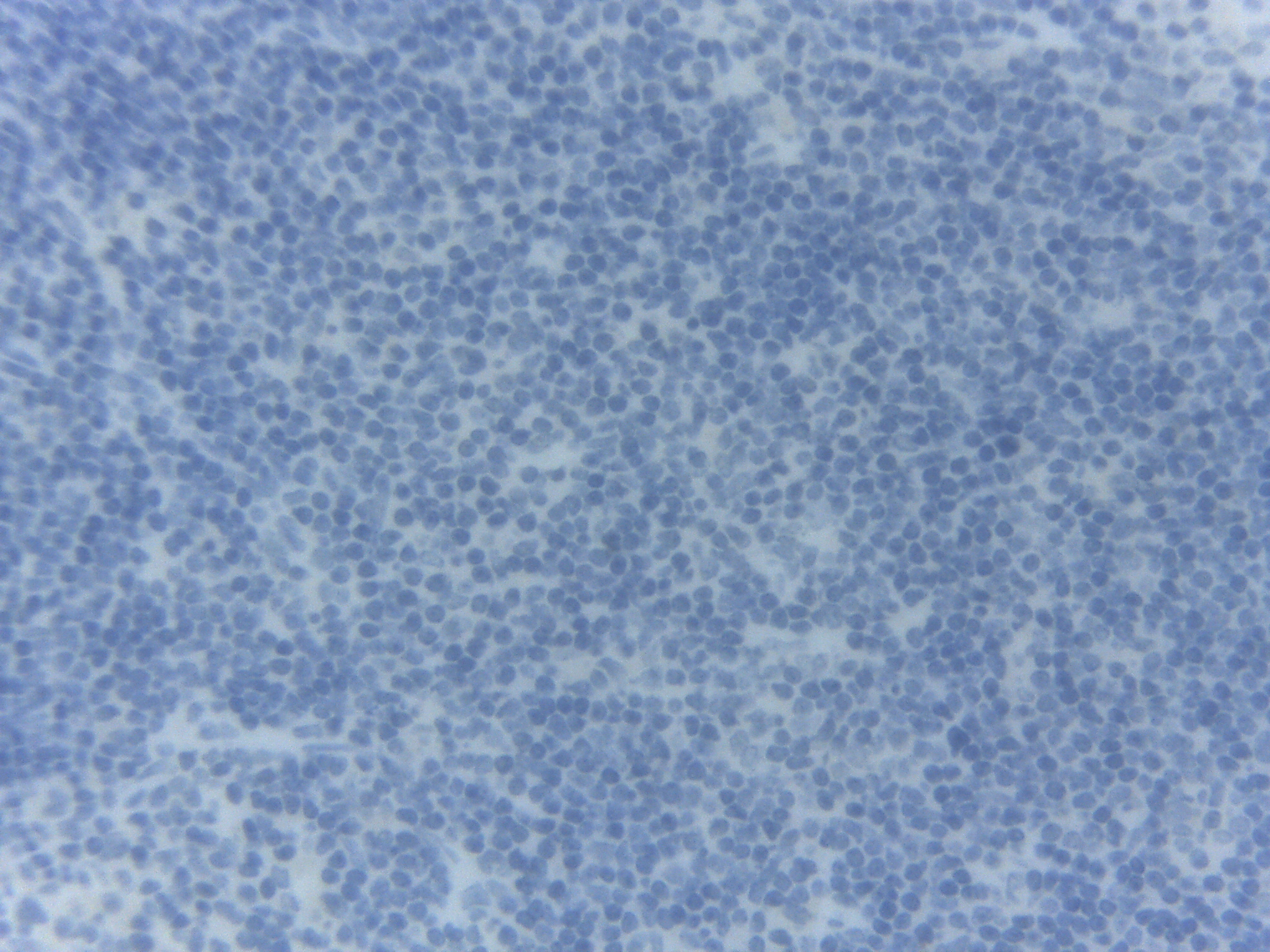

Supplement: S12 Fig — (ZIP) [file pone.0188960.s025.zip › Ly-6G IHC image24 hours/24h-1-3.jpg]

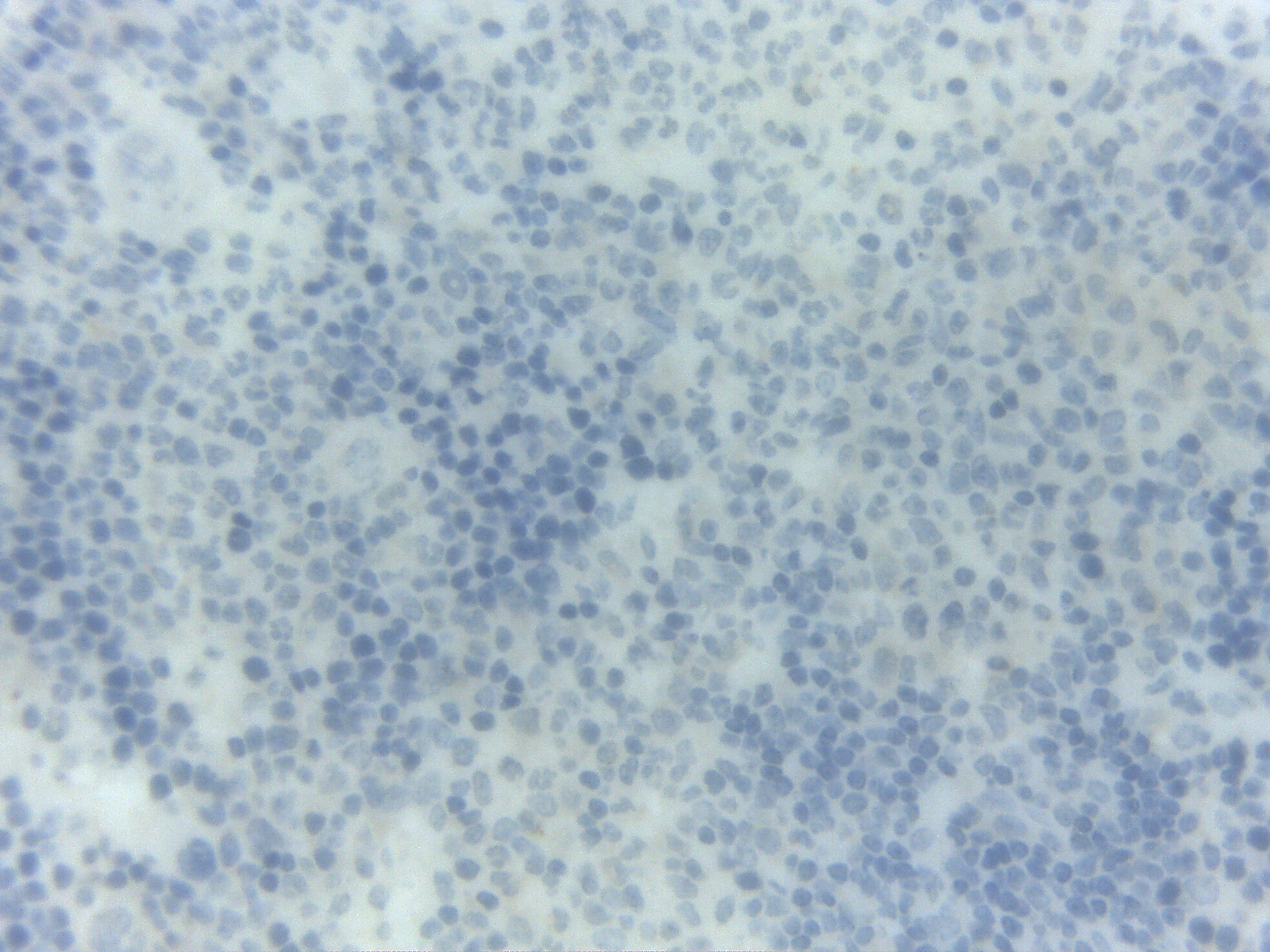

Supplement: S12 Fig — (ZIP) [file pone.0188960.s025.zip › Ly-6G IHC image24 hours/24h-1-4.jpg]

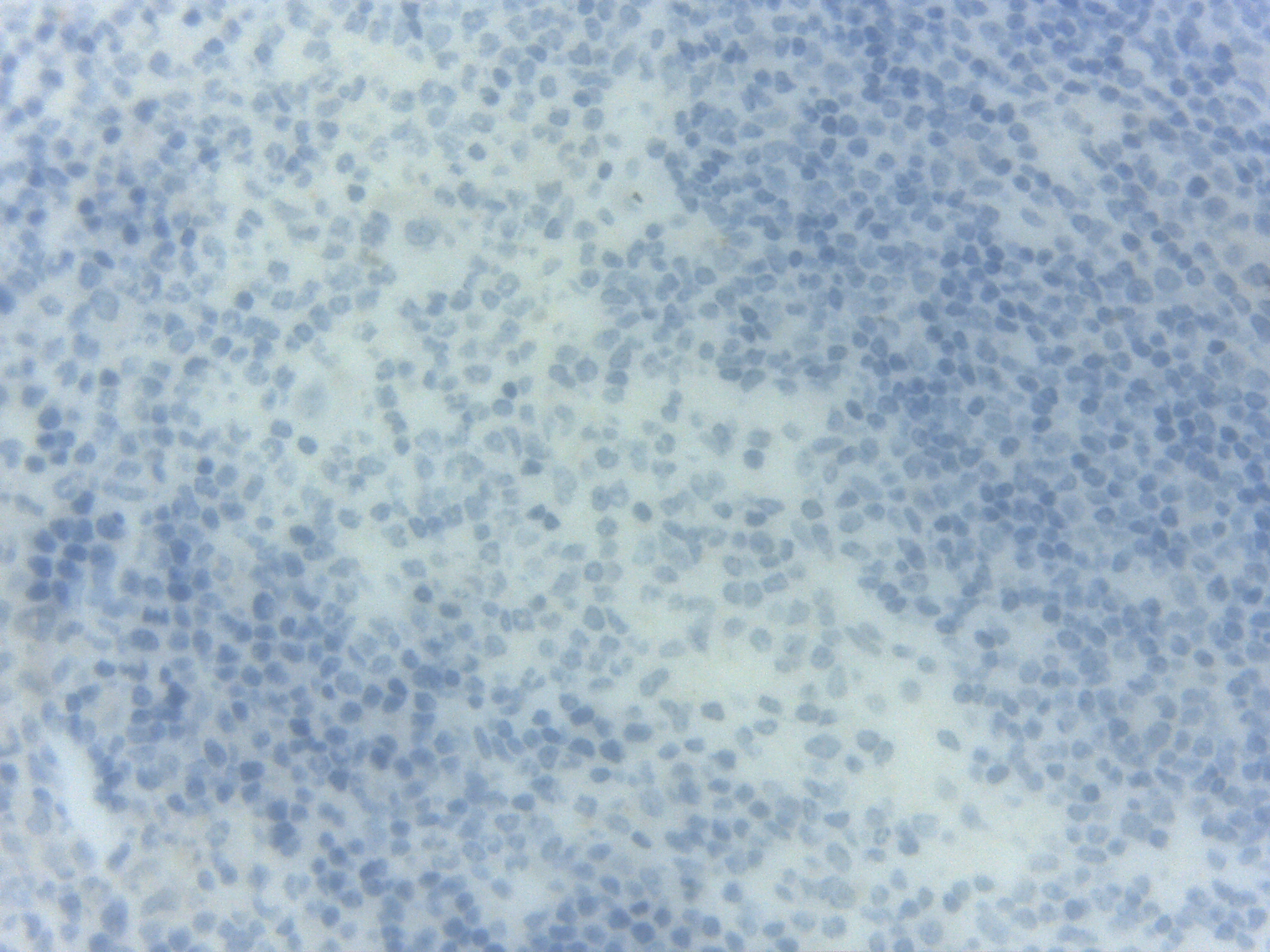

Supplement: S12 Fig — (ZIP) [file pone.0188960.s025.zip › Ly-6G IHC image24 hours/24h-1-5.jpg]

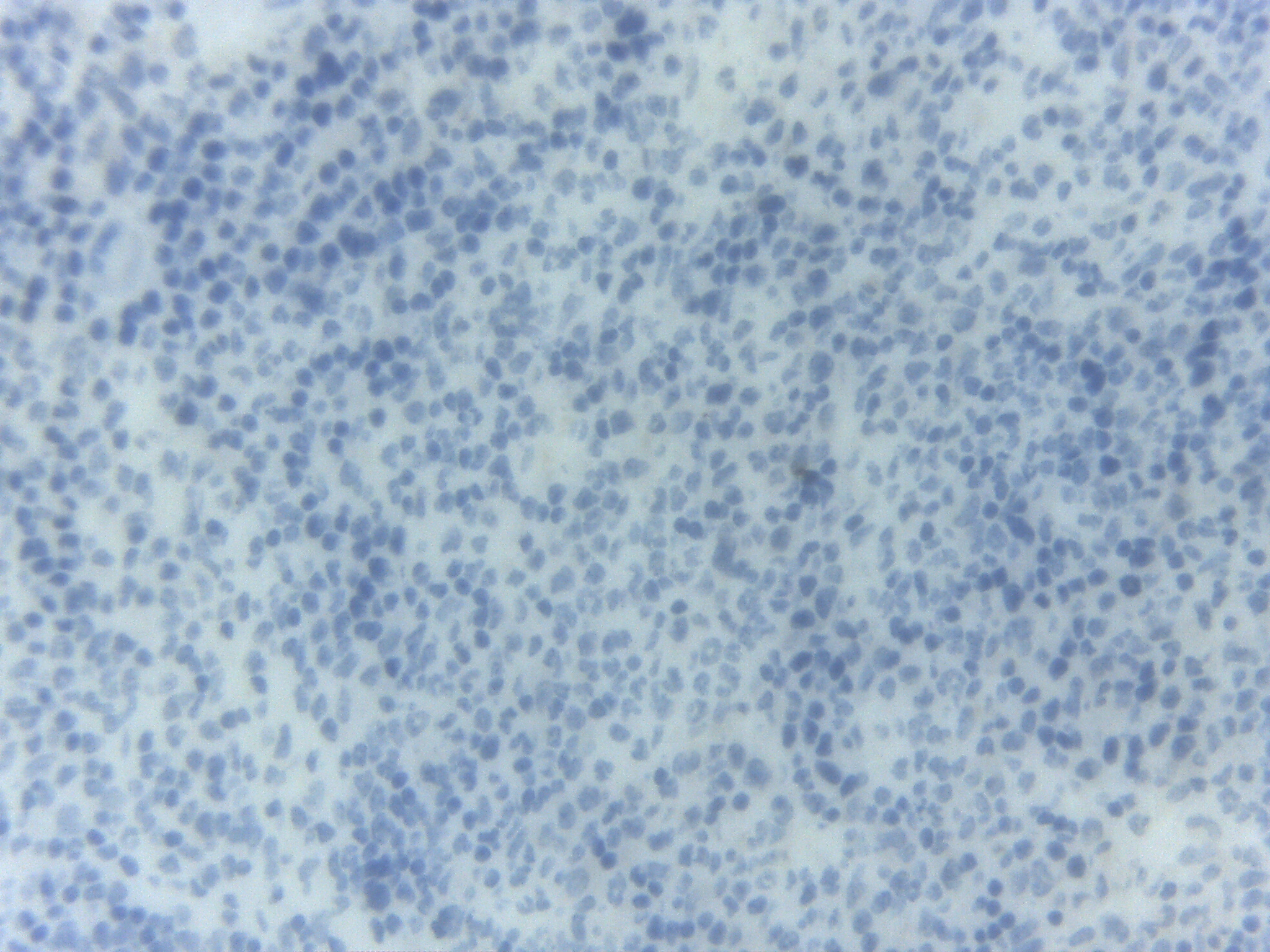

Supplement: S12 Fig — (ZIP) [file pone.0188960.s025.zip › Ly-6G IHC image24 hours/24h-2-1.jpg]

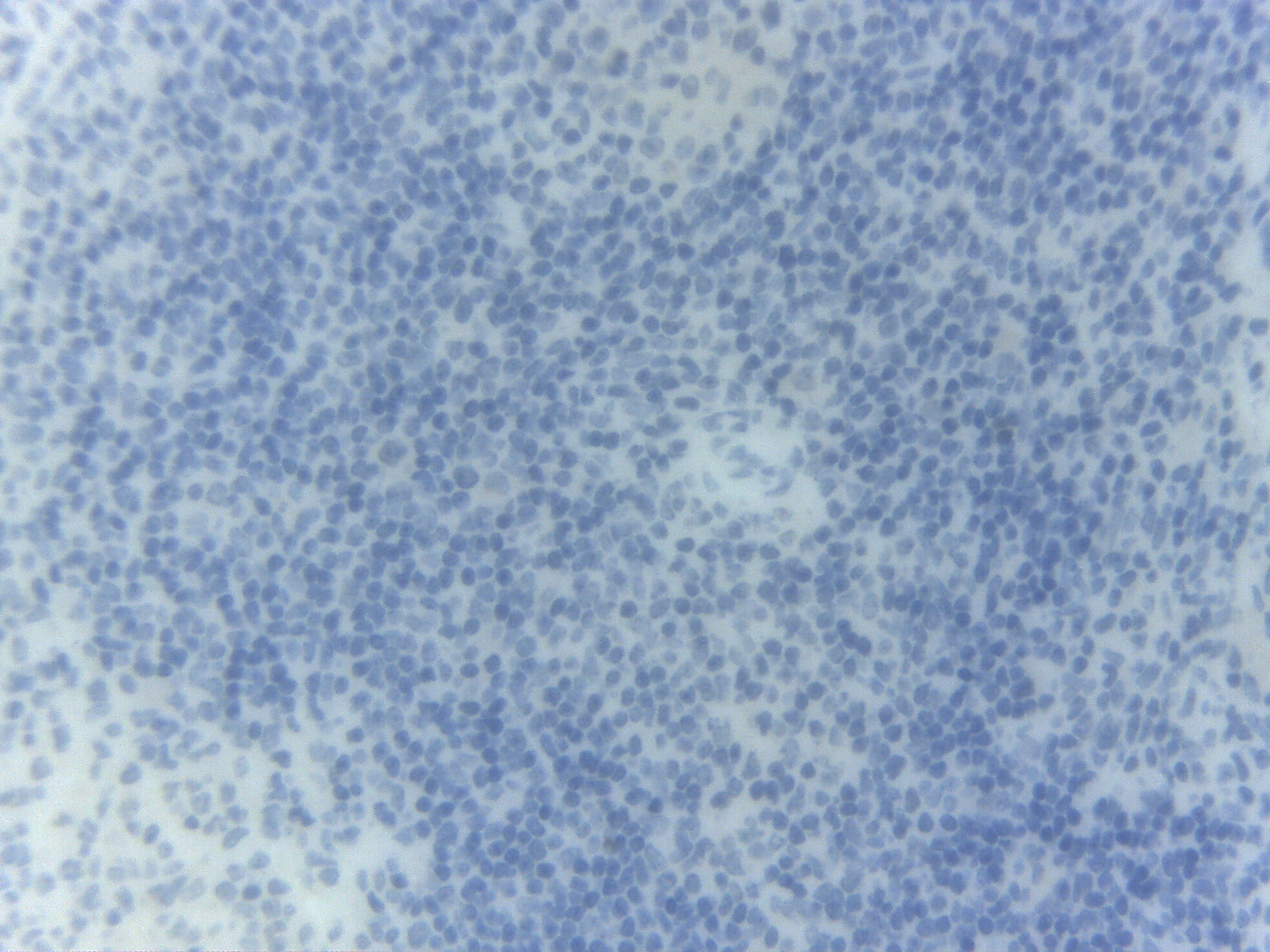

Supplement: S12 Fig — (ZIP) [file pone.0188960.s025.zip › Ly-6G IHC image24 hours/24h-2-2.jpg]

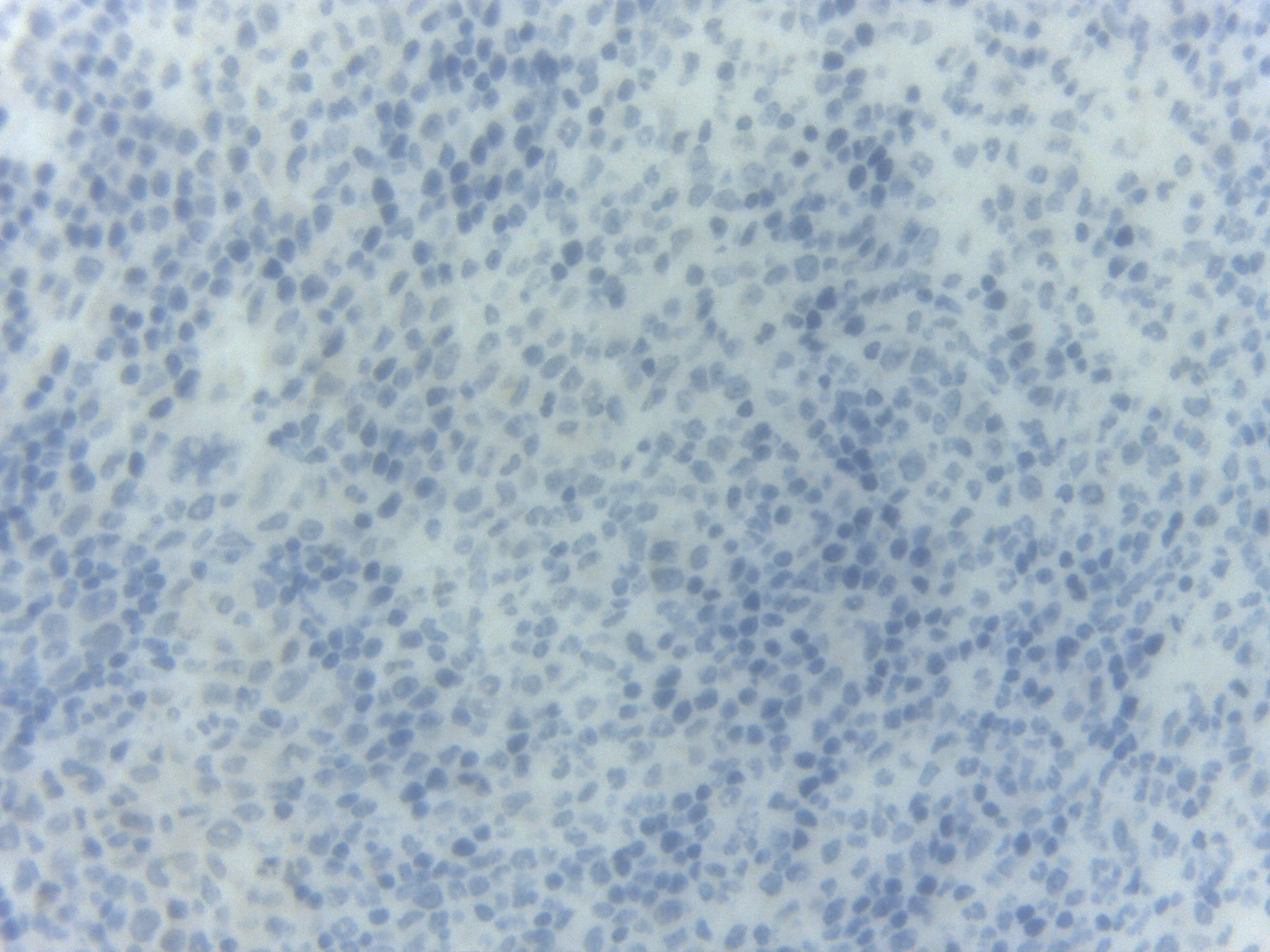

Supplement: S12 Fig — (ZIP) [file pone.0188960.s025.zip › Ly-6G IHC image24 hours/24h-2-3.jpg]

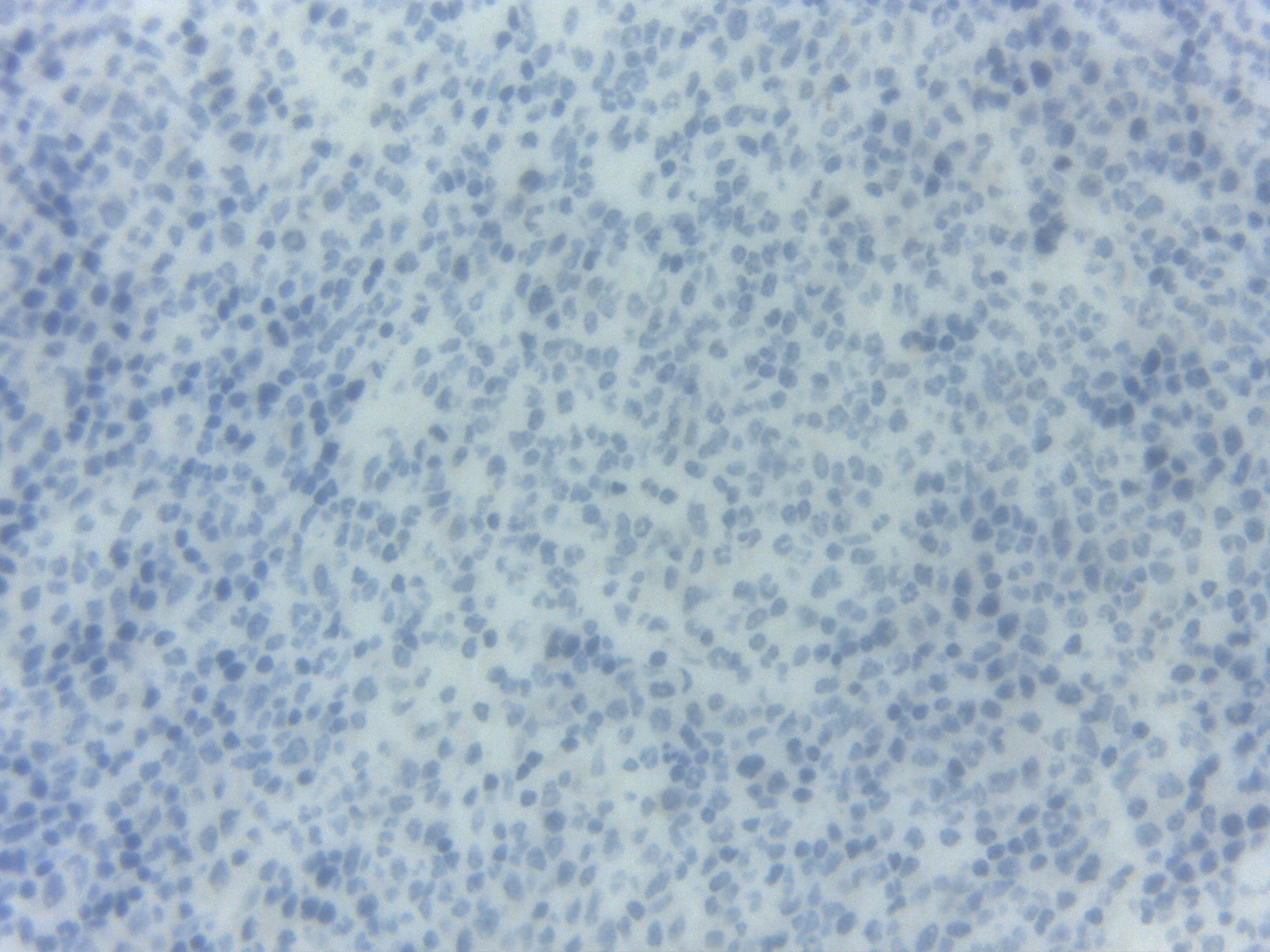

Supplement: S12 Fig — (ZIP) [file pone.0188960.s025.zip › Ly-6G IHC image24 hours/24h-2-4.jpg]

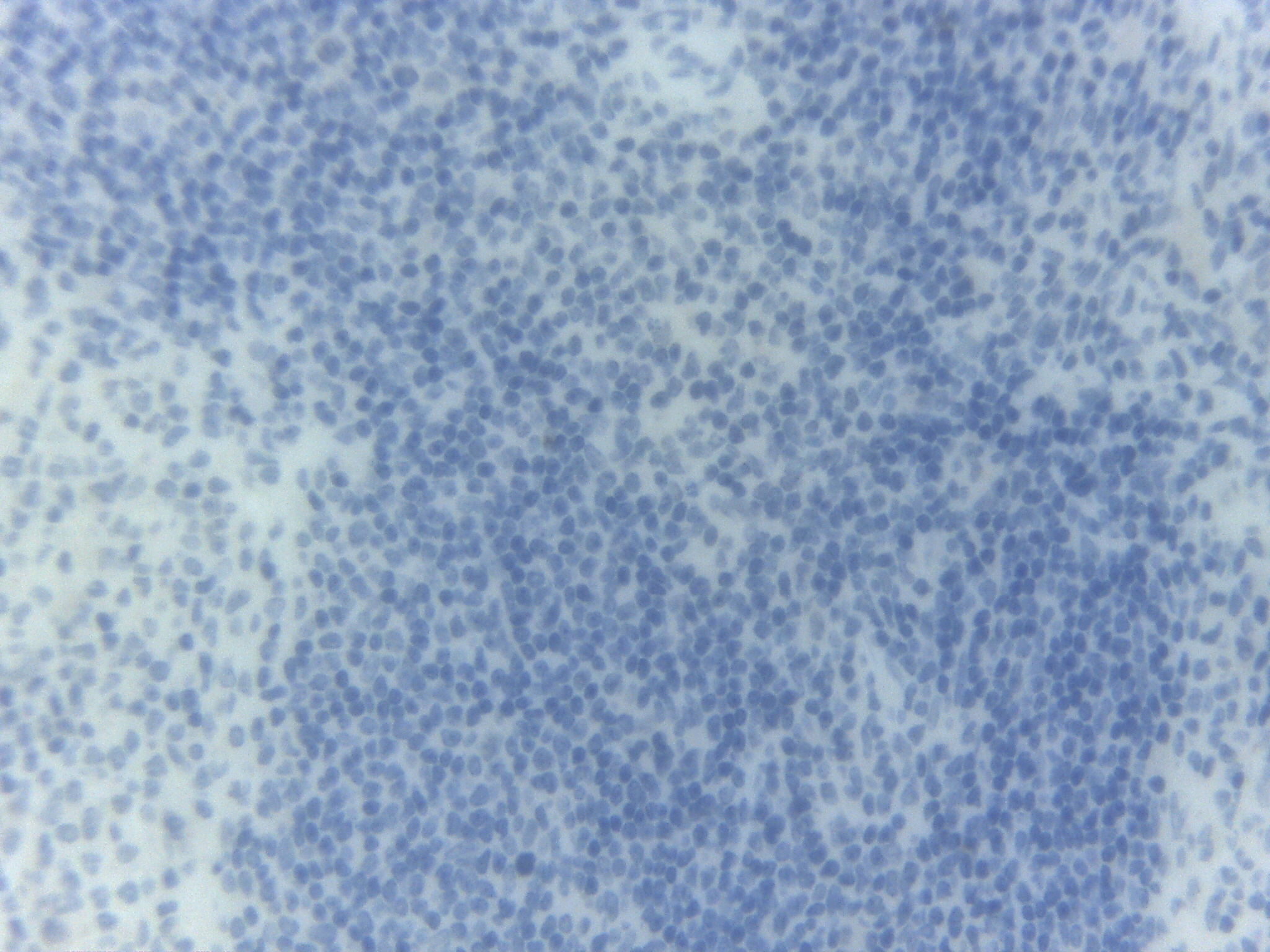

Supplement: S12 Fig — (ZIP) [file pone.0188960.s025.zip › Ly-6G IHC image24 hours/24h-2-5.jpg]

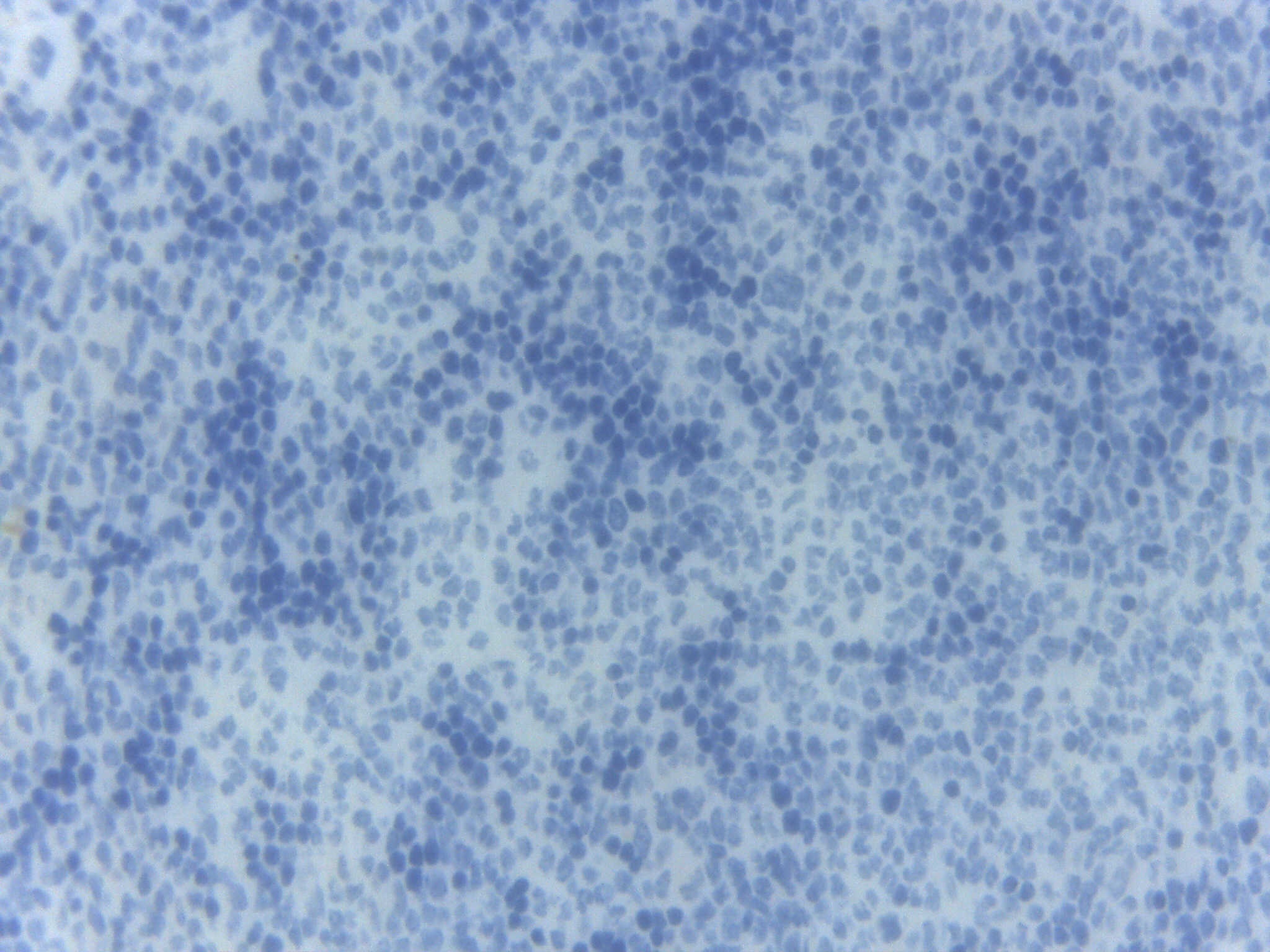

Supplement: S12 Fig — (ZIP) [file pone.0188960.s025.zip › Ly-6G IHC image24 hours/24h-3-1.jpg]

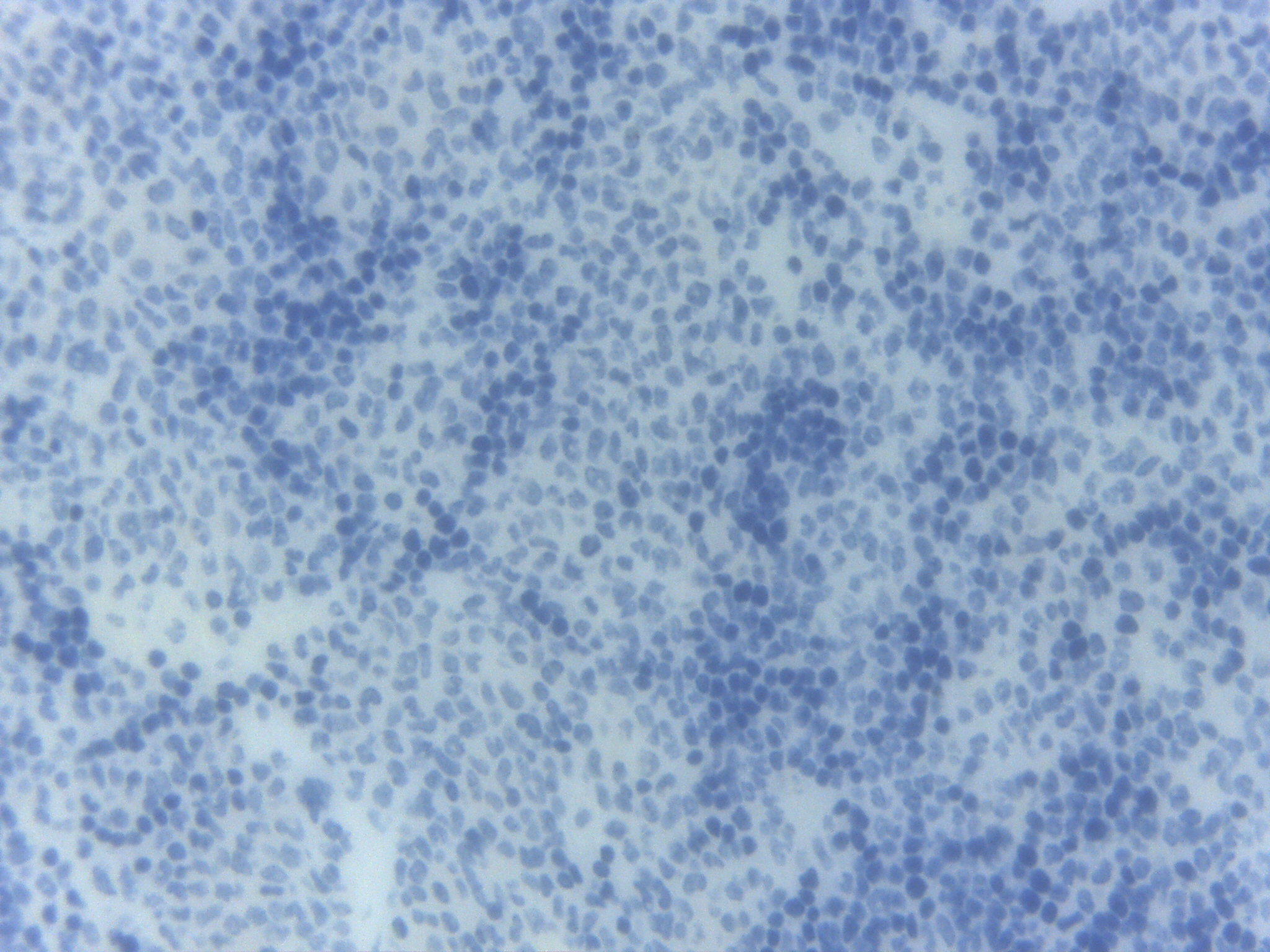

Supplement: S12 Fig — (ZIP) [file pone.0188960.s025.zip › Ly-6G IHC image24 hours/24h-3-2.jpg]

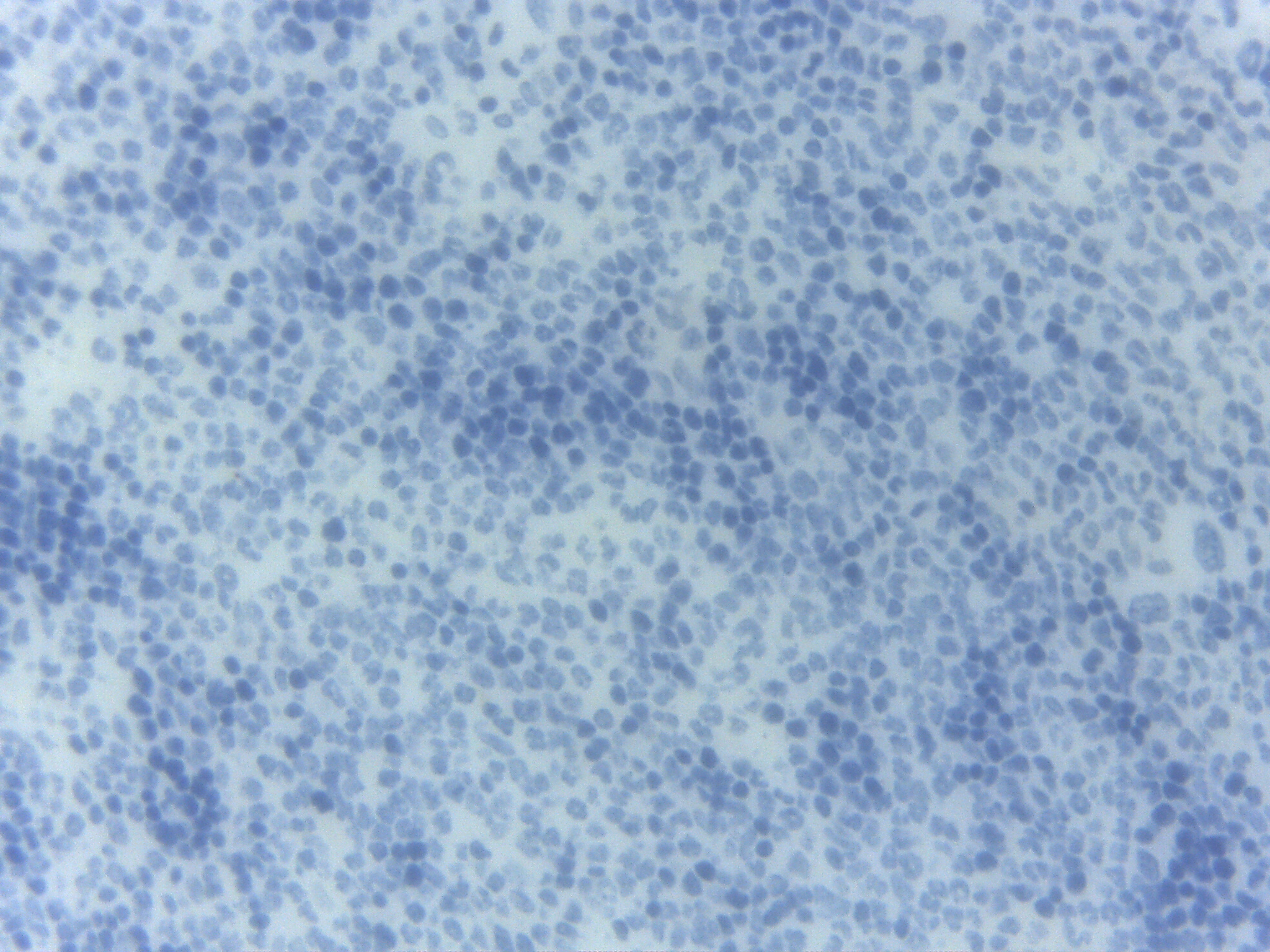

Supplement: S12 Fig — (ZIP) [file pone.0188960.s025.zip › Ly-6G IHC image24 hours/24h-3-3.jpg]

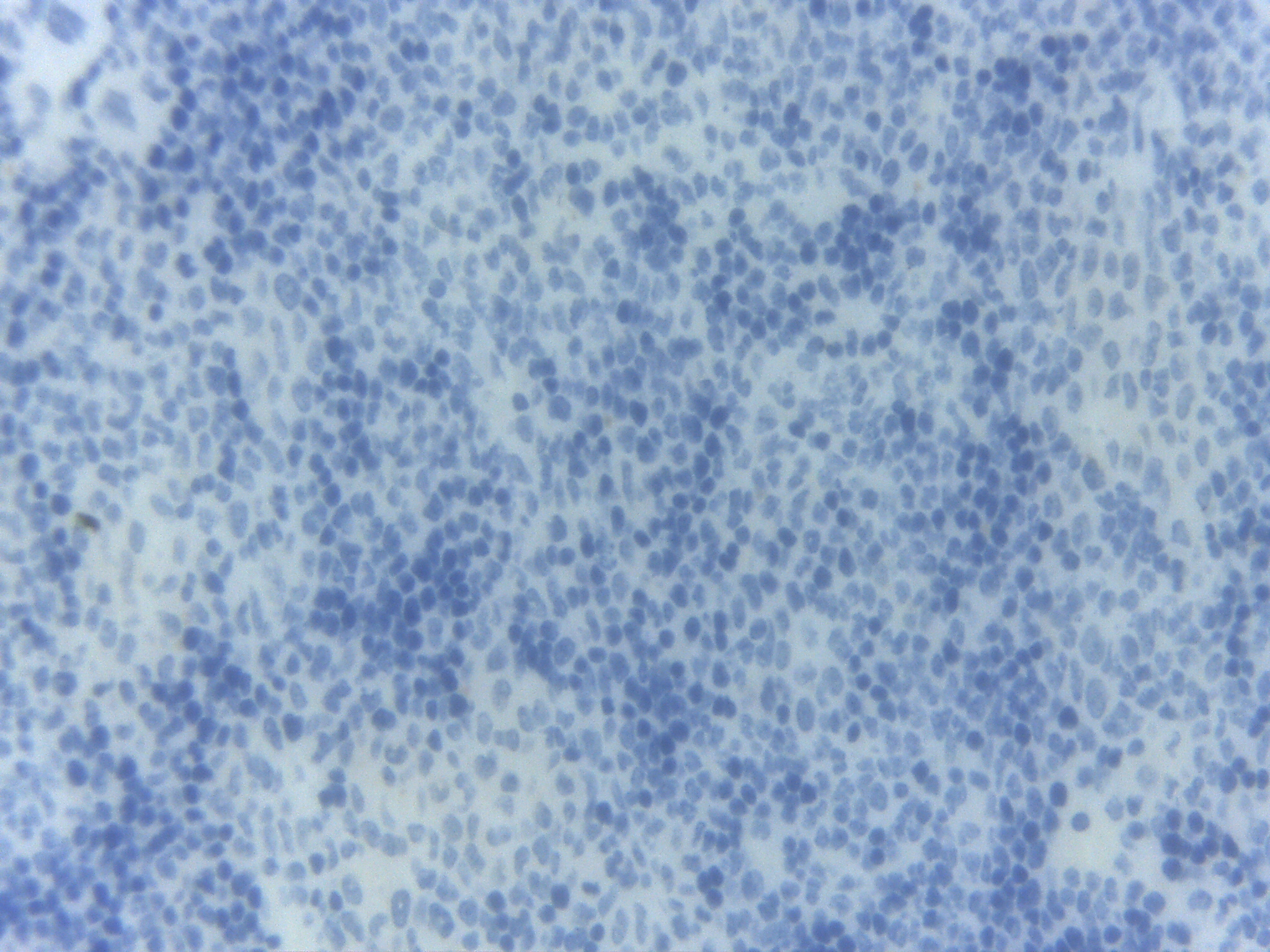

Supplement: S12 Fig — (ZIP) [file pone.0188960.s025.zip › Ly-6G IHC image24 hours/24h-3-4.jpg]

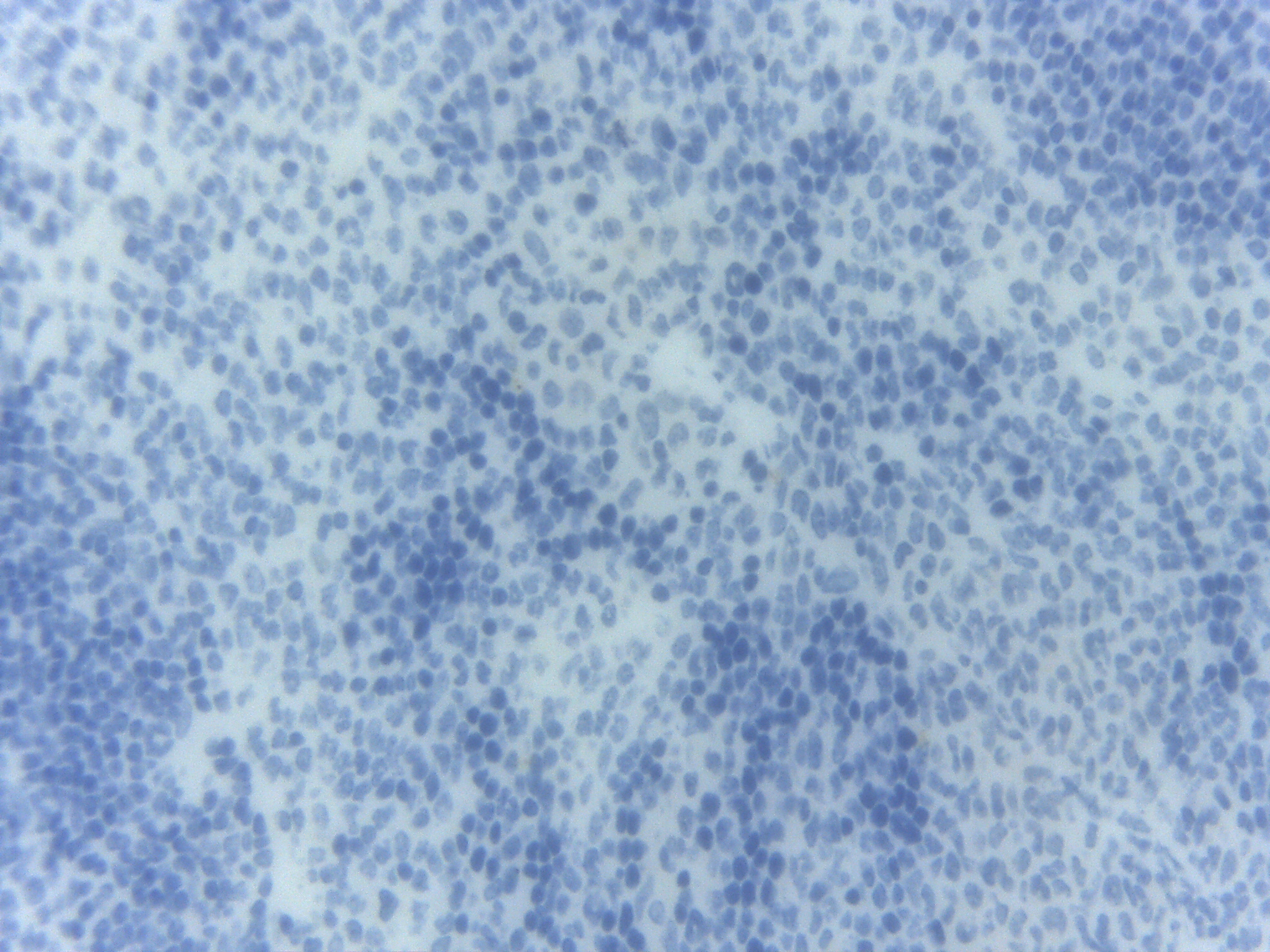

Supplement: S12 Fig — (ZIP) [file pone.0188960.s025.zip › Ly-6G IHC image24 hours/24h-3-5.jpg]

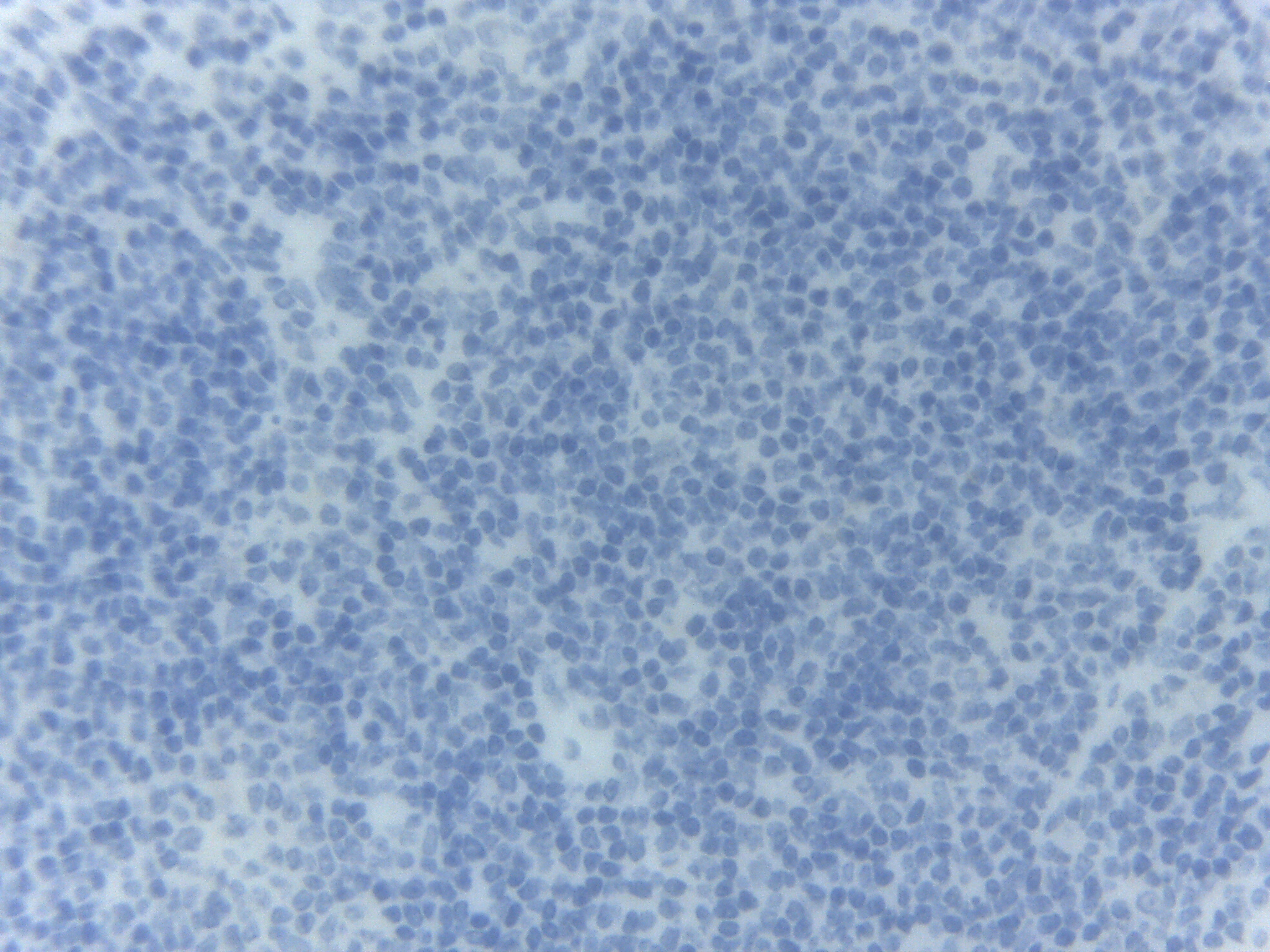

Supplement: S12 Fig — (ZIP) [file pone.0188960.s025.zip › Ly-6G IHC image24 hours/24h-4-1.jpg]

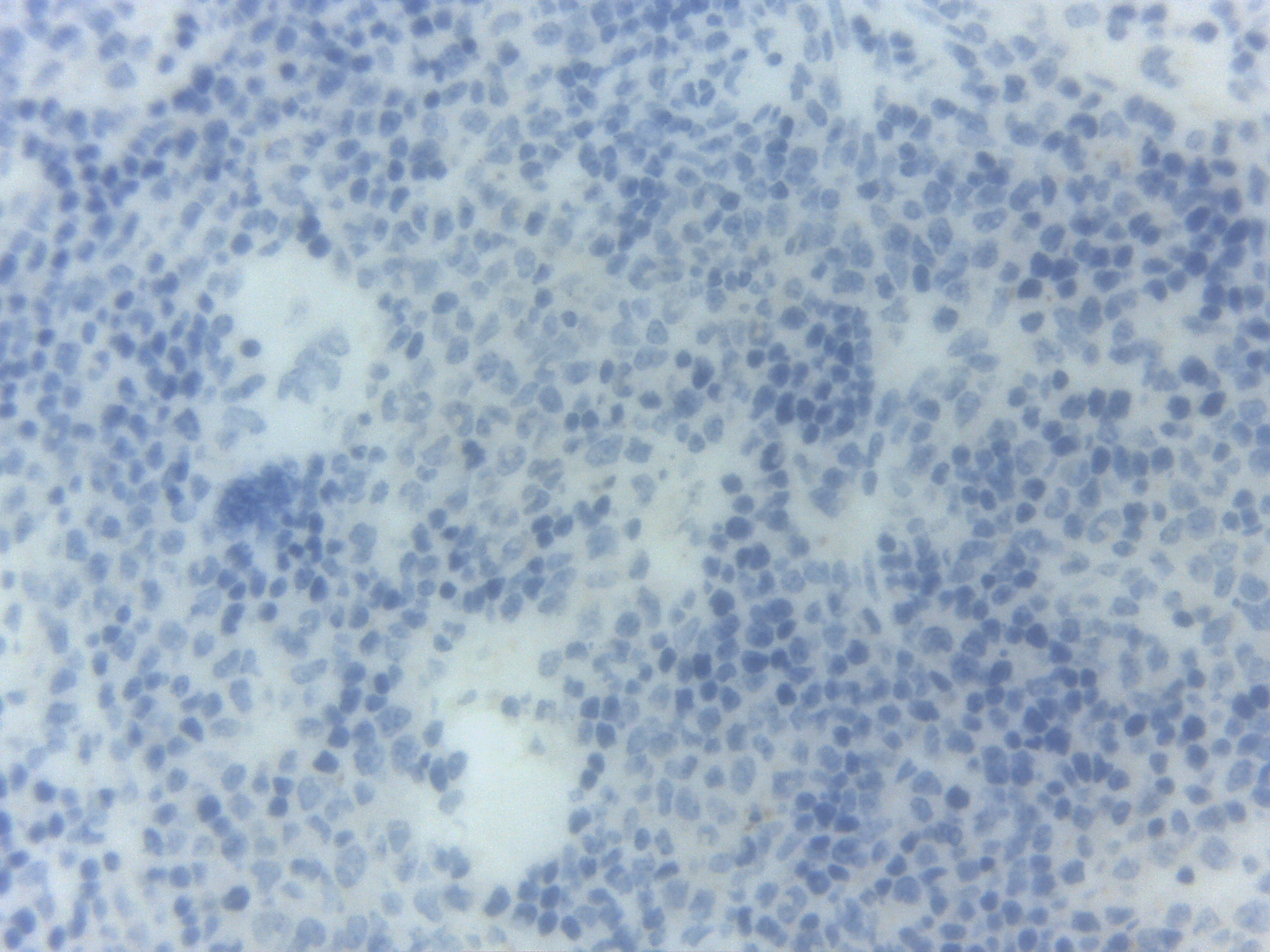

Supplement: S12 Fig — (ZIP) [file pone.0188960.s025.zip › Ly-6G IHC image24 hours/24h-4-2.jpg]

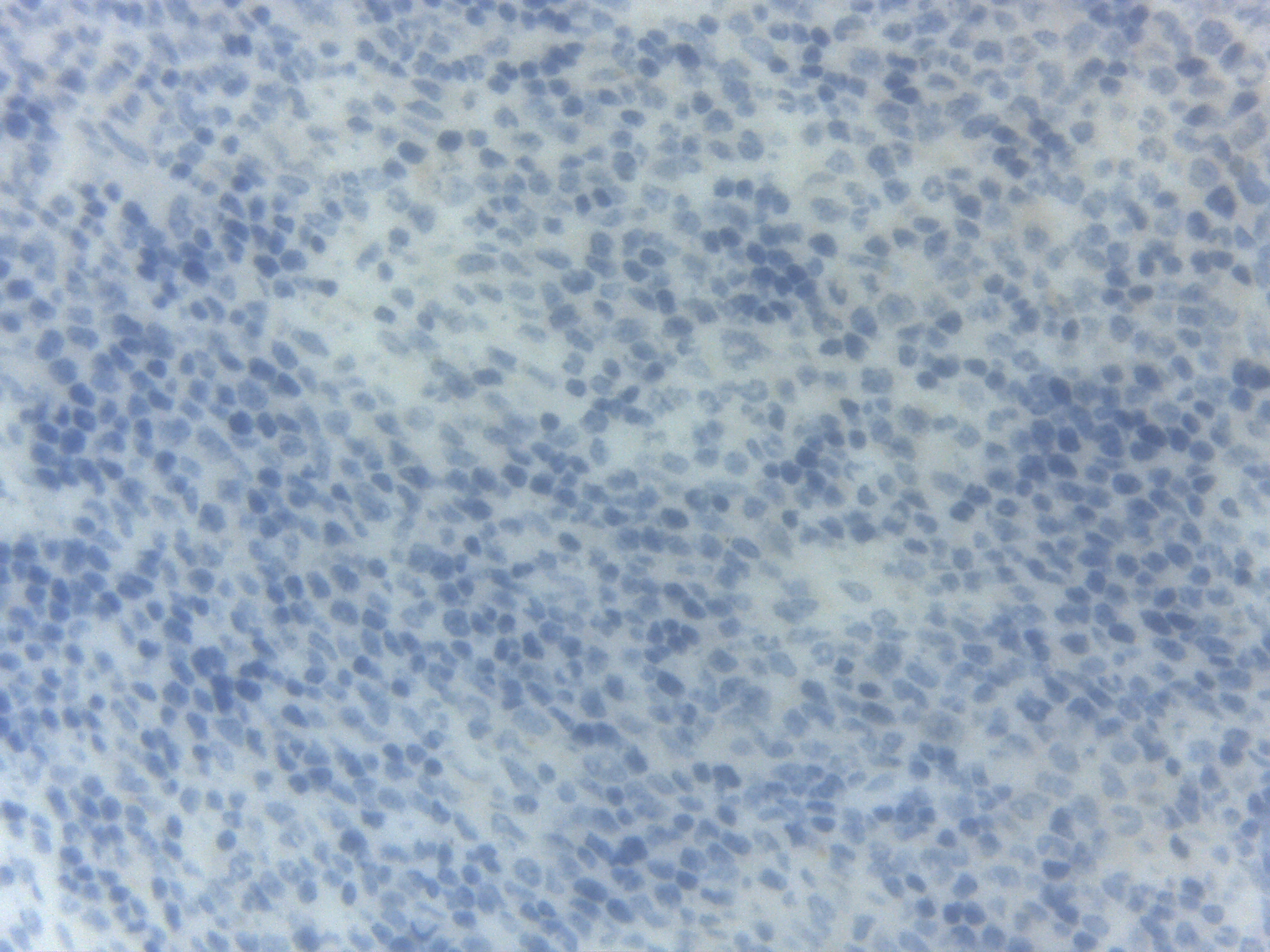

Supplement: S12 Fig — (ZIP) [file pone.0188960.s025.zip › Ly-6G IHC image24 hours/24h-4-3.jpg]

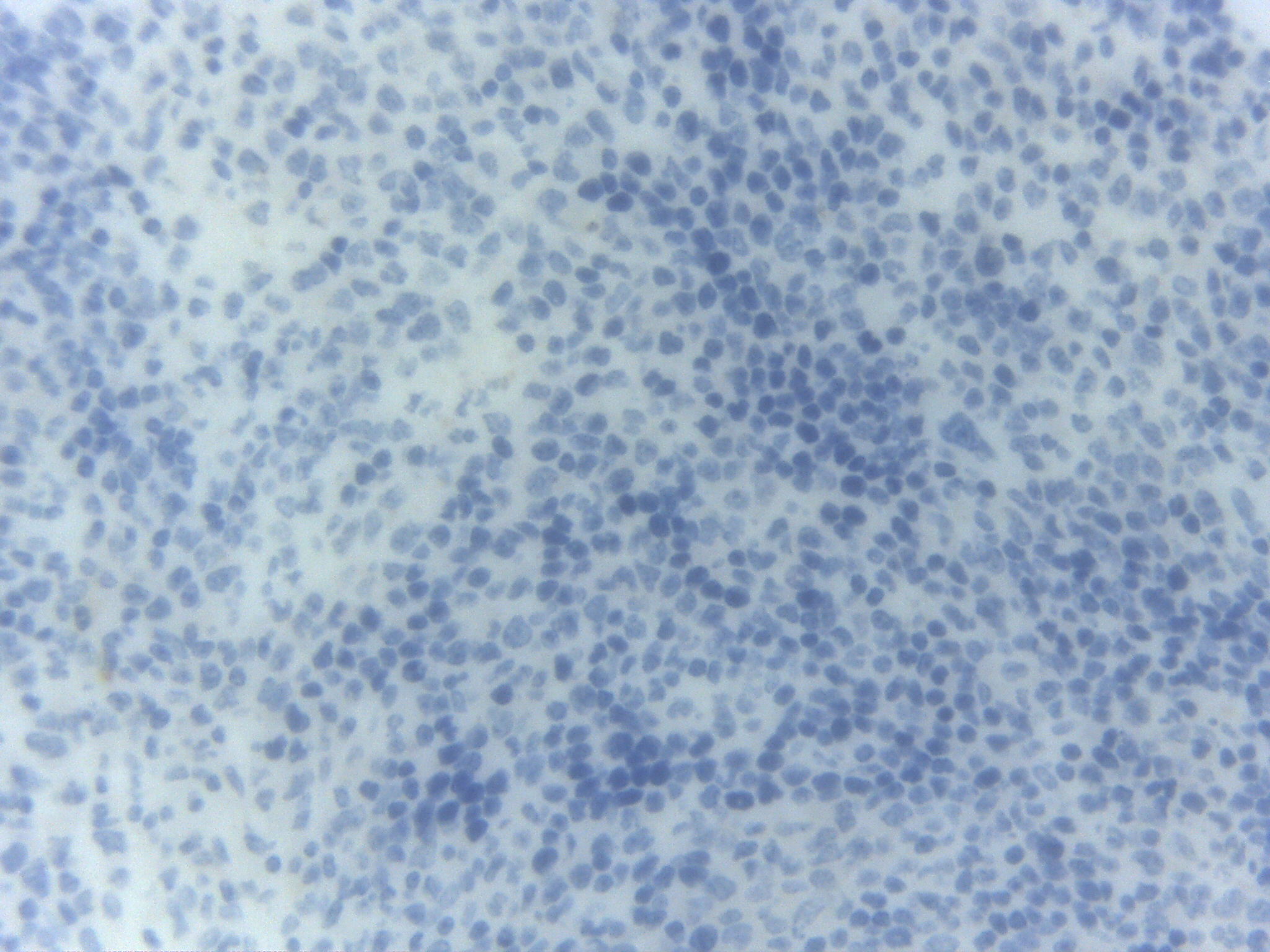

Supplement: S12 Fig — (ZIP) [file pone.0188960.s025.zip › Ly-6G IHC image24 hours/24h-4-4.jpg]

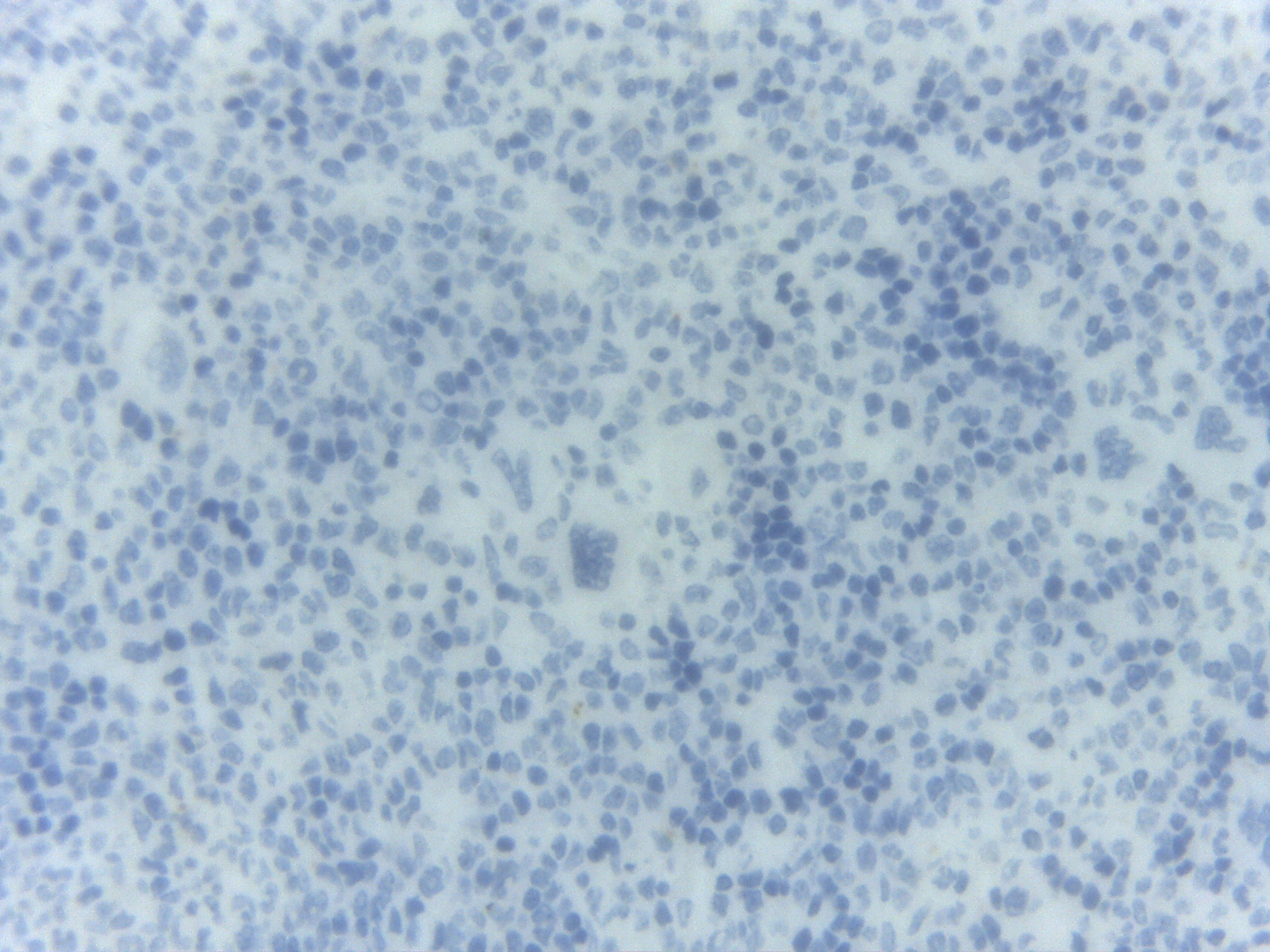

Supplement: S12 Fig — (ZIP) [file pone.0188960.s025.zip › Ly-6G IHC image24 hours/24h-4-5.jpg]

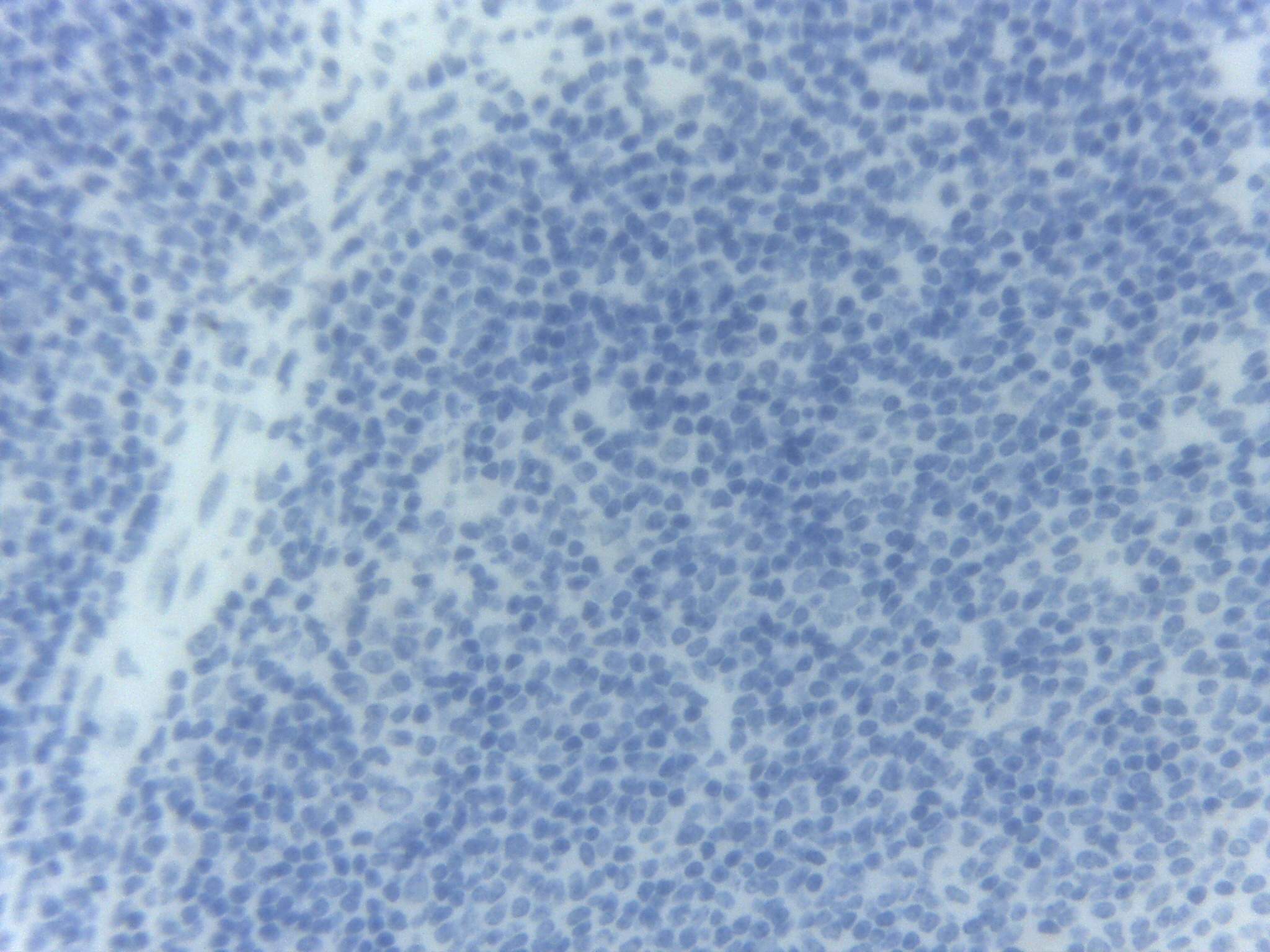

Supplement: S12 Fig — (ZIP) [file pone.0188960.s025.zip › Ly-6G IHC image24 hours/24h-5-1.jpg]

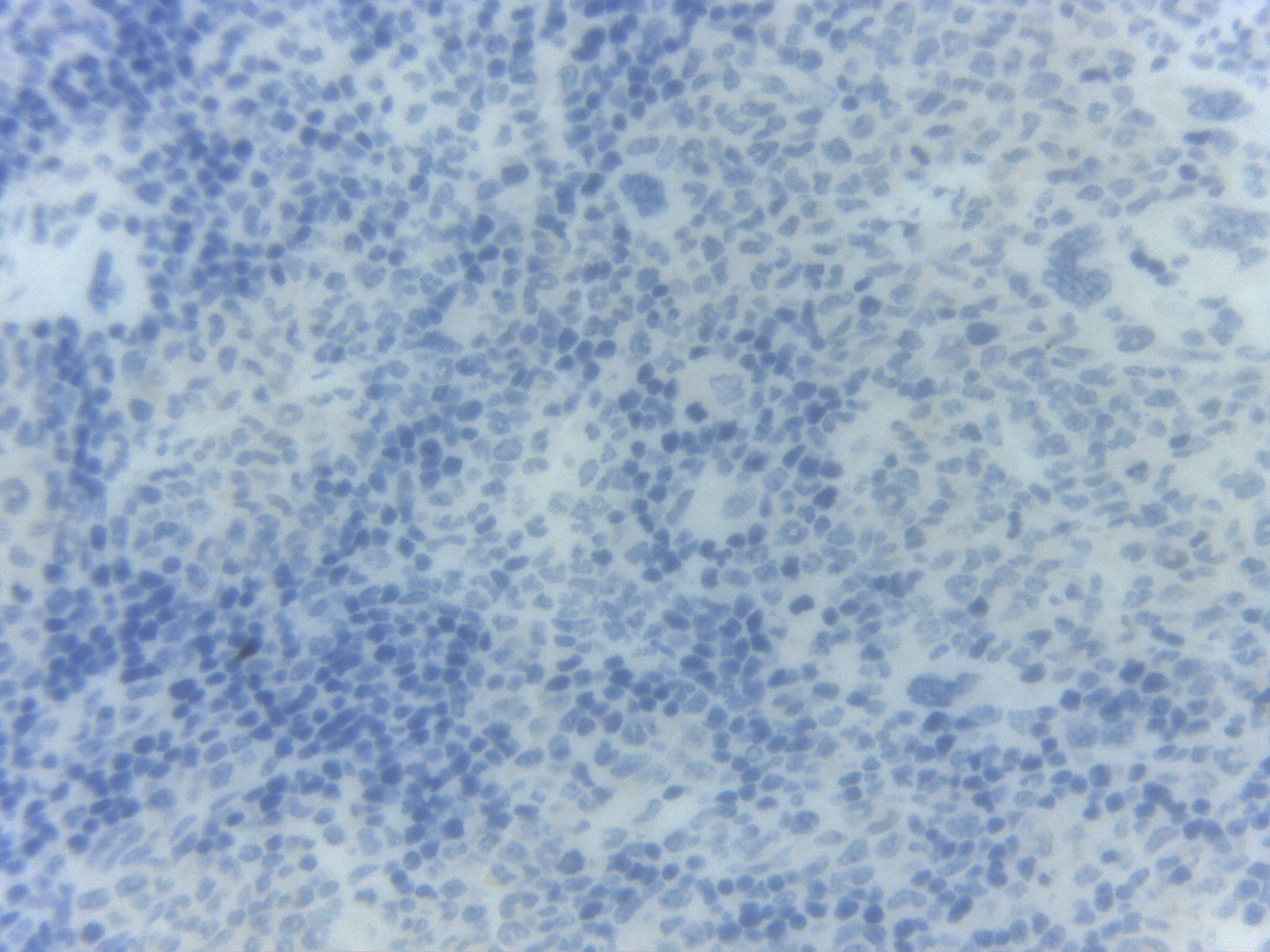

Supplement: S12 Fig — (ZIP) [file pone.0188960.s025.zip › Ly-6G IHC image24 hours/24h-5-2.jpg]

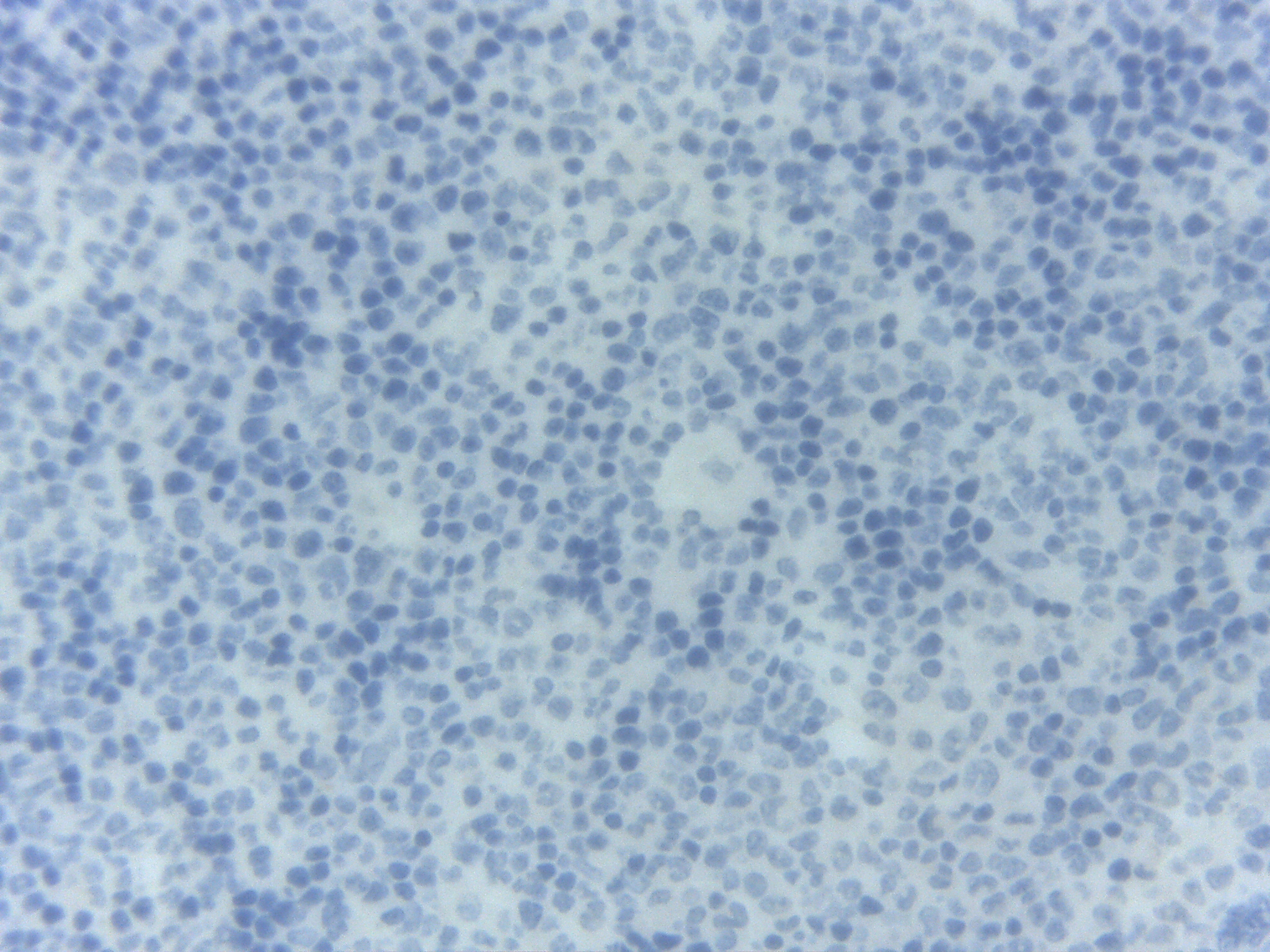

Supplement: S12 Fig — (ZIP) [file pone.0188960.s025.zip › Ly-6G IHC image24 hours/24h-5-3.jpg]

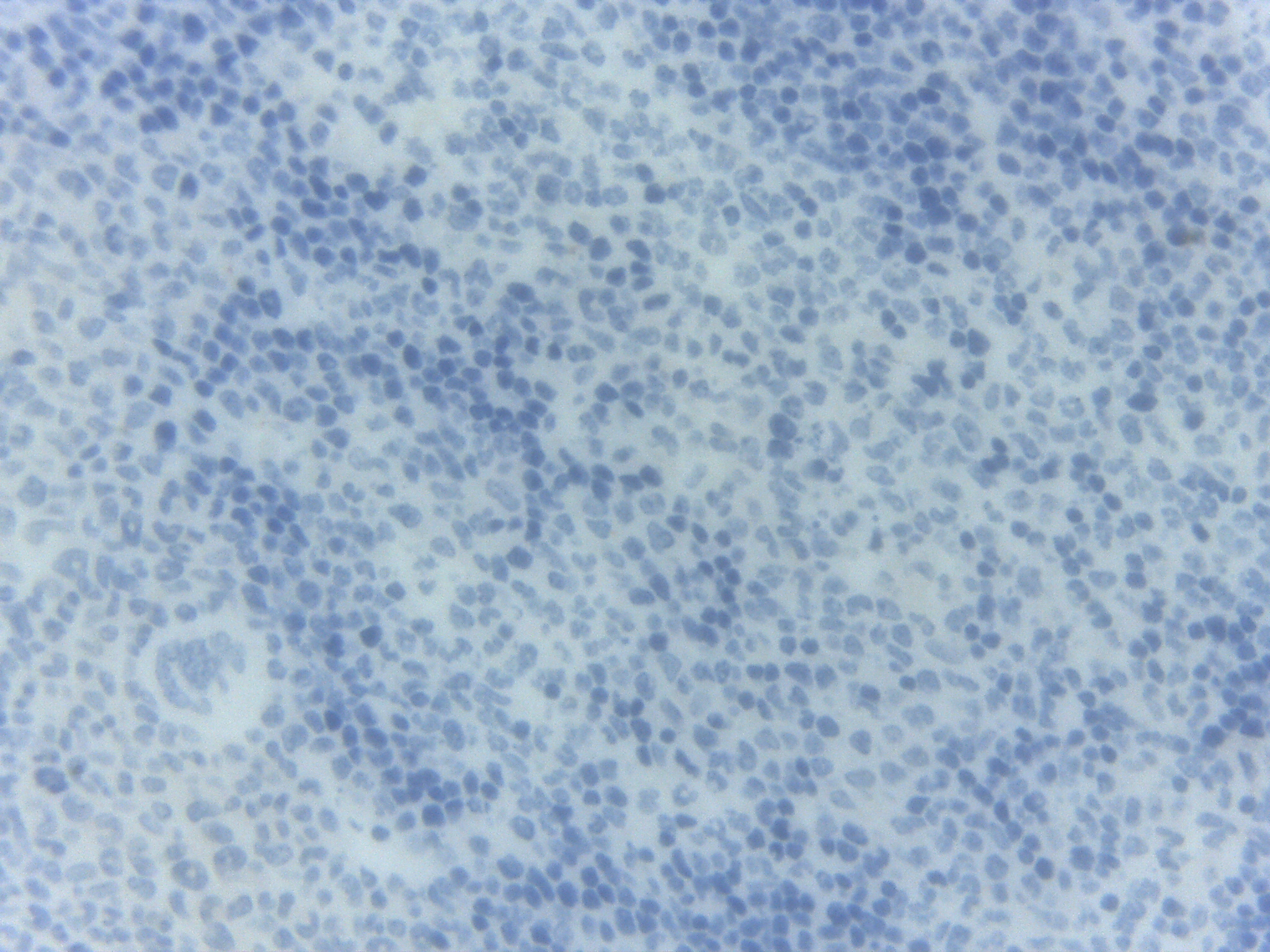

Supplement: S12 Fig — (ZIP) [file pone.0188960.s025.zip › Ly-6G IHC image24 hours/24h-5-4.jpg]

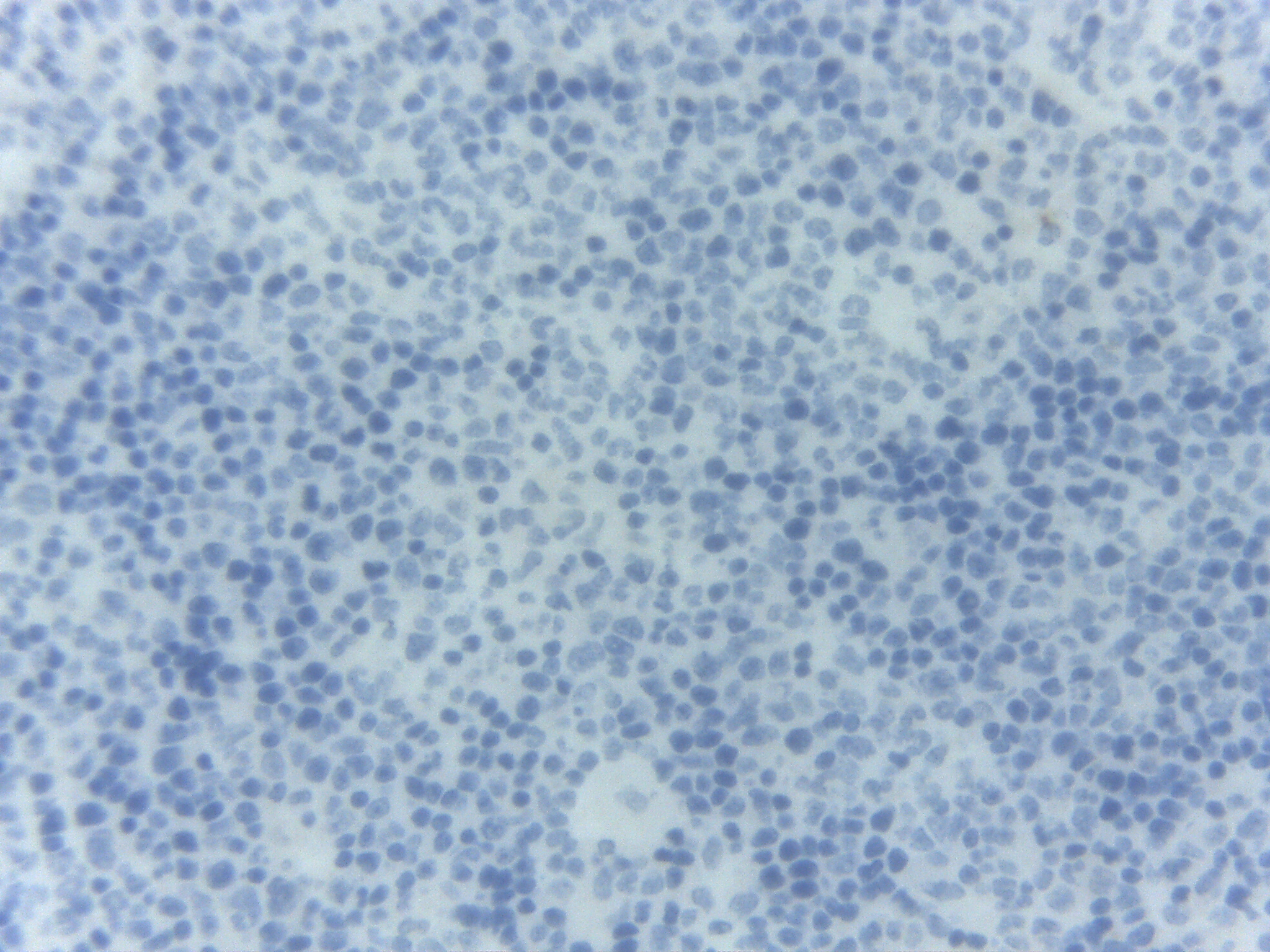

Supplement: S12 Fig — (ZIP) [file pone.0188960.s025.zip › Ly-6G IHC image24 hours/24h-5-5.jpg]

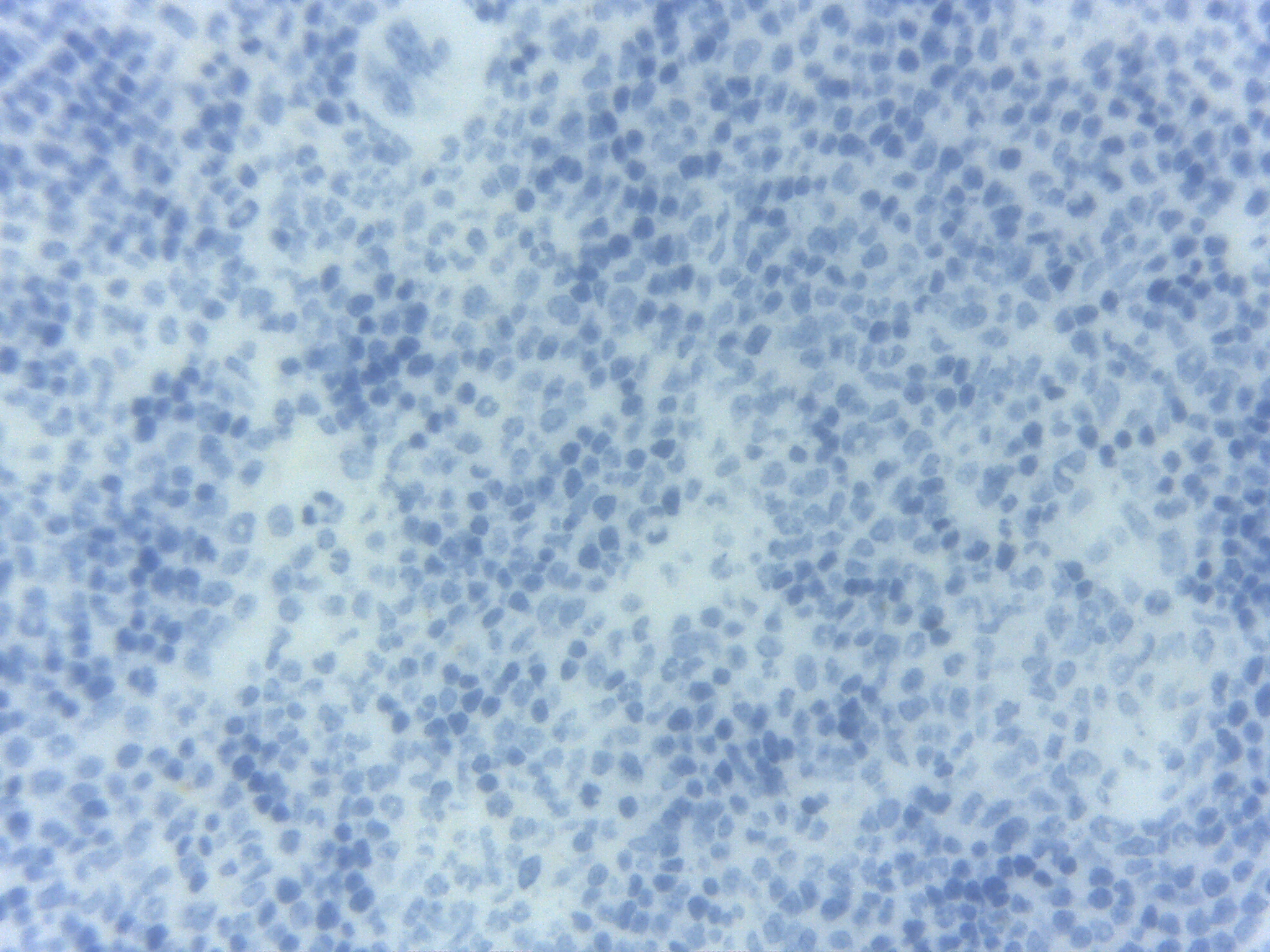

Supplement: S12 Fig — (ZIP) [file pone.0188960.s025.zip › Ly-6G IHC image24 hours/24h-6-1.jpg]

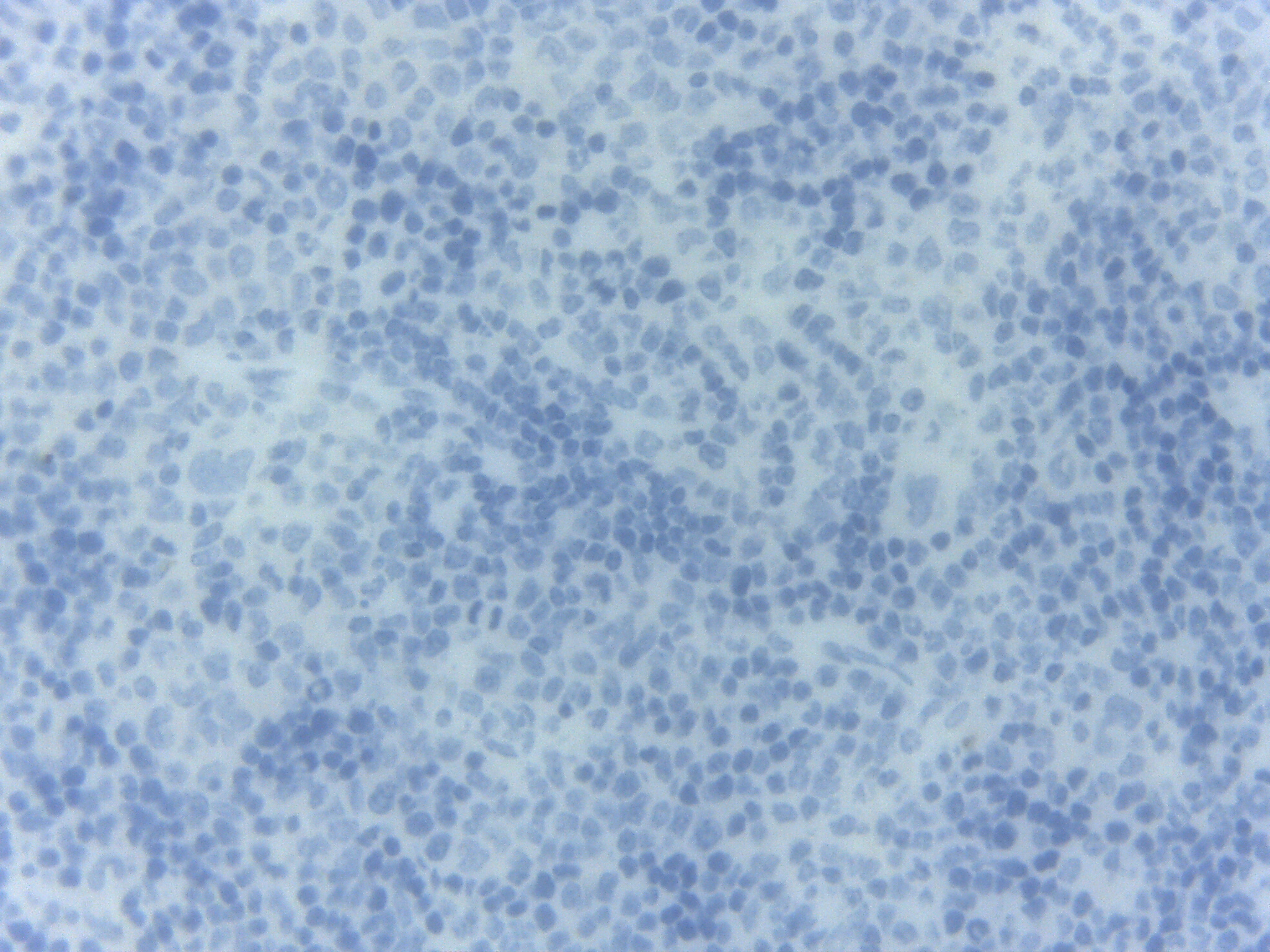

Supplement: S12 Fig — (ZIP) [file pone.0188960.s025.zip › Ly-6G IHC image24 hours/24h-6-2.jpg]

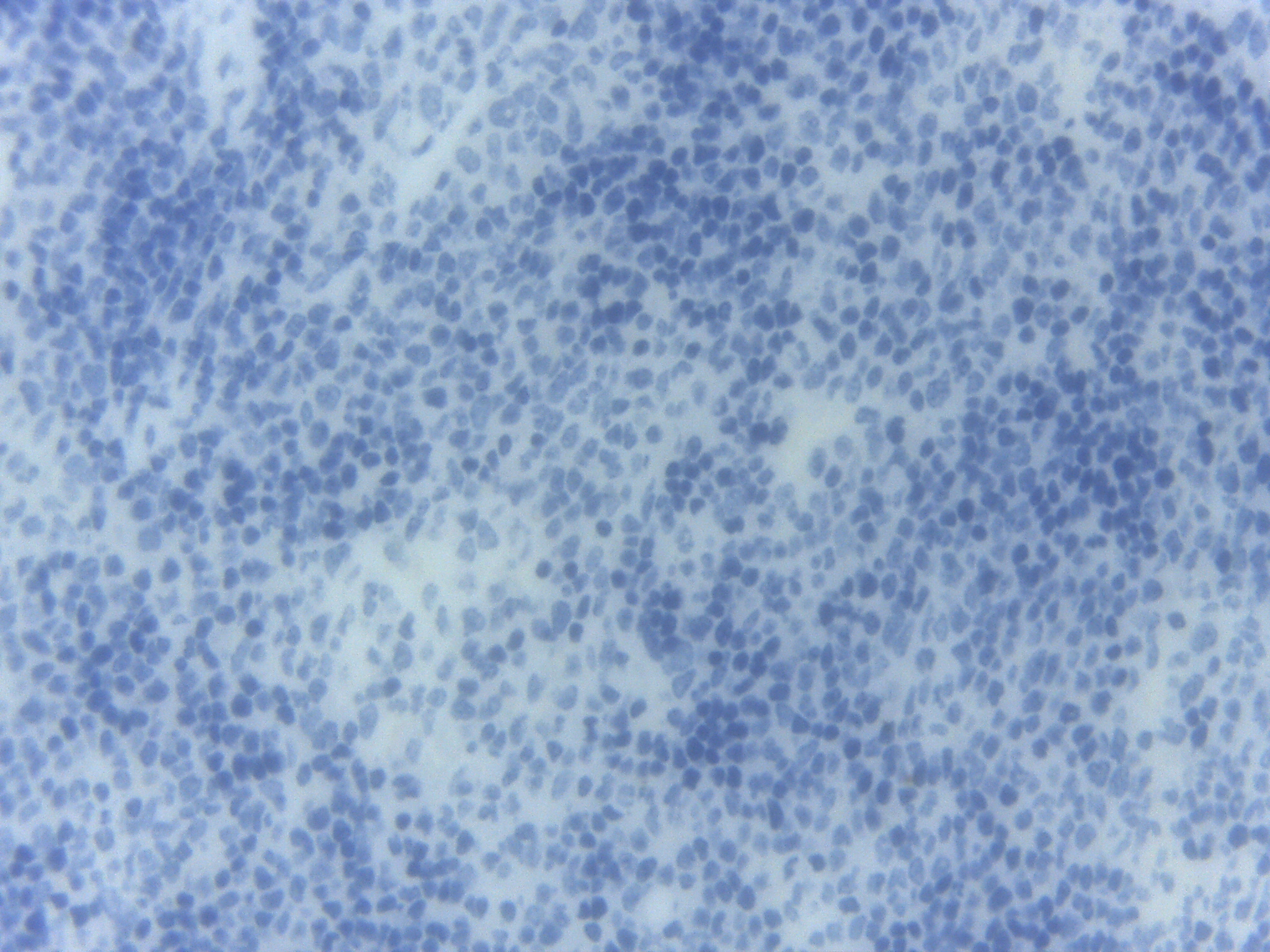

Supplement: S12 Fig — (ZIP) [file pone.0188960.s025.zip › Ly-6G IHC image24 hours/24h-6-3.jpg]

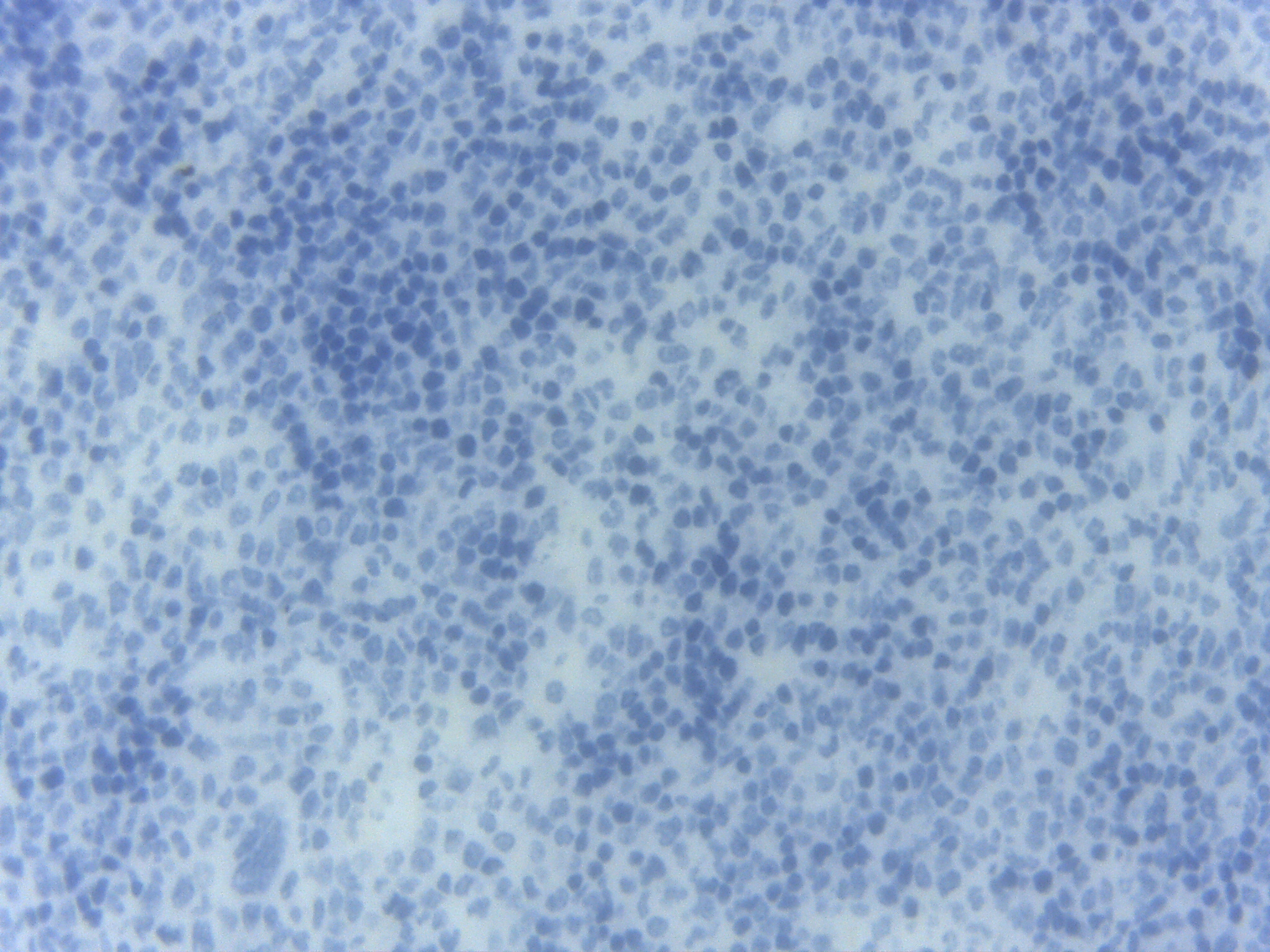

Supplement: S12 Fig — (ZIP) [file pone.0188960.s025.zip › Ly-6G IHC image24 hours/24h-6-4.jpg]

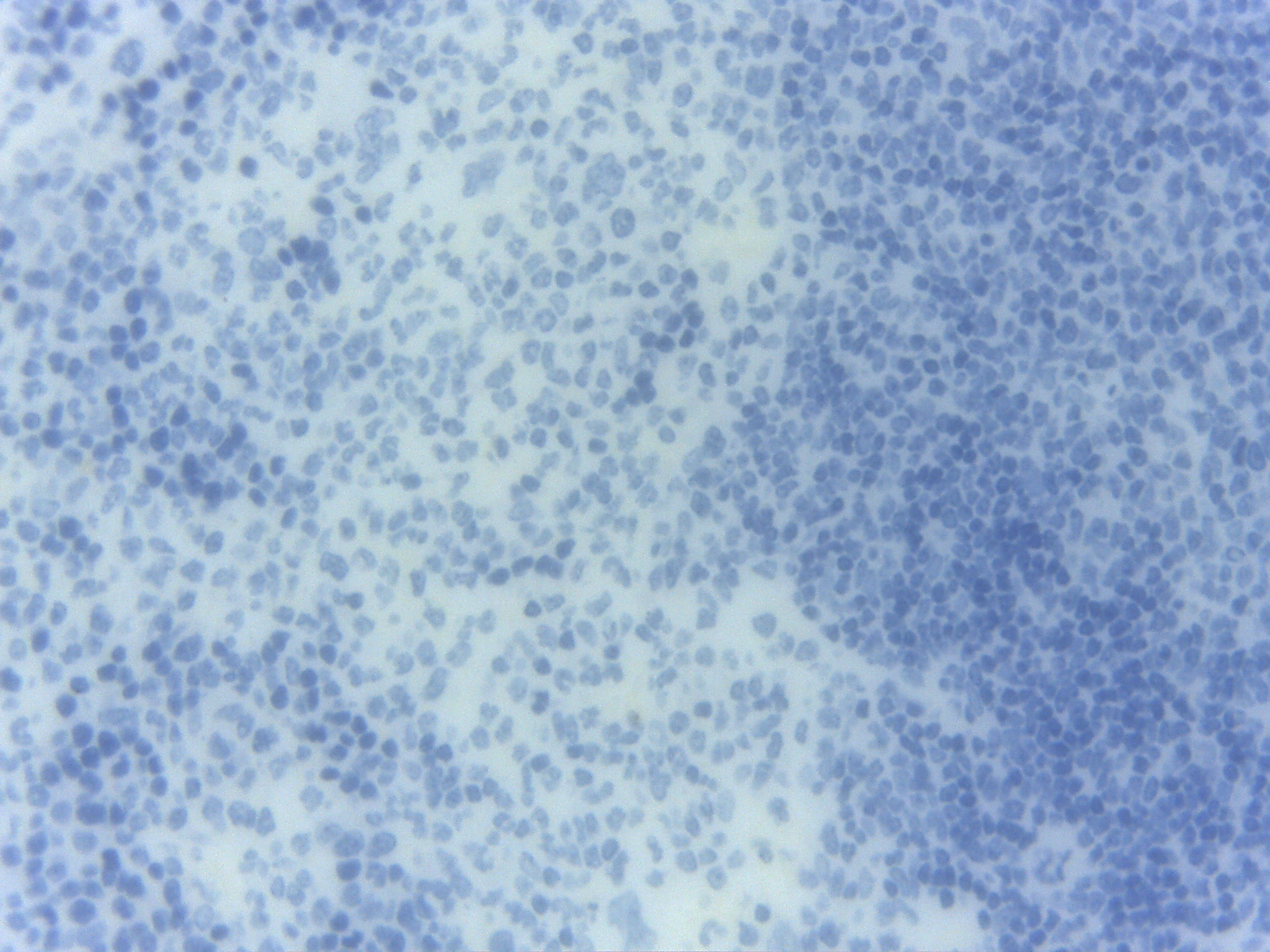

Supplement: S12 Fig — (ZIP) [file pone.0188960.s025.zip › Ly-6G IHC image24 hours/24h-6-5.jpg]

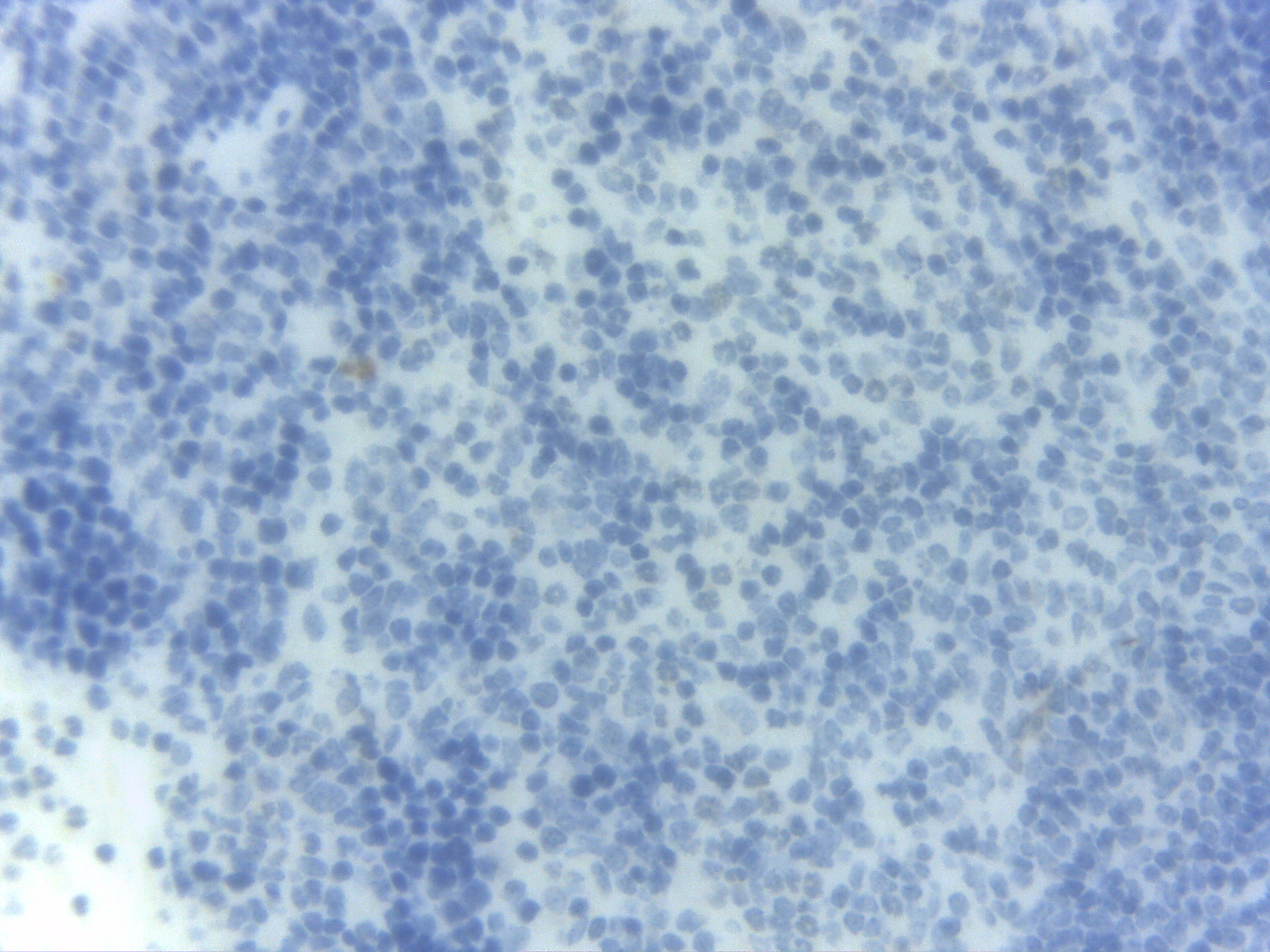

Supplement: S13 Fig — (ZIP) [file pone.0188960.s026.zip › Ly-6G IHC image CON/con-1-1.jpg]

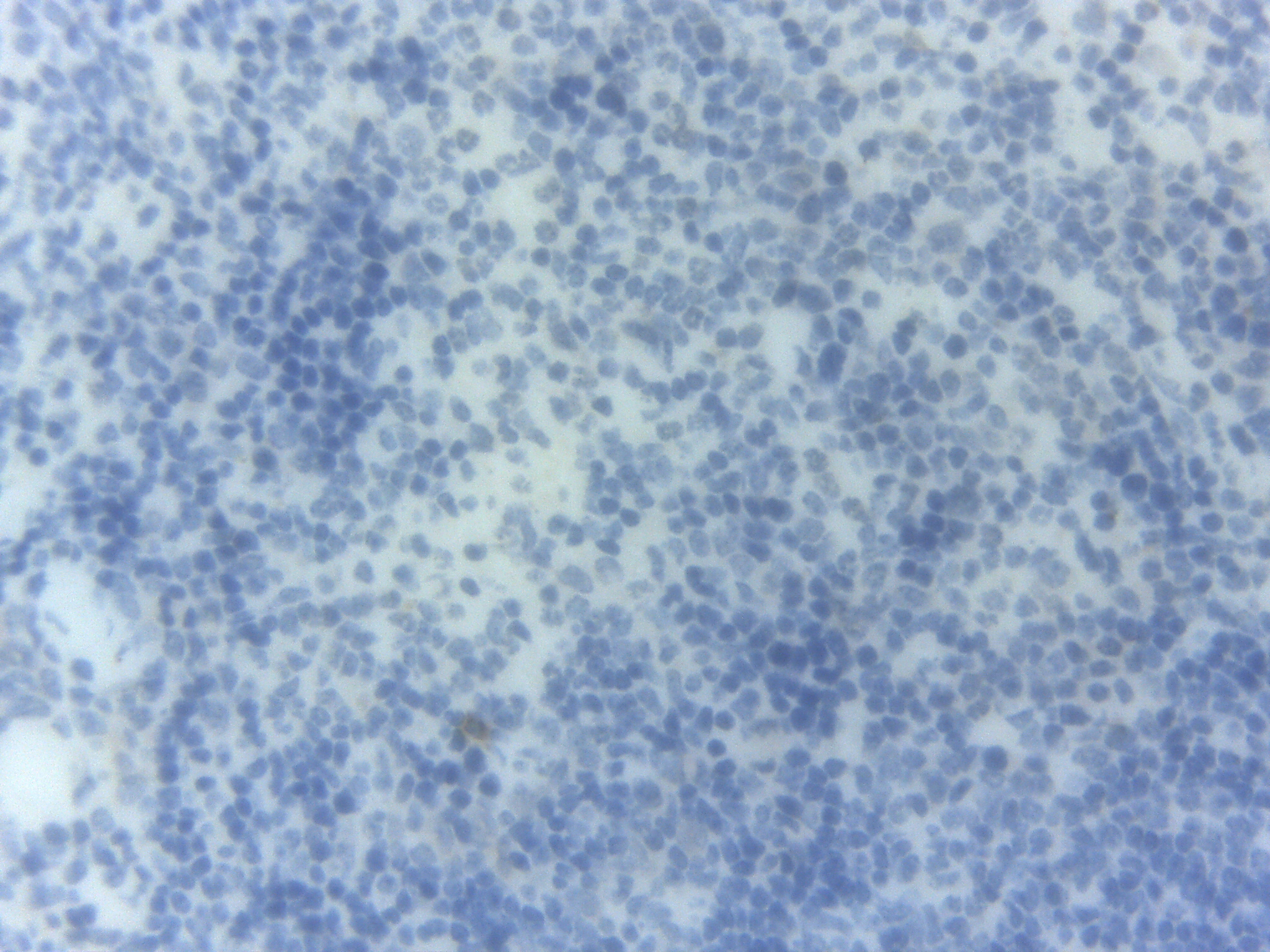

Supplement: S13 Fig — (ZIP) [file pone.0188960.s026.zip › Ly-6G IHC image CON/con-1-2.jpg]

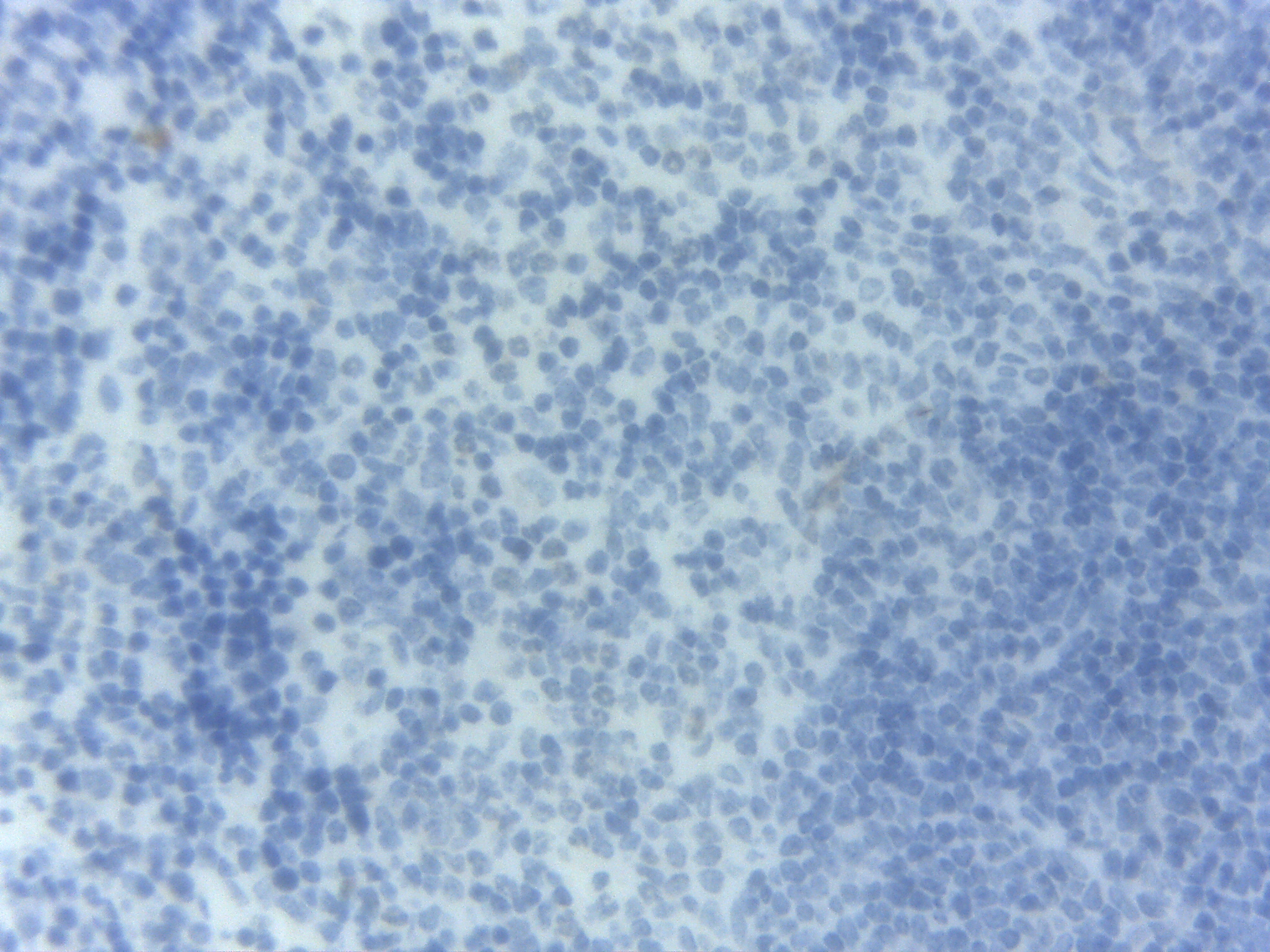

Supplement: S13 Fig — (ZIP) [file pone.0188960.s026.zip › Ly-6G IHC image CON/con-1-3.jpg]

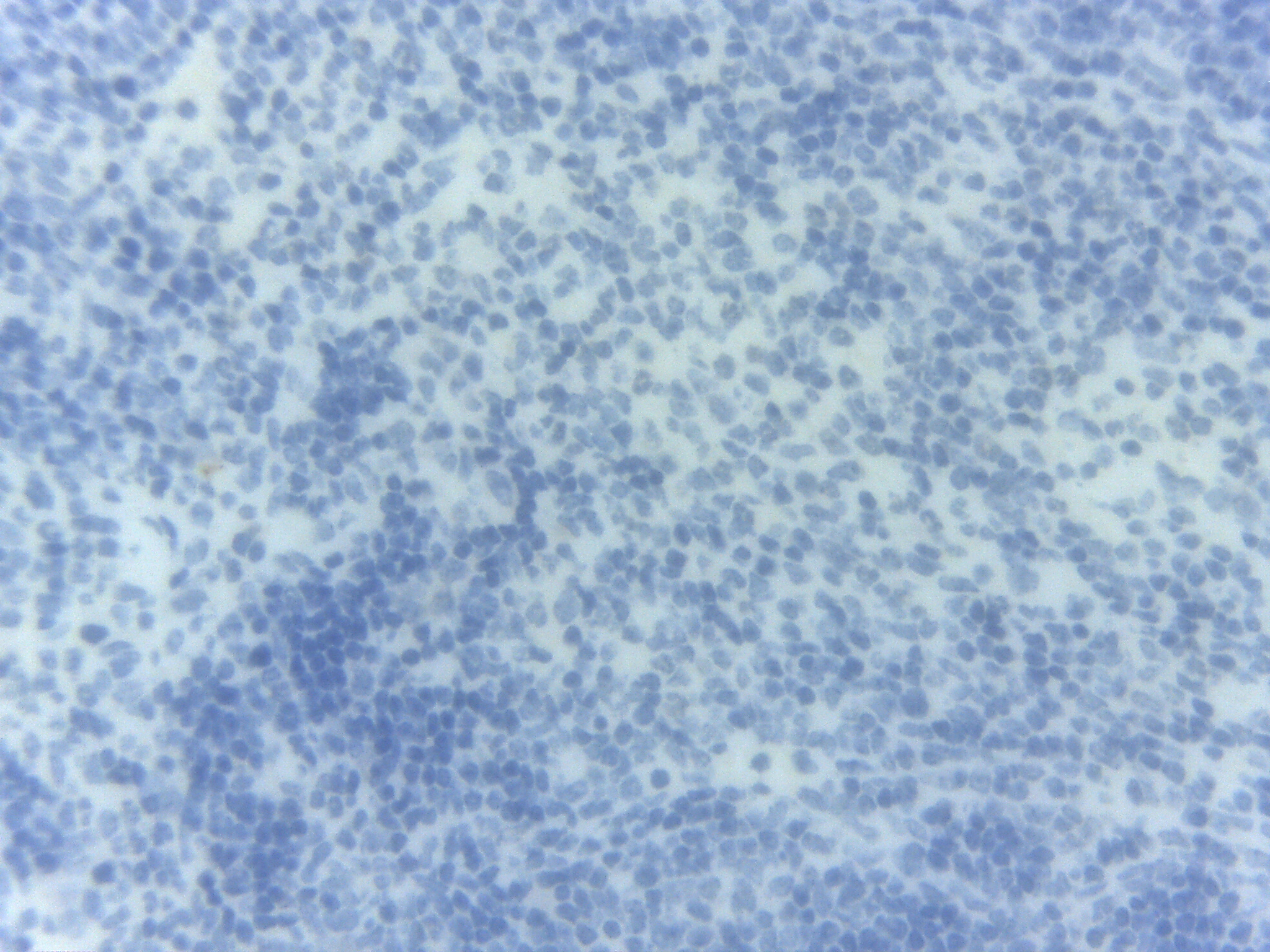

Supplement: S13 Fig — (ZIP) [file pone.0188960.s026.zip › Ly-6G IHC image CON/con-1-4.jpg]

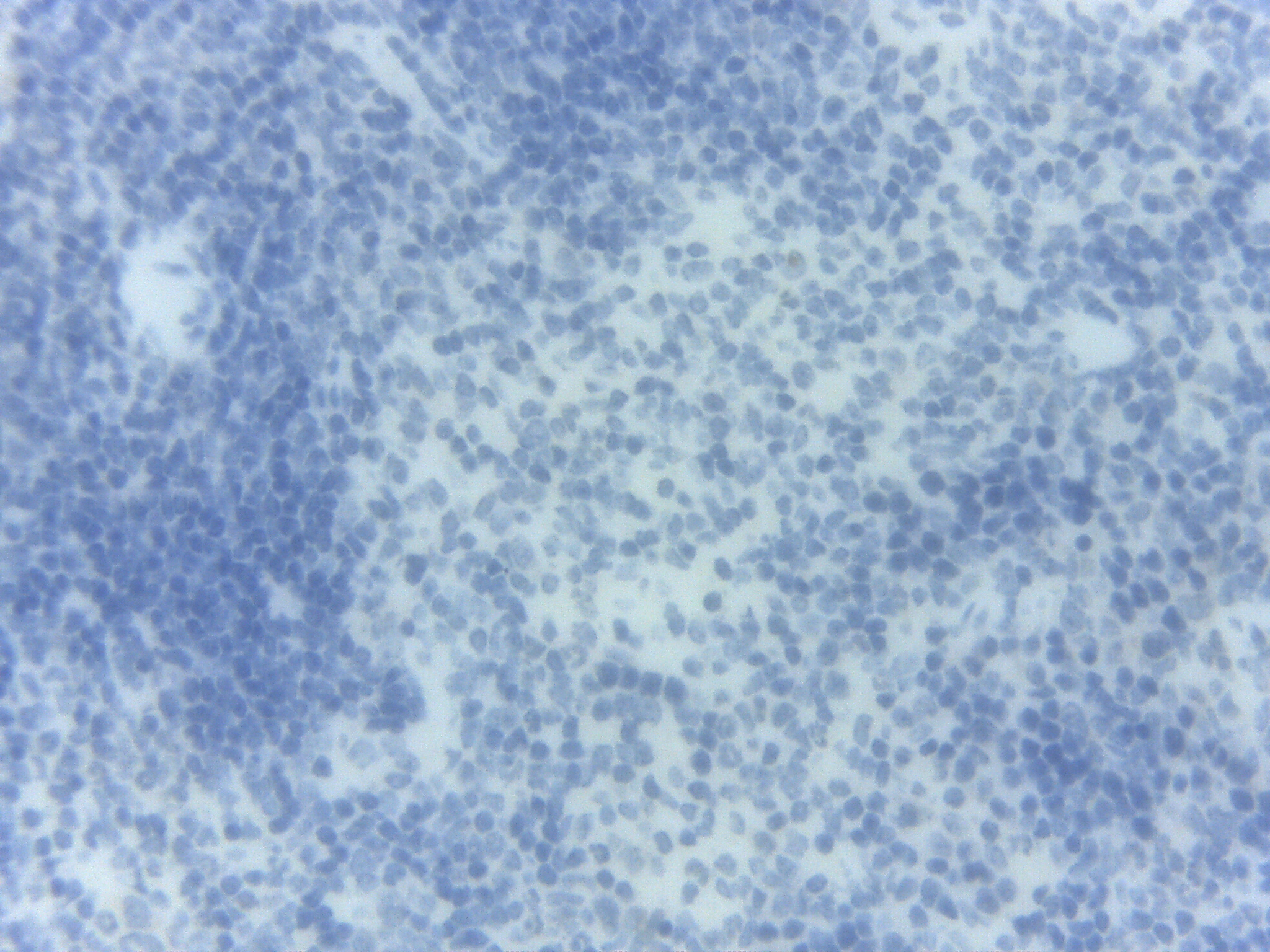

Supplement: S13 Fig — (ZIP) [file pone.0188960.s026.zip › Ly-6G IHC image CON/con-1-5.jpg]

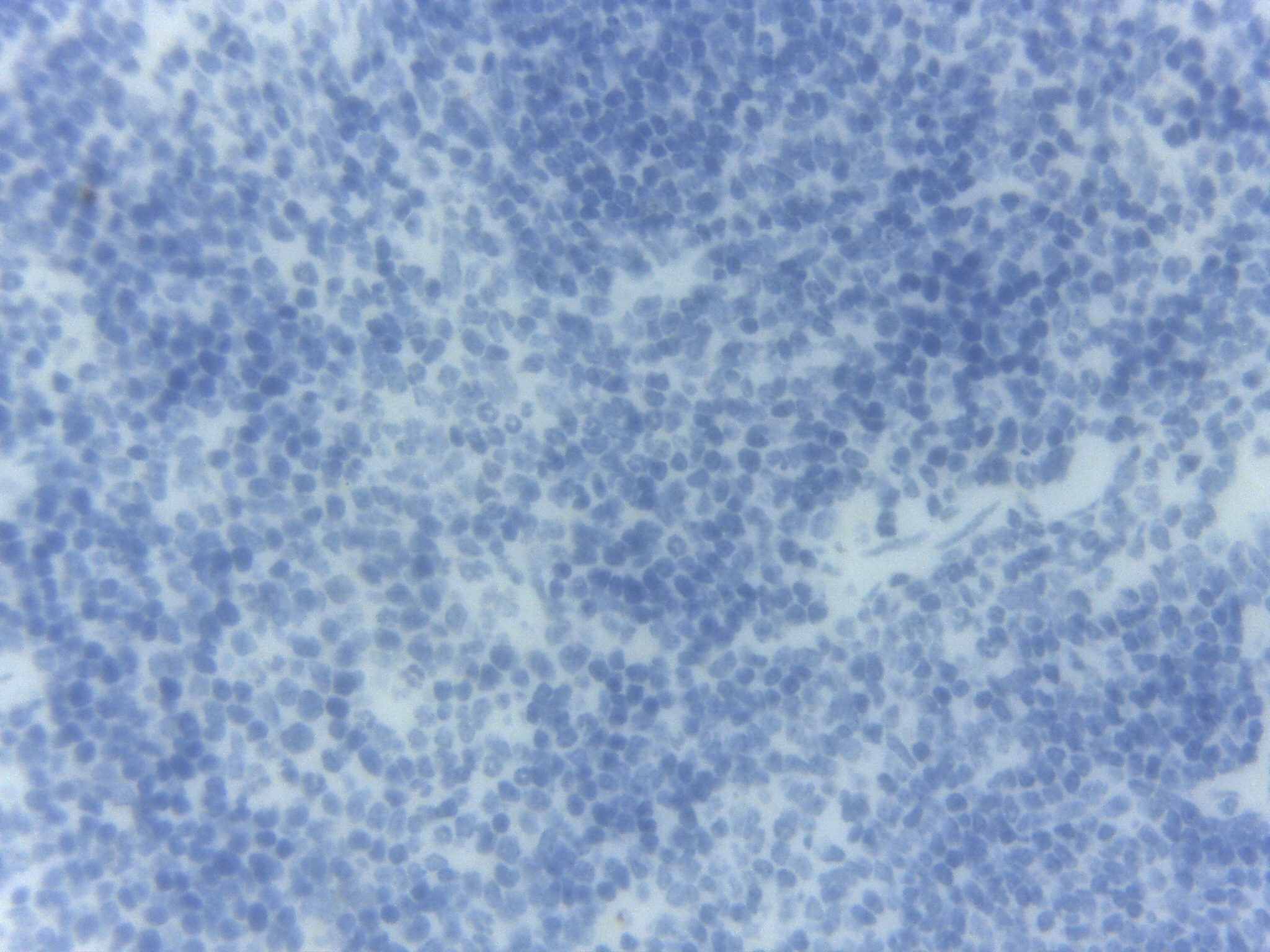

Supplement: S13 Fig — (ZIP) [file pone.0188960.s026.zip › Ly-6G IHC image CON/con-2-1.jpg]

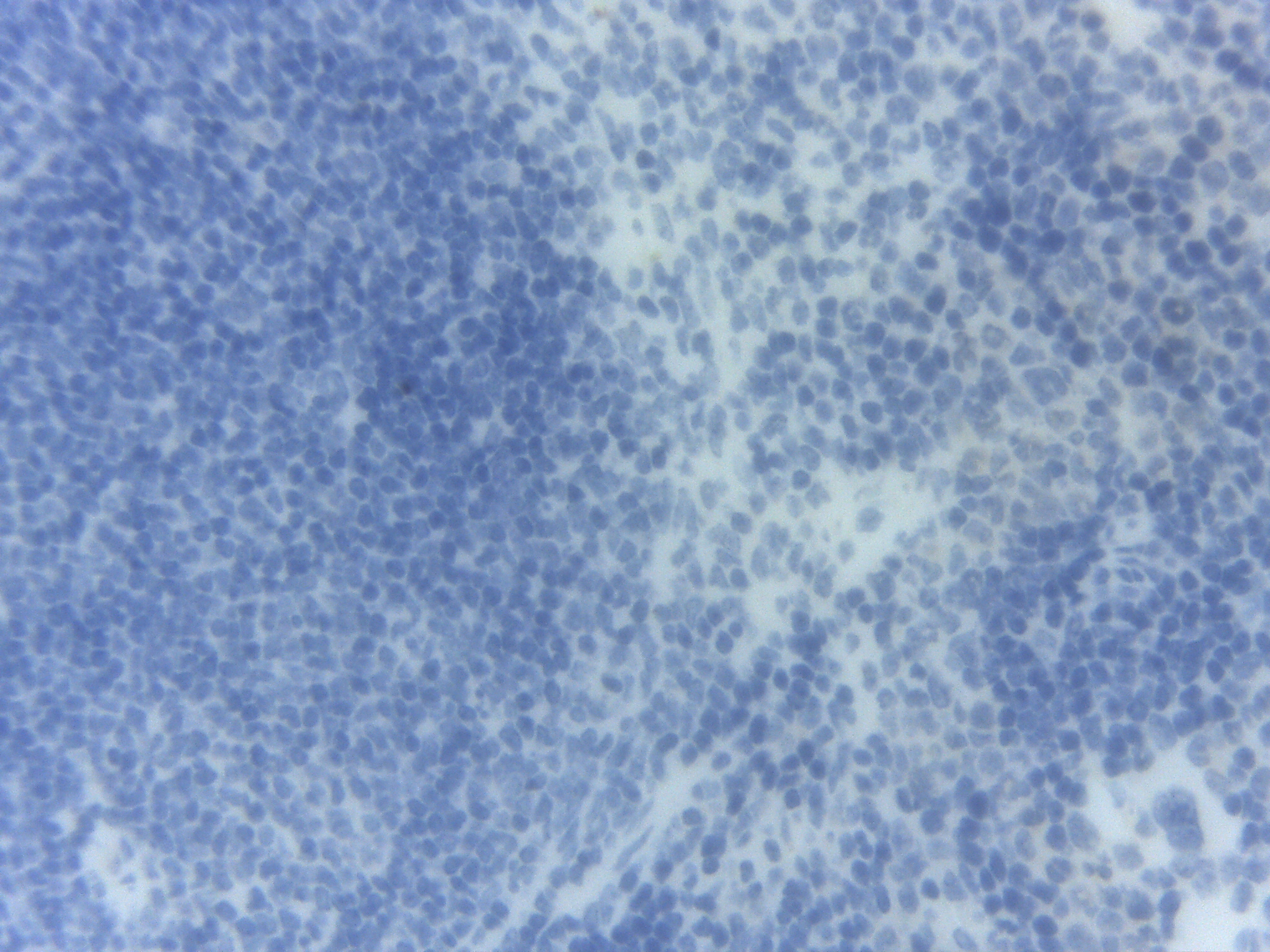

Supplement: S13 Fig — (ZIP) [file pone.0188960.s026.zip › Ly-6G IHC image CON/con-2-2.jpg]

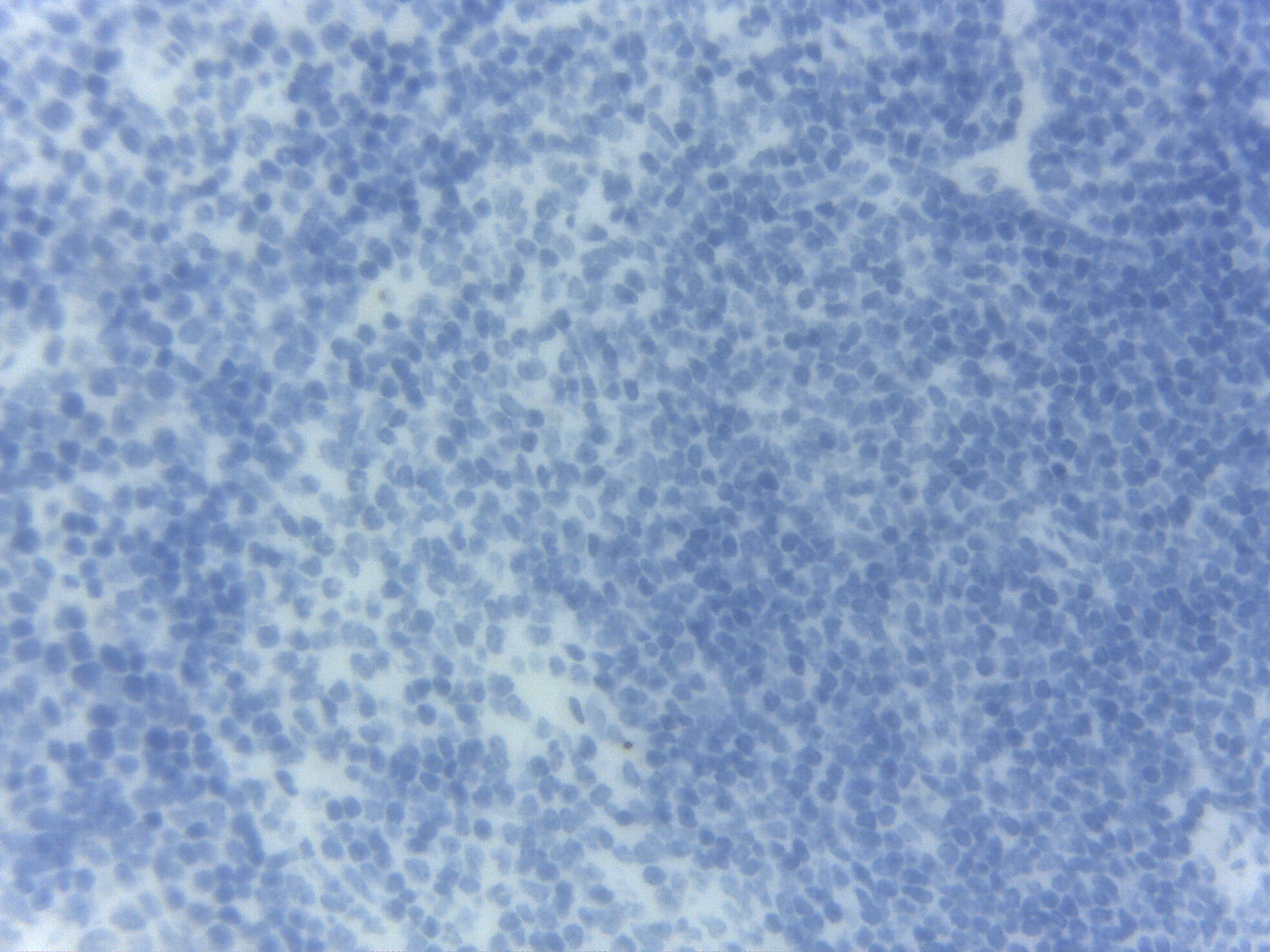

Supplement: S13 Fig — (ZIP) [file pone.0188960.s026.zip › Ly-6G IHC image CON/con-2-3.jpg]

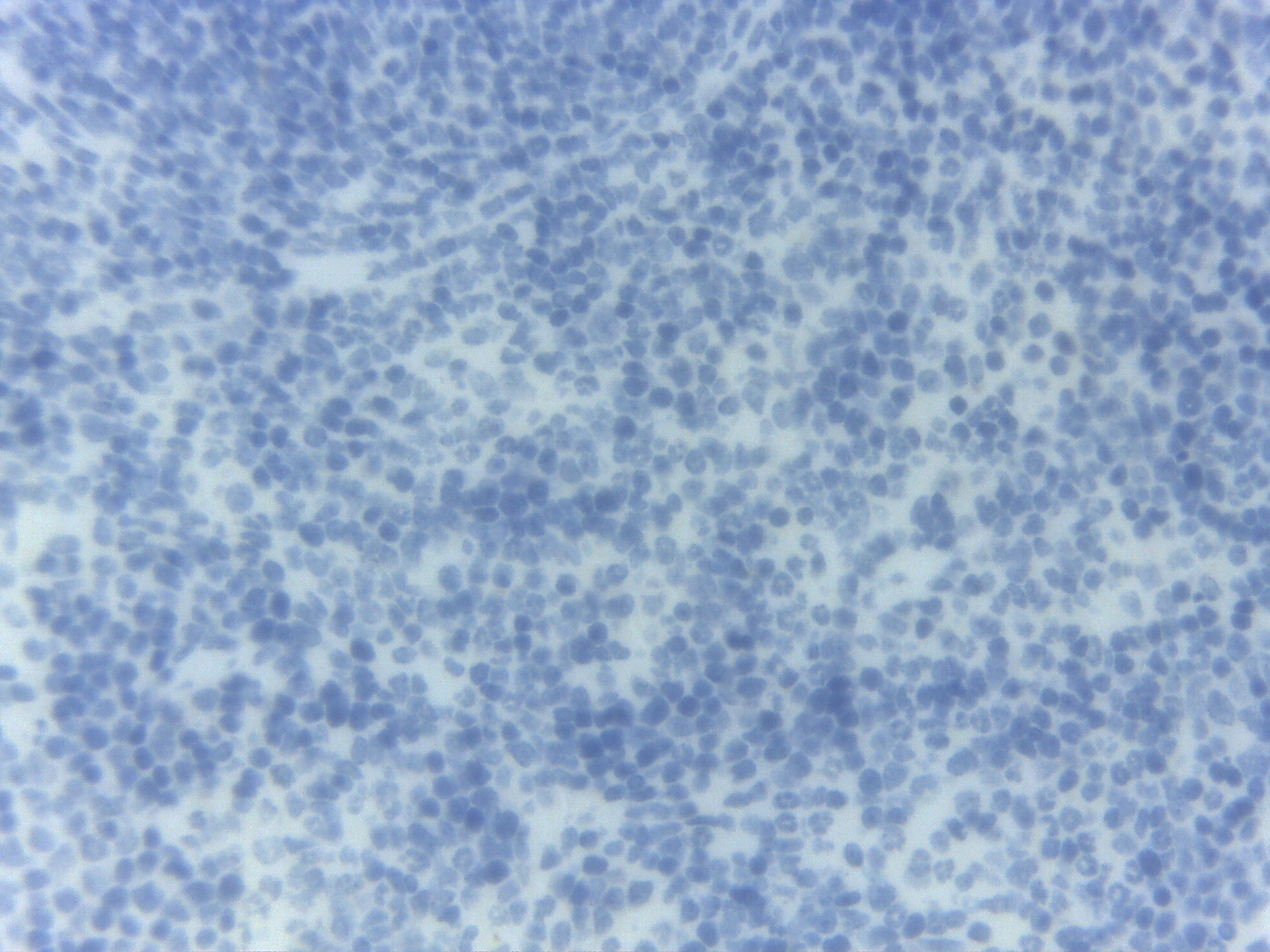

Supplement: S13 Fig — (ZIP) [file pone.0188960.s026.zip › Ly-6G IHC image CON/con-2-4.jpg]

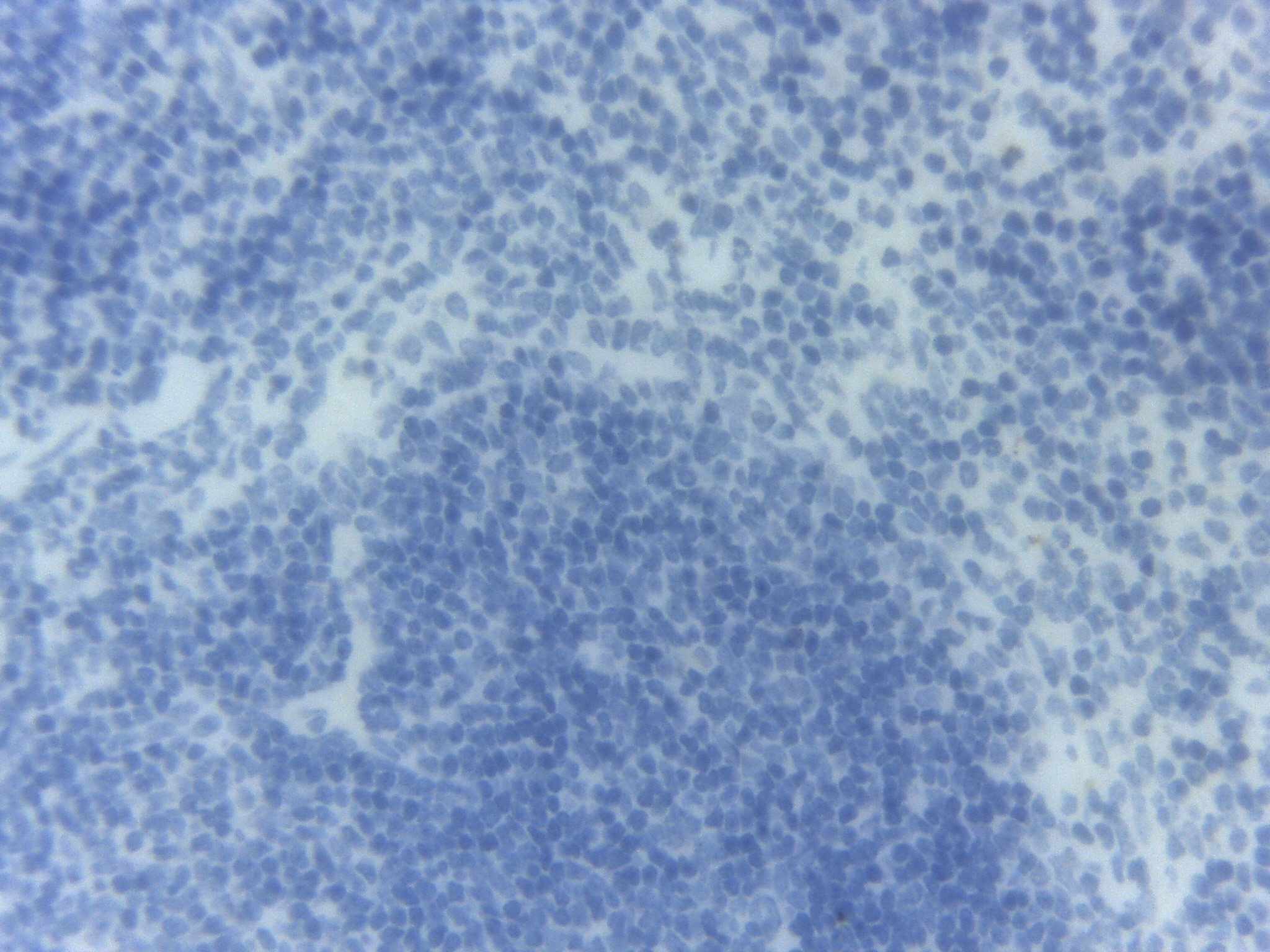

Supplement: S13 Fig — (ZIP) [file pone.0188960.s026.zip › Ly-6G IHC image CON/con-2-5.jpg]

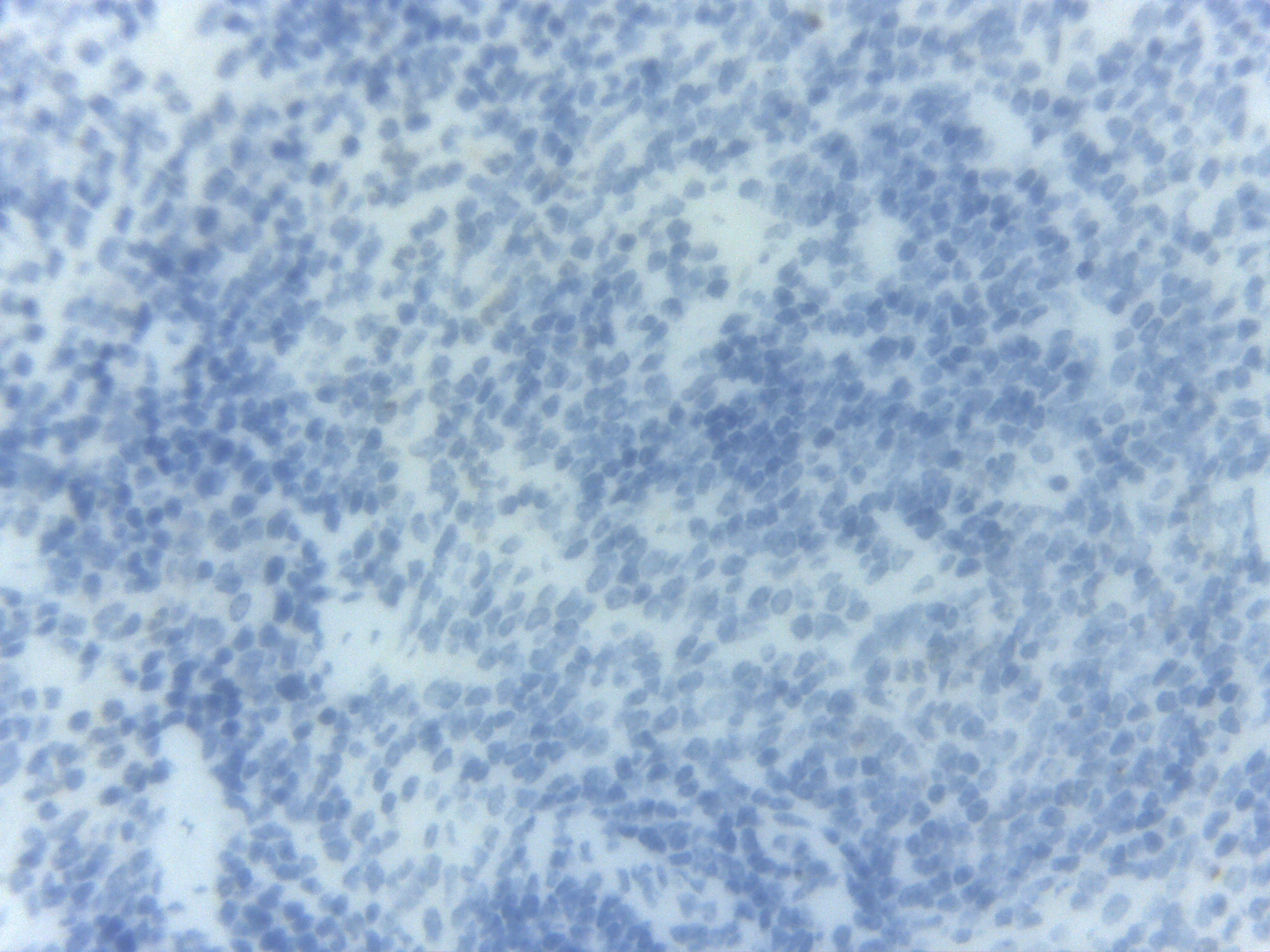

Supplement: S13 Fig — (ZIP) [file pone.0188960.s026.zip › Ly-6G IHC image CON/con-3-1.jpg]

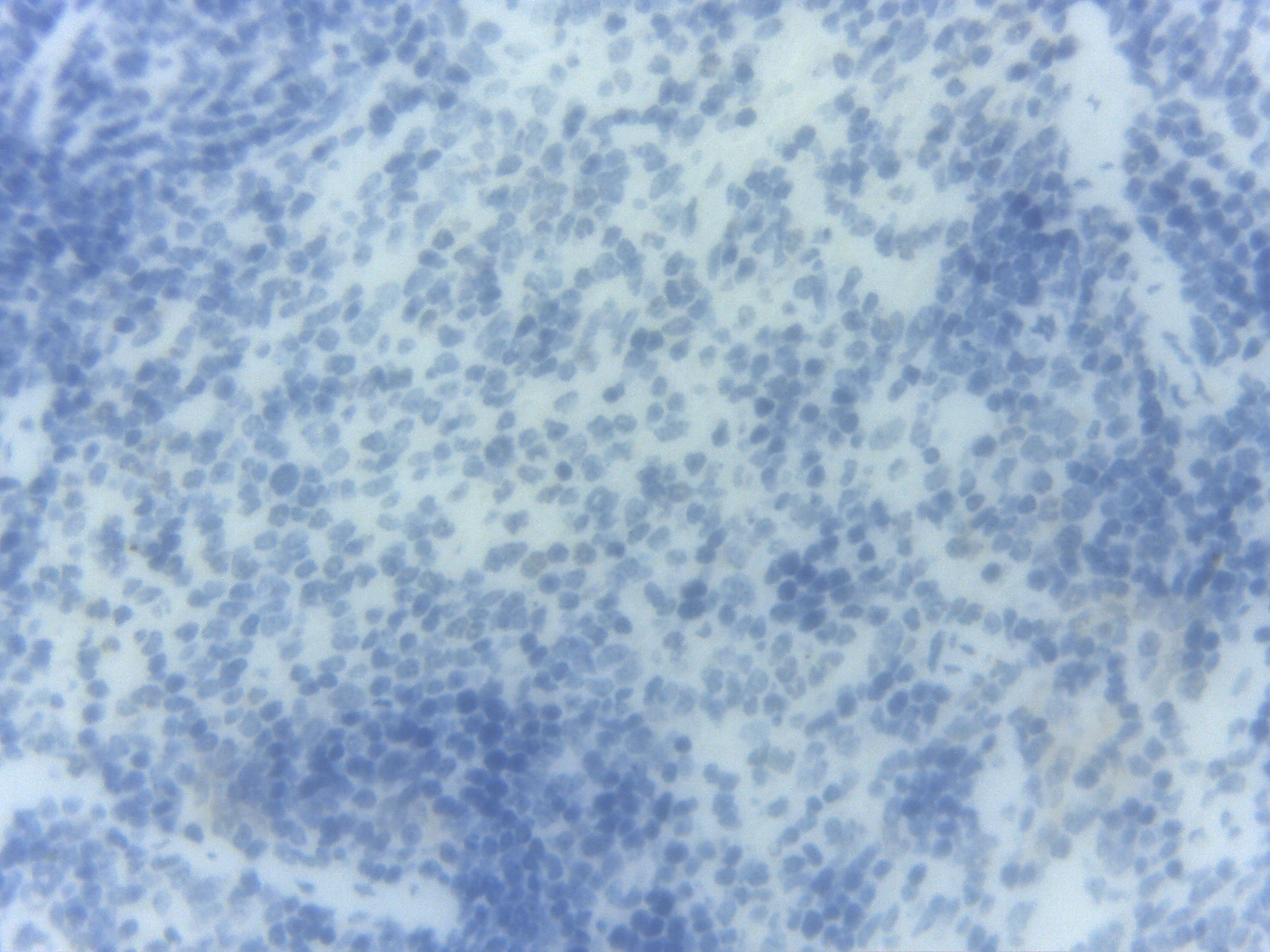

Supplement: S13 Fig — (ZIP) [file pone.0188960.s026.zip › Ly-6G IHC image CON/con-3-2.jpg]

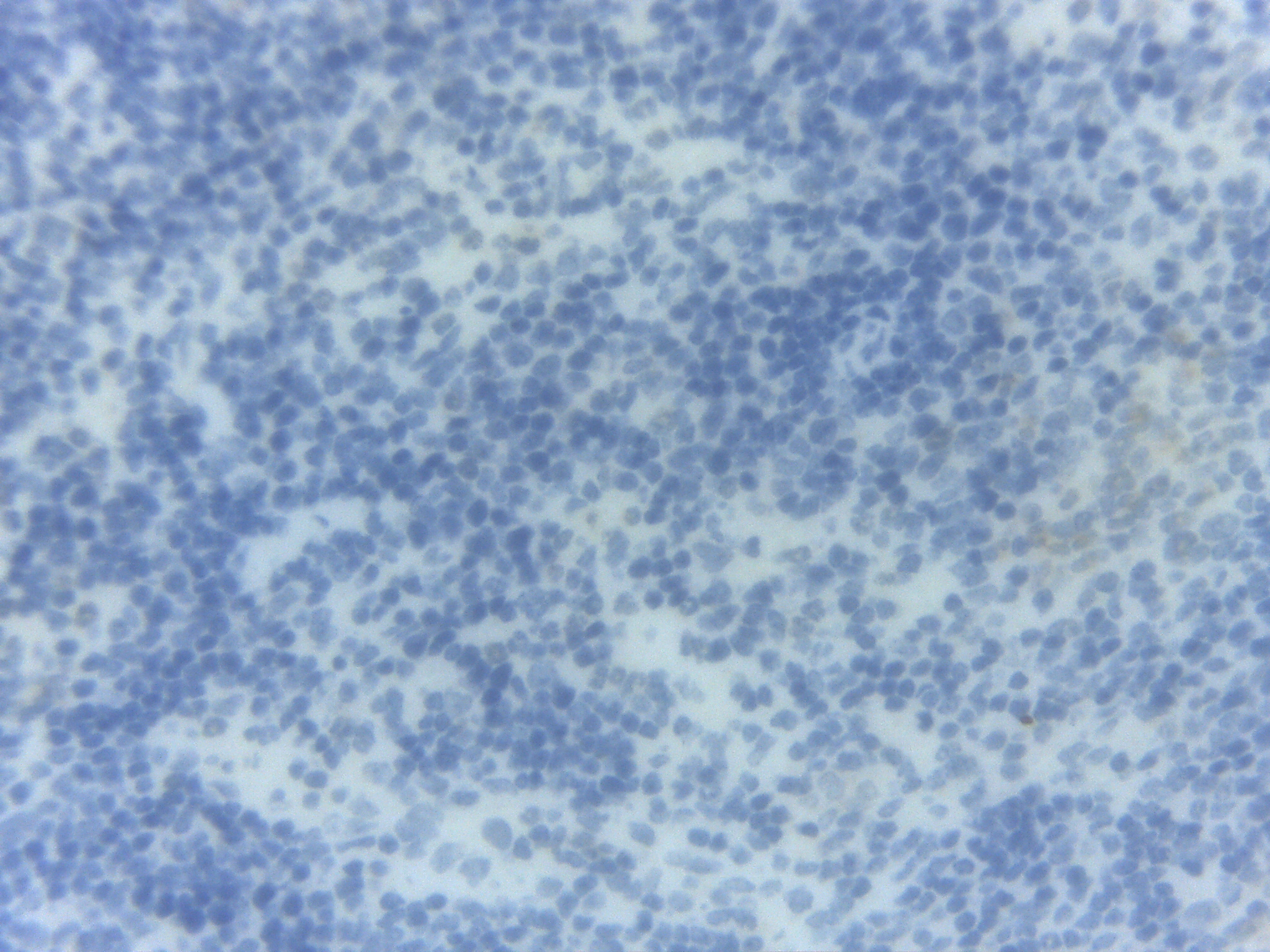

Supplement: S13 Fig — (ZIP) [file pone.0188960.s026.zip › Ly-6G IHC image CON/con-3-3.jpg]

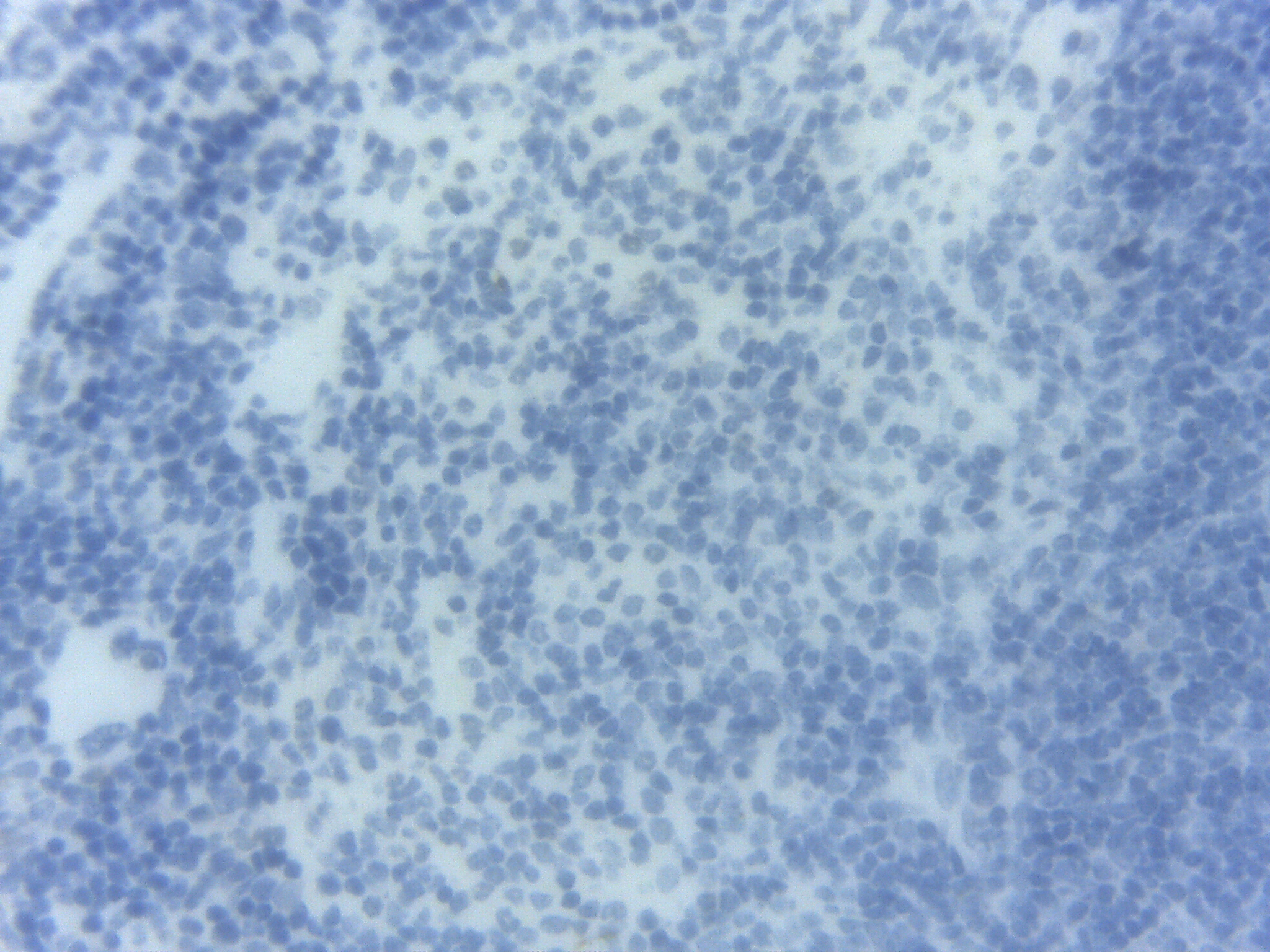

Supplement: S13 Fig — (ZIP) [file pone.0188960.s026.zip › Ly-6G IHC image CON/con-3-4.jpg]

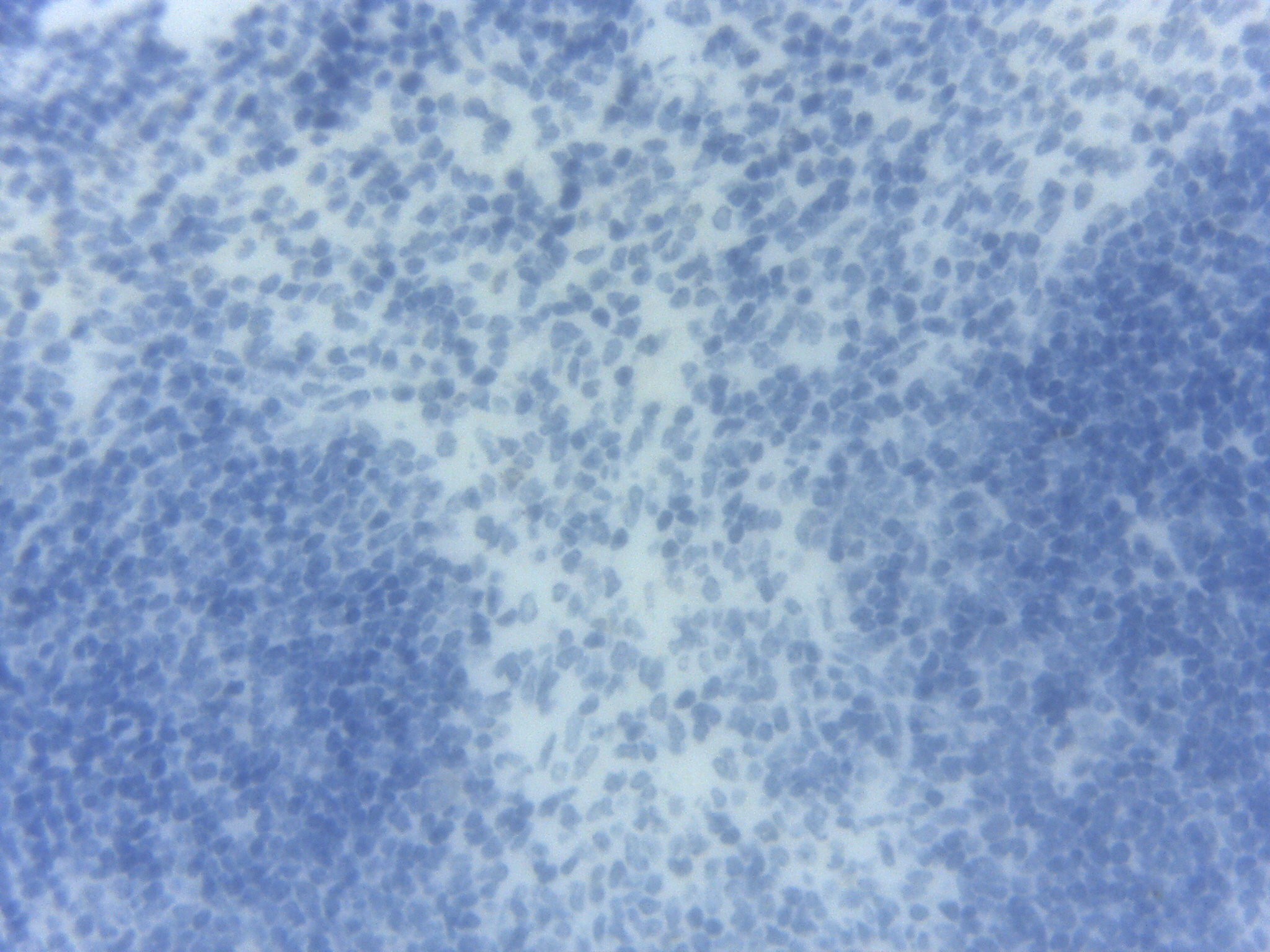

Supplement: S13 Fig — (ZIP) [file pone.0188960.s026.zip › Ly-6G IHC image CON/con-3-5.jpg]

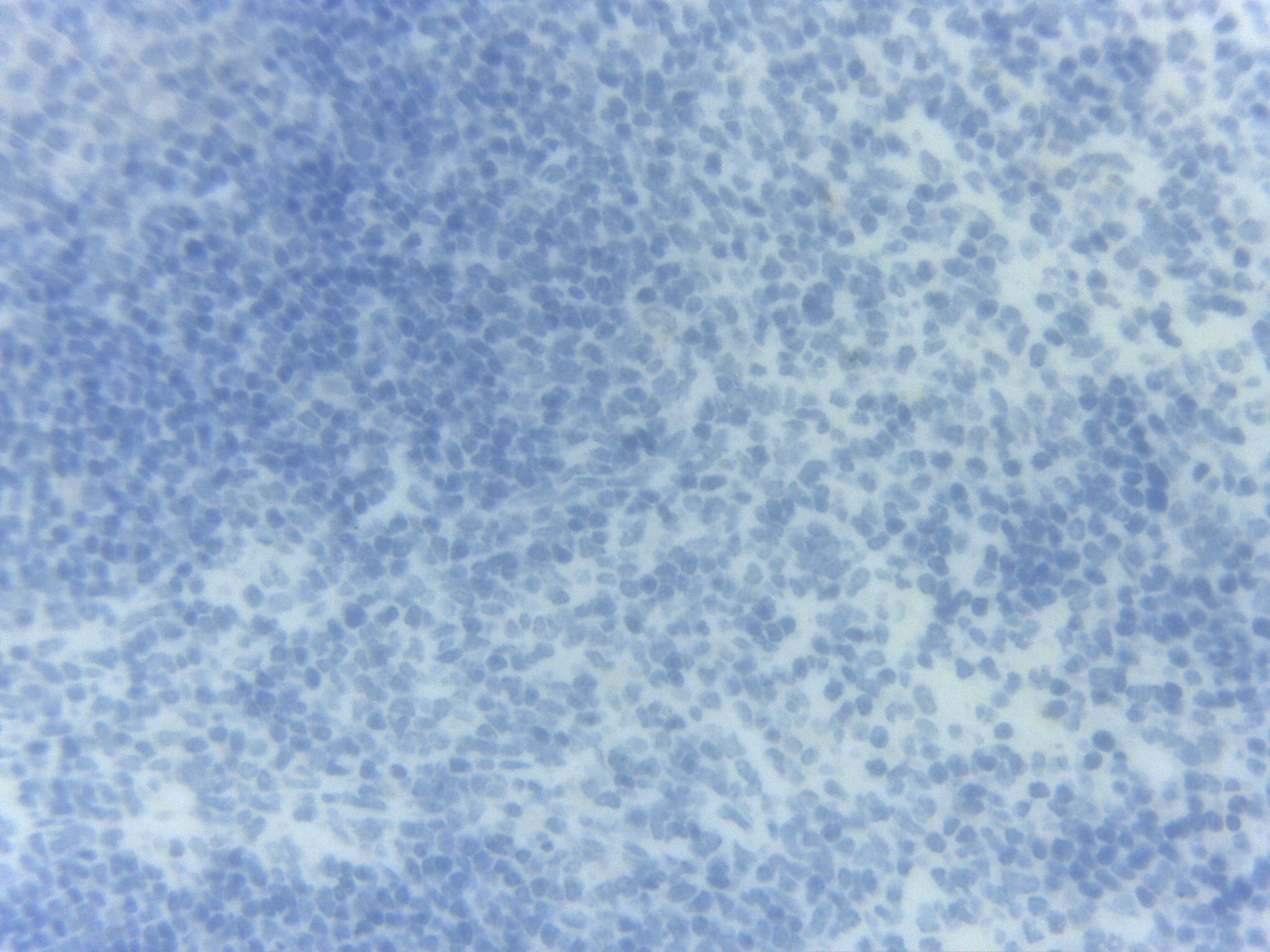

Supplement: S13 Fig — (ZIP) [file pone.0188960.s026.zip › Ly-6G IHC image CON/con-4-1.jpg]

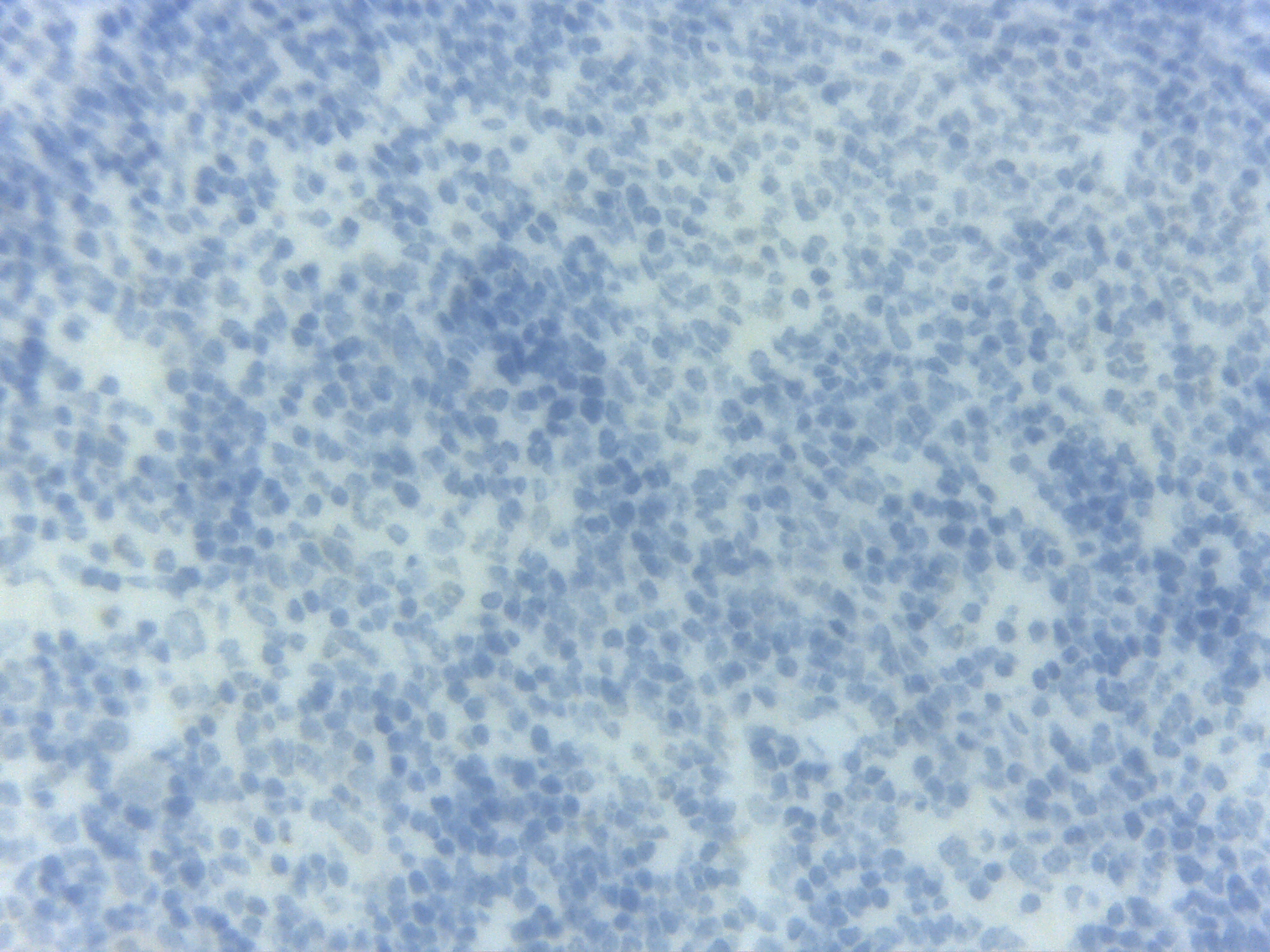

Supplement: S13 Fig — (ZIP) [file pone.0188960.s026.zip › Ly-6G IHC image CON/con-4-2.jpg]

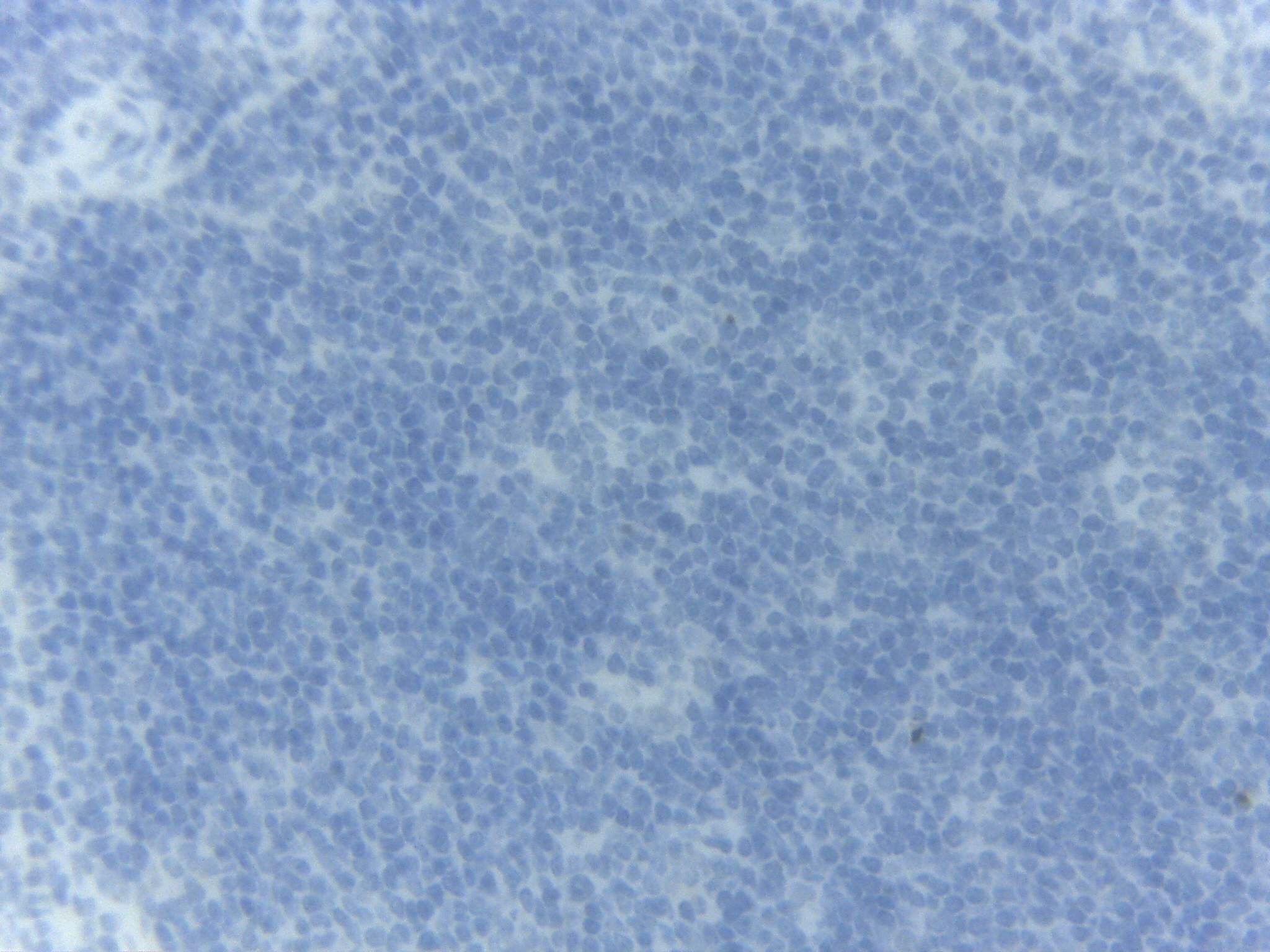

Supplement: S13 Fig — (ZIP) [file pone.0188960.s026.zip › Ly-6G IHC image CON/con-4-3.jpg]

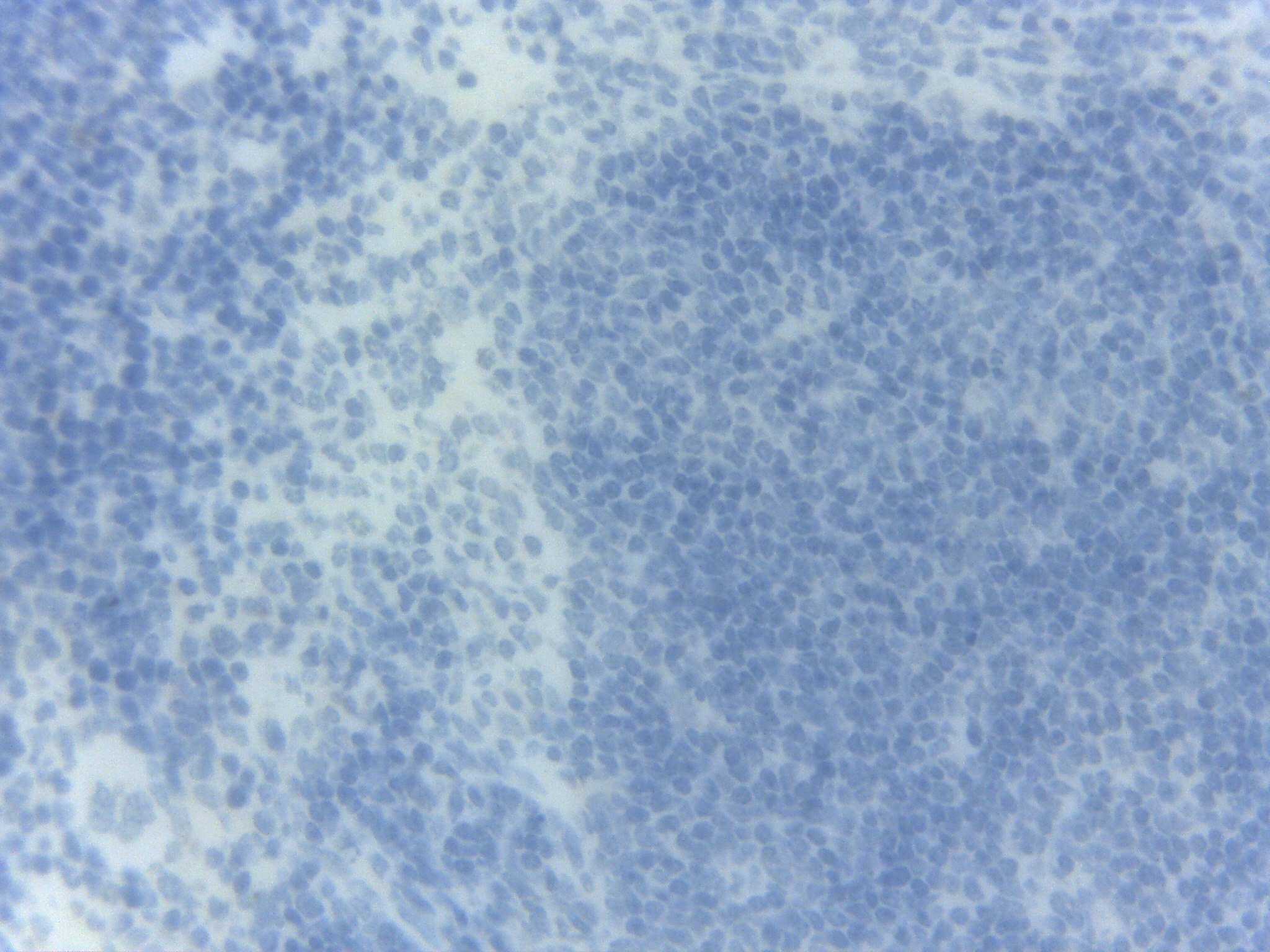

Supplement: S13 Fig — (ZIP) [file pone.0188960.s026.zip › Ly-6G IHC image CON/con-4-4.jpg]

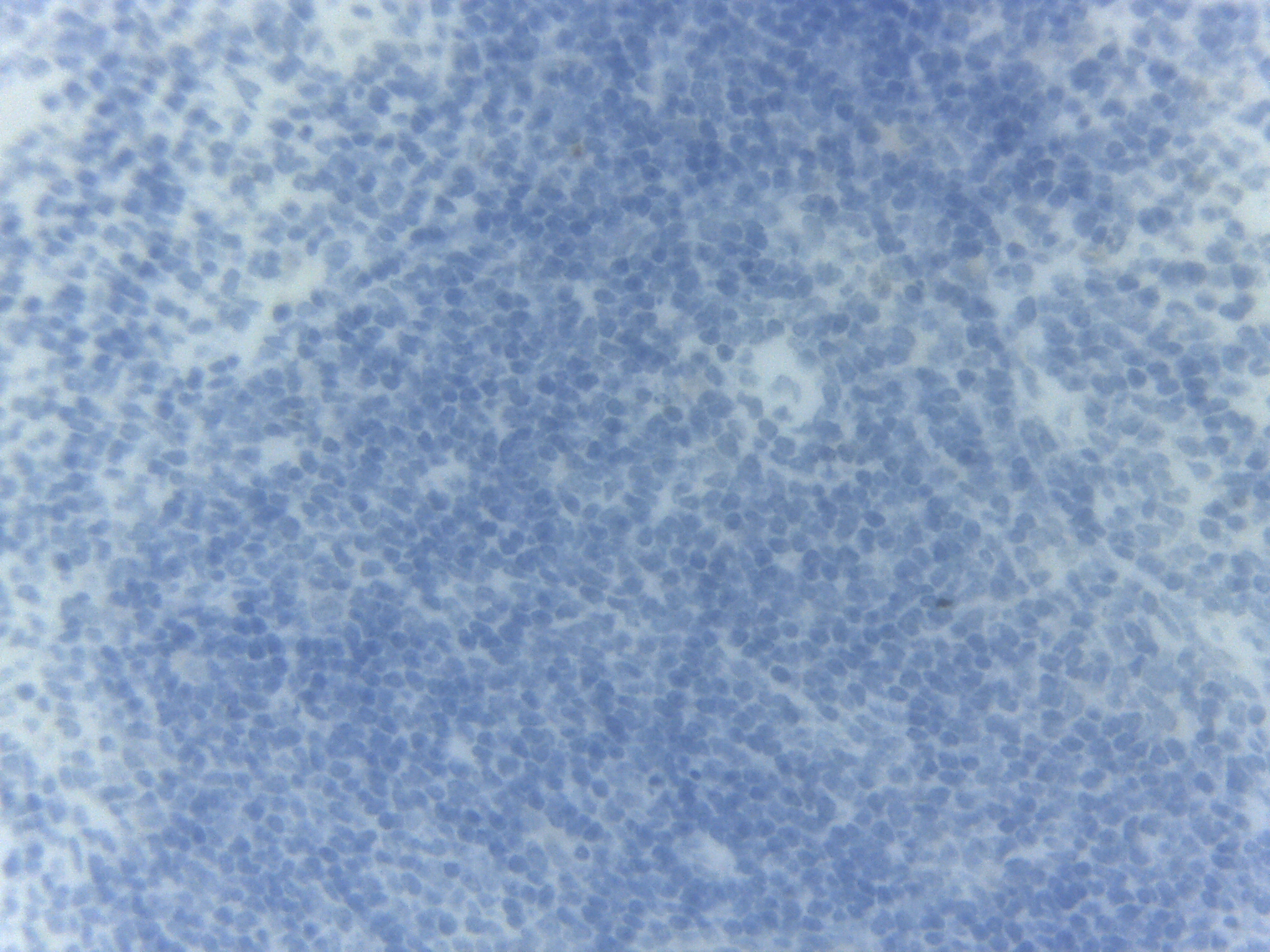

Supplement: S13 Fig — (ZIP) [file pone.0188960.s026.zip › Ly-6G IHC image CON/con-4-5.jpg]

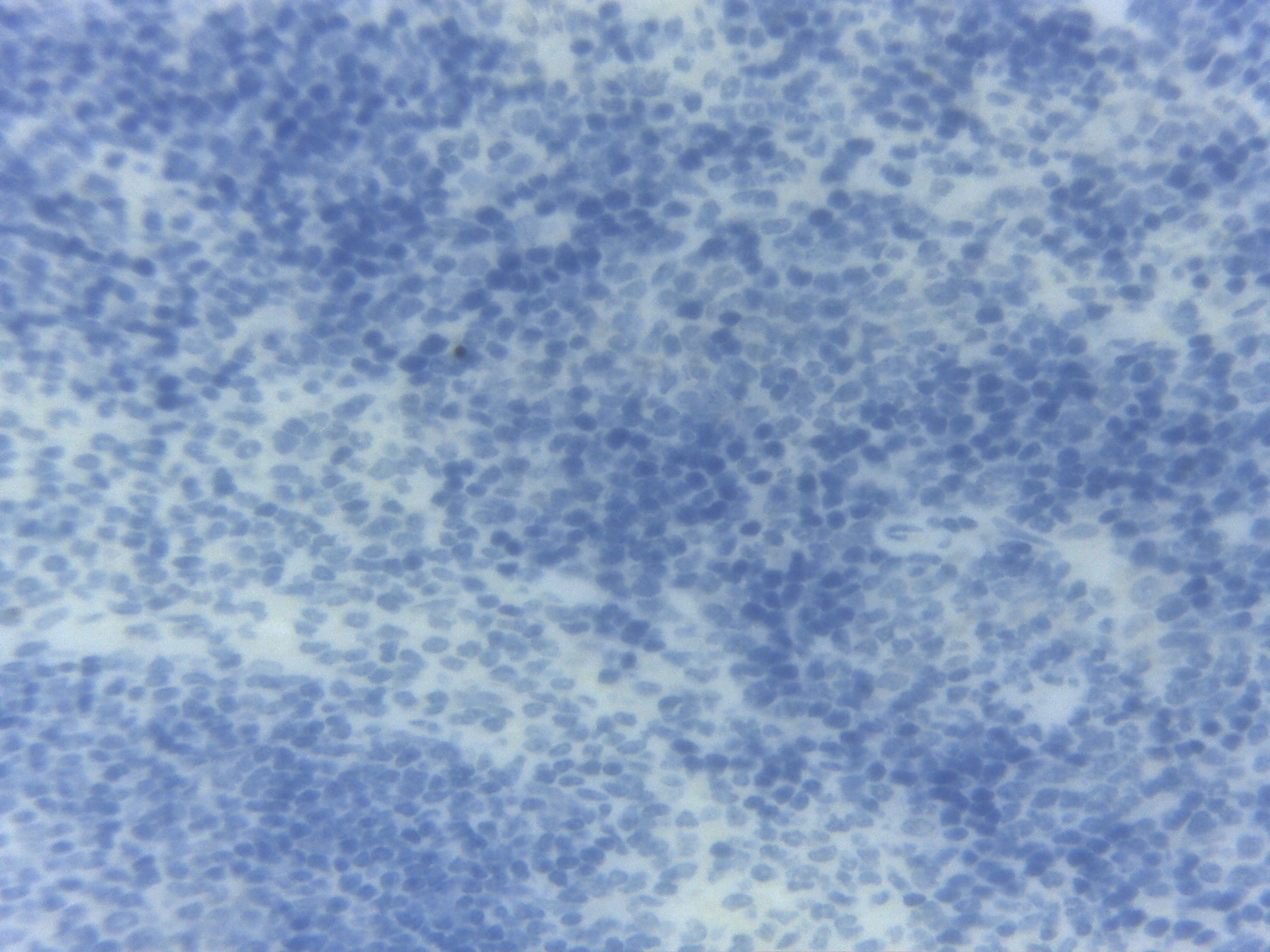

Supplement: S13 Fig — (ZIP) [file pone.0188960.s026.zip › Ly-6G IHC image CON/con-6-1.jpg]

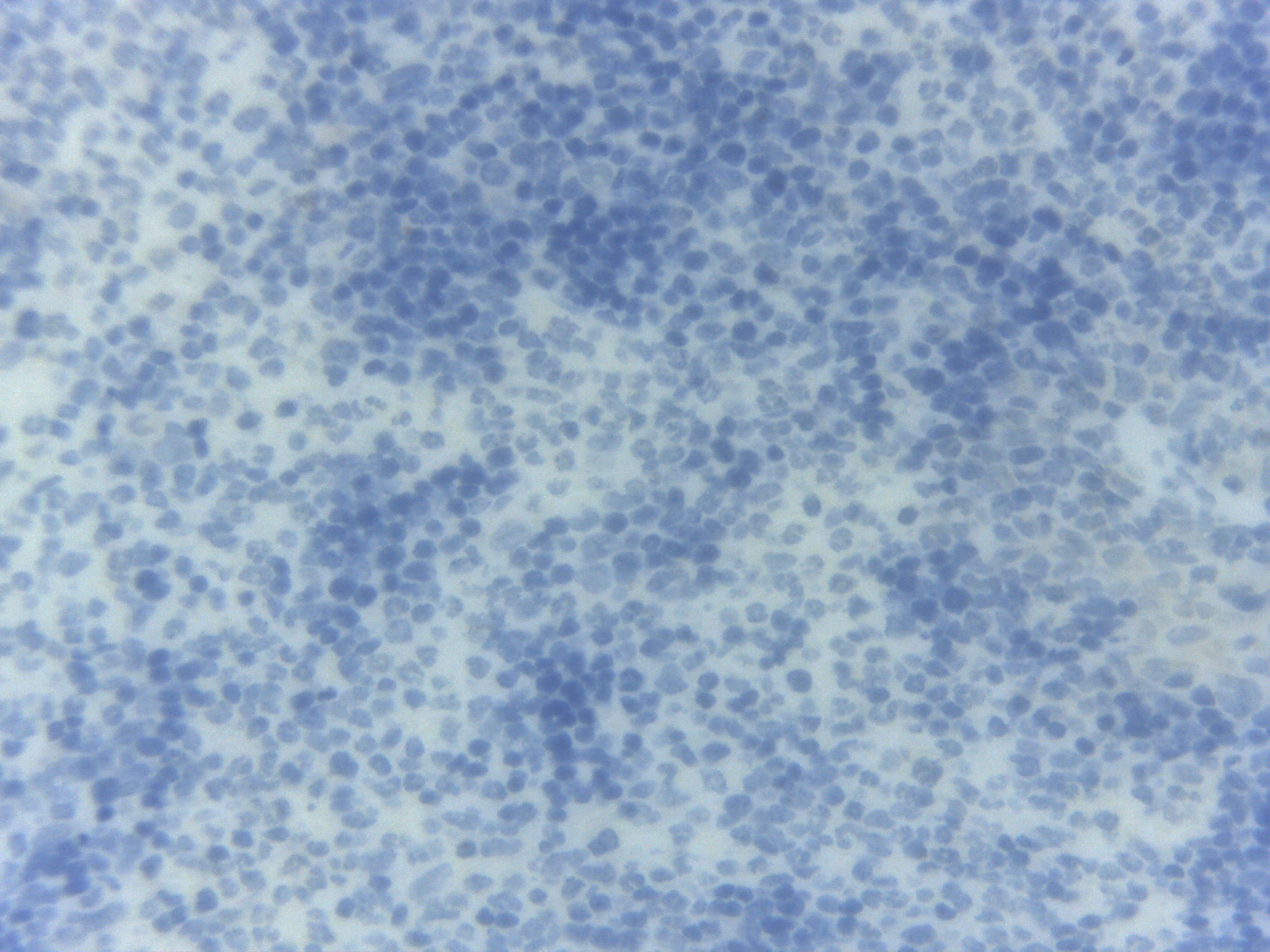

Supplement: S13 Fig — (ZIP) [file pone.0188960.s026.zip › Ly-6G IHC image CON/con-6-2.jpg]

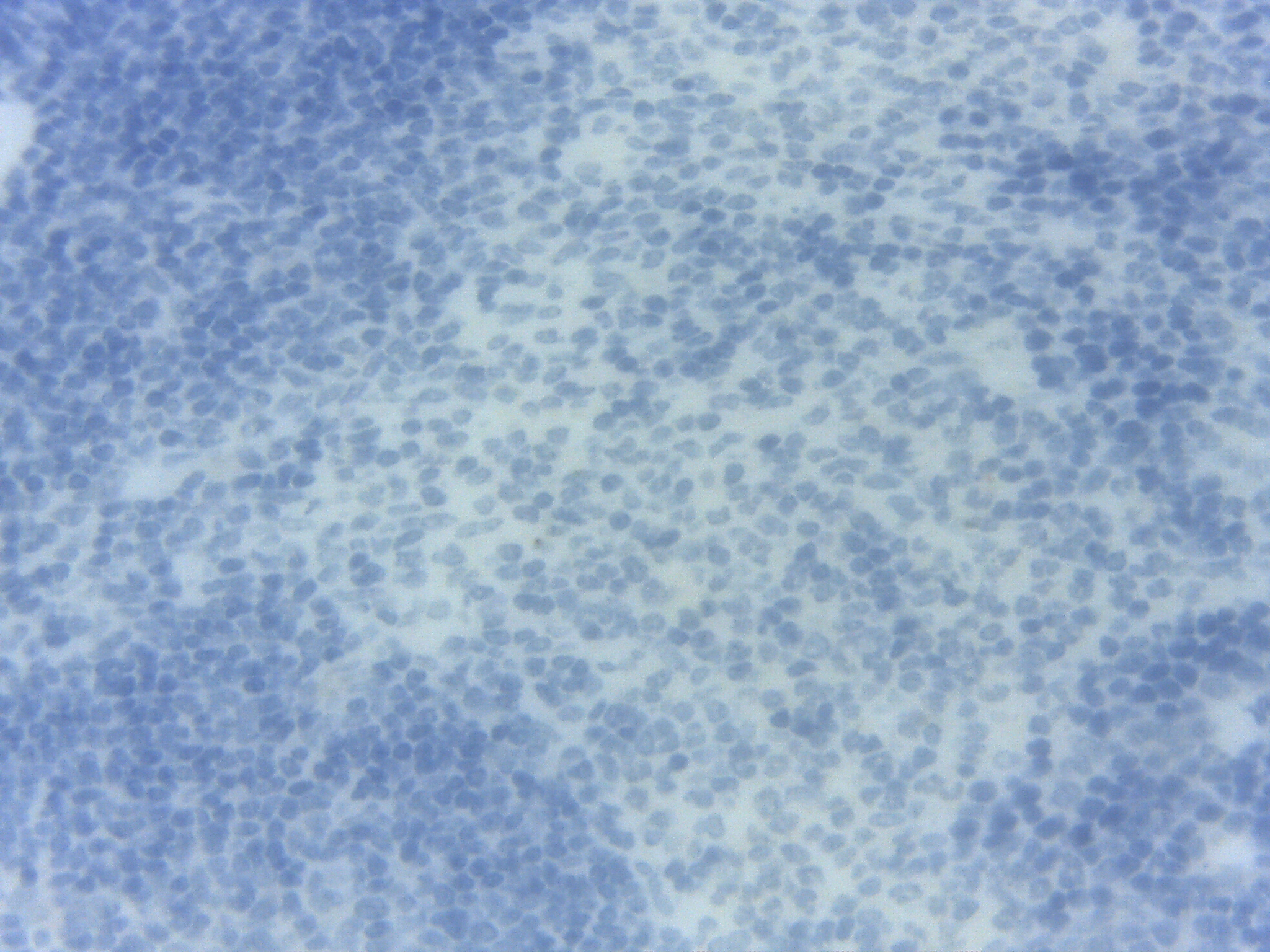

Supplement: S13 Fig — (ZIP) [file pone.0188960.s026.zip › Ly-6G IHC image CON/con-6-3.jpg]

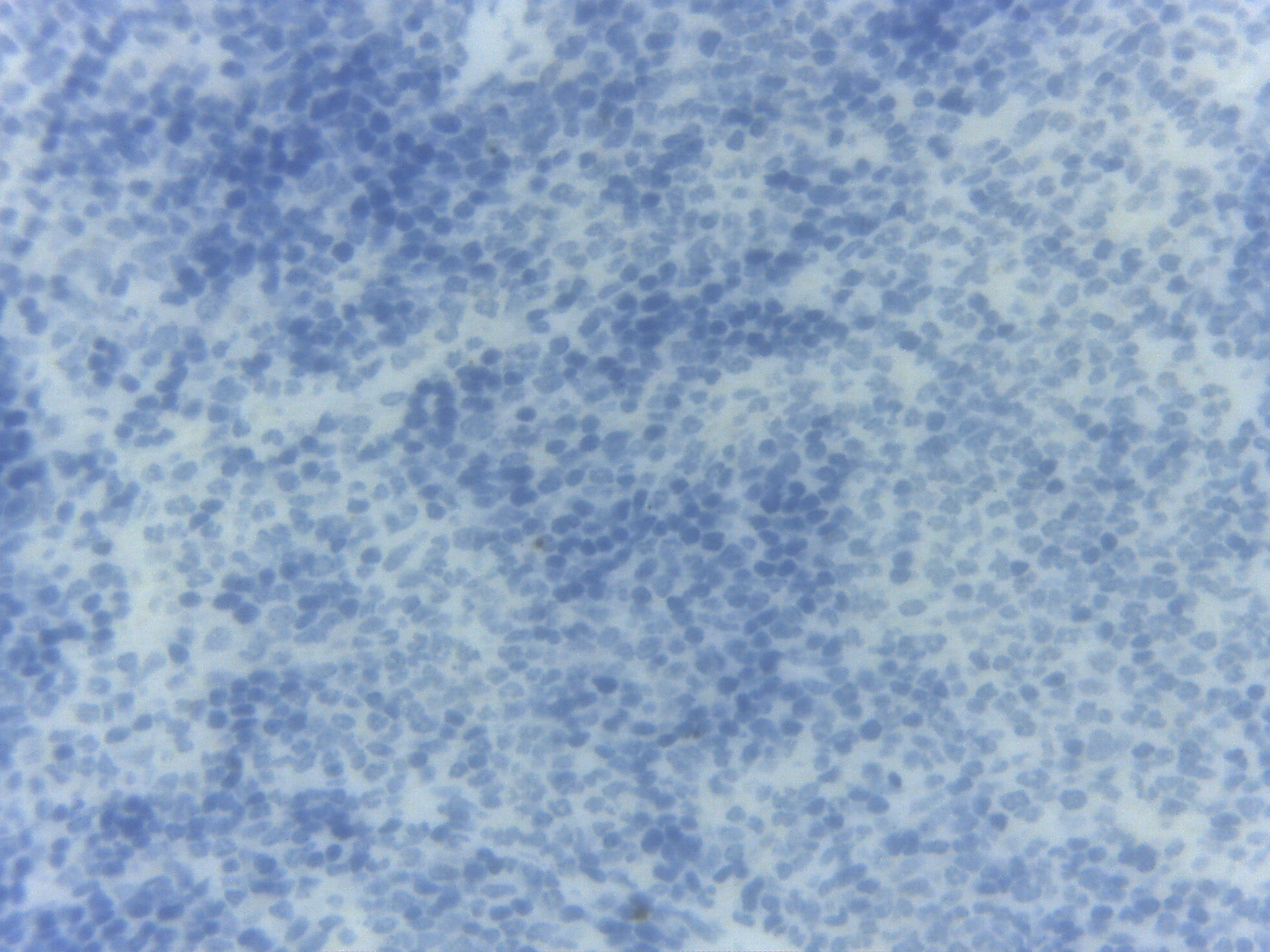

Supplement: S13 Fig — (ZIP) [file pone.0188960.s026.zip › Ly-6G IHC image CON/con-6-4.jpg]

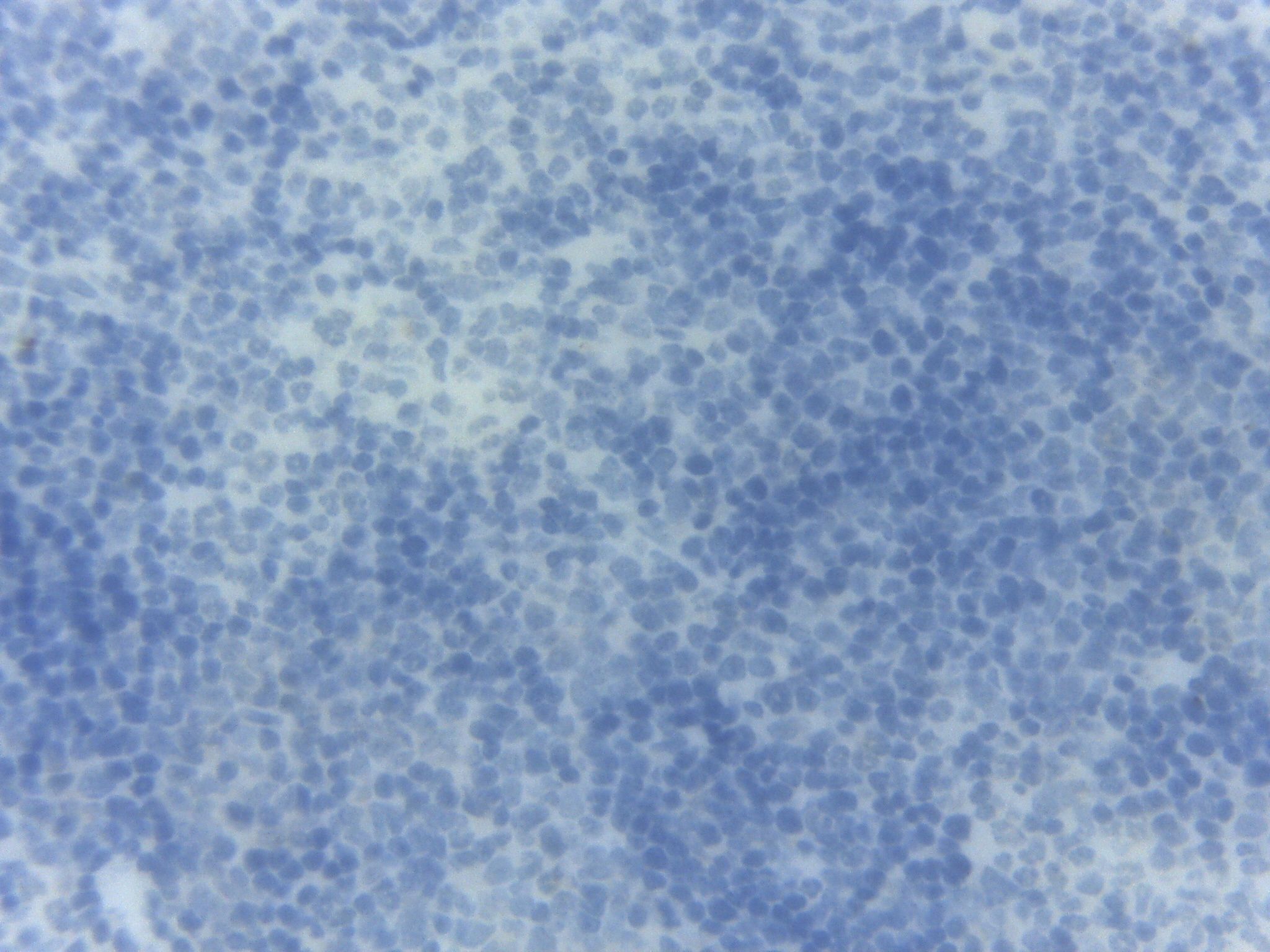

Supplement: S13 Fig — (ZIP) [file pone.0188960.s026.zip › Ly-6G IHC image CON/con-6-5.jpg]

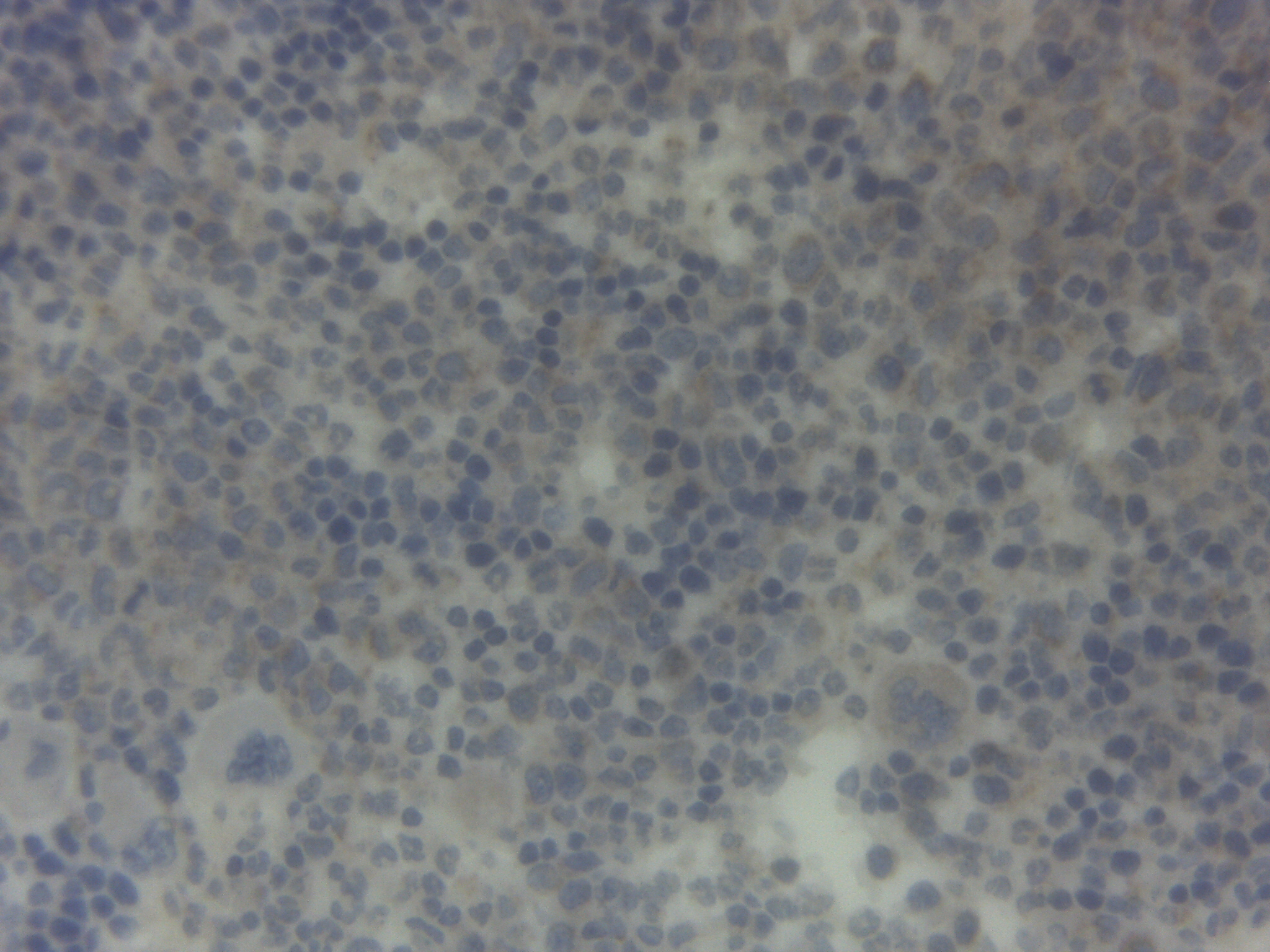

Supplement: S14 Fig — (ZIP) [file pone.0188960.s027.zip › NKp46 IHC image 24 hours/24h-1-1.jpg]

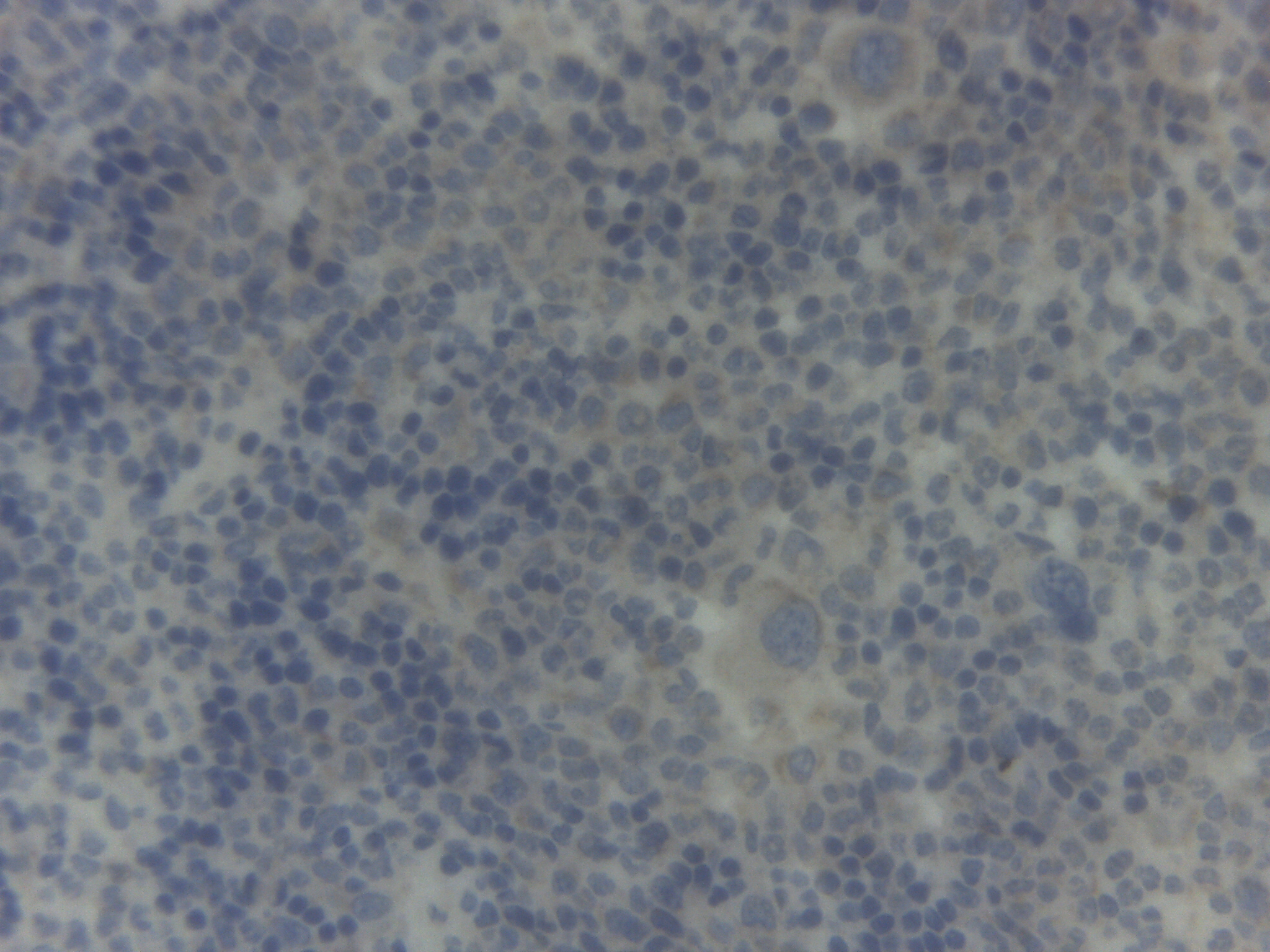

Supplement: S14 Fig — (ZIP) [file pone.0188960.s027.zip › NKp46 IHC image 24 hours/24h-1-2.jpg]

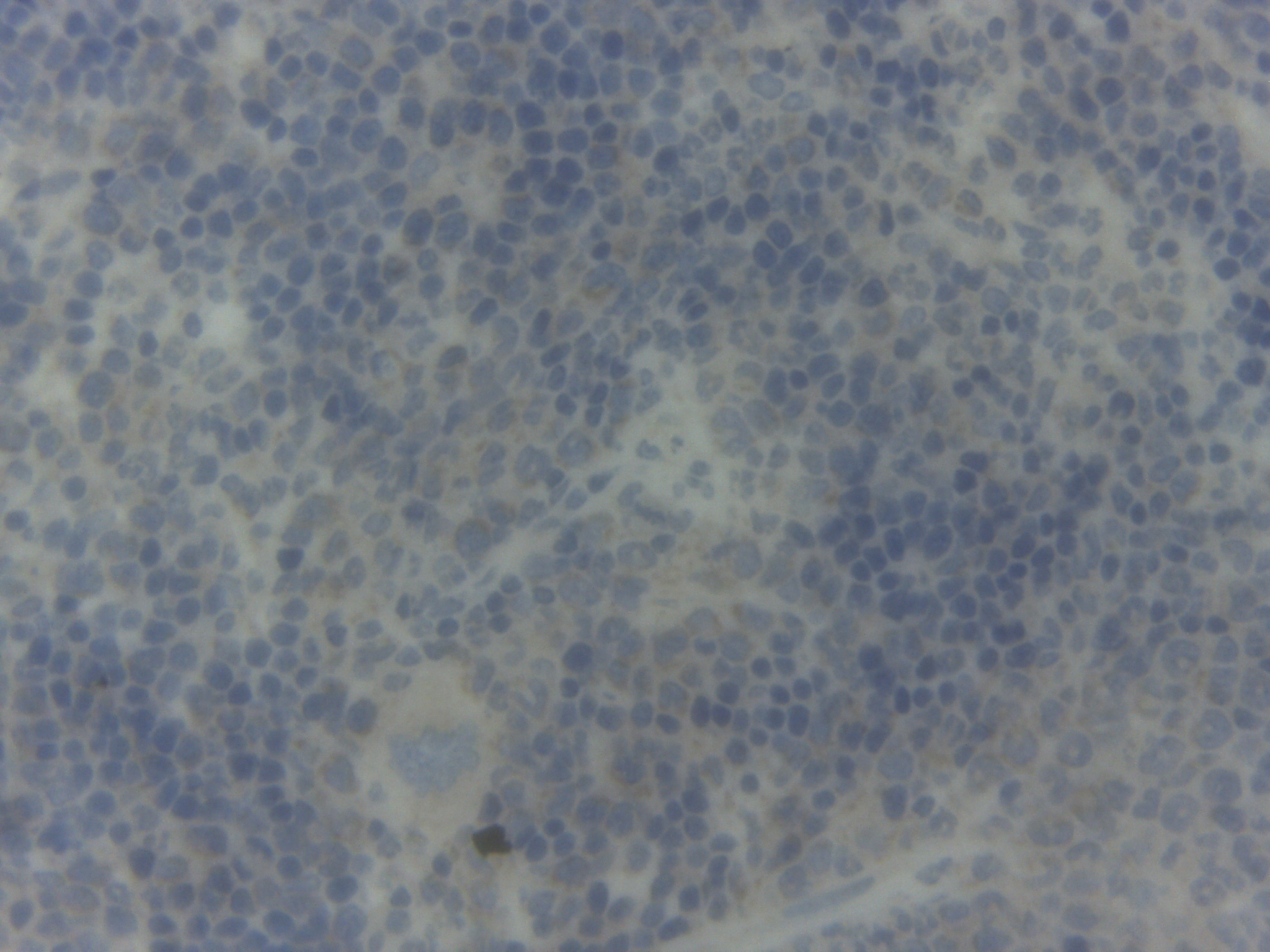

Supplement: S14 Fig — (ZIP) [file pone.0188960.s027.zip › NKp46 IHC image 24 hours/24h-1-3.jpg]

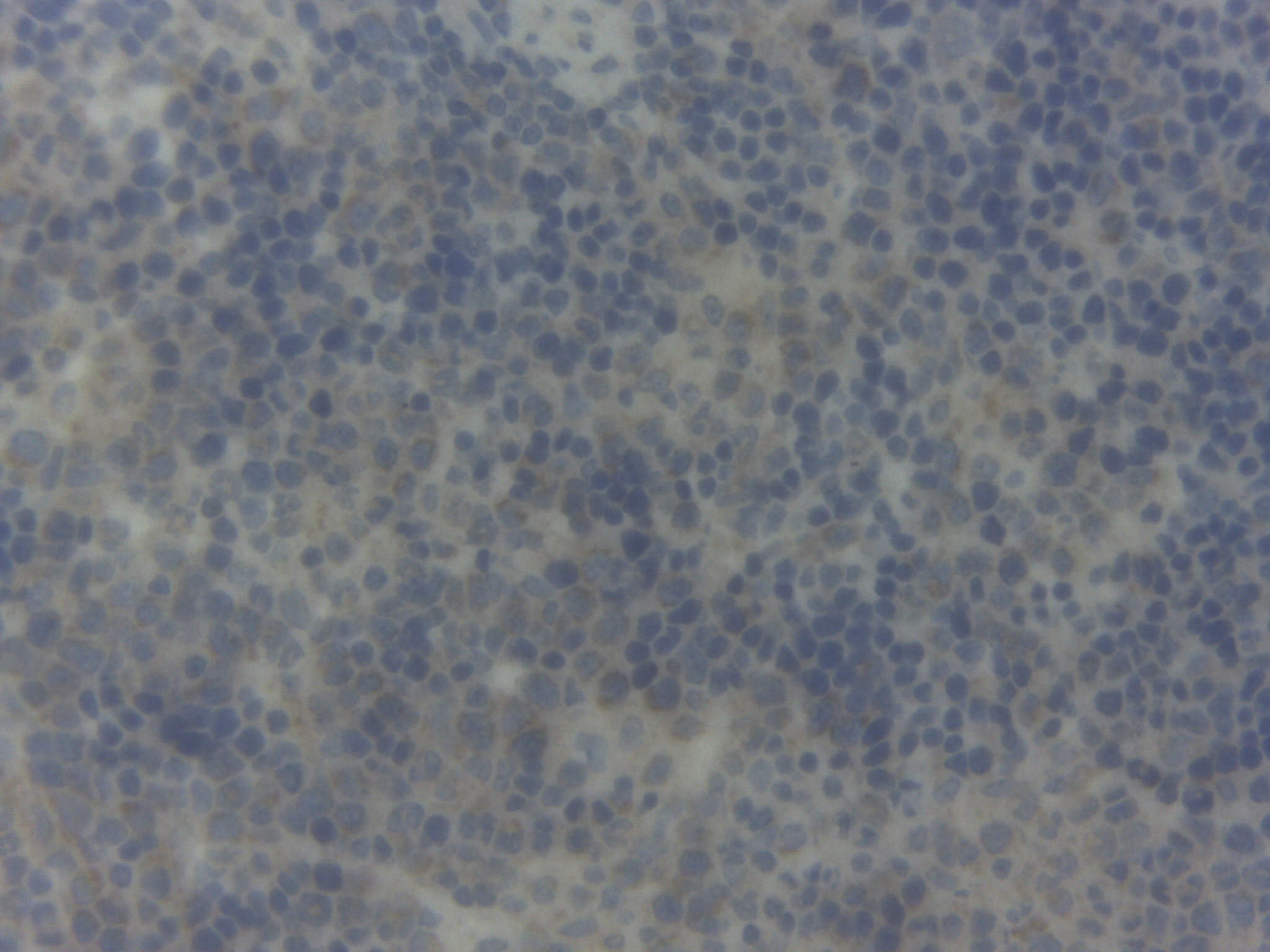

Supplement: S14 Fig — (ZIP) [file pone.0188960.s027.zip › NKp46 IHC image 24 hours/24h-1-4.jpg]

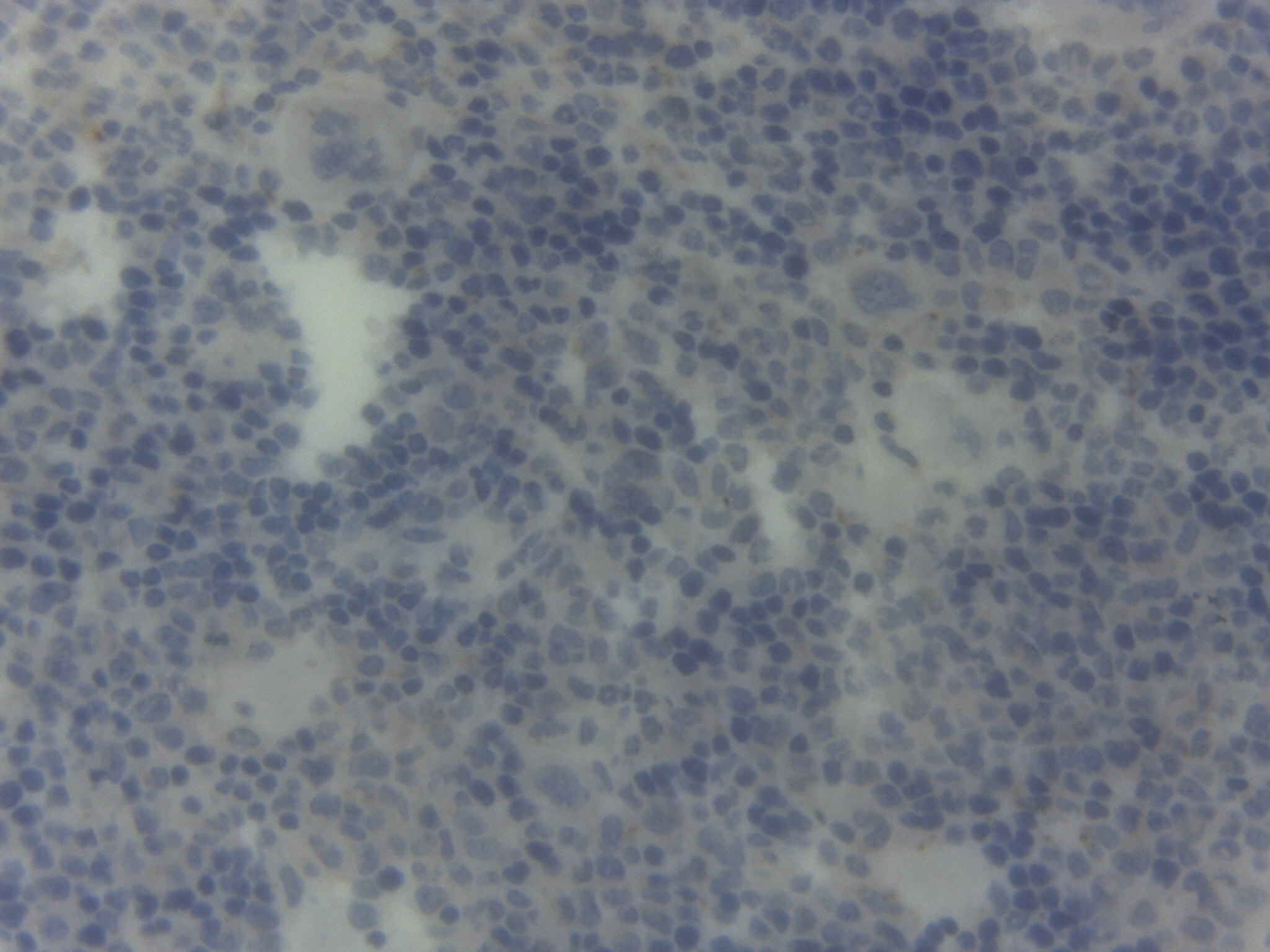

Supplement: S14 Fig — (ZIP) [file pone.0188960.s027.zip › NKp46 IHC image 24 hours/24h-1-5.jpg]

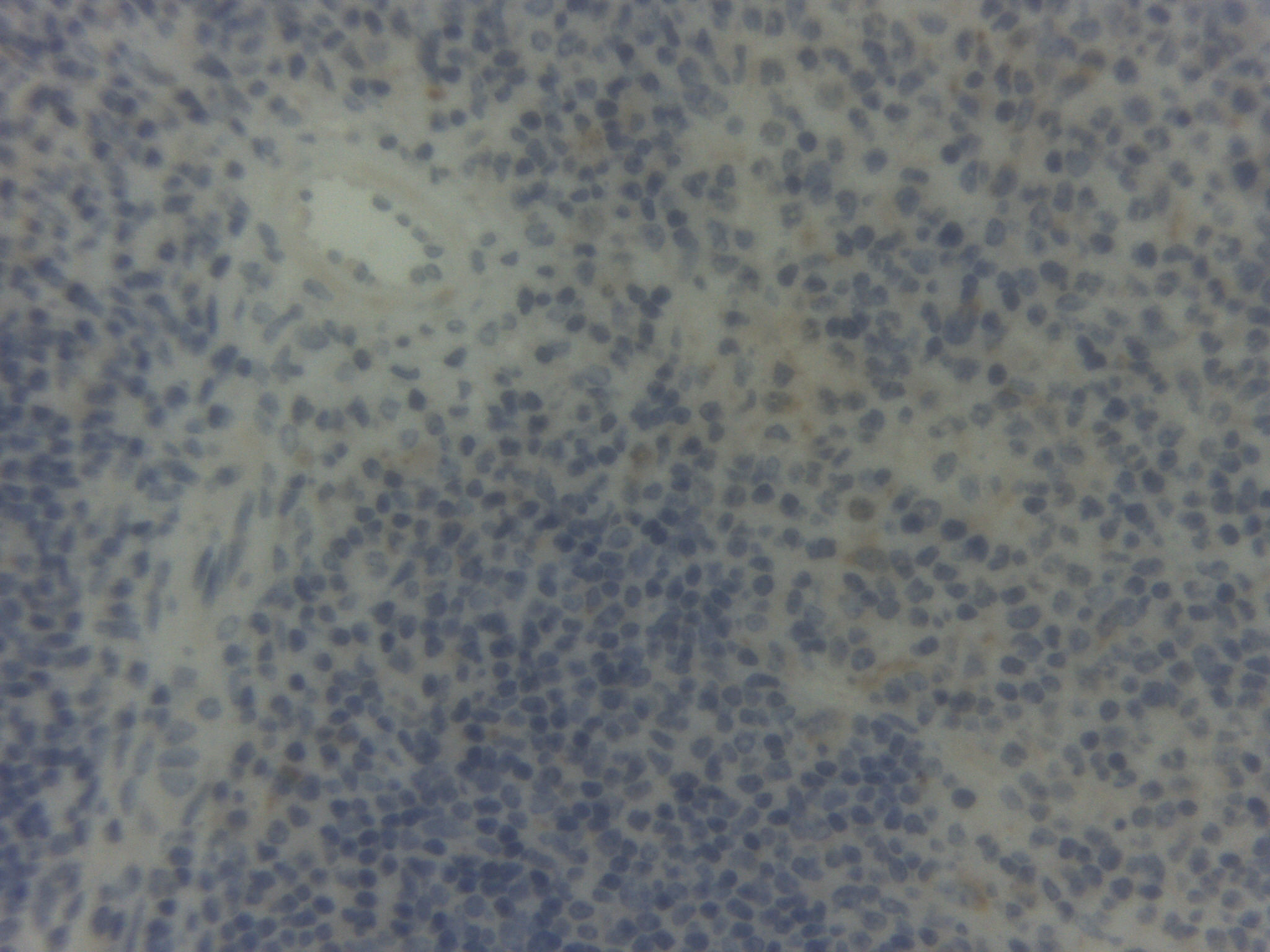

Supplement: S14 Fig — (ZIP) [file pone.0188960.s027.zip › NKp46 IHC image 24 hours/24h-2-1.jpg]

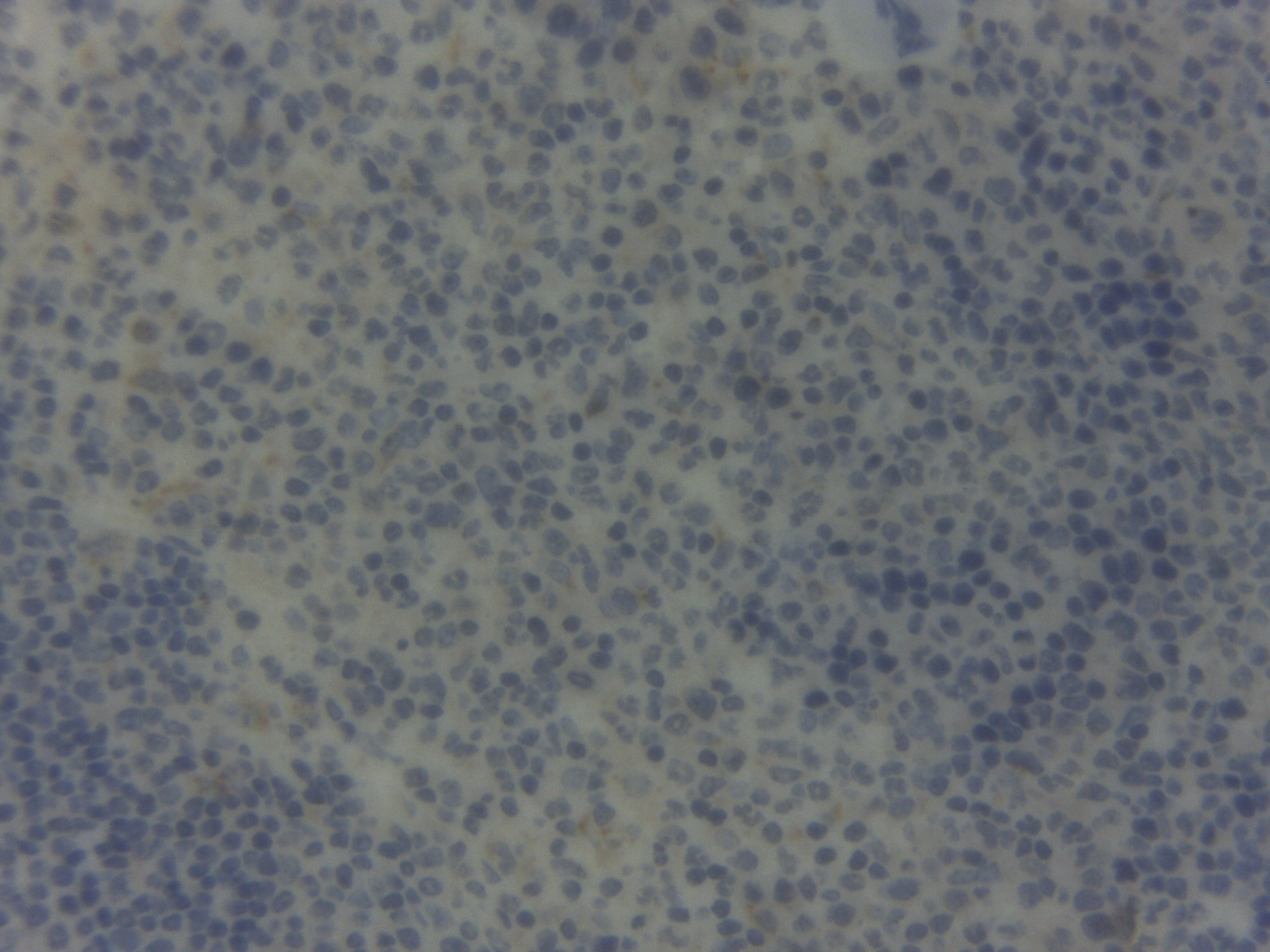

Supplement: S14 Fig — (ZIP) [file pone.0188960.s027.zip › NKp46 IHC image 24 hours/24h-2-2.jpg]

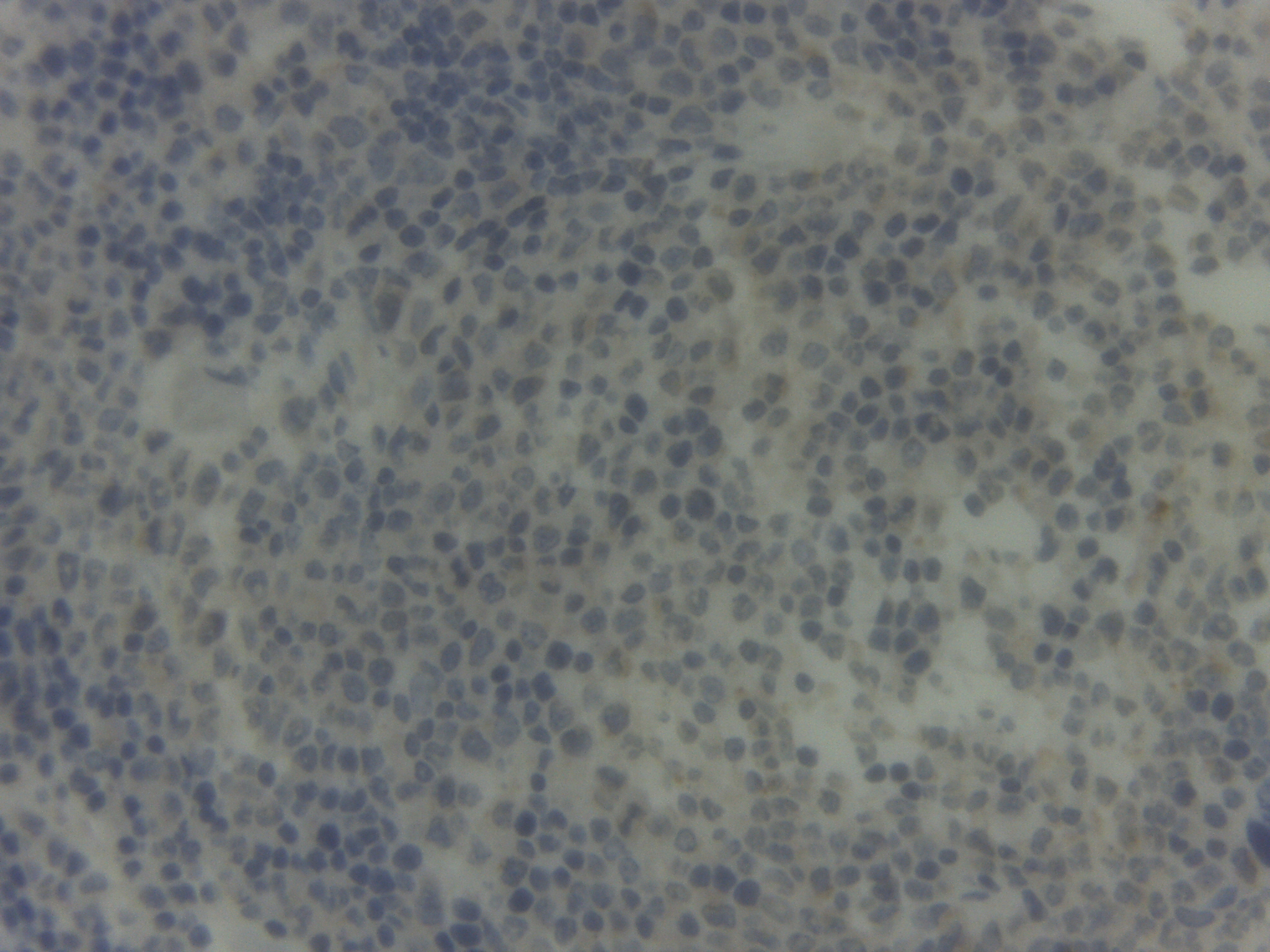

Supplement: S14 Fig — (ZIP) [file pone.0188960.s027.zip › NKp46 IHC image 24 hours/24h-2-3.jpg]

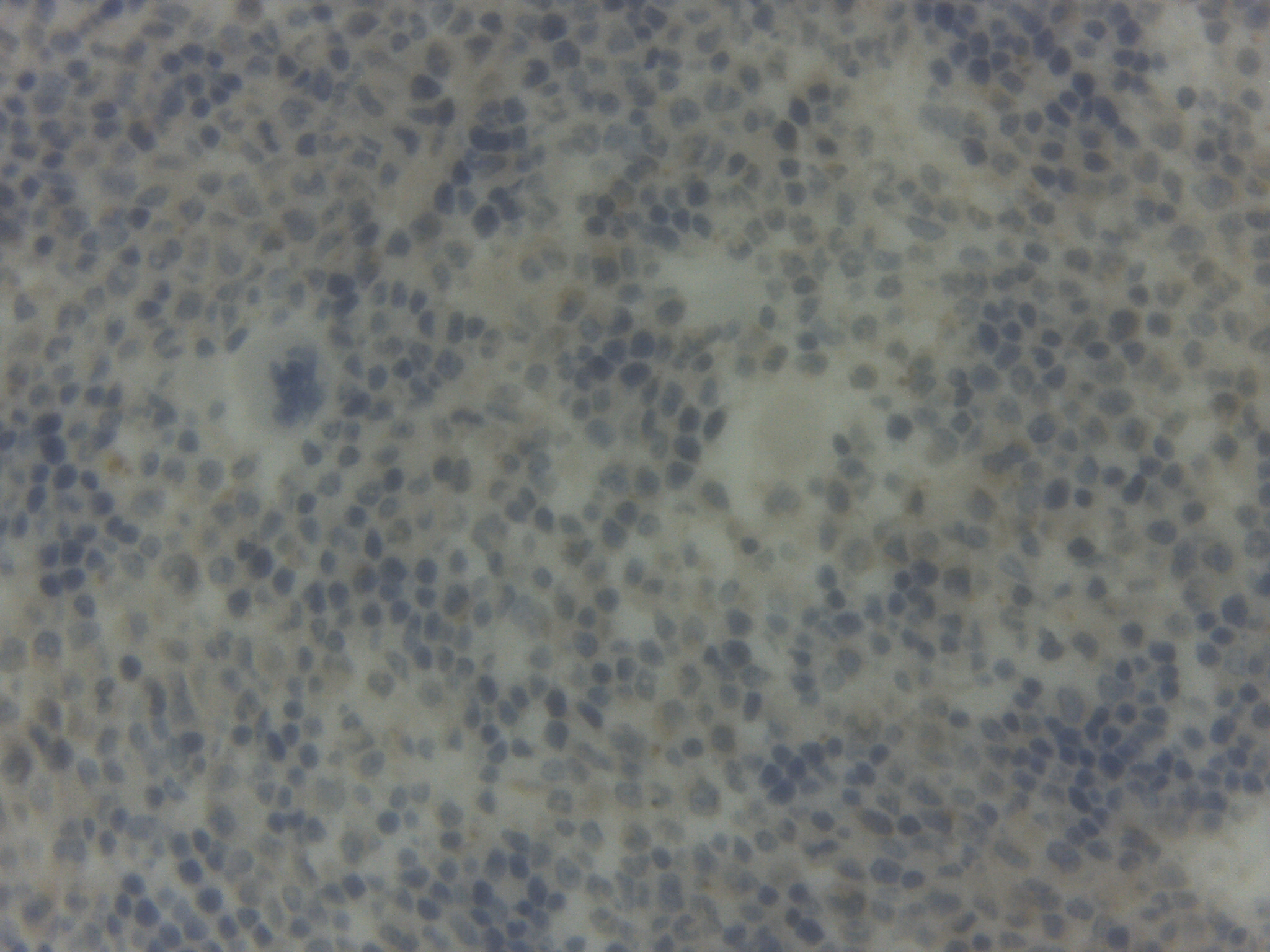

Supplement: S14 Fig — (ZIP) [file pone.0188960.s027.zip › NKp46 IHC image 24 hours/24h-2-4.jpg]

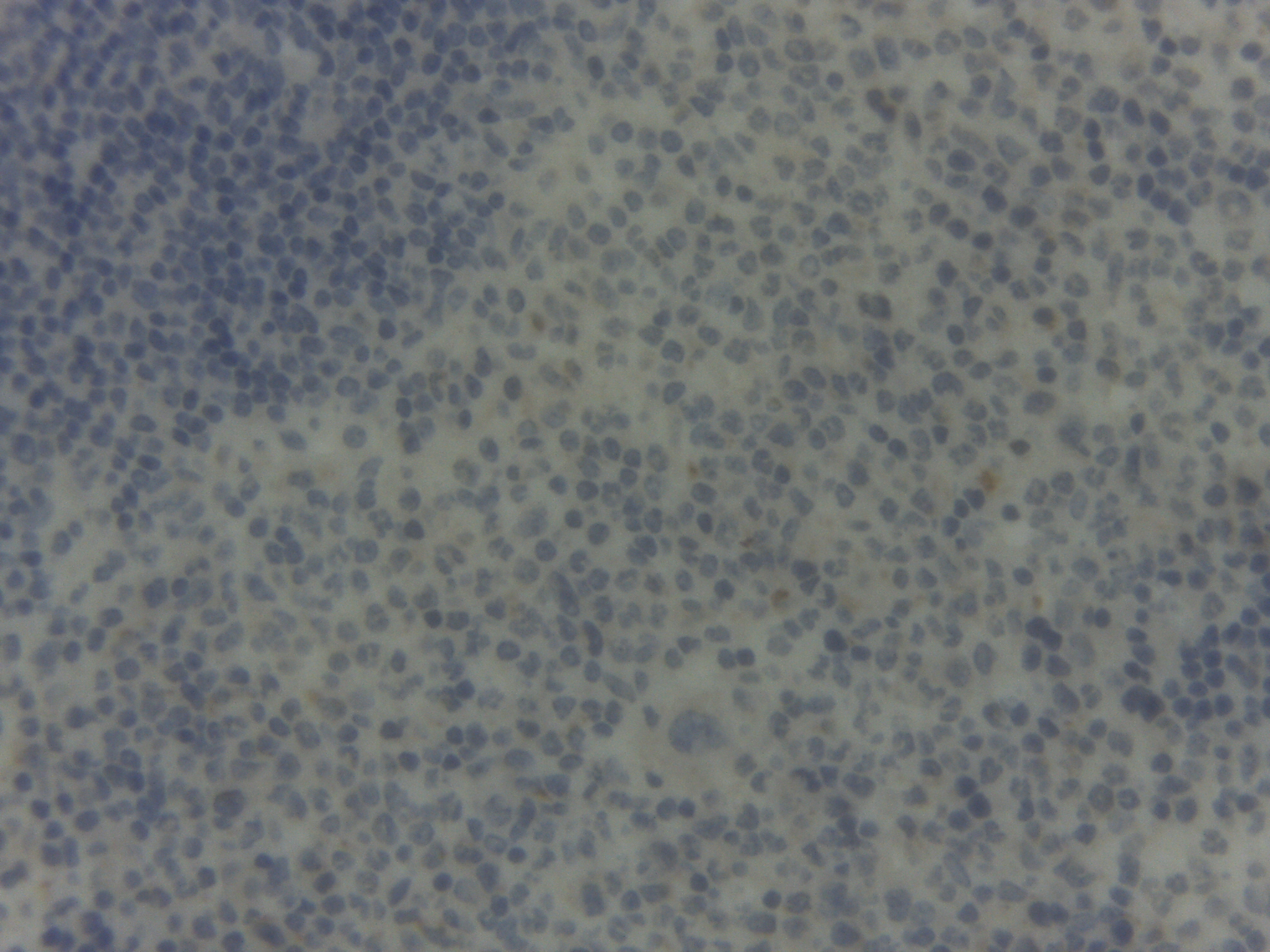

Supplement: S14 Fig — (ZIP) [file pone.0188960.s027.zip › NKp46 IHC image 24 hours/24h-2-5.jpg]

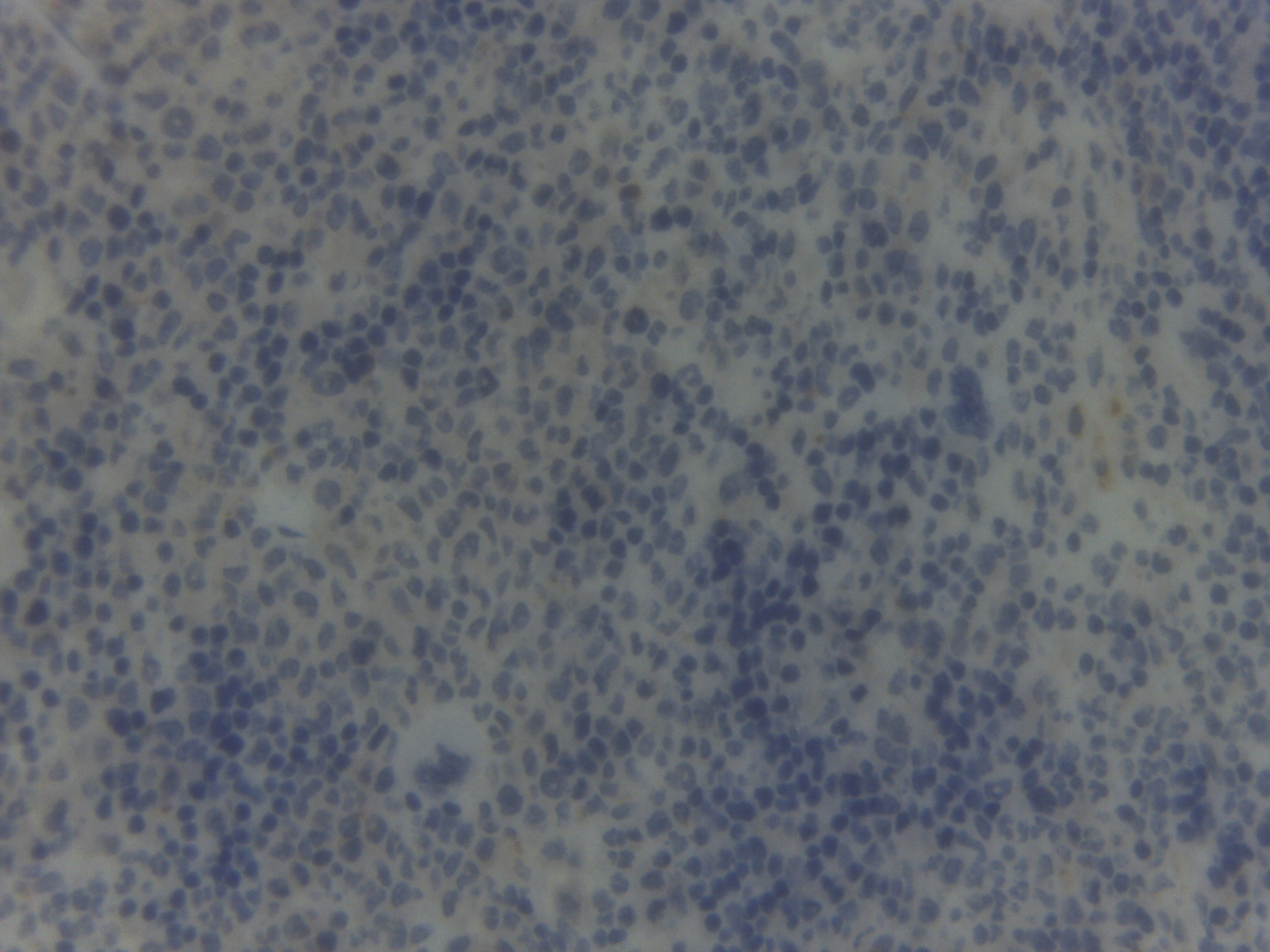

Supplement: S14 Fig — (ZIP) [file pone.0188960.s027.zip › NKp46 IHC image 24 hours/24h-3-1.jpg]

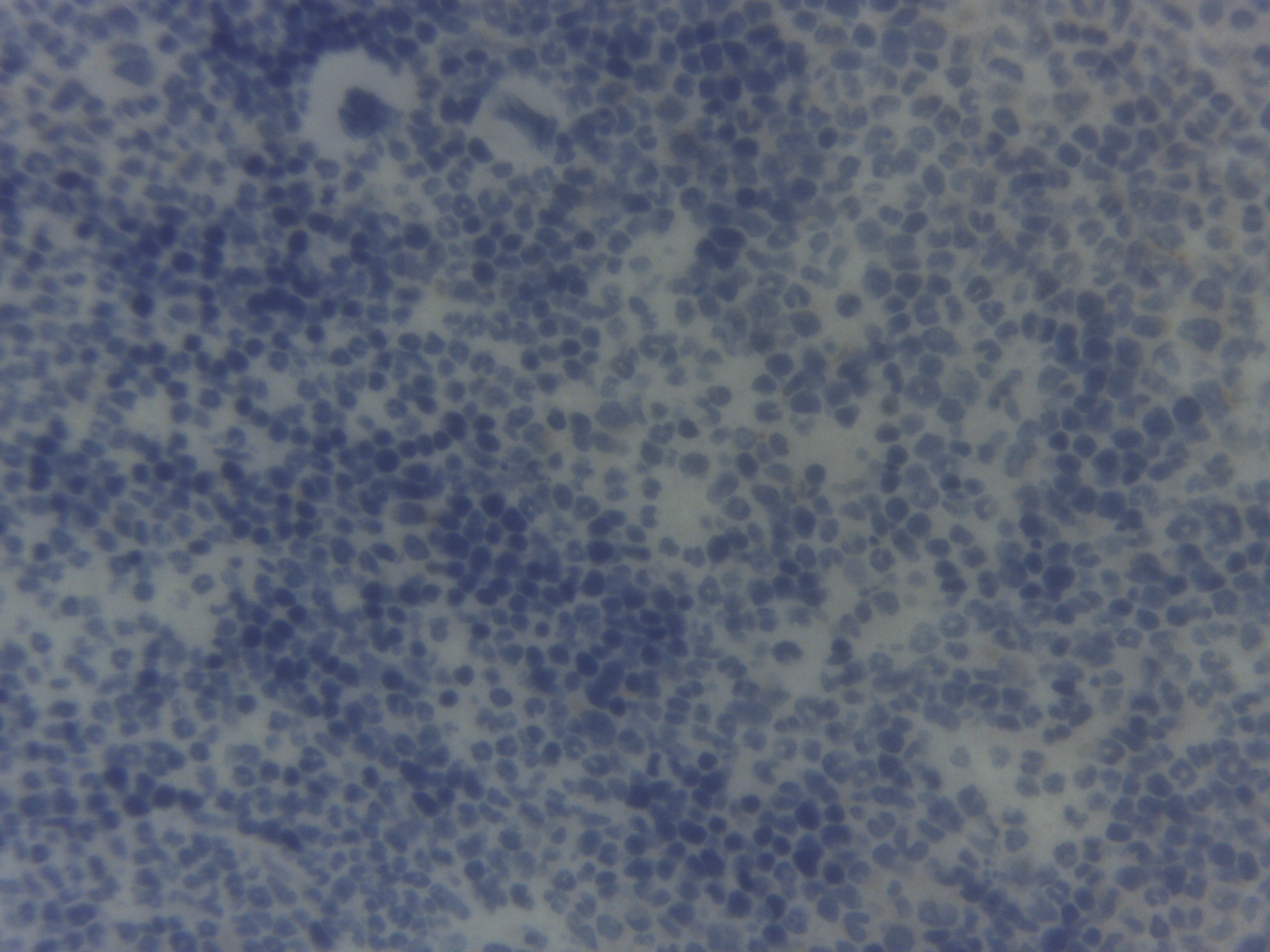

Supplement: S14 Fig — (ZIP) [file pone.0188960.s027.zip › NKp46 IHC image 24 hours/24h-3-2.jpg]

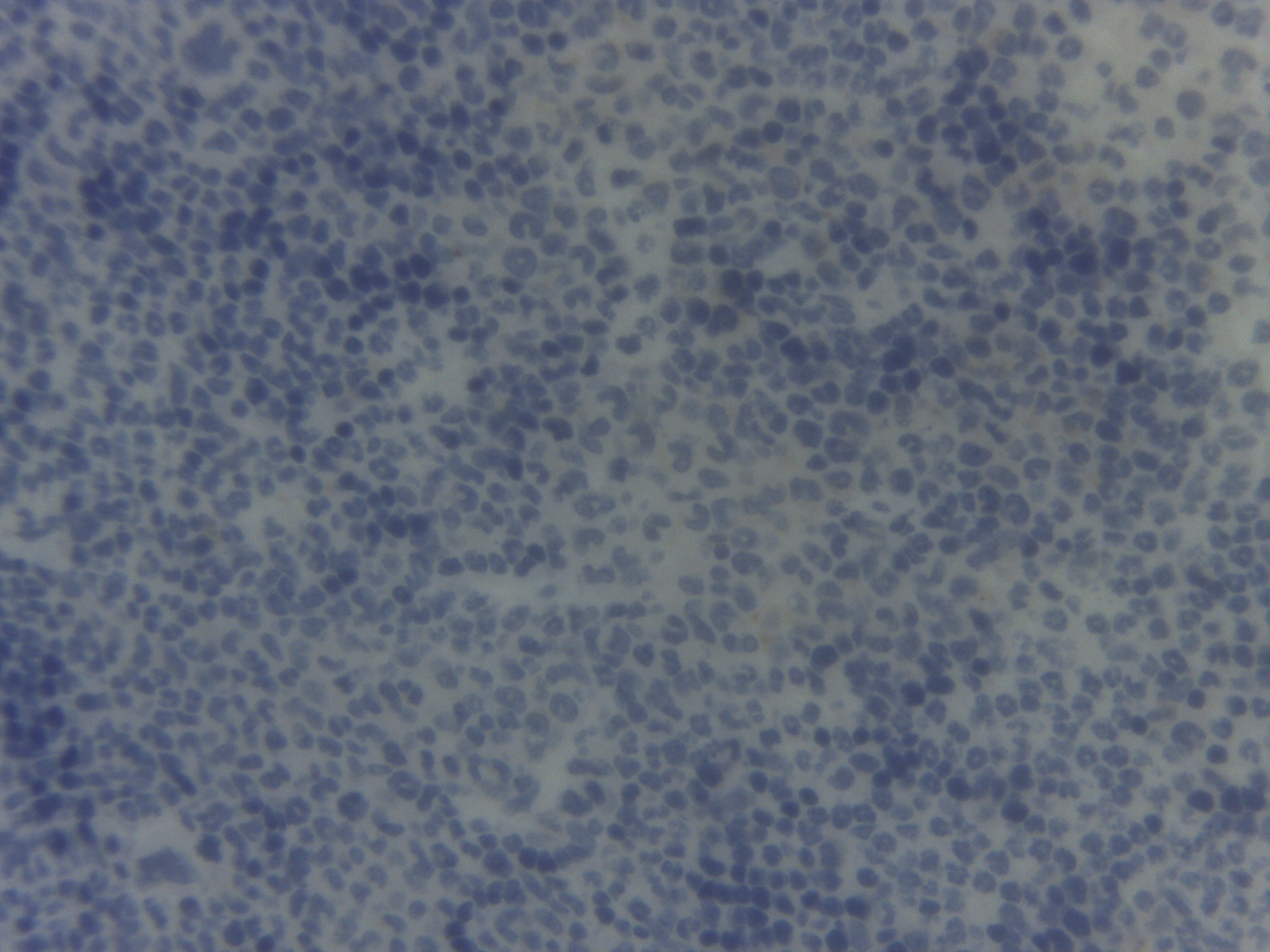

Supplement: S14 Fig — (ZIP) [file pone.0188960.s027.zip › NKp46 IHC image 24 hours/24h-3-3.jpg]

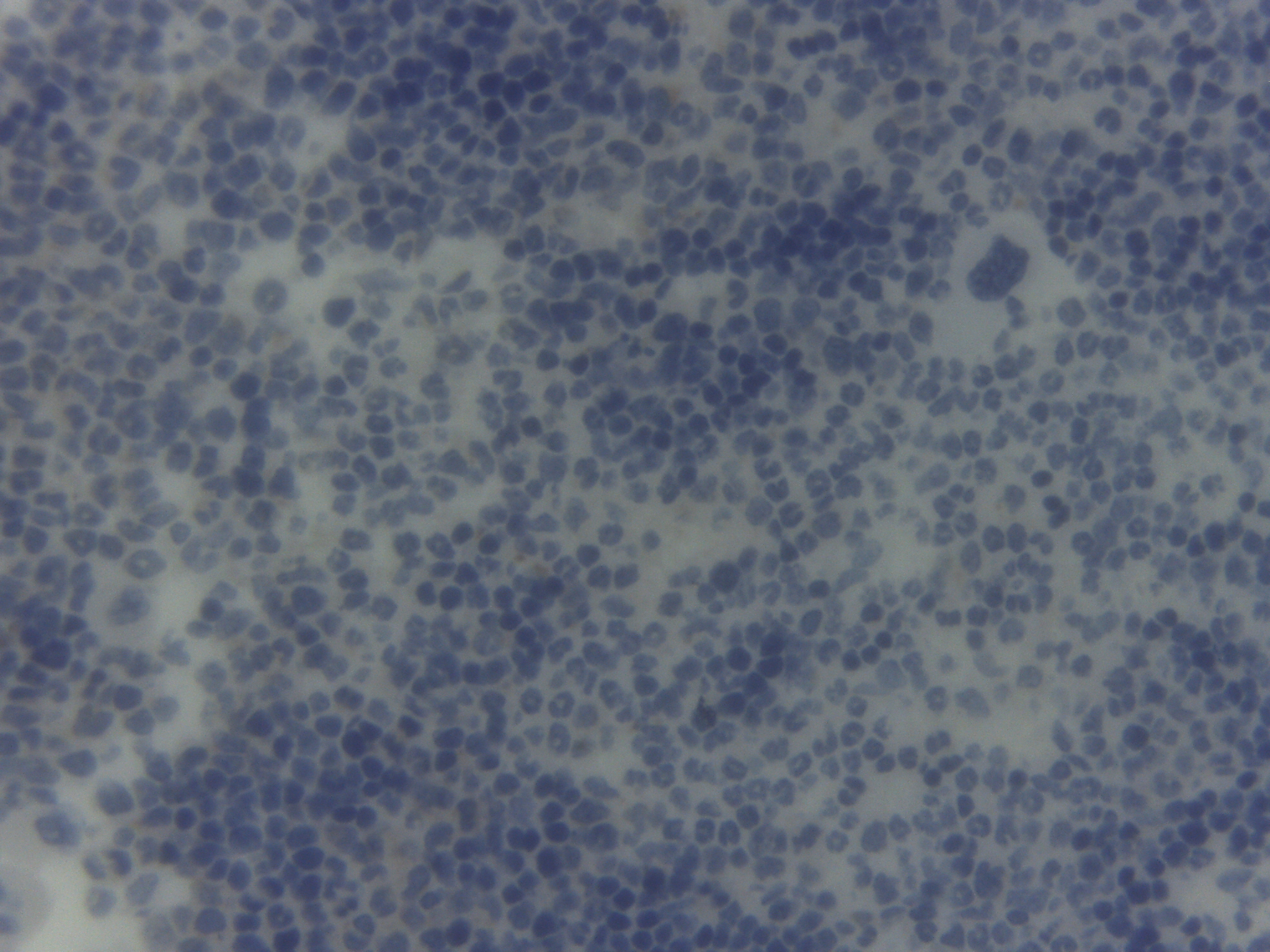

Supplement: S14 Fig — (ZIP) [file pone.0188960.s027.zip › NKp46 IHC image 24 hours/24h-3-4.jpg]

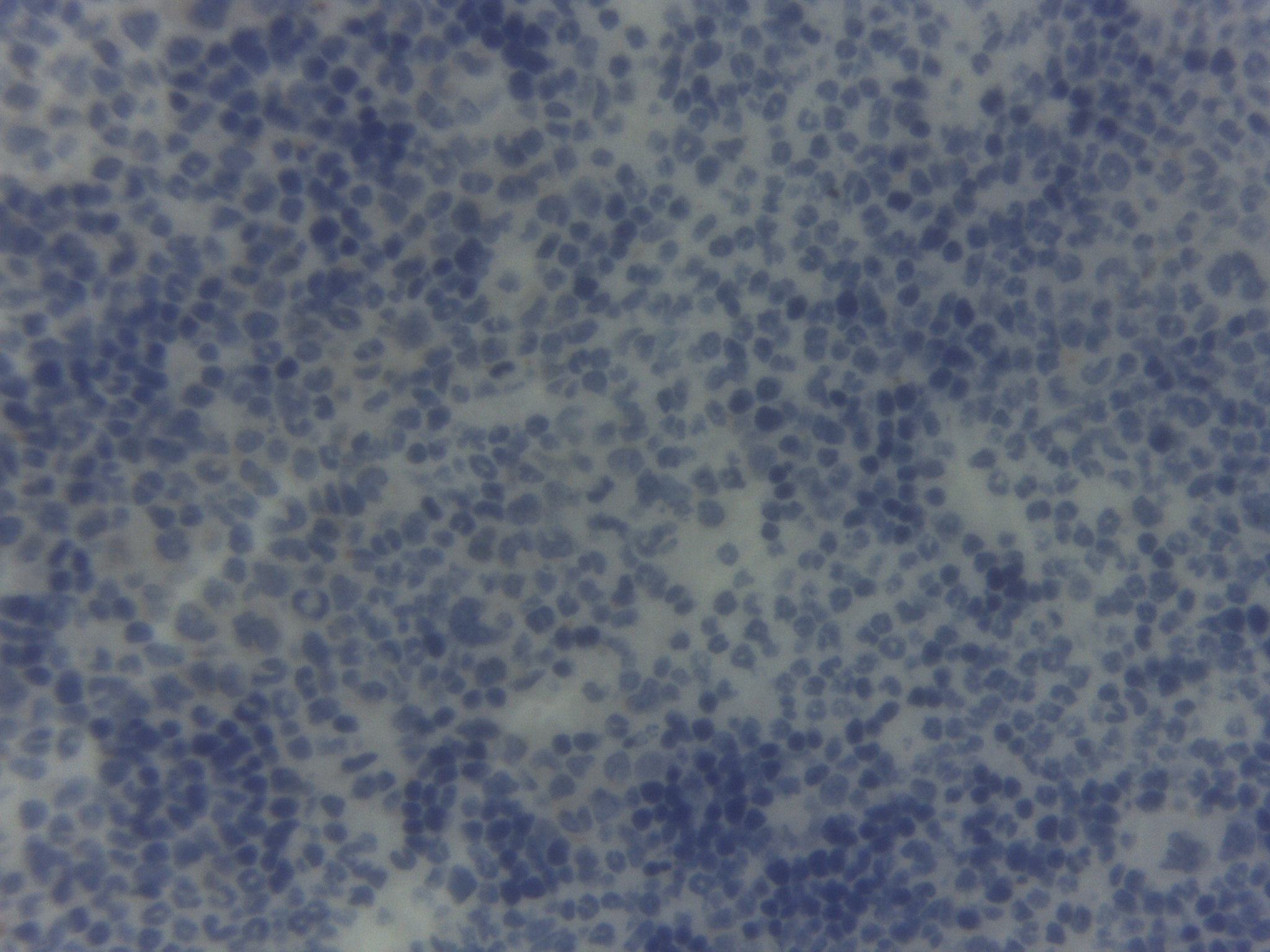

Supplement: S14 Fig — (ZIP) [file pone.0188960.s027.zip › NKp46 IHC image 24 hours/24h-3-5.jpg]

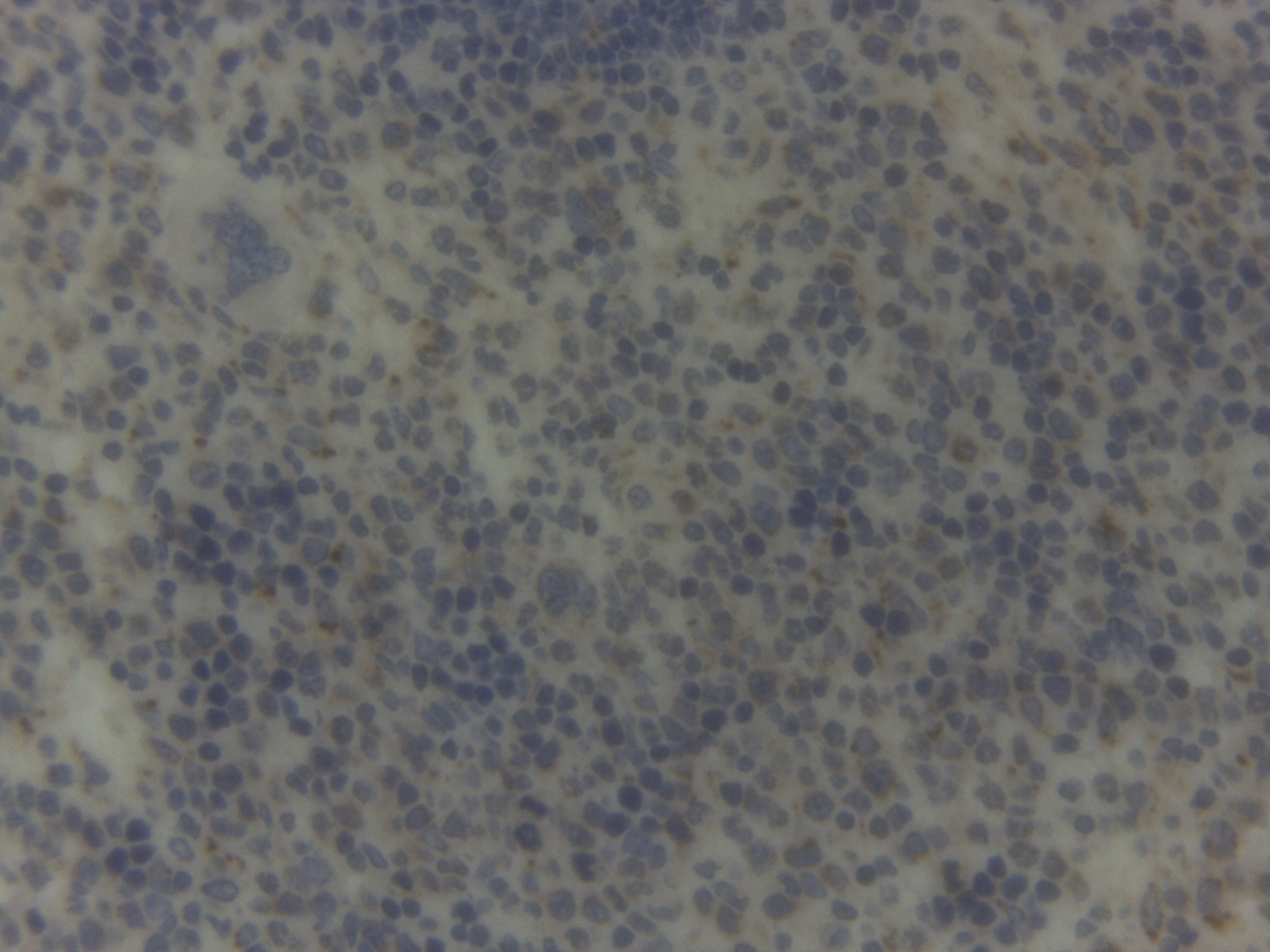

Supplement: S14 Fig — (ZIP) [file pone.0188960.s027.zip › NKp46 IHC image 24 hours/24h-4-1.jpg]

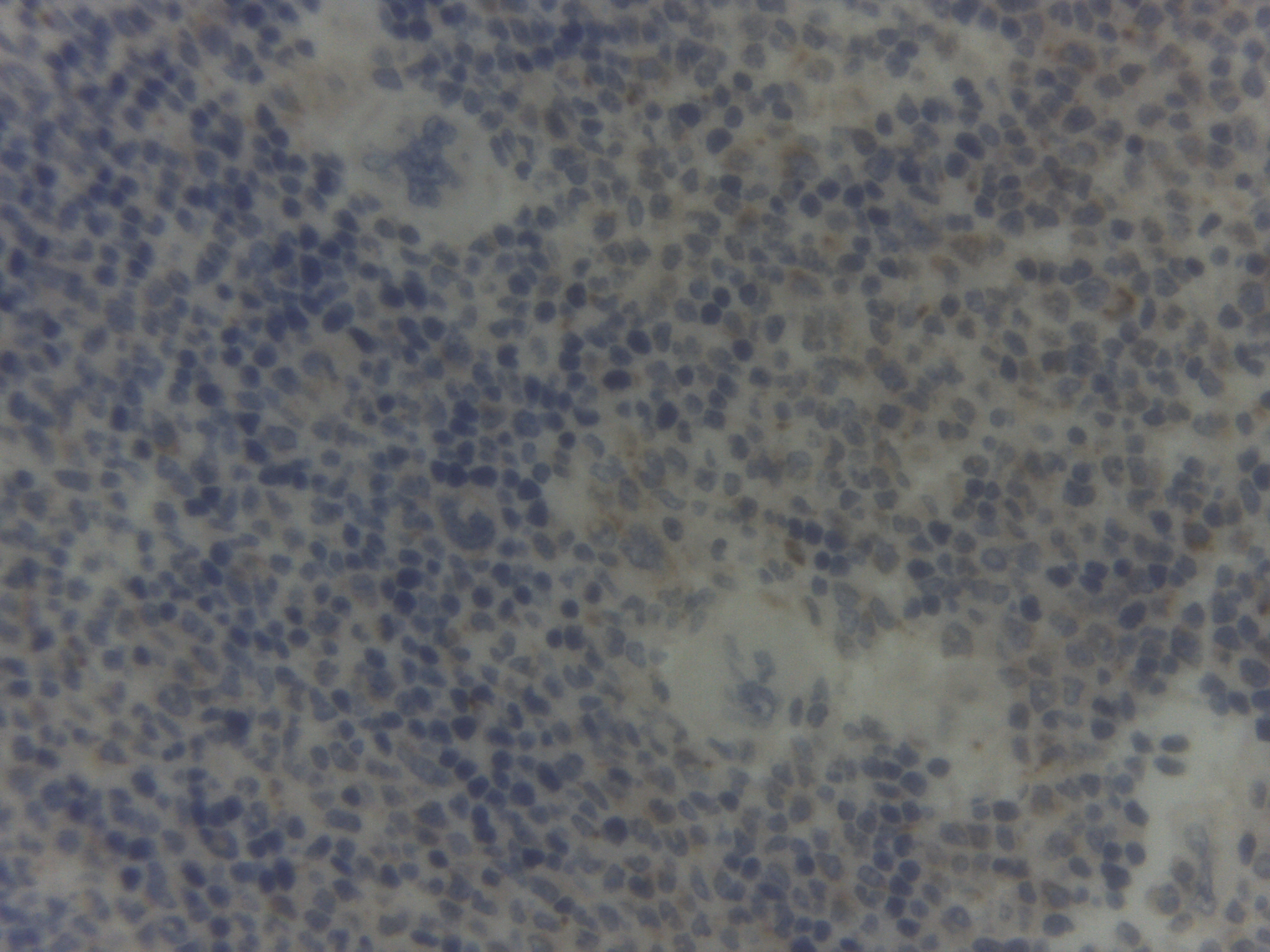

Supplement: S14 Fig — (ZIP) [file pone.0188960.s027.zip › NKp46 IHC image 24 hours/24h-4-2.jpg]

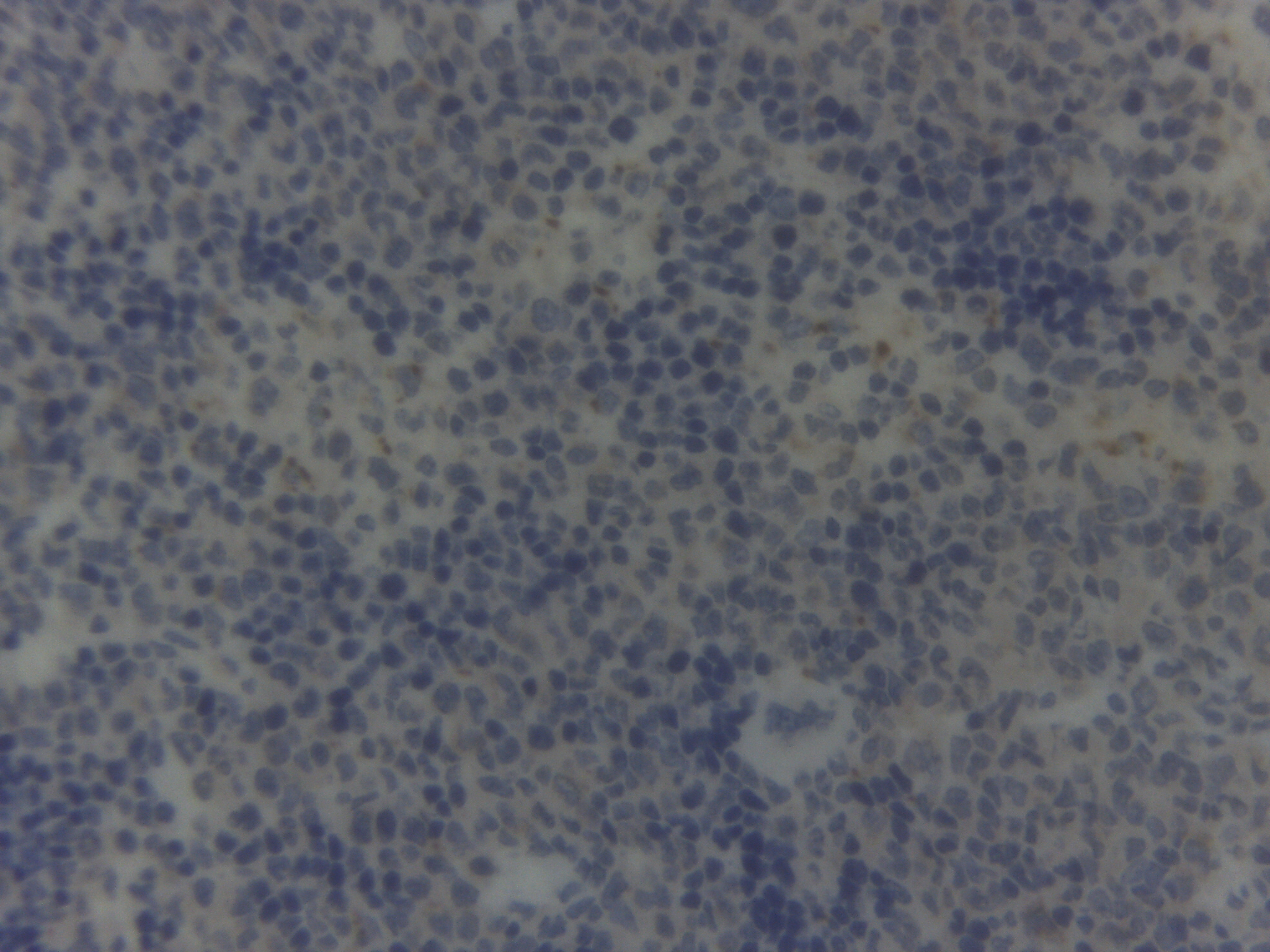

Supplement: S14 Fig — (ZIP) [file pone.0188960.s027.zip › NKp46 IHC image 24 hours/24h-4-3.jpg]

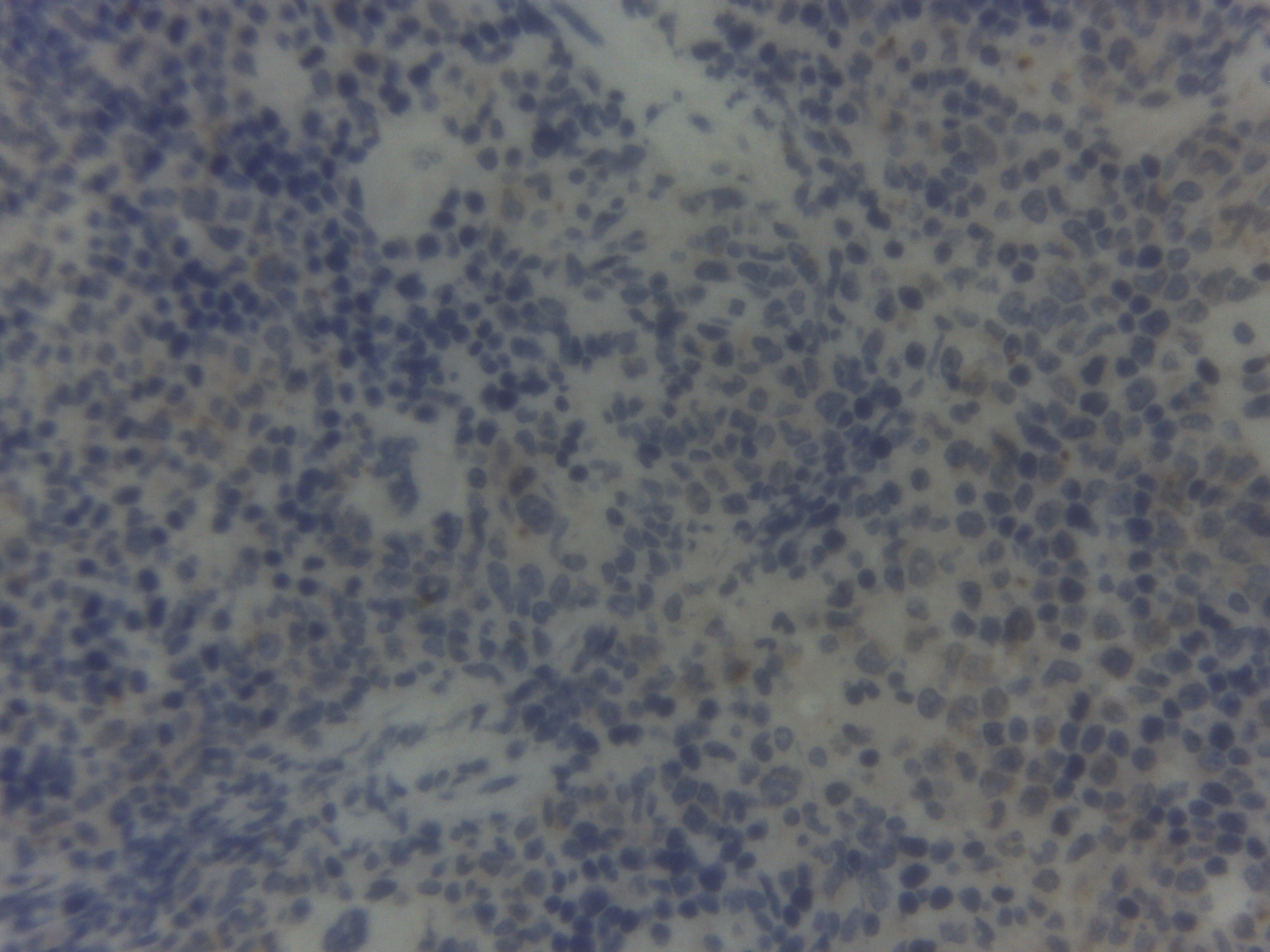

Supplement: S14 Fig — (ZIP) [file pone.0188960.s027.zip › NKp46 IHC image 24 hours/24h-4-4.jpg]

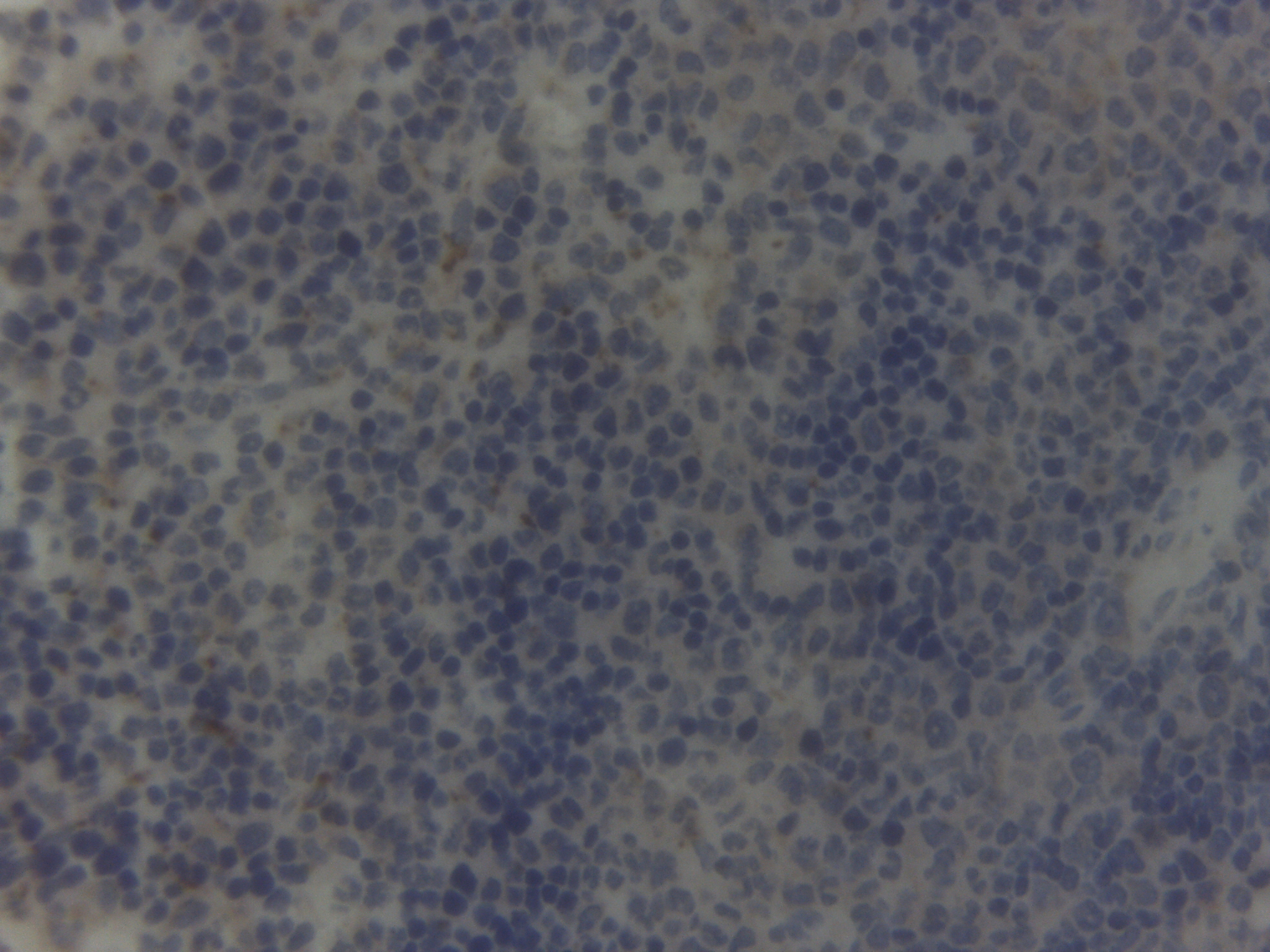

Supplement: S14 Fig — (ZIP) [file pone.0188960.s027.zip › NKp46 IHC image 24 hours/24h-4-5.jpg]

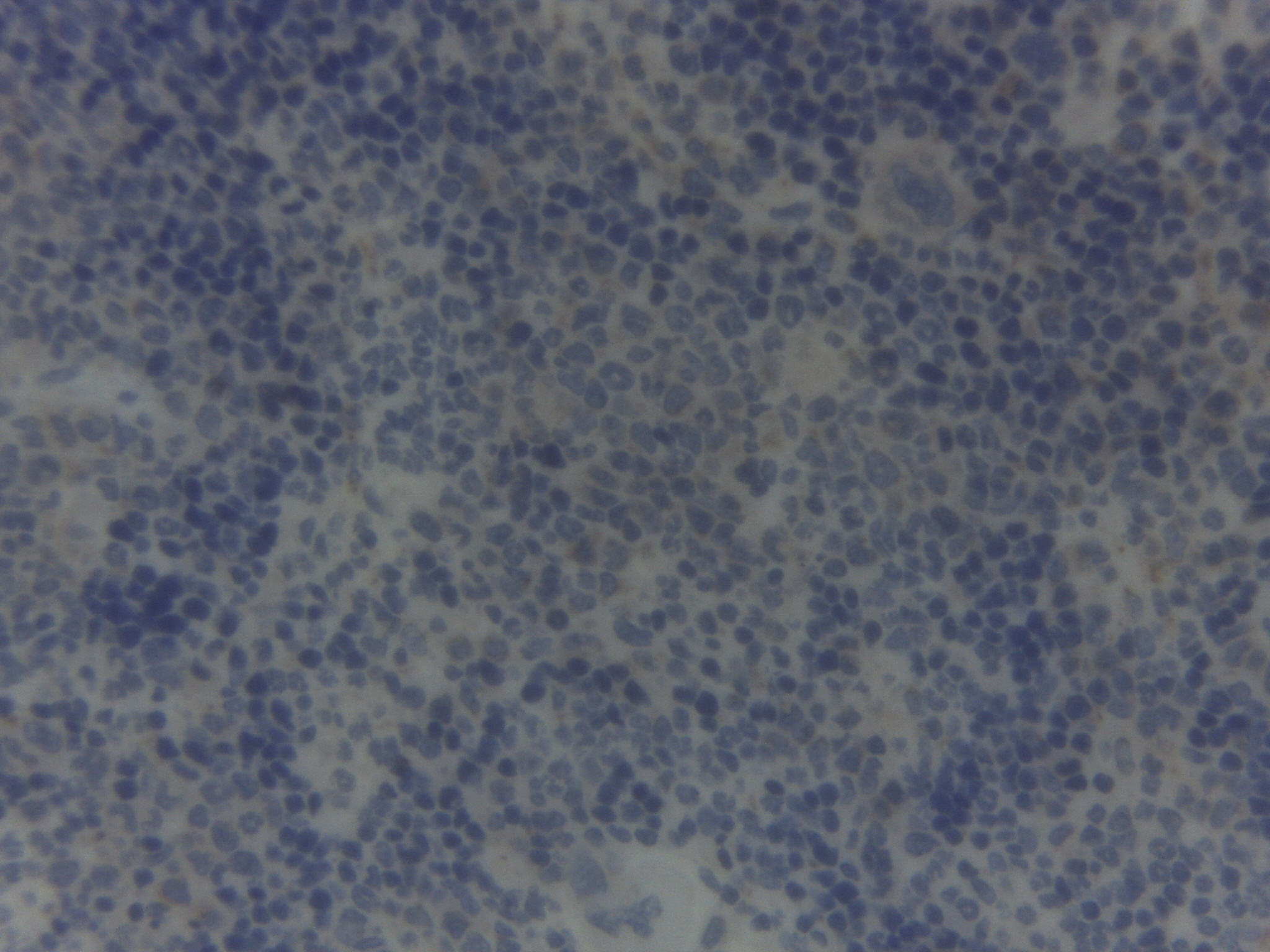

Supplement: S14 Fig — (ZIP) [file pone.0188960.s027.zip › NKp46 IHC image 24 hours/24h-5-1.jpg]

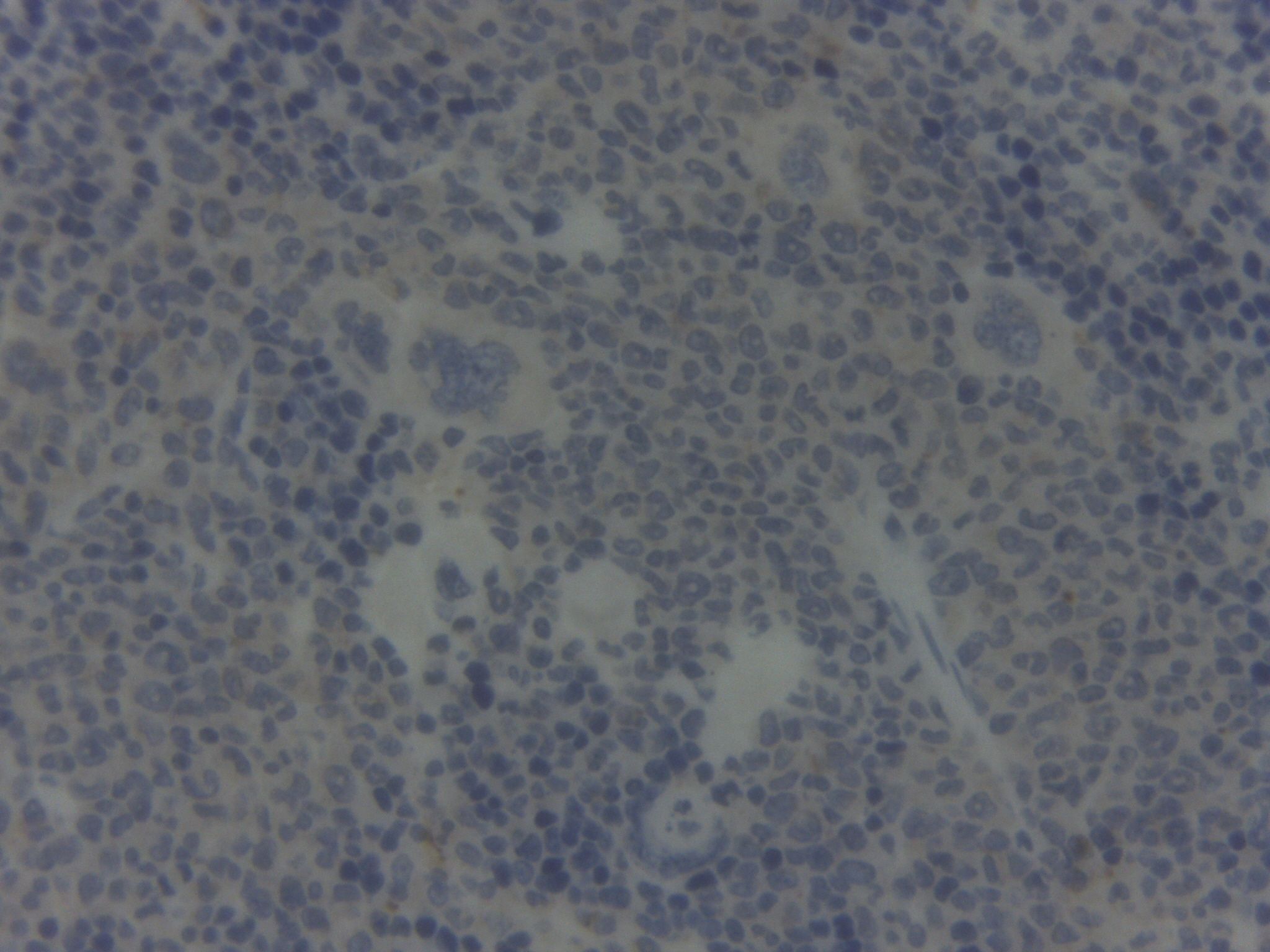

Supplement: S14 Fig — (ZIP) [file pone.0188960.s027.zip › NKp46 IHC image 24 hours/24h-5-2.jpg]

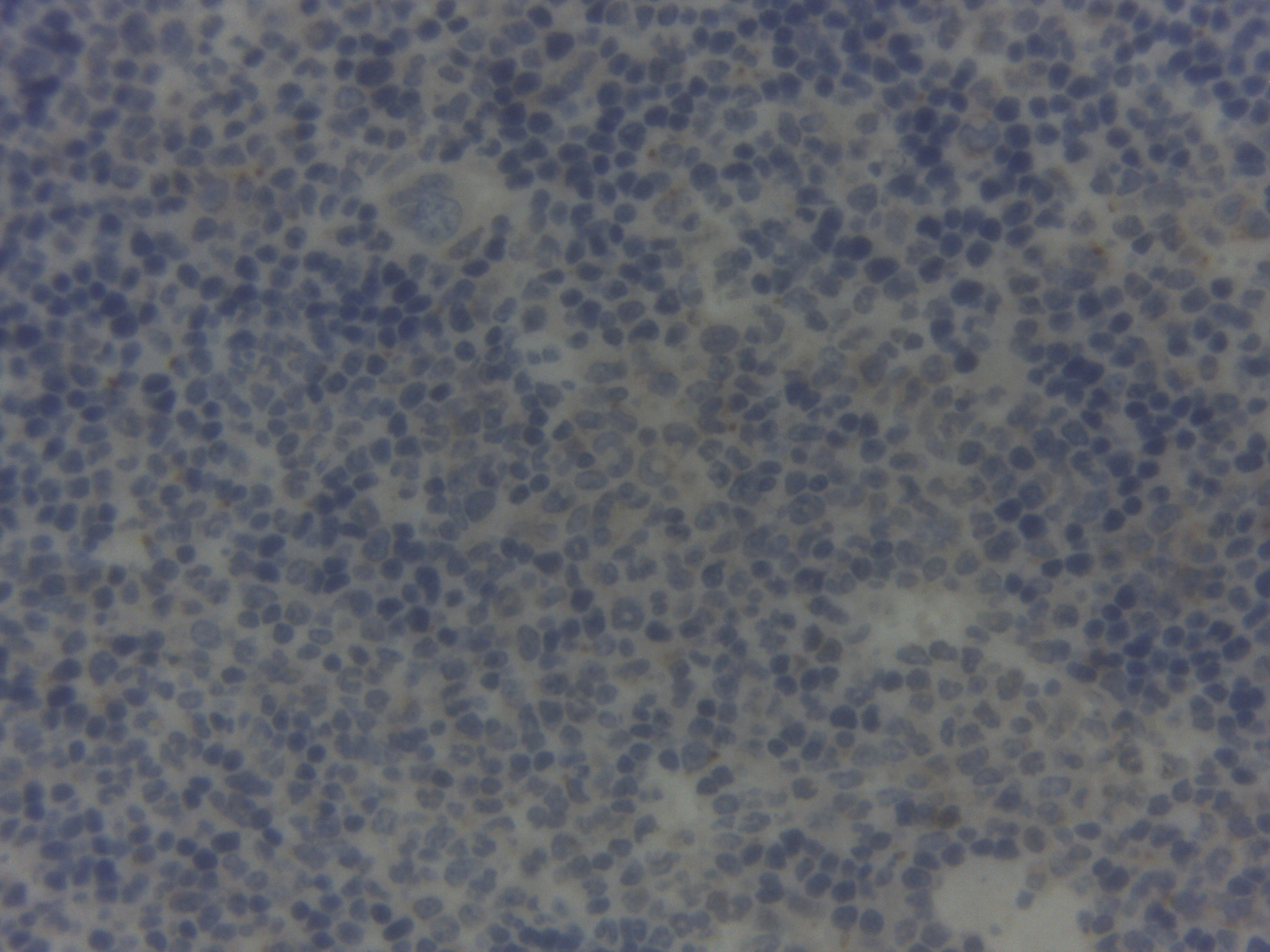

Supplement: S14 Fig — (ZIP) [file pone.0188960.s027.zip › NKp46 IHC image 24 hours/24h-5-3.jpg]

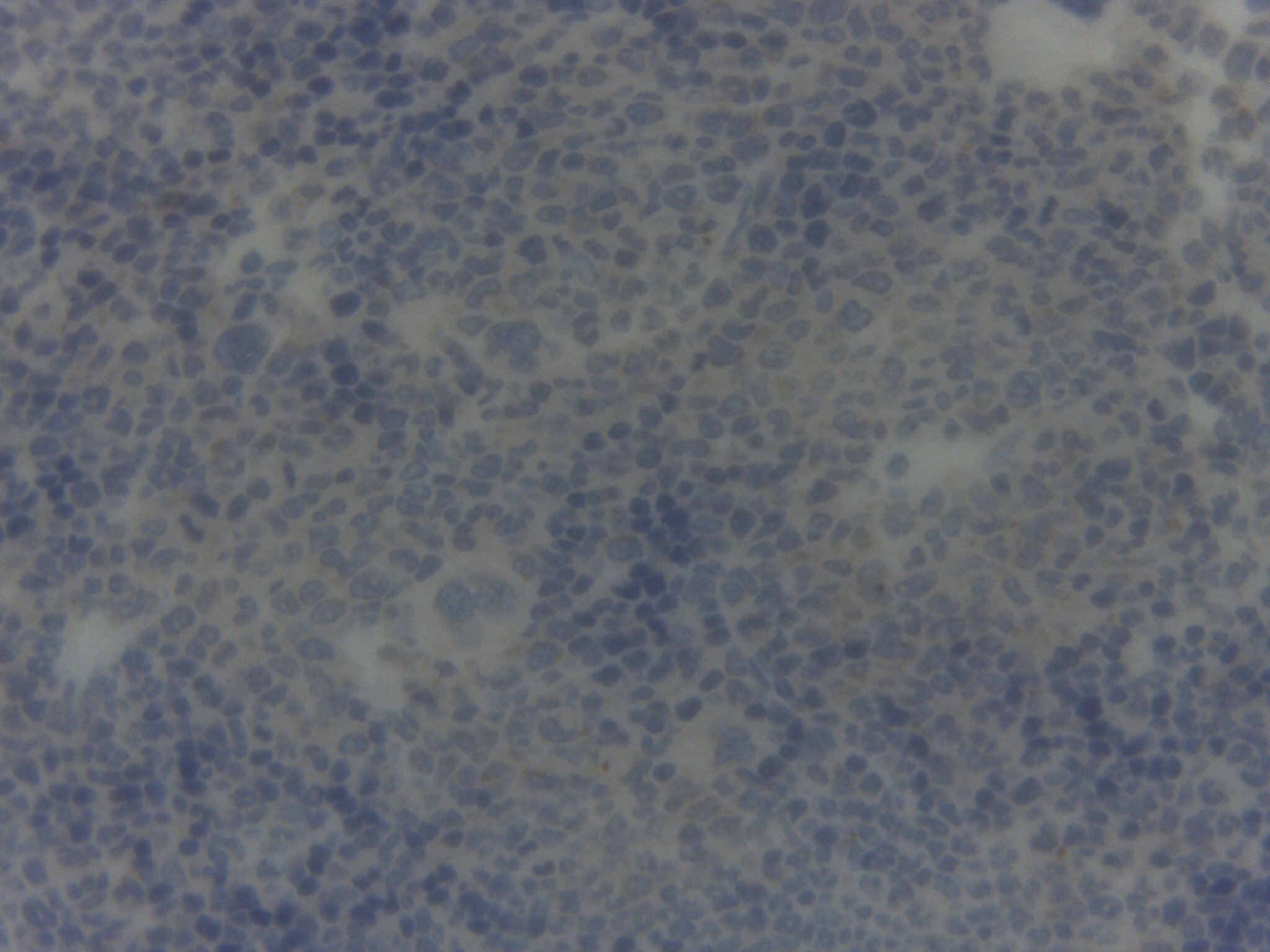

Supplement: S14 Fig — (ZIP) [file pone.0188960.s027.zip › NKp46 IHC image 24 hours/24h-5-4.jpg]

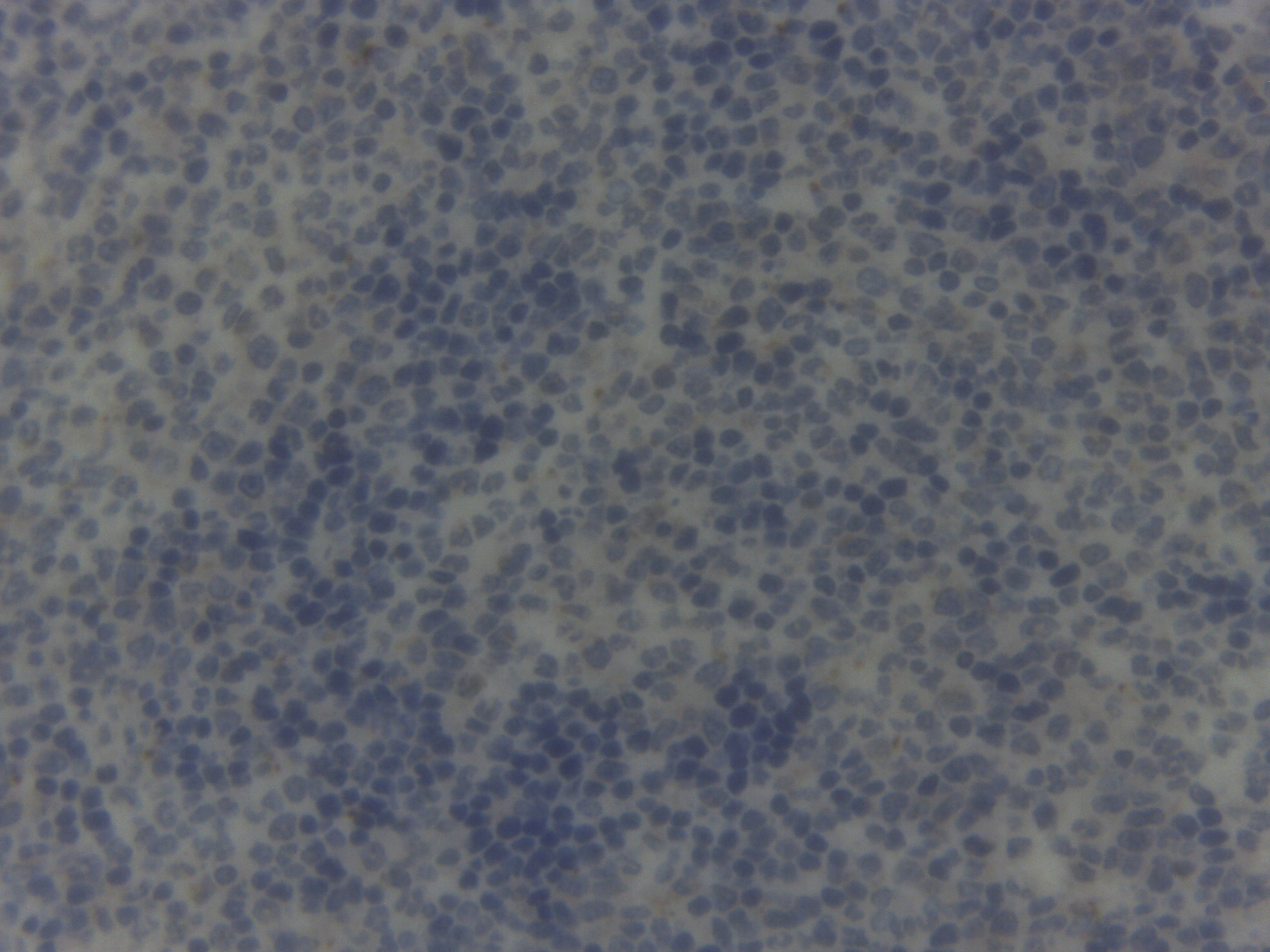

Supplement: S14 Fig — (ZIP) [file pone.0188960.s027.zip › NKp46 IHC image 24 hours/24h-5-5.jpg]

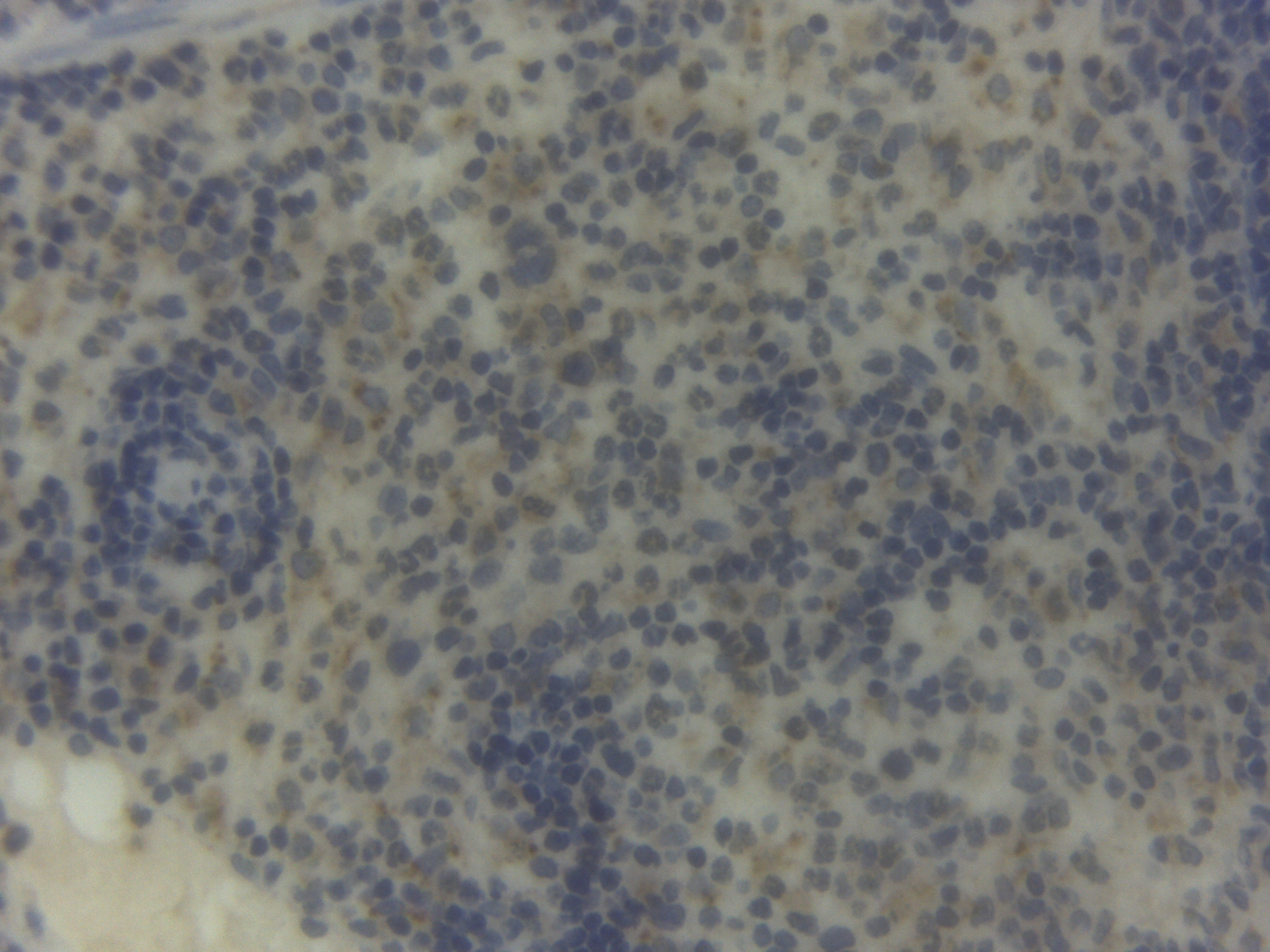

Supplement: S14 Fig — (ZIP) [file pone.0188960.s027.zip › NKp46 IHC image 24 hours/24h-6-1.jpg]

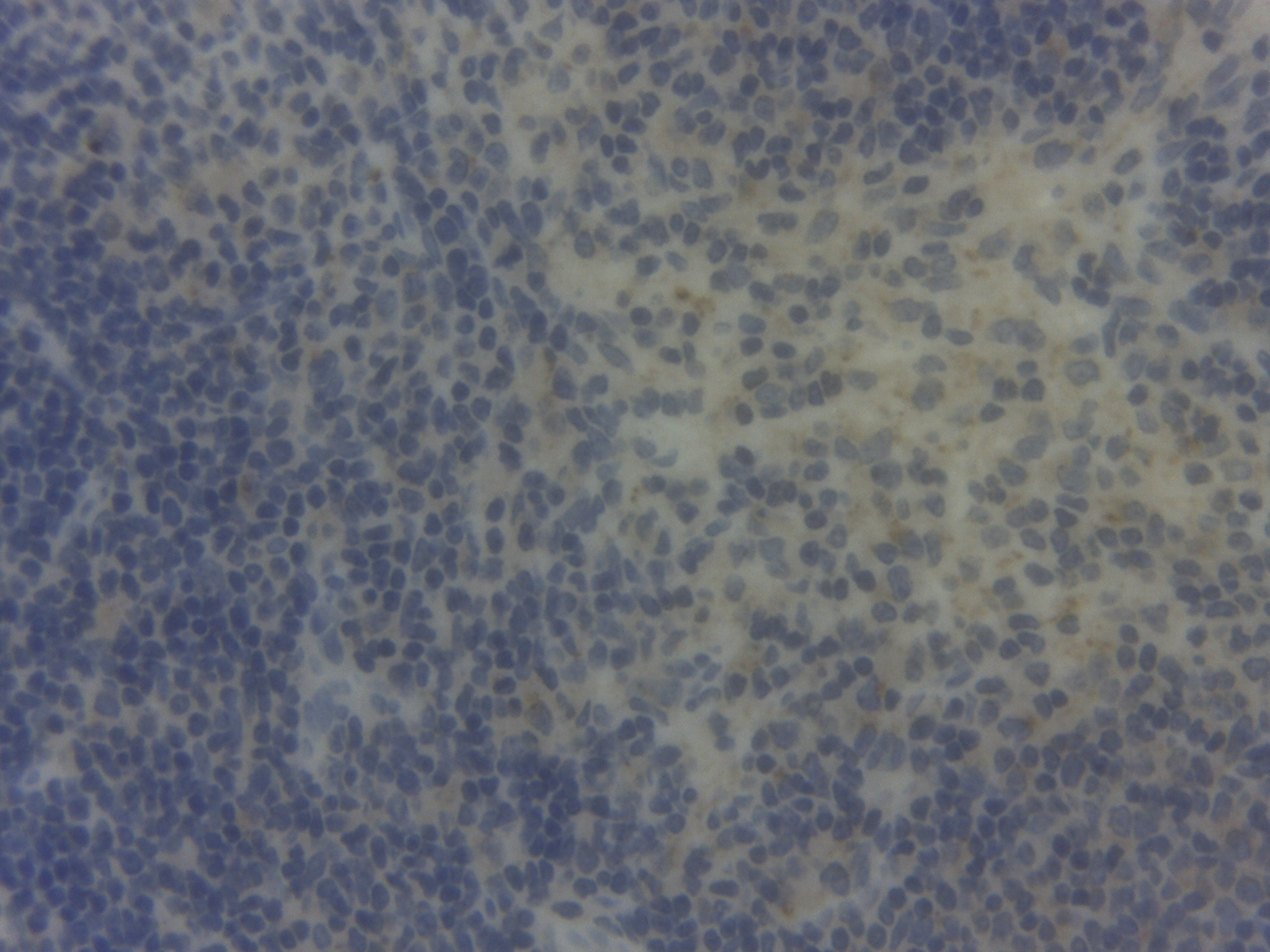

Supplement: S14 Fig — (ZIP) [file pone.0188960.s027.zip › NKp46 IHC image 24 hours/24h-6-2.jpg]

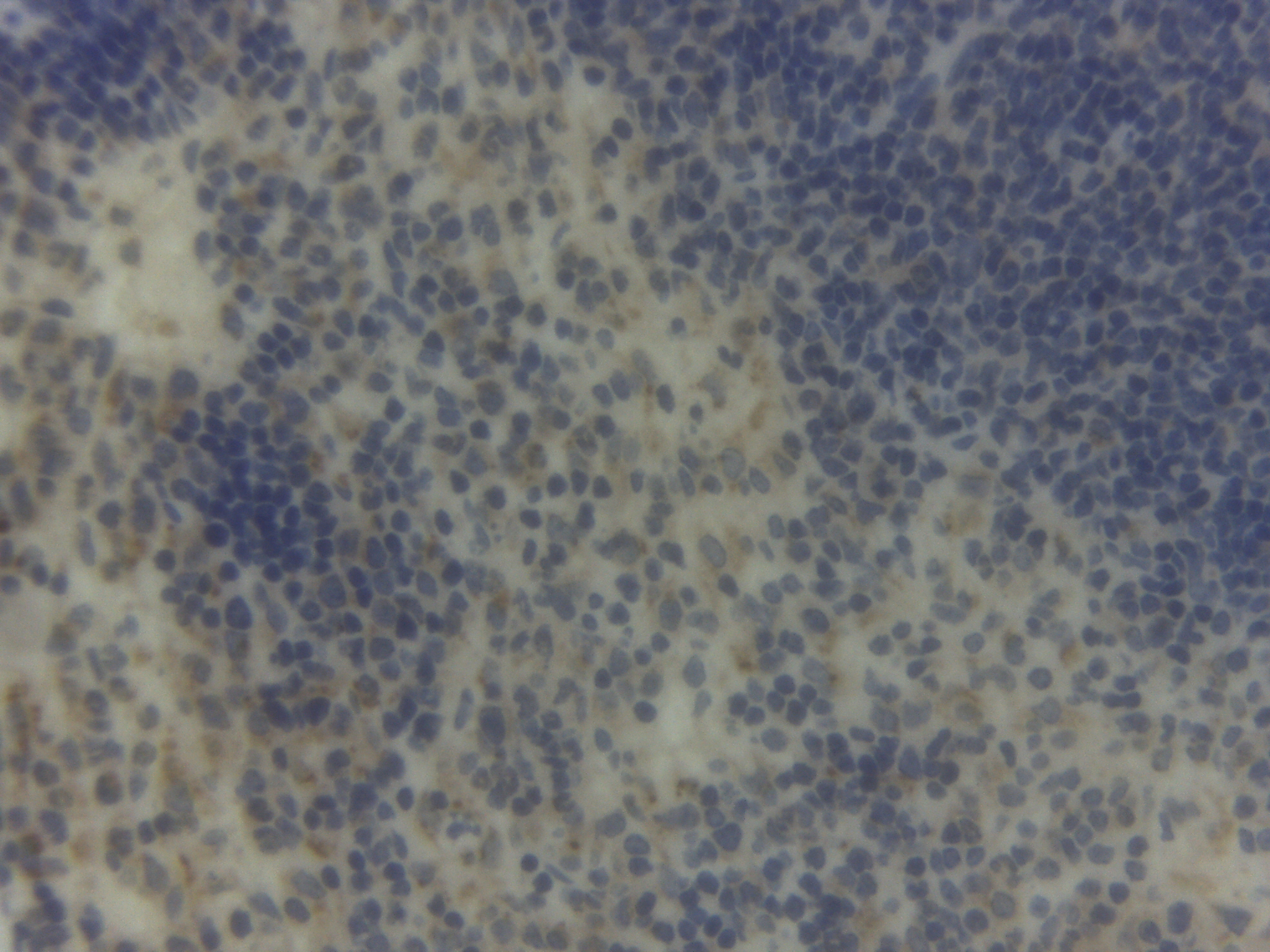

Supplement: S14 Fig — (ZIP) [file pone.0188960.s027.zip › NKp46 IHC image 24 hours/24h-6-3.jpg]

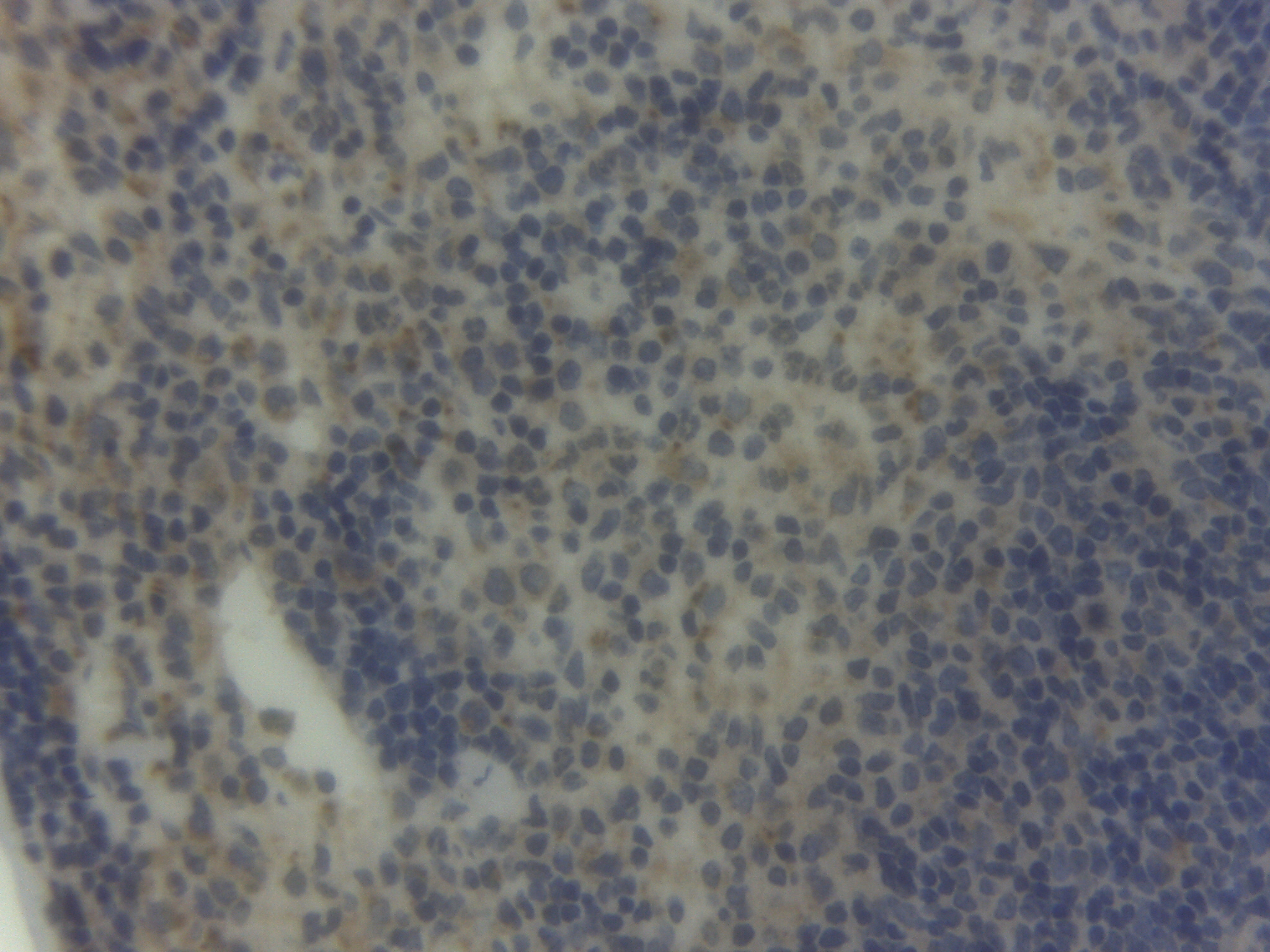

Supplement: S14 Fig — (ZIP) [file pone.0188960.s027.zip › NKp46 IHC image 24 hours/24h-6-4.jpg]

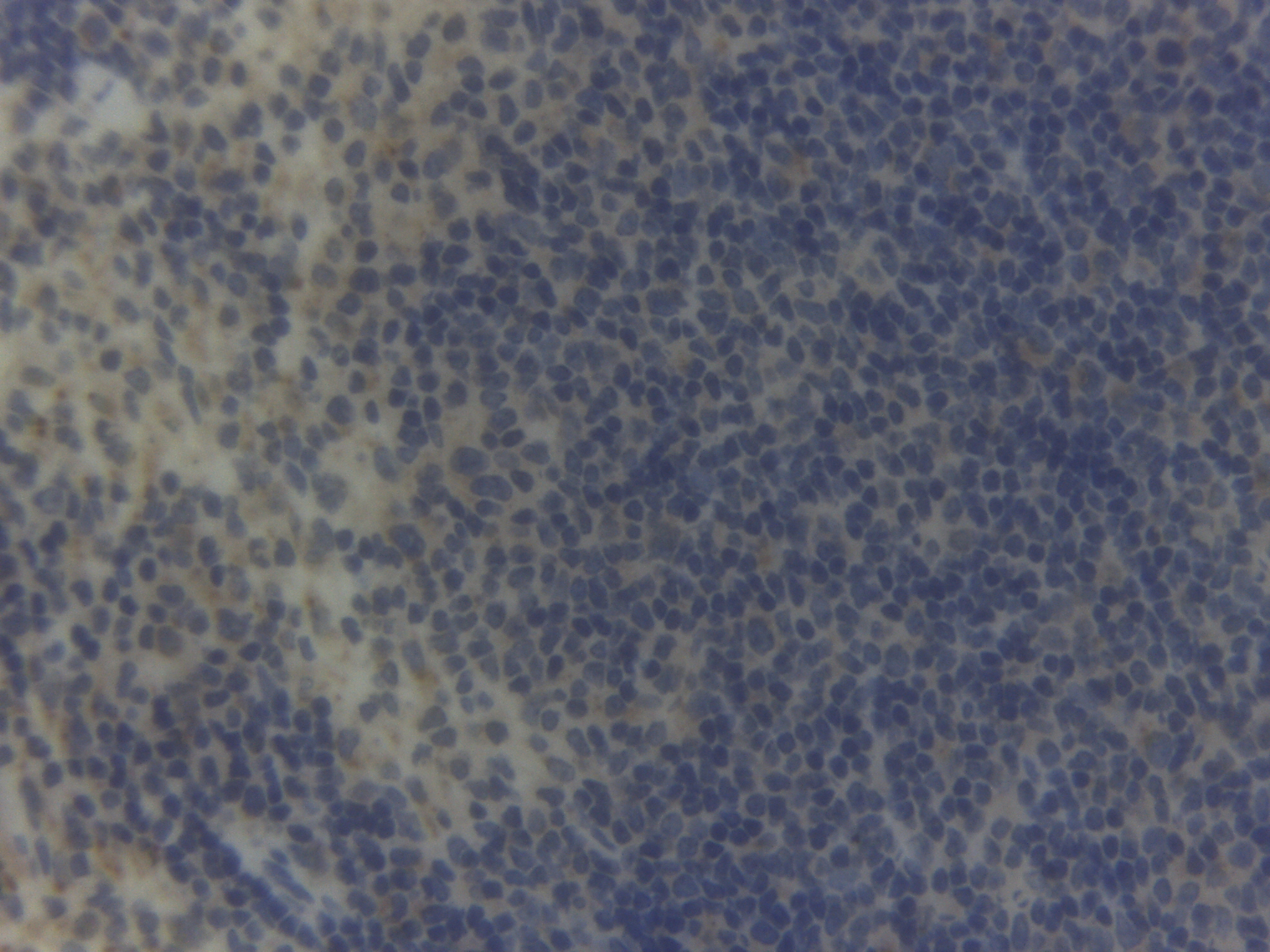

Supplement: S14 Fig — (ZIP) [file pone.0188960.s027.zip › NKp46 IHC image 24 hours/24h-6-5.jpg]

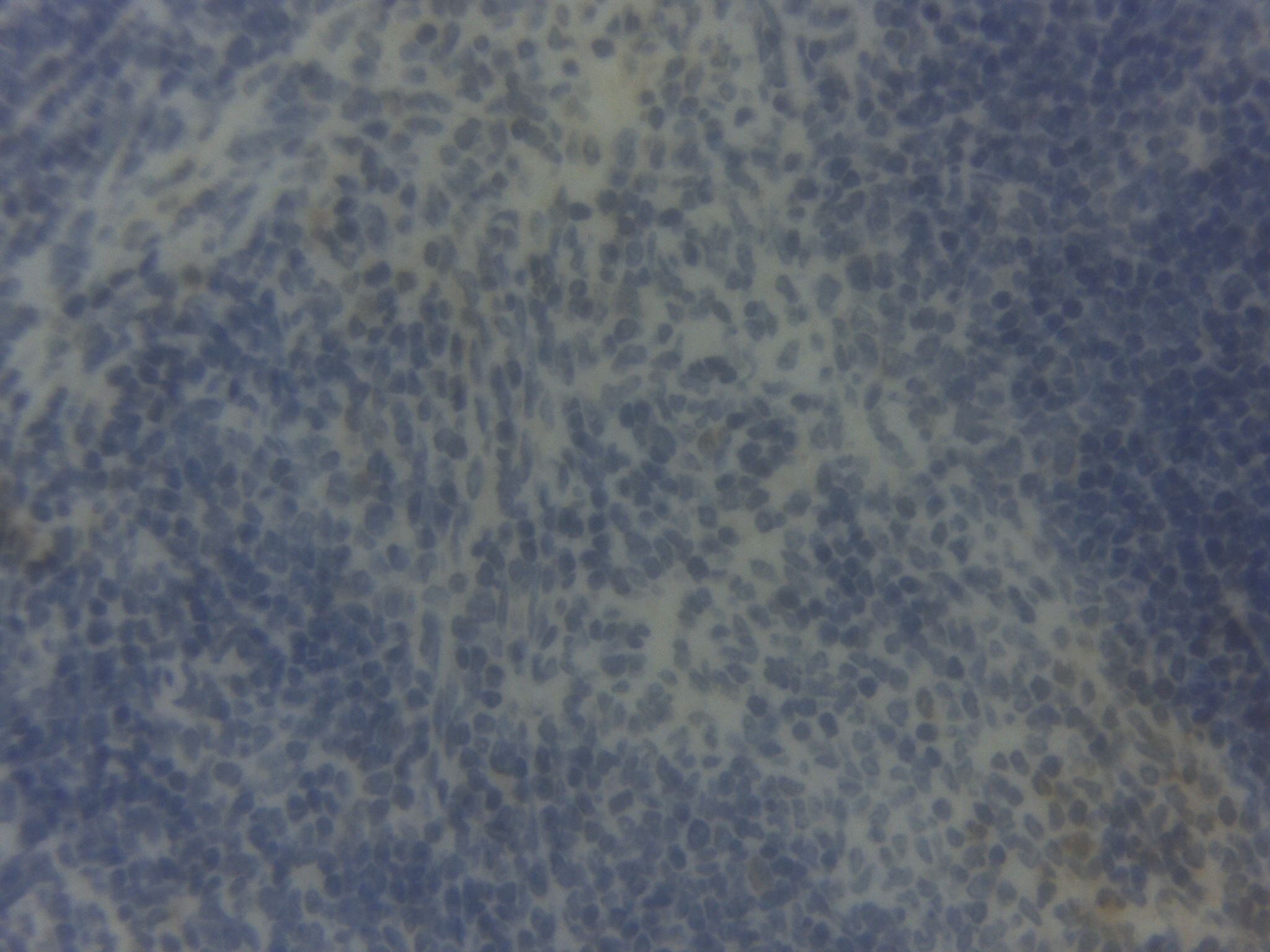

Supplement: S15 Fig — (ZIP) [file pone.0188960.s028.zip › NKp46 IHC image CON/con-1-1.jpg]

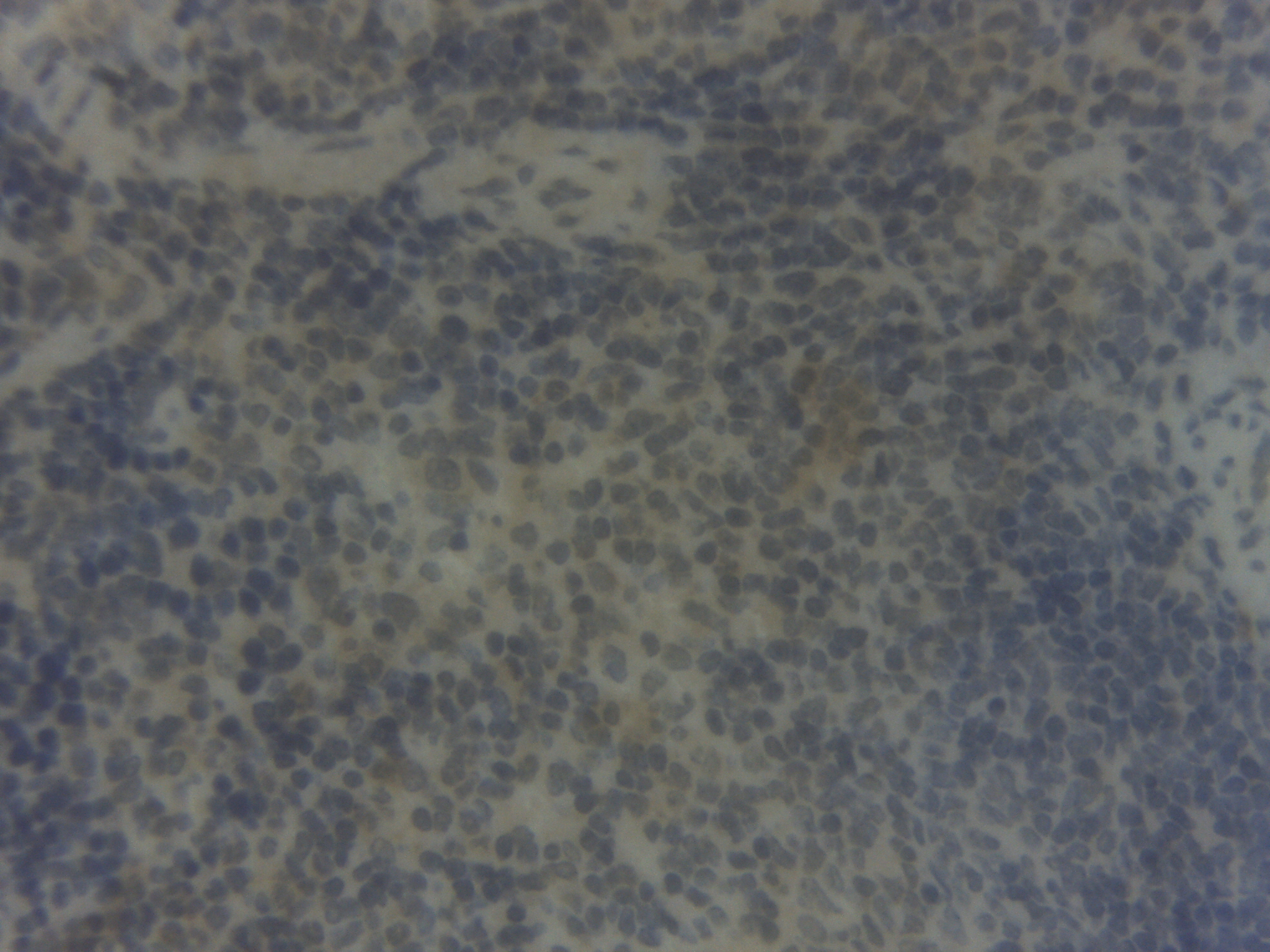

Supplement: S15 Fig — (ZIP) [file pone.0188960.s028.zip › NKp46 IHC image CON/con-1-2.jpg]

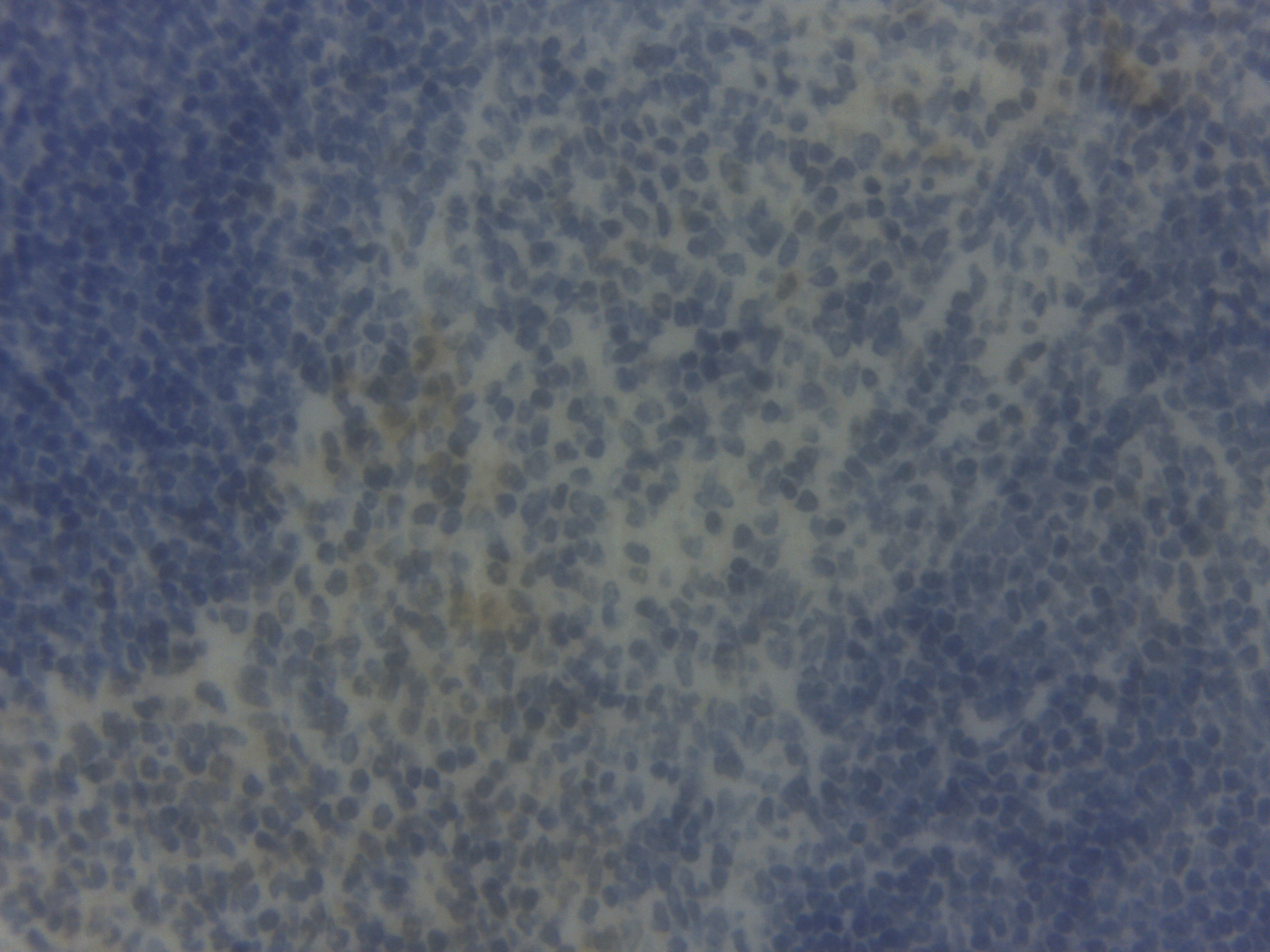

Supplement: S15 Fig — (ZIP) [file pone.0188960.s028.zip › NKp46 IHC image CON/con-1-3.jpg]

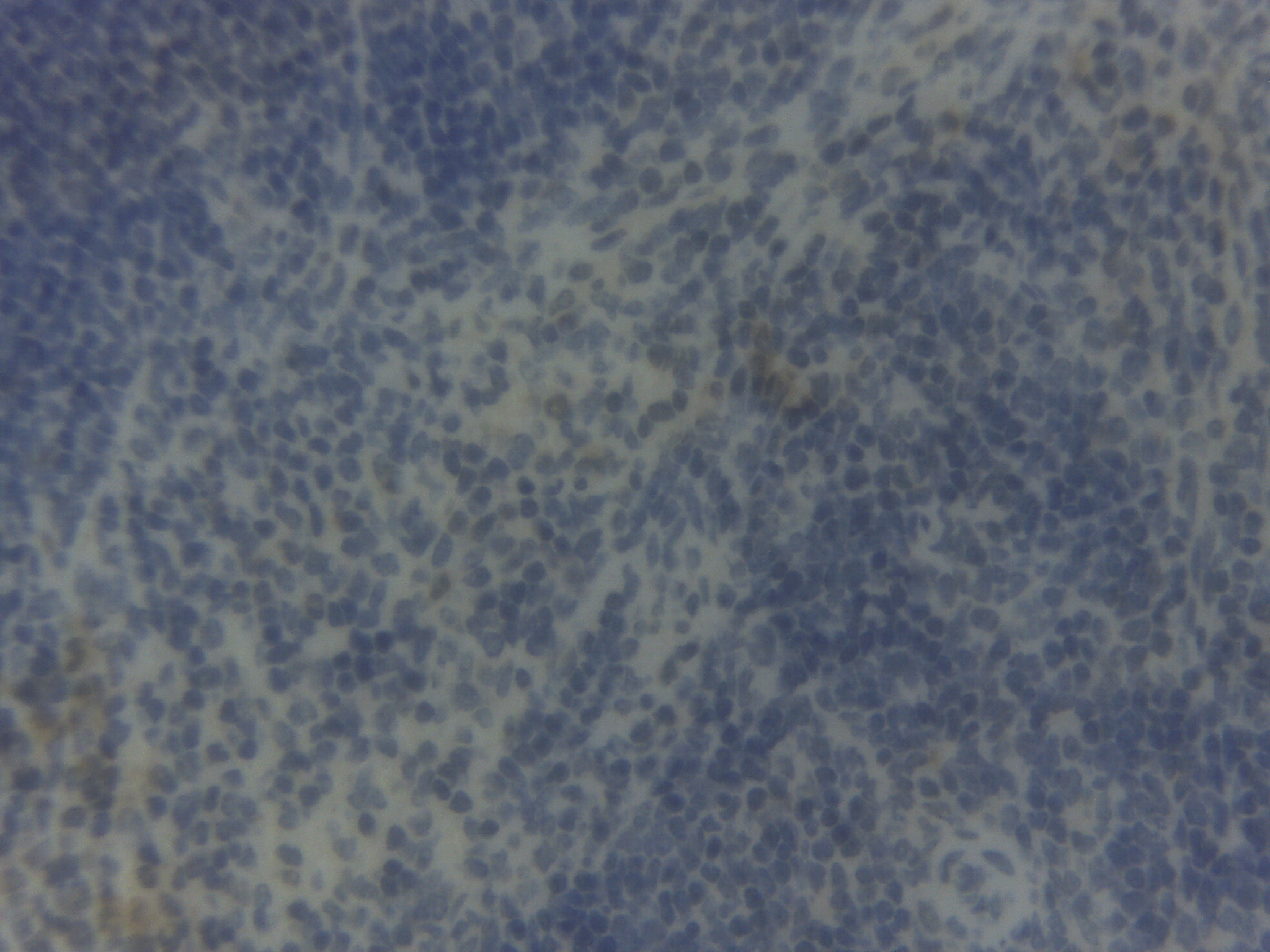

Supplement: S15 Fig — (ZIP) [file pone.0188960.s028.zip › NKp46 IHC image CON/con-1-4.jpg]

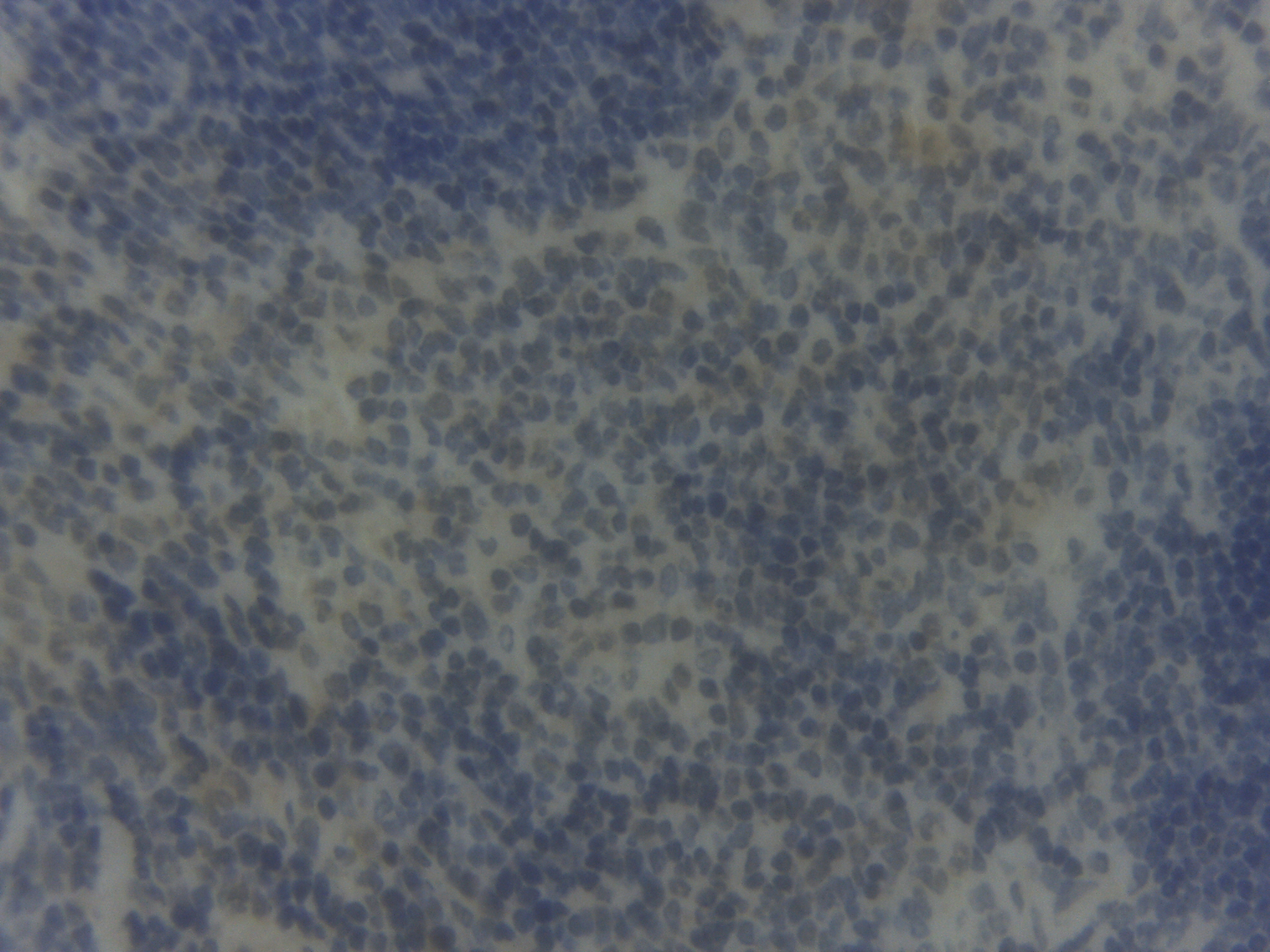

Supplement: S15 Fig — (ZIP) [file pone.0188960.s028.zip › NKp46 IHC image CON/con-1-5.jpg]

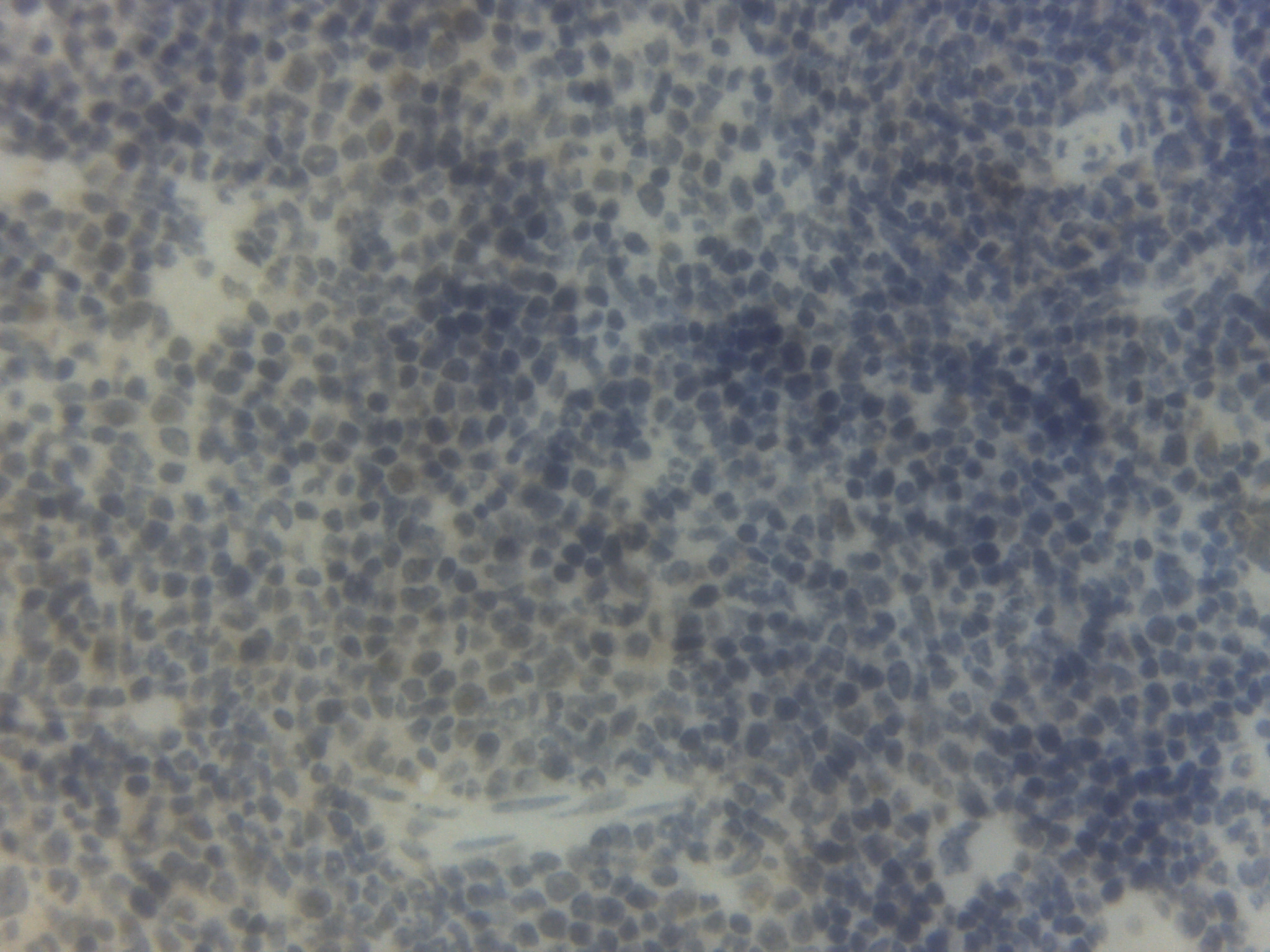

Supplement: S15 Fig — (ZIP) [file pone.0188960.s028.zip › NKp46 IHC image CON/con-2-1.jpg]

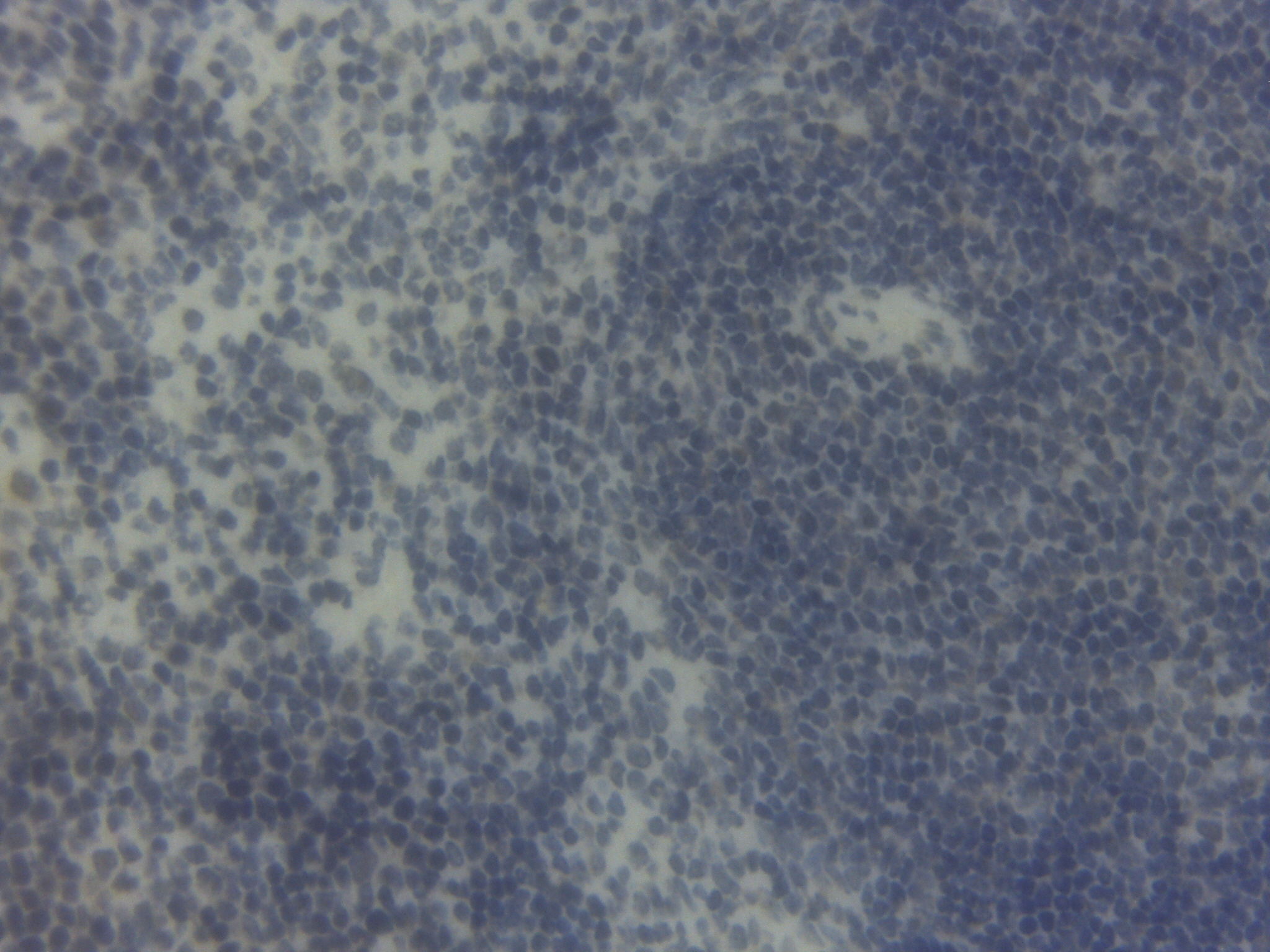

Supplement: S15 Fig — (ZIP) [file pone.0188960.s028.zip › NKp46 IHC image CON/con-2-2.jpg]

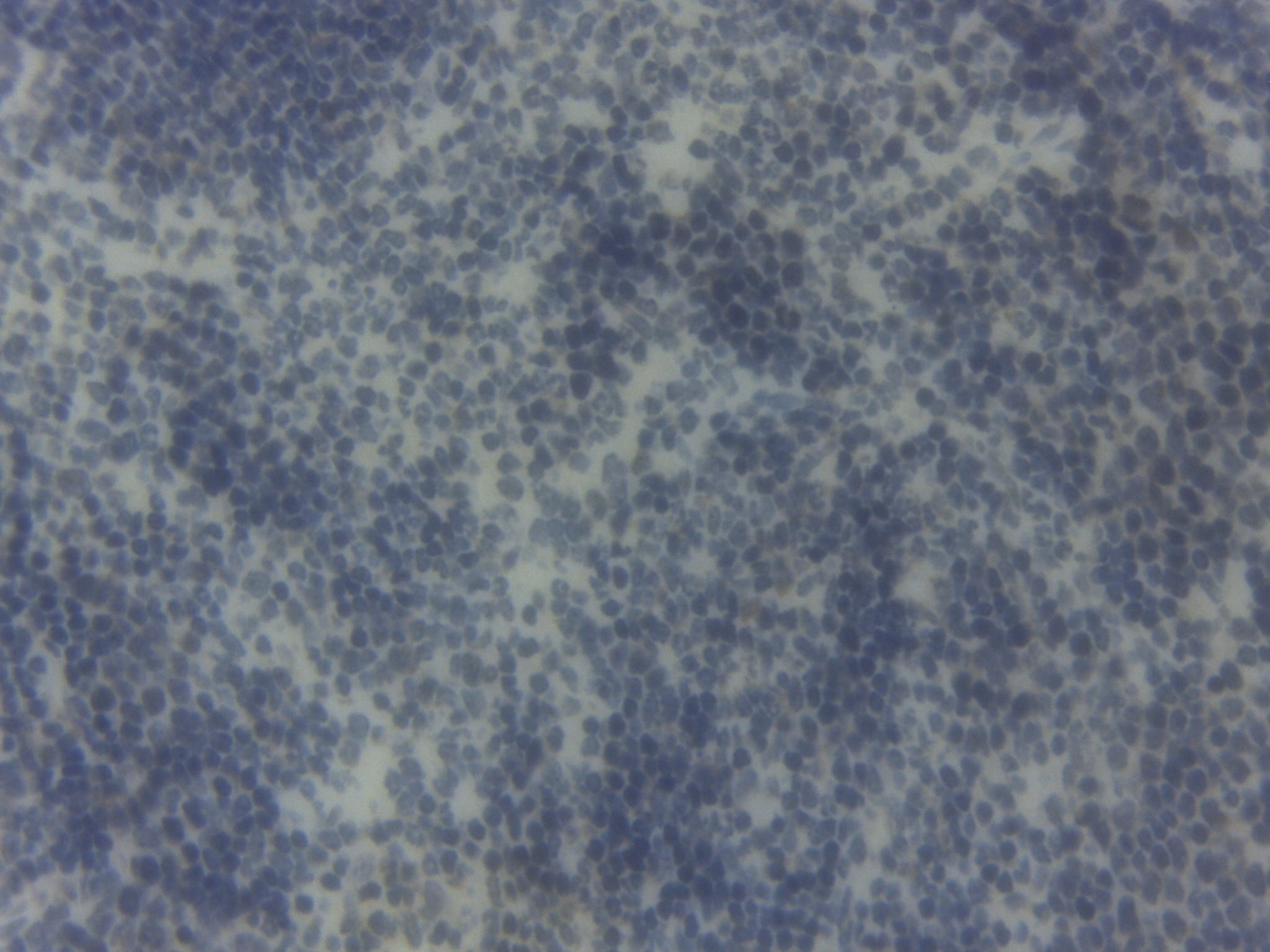

Supplement: S15 Fig — (ZIP) [file pone.0188960.s028.zip › NKp46 IHC image CON/con-2-3.jpg]

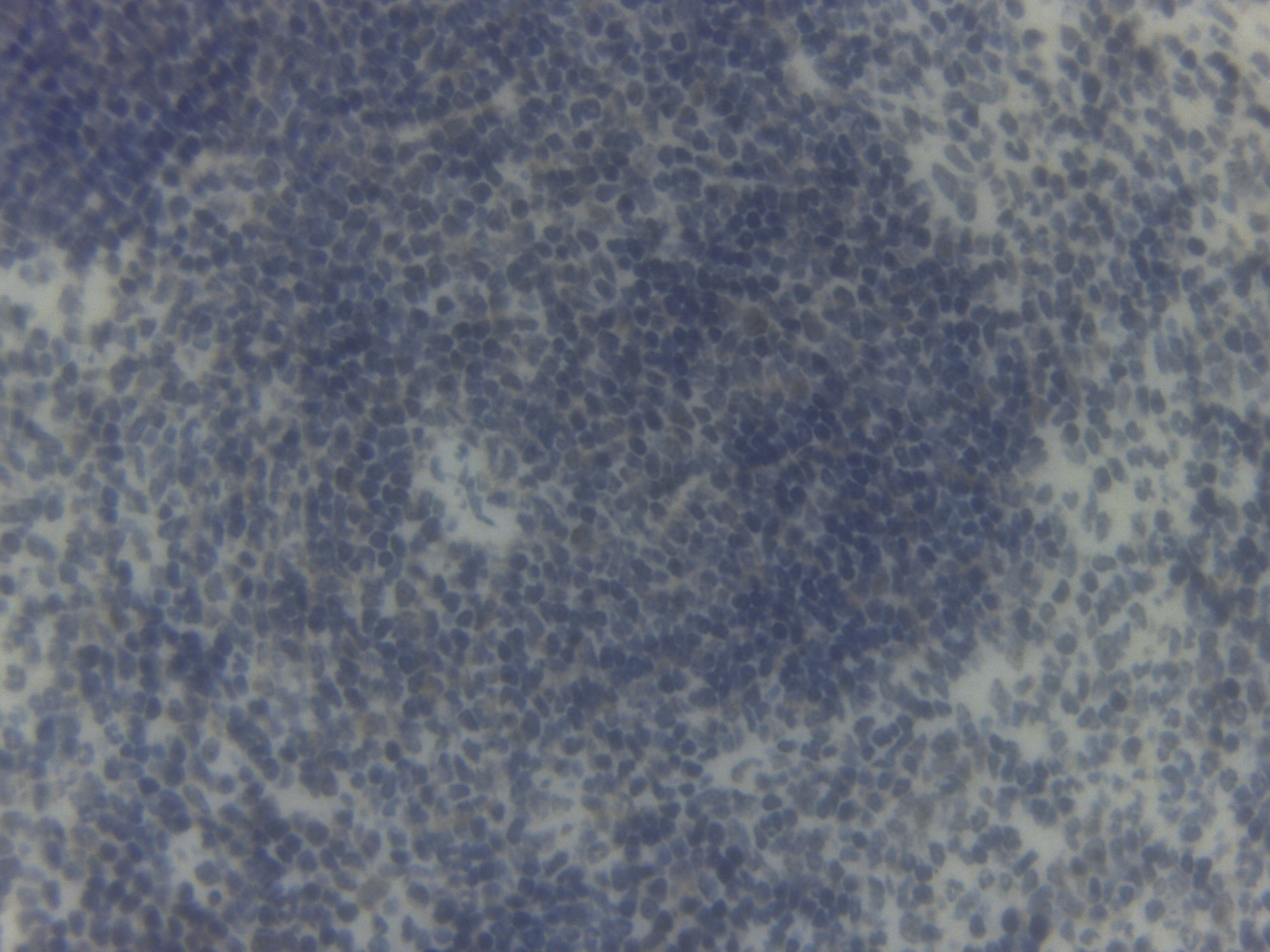

Supplement: S15 Fig — (ZIP) [file pone.0188960.s028.zip › NKp46 IHC image CON/con-2-4.jpg]

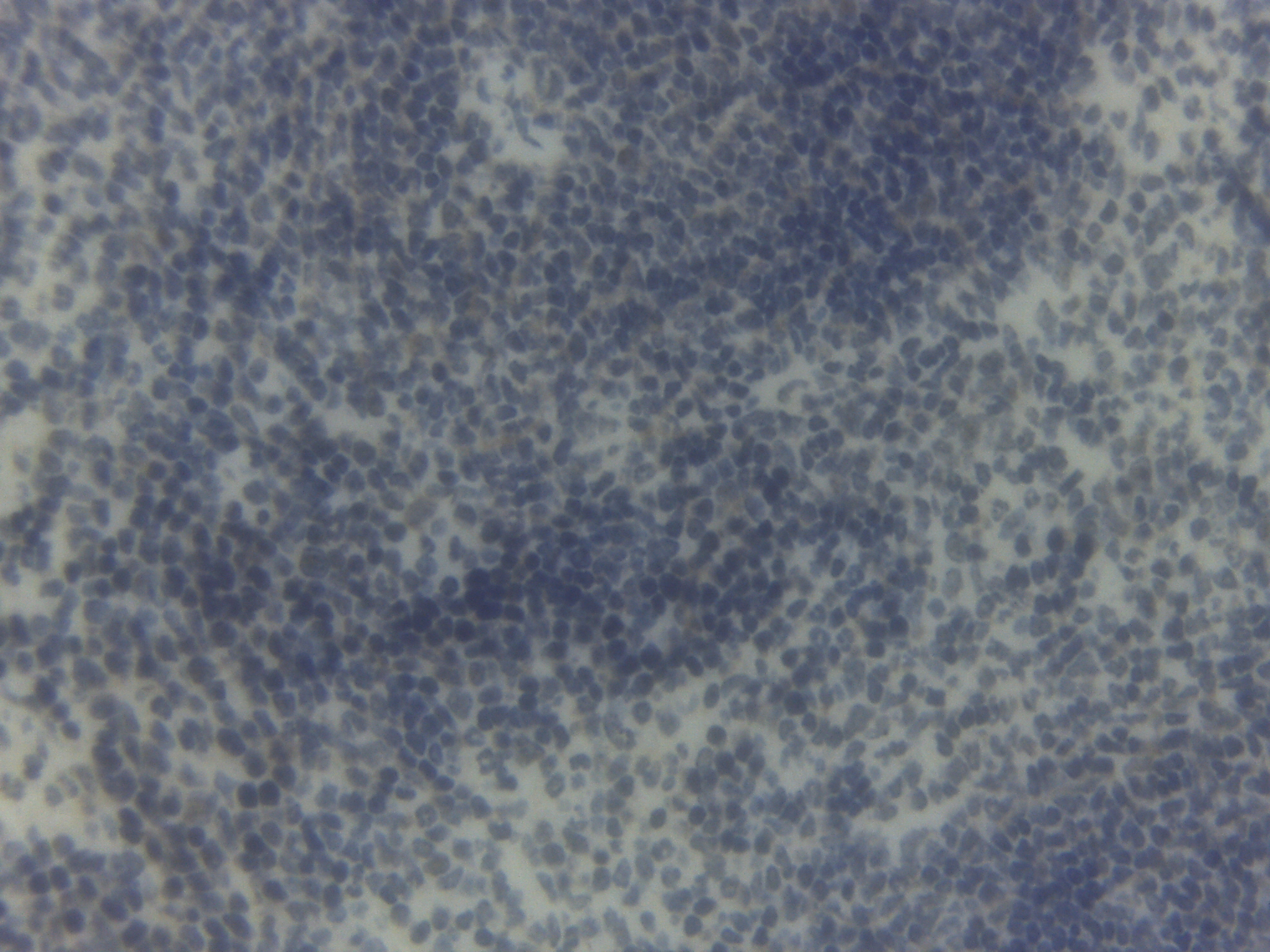

Supplement: S15 Fig — (ZIP) [file pone.0188960.s028.zip › NKp46 IHC image CON/con-2-5.jpg]

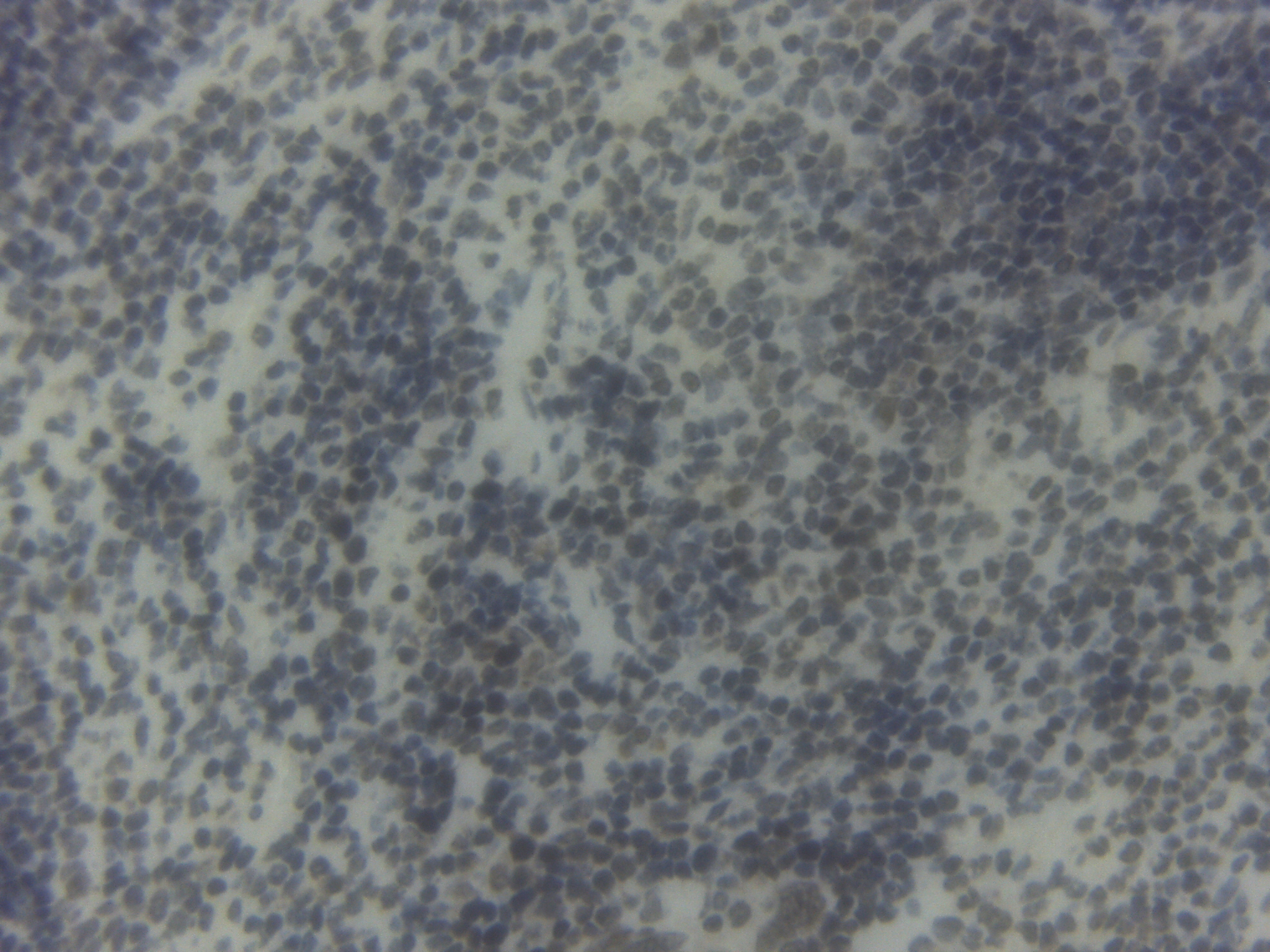

Supplement: S15 Fig — (ZIP) [file pone.0188960.s028.zip › NKp46 IHC image CON/con-3-1.jpg]

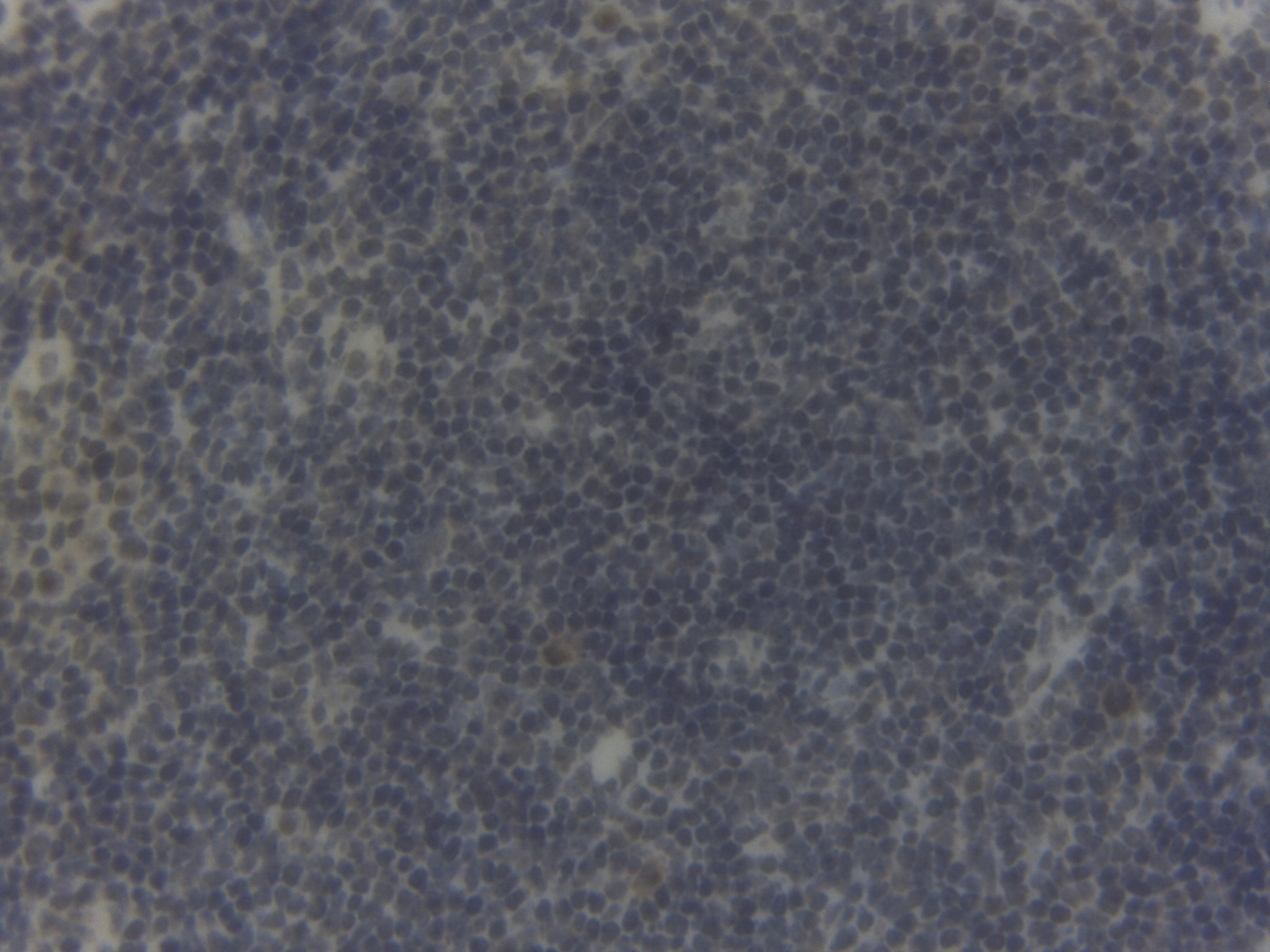

Supplement: S15 Fig — (ZIP) [file pone.0188960.s028.zip › NKp46 IHC image CON/con-3-2.jpg]

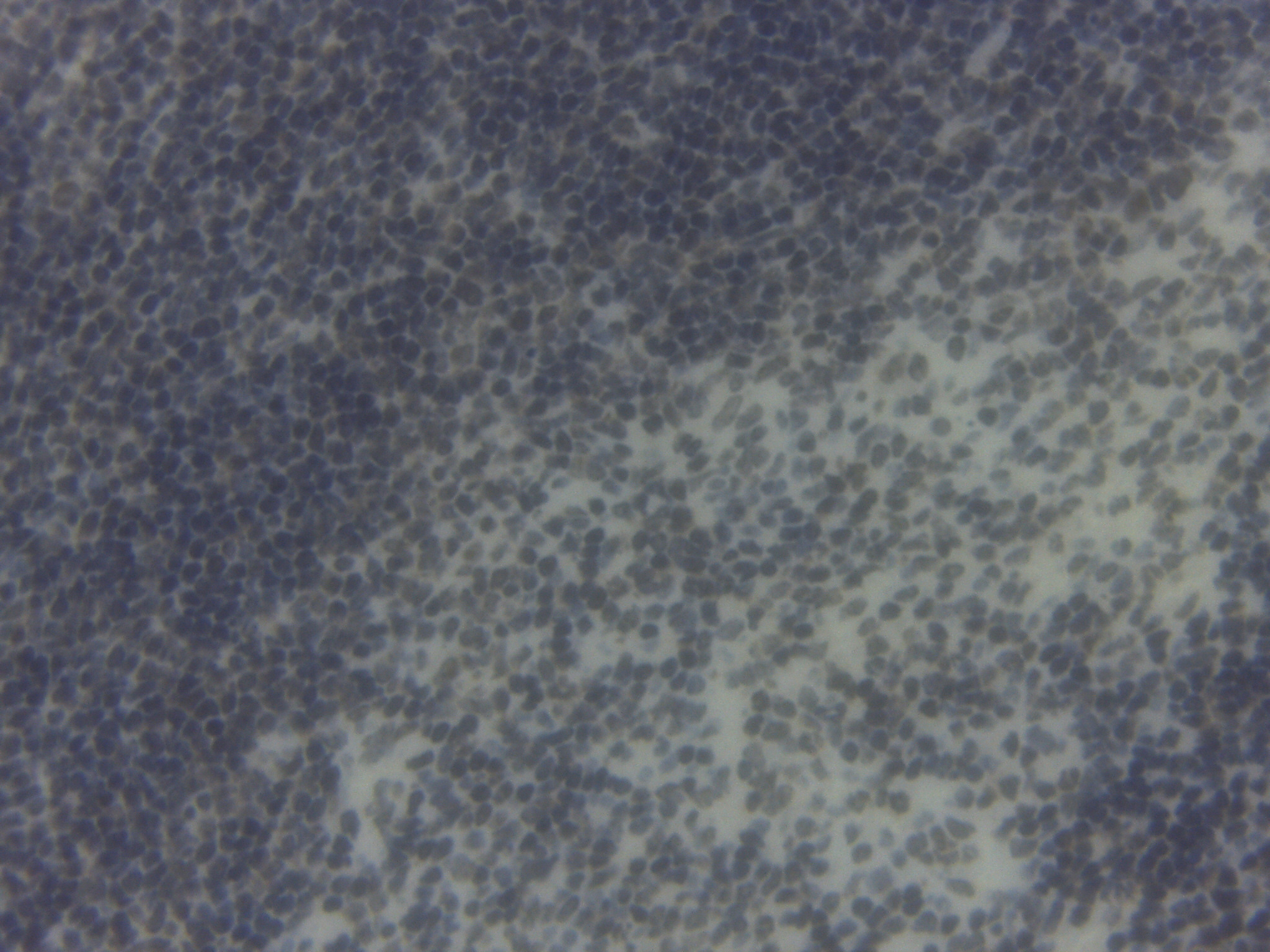

Supplement: S15 Fig — (ZIP) [file pone.0188960.s028.zip › NKp46 IHC image CON/con-3-3.jpg]
