# Supplementary figures and images for: Proteus mirabilis inhibits cancer growth and pulmonary metastasis in a mouse breast cancer model (part 5 of 5)
Source: PLoS One. 2017 Dec 5;12(12):e0188960. doi: 10.1371/journal.pone.0188960 (PMC5716547; doi:10.1371/journal.pone.0188960)

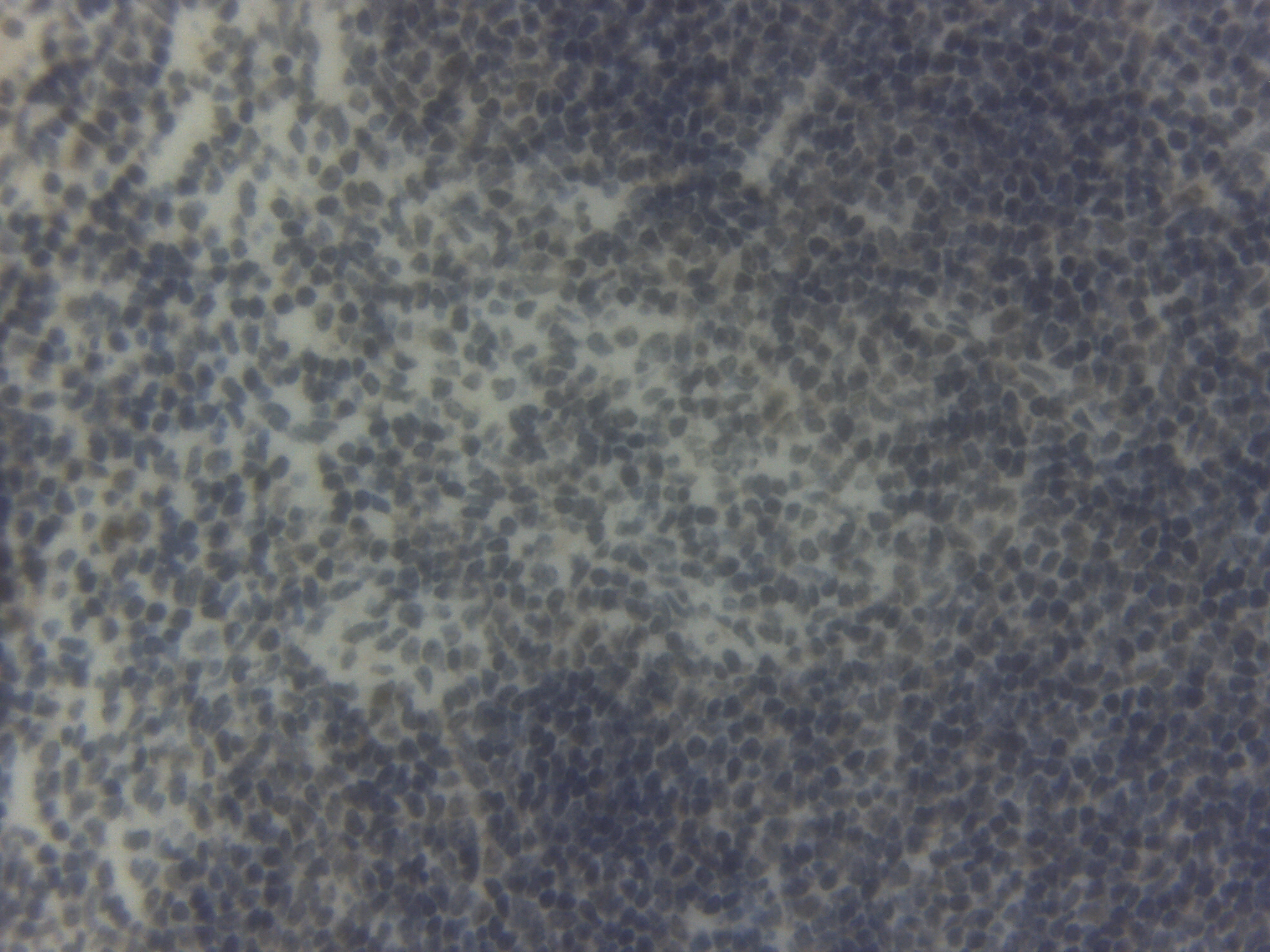

Supplement: S15 Fig — (ZIP) [file pone.0188960.s028.zip › NKp46 IHC image CON/con-3-4.jpg]

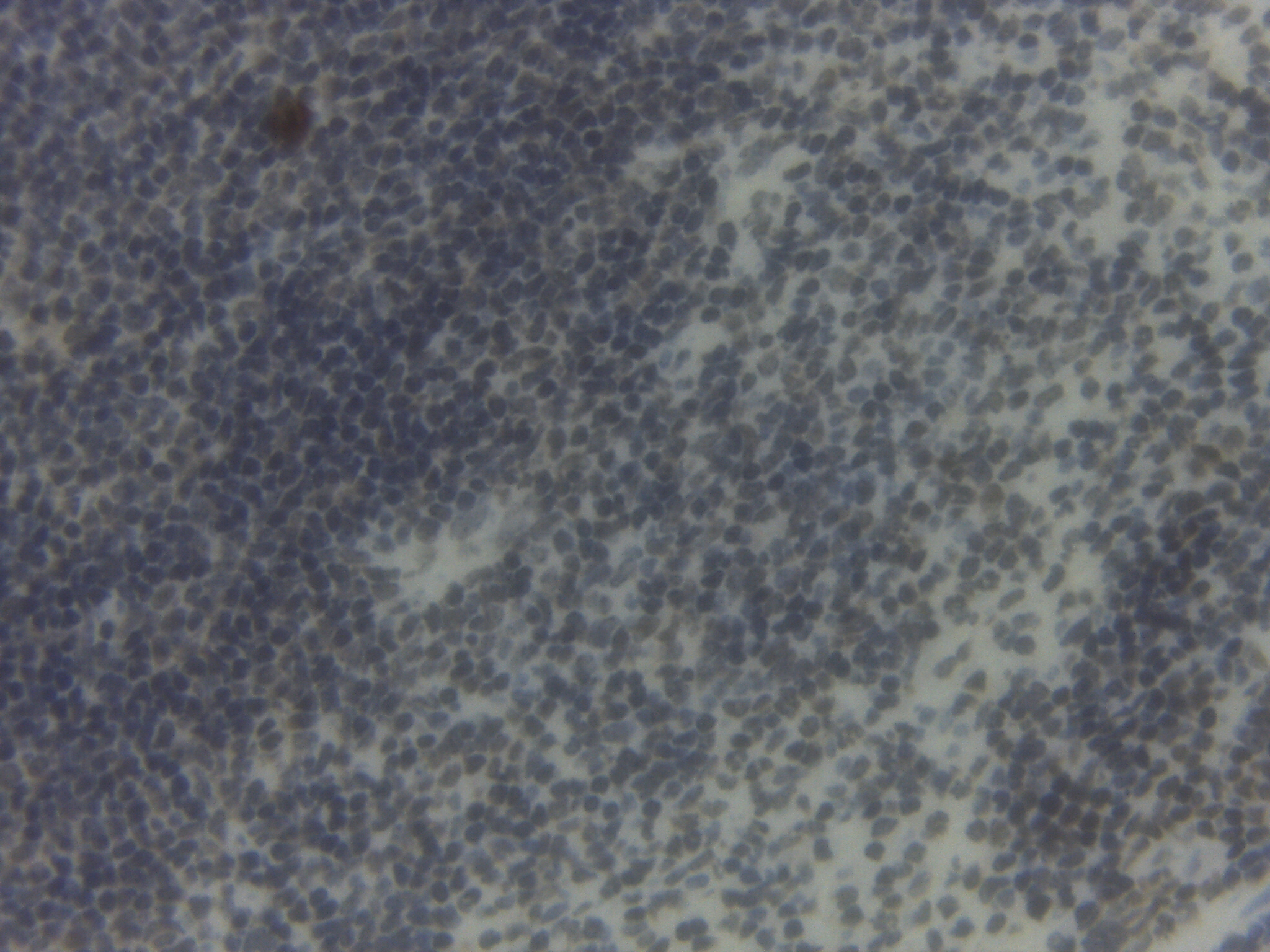

Supplement: S15 Fig — (ZIP) [file pone.0188960.s028.zip › NKp46 IHC image CON/con-3-5.jpg]

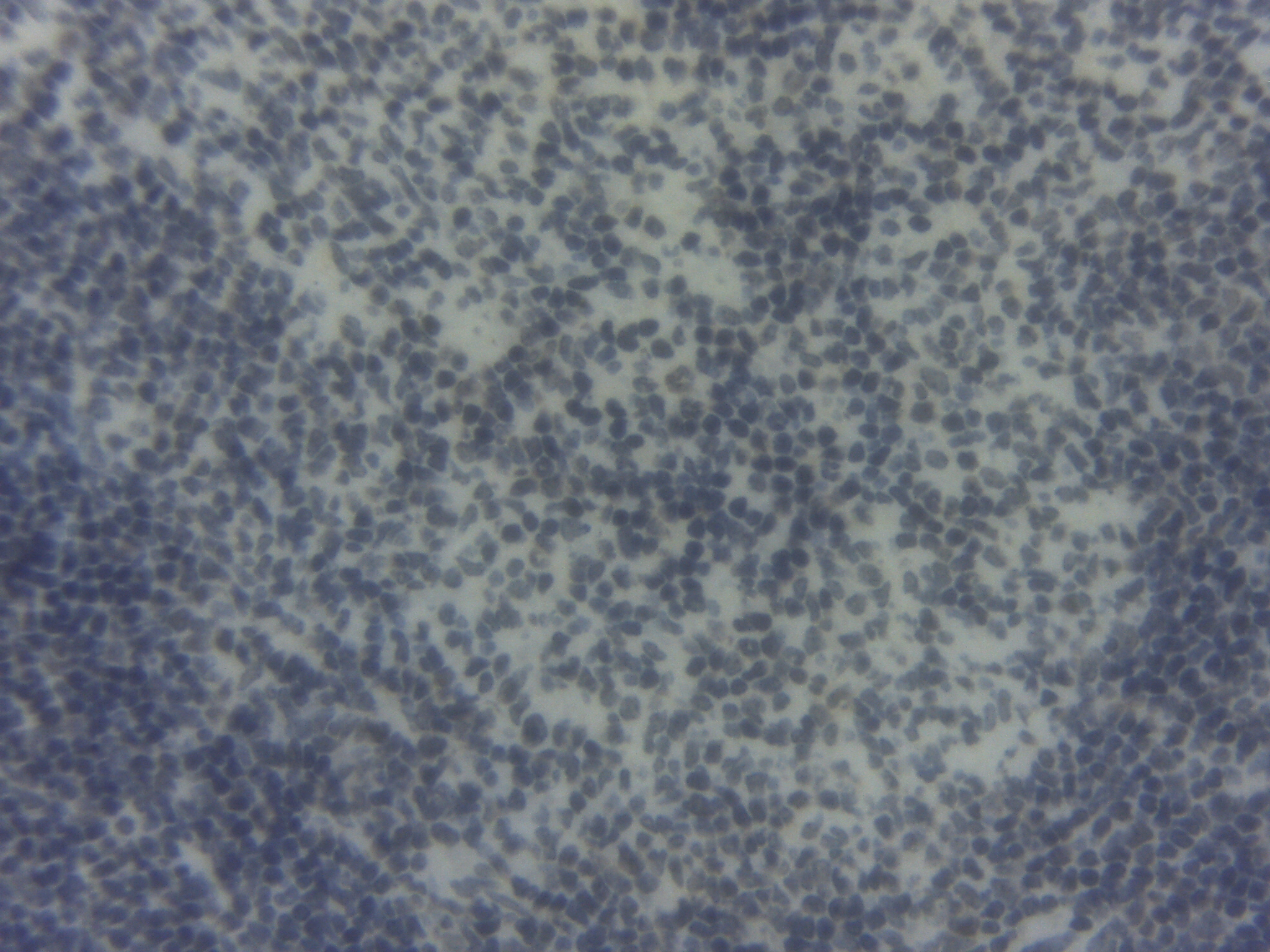

Supplement: S15 Fig — (ZIP) [file pone.0188960.s028.zip › NKp46 IHC image CON/con-4-1.jpg]

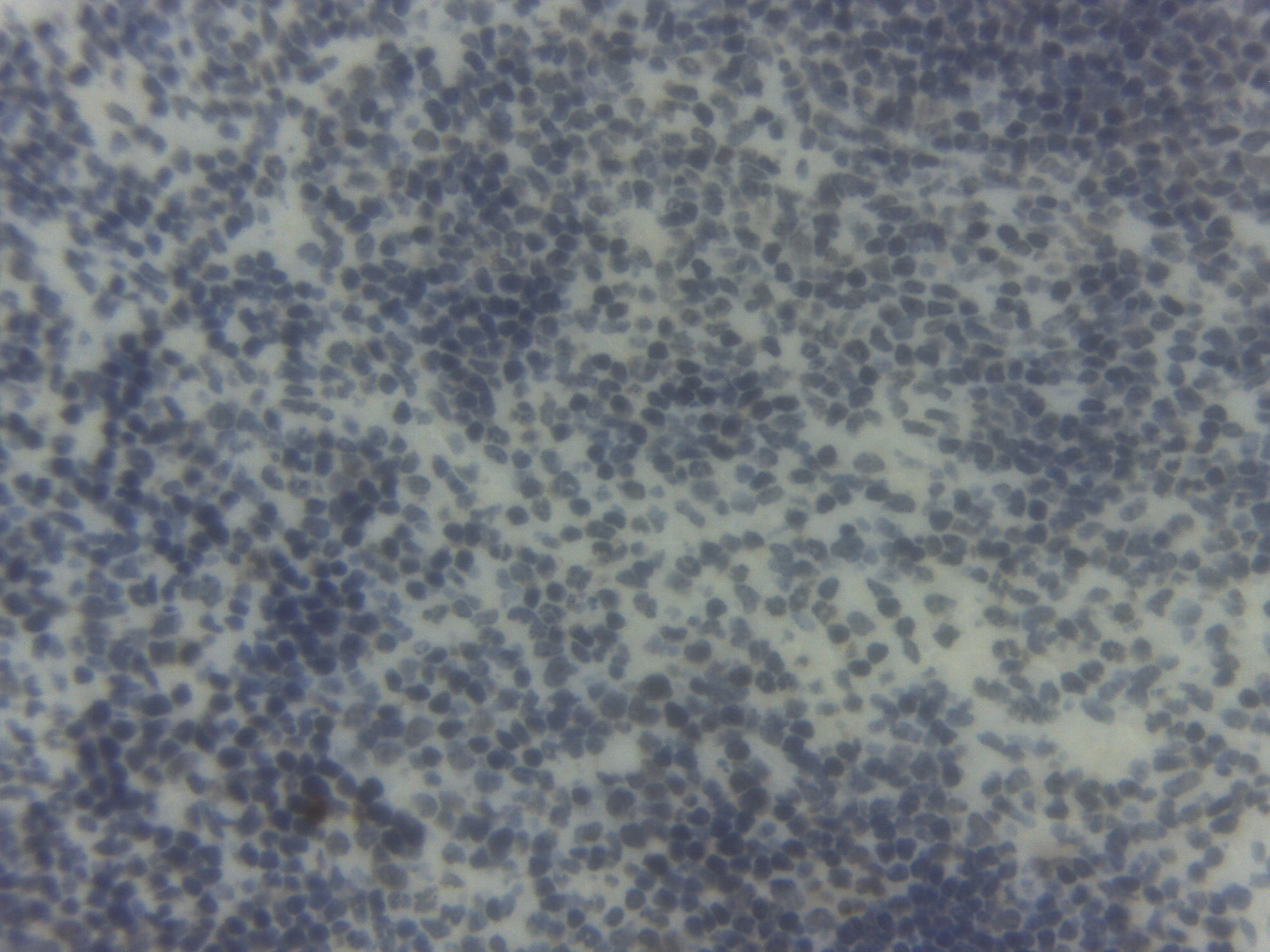

Supplement: S15 Fig — (ZIP) [file pone.0188960.s028.zip › NKp46 IHC image CON/con-4-2.jpg]

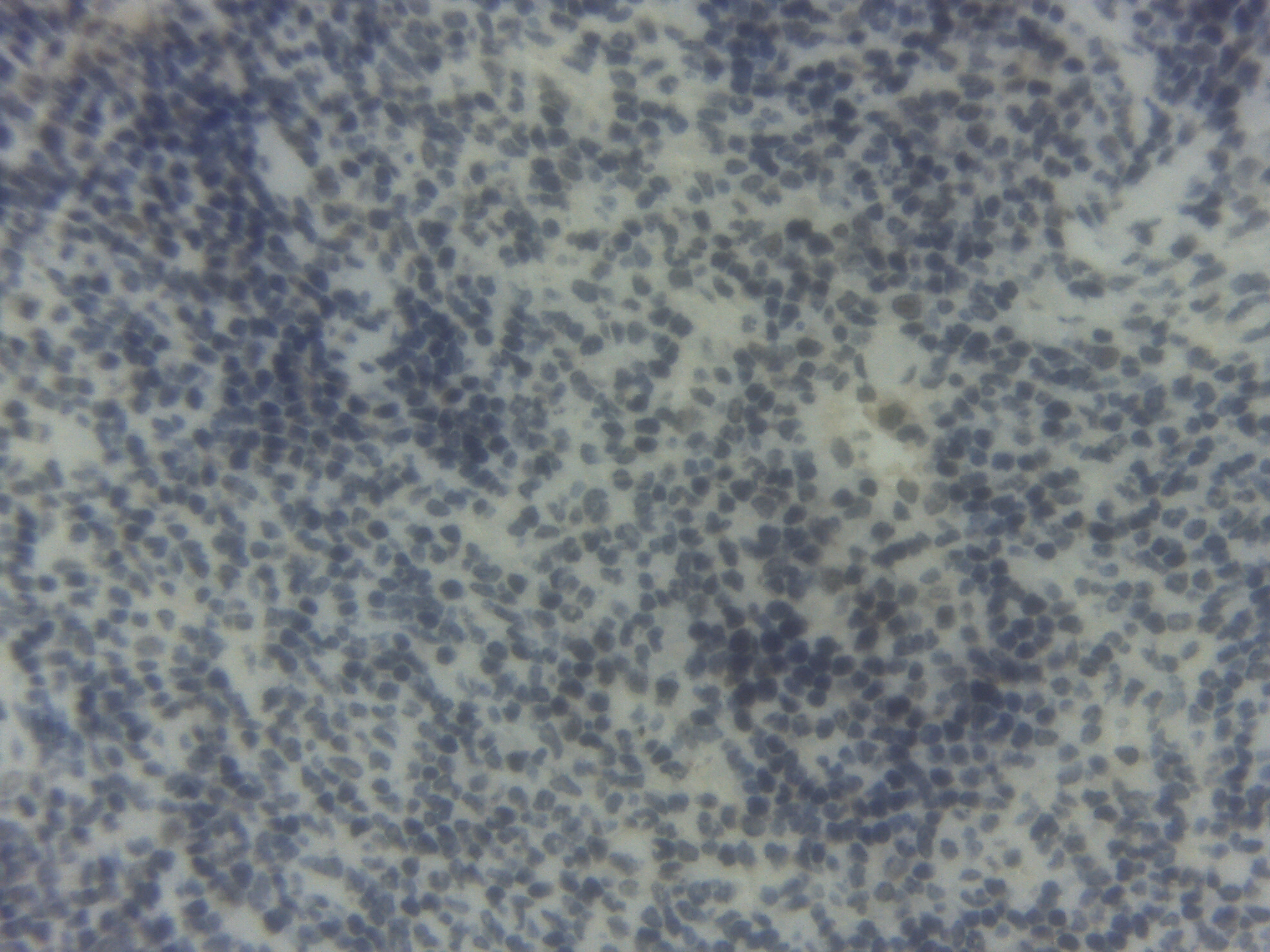

Supplement: S15 Fig — (ZIP) [file pone.0188960.s028.zip › NKp46 IHC image CON/con-4-3.jpg]

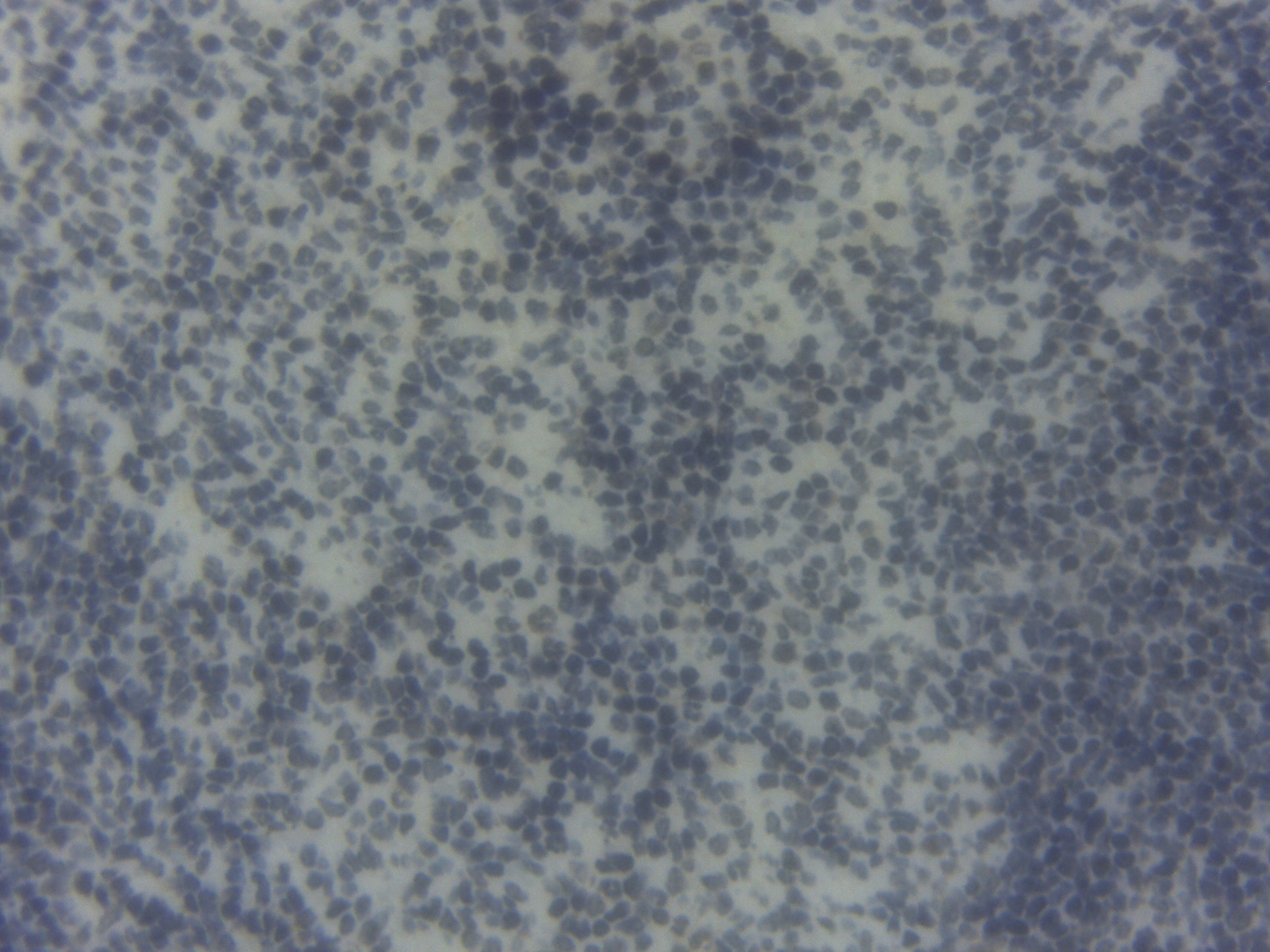

Supplement: S15 Fig — (ZIP) [file pone.0188960.s028.zip › NKp46 IHC image CON/con-4-4.jpg]

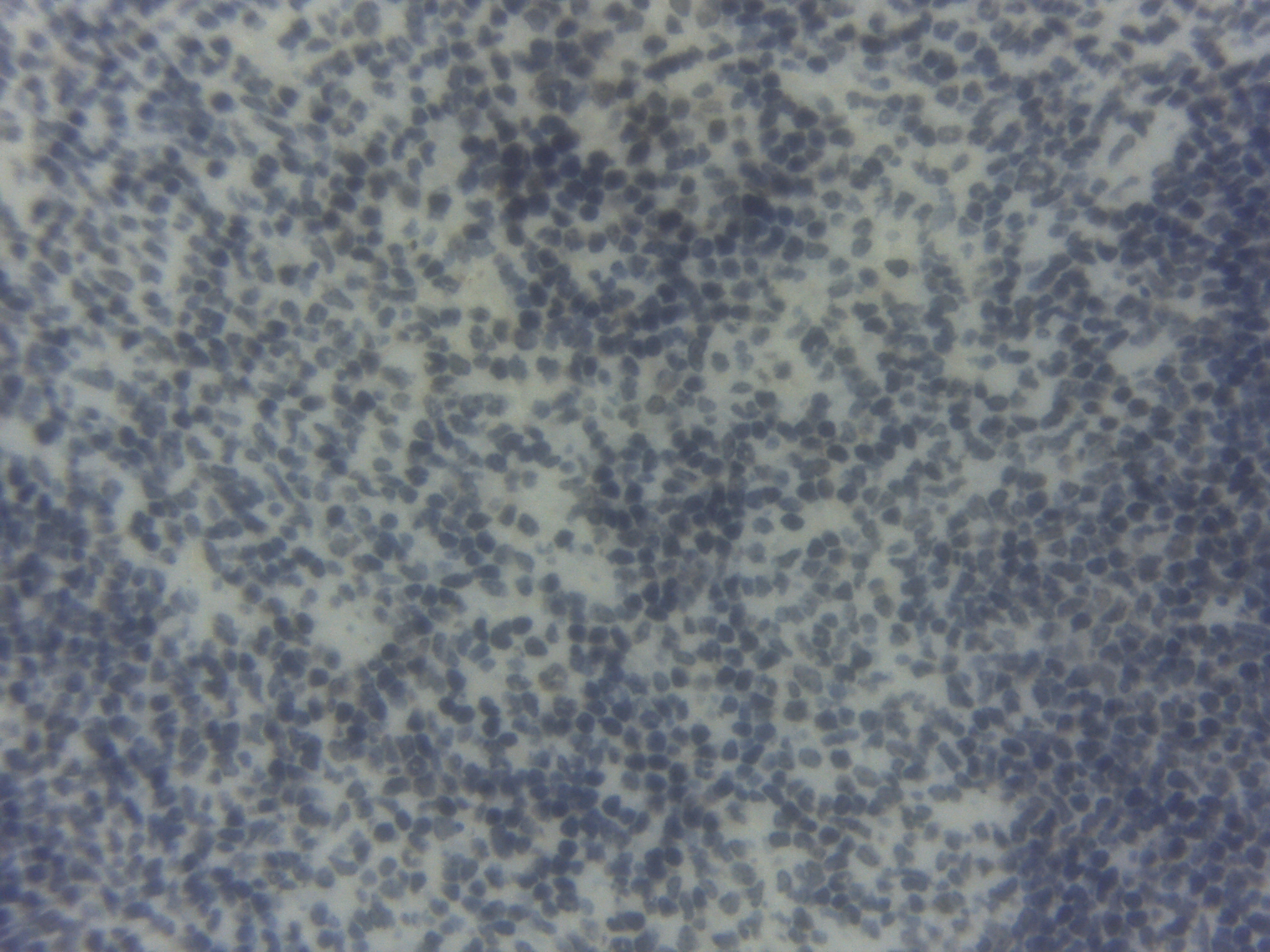

Supplement: S15 Fig — (ZIP) [file pone.0188960.s028.zip › NKp46 IHC image CON/con-4-5.jpg]

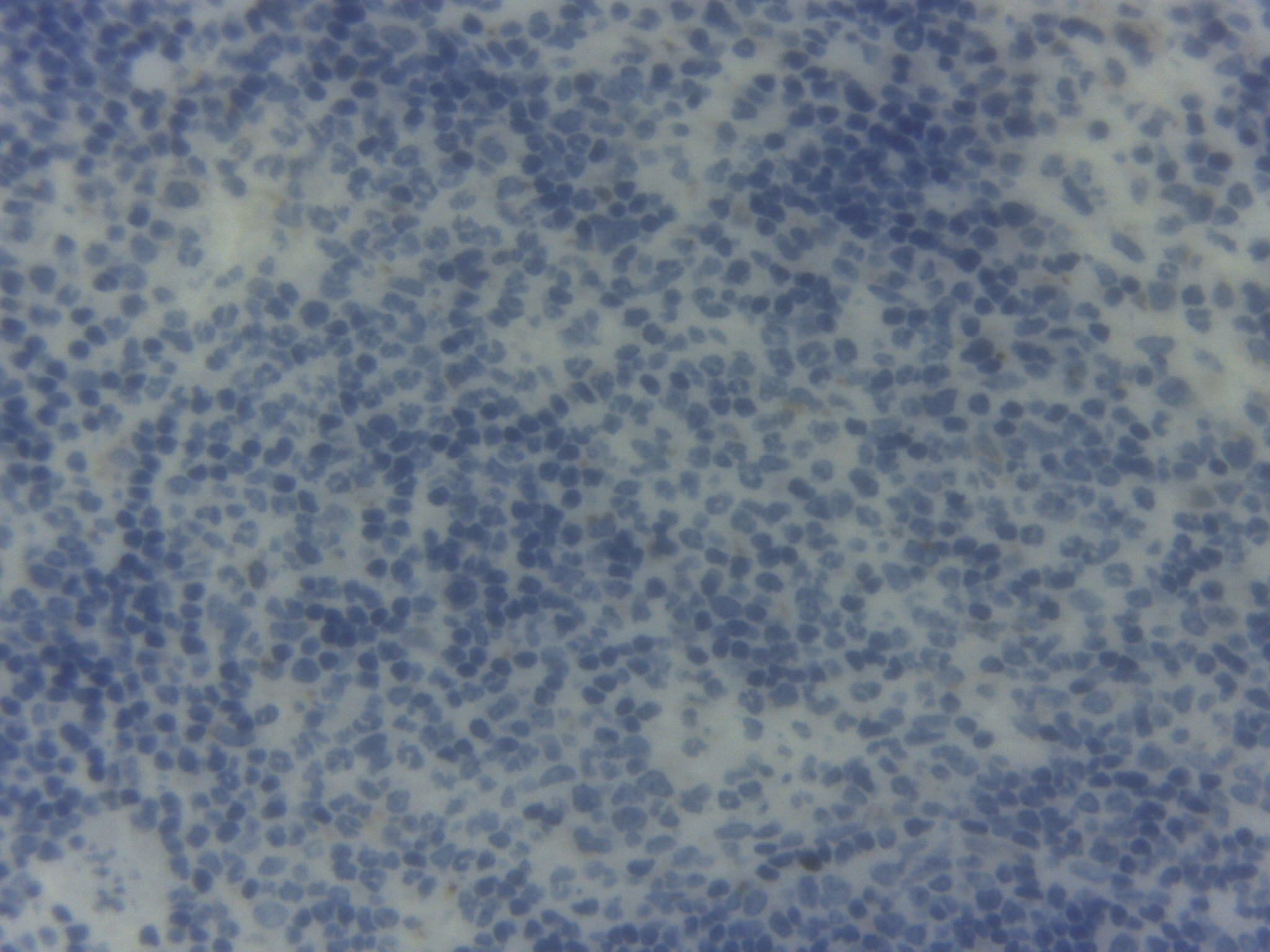

Supplement: S15 Fig — (ZIP) [file pone.0188960.s028.zip › NKp46 IHC image CON/con-5-1.jpg]

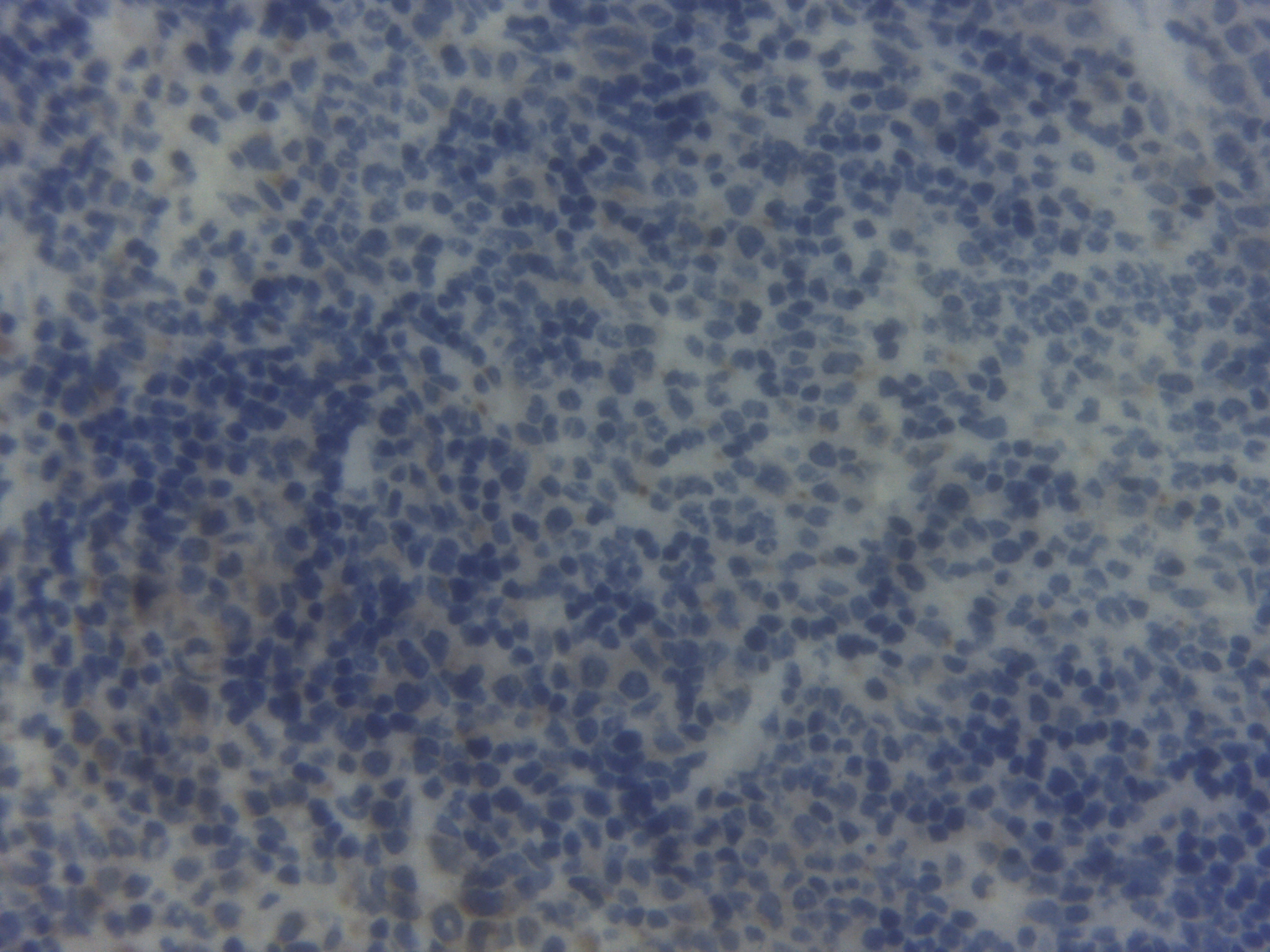

Supplement: S15 Fig — (ZIP) [file pone.0188960.s028.zip › NKp46 IHC image CON/con-5-2.jpg]

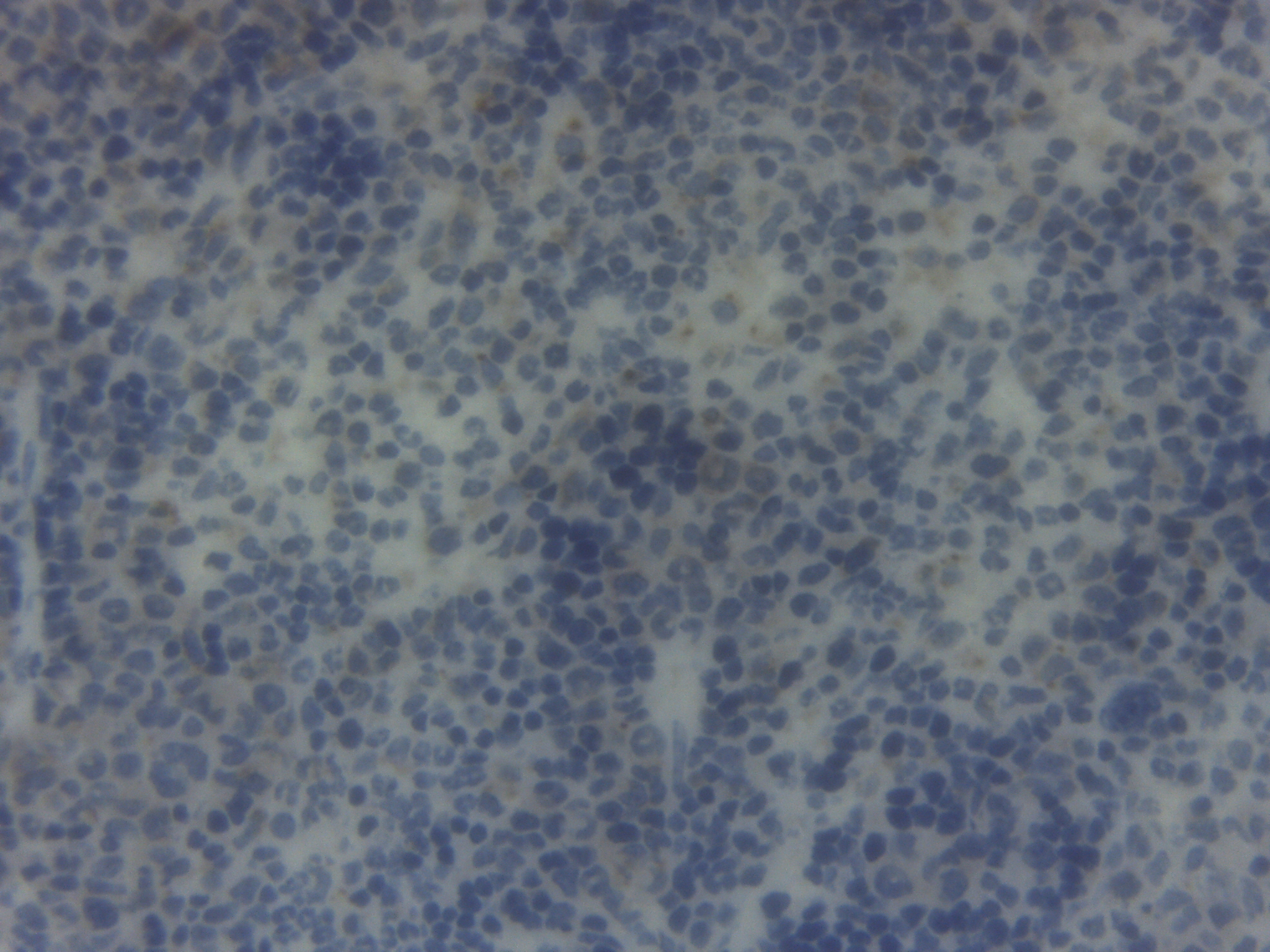

Supplement: S15 Fig — (ZIP) [file pone.0188960.s028.zip › NKp46 IHC image CON/con-5-3.jpg]

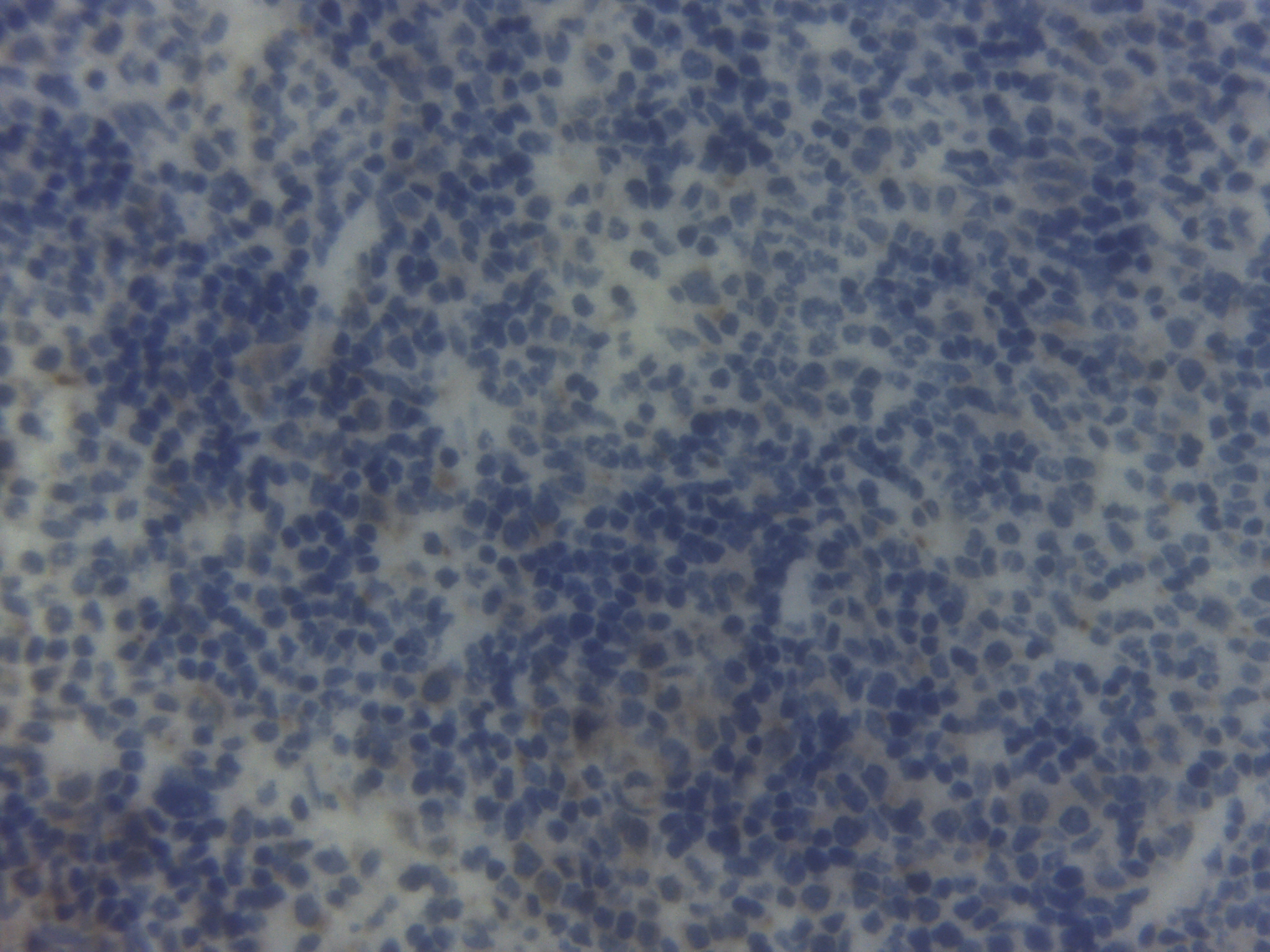

Supplement: S15 Fig — (ZIP) [file pone.0188960.s028.zip › NKp46 IHC image CON/con-5-4.jpg]

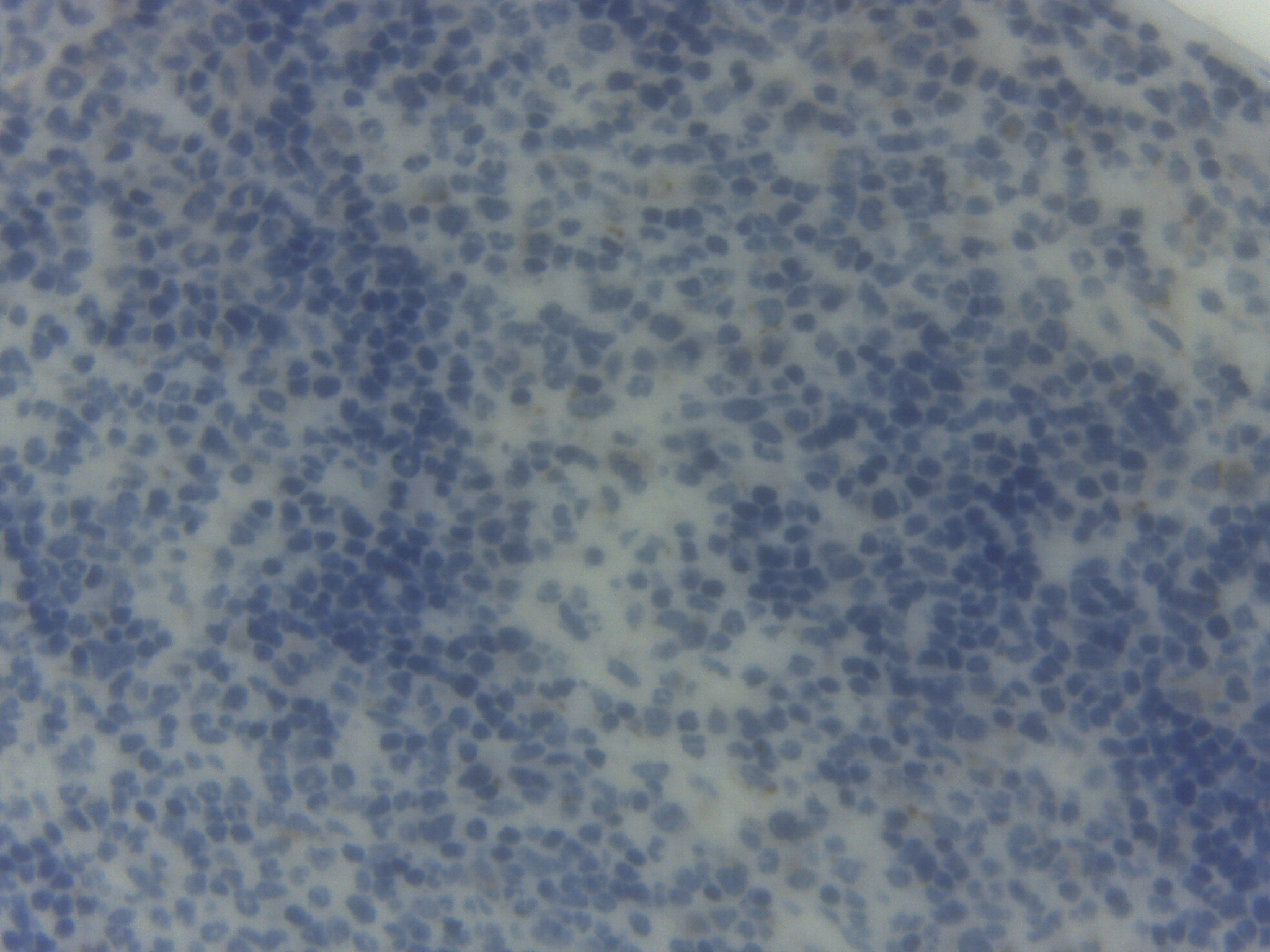

Supplement: S15 Fig — (ZIP) [file pone.0188960.s028.zip › NKp46 IHC image CON/con-5-5.jpg]

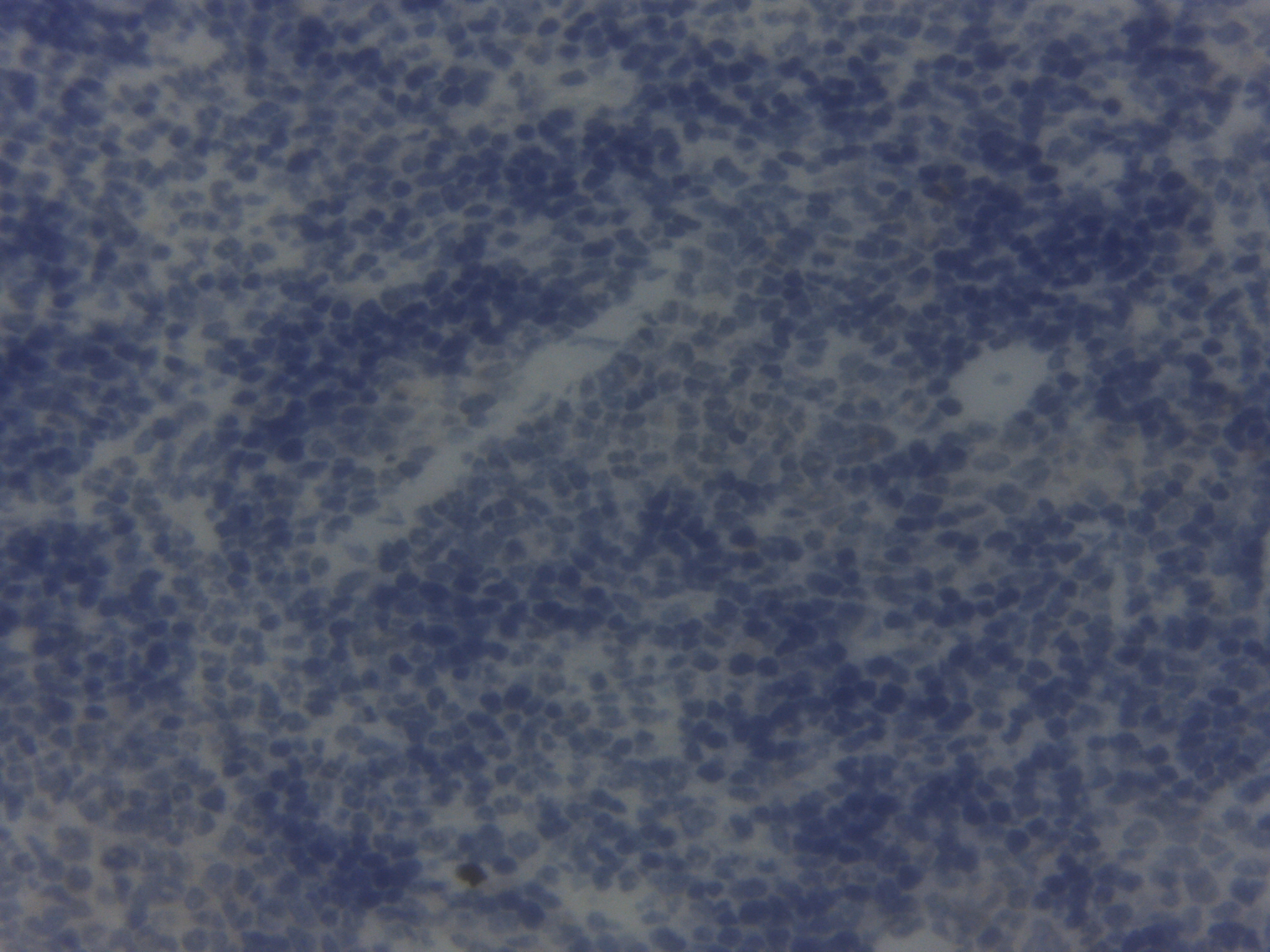

Supplement: S15 Fig — (ZIP) [file pone.0188960.s028.zip › NKp46 IHC image CON/con-6-1.jpg]

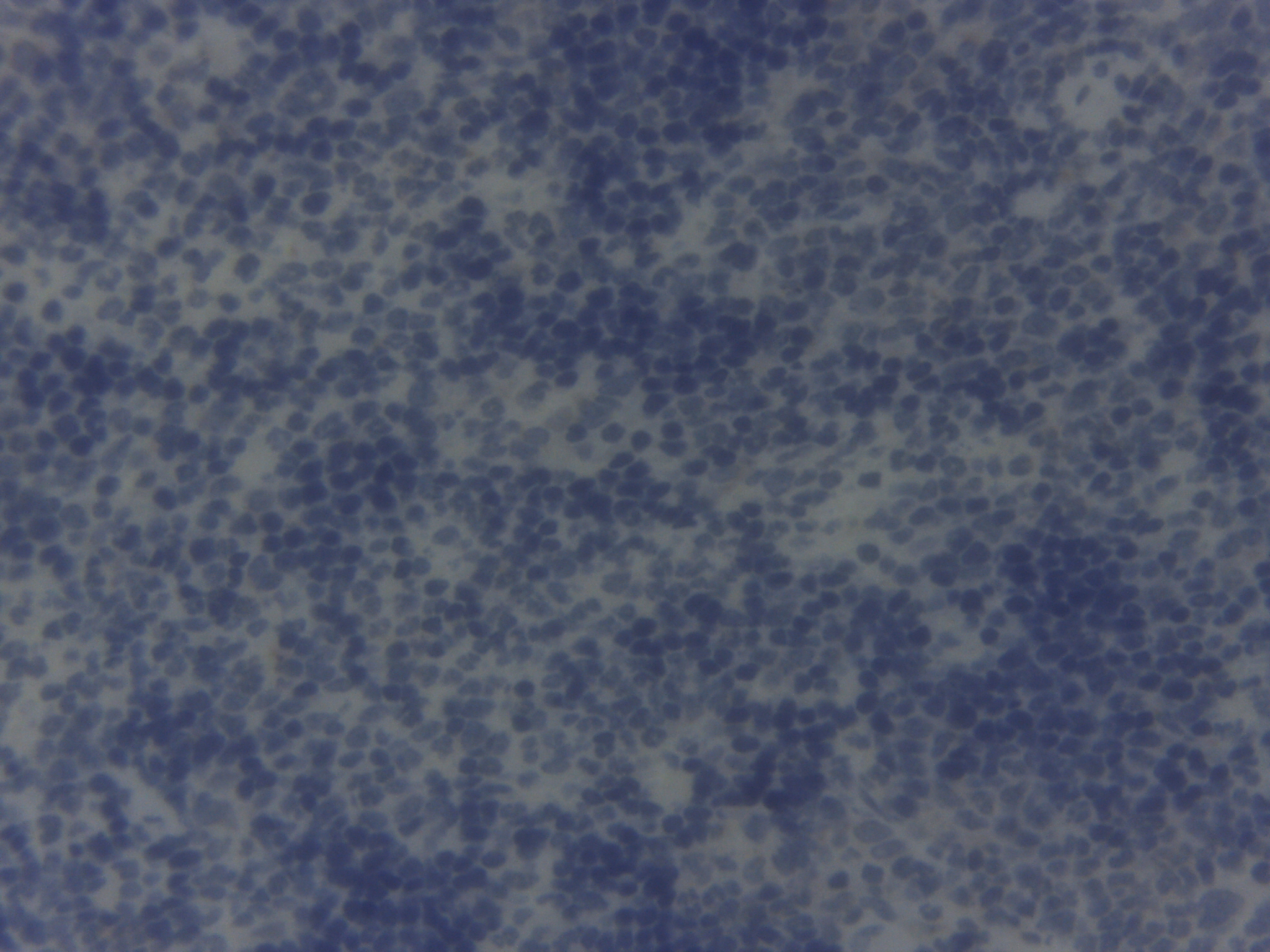

Supplement: S15 Fig — (ZIP) [file pone.0188960.s028.zip › NKp46 IHC image CON/con-6-2.jpg]

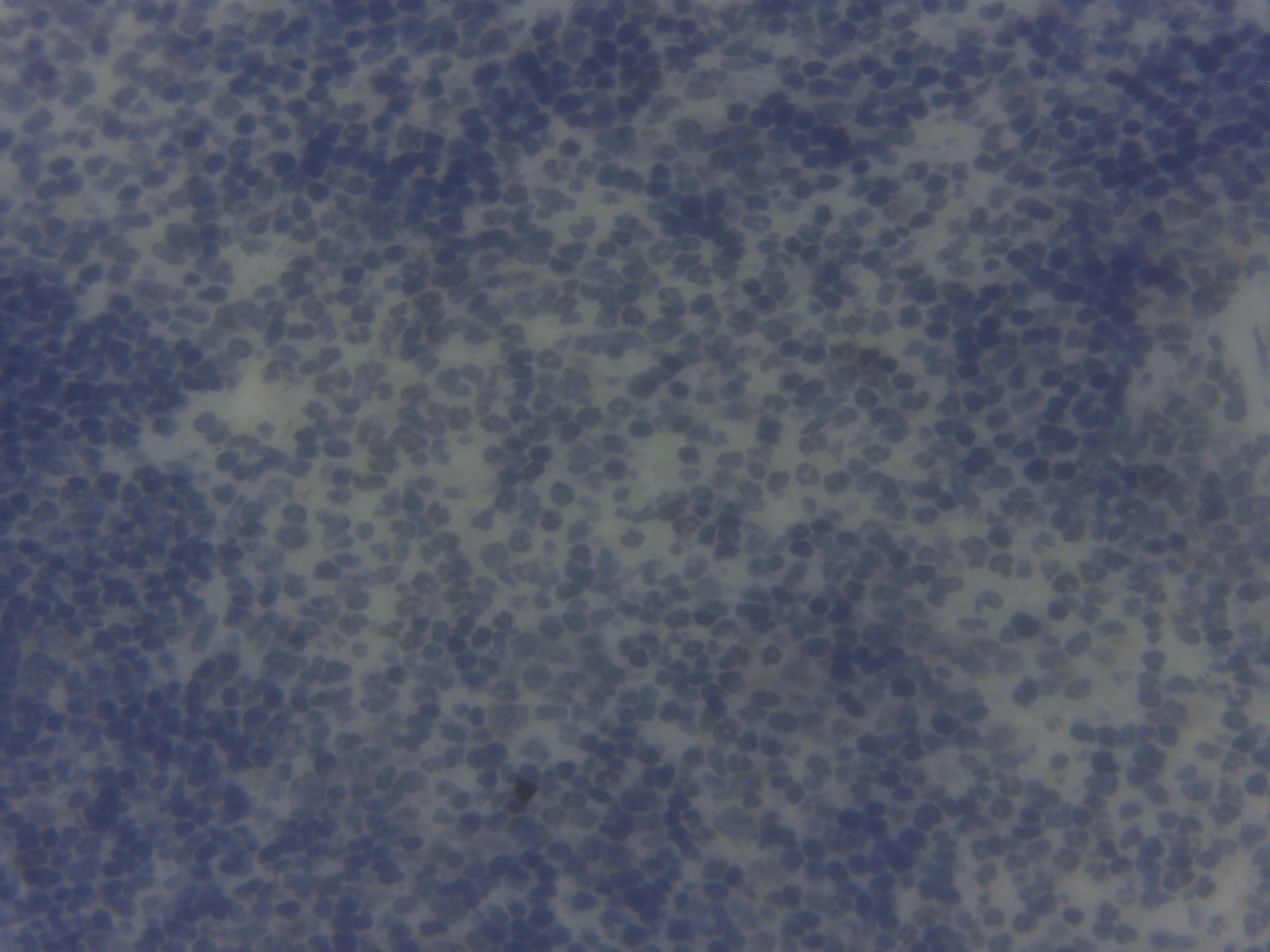

Supplement: S15 Fig — (ZIP) [file pone.0188960.s028.zip › NKp46 IHC image CON/con-6-3.jpg]

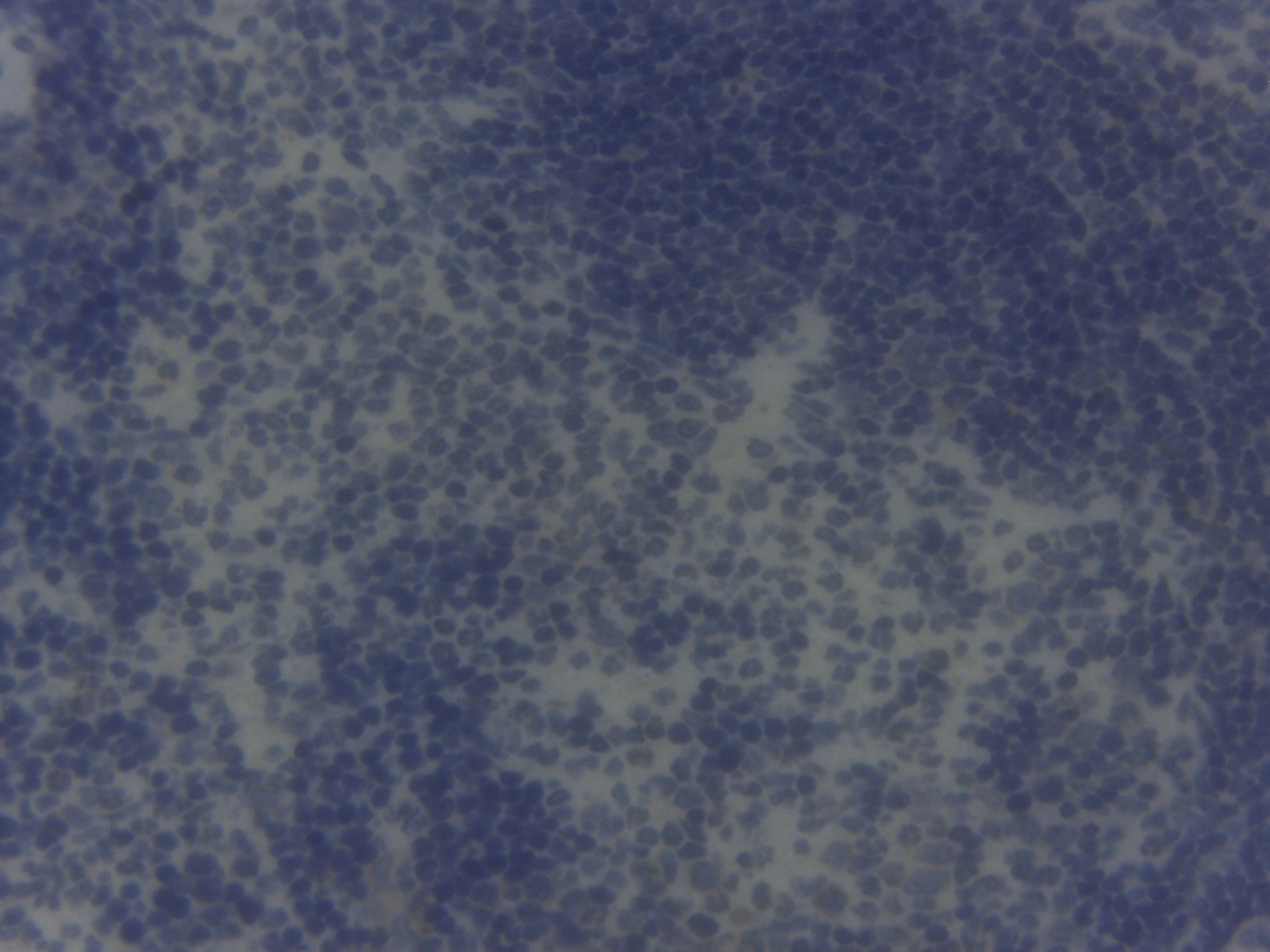

Supplement: S15 Fig — (ZIP) [file pone.0188960.s028.zip › NKp46 IHC image CON/con-6-4.jpg]

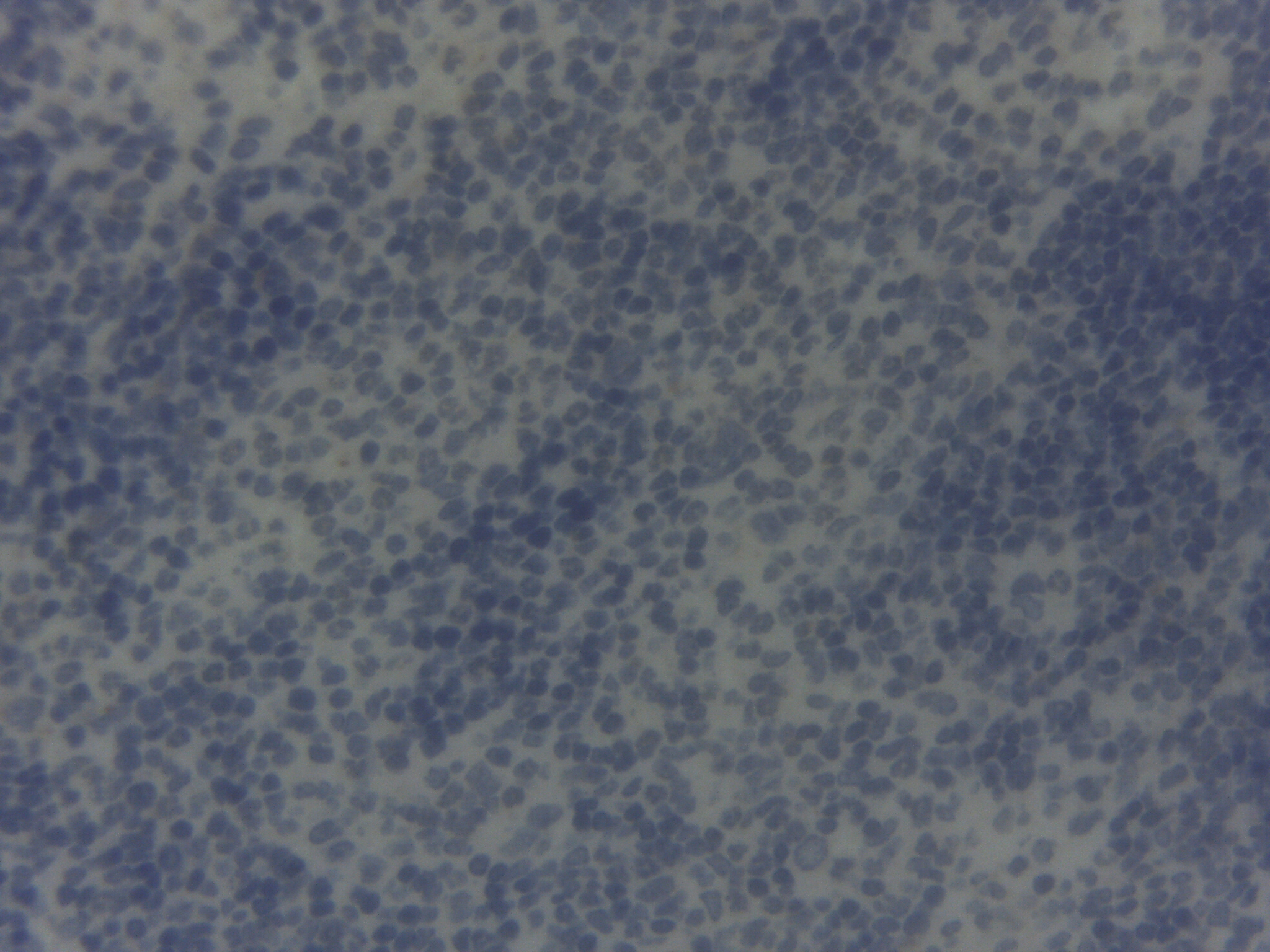

Supplement: S15 Fig — (ZIP) [file pone.0188960.s028.zip › NKp46 IHC image CON/con-6-5.jpg]
